# Origin and Evolution of Bacterial Periplasmic Force Transducers

**DOI:** 10.1093/molbev/msaf138

**Published:** 2025-06-04

**Authors:** Daniel P Williams-Jones, Melissa N Webby, James E Bray, Martin C J Maiden, Colin Kleanthous, Joanna Szczepaniak

**Affiliations:** Department of Biochemistry, University of Oxford, Oxford OX1 3QU, UK; Department of Biochemistry, University of Oxford, Oxford OX1 3QU, UK; Department of Biology, University of Oxford, Oxford OX1 3RB, UK; Department of Biology, University of Oxford, Oxford OX1 3RB, UK; Department of Biochemistry, University of Oxford, Oxford OX1 3QU, UK; Department of Biochemistry, University of Oxford, Oxford OX1 3QU, UK

**Keywords:** bacteria, molecular motors, Tol-Pal system, Ton system, bacterial cell envelope, force transduction

## Abstract

In double-membraned bacteria, non-equilibrium processes that occur at the outer membrane are typically coupled to the chemiosmotically energized inner membrane. TolA and TonB are homologous proteins which energetically couple inner membrane motor proteins to the essential processes of outer membrane stabilization and substrate import, respectively. The evolutionary trajectories of these proteins have been difficult to elucidate due to low-sequence conservation, yet they are thought to transduce force similarly. Here, this problem was addressed using structural prediction approaches to identify and annotate force transduction operons to trace their distribution and evolutionary origins. In the process, we identify a novel outer membrane-tethering system and a previously unknown family of monomeric force transducers. This approach revealed putative *tolA* genes, and thus the core organizational principles of the *tol-pal* operon throughout diverse bacterial taxa. We discovered that the α-helical structure of the periplasm-spanning domain II of TolA previously thought its hallmark, is anomalous amongst most Tol-Pal systems. This structure is mainly prevalent in γ-proteobacteria, likely in adaptation to their lifestyle. Comparison of Tol-Pal and Ton system distribution suggests that TolA emerged from a TonB paralogue and co-emerged with Pal, the outer membrane-tethering lipoprotein that functionalizes the Tol-Pal system. We also determined that TolB, the Pal-mobilizing protein, likely emerged from a family of outer membrane proteins; and CpoB, a periplasmic factor that coordinates peptidoglycan remodeling with cell division, was originally a lipoprotein present in the ancestral Tol-Pal system. The extensive conservation of the Tol-Pal system throughout Gracilicutes highlights its significance in bacterial cell biology.

## Graphical Abstract

**Graphical Abstract:**
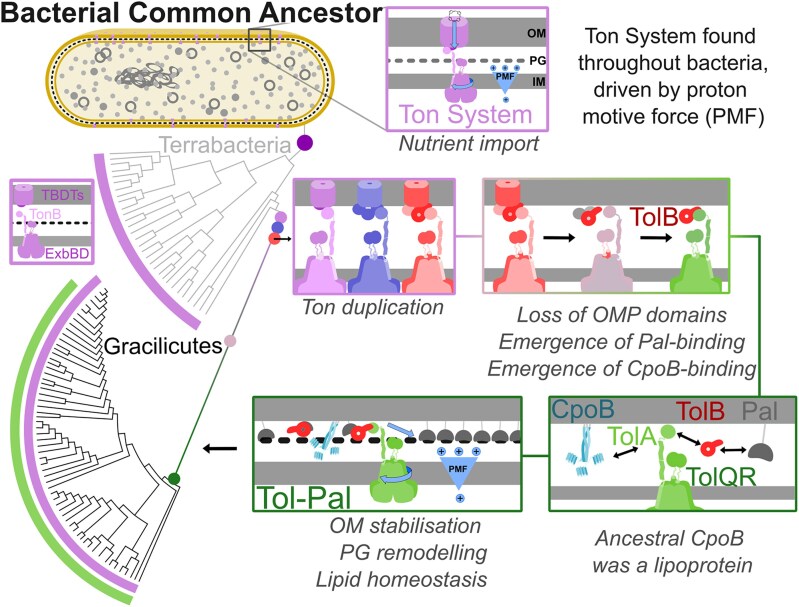

## Introduction

The cell envelope is a multilayered barrier that separates cells from their environment. Prokaryotic cell envelopes are canonically divided into diderms, featuring two membranes, and monoderms, featuring only one. The core elements of the diderm cell envelope include a semipermeable inner membrane (IM), a thin layer of peptidoglycan (PG), and a rigid outer membrane (OM). The OM plays a crucial role in cell survival, serving as a robust protective barrier that impedes the entry of large molecules into cells and is a major contributor to multidrug resistance. The OM is not energized. Hence, energized processes that occur at the OM, such as nutrient uptake, cell motility and drug efflux, must be coupled to the IM.

Many protein systems that serve as IM-to-OM energy conduits utilize a conserved transmembrane pentamer:dimer motor complex powered by the proton motive force (PMF). These motors drive a diverse range of functions at the OM, including motility (MotAB, PomAB, LafTU, AglRQS), phage defense (ZorAB), type I secretion (SiiAB), membrane stabilization (TolQR), and nutrient import (ExbBD) (Ren et al. 2005; Celia et al. 2019; Kirchweger et al. 2019; Onoue et al. 2019; Deme et al. 2020; Santiveri et al. 2020; Hennell James et al. 2021; Williams-Jones et al. 2023; Taylor et al. 2025). The latter two motors power the Tol-Pal and Ton systems, respectively. Each of these apply tensile force to the OM via a similar PMF-driven mechanism despite their distinct functional outcomes (supplementary fig. S1, Supplementary Material online). The ExbBD–TonB complex dislodges a plug domain from a variety of TBDTs in the OM to energize nutrient uptake. The genes for the Ton motor and force transducer are often found in proximity on the chromosome, but not always. In contrast, the Tol-Pal system is encoded by a seven-gene operon in *Escherichia coli*, consisting of a cytoplasmic acyl-thioesterase of unknown function (YbgC), the IM motor-transducer complex TolQRA, the OM-lipoprotein Pal and the periplasmic proteins TolB and CpoB. With the exception of YbgC, all these proteins are recruited to the septum of dividing cells to invaginate the OM through active deposition of Pal. The Tol-Pal system was also shown to stabilize the OM on the cell periphery (Tan and Chng 2025) and implicated in many other cellular functions, including lipid transport (for an in-depth review on Tol-Pal mechanism and functions, see Szczepaniak et al. (2020b) and Webby et al. (2022)).

Despite their low sequence homology, the ExbBD and TolQR motors have very similar structures with several common essential residues (Vianney et al. 1994; Cascales et al. 2001; Goemaere et al. 2007; Zhang et al. 2011; Celia et al. 2019; Webby et al. 2022). In addition, their force transduction proteins, TolA and TonB, share essential residues required for motor-binding (Karlsson et al. 1993; Germon et al. 1998). These two proteins have been shown to interact with the heterologous motor in the absence of their native motor via a conserved Ser-X-His-X-Leu-X-Ser (SHLS) motif, suggesting a conserved mode of motor-binding (Braun and Herrmann 1993; Karlsson et al. 1993; Koebnik 1993; Germon et al. 1998, 2001). Both TolA and TonB have a three-domain architecture comprising an N-terminal transmembrane helix (TMH) that associates with the motor (domain I), a central periplasm-spanning region (domain II), and a globular C-terminal domain (CTD) that binds its target via β-strand addition (domain III) (Bonsor et al. 2009; Szczepaniak et al. 2020a, 2020b). Recent cryo-EM structures demonstrate that the TMH of both TolA and TonB bind their respective motors via their SHLS motifs (Celia et al. 2024; Yeow et al. 2024).

In *E. coli* and many related γ-proteobacterial species, TolA domain II (TolAII) is predominantly α-helical (Levengood et al. 1991; Derouiche et al. 1999; Witty et al. 2002), while TonB domain II (TonBII) features proline-rich segments, expected to form Type II polyproline (PPII) helices comprising Pro-X_1-4_-Pro repeats (Evans et al. 1986; Brewer et al. 1990; Chu et al. 2007; Domingo Köhler et al. 2010). This marks the biggest structural difference between the TolQRA and ExbBD–TonB assemblies. It was recently shown that all three domains of *E. coli*'s TolA and TonB can be swapped to yield partially functional chimeric proteins, with activity determined by the identity of domain III (Williams-Jones et al. 2023). Furthermore, replacement of the structured periplasm-spanning central domain with an intrinsically disordered protein sequence abolished native function yet retained some capacity for force transduction. While highlighting the importance of secondary structure within domain II for functionality, this experimental study raises questions as to how these force transducers evolved and what the TolA/TonB common ancestor might have looked like.

Here, we use a combination of the PubMLST multispecies genome database (Jolley et al. 2018), GenBank (Benson et al. 2012), UniProtKB (Research UCJNa 2019 ), BLAST (Altschul et al. 1990), AlphaFold (AF) (Abramson et al. 2024), and FoldSeek (Van Kempen et al. 2024) to investigate the structural diversity of TolA and TonB. We first assessed the organization of *tol-pal* loci in a variety of taxa and compared the predicted secondary structures of detected TolA and TonB proteins using PsiPred (McGuffin et al. 2000; Buchan et al. 2024). This approach does not predict PPII-helices, so we also utilized PPIIPRED to predict PPII-helix propensity (O’Brien, et al. 2020). Secondary structure predictions are used here as a proxy to compare the structural composition of TolA and TonB proteins. We show that not only is the nutrient transporter TonB present in most didermal taxa, but that TolA most likely emerged after the LOL system by duplication and neofunctionalization of TonB. Moreover, we observe further speciation in the structure of TolAII within γ-proteobacteria, likely due to environmental factors and the growth requirement imposed by a decreased generation time. We utilized structure-based phylogeny to identify the emergence of TolB, which suggests it originated from the periplasmic domain of an outer membrane protein (OMP) upon loss of the OM-embedded barrel domain. We used the same approach to establish the origin of CpoB as an OM lipoprotein.

## Results

Identifying *tolA* genes within bacterial genomes is challenging because while many species have multiple *tonB* genes they typically have only a single *tolA* gene, which can only be definitively assigned through conserved genomic context (Sturgis 2001). Misannotation is common because *tolA* and *tonB* share structurally homologous CTDs and have particularly poor sequence conservation in domain II. Therefore, to perform structural analyses on a broad diversity of TolA sequences, one must first verify that these sequences belong to *bona fide tolA* genes, achieved by detecting a proximal compliment of *tol-pal* genes, such as *pal* and *tolB*. While it is known that the *tol-pal* system is “conserved” within Proteobacteria, the extent of that conservation is poorly understood. Moreover, of taxa outside Proteobacteria only Chlamydiaceae and Chlorobiota have documented *tol-pal* systems (Sturgis 2001). We sought to readdress this question by applying structural prediction approaches and modern databases to putative *tol-pal* protein sequences, allowing us to identify conserved folds and annotate those proteins despite their low total sequence conservation. This was achieved by a combination of algorithmic and manual searches (see Methods).

### Organization of the *tol-pal* Operon is Highly Conserved in Proteobacteria

**Fig. 1.**
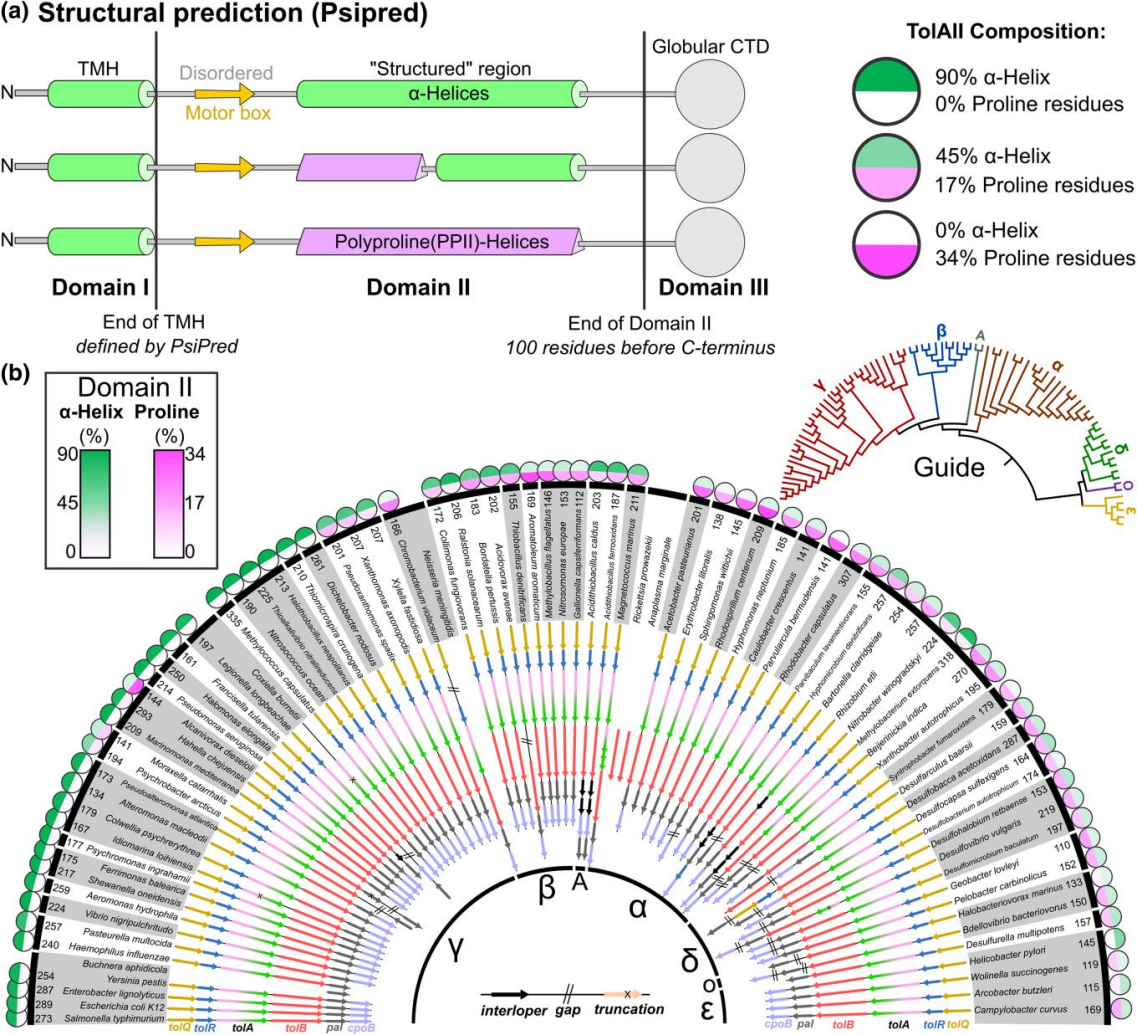
The *tol-pal* operon is widely conserved in Proteobacteria, but TolAII secondary structure varies by clade. a) TolA examples typically have a three-domain structure, where the TMH is followed by a disordered linker, an α-helical/proline-rich region, followed by the globular C-terminus (final 100 residues). TolA PsiPred predictions often show a mixture of α-helical and proline-rich regions, not defined by a specific ratio or relative position within domain II. The vast majority of TolA species feature a predicted β-strand (*motor box*) within a disordered region always found after the TMH, which is strongly suggested to interact with the dimeric component of the motor protein (Loll et al. 2024; Zinke et al. 2024). b) The *tol-pal* loci from a range of species were curated by identifying a contiguous region containing a motor, force transducer, *tolB* and *pal*. Guide tree (*top right*) adapted from Sharma et al. (Sharma et al. 2022) (supplementary fig. S1.2, Supplementary Material online). “A” denotes Acidothiobacillia, while “O” denotes Oligoflexia. TolA secondary structures were predicted using PsiPred, and the α-helix:proline ratios calculated (McGuffin et al. 2000) (supplementary SI S1, Supplementary Material online). These ratios are represented in colored outer circles. Circumferential numbers indicate respective length of domain II. Some species have putative “split” *tol-pal* systems (denoted by “//”), where different components are not proximal on the genome. Evidence for loci and TolAII composition analysis indicated in supplementary SI S1, Supplementary Material online.

To find the limits of secondary structure diversity in TolA domain II, a library of representative TolA protein sequences was required. It is known that the Tol-Pal system is generally conserved in Proteobacteria (Sturgis 2001), thus we manually searched for *tol-pal* operons in representative species selected in a consensus phylogeny tree of Proteobacteria (Sharma et al. 2022). Following identification of *tol-pal* loci, each TolA sequence was structurally predicted using PsiPred 3.0 (McGuffin et al. 2000). From these secondary structural predictions, the proportion of domain II residues predicted to form α-helices was quantified and compared with the proportion of proline residues within domain II, reflective of PPII-helix propensity (Fig. 1a). The propensity of each sequence for PPII-helix formation was also predicted using PPIIPRED, and is indicated for each protein (supplementary SI S1, Supplementary Material online) (O’Brien, et al. 2020). To demonstrate that the transducer analyzed in each case was TolA, the corresponding *tol-pal* loci were aligned accordingly (Fig. 1b).

This catalog of proteobacterial *tol-pal* loci builds on the work of Sturgis (Sturgis 2001), where curation of the corresponding genomic loci indicates that the force transducer analyzed is TolA (and not TonB). Notably, these loci demonstrate considerable conservation of core gene organization/synteny, with most species demonstrating a similar organization to that of *E. coli.* In some cases, “split” *tol-pal* loci were observed, where *tolQRAB* and *pal-cpoB* gene clusters were located distally on the genome, in species such as *P. arcticus*, *L. longbeachae, and S. fumaraoxidans.* In *A. avenae*, there is a split system where *tolQRA* is distal from *tolB-pal-cpoB*. Overall, these outliers suggest that while the traditional “core” locus organization is a suitable guide for *tol-pal* system detection, there are exceptions. Concomitant with previous observations, species completely lacking *tol-pal* loci such as *B. aphidicola, N. meningitidis,* and *Ricksettia/Anaplasma* exhibit intracellular lifestyles (Hardy et al. 2000; Sturgis 2001; Braendle et al. 2003; Perotti et al. 2006; Rymaszewska and Grenda 2008). This suggests that loss of the *tol-pal* system occurs during genome reduction, potentially a consequence of reducing both OM complexity and antigenicity as a protective measure within host cells. The vesiculation phenotype caused by loss of *tol-pal* genes has also been proposed to arm and protect intracellular species from immune responses (Amano et al. 2010; Cecil et al. 2019).

The flanking genes of each *tol-pal* cluster were semi-synthetic but varied between proteobacterial classes, suggestive of positional changes on the chromosome over time (supplementary fig. S1.3, Supplementary Material online). Prevalent conserved upstream genes include *ybgC*, encoding a thioacyl-esterase (γ/β/α-proteobacteria and Acidothiobacillia), *ruvABC* encoding DNA-helicases involved in homologous recombination (γ/β/α-proteobacteria and Acidothiobacillia), and *atpC* encoding ATP synthase subunit C (ε-proteobacteria). Common downstream genes included tRNA synthases and *queEC*, involved in queuosine synthesis for tRNAs (both in γ/β/α-proteobacteria and Acidothiobacillia), and *slyD*, encoding an FKBP-type peptidyl-prolyl cis-trans isomerase (ε-proteobacteria). Crucially, *cpoB* was found in 86% of all proteobacterial species with detectable *tol-pal* systems and directly adjacent to other *tol-pal* genes in 92% of those systems (supplementary SI S1, Supplementary Material online). SignalP 6.0 analysis suggested that 20% of the 70 identified proteobacterial CpoB sequences were OM lipoproteins (supplementary fig. S1.4, Supplementary Material online) (Teufel et al. 2022).

**Fig. 2.**
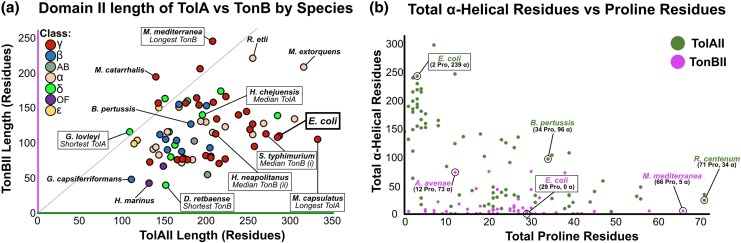
Quantification of TolAII/TonBII structural diversity. a) The total length of domain II for selected TolA and TonB sequences is plotted here by species (supplementary SI S1 to S2, Supplementary Material online). Each point is colored by proteobacterial class (“*AB*”= *Acidothiobacillia*, “*OF*”= *Oligoflexia*). A Pearson's correlation test suggests that the length of TolAII and TonBII is weakly positively correlated in each species (Score = 0.28). These data show that lengths of both TolAII and TonBII also vary greatly within proteobacterial classes, but the majority of TolAII sequences are longer than TonBII in any given species (*below grey line*). Species of note are indicated, to illustrate the variation in TolAII/TonBII length. Two TonB medians were observed (*i/ii*). b) For each selected TolAII and TonBII sequence, the total number of α-helical residues is plotted against the total number of proline residues. Many TolA sequences are α-helix dominant (e.g. *E. coli*), but many mixed (e.g. *B. pertussis*) and proline-dominant sequences (*R. centenum*) are also observed. For TonB sequences, the majority are proline-dominant or mixed, but some α-helix dominant species are present (e.g. *A. avenae*). Note that proline residues are a minimal proxy for the number of PPII-helical residues present.

Structural analyses suggest that the α-helical dominance observed in γ-proteobacterial TolAII is not the case in other proteobacterial taxa, as shown by the spectrum of helix-rich, proline-rich and mixed secondary structures across the proteobacterial tree (Fig. 1b). The same structural composition analysis was thus performed on TonB sequences detected within the same genome accessions, to compare the structural diversity of TonBII. To verify that these were *tonB* genes, neighboring genes were inspected to ensure that no *tolB/pal* genes were present. Many species feature multiple *tonB* genes, but only one was analyzed per species, thus those selected may structurally differ from alternative TonB paralogues. In the case of multiple *tonB* paralogues in a species, the TonB sequence selected was the first detected that contains all three domains and was not previously identified as TolA. The calculated TonBII α-helix:proline ratios were then mapped to the same phylogenetic tree (supplementary fig. S2.1, Supplementary Material online) (Sharma et al. 2022). These data demonstrate that in Proteobacteria, the majority of TonBII sequences are proline-rich, but α-helical and disordered TonBII sequences were also detected (supplementary SI S2, Supplementary Material online). This analysis implies that the synthetic *E. coli* domain II chimeras generated previously (Williams-Jones et al. 2023), are not as unusual as suspected because many proline-rich and disordered TolA sequences exist in nature, as do examples of TonBII featuring α-helices (supplementary SI S1 to S2, Supplementary Material online). Further investigation of TonBII sequences revealed that the two oppositely charged PPII-helix segments observed in *E. coli* are only found in TonBII sequences from Enterobacterales, Pasteurellales, and *B. pertussis*. Many Proteobacteria feature more neutral PPII-helix motifs with a preponderance for alanine and valine residues (e.g. *N. oceani* or *C. violaceum*), or entirely negative PPII-helices (glutamate-rich, e.g. *M. mediterranea*). TonBII PPII-helices also varied in organization, with most featuring Pro-X_1-4_-Pro motifs, but some with long homopolymeric stretches of proline residues alone (e.g. *X. axonopodis*, *T. denitrificans,* and *C. crescentus*). The propensity of each TonBII sequence for PPII-helix formation was also predicted using PPIIPRED, and is indicated for each protein (supplementary SI S2, Supplementary Material online) (O’Brien, et al. 2020). The length of TonBII was also variable; some species feature very short TonBII sequences (e.g. 40 residues in *D. retbaense*) while the longest observed was 245 residues (*M. mediterranea*). In contrast, TolAII ranges between 110 and 335 residues. The median TonBII and TolAII lengths were 113 and 192 residues, respectively. The respective lengths of TolAII and TonBII per species are weakly positively correlated (Fig. 2). This may be linked to the width of the periplasm in each species, but few measurements of bacterial periplasms are available to test this.

These data reiterate that the function of TolA and TonB are indistinguishable from sequence and structural predictions alone; TolAII can be short and PPII-helix dominant, TonBII can be long and α-helix dominant, and both proteins often feature mixtures of both helix types. Protein function must therefore be inferred from genomic context. The close resemblance of some of these proteins reiterates the question of their evolutionary relationship. To contextualize these data and address these questions, a broader sample of bacterial *tol-pal* systems must first be validated.

### TolAII α-helicity in Proteobacteria is Anomalous Amidst Wider Bacterial Diversity

**Fig. 3.**
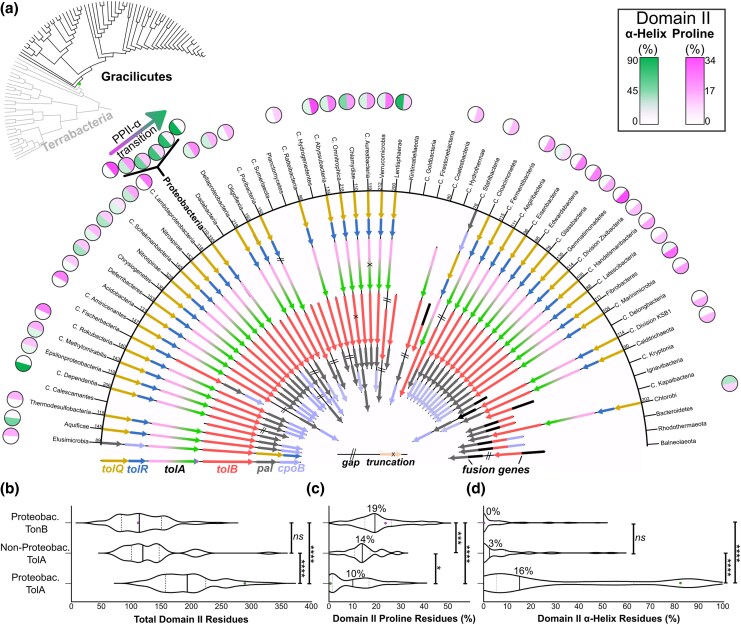
*tol-pal* gene organization and α-helix:proline ratios of selected gracilicute species. a) Gracilicutes with identified *tol-pal* loci display similar gene organizations (*arrows*) and TolAII structural composition analysis (*outer circles*). This alignment is derived from portion of the phylogenetic tree published by Witwinowski et al*.,* and annotated with those taxa (*black branches of guide tree, top left)* (Witwinowski et al. 2022). The emergence of Pal is indicated on the guide tree (*green point*). The α-helix:proline ratio of representative species TolA are indicated, with full *tol-pal* loci and analysis of those species analyzed in supplementary SI S1, Supplementary Material online. Fusion genes indicated by multi-colored arrows. Circumferential figures indicate the number of residues found in TolAII. “PPII-α Transition” refers to the increased representation of α-helix-rich TolA sequences within Proteobacterial taxa. Uncultured taxa are indicated by Candidatus (“*C.”*). b) The distribution of Gracilicute TolAII lengths more closely resembles proteobacterial TonBII than TolAII. The median lengths (*solid bars*) and upper/lower quartiles (*dashed bars*) are indicated. A Mann–Whitney *U* test suggests that proteobacterial TonB and Gracilicute TolA domain II length distributions do not differ significantly (P = 0.1487), while proteobacterial TolAII length distributions differ significantly from both (P < 0.0001). c) The distribution of Gracilicute TolAII sequences features a significantly lower proportion of proline residues than proteobacterial TonBII sequences (P = 0.0003), but greater than proteobacterial TolAII sequences (P = 0.013). d) The distribution of Gracilicute TolAII sequences indicates a similar proportion of α-helical residues to proteobacterial TonBII sequences (P = 0.2704), both of which are significantly lower in α-helical residues than proteobacterial TolAII sequences (P < 0.0001). For both b) and c), median values are labeled. For panels b–d), values for *E. coli* TolA (*green*) and TonB (*pink*) are shown.

To broaden the catalogue TolAII structural analysis beyond proteobacterial lineages, we used a combination of manual locus curation and a search algorithm to identify *tol-pal* loci in the PubMLST Multispecies genome database (Jolley et al. 2018). The algorithm detects *tol-pal* constituent genes by PFAM annotations, then measures their relative proximity. These data were inspected to curate the correct complement of genes for each species, minimally comprising a motor, force transducer, *tolB* and *pal* (supplementary SI S1, Supplementary Material online). Recent work by Witwinowski et al., traced the evolutionary emergence of multiple membrane PG tethering proteins, including Pal (Witwinowski et al. 2022). We adapted the *pal-*containing branch of their phylogenetic tree to catalogue *tol-pal* locus organization and TolAII composition (Fig. 3). These data suggest that the α-helical bias of γ-proteobacterial TolAII is anomalous amongst the more evolutionarily distant bacterial taxa.

A similar analysis performed for a representative TonB from each proteobacterial species demonstrated that the vast majority of TonB sequences are also proline-rich (supplementary SI S2, Supplementary Material online; supplementary fig. S2.1, Supplementary Material online). Non-proteobacterial TolA (henceforth described interchangeably with “Gracilicute TolA”) domain II sequences are generally shorter than proteobacterial TolAII sequences, more akin to proteobacterial TonBII sequences (Fig. 3b), with further analysis revealing that the total number of α-helical residues versus proline residues also resembles the distribution of proteobacterial TonBII sequences more closely than proteobacterial TolAII sequences (Fig. 3c and d). This likely arises because α-helices are more compact than PPII-helices, thus more residues are required for equivalent reach through the periplasm. The apparent structural similarity between non-proteobacterial TolAII and proteobacterial TonBII sequence distributions reveals putative proline-rich TolA sequences that are virtually indistinguishable from TonB. Since the majority of evolutionarily distant TolA sequences sampled are proline-rich, including the early diverging (basal) lineage of Elusimicrobia, this suggests that the common ancestor of TolA was also proline-rich. This reiterates the question of which emerged first, TolA or TonB?

The structural analysis of each TolA protein also revealed that every TolA features a disordered region immediately after the TMH (supplementary SI S1, Supplementary Material online). Moreover, in the 113 validated TolA sequences from both proteobacterial and Gracilicute datasets, 93% feature a predicted β-strand within this disordered region, described here as the motor box (Loll et al. 2024; Zinke et al. 2024). The functional importance of the motor box in the force transduction mechanism of TonB has been investigated in recent work (Loll et al. 2024; Zinke et al. 2024), which suggests it plays a role in the activation of the motor-driven force transduction cycle of TonB. Further analysis suggests the motor box is found in all TonBs (supplementary SI S2, Supplementary Material online). The extensive conservation of this motif within TolA and TonB alike suggests that TolA and TonB utilize a common force transduction mechanism, and that this motif was a feature of their common ancestor.

### A New Class of Motor-driven Force Transducers

**Fig. 4.**
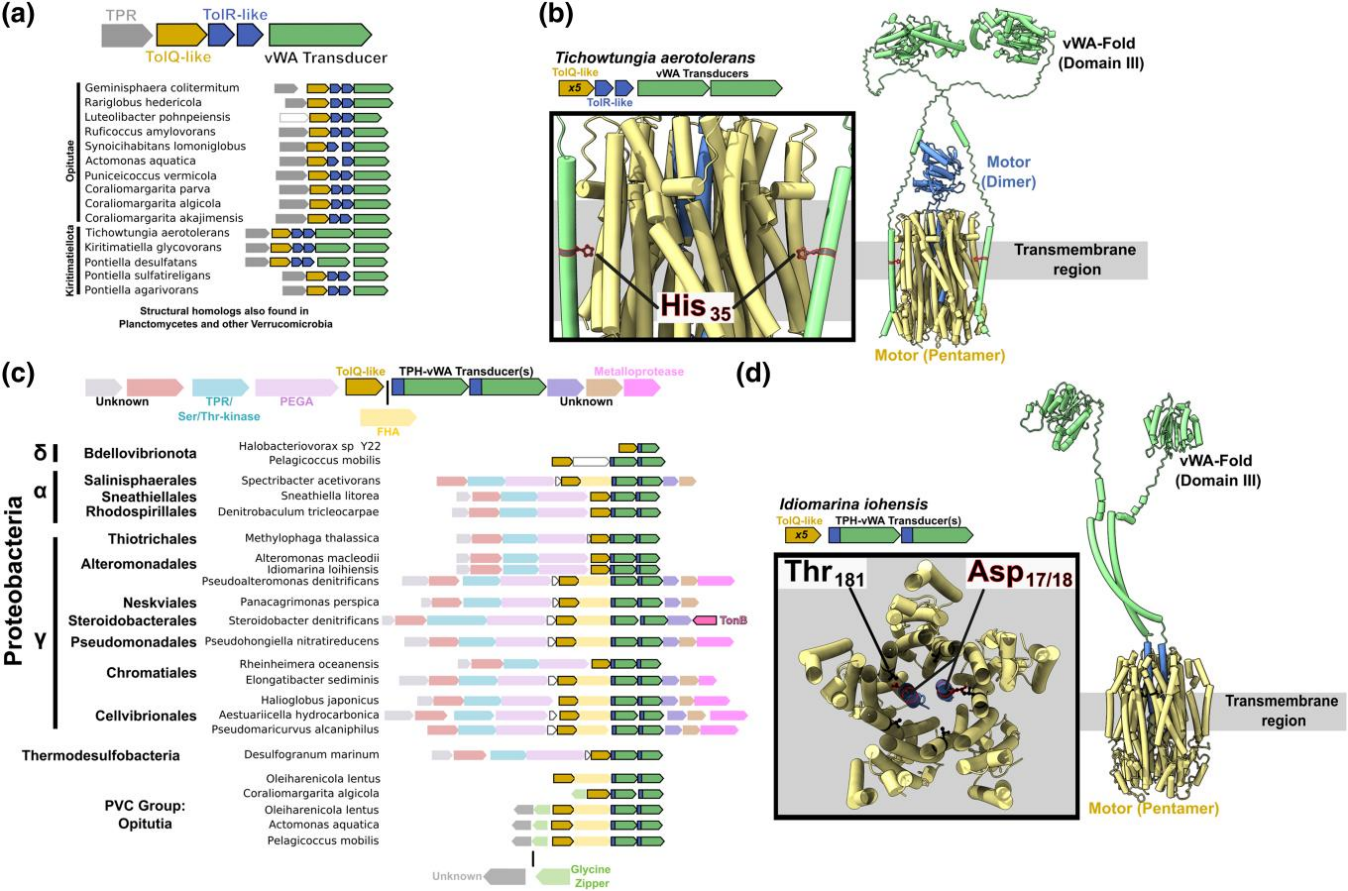
vWA proteins may transduce force via motor coupling. a) Species featuring homologous vWA genes adjacent to *tolQR*-like motor genes, detected using WebFlags by homology to the *Tichowtungia* vWA transducer family (Saha et al. 2020). An uncharacterized tetratricopeptide repeat (TPR) gene was found to co-occur with the motor-vWA genes, suggesting interaction. b) AF3 prediction of vWA proteins with a TolA/TonB-like topology suggest they may transduce force in a similar manner to TolA/TonB, by binding the edge of the motor complex. Notably, these vWA transducer protein TMHs possess the conserved SHLS motif and histidine (*His_35_*) that is essential to motor interactions formed by TolA and TonB. c) Species featuring vWA genes adjacent to *tolQ*-like motor genes only. Gene clusters shown sample the top 200 homologs of *Idiomarina iohensis* as detected by WebFlags (Saha et al. 2020). No *tolR/exbD/motB*-like genes were detected. d) This family of vWA proteins may form an obligate complex with TolQ-like pentamers via a TPH (*blue*). Of note, a conserved pair of aspartate residues (*one per vWA monomer*) are predicted to be coordinated by a conserved ring of threonine residues, an essential interaction conserved in MotA-MotB, ExbB-ExbD and TolQ-TolR. The TPH may rotate within the pentamer to directly couple PMF to the application of tensile force at the OM. AF3 models with plDDT and PAE scores are indicated in supplementary fig. S1.7, Supplementary Material online.

Occasionally, representative species lacked discernible TolA and TonB homologs, e.g. *Tichowtungia aerotolerans.* In such cases, we searched for homologs of motor genes, and assessed neighboring genes for structural homology to TolA/TonB. A family of proteins were identified that resemble TolA/TonB with a three-domain structure comprising a TMH, a proline-rich/disordered linker, and a globular CTD. Many of these proposed force transducers feature a TMH with the conserved SHLS motif, followed by a disordered region with a predicted motor box, and a domain II of approximately 100 residues. However, in these proteins the CTD in question bears no homology to the TolA/TonB-CTD, instead possessing a von Willebrand Factor A (vWA) fold. This fold resembles the free periplasmic YfbK protein from *E. coli*, which comprises two Rossmann-folds. The vWA fold is best known to be involved in adhesion and protein-protein interactions, most notably metazoan hemostasis (Gerke 2011). In bacteria, secreted proteins such as AglZ feature the vWA fold, which forms interactions that are essential for gliding motility in *Myxococcus xanthus* (Islam et al. 2023). Our analysis suggests these newly-identified IM-tethered vWA proteins function in the same way as TolA/TonB to transduce force. This novel family of vWA force transducers was found throughout Proteobacteria, Planctomycetes, Verrucomicrobia, Bacteroidetes, and Rhodothermaota. Their function remains unknown (no phenotypes of deletions have yet been reported), but within the PVC group these genes are often found downstream of a TPR gene, a *tolQ-like* gene and a pair of heterologous *tolR*-like genes (Fig. 4a) (Saha et al. 2020). When these adjacent proteins are structurally modeled (Fig. 4b), they are predicted to form a complex where the transducers' TMH interacts with the same site on the motor via a conserved His residue, precisely as observed within ExbBD–TonB and TolQRA cryo-EM structures (Celia et al. 2024). Their synteny with motor genes and conserved motor-binding site suggest that they are novel PMF-driven proteins, perhaps also involved in gliding motility by fulfilling an AlgZ-like function. Moreover, a second family of TMH-tethered vWA homologs were found to co-occur extremely tightly with *tolQ* homologs, with no co-occurring *tolR/exbD* genes (Fig. 4c). The lack of syntenic *tolR/exbD* homologs is highly atypical amongst this family of motor proteins, suggesting these vWA genes may comprise the dimeric partner of the motor complex. The vWA proteins lack the SHLS motif residues involved in motor interactions and are instead predicted to possess a TolR/ExbD-like transpore helix (TPH) that inserts directly into the TolQ-like pentamer. Notably, the TPHs contain the conserved proton-binding aspartate residue that is essential in MotB/ExbD/TolR (Fig. 4d). Unlike the former vWA transducer family, these TPH-vWA genes were predominantly found in heterologous pairs. These genes were more widely detected, predominantly throughout Proteobacteria and Verrucomicrobia. Within their AF3 structural predictions, the conserved TPH aspartate residues are surrounded by the conserved ring of essential threonine residues lining the lumen of the pentamer, as also seen for MotA/ExbB/TolQ. These data thus suggest that vWA proteins transduce force by rotating directly within the pentamer, rather than attaching at the side as seen for TolA/TonB (Celia et al. 2024). Overall, these putative force-transducing proteins highlight the diversity of trans-envelope systems energized by PMF-driven 5:2 motors that remain to be discovered.

### Structural Phylogeny of TolB Relatives Suggests an OMP-TolB-like Ancestor

**Fig. 5.**
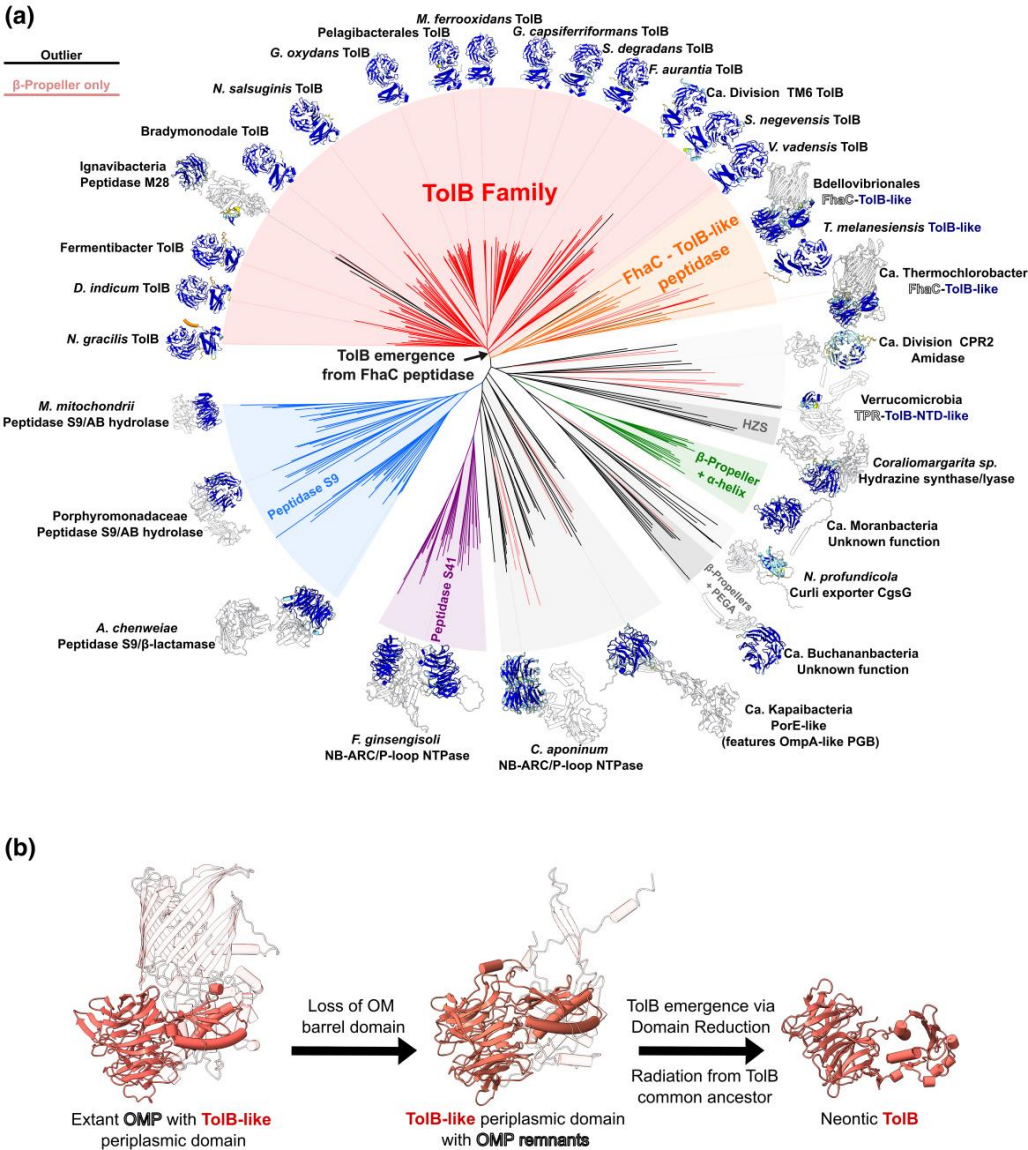
Structural phylogeny of TolB-related protein models suggests TolB emerged from an OMP-TolB-like family. a) Unrooted Foldtree tree reveals clades comprising peptidases, mixed enzymatic functions, small β-propeller proteins featuring a single α-helix, OMPs with a TolB-like periplasmic domain and TolB, and many proteins comprising only a β-propeller. Decorating AF models are colored by plDDT. Annotated TolB query results and UniProt/AF accessions are available in SS1. The phylogenic distribution of TolB-like OMPs is wider than TolB (supplementary fig. S1.6, Supplementary Material online). FoldTree outputs (featuring UniProt/AF accessions), and TolB sequences/alignment/hmm profile are available in SI 3. b) Structural similarities suggest a route for TolB emergence. The OMP-TolB-like ancestor of TolB may resemble the extant example shown from Bdellovibrionales (*A0A1F3SUA3*), with loss of the OM-barrel leading to an ancestor resembling an extant TolB-like protein (*Balneolaceae shown; A0A356X2S7*), followed by domain loss to the TolB common ancestor, to modern (*“neontic”*) TolB (*E. coli shown; P0A855*).

The origin of *pal* has been described previously, by fusion of the *ompA* PG-binding domain with a lipoprotein secretion signal, after the emergence of the LOL pathway (Witwinowski et al. 2022). In contrast, the origin of TolB, which binds and mobilizes Pal, remains unresolved. TolB has a two-domain structure, where the N-terminus adopts a Rossman-fold (near the N-terminal TolA-binding region), and the C-terminus forms a 6-bladed β-propeller (the “WD40 fold”). To assess the distribution and emergence of TolB, the phylogenetically-balanced taxid database constructed by Witwinowski *et. al*., was queried for TolB-like proteins using the curated TolB sequences (Witwinowski et al. 2022). The identified proteins with UniProt-embedded AF models were analyzed using FoldTree, to ascertain the structural phylogeny of the different TolB-like families while overcoming their low sequence homology (UniProt Consortium 2022; Barrio-Hernandez et al. 2023; Moi et al. 2023). This revealed a closely-related clade of OMPs with a TolB-like periplasmic domain featuring both six-bladed β-propellers and an adjacent Rossmann-fold (Fig. 5a). Notably, many of these OMPs are automatically annotated as TolB. Analysis of the OMP-TolB-like protein AF clusters suggests examples are distributed throughout both Terrabacteria and Gracilicutes, with varying degrees of β-barrel loss (supplementary fig. S1.6, Supplementary Material online) and revealed putative extant structural intermediates between an OMP-TolB-like protein and neontic (modern) TolB (Fig. 5b). The analysis also revealed two TolB-like proteins that may have emerged particularly early, convergently, or horizontally in Thermotogae, a taxon that lacks Pal.

### A Putative OM-PG Tethering System in Bacteroidetes

**Fig. 6.**
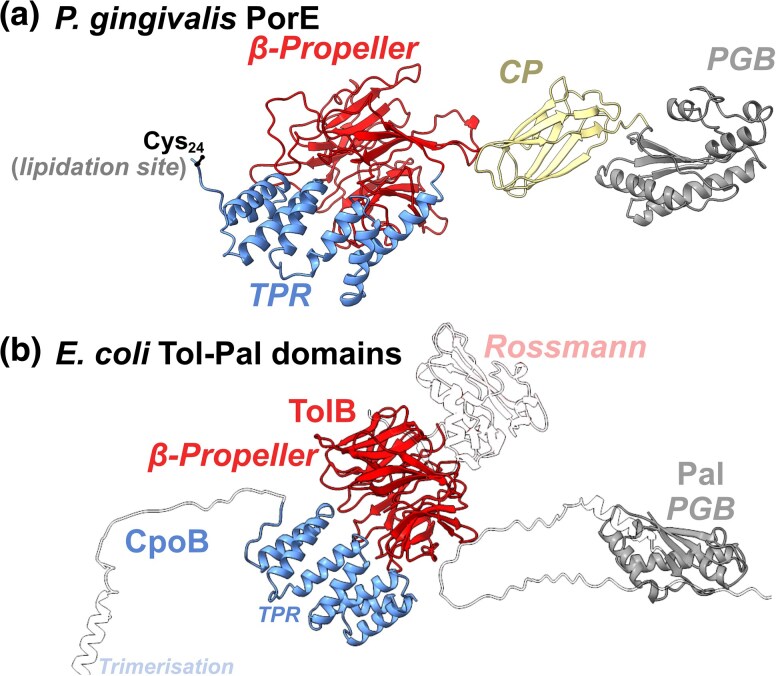
PorE is a putative multi-domain OM-lipoprotein comprising CpoB, TolB, carboxypeptidase and pal subdomains. a) AF3 model of *P. gingivalis* PorE, with labeled subdomains. b) Three of the core six Tol-Pal proteins exhibit subdomains with structural homology to PorE (Research UCJNa 2019; Jumper et al. 2021). Crucially, PorE resembles Pal with the inclusion of TolB/CpoB/CPE resident proteins, suggesting a Pal-adjacent OM-PG tethering role.

During the analysis of Gracilicute genomes, a lack of identifiable *tol-pal* loci in the Bacteroidetes prompted deeper investigation. In the majority of Gracilicute taxa analyzed, *pal* genes were detected, comprising an OmpA-like PG-binding (PGB) domain tethered to the OM at an N-terminal lipidation sequence. No Bacteroidetes *pal* genes were detected in the genome database of Witwinowski et al., though multiple Ton systems have been detected in model species such as *Bacteroides thetaiotaomicron* (Witwinowski et al. 2022; Pollet et al. 2023). Putative TolB-Pal fusion proteins were detected through use of Foldseek, suggesting that in some taxa the Tol-Pal components are combined (supplementary SI S1, Supplementary Material online) (Jumper et al. 2021; Van Kempen et al. 2024). Moreover, a putative multi-domain fusion lipoprotein of *cpoB-tolB-pal* was also detected throughout Bacteroidetes and Rhodothermaota (Fig. 6; supplementary SI S1, Supplementary Material online). This was identified as PorE, recognized as an essential component of the Type 9 Secretion System (T9SS) in *Porphyromonas gingivalis*, and required for gliding motility in *Flavobacterium johnsoniae* (Gorasia et al. 2022), where *porE* is present in multiple copies on the chromosome. This protein also features a carboxypeptidase (CP) domain with an Ig-like fold, akin to the periplasmic N-terminal extension that is necessary for many Bacteroidetes TBDTs to function (Gray et al. 2021; Pollet et al. 2021). In *Bacteroides thetaiotamicron*, the CP domain is implicated in the interaction of TBDTs with TonB (Pollet et al. 2021), suggesting that PorE may utilize force transduction via a TolA/TonB-like protein. The presence of the three periplasmic *tol-pal* protein folds genetically fused within *porE* suggests PorE may fulfill the OM-stabilizing and/or PG-remodeling roles of the *tol-pal* system, in addition to its association with the T9SS.

It is unclear whether PorE plays a role in the stabilization of the OM in Bacteroidetes during cell division, but it appears structurally related to the *tol-pal* system. CpoB, which we identify is likely an OM-lipoprotein in most non-proteobacteria, contains a tetratricopeptide repeat (TPR) motif. This domain typically serves as a hub for protein-protein interactions, such as those with the T9SS. Moreover, PorE topologically resembles Pal sufficiently, with its N-terminal lipidation and C-terminal PGB, to fulfill a similar OM-PG tethering role in vivo. Further, *porE* deletions were shown to impact T9SS-mediated gingipain-secretion (Heath et al. 2016), from which it was concluded that PorE is a component of the T9SS. If PorE plays an OM-PG tethering role as predicted here, then it is plausible that these secretion defects are a consequence of a destabilized OM, in line with observations that loss of *tol-pal* genes abolishes motility in species such as *Pseudomonas putida, E. coli* and *Shewanella oneidensis* (Llamas et al. 2000; Gao et al. 2017; Hirakawa et al. 2019), and abolishes the function of type III secretion systems in enterohemorrhagic *E. coli* and *Citrobacter rodentium* alike (Hirakawa et al. 2020). Further evidence that PorE plays a role in OM-stabilization is presented via its co-occurrence with *queEC* genes, which are often syntenic with proteobacterial *tol-pal* operons (supplementary fig. S1.4, Supplementary Material online). The *P. gingivalis* PorE protein has been shown to bind PG in vitro (Heath et al. 2016). In contrast to other T9SS-related genes, *porE* is not found within the T9SS operon in *P. gingivalis*, suggesting that PorE may not be directly linked to the T9SS. Examining the localization of this protein throughout the cell cycle and its role in OM-stability may help define the role of PorE. As the PorE protein possesses three conserved periplasmic Tol-Pal domains, Pal, TolB and CpoB in tandem, PorE may reflect an extremely speciated/compact Tol-Pal system. We therefore propose that counter to its assumed role as a T9SS accessory protein, PorE may facilitate OM-PG tethering in the Bacteroidetes and potentially undergoes septal trafficking. Identification of this proposed OM-PG tethering protein suggests that other similar OM-stabilizing systems remain to be revealed.

### The Ton System Existed in the Last Common Bacterial Ancestor

**Fig. 7.**
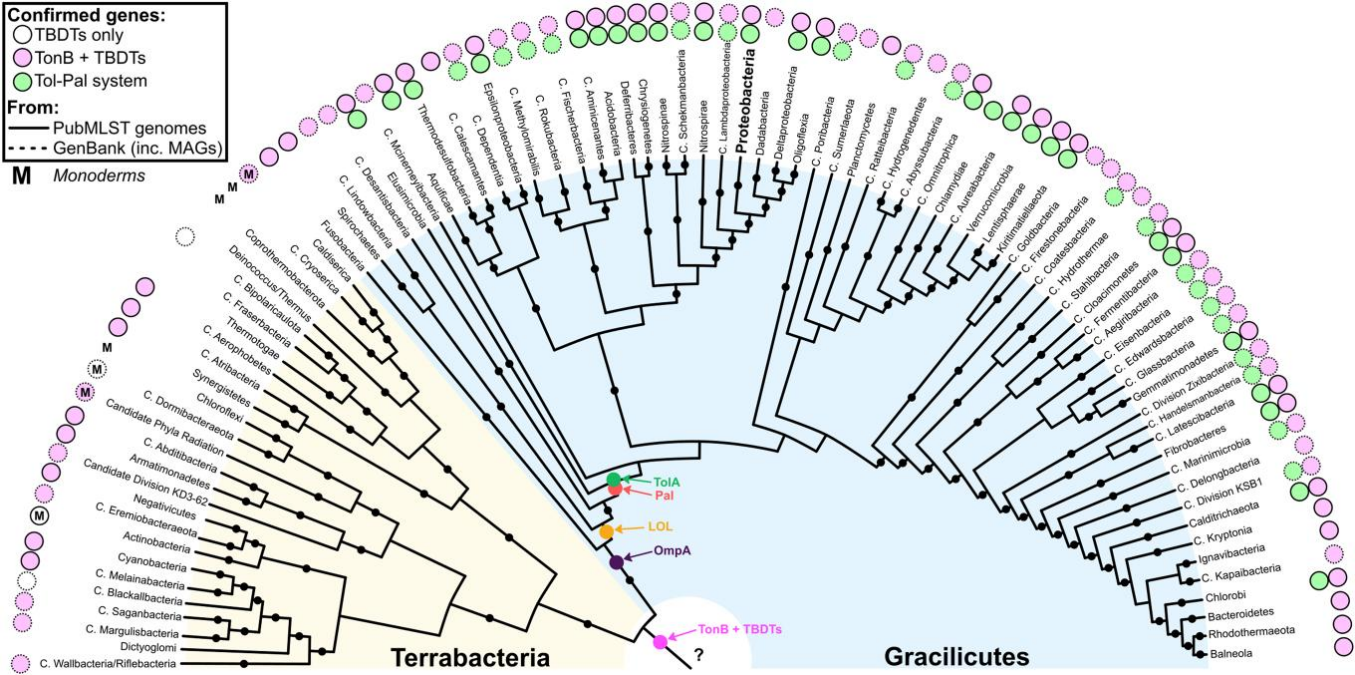
Distribution of tol and ton systems suggest ancestral TolA was a TonB paralogue. Taxa featuring curated Tol-Pal systems are found within Gracilicutes only, whereas most didermal species across both Terrabacteria and Gracilicutes feature Ton systems. This suggests that the LBCA possessed a functional Ton system (*pink arrow*). The most likely point of TolA emergence (*green arrow*) occurs near the origin of Pal proposed previously (Witwinowski et al. 2022). Phylogenetic tree adapted from Witwinowski et al. (Witwinowski et al. 2022). Taxa are predominantly didermal unless noted as monoderms (*“M”*). Of the Firmicutes, Negativicutes are the only diderms where Ton systems were detected. In Actinobacteria, a few species feature TBDT genes, but only in monodermal clades where they are likely non-functional (Beaud Benyahia et al. 2025). Species flagged for Ton systems are noted in SS1, as well as those identified from GenBank accessions that include metagenome assembled genomes (*MAGs*). Uncultured taxa are indicated by Candidatus (“*C.”*).

The preponderance of TolAII structures with little to no α-helical content were previously unknown, revealing that these proline-rich TolA proteins are structurally indistinguishable from TonB, and previous analyses detected TonB only in Proteobacteria, Cyanobacteria, Bacteroidetes, Chlorobi, Chlamydiae, Verrucomicrobia, Fusobacteria, Atribacteria, diderm Firmicutes and Armatimonadetes (Chu et al. 2007; Mirus et al. 2009; Dodsworth et al. 2013; Hu et al. 2014; Poppleton et al. 2017). This prompted a parallel investigation into the distribution of species featuring at least the Ton system. An algorithm was thus used to search for *tonB* and *TBDT* genes within the curated PubMLST genome database, which were then manually curated (supplementary SI S2, Supplementary Material online; supplementary SS1, Supplementary Material online). Unlike *tolA*, *tonB* exhibits infrequent co-occurrence with its motor (*exbB-exbD)* and can be randomly distributed or near TBDT genes. Mapping these data to the Witwinowski tree revealed that Ton systems are far more widely distributed than Tol-Pal systems, suggesting that the Ton system evolutionarily predates the Tol-Pal system (Fig. 7) (Witwinowski et al. 2022). Many terrabacterial taxa feature complex cell envelopes, where the OM was lost, and then subsequently re-emerged by convergent evolution, often with a drastically different composition. For a comprehensive review of terrabacterial OM composition and evolution see Beaud Benyahia et al. 2025 and Hashimi and Tocheva 2024 (Hashimi and Tocheva 2024; Beaud Benyahia et al. 2025). In monoderms, complete Ton systems (in Clostridia) and incomplete Ton systems (in Actinobacteria, Bacilli, Caldiserica, and Dormibacteria) were occasionally observed. These are likely non-functional, and it is unknown whether they were acquired through horizontal gene transfer or remain as vestigial components of a cryptic Ton system. The widespread distribution of Ton systems cataloged here supports their presence in the last bacterial common ancestor (LBCA), which was likely a diderm (Witwinowski et al. 2022; Hashimi and Tocheva 2024).

## Discussion

The OM is essential in most Gram-negative bacteria, and is present in the majority of bacterial taxa (Witwinowski et al. 2022). It provides mechanical stability and protection against antibiotics. However, the OM is not energized, hence processes that require the input of energy are coupled to the IM through PMF-powered envelope spanning protein complexes. The homologous Tol and Ton systems extend through the periplasm to interact with their targets in the OM, but the route of their evolutionary divergence to fulfill their disparate functions has remained unclear until now.

**Fig. 8.**
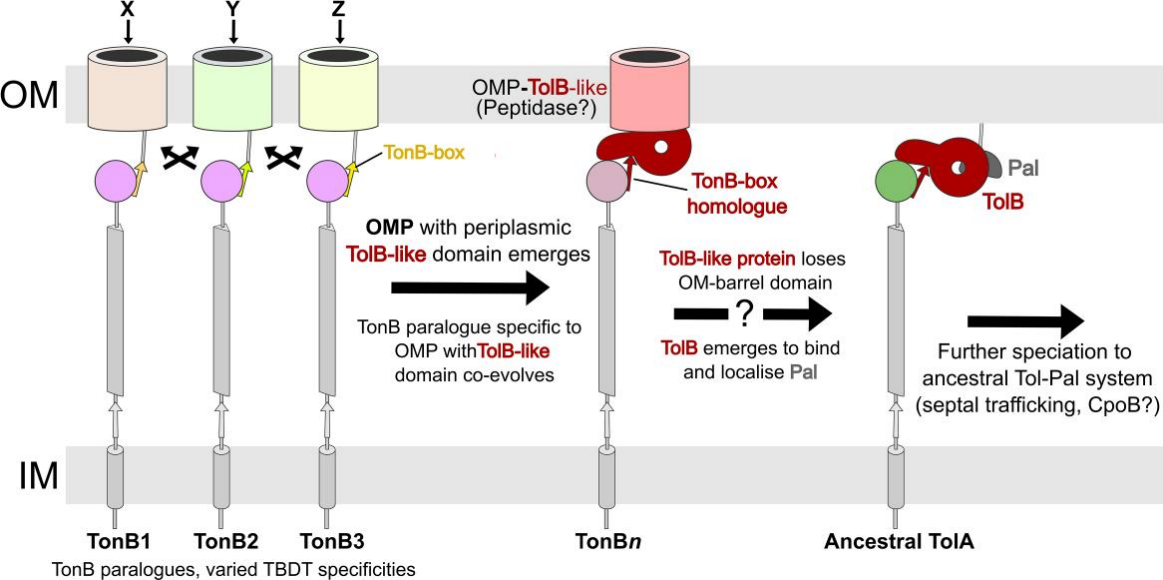
A hypothetical route for TonB-to-TolA speciation. TonB duplication generates paralogues with varied affinities for different TBDT TonB-boxes (*black arrows).* Structural phylogeny revealed a family of FhaC-TolB-like proteins that present plausible extant homologs of an OMP from which TolB emerged, by loss of its β-barrel and subsequent domain reduction (Fig. 5b). Emergence of Pal and the TolB-Pal interaction likely coevolved with the emergence of TolA (*right).* The β-strand addition interaction of TonBIII remains conserved in TolAIII. It is unclear whether TolB or Pal was first-recruited to the division site, or how the Tol-Pal system achieves septal trafficking.

The data presented here suggest the proposed origin of TolA from a TonB paralogue. Firstly, the Ton system is found in most bacterial taxa, suggesting that the Ton system precedes the origin of Tol-Pal. Previous phylogenetic analyses also concluded that ExbBD was likely present in the LBCA, potentially predating the emergence of TBDTs (Marmon 2013). In contrast, Tol-Pal systems can only be found, by definition, in taxa emerging after the origin of Pal (Witwinowski et al. 2022). Secondly, many species exhibit Ton system paralogy, where duplication and speciation of TonB affords TonB paralogues with increased TBDT specificity, such as HasB in *Serratia marcescens* (Amorim et al. 2013), affording sufficient redundancy for speciation to occur. Third, the structural diversity of TolA sequences ranges from a mixture of predominantly α-helical to predominantly proline-rich, the latter of which seems to reflect the TonB-like common ancestor. This is complemented by the observation that both length and α-helix content of TolAII of non-proteobacteria resembles proteobacterial TonBII more closely than proteobacterial TolAII (Fig. 3). Moreover, the wider Gracilicute TolAII sequences feature proline content halfway between that of proteobacterial TolAII and TonBII, suggestive of a transition from proline dominance in the ancestral proto-TolA, to α-helix dominance in modern proteobacterial taxa. In contrast to TolA, analysis of proteobacterial TonB species revealed a general lack of α-helices in domain II. However, conservation of the motor box between TolA and TonB suggests they utilize similar force transduction mechanisms, inherited from their common ancestor (Loll et al. 2024; Zinke et al. 2024). Together, these data suggest the *tol-pal* system evolved from a paralogue of the Ton system that emerged to stabilize the OM during cell division through the energized accumulation of Pal at the mid-cell, to enhance cellular fitness (Fig. 8).

If the Tol-Pal system evolved from the Ton system, many questions arise. First, how has the Tol-Pal system evolved to localize to the division site, which is not the case for the Ton system (Gerding et al. 2007; Jordan et al. 2013; Hale et al. 2022). The mechanism by which TolQRA is trafficked to the septum remains largely unknown, but presumably involves interaction (possibly indirect) with late-stage divisome components such as FtsN, or other as-yet unknown factors. There is some evidence suggesting other components of Tol-Pal system may be involved in TolQRA localization, such as TolB, CpoB, or Pal (Gerding et al. 2007; Baccelli et al. 2022; Hale et al. 2022; Szczepaniak and Webby 2024).

Sequence-base and fold-based searches using TolB yield many distant homologs such as acyl-peptide hydrolases and OMP-propeller fusion proteins (Altschul et al. 1990; Van Kempen et al. 2024) (supplementary SI S1, Supplementary Material online). The TolB β-propeller also shows weak homology with the PG-remodeling protein LysM, suggesting that putative ancestral OMP-TolB-like proteins may have been implicated in septal PG-remodeling before the emergence of the Tol-Pal system. This may also be true of CpoB, which is far more widely conserved than previously suspected, found adjacent to *tol-pal* loci in 70% of non-proteobacterial species and 75% of proteobacterial species respectively (Figs. 1 and 3;supplementary S1.4, Supplementary Material online). Of the non-proteobacterial examples, 82% of CpoB proteins feature an OM-lipidation signal (28 of 34), including the basal lineage of Candidatus Elusimicrobia, suggesting that the CpoB common ancestor was an OM-lipoprotein that co-emerged with the Tol-Pal system (supplementary fig. S1.4, Supplementary Material online), and that lipidation was subsequently lost within a few clades that include Proteobacteria. It is thus plausible that CpoB emerged from another conserved OM-lipoprotein with a TPR domain, such as BamD.

Proteobacteria also demonstrate an anomalous degree of α-helicity within TolAII. We previously demonstrated that the α-helix structure is associated with greater force transduction efficiency than PPII-helices, as indicated by faster bacteriocin import and recruitment of Pal to the septum in *E. coli* (Williams-Jones et al. 2023). The compact structure of α-helices would require more residues for the equivalent reach to PPII-helices/disordered sequences, and we observed that TolAII is typically longer than TonBII in each species (Figs. 2a and 3b). Moreover, the full length of TolAII of γ-proteobacteria are typically much longer than the width of the periplasm, suggesting TolA has excess reach to allow for Pal recruitment in regions of Pal-deficiency and OM-detachment, preventing blebbing and lipid dysregulation (Williams-Jones et al. 2023; Tan and Chng 2025). It has been demonstrated in *E. coli* that deletion of different portions of TolAII are tolerable regardless of position, until the total length of TolAII becomes insufficient to adequately span the periplasm (Schendel et al. 1997).

The TolA common ancestor is expected to be more proline-rich than α-helix-rich, with proline-rich TolAII sequences more frequent in basal clades (Fig. 3a). Whilst the structural features of the protein are more conserved than their sequence on an evolutionary timescale (Siltberg-Liberles et al. 2011), evolutionary fold switching of secondary structures is more common than previously expected (Kim et al. 2021; Chakravarty et al. 2023). What drove the evolutionary transition from PPII-helices to α-helices in domain II is unclear. Perhaps divergent secondary structures better segregate TolA function from TonB. It has been shown, for example, that in *E. coli* both TolQR and TolAII are independently trafficked to the septum by as-yet uncharacterized factors, whereas TonB demonstrates no such trafficking (Gerding et al. 2007; Jordan et al. 2013; Hale et al. 2022).

Alternatively, commensal/pathogenic Proteobacteria with rapid growth rates tend to favor α-helices over PPII-helices, suggesting that shorter division times require faster OM-stabilization. These species tend to be mesothermophilic compared to free-living ones. Perhaps increased TolAII length and α-helicity better tolerates a widened periplasm if OM-fluidity increases as a function of temperature or changes in osmolarity (Los and Murata 2004; Nenninger et al. 2014; de Mendoza and Pilon 2019). The evolutionary advantages of enhanced OM-stabilization to permit increased division speed and altered lipid homeostasis may have driven the emergence of the Tol-Pal system, particularly through structural modifications of CpoB and Pal that increase their mobility. Pal's OmpA-like ancestor was probably immobile, like other OM-β-barrels (Verhoeven et al. 2013), preventing it from accumulating at the septum until it evolved into a lipoprotein. Similarly, loss of lipid tethering from CpoB in Proteobacteria specifically would increase CpoB diffusion rate and thus cause faster septal recruitment, as OM lipoprotein mobility is typically tenfold slower than for periplasmic proteins (Szczepaniak et al. 2020a; Connolley et al. 2022) (supplementary fig. S1.4, Supplementary Material online).

Overall, these findings support the hypothesis that the LBCA was a diderm that featured a Ton system, that later served as an “evolutionary seed” for the Tol-Pal system and other motor-driven force transducers, such as the vWA proteins identified here. The *tol-pal* cluster is far more widely conserved in bacteria than previously suspected, bolstering its significance as a potential target for the development of new strategies in the fight against antimicrobial resistance. Further work is needed to understand the precise mechanism by which proton flux generates rotation and subsequent tensile force at the OM, and the full biological diversity of processes powered by similar undiscovered systems.

## Methods

### PubMLST Multispecies Genome Database *tol-pal* Search Algorithm

To detect *tol-pal* loci within the PubMLST Multispecies genome database, a HMMER 3 hmmscan was performed against all six reading frames using conserved domains (Pfam) (Johnson et al. 2010; Jolley et al. 2018). These included genes encoding the MotA–ExbB–TolQ proton channels (PF01618), the PGB subunit of the motor MotB-ExbB-TolR (PF02472), the TolA–TonB force transducer (PF06519/PF03544/PF13103), the β-propeller and Rossmann-fold of TolB (PF07676/PF04052 respectively), the PG-binding lipoprotein Pal (PF00691), the TPR repeat of CpoB (PF16331/PF13432/PF13174), and the acyl-thioesterase YbgC (PF03061). The output is available in spreadsheet SS1.

Genomes identified with the minimal complement of genes (*tolQRAB* and *pal*) were inspected to confirm the presence of the expected gene products. In cases of poor annotation and/or low sequence homology, protein structures were screened using AF3 to check that they conform to typical three-domain structural predictions of TolA/TonB, containing a TMH, a periplasmic spacer, and globular CTD. Upon confirmation of a minimal *tol-pal* complement, the TolA protein sequence was analyzed using PsiPred 3.0. Genomes of taxa which were not flagged by the search algorithm were searched using genome BLAST to detect the presence of TolB homologs, which were then manually checked for the remaining *tol-pal* complement within genomic vicinity. These loci were also screened by AF3 structure prediction. Manual curation of *tol-pal* loci is outlined in supplementary SI S1, Supplementary Material online.

### Manual *tol-pal* Locus Search

To detect and validate *tol-pal* loci in genomes either poorly represented in the PubMLST Multispecies genome database or presenting as split systems, a manual search was also implemented. Briefly, this search was performed by searching for ““*species*” TolB” on UniProtKB, then investigating the embedded AF-predicted structure (if available) for both TolB domains (Research UCJNa 2019; Abramson et al. 2024). If both the N-terminal domain and the β-propeller were present, the corresponding genome was then accessed via GenBank (Benson et al. 2012) and inspected for the nearby presence of *motA/exbB/tolQ, motB/exbD/tolR, tonB/tolA, pal/ompA,* and *ybgF/cpoB/TPR.* If nearby proteins were labeled “hypothetical protein”, the gene was opened in UniProtKB to determine whether the AF prediction matches any known structures. If no prediction was embedded in UniProtKB, the structure was predicted using AF3 (Abramson et al. 2024). For non-contiguous or poorly annotated genomes, the genome was searched using tBLASTn or BlastP using protein sequences (typically TolB, Pal or TolQ) from the nearest identified homolog.

### TolB Detection

A diverse subset of the curated dataset of TolB was aligned using MAFFT version 7.526 (selected sequences indicated in supplementary SSI S1, Supplementary Material online) with the parameters –localpair and –maxiterate 1,000 (Katoh and Standley 2013). We then used HMMER 3 hmmsearch to query the Witwinowski taxid database for proteins resembling TolB throughout bacterial phylae (Johnson et al. 2010). These were manually inspected via their UniProt-embedded AF models and domain annotations, and these were noted accordingly (supplementary SSI S1, Supplementary Material online) (UniProt Consortium 2022; Abramson et al. 2024). Sequences with UniProt/AF accessions were analyzed using FoldTree, which clusters sequences by structural phylogeny (Moi et al. 2023). The resulting clades were annotated by the noted structural/enzymatic features of the proteins present. The tree was visualized as an unrooted tree using iTol version 7.1, with full branch lengths indicated (Letunic and Bork 2024). The FoldTree output, including the input, accession identifiers, and alignment score/lddt trees, are available in SI 3. MSA-based phylogeny approaches failed due to the extreme diversity of the TolB-related sequences.

### Ton System Detection

To detect whether taxa feature a functional Ton system, the UniProtKB search results for “TonB” and “TonB-dependent-transporters” were each downloaded, and analyzed for the presence of taxa specified by Witwinowski et al. (Witwinowski et al. 2022). A representative TonB from each Taxon was checked for the appropriate length and structure using Αlphafold. A second HMMER 3 hmmscan was queried on the PubMLST Multispecies genome database for PFAM domains corresponding to TonB (PF16031: TonB polyproline region and PF03544: TonB-CTD), and TBDTs (PF00593: TonB-dependent receptor-like beta-barrel and PF07715: TonB-dependent receptor Plug domain) (Johnson et al. 2010; Jolley et al. 2018). The output is available as a spreadsheet (SS1). The flagged species of Proteobacteria were then manually curated for TonBII composition as performed for TolA (supplementary SI S2, Supplementary Material online).

### Secondary Structure/Lipidation Signal Analysis

FASTA sequences for TolA and TonB proteins were input into the PsiPred 3.0 server with default parameters (McGuffin et al. 2000), and the PPIIPred Bioware server with “strict” disabled (O’Brien, et al. 2020). Domain II was then analyzed for α-helix:proline ratio (supplementary SI S1, Supplementary Material online). The end of the predicted TMH was used to mark the beginning of domain II, and the final 100 residues were designated as domain III (Szczepaniak et al. 2020b; Webby et al. 2022). TMHMM 2.0 (hosted by DTU Health Tech) was used to identify the position of the TMH, in cases where PsiPred predictions were unclear (Krogh et al. 2001). The number of domain II residues predicted to be α-helical was compared with the number of proline residues within domain II, each expressed as a percentage of the total number of residues in domain II. To analyze lipidation/secretion signals, the SignalP 6.0 server (hosted by DTU Health Tech) was used.

## Supplementary Material

msaf138_Supplementary_Data

## Data Availability

Supplementary data cataloging TolA and TonB operon/structural compositions are available as additional PDF files (SI1/SI2). Supplementary Spreadsheet (SS1), Witwinowski taxid database queried, TolB MSA profiles & Hmm models, and AF/FoldTree outputs are available in SI 3, deposited on Zenodo (DOI: 10.5281/zenodo.15182822).

## References

[msaf138-B1] Abramson J, Adler J, Dunger J, Evans R, Green T, Pritzel A, Ronneberger O, Willmore L, Ballard AJ, Bambrick JJN. Accurate structure prediction of biomolecular interactions with AlphaFold 3. Nature. 2024:630(8016):493–500. 10.1038/s41586-024-07487-w. 38718835 PMC11168924

[msaf138-B2] Altschul SF, Gish W, Miller W, Myers EW, Lipman DJ. Basic local alignment search tool. J Mol Biol. 1990:215(3):403–410. 10.1016/S0022-2836(05)80360-2. 2231712

[msaf138-B3] Amano A, Takeuchi H, Furuta N. Outer membrane vesicles function as offensive weapons in host–parasite interactions. Microbes Infect. 2010:12:791–798. 10.1016/j.micinf.2010.05.008. 20685339

[msaf138-B4] Amorim G, Prochnicka-Chalufour A, Delepelaire P, Lefevre J, Simenel C, Wandersman C, Delepierre M, Izadi-Pruneyre NJPO. The structure of HasB reveals a new class of TonB protein fold. PLoS One. 2013:8:e58964. 10.1371/journal.pone.0058964. 23527057 PMC3602595

[msaf138-B5] Baccelli P, Rachedi R, Serrano B, Petiti M, Bernard CS, Houot L, Duche D. Timing of TolA and TolQ recruitment at the septum depends on the functionality of the Tol-Pal system. J Mol Biol. 2022:434(7):167519. 10.1016/j.jmb.2022.167519. 35240126

[msaf138-B6] Barrio-Hernandez I, Yeo J, Jänes J, Mirdita M, Gilchrist CLM, Wein T, Varadi M, Velankar S, Beltrao P, Steinegger M. Clustering predicted structures at the scale of the known protein universe. Nature. 2023:622(7983):637–645. 10.1038/s41586-023-06510-w. 37704730 PMC10584675

[msaf138-B7] Beaud Benyahia B, Taib N, Beloin C, Gribaldo S. Terrabacteria: redefining bacterial envelope diversity, biogenesis and evolution. Nat Rev Microbiol. 2025:23(1):41–56. 10.1038/s41579-024-01088-0. 39198708

[msaf138-B8] Benson DA, Cavanaugh M, Clark K, Karsch-Mizrachi I, Lipman DJ, Ostell J, Sayers E. GenBank. Nucleic Acids Res. 2012:41:D36–D42. 10.1093/nar/gks1195. 23193287 PMC3531190

[msaf138-B9] Bonsor DA, Hecht O, Vankemmelbeke M, Sharma A, Krachler AM, Housden NG, Lilly KJ, James R, Moore GR, Kleanthous C. Allosteric beta-propeller signalling in TolB and its manipulation by translocating colicins. EMBO J. 2009:28(18):2846–2857. 10.1038/emboj.2009.224. 19696740 PMC2750012

[msaf138-B10] Braendle C, Miura T, Bickel R, Shingleton AW, Kambhampati S, Stern D. Developmental origin and evolution of bacteriocytes in the aphid–Buchnera symbiosis. PLoS Biol. 2003:1:e21. 10.1371/journal.pbio.0000021. 14551917 PMC212699

[msaf138-B11] Braun V, Herrmann C. Evolutionary relationship of uptake systems for biopolymers in Escherichia coli: cross-complementation between the TonB-ExbB-ExbD and the TolA-TolQ-TolR proteins. Mol Microbiol. 1993:8(2):261–268. 10.1111/j.1365-2958.1993.tb01570.x. 8316079

[msaf138-B12] Brewer S, Tolley M, Trayer IP, Barr GC, Dorman CJ, Hannavy K, Higgins CF, Evans JS, Levine BA, Wormald MR. Structure and function of X-pro dipeptide repeats in the TonB proteins of Salmonella typhimurium and Escherichia coli. J Mol Biol. 1990:216(4):883–895. 10.1016/S0022-2836(99)80008-4. 2266560

[msaf138-B13] Buchan DWA, Moffat L, Lau A, Kandathil SM, Jones DT. Deep learning for the PSIPRED protein analysis workbench. Nucleic Acids Res. 2024:52(W1):W287–W293. 10.1093/nar/gkae328. 38747351 PMC11223827

[msaf138-B14] Cascales E, Lloubes R, Sturgis JN. The TolQ-TolR proteins energize TolA and share homologies with the flagellar motor proteins MotA-MotB. Mol Microbiol. 2001:42(3):795–807. 10.1046/j.1365-2958.2001.02673.x. 11722743

[msaf138-B15] Cecil JD, Sirisaengtaksin N, O’Brien-Simpson NM, Krachler A. Outer membrane vesicle-host cell interactions. Microbiol Spectr. 2019:7:10.1128/microbiolspec.psib-0001-2018. 10.1128/microbiolspec.PSIB-0001-2018. PMC6352913 30681067

[msaf138-B16] Celia H, Botos I, Ghirlando R, Beach BM, Lloubes R, Buchanan SK. Cryo-EM structures of the E. coli Ton and Tol motor complexes. bioRxiv 617233. 10.1101/2024.10.08.617233, 9 October 2024, preprint: not peer reviewed. PMC12215075 40595649

[msaf138-B17] Celia H, Botos I, Ni X, Fox T, De Val N, Lloubes R, Jiang J, Buchanan SK. Cryo-EM structure of the bacterial Ton motor subcomplex ExbB–ExbD provides information on structure and stoichiometry. Commun Biol. 2019:2(1):358. 10.1038/s42003-019-0604-2. 31602407 PMC6778125

[msaf138-B18] Chakravarty D, Sreenivasan S, Swint-Kruse L, Porter LL. Identification of a covert evolutionary pathway between two protein folds. Nat Commun. 2023:14(1):3177. 10.1038/s41467-023-38519-0. 37264049 PMC10235069

[msaf138-B19] Chu BC, Peacock RS, Vogel HJJB. Bioinformatic analysis of the TonB protein family. Biometals. 2007:20:467–483. 10.1007/s10534-006-9049-4. 17225063

[msaf138-B20] Connolley L, Szczepaniak J, Kleanthous C, Murray SM. The quantitative basis for the redistribution of immobile bacterial lipoproteins to division septa. PLoS Comput Biol. 2022:17(12):e1009756. 10.1371/journal.pcbi.1009756. PMC8751993 34965245

[msaf138-B21] Deme JC, Johnson S, Vickery O, Aron A, Monkhouse H, Griffiths T, James RH, Berks BC, Coulton JW, Stansfeld PJ, et al Structures of the stator complex that drives rotation of the bacterial flagellum. Nat Microbiol. 2020:5(12):1553–1564. 10.1038/s41564-020-0788-8. 32929189 PMC7610383

[msaf138-B22] de Mendoza D, Pilon M. Control of membrane lipid homeostasis by lipid-bilayer associated sensors: a mechanism conserved from bacteria to humans. Prog Lipid Res. 2019:76:100996. 10.1016/j.plipres.2019.100996. 31449824

[msaf138-B23] Derouiche R, Lloubes R, Sasso S, Bouteille H, Oughideni R, Lazdunski C, Loret E. Circular dichroism and molecular modeling of the E. coli TolA periplasmic domains. Biospectroscopy. 1999:5(3):189–198. 10.1002/(SICI)1520-6343(1999)5:3<189::AID-BSPY8>3.0.CO;2-O. 10380085

[msaf138-B24] Dodsworth JA, Blainey PC, Murugapiran SK, Swingley WD, Ross CA, Tringe SG, Chain PSG, Scholz MB, Lo C-C, Raymond J, et al Single-cell and metagenomic analyses indicate a fermentative and saccharolytic lifestyle for members of the OP9 lineage. Nat Commun. 2013:4(1):1854. 10.1038/ncomms2884. 23673639 PMC3878185

[msaf138-B25] Domingo Köhler S, Weber A, Howard SP, Welte W, Drescher MJPS. The proline-rich domain of TonB possesses an extended polyproline II-like conformation of sufficient length to span the periplasm of Gram-negative bacteria. Protein Sci. 2010:19:625–630. 10.1002/pro.345. 20095050 PMC2867004

[msaf138-B26] Evans JS, Levine BA, Trayer IP, Dorman CJ, Higgins CF. Sequence-imposed structural constraints in the TonB protein of E. coli. FEBS Lett. 1986:208(2):211–216. 10.1016/0014-5793(86)81020-1. 3023135

[msaf138-B27] Gao T, Meng Q, Gao H. Thioesterase YbgC affects motility by modulating c-di-GMP levels in Shewanella oneidensis. Sci Rep. 2017:7(1):3932. 10.1038/s41598-017-04285-5. 28638070 PMC5479800

[msaf138-B28] Gerding MA, Ogata Y, Pecora ND, Niki H, de Boer PAJ. The trans-envelope Tol-Pal complex is part of the cell division machinery and required for proper outer-membrane invagination during cell constriction in E. coli. Mol Microbiol. 2007:63(4):1008–1025. 10.1111/j.1365-2958.2006.05571.x. 17233825 PMC4428343

[msaf138-B29] Gerke V . Von Willebrand factor folds into a bouquet. EMBO J. 2011:30(19):3880–3881. 10.1038/emboj.2011.321. 21975375 PMC3209786

[msaf138-B30] Germon P, Clavel T, Vianney A, Portalier R, Lazzaroni JC. Mutational analysis of the Escherichia coli K-12 TolA N-terminal region and characterization of its TolQ-interacting domain by genetic suppression. J Bacteriol. 1998:180(24):6433–6439. 10.1128/JB.180.24.6433-6439.1998. 9851983 PMC107741

[msaf138-B31] Germon P, Ray MC, Vianney A, Lazzaroni JC. Energy-dependent conformational change in the TolA protein of *Escherichia coli* involves its N-terminal domain, TolQ, and TolR. J Bacteriol. 2001:183(14):4110–4114. 10.1128/JB.183.14.4110-4114.2001. 11418549 PMC95298

[msaf138-B32] Goemaere EL, Cascales E, Lloubes R. Mutational analyses define helix organization and key residues of a bacterial membrane energy-transducing complex. J Mol Biol. 2007:366(5):1424–1436. 10.1016/j.jmb.2006.12.020. 17222427

[msaf138-B33] Gorasia DG, Seers CA, Heath JE, Glew MD, Soleimaninejad H, Butler CA, McBride MJ, Veith PD, ECJIJoMS R. Type B CTD proteins secreted by the type IX secretion system associate with PorP-like proteins for cell surface anchorage. Int J Mol Sci. 2022:23:5681. 10.3390/ijms23105681. 35628493 PMC9143113

[msaf138-B34] Gray DA, White JBR, Oluwole AO, Rath P, Glenwright AJ, Mazur A, Zahn M, Baslé A, Morland C, Evans SL, et al Insights into SusCD-mediated glycan import by a prominent gut symbiont. Nat Commun. 2021:12(1):44. 10.1038/s41467-020-20285-y. 33398001 PMC7782687

[msaf138-B35] Hale CA, Persons L, de Boer PAJ. Recruitment of the TolA protein to cell constriction sites in Escherichia coli via three separate mechanisms, and a critical role for FtsWI activity in recruitment of both TolA and TolQ. J Bacteriol. 2022:204(1):e00464–e00421. 10.1128/jb.00464-21. 34748387 PMC8765465

[msaf138-B36] Hardy SJ, Christodoulides M, Weller RO, Heckels J. Interactions of Neisseria meningitidis with cells of the human meninges. Mol Microbiol. 2000:36:817–829. 10.1046/j.1365-2958.2000.01923.x. 10844670

[msaf138-B37] Hashimi A, Tocheva EI. Cell envelope diversity and evolution across the bacterial tree of life. Nat Microbiol. 2024:9(10):2475–2487. 10.1038/s41564-024-01812-9. 39294462

[msaf138-B38] Heath JE, Seers CA, Veith PD, Butler CA, Nor Muhammad NA, Chen Y-Y, Slakeski N, Peng B, Zhang L, Dashper S. PG1058 is a novel multidomain protein component of the bacterial type IX secretion system. PLoS One. 2016:11:e0164313. 10.1371/journal.pone.0164313. 27711252 PMC5053529

[msaf138-B39] Hennell James R, Deme JC, Kjӕr A, Alcock F, Silale A, Lauber F, Johnson S, Berks BC, Lea S. Structure and mechanism of the proton-driven motor that powers type 9 secretion and gliding motility. Nat Microbiol. 2021:6:221–233. 10.1038/s41564-020-00823-6. 33432152 PMC7116788

[msaf138-B40] Hirakawa H, Suzue K, Kurabayashi K, Tomita H. The Tol-Pal system of uropathogenic Escherichia coli is responsible for optimal internalization into and aggregation within bladder epithelial cells, colonization of the urinary tract of mice, and bacterial motility. Front Microbiol. 2019:10:1827. 10.3389/fmicb.2019.01827. 31456768 PMC6698795

[msaf138-B41] Hirakawa H, Suzue K, Takita A, Awazu C, Kurushima J, Tomita HJSR. Roles of the Tol-Pal system in the type III secretion system and flagella-mediated virulence in enterohemorrhagic Escherichia coli. Sci Rep. 2020:10:15173. 10.1038/s41598-020-72412-w. 32968151 PMC7511404

[msaf138-B42] Hu Z-Y, Wang Y-Z, Im W-T, Wang S-Y, Zhao G-P, Zheng H-J, Quan Z-X. The first complete genome sequence of the class Fimbriimonadia in the phylum Armatimonadetes. PLoS One. 2014:9(6):e100794. 10.1371/journal.pone.0100794. 24967843 PMC4072686

[msaf138-B43] Islam ST, Jolivet NY, Cuzin C, Belgrave AM, My L, Fleuchot B, Faure LM, Mahanta U, Kezzo AA, Saïdi F, et al Unmasking of the von Willebrand A-domain surface adhesin CglB at bacterial focal adhesions mediates myxobacterial gliding motility. Sci Adv. 2023:9(8):eabq0619. 10.1126/sciadv.abq0619. 36812310 PMC9946355

[msaf138-B44] Johnson LS, Eddy SR, Portugaly E. Hidden Markov model speed heuristic and iterative HMM search procedure. BMC Bioinformatics. 2010:11(1):431. 10.1186/1471-2105-11-431. 20718988 PMC2931519

[msaf138-B45] Jolley KA, Bray JE, MCJWor M. Open-access bacterial population genomics: BIGSdb software, the PubMLST.org website and their applications. Wellcome Open Res. 2018:3:124. 10.12688/wellcomeopenres.14826.1. PMC6192448 30345391

[msaf138-B46] Jordan LD, Zhou Y, Smallwood CR, Lill Y, Ritchie K, Yip WT, Newton SM, Klebba PE. Energy-dependent motion of TonB in the Gram-negative bacterial inner membrane. Proc Natl Acad Sci U S A. 2013:110(28):11553–11558. 10.1073/pnas.1304243110. 23798405 PMC3710835

[msaf138-B47] Jumper J, Evans R, Pritzel A, Green T, Figurnov M, Ronneberger O, Tunyasuvunakool K, Bates R, Žídek A, Potapenko A, et al Highly accurate protein structure prediction with AlphaFold. Nature. 2021:596(7873):583–589. 10.1038/s41586-021-03819-2. 34265844 PMC8371605

[msaf138-B48] Karlsson M, Hannavy K, Higgins CF. A sequence-specific function for the N-terminal signal-like sequence of the TonB protein. Mol Microbiol. 1993:8(2):379–388. 10.1111/j.1365-2958.1993.tb01581.x. 8316087

[msaf138-B49] Katoh K, Standley DM. MAFFT multiple sequence alignment software version 7: improvements in performance and usability. Mol Biol Evol. 2013:30(4):772–780. 10.1093/molbev/mst010. 23329690 PMC3603318

[msaf138-B50] Kim AK, Looger LL, Porter LL. A high-throughput predictive method for sequence-similar fold switchers. Biopolymers. 2021:112(10):e23416. 10.1002/bip.23416. 33462801 PMC8404102

[msaf138-B51] Kirchweger P, Weiler S, Egerer-Sieber C, Blasl AT, Hoffmann S, Schmidt C, Sander N, Merker D, Gerlach RG, Hensel MJMM. Structural and functional characterization of SiiA, an auxiliary protein from the SPI4-encoded type 1 secretion system from Salmonella enterica. Mol Microbiol. 2019:112:1403–1422. 10.1111/mmi.14368. 31419359

[msaf138-B52] Koebnik R . The molecular interaction between components of the TonB-ExbBD-dependent and of the TolQRA-dependent bacterial uptake systems. Mol Microbiol. 1993:9(1):219. 10.1111/j.1365-2958.1993.tb01683.x. 8412667

[msaf138-B53] Krogh A, Larsson B, von Heijne G, Sonnhammer ELL. Predicting transmembrane protein topology with a hidden Markov model: application to complete genomes. J Mol Biol. 2001:305(3):567–580. 10.1006/jmbi.2000.4315. 11152613

[msaf138-B54] Letunic I, Bork P. Interactive Tree of Life (iTOL) v6: recent updates to the phylogenetic tree display and annotation tool. Nucleic Acids Res. 2024:52(W1):W78–W82. 10.1093/nar/gkae268. 38613393 PMC11223838

[msaf138-B55] Levengood SK, Beyer WF Jr, Webster RE. TolA: a membrane protein involved in colicin uptake contains an extended helical region. Proc Natl Acad Sci U S A. 1991:88(14):5939–5943. 10.1073/pnas.88.14.5939. 2068069 PMC51997

[msaf138-B56] Llamas M, Ramos JL, Rodríguez-Herva J. Mutations in each of the tol genes of Pseudomonas putida reveal that they are critical for maintenance of outer membrane stability. J Bacteriol. 2000:182:4764–4772. 10.1128/JB.182.17.4764-4772.2000. 10940016 PMC111352

[msaf138-B57] Loll PJ, Grasty KC, Shultis DD, Guzman NJ, Wiener M. Discovery and structural characterization of the D-box, a conserved TonB motif that couples an inner-membrane motor to outer-membrane transport. J Biol Chem. 2024:300:105723. 10.1016/j.jbc.2024.105723. 38311172 PMC10907165

[msaf138-B58] Los DA, Murata N. Membrane fluidity and its roles in the perception of environmental signals. Biochim Biophys Acta. 2004:1666(1-2):142–157. 10.1016/j.bbamem.2004.08.002. 15519313

[msaf138-B59] Marmon L . Elucidating the origin of the ExbBD components of the TonB system through Bayesian inference and maximum-likelihood phylogenies. Mol Phylogenet Evol. 2013:69(3):674–686. 10.1016/j.ympev.2013.07.010. 23891663

[msaf138-B60] McGuffin LJ, Bryson K, Jones DT. The PSIPRED protein structure prediction server. Bioinformatics (Oxford, England). 2000:16(4):404–405. 10.1093/bioinformatics/16.4.404. 10869041

[msaf138-B61] Mirus O, Strauss S, Nicolaisen K, von Haeseler A, Schleiff E. TonB-dependent transporters and their occurrence in cyanobacteria. BMC Biol. 2009:7(1):68. 10.1186/1741-7007-7-68. 19821963 PMC2771747

[msaf138-B62] Moi D, Bernard C, Steinegger M, Nevers Y, Langleib M, Dessimoz C. Structural phylogenetics unravels the evolutionary diversification of communication systems in gram-positive bacteria and their viruses. bioRxiv 558401. 10.1101/2023.09.19.558401, 23 September 2023, preprint: not peer revie wed. PMC12700828 41073779

[msaf138-B63] Nenninger A, Mastroianni G, Robson A, Lenn T, Xue Q, Leake MC, Mullineaux CW. Independent mobility of proteins and lipids in the plasma membrane of Escherichia coli. Mol Microbiol. 2014:92(5):1142–1153. 10.1111/mmi.12619. 24735432 PMC4276291

[msaf138-B64] O’Brien KT, Mooney C, Lopez C, Pollastri G, Shields DC. Prediction of polyproline II secondary structure propensity in proteins. R Soc Open Sci. 2020:7(1):191239. 10.1098/rsos.191239. 32218953 PMC7029904

[msaf138-B65] Onoue Y, Iwaki M, Shinobu A, Nishihara Y, Iwatsuki H, Terashima H, Kitao A, Kandori H, Homma MJ Sr. Essential ion binding residues for Na+ flow in stator complex of the Vibrio flagellar motor. Sci Rep. 2019:9:11216. 10.1038/s41598-019-46038-6. 31375690 PMC6677748

[msaf138-B66] Perotti B, Clarke HK, Turner BD, Braig HR, Perotti B, Clarke HK, Turner BD, Braig HRJTFJ. Rickettsia as obligate and mycetomic. FASEB J. 2006:20:2372–2374. 10.1096/fj.06-5870fje. 17012243

[msaf138-B67] Pollet RM, Foley MH, Kumar SS, Elmore A, Jabara NT, Venkatesh S, Vasconcelos Pereira G, Martens EC, Koropatkin N. Multiple TonB homologs are important for carbohydrate utilization by Bacteroides thetaiotaomicron. J Bacteriol. 2023:205:e00218-00223. 10.1128/jb.00218-23. 37874167 PMC10662123

[msaf138-B68] Pollet RM, Martin LM, Koropatkin NM. TonB-dependent transporters in the Bacteroidetes: unique domain structures and potential functions. Mol Microbiol. 2021:115(3):490–501. 10.1111/mmi.14683. 33448497

[msaf138-B69] Poppleton DI, Duchateau M, Hourdel V, Matondo M, Flechsler J, Klingl A, Beloin C, Gribaldo S. Outer membrane proteome of Veillonella parvula: a diderm firmicute of the human microbiome. Front Microbiol. 2017:8:1215. 10.3389/fmicb.2017.01215. 28713344 PMC5491611

[msaf138-B70] Ren C-P, Beatson SA, Parkhill J, Pallen M. The flag-2 locus, an ancestral gene cluster, is potentially associated with a novel flagellar system from Escherichia coli. J Bacteriol. 2005:187:1430–1440. 10.1128/JB.187.4.1430-1440.2005. 15687208 PMC545627

[msaf138-B71] Research UCJNa . UniProt: a worldwide hub of protein knowledge. Nucleic Acids Res. 2019:47:D506–D515. 10.1093/nar/gky1049. 30395287 PMC6323992

[msaf138-B72] Rymaszewska A, Grenda SJVM. Bacteria of the genus Anaplasma–characteristics of Anaplasma and their vectors: a review. Vet Med. 2008:53:573–584. 10.17221/1861-VETMED.

[msaf138-B73] Saha CK, Sanches Pires R, Brolin H, Delannoy M, Atkinson GC. Flags and webFlaGs: discovering novel biology through the analysis of gene neighbourhood conservation. Bioinformatics (Oxford, England). 2020:37:1312–1314. 10.1093/bioinformatics/btaa788. PMC8189683 32956448

[msaf138-B74] Santiveri M, Roa-Eguiara A, Kühne C, Wadhwa N, Hu H, Berg HC, Erhardt M, Taylor NMI. Structure and function of stator units of the bacterial flagellar motor. Cell. 2020:183(1):244–257.e216. 10.1016/j.cell.2020.08.016. 32931735

[msaf138-B75] Schendel SL, Click EM, Webster RE, Cramer WA. The TolA protein interacts with colicin E1 differently than with other group A colicins. J Bacteriol. 1997:179(11):3683–3690. 10.1128/jb.179.11.3683-3690.1997. 9171417 PMC179165

[msaf138-B76] Sharma V, Vashishtha A, Jos ALM, Khosla A, Basu N, Yadav R, Bhatt A, Gulani A, Singh P, Lakhera S. Phylogenomics of the phylum Proteobacteria: resolving the complex relationships. 2022:79:224. 10.1007/s00284-022-02910-9. 35704242

[msaf138-B77] Siltberg-Liberles J, Grahnen JA, Liberles DA. The evolution of protein structures and structural ensembles under functional constraint. Genes (Basel). 2011:2(4):748–762. 10.3390/genes2040748. 24710290 PMC3927589

[msaf138-B78] Sturgis JN . Organisation and evolution of the tol-pal gene cluster. J Mol Microbiol Biotechnol. 2001:3:113–122. https://www.caister.com/backlist/jmmb/v/v3/v3n1/11.pdf. 11200223

[msaf138-B79] Szczepaniak J, Holmes P, Rajasekar K, Kaminska R, Samsudin F, Inns PG, Rassam P, Khalid S, Murray SM, Redfield C, et al The lipoprotein Pal stabilises the bacterial outer membrane during constriction by a mobilisation-and-capture mechanism. Nat Commun. 2020a:11(1):1305. 10.1038/s41467-020-15083-5. 32161270 PMC7066135

[msaf138-B80] Szczepaniak J, Press C, Kleanthous C. The multifarious roles of Tol-Pal in Gram-negative bacteria. FEMS Microbiol Rev. 2020b:44(4):490–506. 10.1093/femsre/fuaa018. 32472934 PMC7391070

[msaf138-B81] Szczepaniak J, Webby MN. The Tol Pal system integrates maintenance of the three layered cell envelope. NPJ Antimicrob Resist. 2024:2(1):46. 10.1038/s44259-024-00065-0. 39843782 PMC11721397

[msaf138-B82] Tan WB, Chng S-S. Primary role of the Tol-Pal complex in bacterial outer membrane lipid homeostasis. Nat Commun. 2025:16(1):2293. 10.1038/s41467-025-57630-y. 40055349 PMC11889096

[msaf138-B83] Taylor N, Hu H, Hughes T, Popp P, Roa-Eguiara A, Martin F, Rutbeek N, Hendriks I, Payne L, Yan Y, et al Structure and mechanism of the Zorya anti-phage defense system. Nature. 2025:639:1093–1101. 10.21203/rs.3.rs-3768720/v1. 39662505 PMC11946911

[msaf138-B84] Teufel F, Almagro Armenteros JJ, Johansen AR, Gíslason MH, Pihl SI, Tsirigos KD, Winther O, Brunak S, von Heijne G, Nielsen H. Signalp 6.0 predicts all five types of signal peptides using protein language models. Nat Biotechnol. 2022:40(7):1023–1025. 10.1038/s41587-021-01156-3. 34980915 PMC9287161

[msaf138-B85] UniProt Consortium . UniProt: the universal protein knowledgebase in 2023. Nucleic Acids Res. 2022:51:D523–D531. 10.1093/nar/gkac1052. PMC9825514 36408920

[msaf138-B86] Van Kempen M, Kim SS, Tumescheit C, Mirdita M, Lee J, Gilchrist CL, Söding J, Steinegger M. Fast and accurate protein structure search with Foldseek. Nat Biotechnol. 2024:42:243–246. 10.1038/s41587-023-01773-0. 37156916 PMC10869269

[msaf138-B87] Verhoeven GS, Dogterom M, den Blaauwen T. Absence of long-range diffusion of OmpA in E. coli is not caused by its peptidoglycan binding domain. BMC Microbiol. 2013:13(1):66. 10.1186/1471-2180-13-66. 23522061 PMC3637615

[msaf138-B88] Vianney A, Lewin TM, Beyer WF Jr, Lazzaroni JC, Portalier R, Webster RE. Membrane topology and mutational analysis of the TolQ protein of Escherichia coli required for the uptake of macromolecules and cell envelope integrity. J Bacteriol. 1994:176(3):822–829. 10.1128/jb.176.3.822-829.1994. 8300535 PMC205120

[msaf138-B89] Webby MN, Williams-Jones DP, Press C, Kleanthous C. Force-generation by the trans-envelope Tol-Pal system. Front Microbiol. 2022:13:852176. 10.3389/fmicb.2022.852176. 35308353 PMC8928145

[msaf138-B90] Williams-Jones DP, Webby MN, Press CE, Gradon JM, Armstrong SR, Szczepaniak J, Kleanthous C. Tunable force transduction through the Escherichia coli cell envelope. Proc Natl Acad Sci U S A. 2023:120(47):e2306707120. 10.1073/pnas.2306707120. 37972066 PMC10666116

[msaf138-B91] Witty M, Sanz C, Shah A, Grossmann JG, Mizuguchi K, Perham RN, Luisi B. Structure of the periplasmic domain of Pseudomonas aeruginosa TolA: evidence for an evolutionary relationship with the TonB transporter protein. EMBO J. 2002:21(16):4207–4218. 10.1093/emboj/cdf417. 12169623 PMC126161

[msaf138-B92] Witwinowski J, Sartori-Rupp A, Taib N, Pende N, Tham TN, Poppleton D, Ghigo J-M, Beloin C, Gribaldo SJNM. An ancient divide in outer membrane tethering systems in bacteria suggests a mechanism for the diderm-to-monoderm transition. Nat Microbiol. 2022:7:411–422. 10.1038/s41564-022-01066-3. 35246664

[msaf138-B93] Yeow J, Chia CG, Lim NZ-L, Chng S-S. Structural insights into the force-transducing mechanism of a motor-stator complex important for bacterial outer membrane lipid homeostasis. bioRxiv 625625. 10.1101/2024.11.27.625625, 29 November 2024, preprint: not peer reviewed. 40589080

[msaf138-B94] Zhang XY-Z, Goemaere EL, Seddiki N, Celia H, Gavioli M, Cascales E, Lloubes R. Mapping the interactions between Escherichia coli TolQ transmembrane segments. J Biol Chem. 2011:286(13):11756–11764. 10.1074/jbc.M110.192773. 21285349 PMC3064227

[msaf138-B95] Zinke M, Lejeune M, Mechaly A, Bardiaux B, Boneca IG, Delepelaire P, Izadi-Pruneyre NJNC. Ton motor conformational switch and peptidoglycan role in bacterial nutrient uptake. Nat Commun. 2024:15:331. 10.1038/s41467-023-44606-z. 38184686 PMC10771500

