# Supplementary material for: Origin and Evolution of Bacterial Periplasmic Force Transducers
Source: Mol Biol Evol. 2025 Jun 4;42(6):msaf138. doi: 10.1093/molbev/msaf138 (PMC12204202; doi:10.1093/molbev/msaf138)
Supplement: msaf138_Supplementary_Data [file msaf138_supplementary_data.zip › REVISED- SI 1 Tol-Pal system catalogue.pdf]

Contents

Introduction **4**

Proteobacteria (Sharma tree) **6**

Operon Analysis **11**

γ-Proteobacteria **12**

Enterobacterales 12

Pasteuralleles 17

Vibrionales 19

Aeromondales 20

Alteromondales 21

Iteromondales 27

Pseudomonodales 28

Oceanspirallales 31

Thiotrichales 35

Legionellales 37

Methylococcales 39

Chromatiales 40

Cardiobacteriales 43

Xanthomonadales 44

β-Proteobacteria **47**

Neisseriales 47

Burkholderiales 49

Rhodocyclales 54

Nitrosomonadales 55

Acidithiobacillales **59**

α-Proteobacteria **61**

Magnetococcales 61

Rickettsiales 62

Rhodospirillales 63

Sphingomonadales 65

Rhodobacterales 68

Caulobacterales 70

Parvularculales 71

Rhizobiales 73

δ-Proteobacteria **80**

Syntrophobacterales 80

Desulfarculales 82
Desulfobacterales 83
Desulfovibrionales 85
Bacteriovoracles 88
Desulfurellales 90
Oligoflexia 91
Bacteriovoracles (cont.) 91
Bdellovibrionales 92
ε-Proteobacteria 93
Campylobacterales 93
Wider phylae (Witwinowski tree) 97
Candidatus Elusimicrobia 98
Candidatus mciNerneyibacteriota 100
Aquificae 101
Thermodesulfobacteria 102
Candidadatus Calescamentes 103
Candidatus Dependentia 104
Epsilonproteobacteria 105
Candidatus Methylomirabilis 106
Candidatus Rokubacteria 107
Candidatus Fischerbacteria 108
Candidatus Aminicenantes 109
Acidobacteria 110
Deferribacteres 111
Chrysiogenetes 112
Nitrospinae 113
Candidatus Schekmanbacteria 115
Nitrospirae 116
Candidatus lambdaproteobacteria 117
Candidatus Dadabacteria 118
Oligoflexia 119
Candidatus Poribacteria 120
Candidatus Sumerlaeota 121
Planctomycetes 122
Candidatus Ratteibacteria 123
Candidatus Hydrogendentes 124
Candidatus Abyssubacteria 125

Candidatus Omnitrophica 126
Chlamydiae 127
Candidatus Aureabacteria 128
Verrucomicrobiota 129
Lentisphaerae 130
Kiritimatiellota 132
Candidadatus Goldbacteria 135
Candidadatus Firestonebacteria 136
Candidadatus Hydrothermota 137
Candidatus Coatesbacteria 138
Candidatus Stahlbacteria 139
Candidatus Cloacimonetes 140
Candidatus Fermentibacter 141
Candidatus Aegiribacteria 142
Candidatus Eisenbacteria 143
Candidatus Edwardsbacteria 144
Candidatus Glassbacteria 145
Gemmatimonadetes 146
Candidate Division Zixibacteria 147
Candidatus Handelsmanbacteria 148
Candidatus Latescibacteria 149
Fibrobacteria 150
Candidatus Marinimicrobia 151
Candidatus Delongbacteria 152
Candidate division KSB1 153
Calditrichaeota 155
Candidatus Kryptonia 156
Ignavibacteria 157
Candidatus Kapaibacteria 157
Bacteroidetes 158
Rhodothermaeota 159
Balneolaeota 160
Chlorobi 161
Terrabacteria 162
Thermotogae 162

Introduction

Please note, full references for cited works are available in the main text. Commentary is provided on each *tol-pal* locus and idiosyncrasies are highlighted. Relative gene lengths in locus cartoons are representational only, and are not always reflective of true gene lengths. Relative orientations of flanking genes may be inaccurate, as the data were transcribed from the genomic Genbank FASTA format only, except for split *tol-pal* systems where it was interesting to explore relative positions/orientations of the different components.

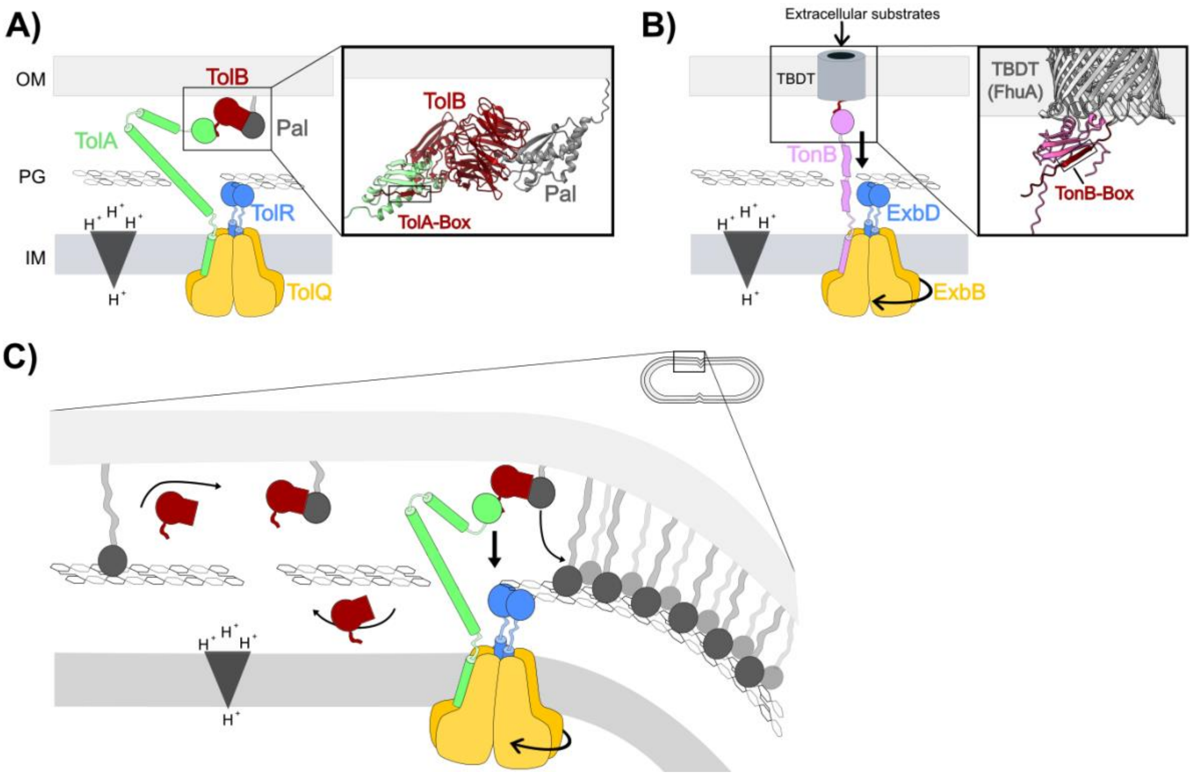

Figure S1.1 | The Tol-Pal system and Ton system use structurally homologous motors to apply force to the OM via β-addition to OM targets. A) TolA dissociates the TolB-Pal complex by applying tensile force, energised by the PMF-driven TolQR motor. TolAIII interacts directly with the exposed N-terminus of TolB from the TolB-Pal complex, by β-addition (*right*). Structural model predicted by AF3 (Abramson *et al.,* 2024), with individual TolB (*red*) and Pal (*grey*) subunits. "TolA-Box" here refers to the TolA-bound β-strand formed by TolB (*residues 23-28*). Rotation is applied to TolA through interaction with TolQR, as means to generate tensile force at the OM. **B)** The Ton system drives uptake of extracellular substrates by applying tensile force. ExbBD-TonB applies force to TBDTs perpendicular to the membrane (*black arrow*), through conformational rearrangements within the monomeric force transducer TonB (*left*). TonB binds its target motif, the TonB-box, by β-addition (AF3 model; *right*). The ExbBD-TonB complex uses the PMF to drive substrate import via rotation of the ExbBD motor, in the same manner as TolQRA. **C)** The Tol-Pal system uses a mobilisation-and-capture mechanism to accumulate Pal at the division site. Pal tethers the inner leaflet of the OM to the PG layer. The soluble periplasmic protein TolB displaces Pal from the PG, effectively mobilising Pal, which can then diffuse around the cell. During cell division, TolQR and the force transducer TolA are independently recruited to the mid-cell, where the TolQRA complex strips TolB from Pal. This results in local deposition of Pal, the accumulation of OM-PG tethers and invagination of the OM. Free TolB is recycled, which diffuses through the periplasm to mobilise additional Pal molecules for mid-cell localisation. Figure adapted from Williams-Jones *et al.,* 2023.

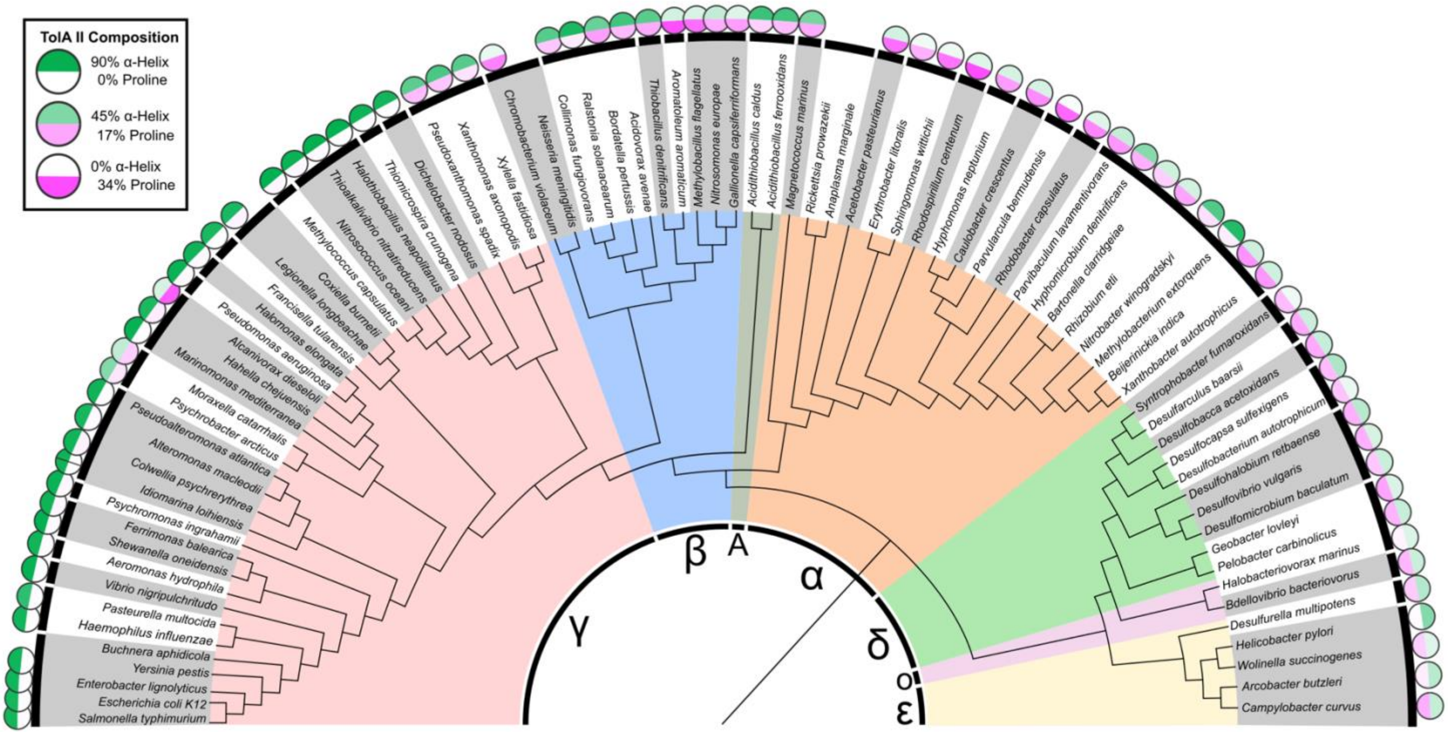

Figure S1.2 | Circularised consensus tree, adapted from Sharma *et al.*, 2022. TolA α-helix: proline ratios are plotted against the representative species. Loci of each *tol-pal* system further verified in this index.

Proteobacteria (Sharma tree)

These loci demonstrate considerably greater conservation of core gene organisation/synteny than expected, the majority featuring a similar organisation to that of *E. coli.* In some cases, "split" *tol-pal* loci were observed, where *tolQRAB* and *pal-cpoB* loci were separated on the genome, while a few appear to have lost their *tol-pal* clusters. Overall, these outliers suggest that while the "core" locus organisation is a suitable guide for *tol-pal* system detection, exceptions exist. The flanking genes of each *tol-pal* cluster were semi-syntenic but varied between classes, suggestive of positional changes on the chromosome over time (Fig. S1.4).

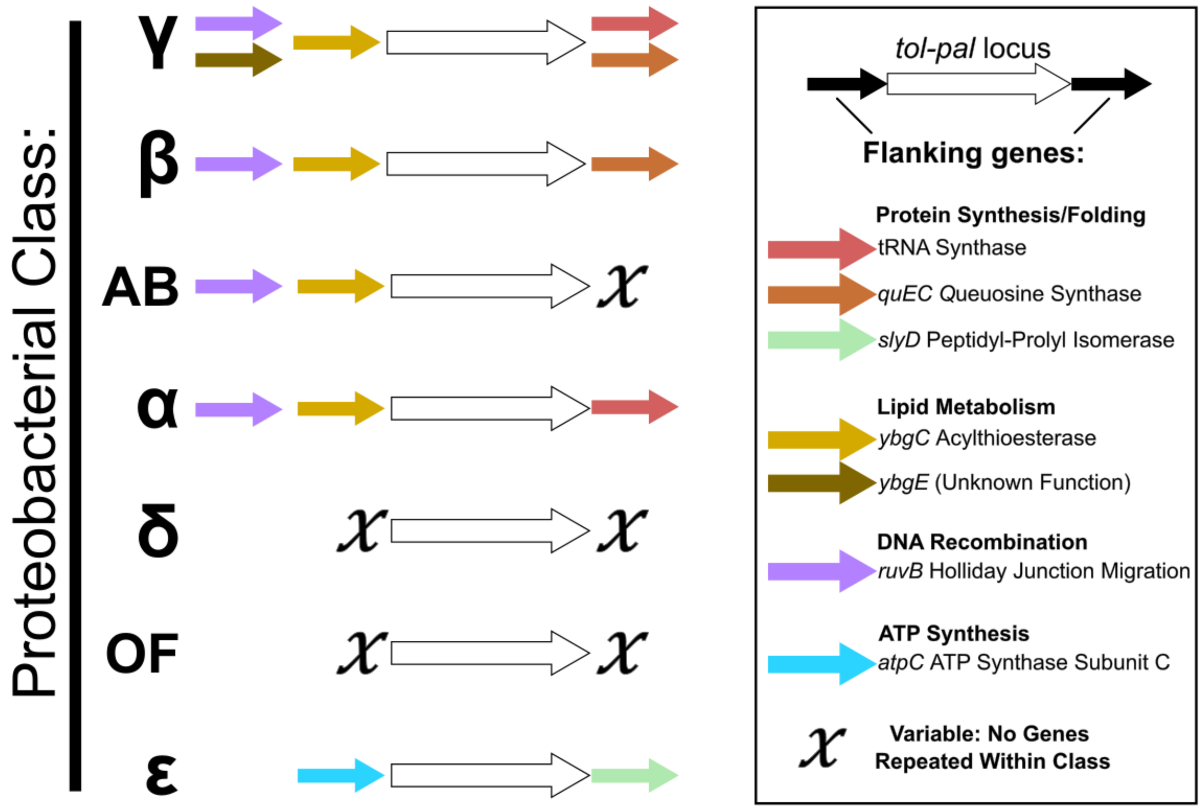

Figure S1.3 | Synteny of proteobacterial *tol-pal* flanking genes. The general functions of syntenic *tol-pal* flanking genes vary between proteobacterial classes including *Acidothiobacillia* (*AB*) and *Oligoflexia* (*OF*). Paired arrows in γ-proteobacteria indicate alternative syntenic genes within the class. "$\mathcal{x}$" denotes the absence of any genes reoccurring more than once within the class. Overall, the genes adjacent to *tol-pal* are generally conserved within proteobacterial classes, but vary between them.

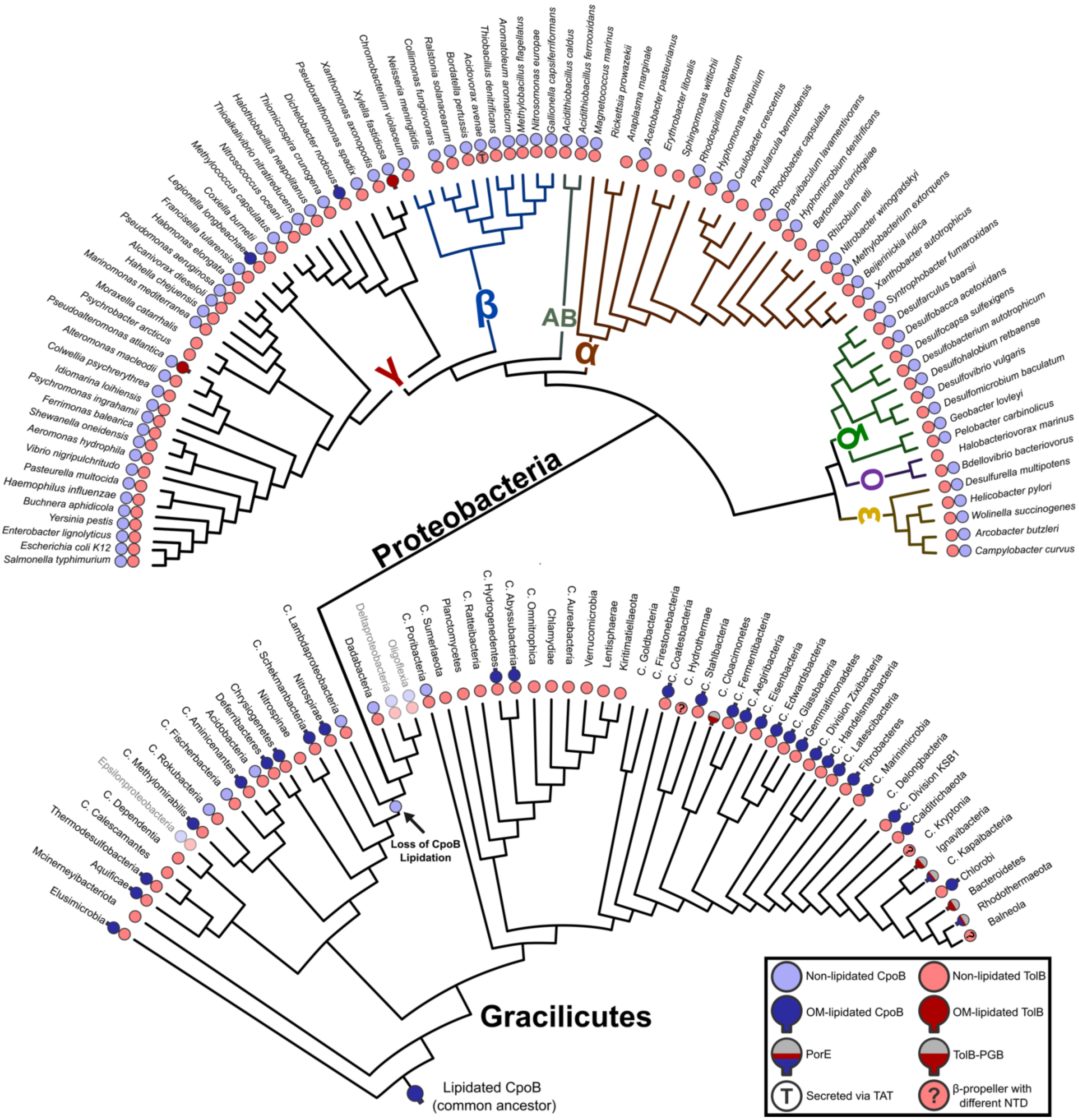

Figure S1.4 | Lipidation states of TolB and CpoB suggest CpoB common ancestor was lipidated, while TolB was not. Of TolB sequences, only *A. macleodii* and *X. fastidiosa* demonstrated propensity for lipidation. In contrast, many TolB sequences from wider Gracilicutes exhibit propensity for lipidation, as predicted by SignalP 6.0 (Teufel *et. al.,* 2022). The conservation of CpoB lipidation from basal clades such as Elusimicrobia to later diverging clades (*right*) suggests that the common ancestor of CpoB was lipidated, and this lipidation was subsequently lost in some clades, including Proteobacteria.

TolA sequences of wider phylae were investigated according to the phylogenetic tree produced by Witwinowski et al., (2022; Fig. S1.5).

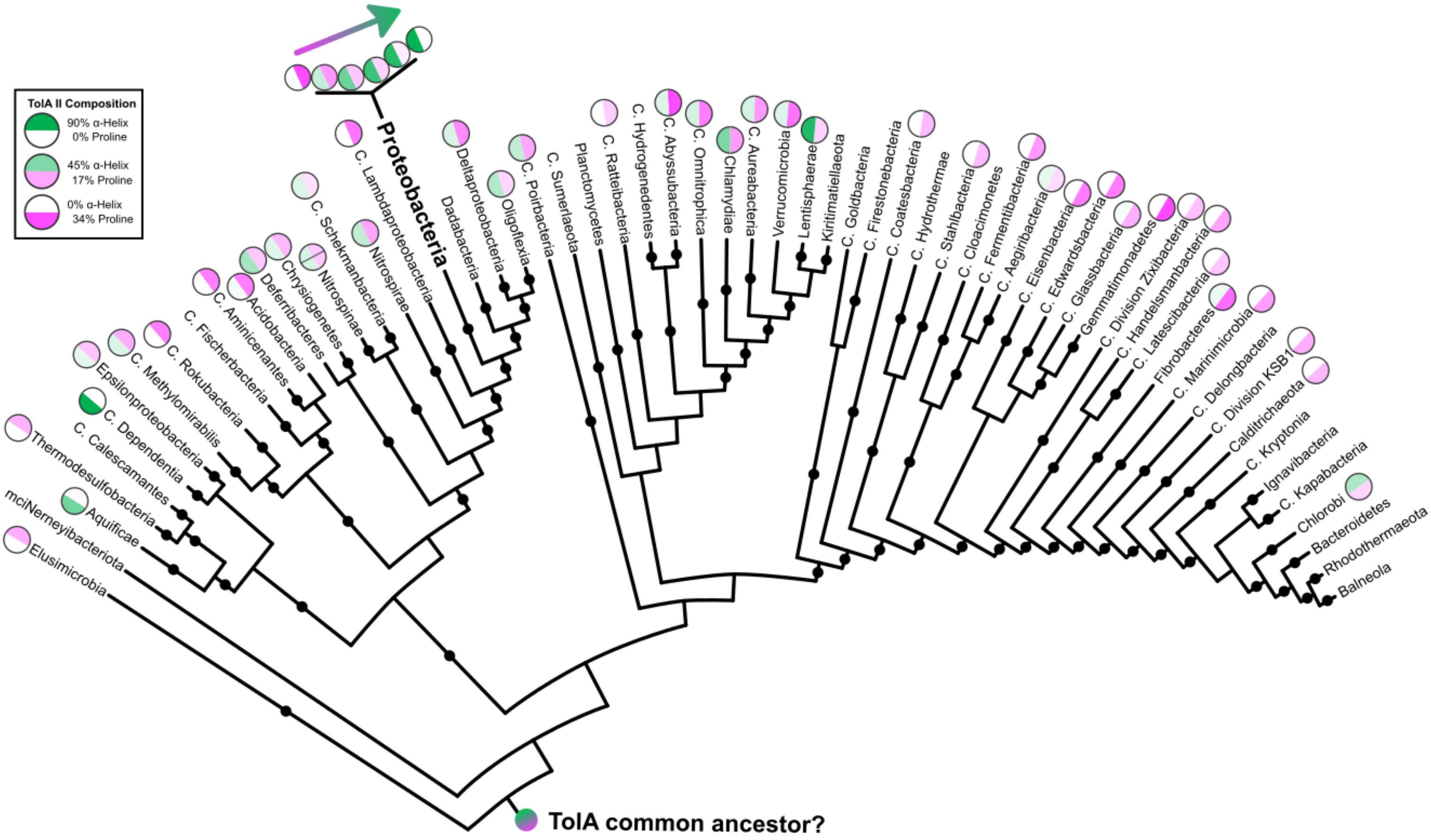

Figure S1.5 | Gracilicute branch of phylogenetic tree containing *pal* (Witwinowski *et al.*, 2022). TolA helix: proline ratios calculated in this index indicate taxa with identifiable *tol-pal* loci. Black dots indicate bootstrap values >90% acquired by Witwinowski *et al.* (2022).

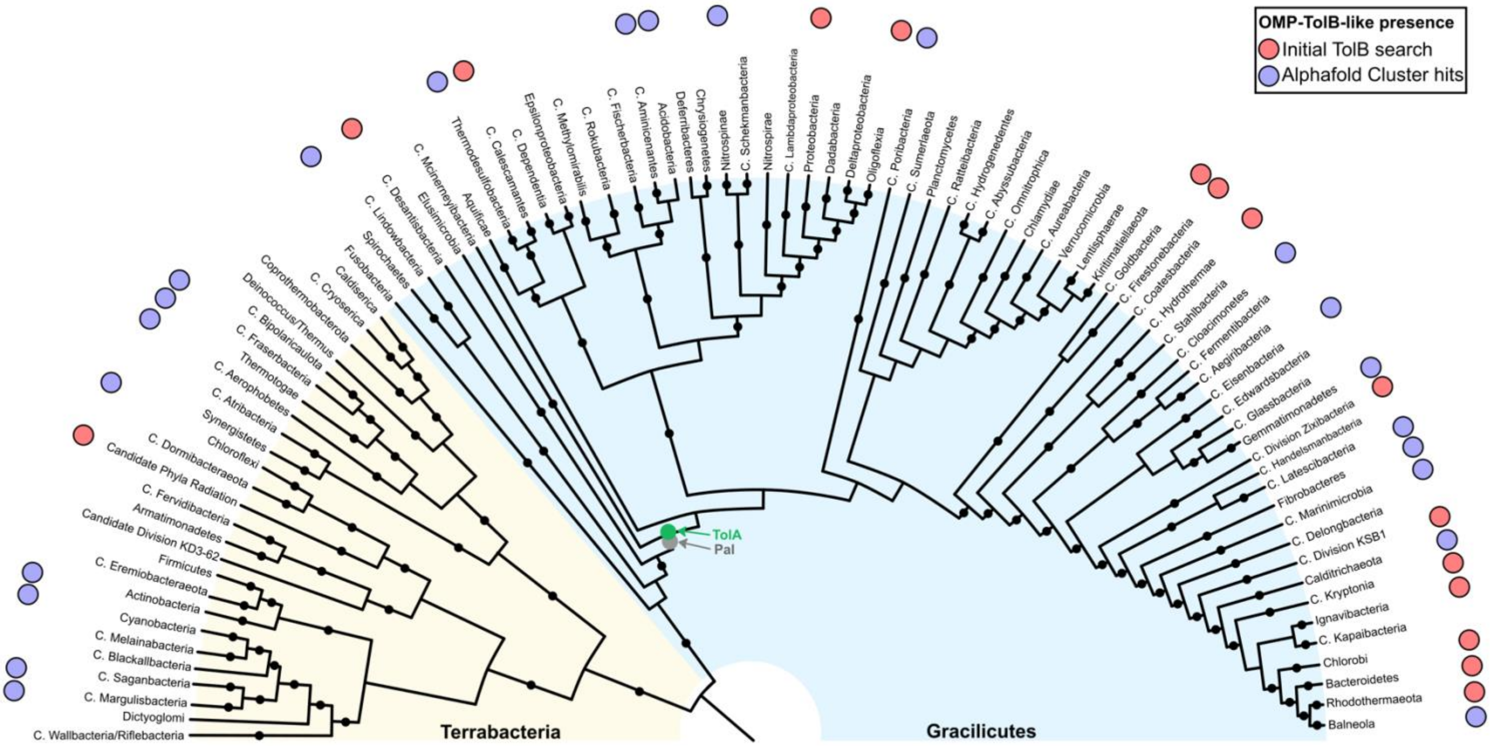

Figure S1.6 | Initial TolB HMMER search and AF clusters suggest OMP-TolB-like proteins are widely distributed throughout bacteria. The HMMER search against the Witwinowski taxid database for TolB revealed a family of OMPs with a TolB-like periplasmic domain (*red*) (Witwinowski *et al*., 2022). The AF accessions for each were used to identify their AF clusters, where proteins are clustered when they exhibit more than 90% structural alignment overlap (Barrio-Hernandez *et al.,* 2023). Assessing the taxonomy of species with proteins within each cluster reveals that structural relatives of the OMP-TolB-like proteins are found throughout both Terrabacteria and Gracilicutes. FASTA sequences, and AF/cluster accessions available in SS1.

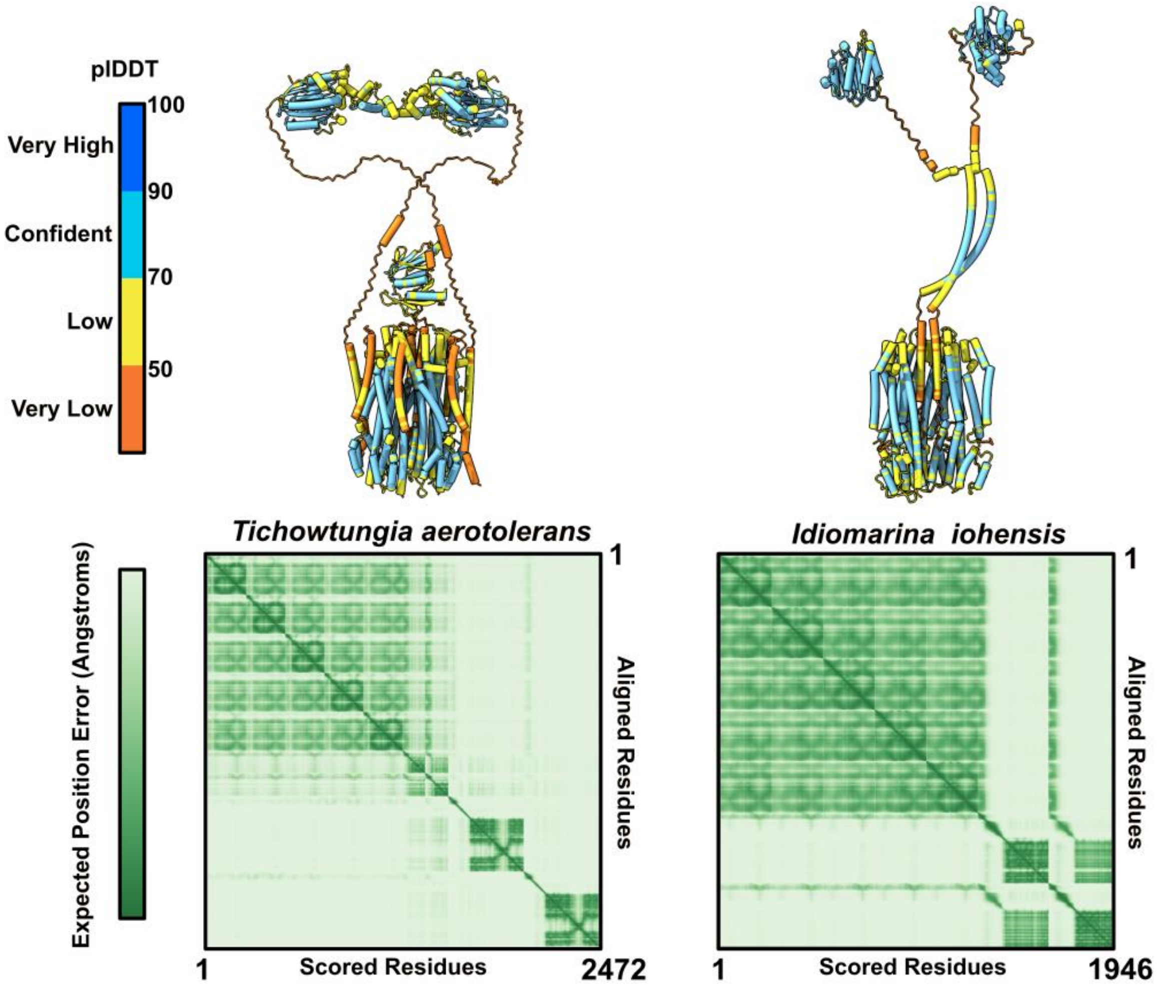

Figure S1.7 | AlphaFold 3 plDDT and PAE scores for vWA protein complex predictions. One pentamer subunit is hidden from the *I. iohensis* complex to show the trans-pore helices of the dimer component. Disordered regions near the C-terminal vWA domain were manually reoriented for clarity, original files are available in supplementary folder SI 3.

Operon Analysis

Each PsiPred prediction uses the following legend:

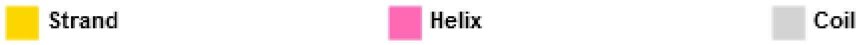

Helix: Proline analysis was performed by first defining domain II (indicated in bold for each protein), specifically after the predicted TMH, up to 100 residues before the C-terminus.

Each PPIIPRED prediction uses the following legend:

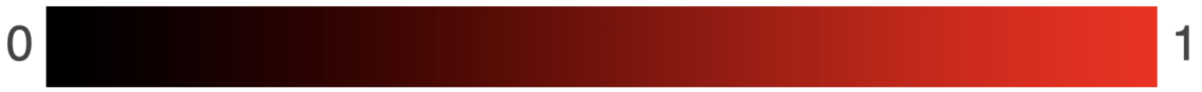

Where 0 indicates no propensity for PPII helix formation, and 1 indicates maximum propensity for PPII helix formation.

The quantified ratio was plotted (supplementary spreadsheet) and conditionally formatted according to the colour scheme indicated on the phylogenetic tree. Every TolA in this list was also visually inspected in the embedded UniProt Alphafold prediction model to check for the presence of an N-terminal helix (domain I), domain II, and globular domain III. In cases where part of TolA or Pal is missing, this is highlighted. In cases of poor annotation, pBLAST searches were performed using Pal or TolB from the closest phylogenetic branch on the tree (Fig. S1.2;1.5).

γ-Proteobacteria

Enterobacterales

Salmonella typhimurium

Locus: 812263-818850

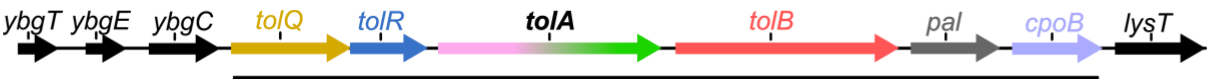

Flanked by *ybgTE* and *lysT* tRNA synthase.

>AAL19691.1 tol protein, membrane spanning protein [Salmonella enterica subsp. enterica serovar Typhimurium str. LT2]

MSKATEQNDKLKRAIIISAVLHIILFAVLIWSSFDEHIEASAGGGGGSAIDAVMVDPGAVVQQYNRQQDQQASARRAEEERKKLQ
QQQAEELQQKQAAEQERLKQLEKERLAAQEQQKQAEEAAKLAQQQQQQAEEAAKAAADAKKKAEAEAAKAAADAKKKAEAEA
VKAAADAKKKAEAEAAKAAADAKKKAEAEAAKAAAEAKKKAEAEAAKAAAEAKKKADAEAAKAAAEAKKKADAAAAKAAAEAKK
KADAAAAKAAADAKKKAAAEKAAAAEGVDDLLGDLSSGKNAPKTGGGAKGNGQPSKDSGTSGANGGATGADISAYAKQIQVAI
QSRLYDASLYQGKQCVLHISLAPDGSLKSITSEGGDPALCQAALMAAKTAKIPKPPSQAVYEKIKDAKLDFKL

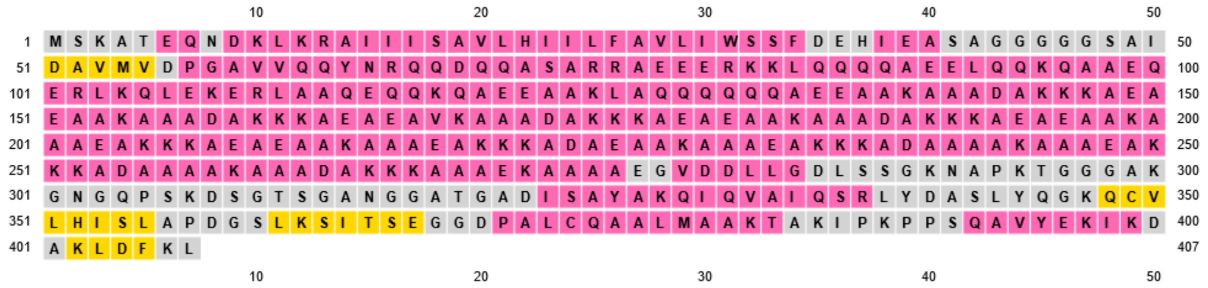

MSKATEQNDKLKRAIIISAVLHIILFAVLIWSSF
**DEHIEASAGGGGGSAIDAVMVDPGAVVQQYNRQQDQQASARRAEEERKKLQQQQAEELQQKQAAEQERLKQLEKERLAA
QEQQKQAEEAAKLAQQQQQQAEEAAKAAADAKKKAEAEAAKAAADAKKKAEAEAVKAAADAKKKAEAEAAKAAADAKK
KAEAEAAKAAAEAKKKAEAEAAKAAAEAKKKADAEAAKAAAEAKKKADAAAAKAAAEAKKKADAAAAKAAADAKKKAA
AEKAAAAEGVDDLLGDLSSGKNAPKTGGGAKGNGQPSK**
DSGTSGANGGATGADISAYAKQIQVAIQSRLYDASLYQGKQCVLHISLAPDGSLKSITSEGGDPALCQAALMAAKTAKIPKPPSQ
AVYEKIKDAKLDFKL

PPIIPRED:

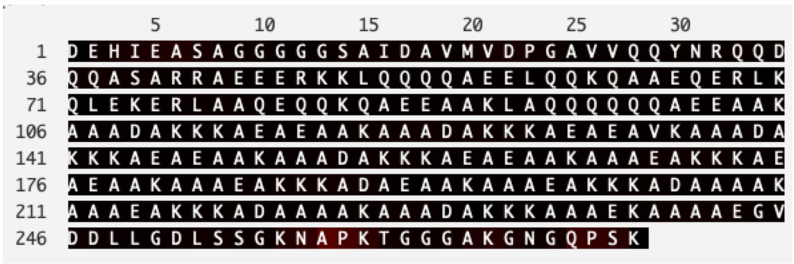

273 Domain II residues

3 Proline residues

229 α-Helix residues

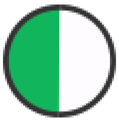

Escherichia coli K12

Locus: 774309-780629

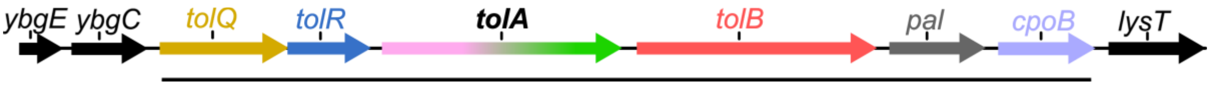

>AAC73833.1 Tol-Pal system protein TolA [Escherichia coli str. K-12 substr. MG1655]

MSKATEQNDKLKRAIIISAVLHVILFAALIWSSFDENIEASAGGGGGSSIDAVMVDSGAVVEQYKR
MQSQESSAKRSDEQRKMKEQQAAEELREKQAAEQERLKQLEKERLAAQEQKKQAEEAAKQAE
LKQKQAEEAAAKAAADAKAKAEADAKAAEEAAKKAAADAKKKAEAEAAKAAAEAQKKAEAAAA
ALKKKAEAAEAAAAEARKKAATEAAEKAKAEAEKKAAAEKAAADKKAAAEKAAADKKAAEKAAA
EKAAADKKAAAEKAAADKKAAAAKAAAEKAAAAKAAAEADDIFGELSSGKNAPKTGGGAKGNN
ASPAGSGNTKNNGASGADINNYAGQIKSAIESKFYDASSYAGKTCTLRIKLAPDGMLLDIKPEGG
DPALCQAALAAAKLAKIPKPPSQAVYEVFKNAPLDFKP

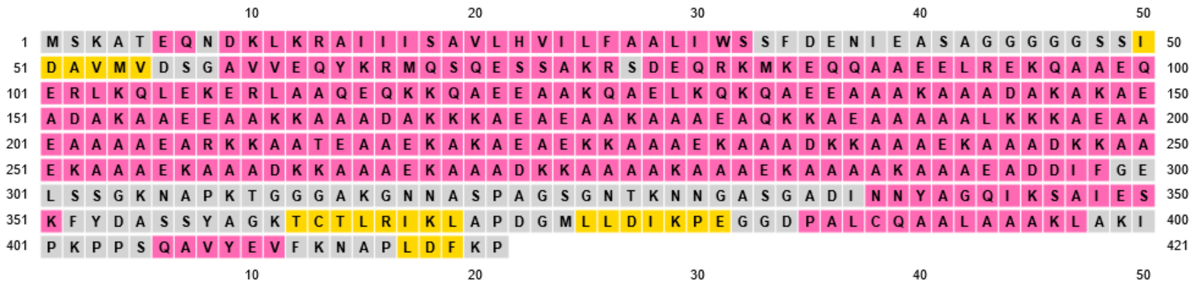

MSKATEQNDKLKRAIIISAVLHVILFAALIWS
**SFDENIEASAGGGGGSSIDAVMVDSGAVVEQYKRMQSQESSAKRSDEQRKMKEQQAAEELR
EKQAAEQERLKQLEKERLAAQEQKKQAEEAAKQAELKQKQAEEAAAKAAADAKAKAEADA
KAAEEAAKKAAADAKKKAEAEAAKAAAEAQKKAEAAAAALKKKAEAAEAAAAEARKKAAT
EAAEKAKAEAEKKAAAEKAAADKKAAAEKAAADKKAAEKAAAEKAAADKKAAAEKAAADK
KAAAAKAAAEKAAAAKAAAEADDIFGELSSGKNAPKTGGGAKGNNASP**
AGSGNTKNNGASGADINNYAGQIKSAIESKFYDASSYAGKTCTLRIKLAPDGMLLDIKPEGGDPA
LCQAALAAAKLAKIPKPPSQAVYEVFKNAPLDFKP

PPIIPRED:

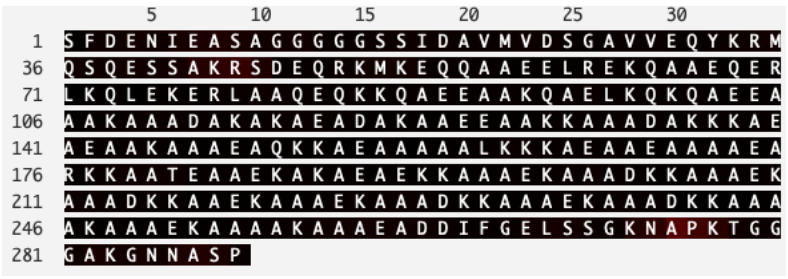

289 Domain II residues

2 Proline residues

239 helical residues

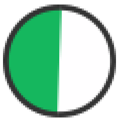

Enterobacter lignolyticus

Locus: 1485221-1491504

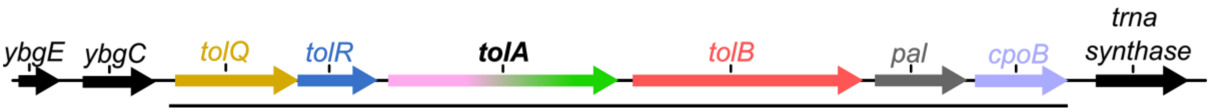

Flanked by an unspecified *ybgE* gene and tRNA synthase genes.

>ALR76035.1 protein TolA [[Enterobacter] lignolyticus]

MSKATEQNDKLKRAIIISVVLHIILIALLIWSSFDEHIDASAGGGGGSSIDAVMVDPGAVVQNYNRQ
QQQAASAKRAQEQREKQAQQQAEEMREKQAAEQERLKQLEQEKLQAQEAAKQAQEQQKQA
QEQQKQAEQAAAKAAADAKAKAEADAKLAAEAAKKAAADAQKKAEAEAVKAAAAAQKKAEADA
AKAAADAQKKAEAEAAKKAQQEAEKKAAADAAKKAAAAEKAAAEKAAAEQAAAEKAAADKAEK
AAKAAAEKKAAAAEKAAAEKAAKAAAAKKAAEAKEASGVDDLLGDLSSGKNAPKTGGGAKGKG
QPAKDSGTSGANGGATGADISAYAAQIRSAIQSRLYDSSLYAGQQCDLHISLAPDGTLKSITSAG
GDPALCQAALTAAKTATIPKPPSQAVYEKIKNATLGFKL

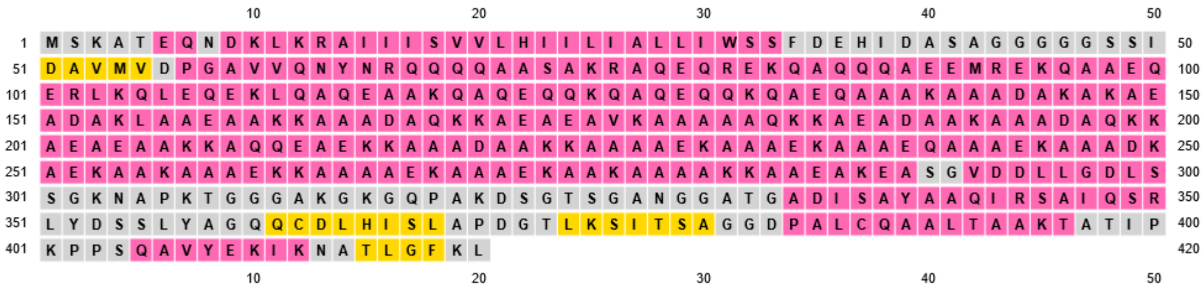

MSKATEQNDKLKRAIIISVVLHIILIALLIWSS
**FDEHIDASAGGGGGSSIDAVMVDPGAVVQNYNRQQQQAASAKRAQEQREKQAQQQAEEMR
EKQAAEQERLKQLEQEKLQAQEAAKQAQEQQKQAQEQQKQAEQAAAKAAADAKAKAEAD
AKLAAEAAKKAAADAQKKAEAEAVKAAAAAQKKAEADAAKAAADAQKKAEAEAAKKAQQ
EAEKKAAADAAKKAAAAEKAAAEKAAAEQAAAEKAAADKAEKAAKAAAEKKAAAAEKAAA
EKAAKAAAAKKAAEAKEASGVDDLLGDLSSGKNAPKTGGGAKGKGQPAK**
DSGTSGANGGATGADISAYAAQIRSAIQSRLYDSSLYAGQQCDLHISLAPDGTLKSITSAGGDPA
LCQAALTAAKTATIPKPPSQAVYEKIKNATLGFKL

PPIIPRED:

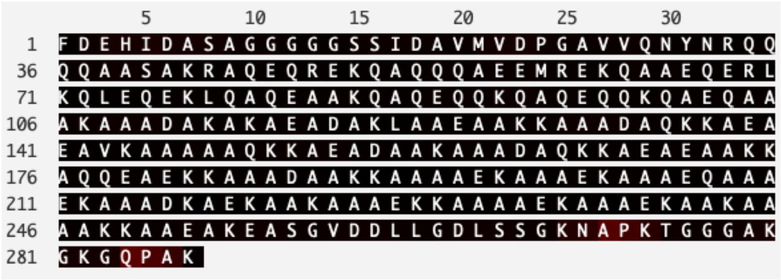

287 Domain II residues

3 Proline residues

242 α-Helix residues

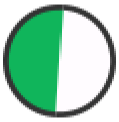

Yersinia pestis

Locus: 683535-689859

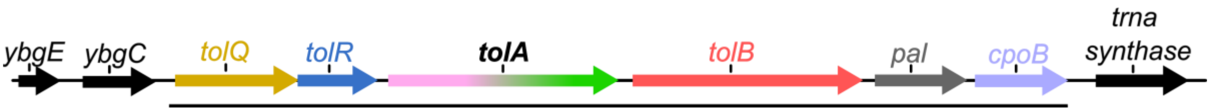

Flanked by *ybgE* cytochrome d oxidase subunits, and a tRNA synthase gene.

>ABG12569.1 TolA colicin import membrane protein [Yersinia pestis Antiqua]

MWKSSVGKATEQNDKLNRAVIVSVVLHIILIALLIWGSLTQTTEMGGGGAGGEVIDAVMVDPGAV
TEQYNRQQQQQTDAKRAEQQRQKKAEQQAEELQQKQAAEQQRLKELEKERLQAQEDAKLAA
EEQKKQVAEQQKQIAEQQKQAAEQQKIAAAAVAKAKEEQKQAETAAAQAKAEADKIVKAQAEA
QKKAEAEAKKEAAVAAAAKKQADADAKKAVEVAEKAAADAAEKKAAADAEKKAAAAKKVAAAA
EAKKKAAAEAAASTDVDDLFGGLANAKNAPKSGSGAGAAAAGKGGGKKSGASGADISGYLGQI
TGAIQSKFYDADLYKGRTCDLRIKLAPDGLLIDVKAEGGDPALCQAAIAAAKQAKIPKPPSTDVYE
QFKNAPLVFKPQ

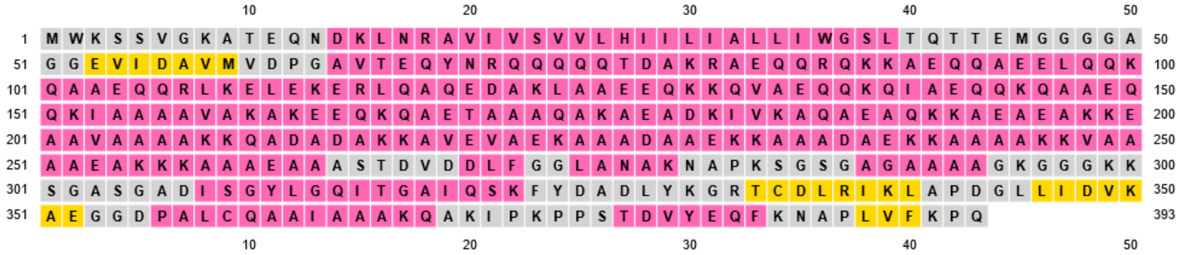

MWKSSVGKATEQNDKLNRAVIVSVVLHIILIALLIWGSL
**TQTTEMGGGGAGGEVIDAVMVDPGAVTEQYNRQQQQQTDAKRAEQQRQKKAEQQAEELQQ
KQAAEQQRLKELEKERLQAQEDAKLAAEEQKKQVAEQQKQIAEQQKQAAEQQKIAAAAVAK
AKEEQKQAETAAAQAKAEADKIVKAQAEAQKKAEAEAKKEAAVAAAAKKQADADAKKAVE
VAEKAAADAAEKKAAADAEKKAAAAKKVAAAAEAKKKAAAEAAASTDVDDLFGGLANAKN
APKSGSGAGAAAA**
GKGGGKKSGASGADISGYLGQITGAIQSKFYDADLYKGRTCDLRIKLAPDGLLIDVKAEGGDPAL
CQAAIAAAKQAKIPKPPSTDVYEQFKNAPLVFKPQ

PPIIPRED:

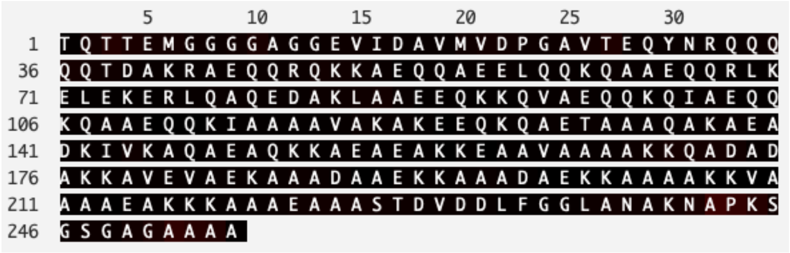

254 Domain II residues

2 Proline residues

214 α-Helix residues

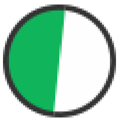

Buchnera aphidicola

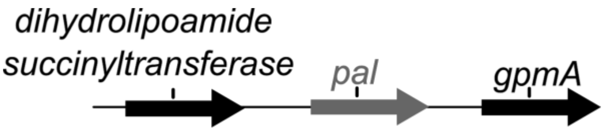

Only *pal* detected in genome.

Flanked by genes for dihydrolipoamide succinyltransferase and a phosphoglyceromutase.

Pasteuralleles

Haemophilus ducreyi

- TolA pseudogene found in genome (locus 1488353) with frameshift truncation

Therefore performed analysis on relative:

Haemophilus influenzae

Locus: 402127

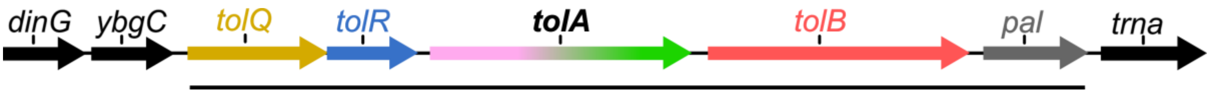

Flanked by *dinG* encoding an ATP-dependent helicase and tRNA synthase genes.

BLAST found no CpoB.

>AAC22041.1 outer membrane integrity protein (tolA) [Haemophilus influenzae Rd KW20]

MQNNRQKKGINAFAISILLHFILFGLLILSSLYHTVEIMGGGEGEGDVIGAVIVDTGTAAQEWGRIQQQKKGQADKQ
KRPEPVVEEKPPEPNQEEIKHQQEVQRQEELKRQQEQQRQQEIKKQQEQARQEALEKQKQAEEAKAKQAAEAA
KLKADAEAKRLAAAAKQAEEEAKAKAAEIAAQKAKQEAEAKAKLEAEAKAKAVAEAKAKAEAEAKAKAAAEAKAK
ADAEAKAATEAKRKADQASLDDFLNGGDIGGGSASKGGNTNKGGTQGSGAALGSGDGGKVGDQYAGVIKKEIQ
RRFLKDPNFAGKVCRIKIQLGRDGTILGYQKISGSDDICSAALSAVARTKKVPAAPSDEIYEKYKSPIIDFDIR

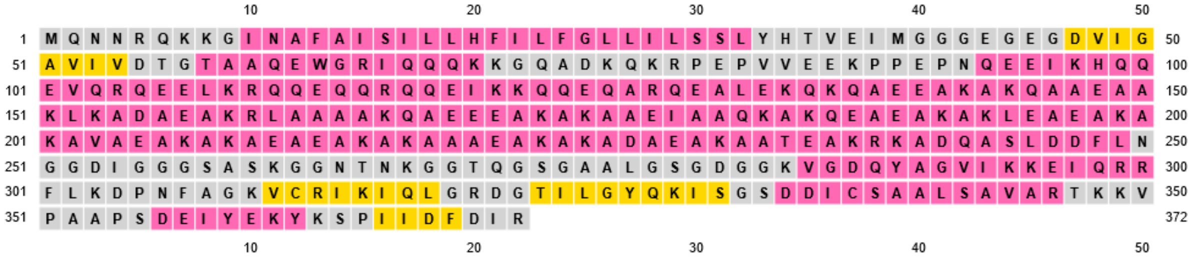

MQNNRQKKGINAFAISILLHFILFGLLILSSL
YHTVEIMGGGEGEGDVIGAVIVDTGTAAQEWGRIQQQKKGQADKQKRPEPVVEEKPPEPNQEEIKHQQEVQR
QEELKRQQEQQRQQEIKKQQEQARQEALEKQKQAEEAKAKQAAEAAKLKADAEAKRLAAAAKQAEEEAKA
KAAEIAAQKAKQEAEAKAKLEAEAKAKAVAEAKAKAEAEAKAKAAAEAKAKADAEAKAATEAKRKADQASL
DDFLNGGDIGGGSASKGGNTNKGGTQG
SGAALGSGDGGKVGDQYAGVIKKEIQRRFLKDPNFAGKVCRIKIQLGRDGTILGYQKISGSDDICSAALSAVARTK
KVPAAPSDEIYEKYKSPIIDFDIR

PPIIPRED:

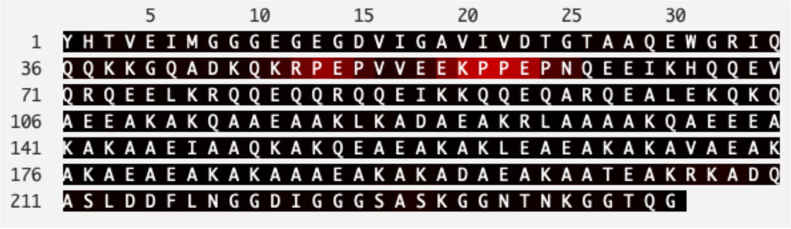

240 Domain II residues

5 Proline residues

170 α-Helix residues

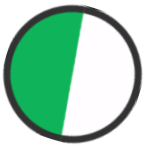

Pasteurella multocida

Locus: 1136139- 1144045

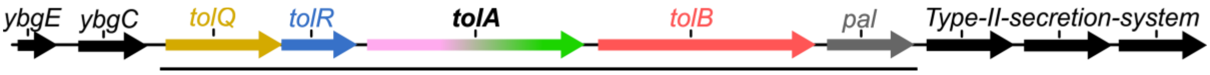

Flanked by *ybgE* component of the cytochrome d oxidase complex and Type-II Secretion system genes *PM0965* (structure), *pulJ*, *PM0963*. May suggest Tol-Pal implicated in T2SS in Pasteruella. BLAST found no CpoB.

>AAK03052.1 TolA [Pasteurella multocida subsp. multocida str. Pm70]

MQSKQNNKDISAVILSVILHAILFGLLILGSLYHRVEIMGGGEGDSEVMGAVMVDTGAAAQEWGR
LQQQKKGQTDKAKKTEPVIEEKPVQPDEQEIIRQQEIEKQKALEKQKEIEQQKEIERQKQLAEQK
KQEEQARLAALEKQKQAEEAKAKQLAEAAKLKAEAEAKRLAALAKQAEEEAKAKAAEEAKRKAE
KAKAEAEAKAKVEKAKAEAEAKVKAEKAKAEAEAKAKAEKAKAEAKAKAEKAKADAEAAQRKAN
QAALDDFFSGGDVGGGSATRGSNTDRQGSQGSGAALGAGDGGKTGDQYAGVIKREIQRRFLK
EPSFANKVCVVEVEFLRDGTIANYRRVSGPDDICQAAVSAVARTKKVPPAPTDDIYQRYKKSPIE
FKLR

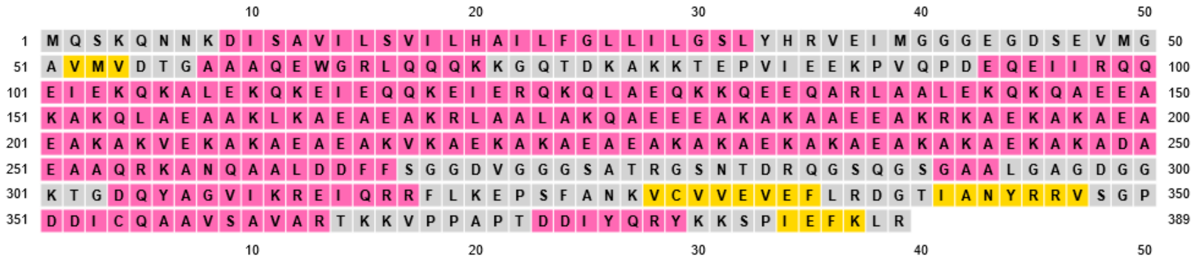

MQSKQNNKDISAVILSVILHAILFGLLILGSL
**YHRVEIMGGGEGDSEVMGAVMVDTGAAAQEWGRLQQQKKGQTDKAKKTEPVIEEKPVQPD
EQEIIRQQEIEKQKALEKQKEIEQQKEIERQKQLAEQKKQEEQARLAALEKQKQAEEAKAKQL
AEAAKLKAEAEAKRLAALAKQAEEEAKAKAAEEAKRKAEKAKAEAEAKAKVEKAKAEAEAK
VKAEKAKAEAEAKAKAEKAKAEAKAKAEKAKADAEAAQRKANQAALDDFFSGGDVGGGSA
TRGSNTDRQGSQG**
SGAALGAGDGGKTGDQYAGVIKREIQRRFLKEPSFANKVCVVEVEFLRDGTIANYRRVSGPDDI
CQAAVSAVARTKKVPPAPTDDIYQRYKKSPIEFKLR

PPIIPRED:

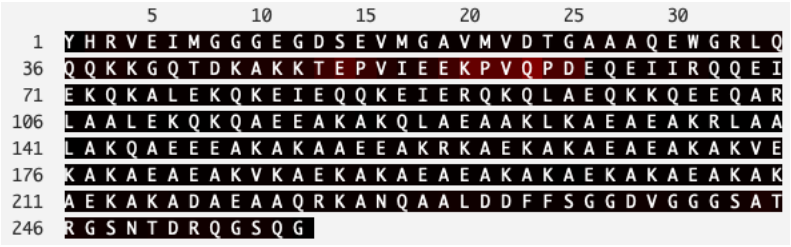

257 Domain II residues

3 Proline residues

187 α-Helix residues

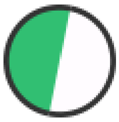

Vibrionales

Vibrio nigripulchritudo

Locus: 2406873- 2413978

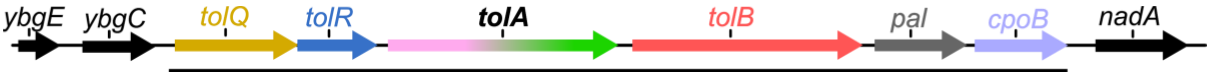

Flanked by *ybgE*, part of the cytochrome d oxidase assembly, and *nadA*, encoding a quinolinate synthase.

>CCO58331.1 TolA protein [Vibrio nigripulchritudo]

MKNKNNYTSAITISAVLHGVLIVGLLFGTDFSMPEQDQTGNMVQAVVIDPQMVQQQAREIRQQR
EAAQRAEQERLDKLRKQSDQLEKDRKAEEERIRKLKEQKVKAEKEAREAEKARQKKEQERKAA
EERAKKERERVAKAEAERKAKEEAVEKAEQERLKKEAAIAKAEEERVAREKAAKEAEEKARRER
EAAEKAEAERIAKEKAAKEAAEKARKEKERLERLERERKEQEAALNDIFAGLESESSQNSAAKQ
QHVTSEVQRYGAIYKQLIQSRLLKDDSFLGKSCRVNMRLIHTGNSAIVNGVKVLGGDSRVCAAT
KSAIAQVASFPVPKEKDVADQLKNINLTVELD

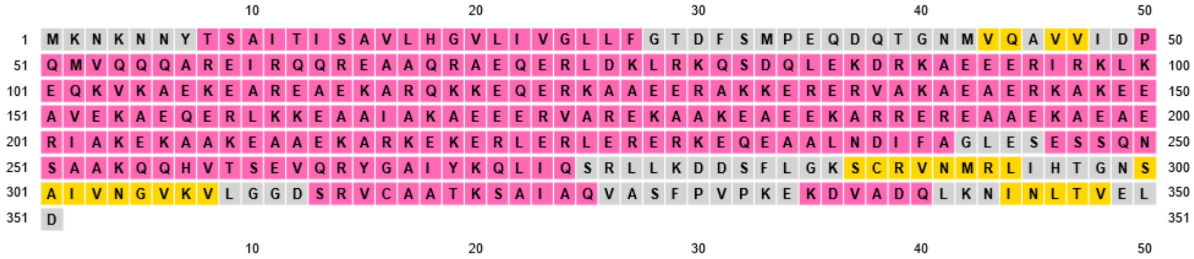

MKNKNNYTSAITISAVLHGVLIVGLLF
**GTDFSMPEQDQTGNMVQAVVIDPQMVQQQAREIRQQREAAQRAEQERLDKLRKQSDQLEKD
RKAEEERIRKLKEQKVKAEKEAREAEKARQKKEQERKAAEERAKKERERVAKAEAERKAKE
EAVEKAEQERLKKEAAIAKAEEERVAREKAAKEAEEKARREREAAEKAEAERIAKEKAAKEA
AEKARKEKERLERLERERKEQEAALNDIFAGLESESSQNS**
AAKQQHVTSEVQRYGAIYKQLIQSRLLKDDSFLGKSCRVNMRLIHTGNSAIVNGVKVLGGDSRV
CAATKSAIAQVASFPVPKEKDVADQLKNINLTVELD

PPIIPRED:

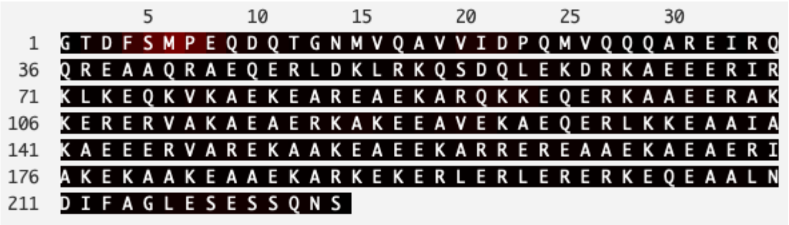

224 Domain II residues

2 Proline residues

198 α-Helix residues

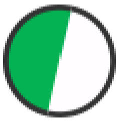

Aeromondales

Aeromonas hydrophila

Locus: 4069690- 4077375

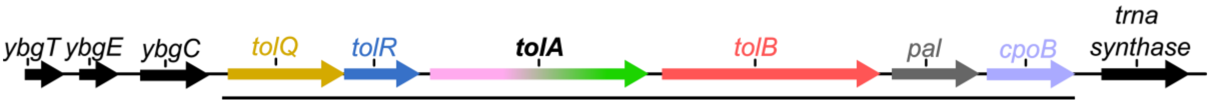

Flanked by *ybgT*, *ybgE* and tRNA synthase genes.

>ABK36017.1 protein TolA [Aeromonas hydrophila subsp. hydrophila ATCC 7966]

MDVKRGISGYLIASLLLHLIIGIILFVGLDFSKPKRPESKGQIVNAVMLDENFLNEQAKQIQQQKSQ
PKPQKVEKEKDKEDTDIAKRELAQQQERLRIAETKRKEAEEATRKAEAEKQKKVAEQKQAEEKA
QKAEEARKLEEQKAKKVEAERKAAEEESKALALKKKKEQEKKEAEEKQAKADAAKKAEAEKKAK
QEAEKKAKAEADKKAKAEAEKKAKAEADKKAKEAKEEAAKKAKADAEKKAKAEADKQAKIAAER
KRKAAEEAKLQKEMEDMMQQQLAAEANARSQAASAAAQGEVDKYAALIKATVERYMILDPTMR
GKTCTIGVRLASTGFVISVDNGQGDPAVCRSGKAAVLKANQLPVPKDPAAFEMLKEFNLKLEPSI

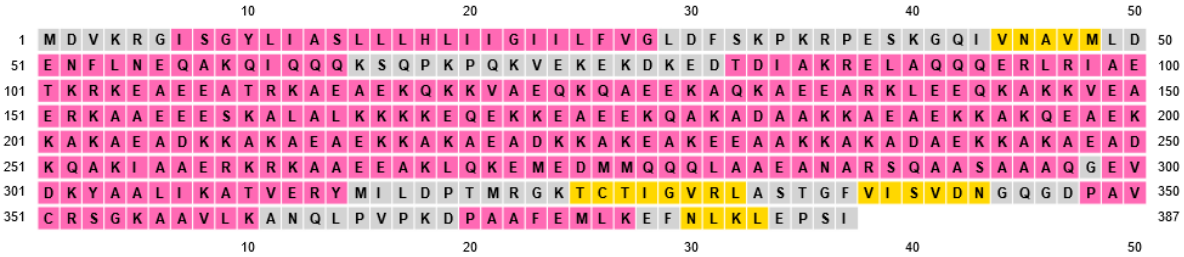

MDVKRGISGYLIASLLLHLIIGIILFVG
LDFSKPKRPESKGQIVNAVMLDENFLNEQAKQIQQQKSQPKPQKVEKEKDKEDTDIAKRELA QQQERLRIAETKRKEAEEATRKAEAEKQKKVAEQKQAEEKAQKAEEARKLEEQKAKKVEAE RKAAEEESKALALKKKKEQEKKEAEEKQAKADAAKKAEAEKKAKQEAEKKAKAEADKKAK AEAEKKAKAEADKKAKEAKEEAAKKAKADAEKKAKAEADKQAKIAAERKRKAAEEAKLQKE MEDMMQQQLAAEANA
RSQAASAAAQGEVDKYAALIKATVERYMILDPTMRGKTCTIGVRLASTGFVISVDNGQGDPAVC
RSGKAAVLKANQLPVPKDPAAFEMLKEFNLKLEPSI

PPIIPRED:

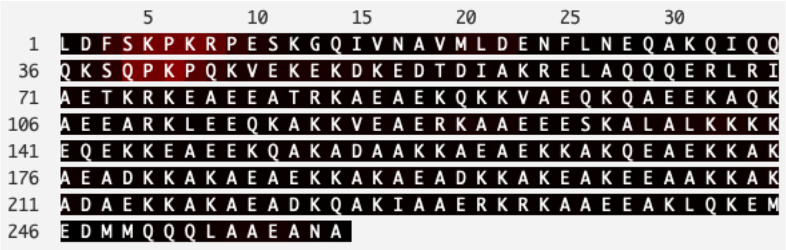

259 Domain II residues

4 Proline residues

220 α-Helix residues

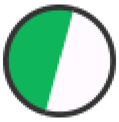

Alteromondales

Shewanella oneidensis

Locus: 2867620- 2875435

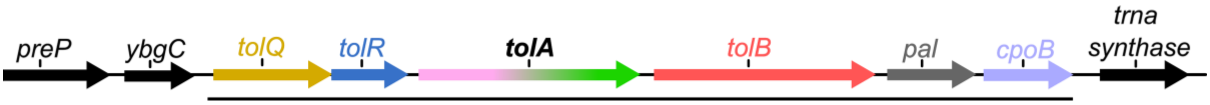

Flanked by *preP* encoding a prolyl endopeptidase and a tRNA synthase gene.

>AAN55777.1 TolA energy-transducing system inner membrane component TolA [Shewanella oneidensis MR-1]

MADNSNVALPLSISAGIHIGVIIILAIGIDFTHKPEPVQQVSAPAVKAVMVDQQQVANQVEKLKQEK
RDTERRERERQAELERKAQEAKQAREREQAQLKQLAEERKQQEIETQKANEAAKAAQLKQQQ
EKEKAQKAEADRKLKEQERKLAEDAAQKAAEKRKVEEAAVAKAEADRKQKEAEAKAKAEADAK
AKADKAKADAEAKAKAEAKAKADAKAKADAEAKALAQQEQEMADALAAEQAALSQTMNKQMQ
TEVGKYTAMIKSTIQRNLVVDESMRGKTCTVSVRLANDGFVISSQTQGGDPNVCRATKAAILKA
GKLPVSPDPAVYNLMKEINLIVEPTFN

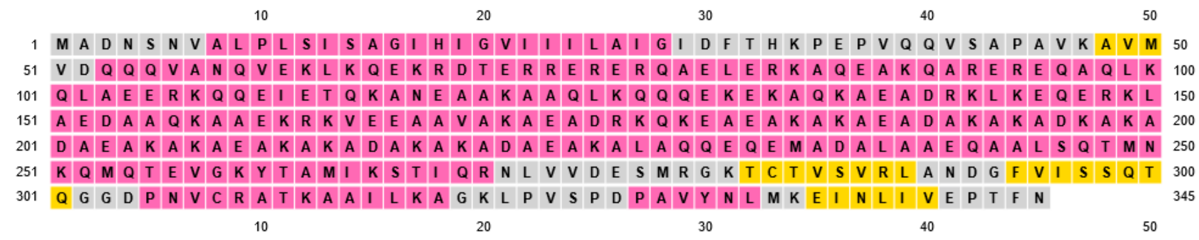

MADNSNVALPLSISAGIHIGVIIILAIG
**IDFTHKPEPVQQVSAPAVKAVMVDQQQVANQVEKLKQEKRDTERRERERQAELERKAQEAK
QAREREQAQLKQLAEERKQQEIETQKANEAAKAAQLKQQQEKEKAQKAEADRKLKEQERK
LAEDAAQKAAEKRKVEEAAVAKAEADRKQKEAEAKAKAEADAKAKADKAKADAEAKAKAE
AKAKADAKAKADAEAKALAQQEQEMADALAAEQAAL**
SQTMNKQMQTEVGKYTAMIKSTIQRNLVVDESMRGKTCTVSVRLANDGFVISSQTQGGDPNVC
RATKAAILKAGKLPVSPDPAVYNLMKEINLIVEPTFN

PPIIPRED:

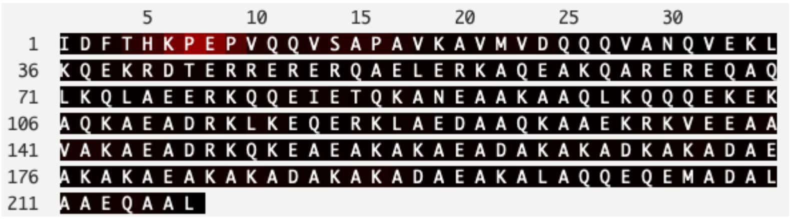

217 Domain II residues

3 Proline residues

193 α-Helix residues

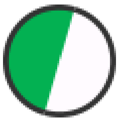

Ferrimonas balearica

Locus: 2558978- 2566779

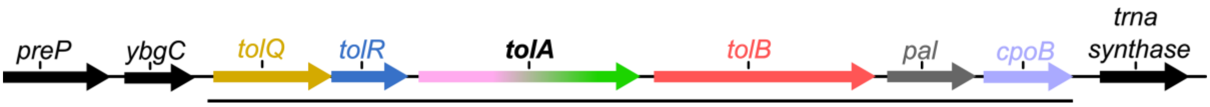

Flanked by *preP*, encoding a prolyl protease with a β-propeller structure, and a tRNA synthase gene.

>ADN76491.1 Cell division and transport-associated protein TolA [Ferrimonas balearica DSM 9799]

MSKQTQPGQSPIGIPLAVSVLLHAGLLGLLAVSVDFSSKPKPQPQASAPVVQAVTVDKAQVEQH
VKRIQAEQAAAREAERQRQADLERQAKEAERKRQAEQDRLRKLEQERQQRQAEVKRAEEAAK
QAKIKQQEEEKRARQAAEQRAKEEAARQEAERKRKAEEEAARKAEAERKRKEEERKRKEAEEA
ARKAREAELAAQMAAEQAELAQARQQQVLTEVERYSVLIRQVIQRNLRTDTSMRGKQCRVRINL
APDGFVFDSRVLSGDPGVCRATQAAIQAAGQLPVSKEPDVYNQLKQIDLTVEPEL

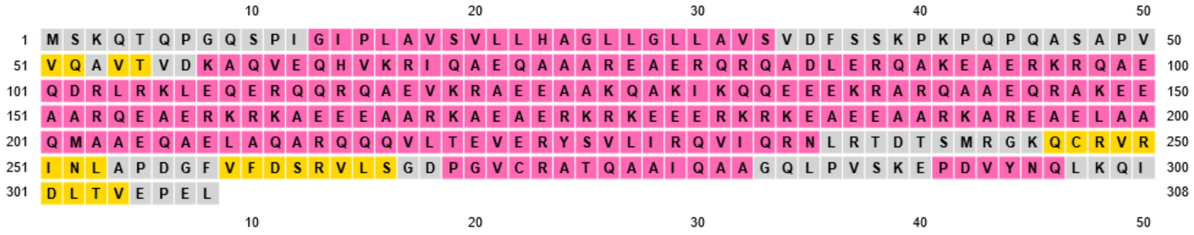

MSKQTQPGQSPIGIPLAVS
VLLHAGLLGLLAVSVDFSSKPKPQPQASAPVVQAVTVDKAQVEQHVKRIQAEQAAAREAERQ RQADLERQAKEAERKRQAEQDRLRKLEQERQQRQAEVKRAEEAAKQAKIKQQEEEKRARQ AAEQRAKEEAARQEAERKRKAEEEAARKAEAERKRKEEERKRKEAEEAARKAREAELAAQ MAAEQAE
LAQARQQQVLTEVERYSVLIRQVIQRNLRTDTSMRGKQCRVRINLAPDGFVFDSRVLSGDPGVC RATQAAIQAAGQLPVSKEPDVYNQLKQIDLTVEPEL

PPIIPRED:

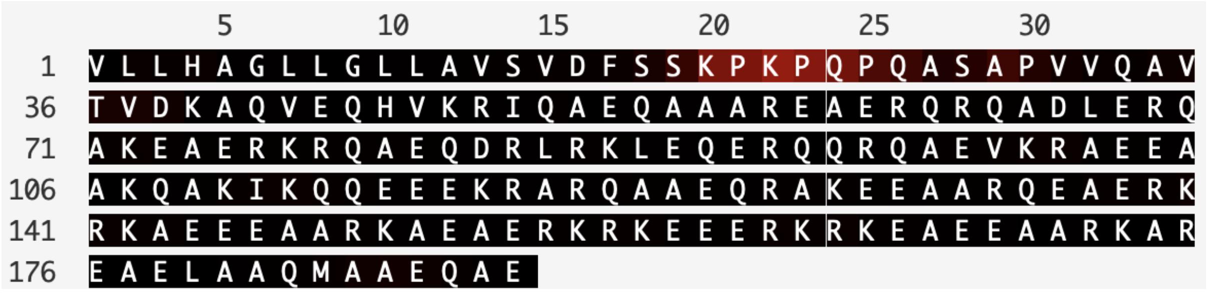

175 Domain II residues

4 Proline residues

151 α-Helix residues

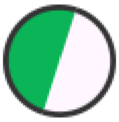

Noted a second *tolB*, also is the case for other species too but have not documented this.

Idiomarina loihiensis

Locus: 1163996- 1170927

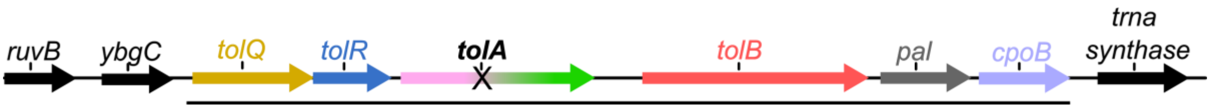

Flanked by *ruvB* and tRNA synthase gene.

>AAV81921.1 TolA-like protein [Idiomarina loihiensis L2TR]

MEVNLDQSELDMSEEIVSAVTVDKSKVQQQADKIRQQKAEQEAAEQRRIERLERRAEEARKAR
EREEQRKKEIERQQELEKQEAAEARKQAERERKEAERLAQERAKAEAAAKEAERRKREAEEAE
RRAEEERRKRLEEQRKREQEQREAAEREAQLQKEMEEERAR
RQAARRQQVLSEVEKYQALIQQTIQRNWNVDDSMQGKSCELTIRVAPSGFVKSVDTGSGDANV
CRSAQNAVLKATTLPVSEDPEIYEQMSTIKLTVKPQL

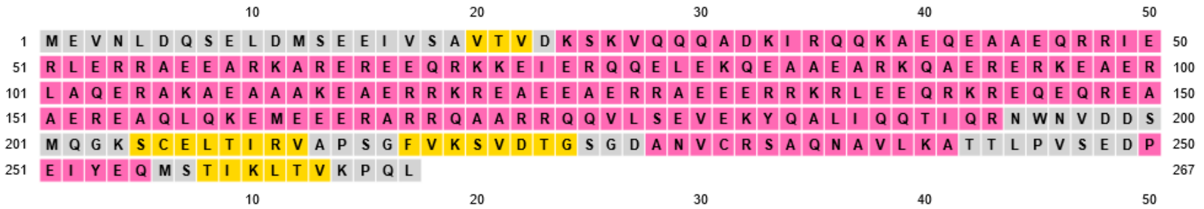

MEVNLDQSELDMSEEIVSAVTVDKSKVQQQADKIRQQKAEQEAAEQRRIERLERRAEEARKA
REREEQRKKEIERQQELEKQEAAEARKQAERERKEAERLAQERAKAEAAAKEAERRKREAE
EAERRAEEERRKRLEEQRKREQEQREAAEREAQLQKEMEEERAR
RQAARRQQVLSEVEKYQALIQQTIQRNWNVDDSMQGKSCELTIRVAPSGFVKSVDTGSGDANV
CRSAQNAVLKATTLPVSEDPEIYEQMSTIKLTVKPQL

Analysis performed with caveat that the protein is non-functional.

PPIIPRED:

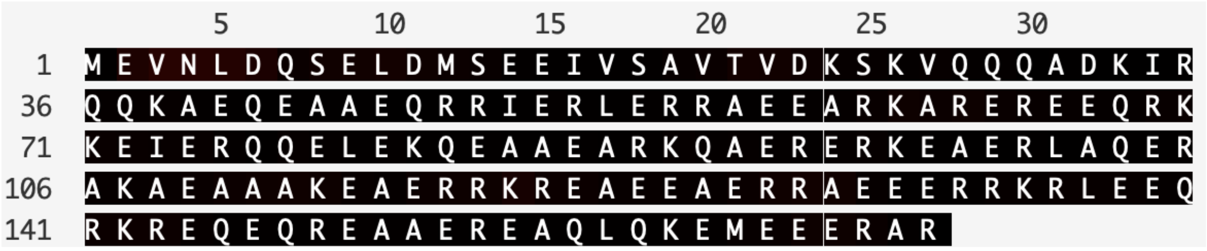

167 "Domain II residues"

0 Proline residues

144 α-Helix residues

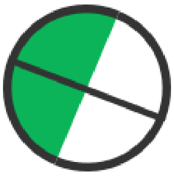

Verified by downloading Alphafold prediction- Shows no hydrophobic helix region

VTV (residues 20-23) appears to be the stator box

Colwellia psychrerythraea

Locus: 1775322- 1783074

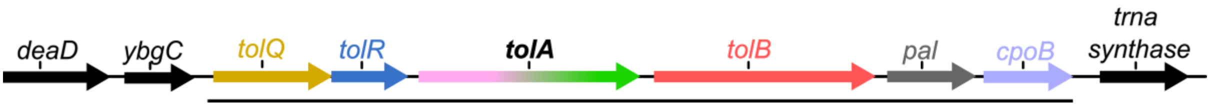

Flanked by *deaD*, encoding an RNA helicase, and a tRNA synthase gene.

>AAZ24973.1 putative tolA protein [Colwellia psychrerythraea 34H]

MKSPYFKAVWLSIALHVVLLVGLLSGDFSSEPKPLPTPTPTSQSAEPIKAVVIDKAKFEQAVNKIKRQ
KINERDAEKKRLKAVEKRASDAKKRRVQEQARIKKLEKQRKQKEQEKIKADKAAKSSKAKAAKA
EKVRKQKEQEKQVAEKAAAAARSKRIKEEADAKKAEDLRLKKIADRKRQEIAAKEQAIQDAMLAE
QMADEMATRNKARHQQVMTEVQRYSALIQQSINQRMITDRSTMADKSCRLTLTLAPSGFVIDVK
VGQGDKVVCDAANIAIGKAGTLPVSKDPEVFKEMREIAVTVEPKF

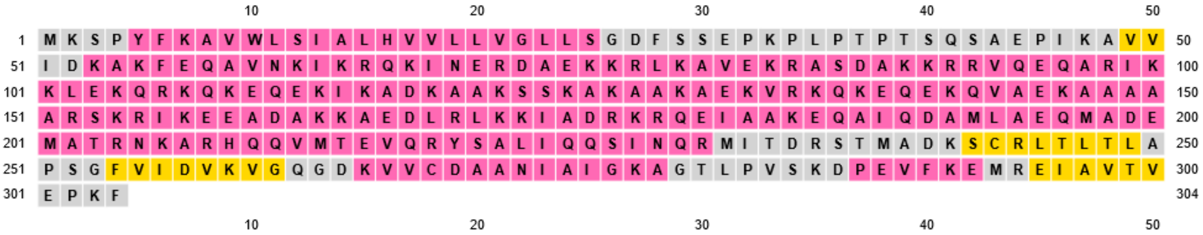

MKSPYFKAVWLSIALHVVLLVGLLS
GDFSSEPKPLPTPTPTSQSAEPIKAVVIDKAKFEQAVNKIKRQKINERDAEKKRLKAVEKRASDAK
KRRVQEQARIKKLEKQRKQKEQEKIKADKAAKSSKAKAAKAEKVRKQKEQEKQVAEKAAAA
ARSKRIKEEADAKKAEDLRLKKIADRKRQEIAAKEQAIQDAMLAEQMADEMATR
NKARHQQVMTEVQRYSALIQQSINQRMITDRSTMADKSCRLTLTLAPSGFVIDVKVGQGDKVVC
DAANIAIGKAGTLPVSKDPEVFKEMREIAVTVEPKF

PPIIPRED:

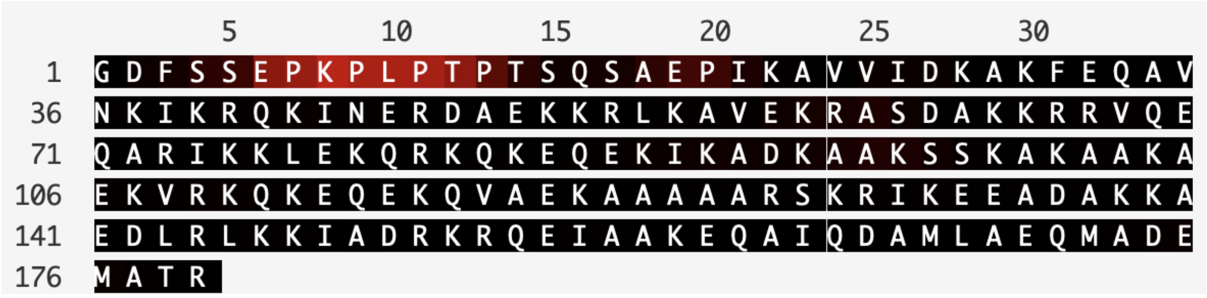

179 Domain II residues

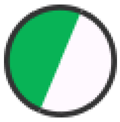

5 Proline residues

152 α-Helix residues

Alteromonas macleodii

Locus: 1540250- 1547397

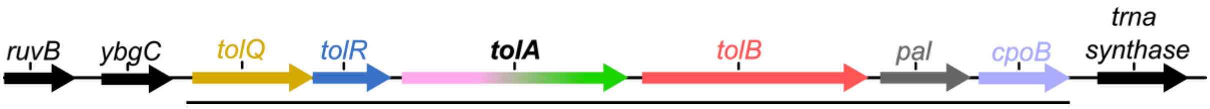

Flanked by *ruvB*, and a tRNA synthase gene.

>CAI3945313.1 Cell division and transport-associated protein TolA [Alteromonas macleodii]

MSKPEVSKSTSTASHSNNASNGVSGDKKNGKSAKQANSISPEQVGLYKSIGLHLFIAALLLISVSF
SPDPLPSMPSNAPVIEATFIDAQAIADQKREQAQAEAQARAAEQERQRKAEAAAEKKRQQQLAA
KRAKEKREAEAAEAKRQKDLERLAAQKEQERKEREAKAKAEAERKKKEAAERAEMERIMQEQL
AKEQAAQQERRRKQVLTEVERYTALIQQTIKRNLYSDDSYQGKTCRLNIRLATTGFVTSIRVLGG
NDALCRAAESAVRRAEKLPVSDAPDVYEQLKDITLKVEL

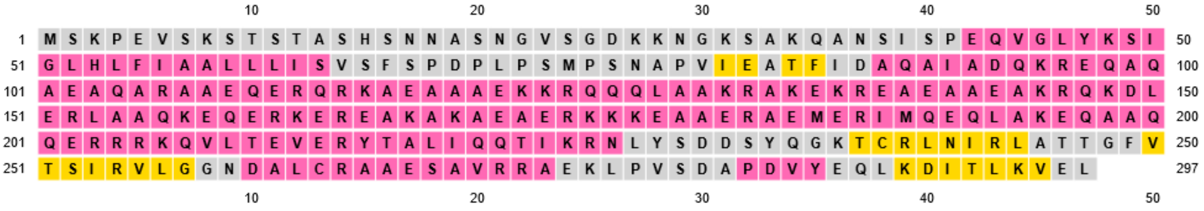

MSKPEVSKSTSTASHSNNASNGVSGDKKNGKSAKQANSISPEQVGLYKSIGLHLFIAALLLIS
**VSFSPDPLPSMPSNAPVIEATFIDAQAIADQKREQAQAEAQARAAEQERQRKAEAAAEKKRQ
QQLAAKRAKEKREAEAAEAKRQKDLERLAAQKEQERKEREAKAKAEAERKKKEAAERAEM
ERIMQEQLAKEQ**
AAQQERRRKQVLTEVERYTALIQQTIKRNLYSDDSYQGKTCRLNIRLATTGFVTSIRVLGGNDAL
CRAAESAVRRAEKLPVSDAPDVYEQLKDITLKVEL

PPIIPRED:

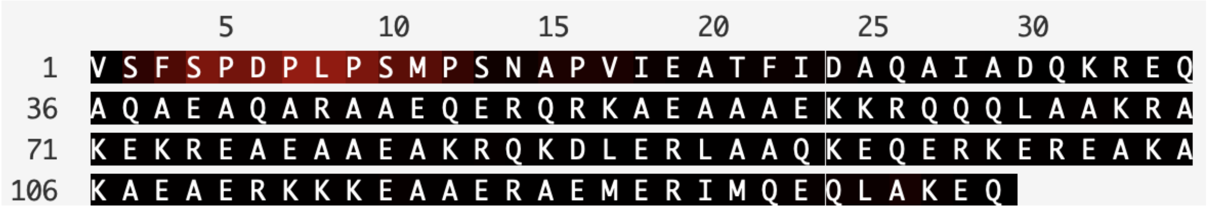

134 Domain II residues

5 Proline residues

110 α-Helix residues

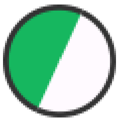

----- has cytoplasmic (N-terminal) disordered region

Pseudoalteromonas atlantica

Locus: 3560104- 3567338

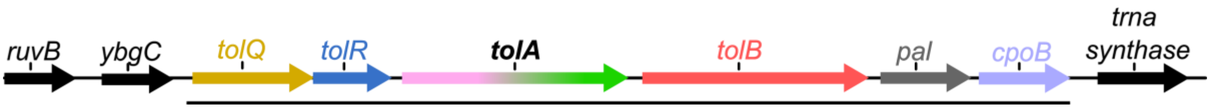

Flanked by *ruvB* and a tRNA synthase gene.

TolB may be a lipoprotein:

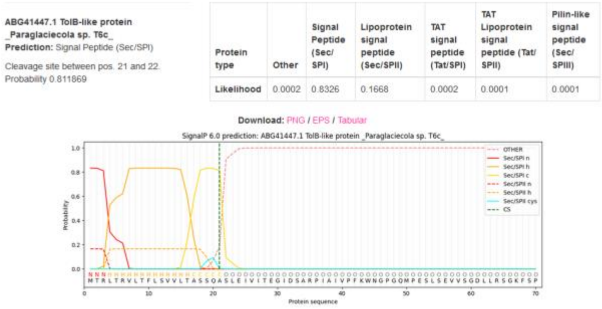

>ABG41448.1 Cell division and transport-associated protein TolA [Paraglaciecola sp. T6c]

MKLKLSLPLIKSLSLHLVFGVLLFAGMDVKLPQETLEMASSQPVIEAVAVDANAVDDQIKRIEDQK
KRKQKVEEDRIAELERRANEAERKRKQQEQEAVEIEQRTKRQREERKQAEQAAIAARKKQERE
KAKAKAAEAERKRKELERKKAEERARKAKEAREKEEKALKEAQRKKAEAAEKARQERALQDQL
EAEQAVRQQRRSKQVLTEVQKYQALIHQAIQRQLIVDNSMRGKSCQLNIRLASSGLVIQVKELG
GDPILCRAAKSAVFKAGTLPVSKEADVYEKLRDINLTVEPDL

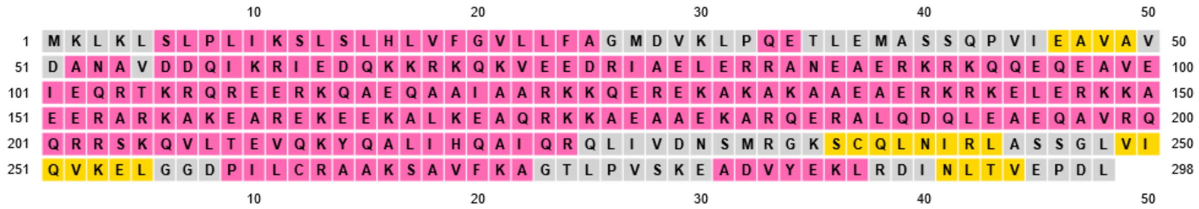

MKLKLSLPLIKSLSLHLVFGVLLFA
**GMDVKLPQETLEMASSQPVIEAVAVDANAVDDQIKRIEDQKKRKQKVEEDRIAELERRANEAE
RKRKQQEQEAVEIEQRTKRQREERKQAEQAAIAARKKQEREKAKAKAAEAERKRKELERKK
AEERARKAKEAREKEEKALKEAQRKKAEAAEKARQERALQDQLEAEQAV**
RQQRRSKQVLTEVQKYQALIHQAIQRQLIVDNSMRGKSCQLNIRLASSGLVIQVKELGGDPILCR
AAKSAVFKAGTLPVSKEADVYEKLRDINLTVEPDL

PPIIPRED:

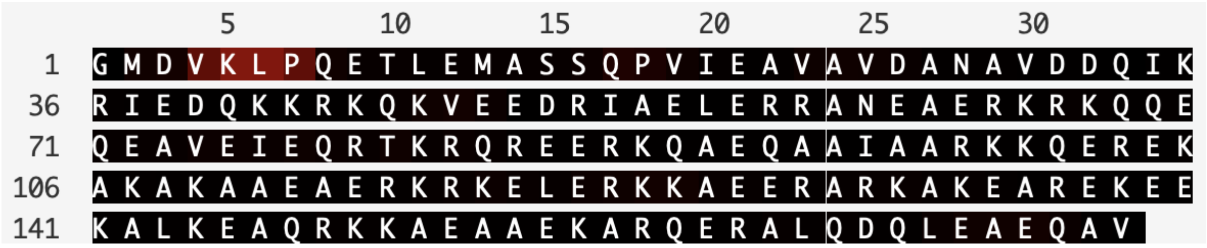

173 Domain II residues

2 Proline residues

148 α-Helix residues

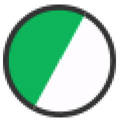

Iteromondales

Psychromonas ingrahamii

Locus: 916930-922989

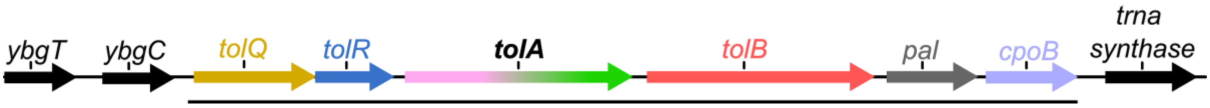

Flanked by the *ybgT* gene (encoding a subunit for the cytochrome d oxidase complex), and a tRNA synthase genes.

>ABM02578.1 Cell division and transport-associated protein TolA [Psychromonas ingrahamii 37]

MKNRSTLIAFIGALLMHIIVGTLLLLNVNFSNPKIKPKNEVPIINATVVDQKLLDNLAQRQSEKKQAE
RKKVEQEKQRIEQEKKKAAERVAAEKKQAAEKLKAKQDAEKAKKVAAEKAAAEKVAAEKVAAEK
VAAEKVAAEKAAAEKAAAEKAAAEKAAAEKAAAEKAAAEKAAAAAKVKAEQEKERLRRAELDKQ
MDAQFEDTFSNAQSSRQLSEIARYTALIQDKIGRNWQIEPSMKGKSCTLSIRLAADGLVISAKRS
SGDIQLCESAKRAALKANTLPIPKDPDISAKFRDFDLNLKPEF

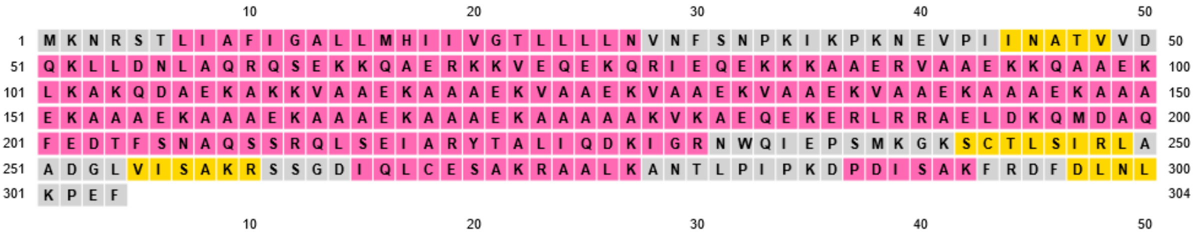

MKNRSTLIAFIGALLMHIIVGTLLLLN
**VNFSNPKIKPKNEVPIINATVVDQKLLDNLAQRQSEKKQAERKKVEQEKQRIEQEKKKAAERV
AAEKKQAAEKLKAKQDAEKAKKVAAEKAAAEKVAAEKVAAEKVAAEKVAAEKAAAEKAAA
EKAAAEKAAAEKAAAEKAAAEKAAAAAKVKAEQEKERLRRAELDKQMDAQFEDT**
FSNAQSSRQLSEIARYTALIQDKIGRNWQIEPSMKGKSCTLSIRLAADGLVISAKRSSGDIQLCES
AKRAALKANTLPIPKDPDISAKFRDFDLNLKPEF

PPIIPRED:

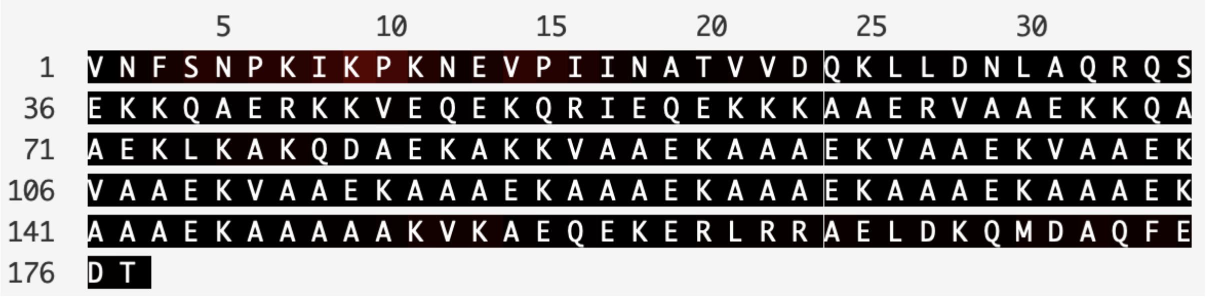

177 Domain II residues

3 Proline residues

154 α-Helix residues

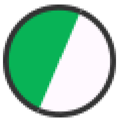

Pseudomonodales

Psychrobacter arcticus

Split system.

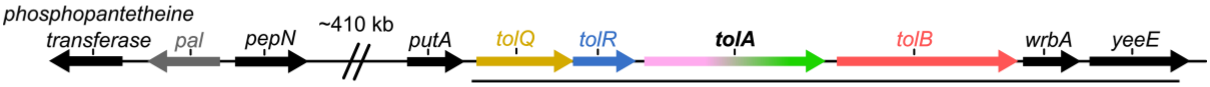

Loci: *tolQRAB* 1497926- 1510048 *pal*: 1088176

tolQRAB flanked by *putA* encoding a proline dehydrogenase and TRP repressor gene *wrbA*. Another gene, *yeeE* is falsely annotated as a *tolB.* Likely encodes a sulphur transporter.

pal flanked by a phosphopantetheine transferase gene and peptidase N gene.

Pal structure confirmed. BLAST found no CpoB.

>AAZ19102.1 Cell division and transport-associated protein TolA [Psychrobacter arcticus 273-4]

MHKAPTVYVPTEPEGNGLTLPTLLSLLAHGLVIGILIYTYQNMDVETAGSIETTMVSPEQLAEMQG
QILANRAAAVSTMQADSGASSTATPTEAFDGSVSTSNPAQPNSQQVPVFTRSKESATSPMLMS
EEQHQRLLEQNQEYERSMAEWAARLDESVLEEHDQIEKERRQQLIEEQKTLSDFRNKQNNPPK
IKRPTATDRNIEINTGGSSSSGQTLSPSNDGQSNLSGDSTTSSPSKGSSRSASGGSRGASNSEII
NLIRRNYNPPTAAKGSTQRATLTITVNASGNITNVTASGPDEAVNEAAKQAVLNTGSLPIDADDP
KYPTFTLQFKGIN

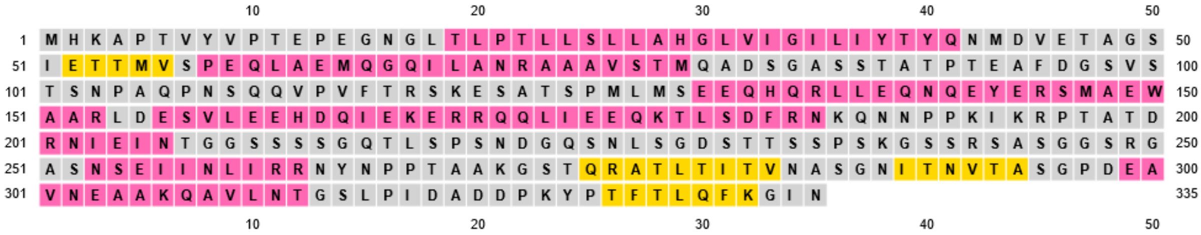

MHKAPTVYVPTEPEGNGLTLPTLLSLLAHGLVIGILIYTYQ
**NMDVETAGSIETTMVSPEQLAEMQGQILANRAAAVSTMQADSGASSTATPTEAFDGSVSTSN
PAQPNSQQVPVFTRSKESATSPMLMSEEQHQRLLEQNQEYERSMAEWAARLDESVLEEHDQ
IEKERRQQLIEEQKTLSDFRNKQNNPPKIKRPTATDRNIEINTGGSSSSGQTLSPSNDGQSNLS
GDSTTSS**
PSKGSSRSASGGSRGASNSEIINLIRRNYNPPTAAKGSTQRATLTITVNASGNITNVTASGPDEA
VNEAAKQAVLNTGSLPIDADDPKYPTFTLQFKGIN

PPIIPRED:

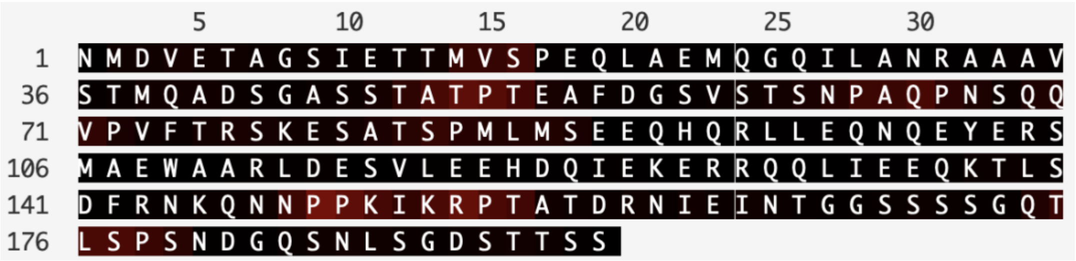

194 Domain II residues

10 Proline residues

83 α-Helix residues

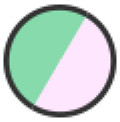

Moraxella catarrhalis

Split operon.

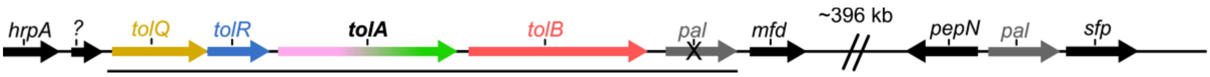

Loci: *tolQRAB* 1055899 -1067420 *pal* 1452081

tolB confirmed to have correct fold. Broken truncated *pal* next to *tolB*. Locus flanked by *hrpA* DNA-helicase followed by a short broken gene, and *mfd* encoding a transcription-repair coupling factor.

A distal *pal* flanked by aminopeptidase N gene and phosphopantetheinyl transferase gene.

>AZQ92269.1 hypothetical protein EJK53_1105 [Moraxella catarrhalis]

MRLSDNHPTVNFDKSALILPILASVLLHTVIIIAVAAPLITPPTKPNTTIQTALVGQEAFNRVKTALSN
HHANQNKPTATNTSSTITANDNAFMQAQNQHRYHPQVSTSATTTQAYHPPPNSAPFESNSPNI
QNQPTNAHAKLAEYSNHVSDLEQSNHTESTPSRAQINAAITSVKHRIEAIWQRYPKQPNQTITFQ
VNMNQQGDVTSIQFGGGHPDLRESVEAAVYAAAPFYELGGMRDSIRLQFTTEQLIMDNNQTTN
EPNH

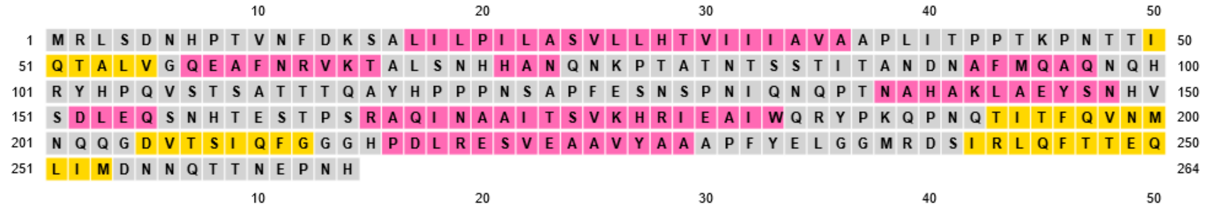

MRLSDNHPTVNFDKSALILPILA
**SVLLHTVIIIAVAAPLITPPTKPNTTIQTALVGQEAFNRVKTALSNHHANQNKPTATNTSSTITAN
DNAFMQAQNQHRYHPQVSTSATTTQAYHPPPNSAPFESNSPNIQNQPTNAHAKLAEYSNHVS
DLEQSNHTESTPS**
RAQINAAITSVKHRIEAIWQRYPKQPNQTITFQVNMNQQGDVTSIQFGGGHPDLRESVEAAVYA
AAPFYELGGMRDSIRLQFTTEQLIMDNNQTTNEPNH

PPIIPRED:

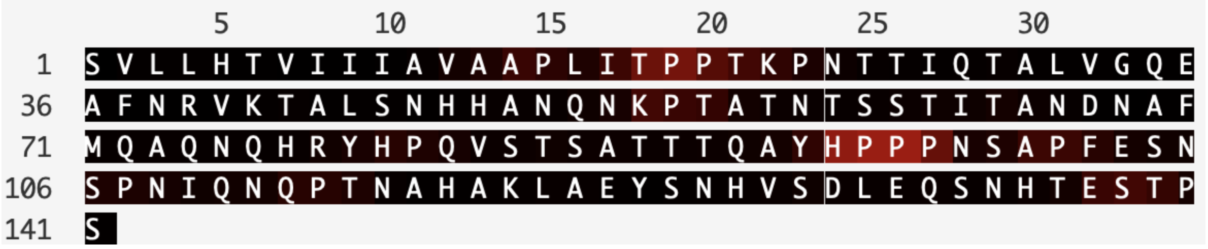

141 Domain II residues

13 Proline residues

33 α-Helix residues

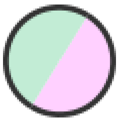

Pseudomonas aeruginosa

Locus: 1052289- 1060296

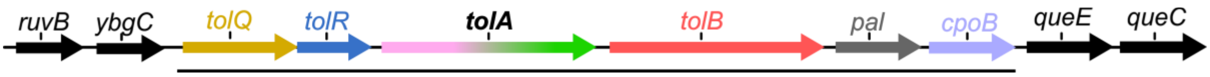

Flanked by *ruvB* and *queEC.*

>sp|P50600|TOLA_PSEAE Tol-Pal system protein TolA OS=Pseudomonas aeruginosa (strain ATCC 15692 / DSM 22644 / CIP 104116 / JCM 14847 / LMG 12228 / 1C / PRS 101 / PAO1) OX=208964 GN=tolA PE=1 SV=2

MKQQFERSPSESYFWPVVLAVVLHVLIFAMLFVSWAFAPELPPSKPIVQATLYQLKSKSQATTQT
NQKIAGEAKKTASKQYEVEQLEQKKLEQQKLEQQKLEQQQVAAAKAAEQKKADEARKAEAQKA
AEAKKADEAKKAAEAKAAEQKKQADIAKKRAEDEAKKKAAEDAKKKAAEDAKKKAAEEAKKKAA
AEAAKKKAAVEAAKKKAAAAAAAARKAAEDKKARALAELLSDTTERQQALADEVGSEVTGSLDD
LIVNLVSQQWRRPPSARNGMSVEVLIEMLPDGTITNASVSRSSGDKPFDSSAVAAVRNVGRIPE
MQQLPRATFDSLYRQRRIIFKPEDLSL

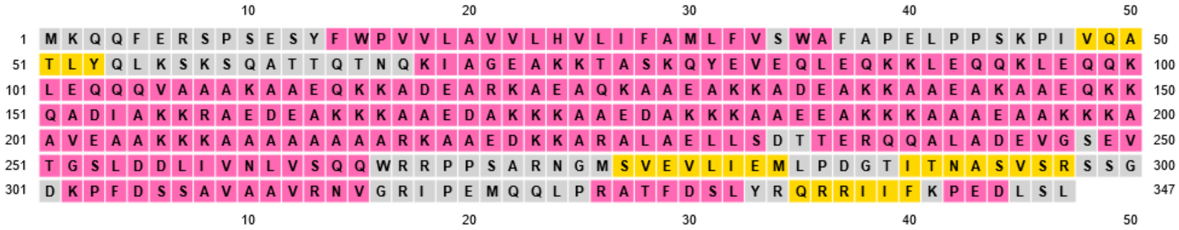

MKQQFERSPSESYFWPVVLAVVLHVLIFAMLFV
**SWAFAPELPPSKPIVQATLYQLKSKSQATTQTNQKIAGEAKKTASKQYEVEQLEQKKLEQQK
LEQQKLEQQQVAAAKAAEQKKADEARKAEAQKAAEAKKADEAKKAAEAKAAEQKKQADIA
KKRAEDEAKKKAAEDAKKKAAEDAKKKAAEEAKKKAAAEAAKKKAAVEAAKKKAAAAAAAA
ARKAAEDKKARALAELLSDTTERQQALADEVG**
SEVTGSLDDLIVNLVSQQWRRPPSARNGMSVEVLIEMLPDGTITNASVSRSSGDKPFDSSAVAA
VRNVGRIPEMQQLPRATFDSLYRQRRIIFKPEDLSL

PPIIPRED:

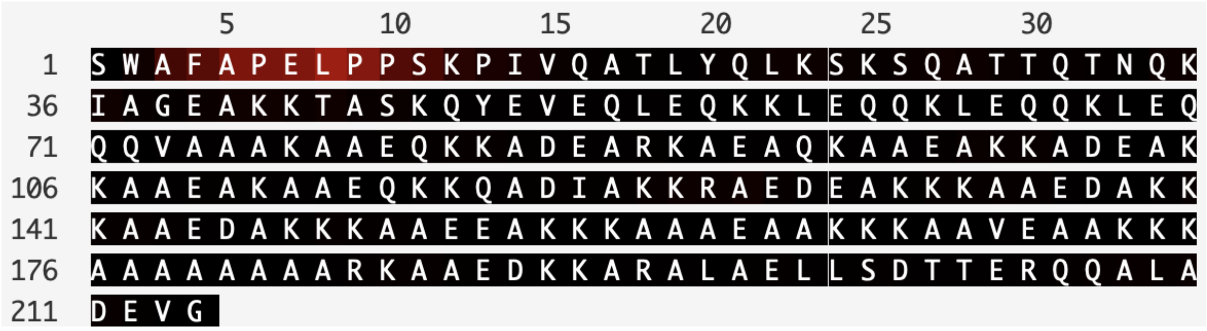

214 Domain II residues

4 Proline residues

180 α-Helix residues

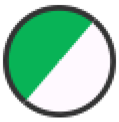

Oceanspirallales

Marinomonas mediterranea

Locus: 2155540- 2161547

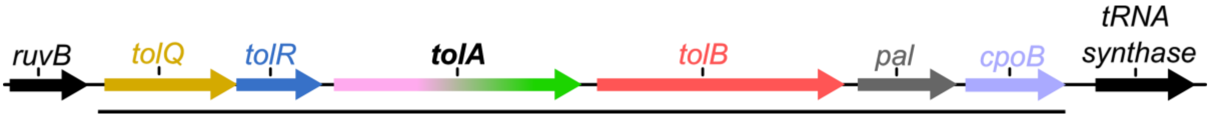

Flanked by *ruvB* and a tRNA synthase gene.

>ADZ91222.1 protein TolA [Marinomonas mediterranea MMB-1]

MKWLDKENYSLPVVFAIALHAVVLVGGAFAVNFSEEAKVAPKPAIVQATVIDISQTIIGKREAENK
AAAIQVANEAKKKADKEKARSIQKKALAEKKKQLAETKRKQEEAQKRAEQARNKELQRQKLEAQ
RKADEKKKAQAAKQKAKEEADKKARDKAKADADAKAREQEKAKEEKRKRQEVERRKQLEAEQ
KKKAEQELLARQREETAKKAAEARKKRDDQKRQQQEVQAQKDRDVAEAQAVQSLSGLINERIT
ASWIRPPSARNNMNVKLRIFFVPTGEVRDVQIMDRSGNDAFDRSAIAAVKKVERIEELSELDSYIF
ERNFRQVDLIFNPQDLRN

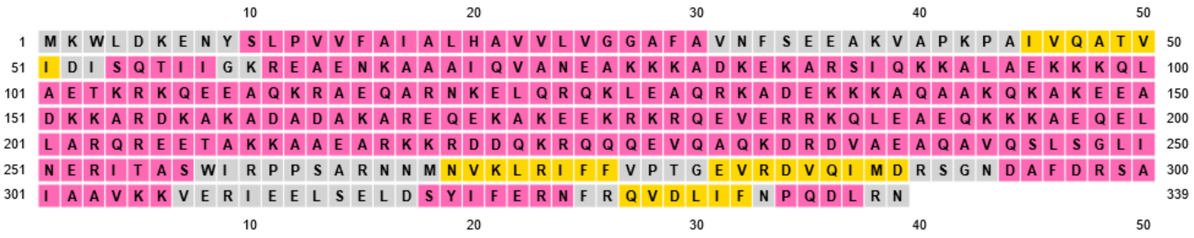

MKWLDKENYSLPVVFAIALHAVVLVGGAFA
**VNFSEEAKVAPKPAIVQATVIDISQTIIGKREAENKAAAIQVANEAKKKADKEKARSIQKKALAE
KKKQLAETKRKQEEAQKRAEQARNKELQRQKLEAQRKADEKKKAQAAKQKAKEEADKKAR
DKAKADADAKAREQEKAKEEKRKRQEVERRKQLEAEQKKKAEQELLARQREETAKKAAEA
RKKRDDQKRQQQEVQAQKDRDVAE**
AQAVQSLSGLINERITASWIRPPSARNNMNVKLRIFFVPTGEVRDVQIMDRSGNDAFDRSAIAAV
KKVERIEELSELDSYIFERNFRQVDLIFNPQDLRN

PPIIPRED:

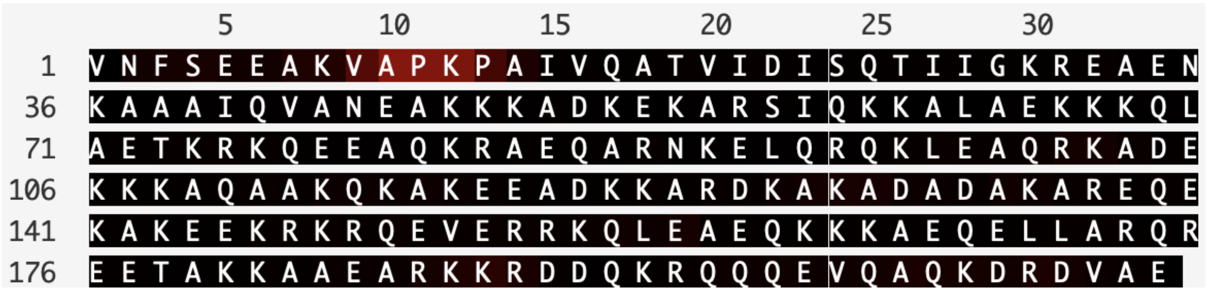

209 Domain II residues

2 Proline residues

184 α-Helix residues

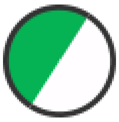

Hahella chejuensis

Locus: 5047225- 5053966

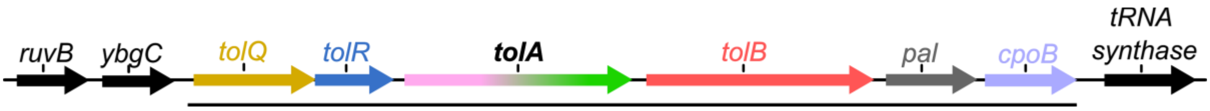

Flanked by *ruvB* and tRNA synthase genes.

>ABC31607.1 TolA family protein [Hahella chejuensis KCTC 2396]

MKDSSGNQPKSGYIGPALFSVILHVGIAGLAFLGWSAHTPPTPVIPHMQAVVIDADALKQMTKPE
PRPAVKKEEPKREEEQQKKRQEQEKQRQEELKRQEQAKQEAERKAAAEKKREQEAIALKKKQ
EEERKKKEEEKRQVEEKRKAEEKKQAEEERKKKEAERKKKEEEKRLAEQKQKELERQMKEARE
KKRQEELKKAEELKMAQEAAEYERRLQEQLEAESAAQRQQRELTEVEKYRALIYSAVTQAWLQ
PPGEIKGLKAELQLQLLPTGELLSVKVVKSSGNSAFDQSAISAANAVRKYSVPSDPGLFNREFRN
VTFIFNPK

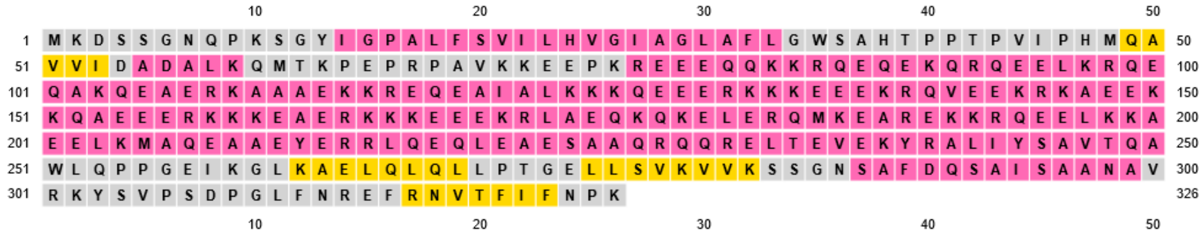

MKDSSGNQPKSGYIGPALFSVILHVGIAGLAFL
**GWSAHTPPTPVIPHMQAVVIDADALKQMTKPEPRPAVKKEEPKREEEQQKKRQEQEKQRQE
ELKRQEQAKQEAERKAAAEKKREQEAIALKKKQEEERKKKEEEKRQVEEKRKAEEKKQAEE
ERKKKEAERKKKEEEKRLAEQKQKELERQMKEAREKKRQEELKKAEELKMAQEAAEYERRL
QEQLEAESAA**
QRQQRELTEVEKYRALIYSAVTQAWLQPPGEIKGLKAELQLQLLPTGELLSVKVVKSSGNSAFD
QSAISAANAVRKYSVPSDPGLFNREFRNVTFIFNPK

PPIIPRED:

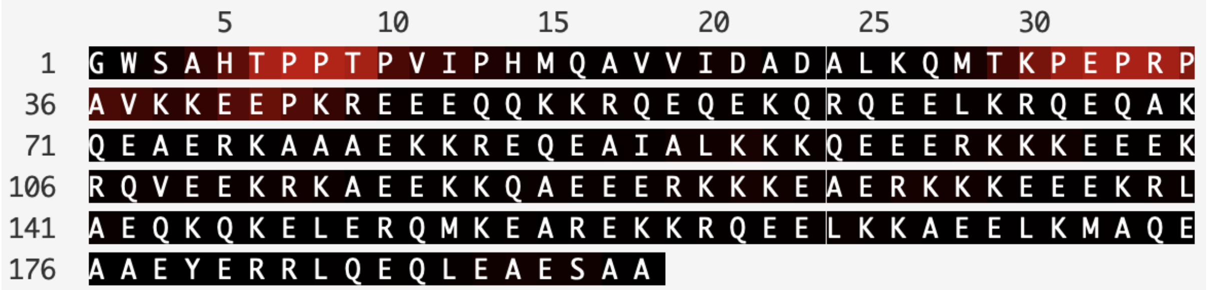

193 Domain II residues

8 Proline residues

155 α-Helix residues

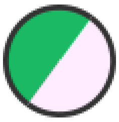

Alcanivorax dieselolei

Locus: 3597788-3591791

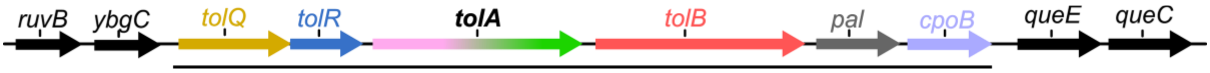

Flanked by *ruvB* and *queEC*.

>AFT71494.1 Protein TolA [Alloalcanivorax dieselolei B5]

MSDRGVGAGLSLFLHLLVLGMVLFTWTSEPELRRPPKIPPHVMAVVTDQTERAAAPTRPEPKQE
TPQPKPEPKPKPKPEPKPEPKPEPKPKPEPKPEPKPKPEPKPELKPKPEPKPEPKKPEPKPKPK
PEPPLKFEQPDMEELLAQERLEMARQGKKQDQENPGSGDPDSKVEQETASYIDAISSAVAQRW
RIPGNYRNRNDIRTRVRIRMVPGGDVVNVDVVKSSGYPDFDDSVVKGVQLASPLPVPKGDGFE
EFRTLVLEFEPGDAK

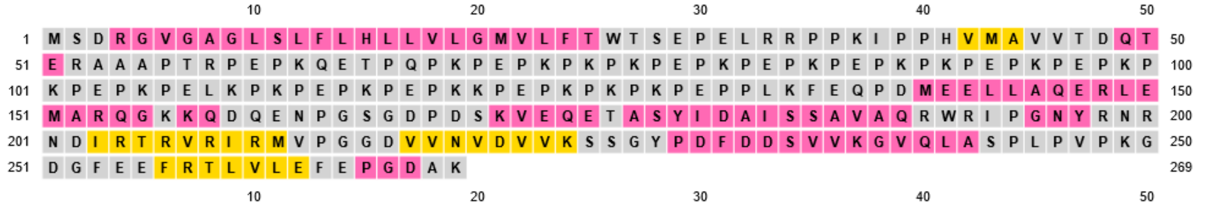

MSDRGVGAGLSLFLHLLVLGMVLFT
WTSEPELRRPPKIPPHVMAVVTDQTERAAAPTRPEPKQETPQPKPEPKPKPKPEPKPEPKPE
PKPKPEPKPEPKPKPEPKPELKPKPEPKPEPKKPEPKPKPKPEPPLKFEQPDMEELLAQERL
EMARQGKKQDQENPGSGDPD
SKVEQETASYIDAISSAVAQRWRIPGNYRNRNDIRTRVRIRMVPGGDVVNVDVVKSSGYPDFDD
SVVKGVQLASPLPVPKGDGFEEFRTLVLEFEPGDAK

PPIIPRED:

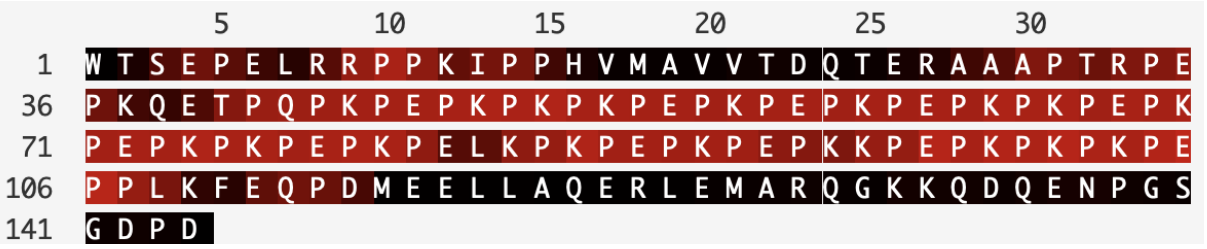

144 Domain II residues

44 Proline residues

21 α-Helix residues

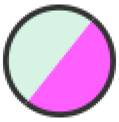

Halomonas elongata

Locus: 661747-668623

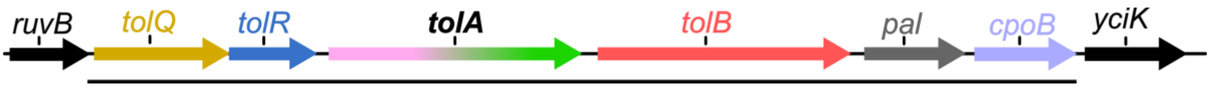

Flanked by *ruvB* and an oxidoreductase gene *yciK*.

>OBX36406.1 cell envelope integrity inner membrane protein TolA [Halomonas elongata]

MARQHRRVGYGLPTLLAVALHLGILVISVIRFPTAESEPPSQSIVQATLVSTETTTNQAQRATEPQ
ASAEADETSEDEATRQAEEEEARRQREAEEQRQREAEAEQQRQAEQERAEALEAAQAAAAEA
ERRAEEAKAEAERREQQEAERQRQAEEEAKRKAEEEAKRKAEEEAKRKAEEEAKRKAEEEAK
RKAEEEAKRKAEEEAKRKAEEEAKRKAEEEAKRKAEEEAKRKAEEEAKRKAEEEARRKAEEEA
RRKAEAAAQNSLDRAIAGEAEAAANAEQAQQAANGFINLVRQAVEQAWVIPPNVGNNANALVAI
RLGPSGEVFSASIARSSGNSAFDRAAIQAVESAAPFSELRELPASVQRDYREFNLNFRPGDIR

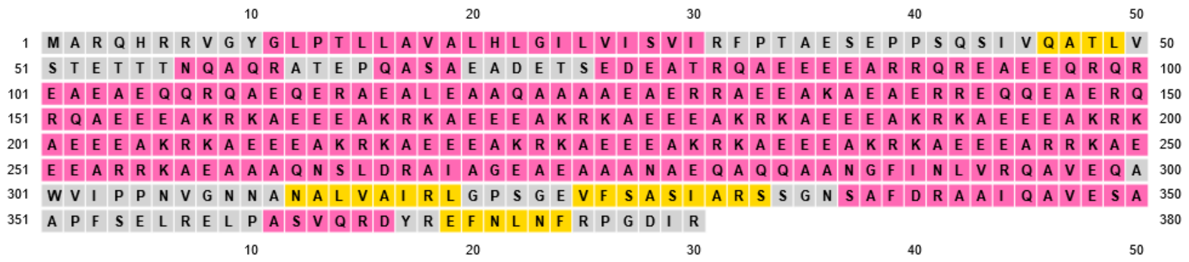

MARQHRRVGYGLPTLLAVALHLGILVISVI
**RFPTAESEPPSQSIVQATLVSTETTTNQAQRATEPQASAEADETSEDEATRQAEEEEARRQRE
AEEQRQREAEAEQQRQAEQERAEALEAAQAAAAEAERRAEEAKAEAERREQQEAERQRQA
EEEAKRKAEEEAKRKAEEEAKRKAEEEAKRKAEEEAKRKAEEEAKRKAEEEAKRKAEEEAK
RKAEEEAKRKAEEEAKRKAEEEAKRKAEEEARRKAEEEARRKAEAAAQNSLDRAIAGEAEA
AANAE**
QAQQAANGFINLVRQAVEQAWVIPPNVGNNANALVAIRLGPSGEVFSASIARSSGNSAFDRAAI
QAVESAAPFSELRELPASVQRDYREFNLNFRPGDIR

PPIIPRED:

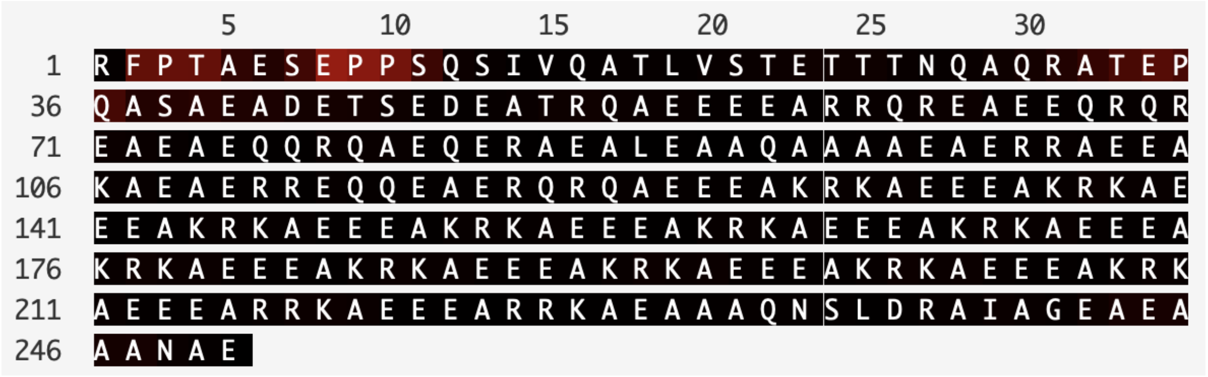

250 Domain II residues

4 Proline residues

214 α-Helix residues

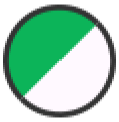

Thiotrichales

Francisella tularensis

Locus: 1262343- 1270074

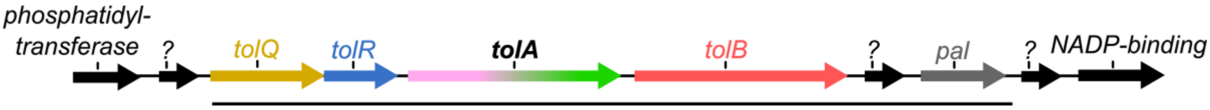

Flanked by CDP-alcohol phosphatidyltransferase gene, a lipoprotein gene, and a gene for a NADP+ binding protein.

Features a short interloping lipoprotein gene.

>ABO47107.1 protein TolA [Francisella tularensis subsp. tularensis WY96-3418]

MANLNYHKFLRFCNKQIDENPFLVKAILIHIALIILLYILSFVSSLKFEKTQASLTAQVSNMPKKFEIIQ
ATSISSSELNKQISAYENHQQELKQAKEDIKQAKLQALRKHQQQLKEKAEAEKKAKQQAILEAKK
KAQQEAQRQAEQEKQAKLEAERKAKAEAEQKAQQELQRKKEQELKAKQQAEEKARQEKLAKA
RAEAEAAARKQIEQNQAQSAISSYIAAYQDRVGANWIKDSCRGIYDLPRAIIRDGKFIKLTGTSGN
YRCDQSLIDAIKNTTPPTITNNVARKTIQTENISFIFKQS

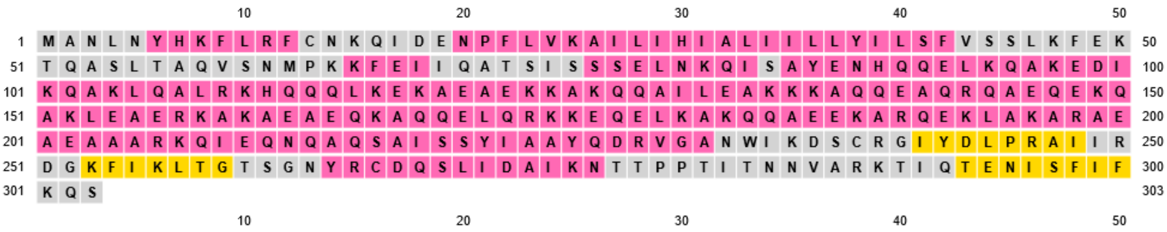

MANLNYHKFLRFCNKQIDENPFLVKAILIHIALIILLYILSF
**VSSLKFEKTQASLTAQVSNMPKKFEIIQATSISSSELNKQISAYENHQQELKQAKEDIKQAKLQ
ALRKHQQQLKEKAEAEKKAKQQAILEAKKKAQQEAQRQAEQEKQAKLEAERKAKAEAEQK
AQQELQRKKEQELKAKQQAEEKARQEKLAKARAEAEA**
AARKQIEQNQAQSAISSYIAAYQDRVGANWIKDSCRGIYDLPRAIIRDGKFIKLTGTSGNYRCDQS
LIDAIKNTTPPTITNNVARKTIQTENISFIFKQS

PPIIPRED:

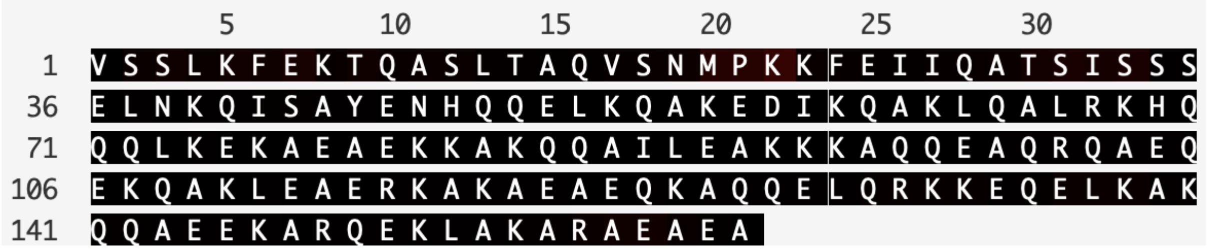

161 Domain II residues

1 Proline

131 α-Helix residues

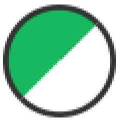

------No stator box predicted

Thiomicrospira crunogena

Locus: 973687- 981617

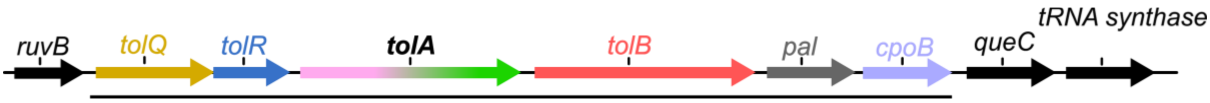

Flanked by *ruvB* and *queC* followed by a tRNA synthase gene.

>ABB41493.1 TolA protein [Hydrogenovibrio crunogenus XCL-2]

MFAFIIRHPVSMVLAIVLHSFIAIGLVYSSFQEEDVLKVKLNGETSDAEQMPVKQIQPMKTFTVDS
SLVKQQLAKIKQEEAEKIEAQKRLKRQSEAEKRHLAELKRKQLEEKKKAEAERRKALAEQRKAD
EAKRLAEIERQKVLAEQKRAQEAKRSAEKAKKEALLAEKKREEAKQLVAEAQQKRQQEEAKKKA
LEEQIQKHNAEKKRLEAEALQAKLRREQLQQEAALQRQLEEEEAKKRQAAKQKEMLSLRETYIS
SIAASVKDNWRTAAKVSEKAECVVSITQTPKGMISSVKVEKCNKFANEQFKKDAEKAVYRAEPL
PQPPIKELFERNIKFIFNP

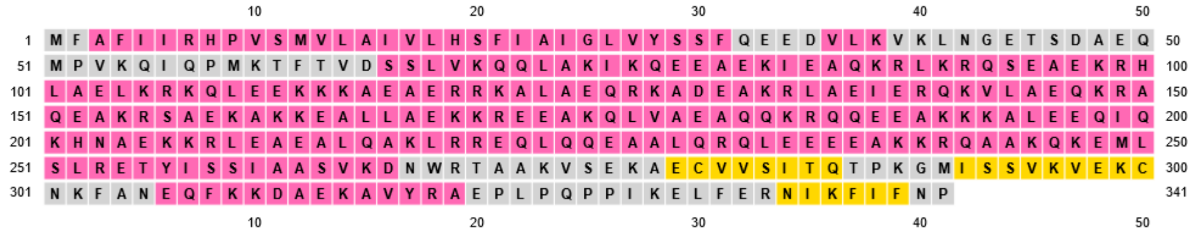

MFAFIIRHPVSMVLAIVLHSFIAIGLVYSSF
**QEEDVLKVKLNGETSDAEQMPVKQIQPMKTFTVDSSLVKQQLAKIKQEEAEKIEAQKRLKRQS
EAEKRHLAELKRKQLEEKKKAEAERRKALAEQRKADEAKRLAEIERQKVLAEQKRAQEAKR
SAEKAKKEALLAEKKREEAKQLVAEAQQKRQQEEAKKKALEEQIQKHNAEKKRLEAEALQA
KLRREQLQQEAALQRQLEEEEAKKR**
QAAKQKEMLSLRETYISSIAASVKDNWRTAAKVSEKAECVVSITQTPKGMISSVKVEKCNKFANE
QFKKDAEKAVYRAEPLPQPPIKELFERNIKFIFNP

PPIIPRED:

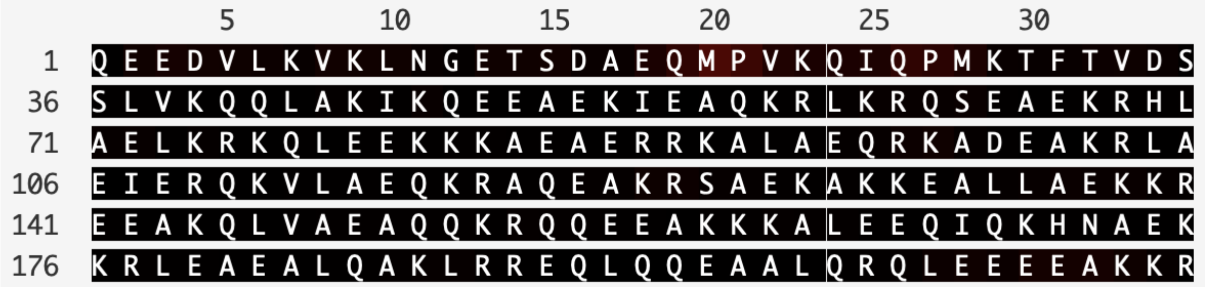

210 Domain II residues

2 Proline residues

179 α-Helix residues

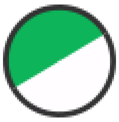

Legionellales

Legionella longbeachae

Locus: *tolQRAB* 1782229-1789629

Pal-cpoB: 2438092-2441486 **Split operon.**

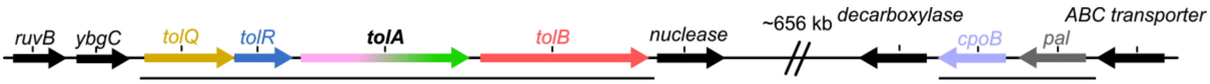

tolQRAB flanked by *ruvB* and nuclease gene and a gene for a J-domain protein.

Pal-cpoB flanked by ABC-transporter gene and diphosphomevalonate decarboxylase gene.

CpoB may be a lipoprotein (SignalP 6)

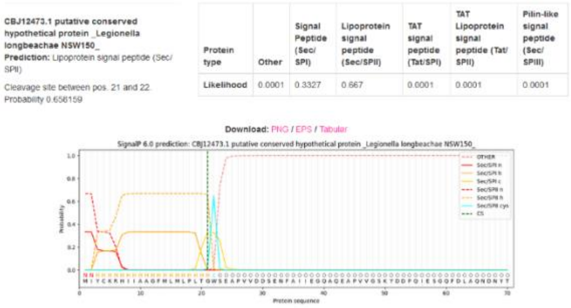

>CBJ11879.1 TolA colicin import membrane protein [Legionella longbeachae NSW150]

MISNSSYRNAFFAAIGLHLFLIVMLLTDNSSQRPVLTPETKNTPGIEQPIAVTPQNEVVKAVSVDNKQVMETVNRLKQEREQQKR
AEINRQNELKRQAEAARQQRIKEQQQLARLKEEANKIAIARKKQAEEEKKRLKQMAEQKALEAKRIEELKKQKDELVKQQKLEAQ
KLAELNKKKLAEKEKAEKIQAEMEKKKAATEAKKQAEAEAAARQAQAAQNAERQARIAGEVDKYKALIVNAIGRNWILPENVDST
LSSQFRIRLAPDGMVLEVSLTRSSGDPLLDRSAQTAIYKASPLPVPTDPDTFNLFRDISLTVRPEQVRG

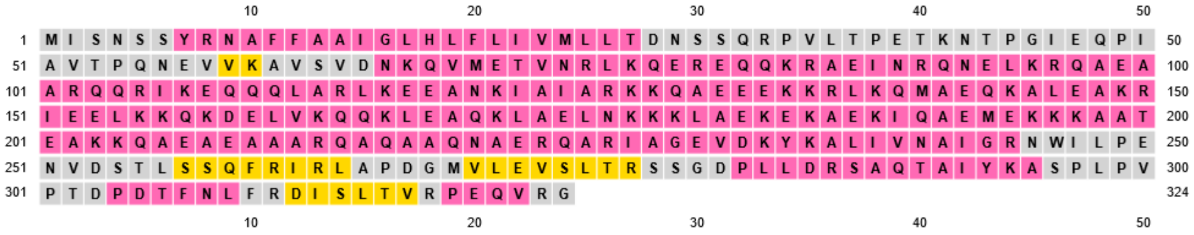

MISNSSYRNAFFAAIGLHLFLIVMLLT
DNSSQRPVLTPETKNTPGIEQPIAVTPQNEVVKAVSVDNKQVMETVNRLKQEREQQKRAEINRQNELKRQAEAARQQRIKEQ QQLARLKEEANKIAIARKKQAEEEKKRLKQMAEQKALEAKRIEELKKQKDELVKQQKLEAQKLAELNKKKLAEKEKAEKIQA EMEKKKAATEAKKQAEAEAAARQAQAAQNAERQ
ARIAGEVDKYKALIVNAIGRNWILPENVDSTLSSQFRIRLAPDGMVLEVSLTRSSGDPLLDRSAQTAIYKASPLPVPTDPDTFNLFR DISLTVRPEQVRG

PPIIPRED:

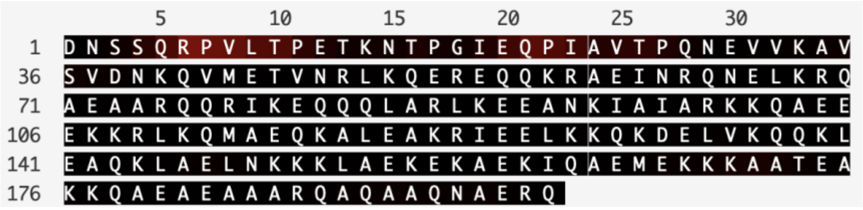

197 Domain II residues

5 Proline residues

159 α-Helix residues

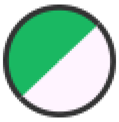

Coxiella burnetii

Locus: 78747-85952

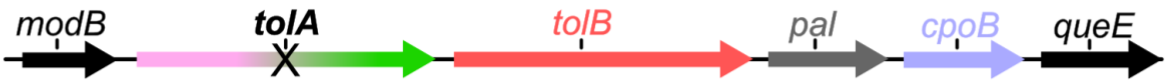

Flanked by *mdoB* phosphoglycerol transferase *queE* queuosine biosynthesis gene

- *tolA* pseudogene found with frameshift truncation

  no *TolQR* genes, broken *tolA,* intact *tolB* and *pal*

Methylococcales

Methylococcus capsulatus

Locus: 1287923-1299381

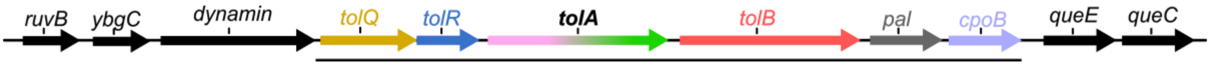

Interloping (?) dynamin GTP hydrolase gene. These are involved in membrane fission, seems functionally linked. Flanked by *ruvB* and *queEC*.

>AAU92501.1 putative TolA protein [Methylococcus capsulatus str. Bath]

MADPRLRSLRGPFALALVLHIVLLILFALNFEASQRSAETAQPDIIEAVVLDDSRIAAEAERLKTEAAPPA
ETGPEPVSTPNPEESRRAQQIEAEKAAEESRRQMEAKKLADQARKDEQRRKAEAEEKARAAAEAAA
RKKAEAEAKEKAEAEARRRAAEEARAKAAEAEAKRKAAEAARKKAEAEAKEKAEAEARRRAAEEAR
AKAAAEAEAKRKAAEAAREKAEAEAREKAAAEAAARKKAEAEAKEKAEAEARRRAAEEARAKAAAEA
EAKRRAAEAAREKAEAEAREKAAAEAAARKKAEAEAKEKAEAEARRRAAEEARARAMAEATREMEE
EVKAKAAAEARKKAVEDARRKAELEEQLNNERRELAEREANKWANRYIKPRVEGLWLKPSAARGGL
FCIIQVRTLGDGTVVEARVVKSSGDAAFDRSAESAVKKASPLPMPSDPRVAAELRSFTFKFEPAG

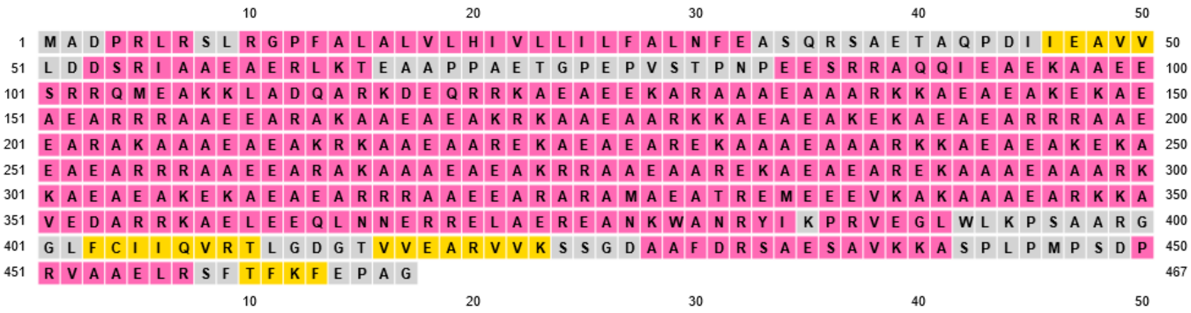

MADPRLRSLRGPFALALVLHIVLLILFALNFE
**ASQRSAETAQPDIIEAVVLDDSRIAAEAERLKTEAAPPAETGPEPVSTPNPEESRRAQQIEAEKAAE
ESRRQMEAKKLADQARKDEQRRKAEAEEKARAAAEAAARKKAEAEAKEKAEAEARRRAAEEAR
AKAAEAEAKRKAAEAARKKAEAEAKEKAEAEARRRAAEEARAKAAAEAEAKRKAAEAAREKAE
AEAREKAAAEAAARKKAEAEAKEKAEAEARRRAAEEARAKAAAEAEAKRRAAEAAREKAEAEA
REKAAAEAAARKKAEAEAKEKAEAEARRRAAEEARARAMAEATREMEEEVKAKAAAEARKKAV
EDARRKAELEEQLNNE**
RRELAEREANKWANRYIKPRVEGLWLKPSAARGGLFCIIQVRTLGDGTVVEARVVKSSGDAAFDRSA
ESAVKKASPLPMPSDPRVAAELRSFTFKFEPAG

PPIIPRED:

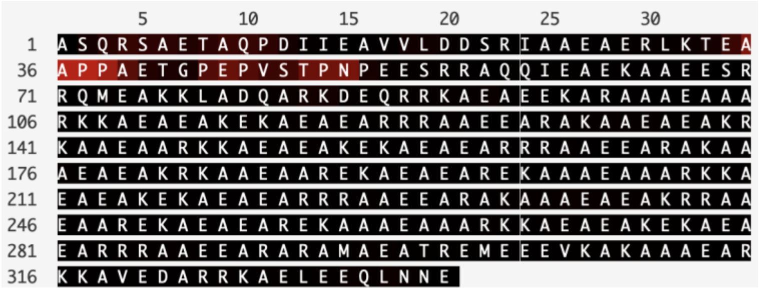

335 Domain II residues

7 Proline residues

297 α-Helix residues

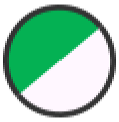

Chromatiales

Nitrosococcus oceani

Locus: 152065-159898

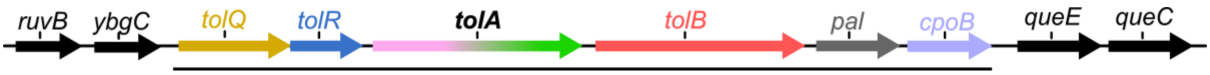

Flanked by *ruvB* and *queEC*.

>ABA56677.1 Cell division and transport-associated protein TolA [Nitrosococcus oceani ATCC 19707]

MENPRQKWFAFALALLVHGILFAVLVISLGWTPKPEPVNIAPAQIVQATVVDKEKLLAEAAQRRQ
AEQDALARQQALVQKREAAERQRAEAQRQQQARAEQARRETQEQQALEQKQQEEQARLKRL
EAERQAKEEAARRQAEAEKKRAEEKKRQAEAEKRRLEEERRRAEAAKRKAEEERKKIEAAKRK
AEEERKKAEAARRQQELQDRIEAEENAAQVEAARNRFLTQVKMRVQRYWIRPPSARDDLVVLL
QVEMLPSGEVRNVKVAKSSGDRAFDRSGEAAVYKAAPLPIPAESAAAEDFLPRFNFKFCSDPSN
CK

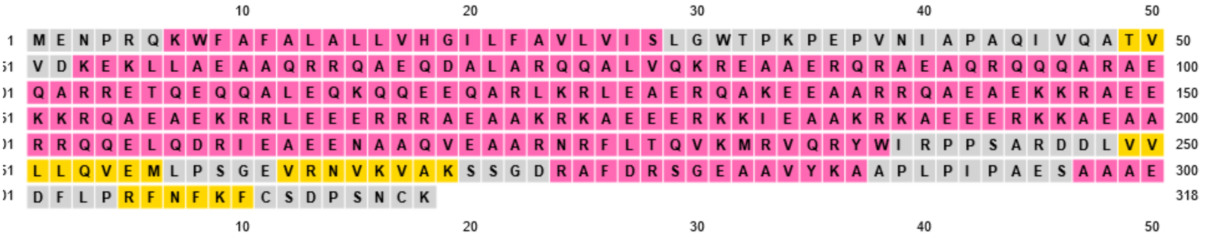

MENPRQKWFAFALALLVHGILFAVLVIS
**LGWTPKPEPVNIAPAQIVQATVVDKEKLLAEAAQRRQAEQDALARQQALVQKREAAERQRA
EAQRQQQARAEQARRETQEQQALEQKQQEEQARLKRLEAERQAKEEAARRQAEAEKKRAE
EKKRQAEAEKRRLEEERRRAEAAKRKAEEERKKIEAAKRKAEEERKKAEAARRQQELQDRIE
AEENAAQ**
VEAARNRFLTQVKMRVQRYWIRPPSARDDLVVLLQVEMLPSGEVRNVKVAKSSGDRAFDRSG
EAAVYKAAPLPIPAESAAAEDFLPRFNFKFCSDPSNCK

PPIIPRED:

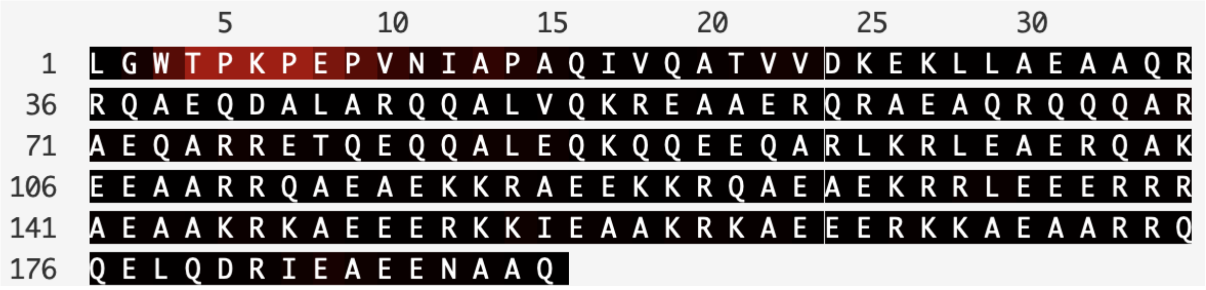

190 Domain II residues

4 Proline residues

166 α-Helix residues

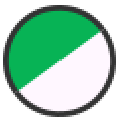

Thioalkalivibrio nitratireducens

Locus: 3482898-3490964

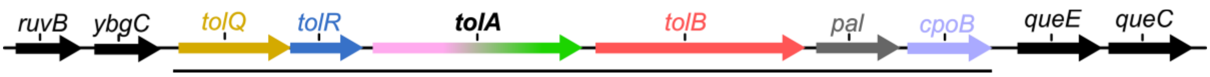

Flanked by *ruvB* and *queEC*.

>AGA35006.1 Translation initiation factor 2 [Thioalkalivibrio nitratireducens DSM 14787]

MFSLIRQHFGQLLLVLLLHAVLFAALLLNVSFLQPSASSALRFSPDPAEVIEAVAMDVETFERIERE
IRTAEQERIAAELQAEEEARREAERLRQEELRRQREEQERREAEERARQEALRAAEEAREEAR
RVAEEAQREAQRVAEEARRMAEAERARIEQERREAEAERARAEQERRAAEEARRAAEEARRA
EEAERREREAAAQREREAAERRRAEEEARRREAQARELEEQRRREQMEQERQRLAAARAQR
EAADRRAQQDALRDAYIADVQGTVERRWRRPEGLRSGDEAFVLVRVNPDTGRILSFQVQSCSG
APAFCESVRQTMERLQSLPRPPDAAVVQGGIRIRFAPDQG

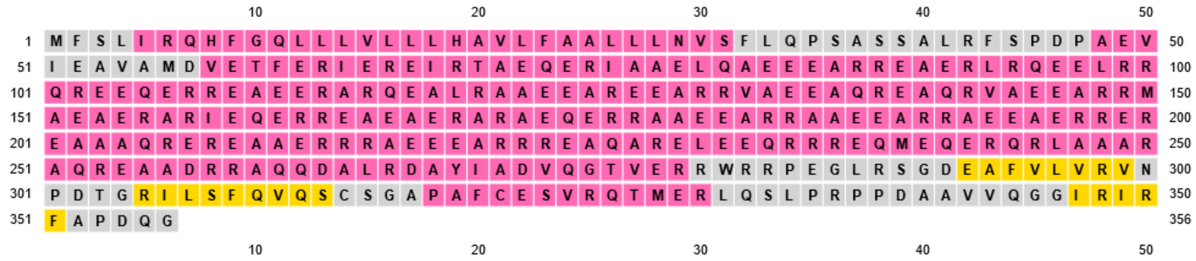

MFSLIRQHFGQLLLVLLLHAVLFAALLLNVS
**FLQPSASSALRFSPDPAEVIEAVAMDVETFERIEREIRTAEQERIAAELQAEEEARREAERLRQ
EELRRQREEQERREAEERARQEALRAAEEAREEARRVAEEAQREAQRVAEEARRMAEAER
ARIEQERREAEAERARAEQERRAAEEARRAAEEARRAEEAERREREAAAQREREAAERRRA
EEEARRREAQARELEEQRRREQMEQERQRLAAARAQREAA**
DRRAQQDALRDAYIADVQGTVERRWRRPEGLRSGDEAFVLVRVNPDTGRILSFQVQSCSGAPA
FCESVRQTMERLQSLPRPPDAAVVQGGIRIRFAPDQG

PPIIPRED:

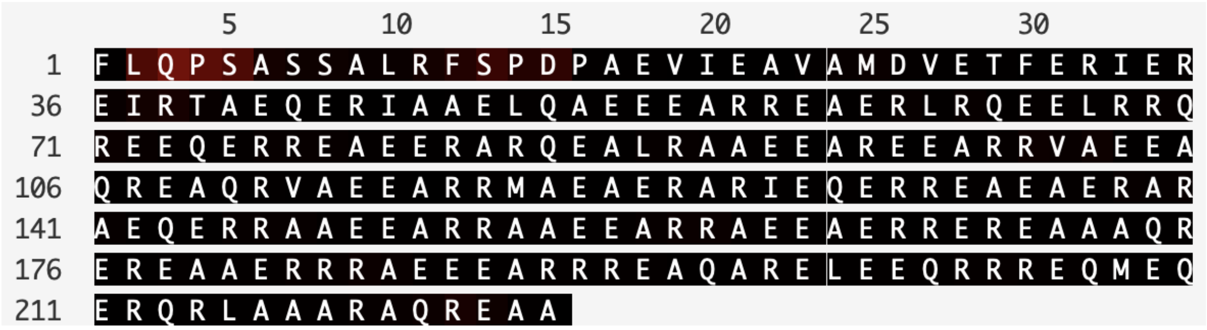

225 Domain II residues

3 Proline residues

202 α-Helix residues

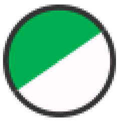

Halothiobacillus neapolitanus

Locus: 565881-573295

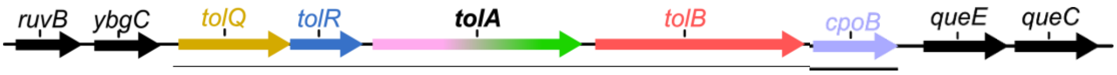

Flanked by *ruvB* and *queEC.*

Two distant “*pal”* genes one and two. First one has a resident fusion domainm, second one seems too short.

>ACX95389.1 protein TolA [Halothiobacillus neapolitanus c2]

MFRWFTRMPLSILLAIALHLLIILSVLIAWKFNAFSAASQSSSTSNEPVIQAQSVSQAAIDQQVNRLKQADQQREKS
LKQQQTEAQQAAAARRAEQARLQQLEAMREAKQKAAAAQEQQLKALQDEQKKAQESVQAQKQALAKMQEEAA
KAAAEKQAAQAAAAKERATAAAAKQAAAEAAAQAEQAKKQAAAEKAAAEKAAKEQAAKEKAAKAAADKAAKEK
AANLAAQKAAQARKAALQQQLQDELSASQAQGILAAYAAAIQQKVTAQWFKPPGWQPDWTCDVRISQAKDGTV
LNVKILQCDGDQLFQRSVQQAVERASPLPLPSDMSLFQSTINFKFRANTQ

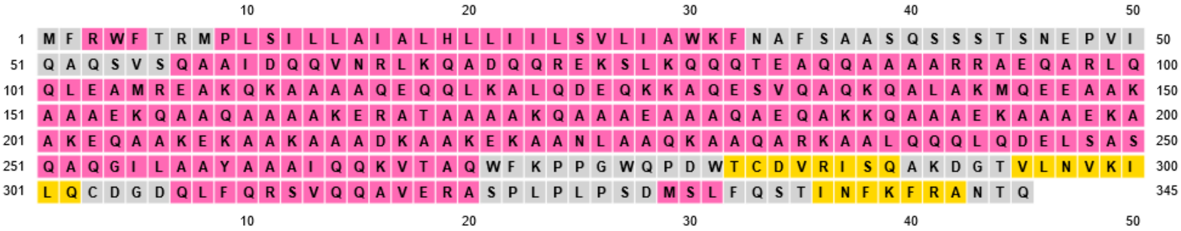

MFRWFTRMPLSILLAIALHLLIILSVLIAWKF
NAFSAASQSSSTSNEPVIQAQSVSQAAIDQQVNRLKQADQQREKSLKQQQTEAQQAAAARRAEQARLQQLE AMREAKQKAAAAQEQQLKALQDEQKKAQESVQAQKQALAKMQEEAAKAAAEKQAAQAAAAKERATAAAA KQAAAEAAAQAEQAKKQAAAEKAAAEKAAKEQAAKEKAAKAAADKAAKEKAANLAAQKAAQARKAALQQ QLQD
ELSASQAQGILAAYAAAIQQKVTAQWFKPPGWQPDWTCDVRISQAKDGTVLNVKILQCDGDQLFQRSVQQAVE
RASPLPLPSDMSLFQSTINFKFRANTQ

PPIIPRED:

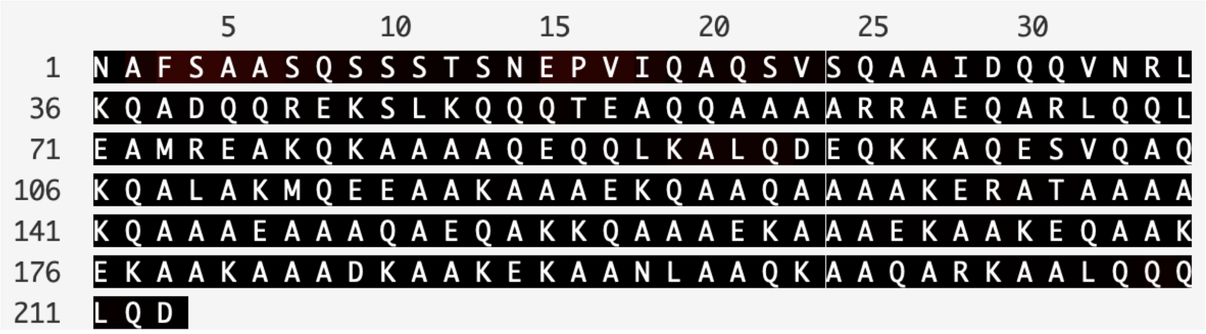

213 Domain II residues

1 Proline

189 α-Helix residues

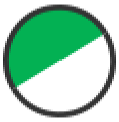

Cardiobacteriales

Dichelobacter nodosus

Locus: 1215097- 1223422

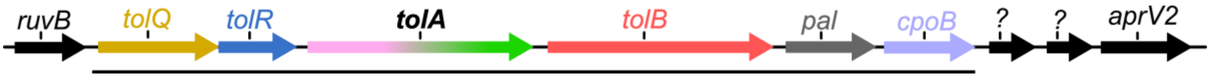

Flanked by *ruvB* and 2x short genes followed by a subtilisin-like gene encoding a peptidase AprV2. *cpoB* annotated *bamD* (model), TPR repeats confirmed. Structure of this CpoB is shorter, and is likely a lipoprotein:

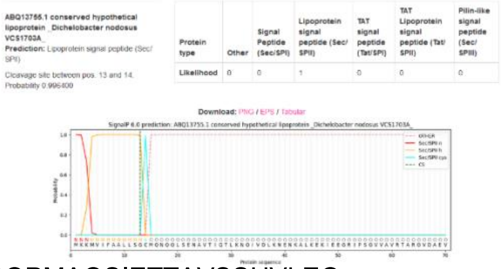

>ABQ13251.1 TolA protein [Dichelobacter nodosus VCS1703A]

MIERLSKRWEDIFSIVAVGIFYAVLGVGIFLNYQWAKSQNATFVAPRAGQPMAQSIETTAVSSHVLEQ
RLEAIAAKKEAIEQARLAKIRAEEEEKRLAAERERLAKEKAEQERRARLEAERLAKERAEQEEKAAAE
QAAKEKAEKAAAEKEKAAKAAAAKKKAEKAAAAKEKAAKAAAAKKKAEKAAAAKEKAAKAAAAKKKA
EKAAAAKEKAAKAAAAKEKAAKAATAKKKAATAKKKAAAAKEKAAKAAAAKEKAAKAKAAREARELE
AKLAALGAPEGLGNGLASFDEQAFAAAKSEAVELFGDAVKARMKRFWQLPPNIPTNLSAQLLLRIDQ
HGKVVHVEVNRSSGYPLFDTAAMSAAKAASPLPLPNFDDLTAELVRDGVLINFSP

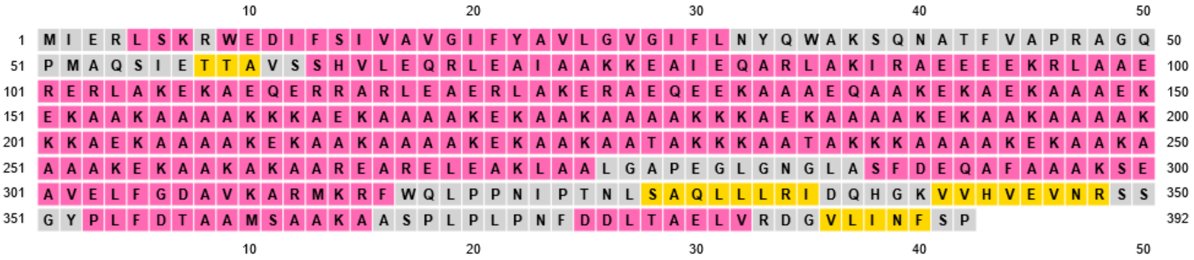

MIERLSKRWEDIFSIVAVGIFYAVLGVGIFL
**NYQWAKSQNATFVAPRAGQPMAQSIETTAVSSHVLEQRLEAIAAKKEAIEQARLAKIRAEEEEKRL
AAERERLAKEKAEQERRARLEAERLAKERAEQEEKAAAEQAAKEKAEKAAAEKEKAAKAAAAK
KKAEKAAAAKEKAAKAAAAKKKAEKAAAAKEKAAKAAAAKKKAEKAAAAKEKAAKAAAAKEKA
AKAATAKKKAATAKKKAAAAKEKAAKAAAAKEKAAKAKAAREARELEAKLAALGAPEGLGNGL
ASFDEQ**
AFAAAKSEAVELFGDAVKARMKRFWQLPPNIPTNLSAQLLLRIDQHGKVVHVEVNRSSGYPLFDTAA
MSAAKAASPLPLPNFDDLTAELVRDGVLINFSP

PPIIPRED:

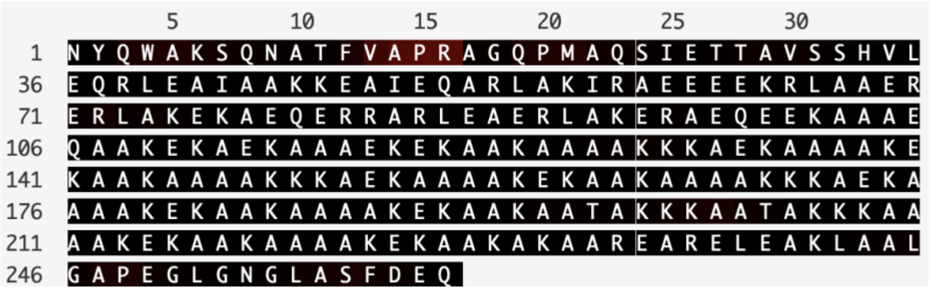

261 Domain II residues

3 Proline residues

218 α-Helix residues

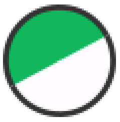

Xanthomonadales

Pseudoxanthomonas spadix

Locus: 843922- 851740

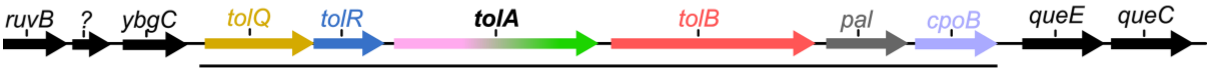

Flanked by *ruvB*, a short gene, and *queEC*.

>AER55631.1 protein TolA [Pseudoxanthomonas spadix BD-a59]

MHAEASSFDRRDPRDRPGTAVVLAVSLHLLLLLLLIVLSPYFNWNTDQASAAAGAPAIEASLEMSA
SDMRAAQQAIREASKVEPKPEPPTPMPTPQPVAEQDTVPPPQPLPEPRPQDSPVPQQQQAQE
PVPDPDQVNQEQASRLAIAQEKAEREQEAKRRQEQIDLTERKRQEEAERKQRLAEQQLEDDRQ
KKLAAIRAQREQAARQARLAEQKLRQLADARAKQASAQAATAAPSAPPGQNGVDEGLLARYAA
AIQSAVAQQWTRPDSVSVGTRCVVEITQLPGGEVMEAQVRPGCPMDAAGQRSIEAAVLKAQPL
PYRGFEPVFRRKLTFIFEAQDR

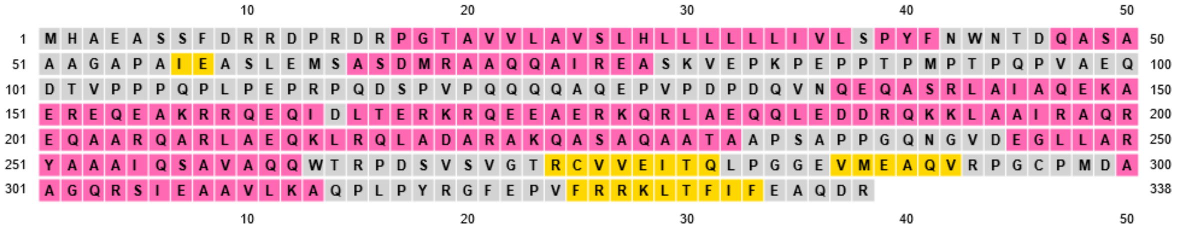

MHAEASSFDRRDPRDRPGTAVVLAVSLHLLLLLLLIVL
**SPYFNWNTDQASAAAGAPAIEASLEMSASDMRAAQQAIREASKVEPKPEPPTPMPTPQPVAE
QDTVPPPQPLPEPRPQDSPVPQQQQAQEPVPDPDQVNQEQASRLAIAQEKAEREQEAKRRQ
EQIDLTERKRQEEAERKQRLAEQQLEDDRQKKLAAIRAQREQAARQARLAEQKLRQLADAR
AKQASAQAATAAPSAPP**
GQNGVDEGLLARYAAAIQSAVAQQWTRPDSVSVGTRCVVEITQLPGGEVMEAQVRPGCPMDA
AGQRSIEAAVLKAQPLPYRGFEPVFRRKLTFIFEAQDR

PPIIPRED:

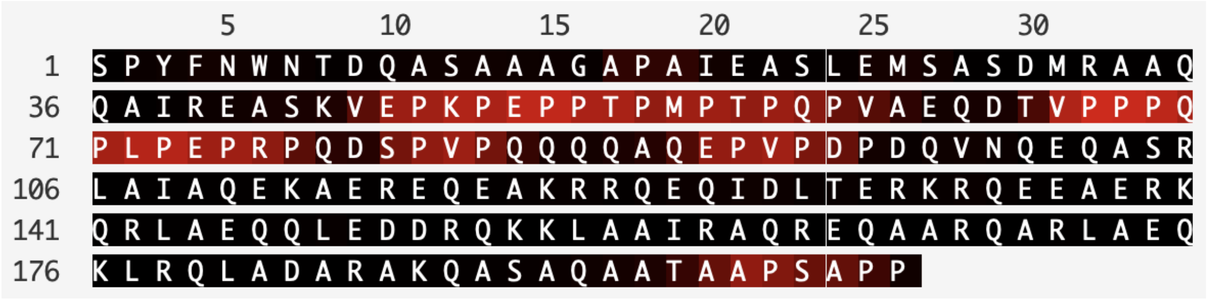

201 Domain II residues

25 Proline residues

116 α-Helix residues

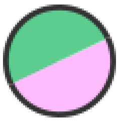

Xanthomonas axonopodis

Locus: 3689159- 3697810

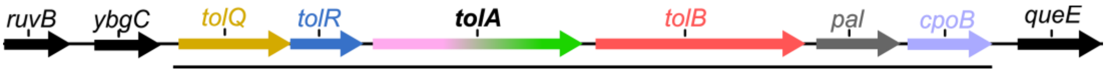

Flanked by *ruvB* and *queEC.*

>AAM37987.1 TolA protein [Xanthomonas citri pv. citri str. 306]

MHADALPTQAARDDGWFRPVVLALVVHVLVALVFIAGWLWSPERSVEPAAGDPSMEASLDVSA
AEARVARQALKATPVETPPPPAPLPEPAPEDSVPPPQPIPEPRPQDAPTPQQAQAQERVAQPD
KVDQERVDALAISAEKAKQEQEAKRRQEQIDLTERKRQEEAEQKLRLAKQQEEADAKKKQAAA
QQAAEEAERQKKIADIRRQRAQADKEMALAEQKLRQVAAARAQQASAAAATSAQPTAGQGGT
STDLSAKYAAAIQQKVLAQWVRPPSVPPGQKCTINIRQLPGGSVMEAKVAPGCPYDEAGQRSIE
AAVLSAQPLPYRGFESVFQRNLTFVFTAQDQ

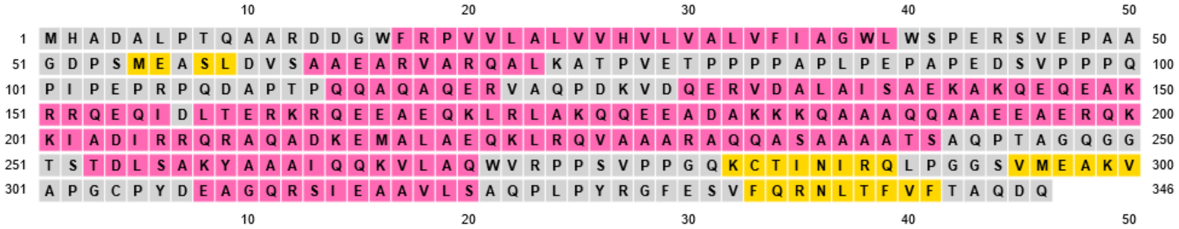

MHADALPTQAARDDGWFRPVVLALVVHVLVALVFIAGWL
WSPERSVEPAAGDPSMEASLDVSAAEARVARQALKATPVETPPPPAPLPEPAPEDSVPPPQP IPEPRPQDAPTPQQAQAQERVAQPDKVDQERVDALAISAEKAKQEQEAKRRQEQIDLTERKR QEEAEQKLRLAKQQEEADAKKKQAAAQQAAEEAERQKKIADIRRQRAQADKEMALAEQKLR QVAAARAQQASAAAATSAQPTA
GQGGTSTDLSAKYAAAIQQKVLAQWVRPPSVPPGQKCTINIRQLPGGSVMEAKVAPGCPYDEA
GQRSIEAAVLSAQPLPYRGFESVFQRNLTFVFTAQDQ

PPIIPRED:

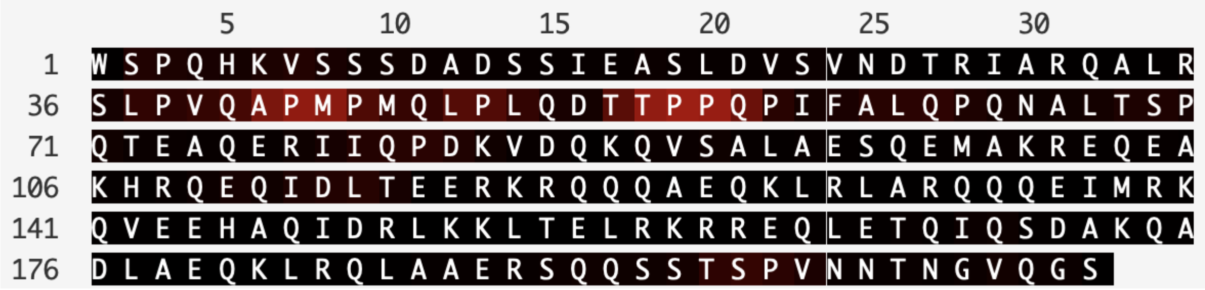

207 Domain II residues

23 Proline residues

130 α-Helix residues

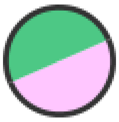

Xylella fastidiosa

Locus: 1149471-1157166

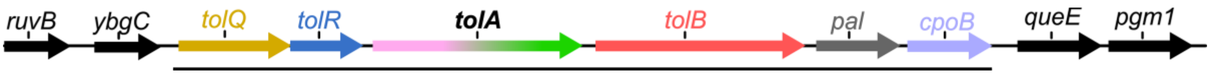

Flanked by *ruvB* and *queE* and *pgm1*, a phosphoglycerate mutase.

TolB may be a lipoprotein.

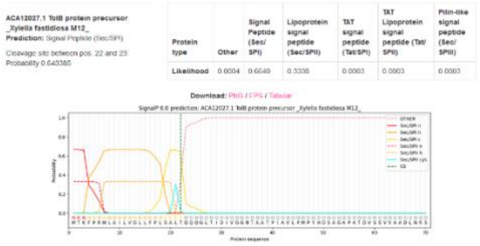

>ACA12026.1 TolA protein [Xylella fastidiosa M12]

MHADSRLGHSRRREGIVLPVLMALLLHGFIGGVFLLSWLWSPQHKVSSSDADSSIEASLDVSVN
DTRIARQALRSLPVQAPMPMQLPLQDTTPPQPIFALQPQNALTSPQTEAQERIIQPDKVDQKQVS
ALAESQEMAKREQEAKHRQEQIDLTEERKRQQQAEQKLRLARQQQEIMRKQVEEHAQIDRLKK
LTELRKRREQLETQIQSDAKQADLAEQKLRQLAAERSQQSSTSPVNNTNGVQGSGKNVDNKGL
RDKYKAAIQQAVSGQWSRPPSVPLGQECMIHITQLPGGRVLSAEVAPDCPYDDAGRRSIESAV
MRAQPLPYRGFEPVFERDVYFKFIPEDH

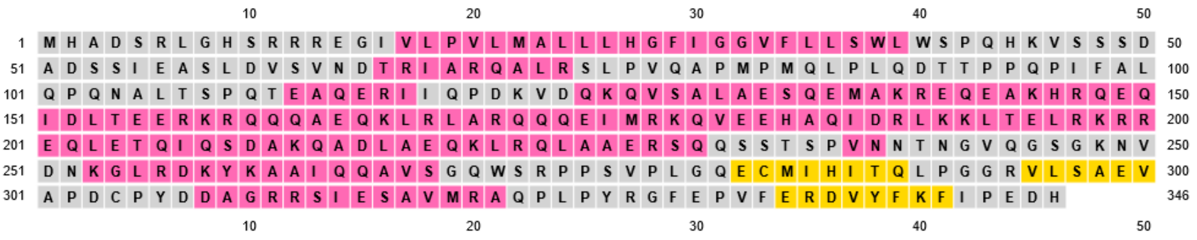

MHADSRLGHSRRREGIVLPVLMALLLHGFIGGVFLLSWL
**WSPQHKVSSSDADSSIEASLDVSVNDTRIARQALRSLPVQAPMPMQLPLQDTTPPQPIFALQP
QNALTSPQTEAQERIIQPDKVDQKQVSALAESQEMAKREQEAKHRQEQIDLTEERKRQQQAE
QKLRLARQQQEIMRKQVEEHAQIDRLKKLTELRKRREQLETQIQSDAKQADLAEQKLRQLAA
ERSQQSSTSPVNNTNGVQGS**
GKNVDNKGLRDKYKAAIQQAVSGQWSRPPSVPLGQECMIHITQLPGGRVLSAEVAPDCPYDDA
GRRSIESAVMRAQPLPYRGFEPVFERDVYFKFIPEDH

PPIIPRED:

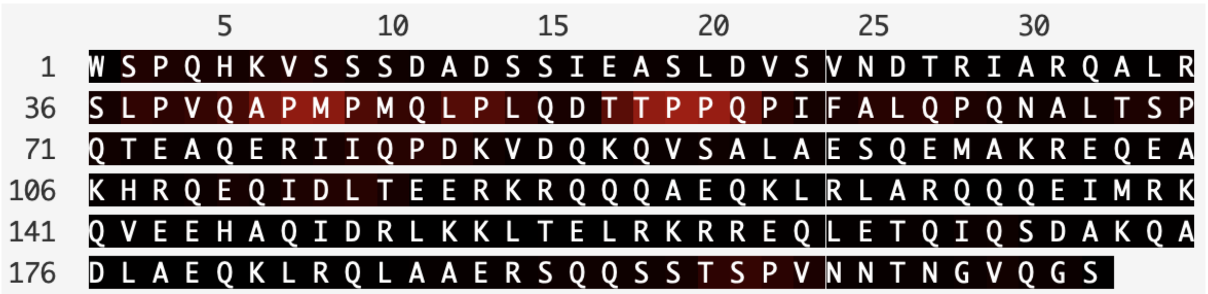

207 Domain II Residues

12 Proline residues

123 α-Helix residues

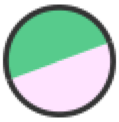

β-Proteobacteria

Neisseriales

Chromobacterium violaceum

Split operon. *tolQ* seems to have been separated.

Locus: 119471- 125106

tolQ locus: 588718

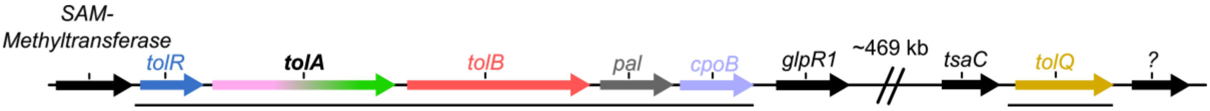

tolRAB-pal-cpoB flanked by an O-methyltransferase gene and *glpR1* transcriptional repressor protein.

tolQ gene is distal, annotated as "*tolQ*". Flanked by *tsaC* and a probable transmembrane protein gene.

>AAQ57787.1 conserved hypothetical protein [Chromobacterium violaceum ATCC 12472]

MKPQSSASPRPWTFIASALFHAGVIGLLLWGGLQTTPPPSPAPLALELWTTAPPPPPAVTAPVAPAPA
PQPAPEPKPVVETPPAEVNLGKKDAPKPKHETPKPEPKPAAEPEPKKPKHAPKPAEPEKKVPEKAKP
AEKAPDRKEAKAQTPPAAKPGHKTAKAYNNEADDLLSSLDSARTSGKPNARSDQAGSKHGVAGGAV
NGSGVSAGWIDLVKAKVTPLVQIPPGIAGNPAAVVRVTLLPTLEVRAVQLVKSSGVPAYDDAVQRAV
WEAKTFPKLPAGANFNDGYRTFTLTFRPR

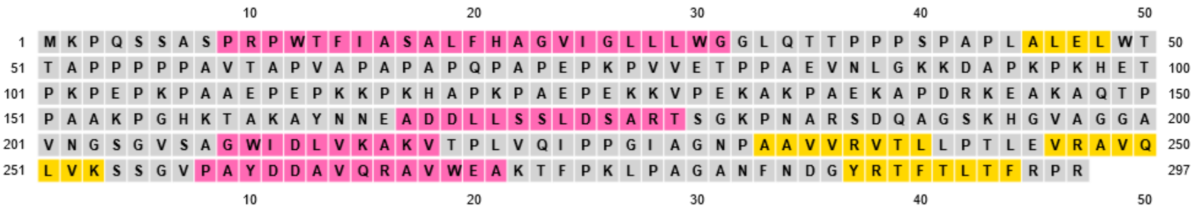

MKPQSSASPRPWTFIASALFHAGVIGLLLWG
**GLQTTPPPSPAPLALELWTTAPPPPPAVTAPVAPAPAPQPAPEPKPVVETPPAEVNLGKKDAPKPK
HETPKPEPKPAAEPEPKKPKHAPKPAEPEKKVPEKAKPAEKAPDRKEAKAQTPPAAKPGHKTAK
AYNNEADDLLSSLDSARTSGKPNARSDQAGSKHGVA**
GGAVNGSGVSAGWIDLVKAKVTPLVQIPPGIAGNPAAVVRVTLLPTLEVRAVQLVKSSGVPAYDDAV
QRAVWEAKTFPKLPAGANFNDGYRTFTLTFRPR

PPIIPRED:

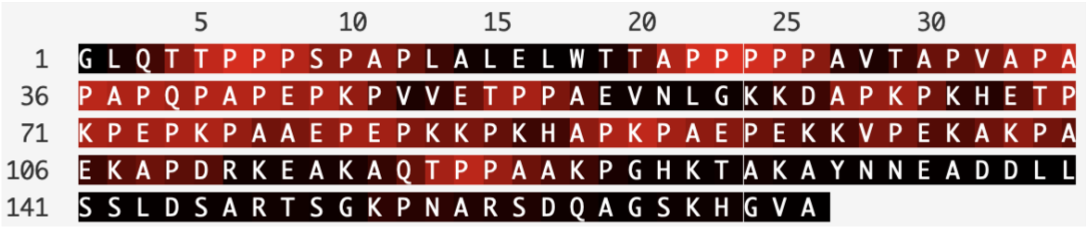

166 Domain II residues

39 Proline residues

13 α-Helix residues

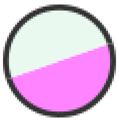

Neisseria meningitidis

---No *tolA* or *tolB* or *pal* detected

Burkholderiales

Collimonas fungivorans

Locus: 4403030- 4411544

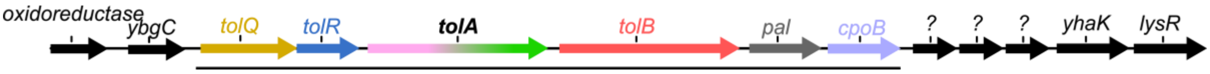

Flanked by a membrane-bound NADP-dependent oxidoreductase gene and three short genes followed by a redox-sensitive bicupin gene *yhaK* and *lysR*.

>AMO96805.1 protein TolA [Collimonas fungivorans]

MLAAAVHAALFAFLWFGIHWQSTQPIAVEAEIWDPQIKDAAPTPPEPVPQPTPEPKPEPVIQDTT
PPKVVAPPLPDPEIALEQEKKRKEQERKDLQRQEELEKQRKLQKQQEEQEEKDRLKAEAEQKA
AKDKAAAQEKQKLDAAKKTEADAKKKQAATEAAAETKRRSDEMKRMLGQAVGNGPANSTGTA
AQSQGPRGDPGYAQKVASKIKSNIIFNVPEGLAGNPAAEFDVQLLPDGSVASVRLRKSSGVSGF
DEAVKRAIEKSAPYPKDKTGSVPSSFIGTHKPKDQ

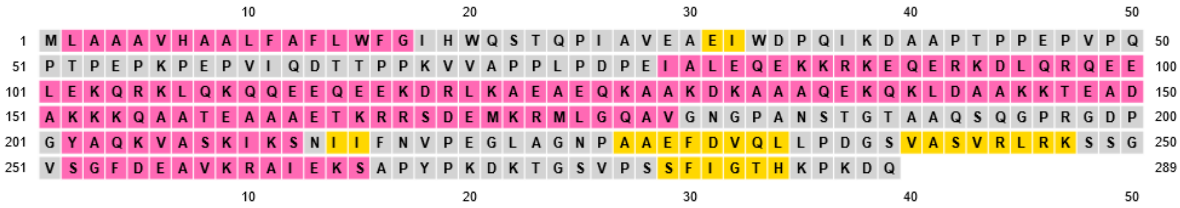

MLAAAVHAALFAFLWFG
IHWQSTQPIAVEAEIWDPQIKDAAPTPPEPVPQPTPEPKPEPVIQDTTPPKVVAPPLPDPEIALE
QEKKRKEQERKDLQRQEELEKQRKLQKQQEEQEEKDRLKAEAEQKAAKDKAAAQEKQKLD
AAKKTEADAKKKQAATEAAAETKRRSDEMKRMLGQAVGNGPANSTGT
AAQSQGPRGDPGYAQKVASKIKSNIIFNVPEGLAGNPAAEFDVQLLPDGSVASVRLRKSSGVSG
FDEAVKRAIEKSAPYPKDKTGSVPSSFIGTHKPKDQ

PPIIPRED:

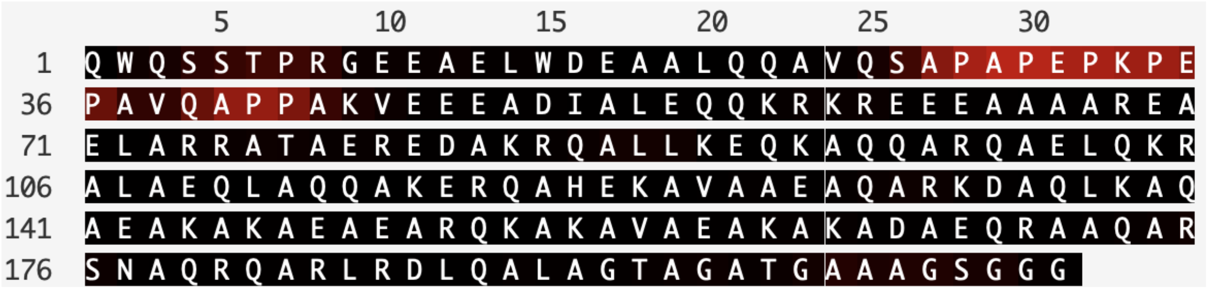

172 Domain II residues

19 Proline residues

101 α-Helix residues

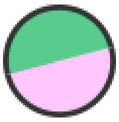

Ralstonia solanacearum

Locus: 778153- 785194

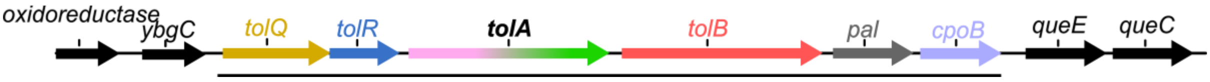

Flanked by a membrane-bound NADP-dependent oxidoreductase gene and *queEC*.

CpoB tagged as transmembrane?

>CAD14264.1 probable tola-related transport transmembrane protein [Ralstonia pseudosolanacearum GMI1000]

MATTSLHRYEPVRERGTLRAFALAVLTHLLLLTFLYFGVQWQSSTPRGEEAELWDEAALQQAV
QSAPAPEPKPEPAVQAPPAKVEEEADIALEQQKRKREEEAAAAREAELARRATAEREDAKRQAL
LKEQKAQQARQAELQKRALAEQLAQQAKERQAHEKAVAAEAQARKDAQLKAQAEAKAKAEAE
ARQKAKAVAEAKAKADAEQRAAQARSNAQRQARLRDLQALAGTAGATGAAAGSGGGVGSGT
GAGGTASAGYADRVRAKVQPNINFYDDTSGNPIVTVLVQMAPDGTVLSRRVTKPSGYPVLDEA
VLRAIDLSSPLPRDTNGKAPASVVVGFKPKRG

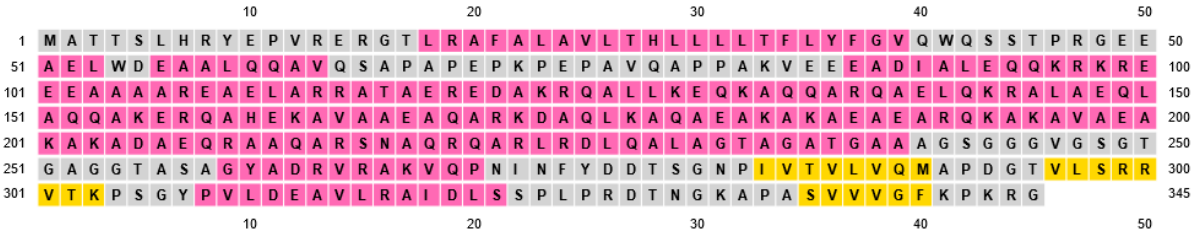

MATTSLHRYEPVRERGTLRAFALAVLTHLLLLTFLYFGV
**QWQSSTPRGEEAELWDEAALQQAVQSAPAPEPKPEPAVQAPPAKVEEEADIALEQQKRKRE
EEAAAAREAELARRATAEREDAKRQALLKEQKAQQARQAELQKRALAEQLAQQAKERQAH
EKAVAAEAQARKDAQLKAQAEAKAKAEAEARQKAKAVAEAKAKADAEQRAAQARSNAQR
QARLRDLQALAGTAGATGAAAGSGGG**
VGSGTGAGGTASAGYADRVRAKVQPNINFYDDTSGNPIVTVLVQMAPDGTVLSRRVTKPSGYP
VLDEAVLRAIDLSSPLPRDTNGKAPASVVVGFKPKRG

PPIIPRED:

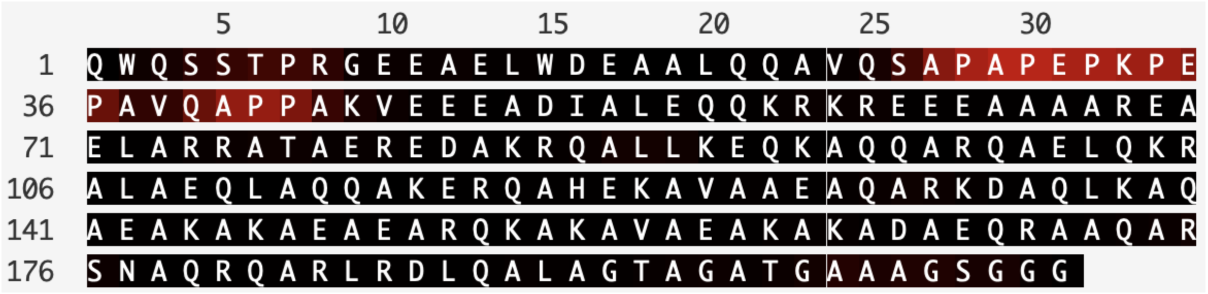

206 Domain II residues

8 Proline residues

164 α-Helix residues

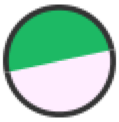

Bordetella pertussis

Locus: 84596- 92646

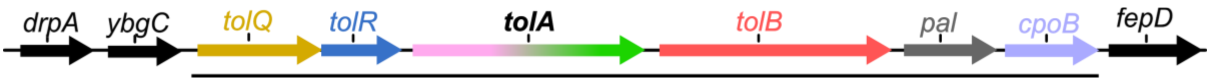

Flanked by *proS/drpA* tRNA synthase gene, and *fepD*, encoding an iron-importing TBDT.

>CAE43609.1 Proline-rich inner membrane protein [Bordetella pertussis Tohama I]

MTPPLIRSDRSAPPSPATQDNRKALGLAVLVHLLLLLVLIFGVNWRSENPGPVQVELWADGDSP
VNPPPDEVKPQPKPEPQPEPEPQPQPEPEPEPEPPPPPPPPPPPQPEVQPKEQPDPEIALEEA
RKKREQEEKARQAAEAAKEKARLEEERRQAELKEKQRLEQERRAAEKAAAEKAAAEKQAAEKK
AKEEAAKKAAADKALRDAFRNDALGAAGIPGGTADRNQAGGGRDSGYGAKVRACVQPGVAYP
PPPRAGSANPTAQYRVQLGSDGKVNGVTLTSSSGNPGFDRAVETGIRRCNPFPKPSTGRYEPII
DVVYRMYD

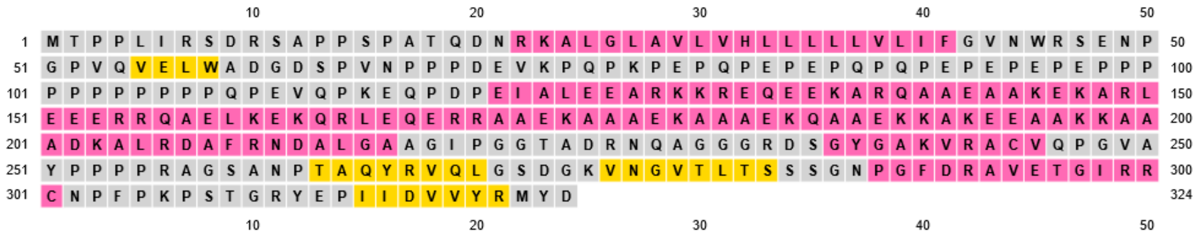

MTPPLIRSDRSAPPSPATQDNRKALGLAVLVHLLLLLVLIF
GVNWRSENPGPVQVELWADGDSPVNPPPDEVKPQPKPEPQPEPEPQPQPEPEPEPEPPPPP
PPPPPPQPEVQPKEQPDPEIALEEARKKREQEEKARQAAEAAKEKARLEEERRQAELKEKQR
LEQERRAAEKAAAEKAAAEKQAAEKKAKEEAAKKAAADKALRDAFRNDALGAAGIPGGTA
DRNQAGGGRDSGYGAKVRACVQPGVAYPPPPRAGSANPTAQYRVQLGSDGKVNGVTLTSSS
GNPGFDRAVETGIRRCNPFPKPSTGRYEPIIDVVYRMYD

PPIIPRED:

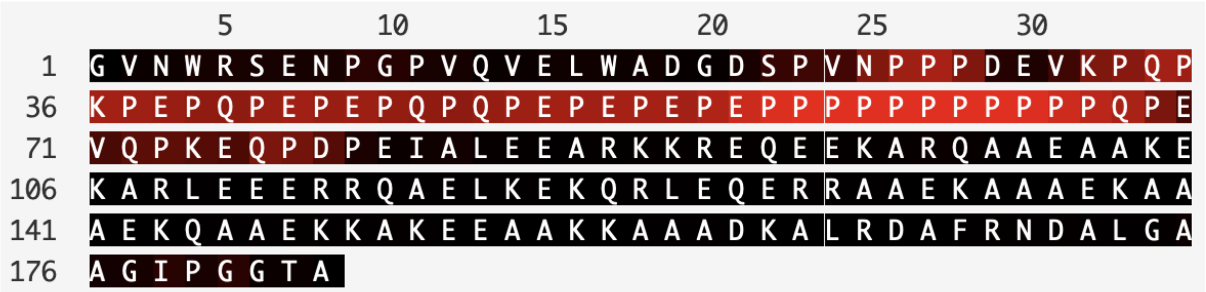

183 Domain II residues

34 Proline residues

96 α-Helix residues

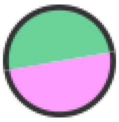

Acidovorax avenae

Split system.

We do not know for sure that we have correctly identified TolA, but α-helical content suggestive of TolA- no observed TonBs with this extent of α-helices. Gene is labelled as *tolA* and is preceded by *ybgC*.

Locus: *tolQRA*: 3293705-3300874

tolB-*pal*-*cpoB*: 2027060- 2032631

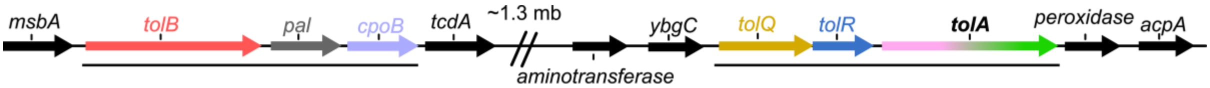

tolQRA flanked by an aspartate transaminase gene and a gene for a di-heme cytochrome c peroxidase, *acpA* for an acid phosphatase.

tolB-pal-cpoB flanked by *msbA*, encoding a ATP-driven lipid A transporter, and a gene for UBA/THIF-type NAD/FAD binding protein.

TolB predicted to use TAT pathway:

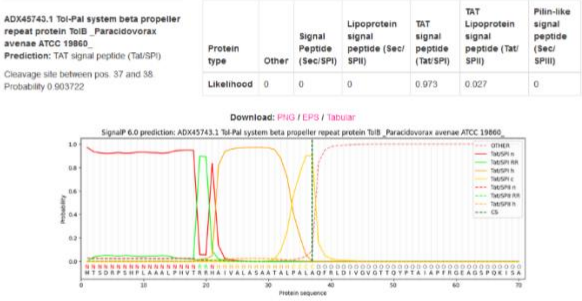

>ADX46819.1 protein TolA [Paracidovorax avenae ATCC 19860]

MHAHNDRDQFAPTRPPGRLRAIALAVLVHAALIGALTWGVNWKTSADQPAIEAEIWSALPQQAAPAAVAPPPPPQPVQQPTPPAPPPPA
PAPPPPPPPPKAAPDPREADIAIEREKKRLEQEKKERQLQAEQDRRERERKEQAEQEKKERQQKEKAQREKDQQQQREKEQREKDRR
EQQEKLEKQQAEKERQEQLQKKQAEDKRKADEKRKADAADAKRLEALRQENLRRMQGLAGATGGETATGNAQRSSGPSGSYGGKVA
AKVRPNIVYPDAISDNLRTEVEVRASPDGTIVGIRVTKSSGNKSWDDAVVRALEKTDTLPRDVDGRVPSSLVIGFRPKD

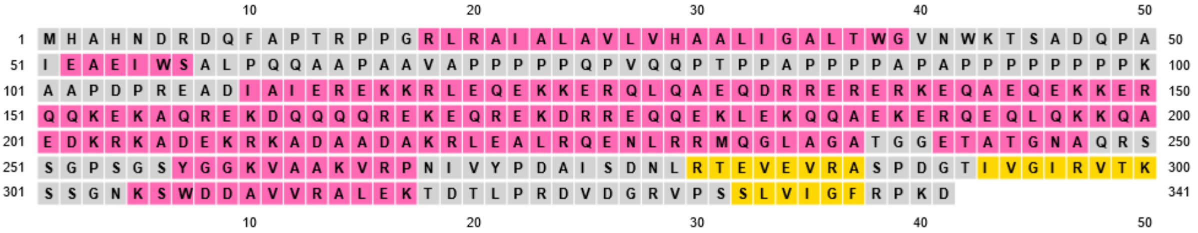

MHAHNDRDQFAPTRPPGRLRAIALAVLVHAALIGALTWG
VNWKTSADQPAIEAEIWSALPQQAAPAAVAPPPPPQPVQQPTPPAPPPPAPAPPPPPPPPKAAPDPREADIAIEREKKRLEQEKKERQLQAEQDRRERERKEQAEQEKKERQQKEKAQREKDQQQQREKEQREKDRREQQEKLEKQQAEKERQEQLQKKQAEDKRKADEKRKADAADAKRLEALRQENLRRMQGLAGATGGE
TATGNAQRSSGPSGSYGGKVAAKVRPNIVYPDAISDNLRTEVEVRASPDGTIVGIRVTKSSGNKSWDDAVVRALEKTDTLPRDVDGRVPSSLVIGFRPKD

PPIIPRED:

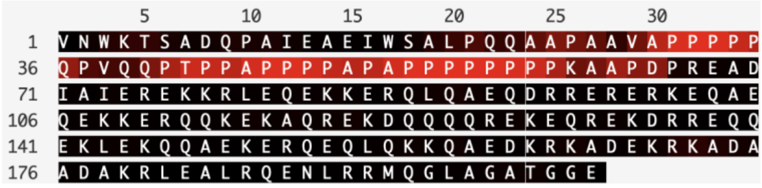

202 Domain II residues

27 Proline residues

135 α-Helix residues

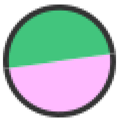

Rhodocyclales

Aromatoleum aromaticum

Locus: 1575164- 1583394

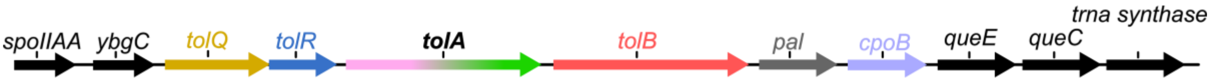

Flanked by spoIIAA anti-anti-sigma factor

>CAI07615.1 hypothetical protein ebA2648 [Aromatoleum aromaticum EbN1]

MNGAVPNSPRDEPGKWASLALTIAVHLGLIAFLVLGVRWQSTPPAALEVDLVAAVPARPAPLPK
PEPEAKPKPEPKPEPKPEPKPIPRPEPPKPAPPPEPPKAQAKPDIAMKAPEAKKPVKPEPPKPEP
PKPEPKKPEPKPEPKPPEPKKPEPKPEPKKPEPAKPEPKKPQPVVPPKDDYMAQRLAQETERT
QLERMMRADAARASAARNRAAGDAYENAVRTKVRGNLLRPPGLSGNPEAVFEVDQLPSGEVL
AIRLKRSSGIPALDEAIERAIRRSSPLPLPQAKELFERTLELKFKPLDED

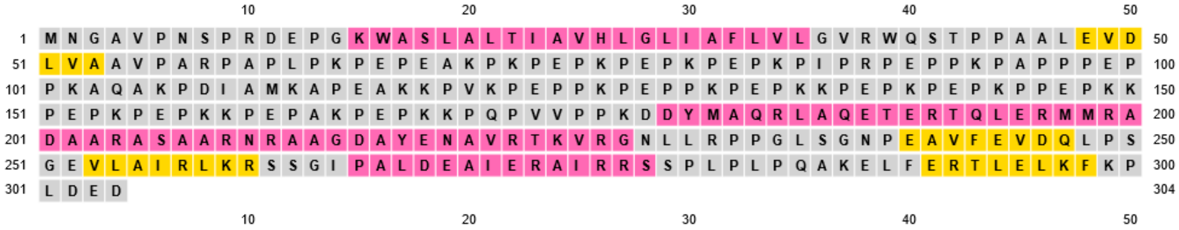

MNGAVPNSPRDEPGKWASLALTIAVHLGLIAFLVL
**GVRWQSTPPAALEVDLVAAVPARPAPLPKPEPEAKPKPEPKPEPKPEPKPIPRPEPPKPAPPP
EPPKAQAKPDIAMKAPEAKKPVKPEPPKPEPPKPEPKKPEPKPEPKPPEPKKPEPKPEPKKP
EPAKPEPKKPQPVVPPKDDYMAQRLAQETERTQLERMMRADAAR**
ASAARNRAAGDAYENAVRTKVRGNLLRPPGLSGNPEAVFEVDQLPSGEVLAIRLKRSSGIPALD
EAIERAIRRSSPLPLPQAKELFERTLELKFKPLDED

PPIIPRED:

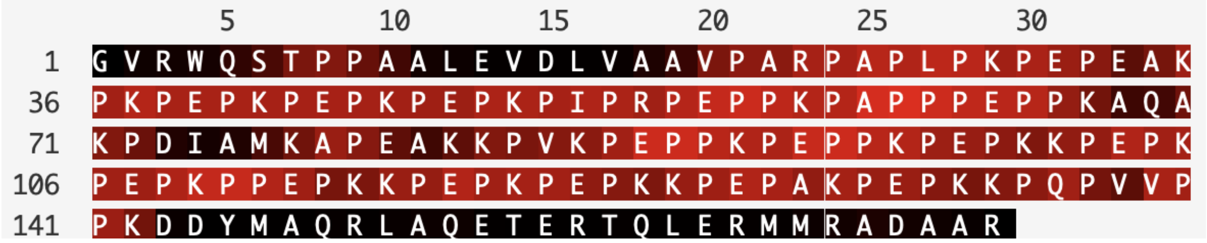

169 Domain II residues

56 Proline residues

26 α-Helix residues

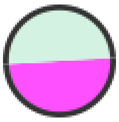

Nitrosomonadales

Thiobacillus denitrificans

Locus: 2283098- 2290686

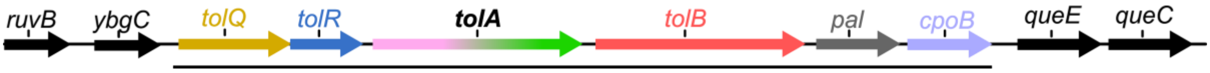

Flanked by *ruvB* and *queEC*.

>AAZ98160.1 putative TonB protein [Thiobacillus denitrificans ATCC 25259]

MTRGDGRFVSRPVPRLAAGLLALGVHLLFVLLLVFGVSWQTRHPAPVTVDLWESIPQPPAPRPP
VPAPPPPEPPQPAPRPVPEPEVKNEPPPPKAPDIALEKKKAAAAKAAALEKAALEKAAQEKAAQ
EKAAQEKAAQQKAEQEKAAAQRKRDLLRQMEEEDAIRRMADEEAANQARLLKQAEAKAAAGA
RQSEIARLVGQHRDLISAKVRGNTRLPDNLAGNPEVRCLVRLLPTGEVQSVRVTRSSGNPAYDD
AVKRAIEKSSPLPLPADRDARAEFVPELSFVHRPKE

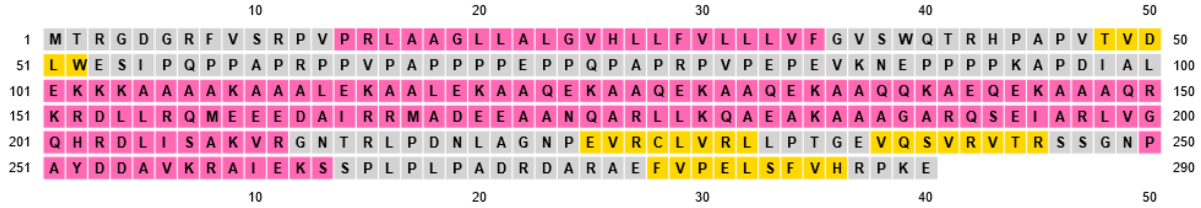

MTRGDGRFVSRPVPRLAAGLLALGVHLLFVLLLVF
GVSWQTRHPAPVTVDLWESIPQPPAPRPPVPAPPPPEPPQPAPRPVPEPEVKNEPPPPKAPDI ALEKKKAAAAKAAALEKAALEKAAQEKAAQEKAAQEKAAQQKAEQEKAAAQRKRDLLRQM EEEDAIRRMADEEAANQARLLKQAEAKAAAGA
RQSEIARLVGQHRDLISAKVRGNTRLPDNLAGNPEVRCLVRLLPTGEVQSVRVTRSSGNPAYDD
AVKRAIEKSSPLPLPADRDARAEFVPELSFVHRPKE

PPIIPRED:

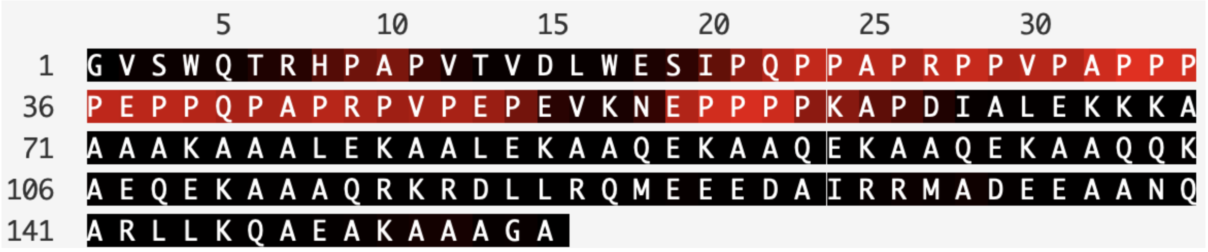

155 Domain II residues

25 Proline residues

90 α-Helix residues

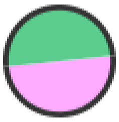

Methylobacillus flagellatus

Locus: 2490296- 2497252

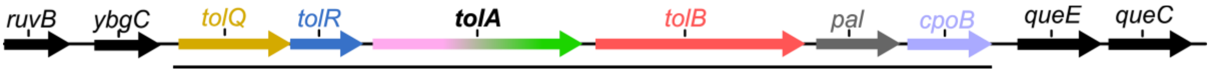

Flanked by a *queE* and *queC* encoding a radical SAM 7-carboxy-7-deazaguanine synthase.

>ABE50607.1 Cell division and transport-associated protein TolA [Methylobacillus flagellatus KT]

MKKRRANPYKARAAALSLFVHLLLLALLLVSFEWKTQPPVMVAQVELWESLPADNPVVKPPQP
QPVPEPPTPEPKPAPQPEPKPEPKPEPAPEPQAEIQVKKEPEKPKPPKPEKKPEPKKPEKKPDP
KKPEPKKPVPKKEDKVKLDELKKLQQELLEESSQPAPAQKQGPTQLGANVANQGEIDKYLGQIR
DKIRRNVNRQLCGTGKPELEVGISLMPTGEVIGVPRILKSSGLPACDEAIERAILQAQPLPVPTQP
DLFSKFRDLRLKFRPNEEN

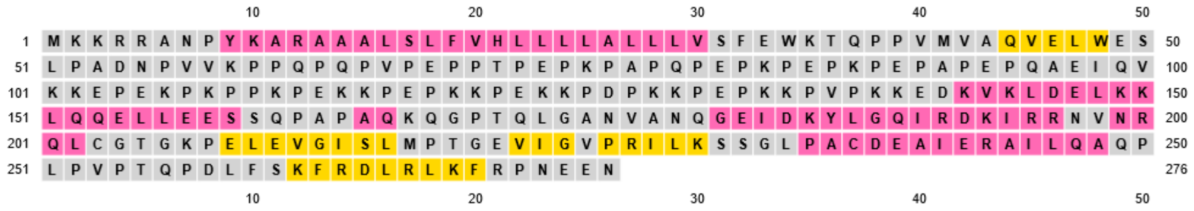

MKKRRANPYKARAAALSLFVHLLLLALLLV
SFEWKTQPPVMVAQVELWESLPADNPVVKPPQPQPVPEPPTPEPKPAPQPEPKPEPKPEPAP EPQAEIQVKKEPEKPKPPKPEKKPEPKKPEKKPDPKKPEPKKPVPKKEDKVKLDELKKLQQE LLEESSQPAPAQKQGPTQLGAN
VANQGEIDKYLGQIRDKIRRNVNRQLCGTGKPELEVGISLMPTGEVIGVPRILKSSGLPACDEAIE
RAILQAQPLPVPTQPDLFSKFRDLRLKFRPNEEN

PPIIPRED:

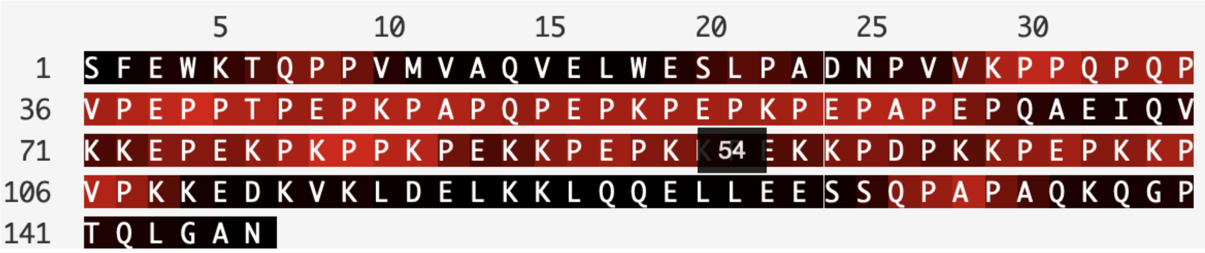

146 Domain II residues

40 Proline residues

20 α-Helix resides

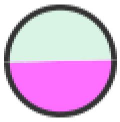

Nitrosomonas europaea

Locus: 247919-254874

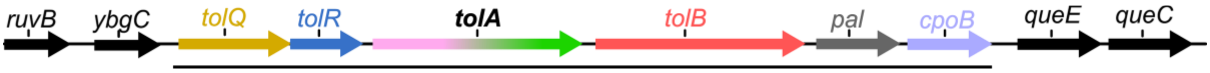

Flanked by *ruvB* and *queE*, encoding a radical SAM 7-carboxy-7-deazaguanine synthase.

>CAD84128.1 Proline-rich region [Nitrosomonas europaea ATCC 19718]

MVRLPGDNSEPGKLRAALFALLVHAAFLALLVFGLNWKNEVSEMMSVDLWAELPRHPVEPPSS
AAKVIPEPVKVKPQPQQKTQPQPQPVIKAAPPPVRKPDIALKDKTEKPQLKEEVKKPEPVKKEVP
KKEPTKKPVEKLVPKEEVKKPEPVKKVEQKTEKKEDVRQQAEAQKQAQQREREAAAAKAERAR
ADGEIEKYREMIKAKIRSRIIMPPDLPGNPAVEFTVTLLPGGDVLTVTLRKSSGYTAFDEAVERAIY
LAKPLPLPPDPGLFNAFRNLDLKVYYRE

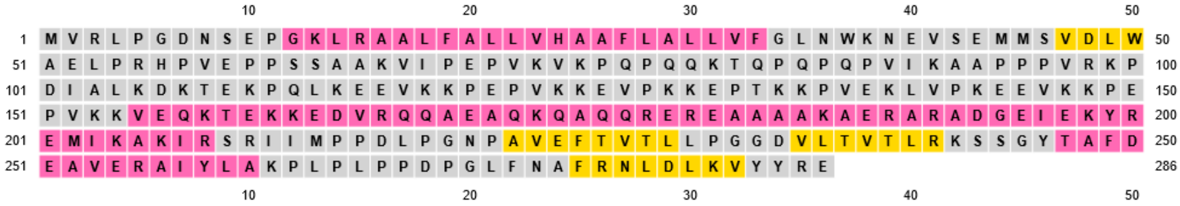

MVRLPGDNSEPGKLRAALFALLVHAAFLALLVF
**GLNWKNEVSEMMSVDLWAELPRHPVEPPSSAAKVIPEPVKVKPQPQQKTQPQPQPVIKAAPP
PVRKPDIALKDKTEKPQLKEEVKKPEPVKKEVPKKEPTKKPVEKLVPKEEVKKPEPVKKVEQK
TEKKEDVRQQAEAQKQAQQREREAAAAK**
AERARADGEIEKYREMIKAKIRSRIIMPPDLPGNPAVEFTVTLLPGGDVLTVTLRKSSGYTAFDEA
VERAIYLAKPLPLPPDPGLFNAFRNLDLKVYYRE

PPIIPRED:

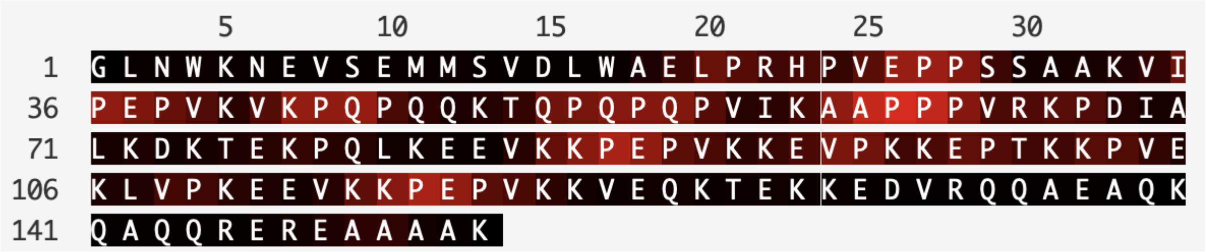

153 Domain II residues

24 Proline residues

32 α-Helix residues

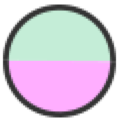

Gallionella capsiferriformans

Locus: 2781569-2788081

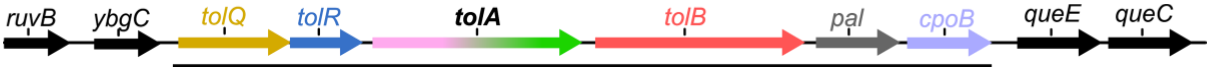

Flanked by *ruvB* and two radical SAM *queEC* genes for a 7-carboxy-7-deazaguanine synthases.

>ADL56582.1 protein TolA [Gallionella capsiferriformans ES-2]

MNTVVYQDLYRLPACLLAVLVHGAFLALLYFGFSWQTEPPVVMSVELWQSMPDSAPATPVEAR
VEEVAPVEPEVEIKPEIVVPDKKPEKKPEKKPEIKPPEKKPEIKPVVKPEVKKPLDVKKTAPQPAQ
PSAAEQQAARDKATQEAATGRVVDEFVGKIQGKIRRNVVEPPDVSKEARAEFLVTVLPGGRVLP
PRLLKSSGNPAYDNAVERAILKSDPLPLPADAALFNRFRELKLGFQPDNK

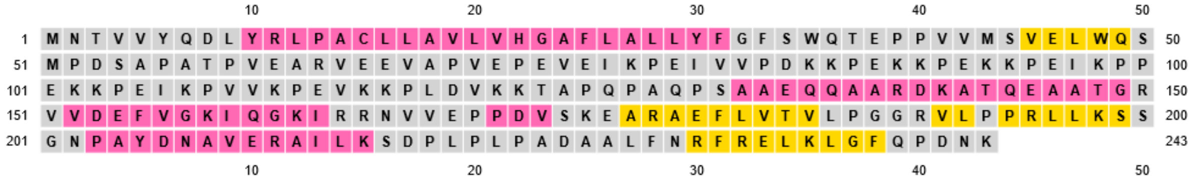

MNTVVYQDLYRLPACLLAVLVHGAFLALLY
**FGFSWQTEPPVVMSVELWQSMPDSAPATPVEARVEEVAPVEPEVEIKPEIVVPDKKPEKKPEK
KPEIKPPEKKPEIKPVVKPEVKKPLDVKKTAPQPAQPSAAEQQAARDKAT**
QEAATGRVVDEFVGKIQGKIRRNVVEPPDVSKEARAEFLVTVLPGGRVLPPRLLKSSGNPAYDN
AVERAILKSDPLPLPADAALFNRFRELKLGFQPDNK

PPIIPRED:

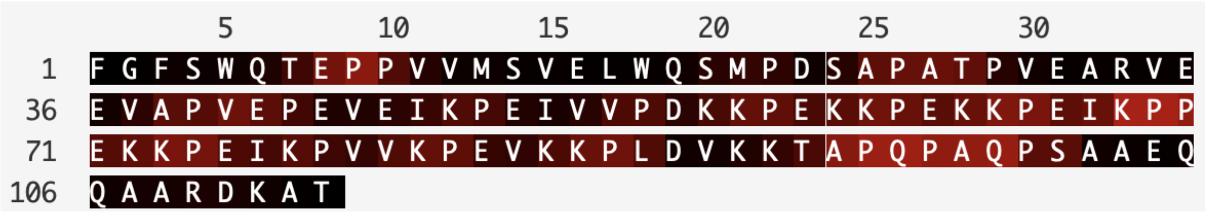

112 Domain II residues

21 Proline residues

13 α-Helix residues

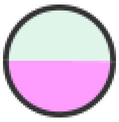

Acidithiobacillales

Acidithiobacillus caldus

Locus: 10192- 18965

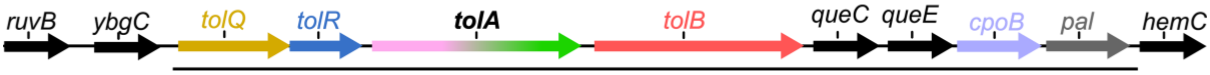

Features interloping *queC* and *queE* genes for 7-carboxy-7-deazaguanine synthases, and flanked by *hemC*, encoding a hydroxymethylbilane synthase.

>OFC35716.1 protein TolA [Acidithiobacillus caldus]

MKREPRTAPLVLAILLNAALLVALFWTFHVNIPRVGSTPMQAQLISSAATPAATPSVKPAAVEPAK
ATPKPAPTPPPKPETKPQPKPQPSPKLEAQAQAKAQAREQAEEAQKQHKAQEKAAELAAQRAA
AERQAQELAAQKRAAKLAAQRRAQEKAAAQKAAAEKAAAEKAAAEKAAAEKAAAERAAQLKKE
LAEQAAKAAEAARAKALRERMEAQAKLQAEANIAAARAAQAAEDQKLSALFSQEVQRRVYRQW
DTNFAAALSCVVQIHLSPTGAIIGAPKILRSSGNPQFDRAVIAAVEQAAPFVPPLGLSYNAYREVNI
QFNAEELNHG

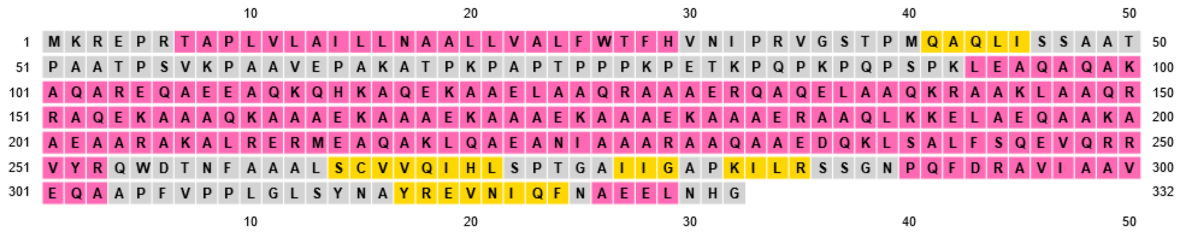

MKREPRTAPLVLAILLNAALLVALFWTFH
**VNIPRVGSTPMQAQLISSAATPAATPSVKPAAVEPAKATPKPAPTPPPKPETKPQPKPQPSPK
LEAQAQAKAQAREQAEEAQKQHKAQEKAAELAAQRAAAERQAQELAAQKRAAKLAAQRR
AQEKAAAQKAAAEKAAAEKAAAEKAAAEKAAAERAAQLKKELAEQAAKAAEAARAKALRE
RMEAQAKLQAEANIAAARAAQ**
AAEDQKLSALFSQEVQRRVYRQWDTNFAAALSCVVQIHLSPTGAIIGAPKILRSSGNPQFDRAVI
AAVEQAAPFVPPLGLSYNAYREVNIQFNAEELNHG

PPIIPRED:

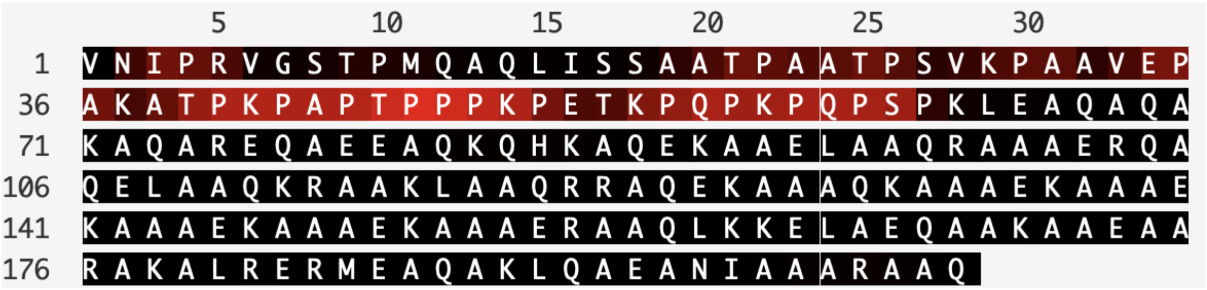

203 Domain II residues

18 Proline residues

140 α-Helix residues

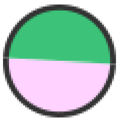

Acidithiobacillus ferrooxidans

Locus: 9503- 18303

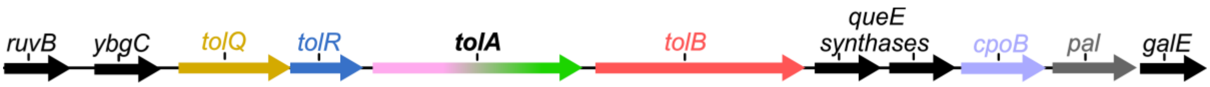

Flanked by *ruvB* and *galE*. Features two interloping *que* genes for 7-carboxy-7-deazaguanine synthases.

>OAP91747.1 protein TolA [Acidithiobacillus ferrooxidans]

MKERKSFWPLALAIALNSAILAALFWSFHMEVPSAGDTPMQAQLSSGMPPVAAPAPQPKPAPK
PAPVPPPAPKPVPVPKPKPSTPPPPSAAQKAAEKAAQEKAAEQKTAQITAEKQAAEKRAEELAA
RAAHEKAAQRAAAKATAQKAAAEKAAAEKQAAQKAAAAQRAAALKQQLAKQQAKAAEAARAK
ALQQRMEARAKLQAEANMAAALAKQAAENQRLSAIFGSEIAERVKRHWQPVFAPNLHCQVRIKL
SPQGQLEGDPAITQSSGNAQFDSAVIAAIEAAAPFPPPIGLSYSEFKVVNIVFSAKELSNG

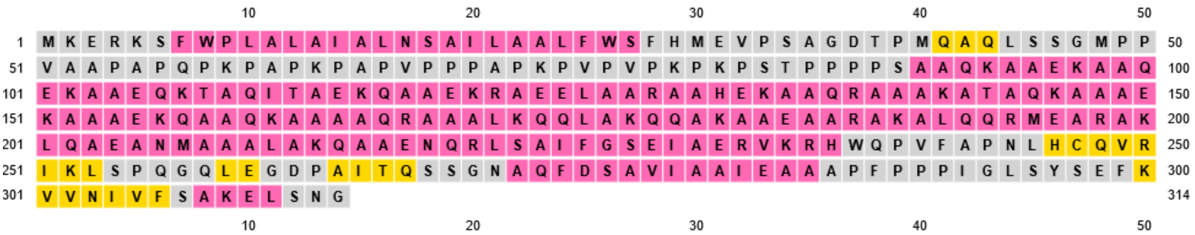

MKERKSFWPLALAIALNSAILAALFWS
**FHMEVPSAGDTPMQAQLSSGMPPVAAPAPQPKPAPKPAPVPPPAPKPVPVPKPKPSTPPPP
SAAQKAAEKAAQEKAAEQKTAQITAEKQAAEKRAEELAARAAHEKAAQRAAAKATAQKAA
AEKAAAEKQAAQKAAAAQRAAALKQQLAKQQAKAAEAARAKALQQRMEARAKLQAEANM
AAALAKQ**
AAENQRLSAIFGSEIAERVKRHWQPVFAPNLHCQVRIKLSPQGQLEGDPAITQSSGNAQFDSAVI
AAIEAAAPFPPPIGLSYSEFKVVNIVFSAKELSNG

PPIIPRED:

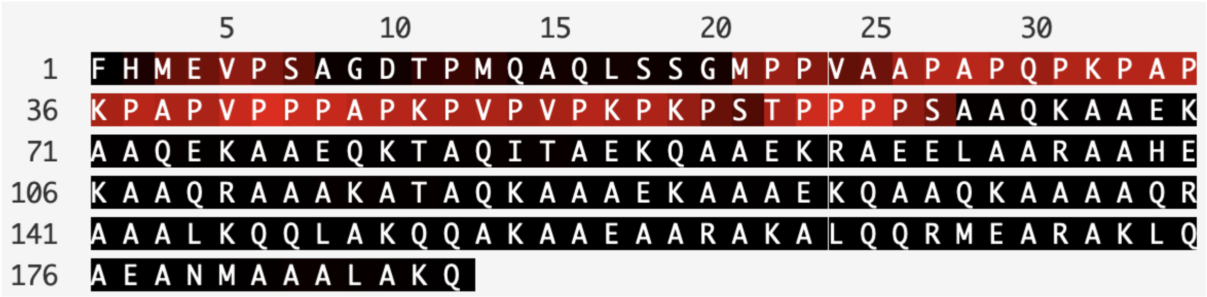

187 Domain II residues

24 Proline residues

125 α-Helix residues

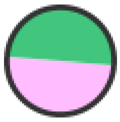

α-Proteobacteria

Magnetococcales

Magnetococcus marinus

Locus: 563676-572563

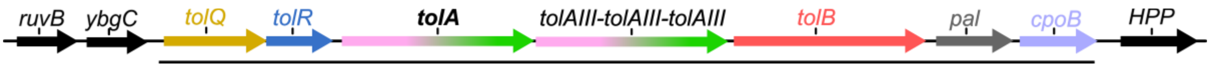

Note there is a transposase system just upstream. Flanked by HPP-family gene, which form integral inner membrane proteins comprising 4x TMHs. There is also a strange gene comprising three repeats of TolA domain III

>ABK42994.1 Cell division and transport-associated protein TolA [Magnetococcus marinus MC-1]

MFADRQFLWWSLGLHVGVVLIALLLPLLTPRVAPPPAAMMVNLVDLPAPQAKATQPEPPQLQKP
KPPEPKPVAPKPVEPKPVESKPVAKPEIEPTPPEPKPQPAPKPPEKVVEEPDQPAEKVDITPMR
RKPDPKKIAEEQRAKEEALKKKQAEEKRLQEEAAKKKQAEEQRKKEEARKQAEEQRKKEEARK
QAEIKRKQELAAQQMREAIAKQQAEIKAQQQREEAIRQQQLREAALAQQQAEEIRKWQGALRE
ATYKAWRPPFGVDISSMKCLATVKITVDGRLSLISISQTSGFAPYDDSVQRAIAATRNLPKPPSHC
SACRGEITISFTPSR

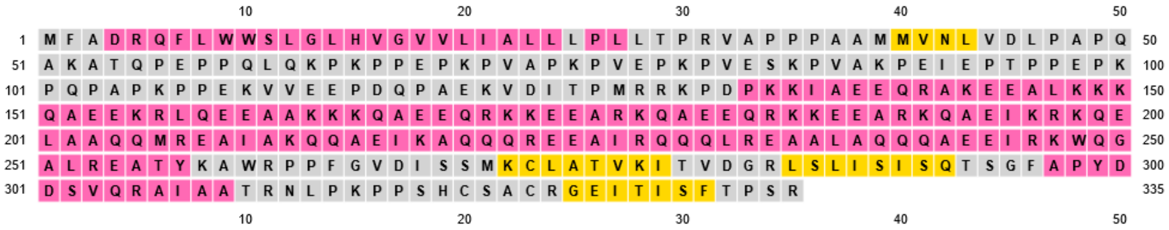

MFADRQFLWWSLGLHVGVVLIALL
**LPLLTPRVAPPPAAMMVNLVDLPAPQAKATQPEPPQLQKPKPPEPKPVAPKPVEPKPVESKP
VAKPEIEPTPPEPKPQPAPKPPEKVVEEPDQPAEKVDITPMRRKPDPKKIAEEQRAKEEALKK
KQAEEKRLQEEAAKKKQAEEQRKKEEARKQAEEQRKKEEARKQAEIKRKQELAAQQMREAI
AKQQAEIKAQQQREEAIRQQQLREA**
ALAQQQAEEIRKWQGALREATYKAWRPPFGVDISSMKCLATVKITVDGRLSLISISQTSGFAPYD
DSVQRAIAATRNLPKPPSHCSACRGEITISFTPSR

PPIIPRED:

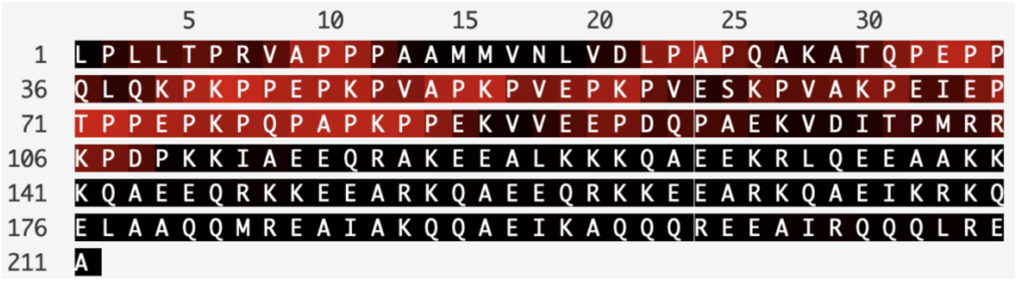

211 Domain II residues

35 Proline residues

103 α-helix residues

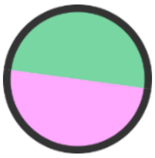

Rickettsiales

Rickettsia prowazekii

----No *tolA* found, orphan *tonB*

Anaplasma marginale

----No *tolA* found, orphan *tolB*

Rhodospirillales

Acetobacter pasteurianus

Locus: 33835- 44100

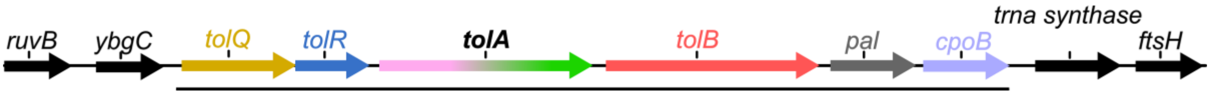

>OAZ61227.1 RE1-silencing transcription factor [Acetobacter pasteurianus]

MPMARPRRSDQIILRRSLYLSGGAHVALLLALLITLPPPKPPEEPPQPTIEMQFEQSDAQEGGPA
AKAEKAAAEPKPPAPEEKEAPPTPEPPKDVPNEEAPPPPPPPQPVPPPPVPESEKLPDVPLPPK
AEEPSPEVVKTPPSPPSPVQPTNSVAPPSPITQPTDTLPDEAVPSHITQPNKAKKSQAESHSLLE
TLDTFRADQKQTHAPKARANPVAGGAPKGGGSPVGSNITGSLSAGQQKAIGAAVRRCYTEDTA
AKDYASFVAHLVVTVDGTGEARIVQFAPETQARMNADSSYRALAERARAAVLSPTCAKLPIPKEL
LGQPRQLKFVFRP

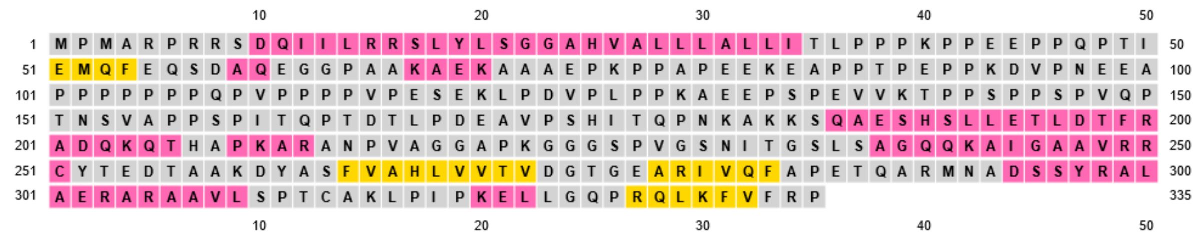

MPMARPRRSDQIILRRSLYLSGGAHVALLLALLI
**TLPPPKPPEEPPQPTIEMQFEQSDAQEGGPAAKAEKAAAEPKPPAPEEKEAPPTPEPPKDVP
NEEAPPPPPPPQPVPPPPVPESEKLPDVPLPPKAEEPSPEVVKTPPSPPSPVQPTNSVAPPSPI
TQPTDTLPDEAVPSHITQPNKAKKSQAESHSLLETLDTFRADQKQTHAPKARANPVAGGAPK
GGGSPVGSNITGS**
LSAGQQKAIGAAVRRCYTEDTAAKDYASFVAHLVVTVDGTGEARIVQFAPETQARMNADSSYR
ALAERARAAVLSPTCAKLPIPKELLGQPRQLKFVFRP

PPIIPRED:

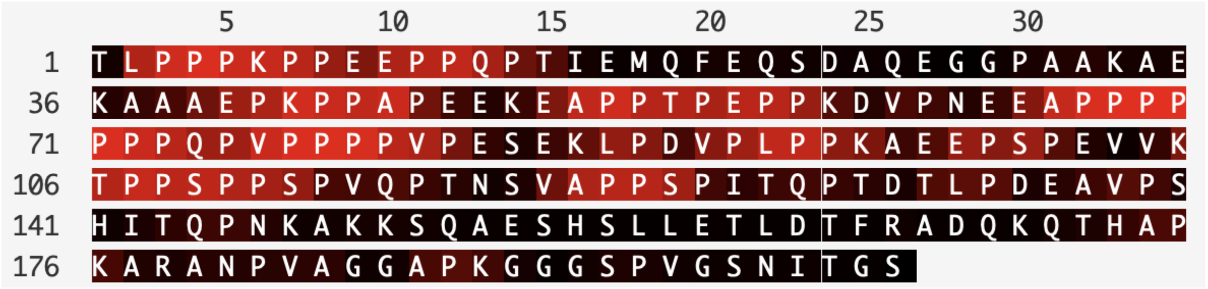

201 Domain II residues

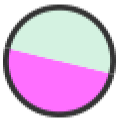

55 Proline residues

31 α-Helix residues

Rhodospirillum centenum

Locus: 1851409-1861805

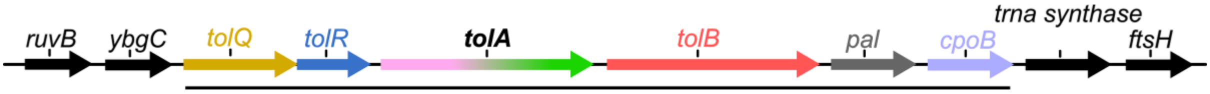

Flanked by *ruvB* and *ftsH*.

>ACI99198.1 protein TonB, putative [Rhodospirillum centenum SW]

MRRALVLSGVLHVALFLIALFGLPALKSQDVLMLQEIPVDVVEVGPMTIARLQQDAPKPRAQPKP
QPPKPEPPKPEPPKQEPPPPPPKAQALPPPPEPEPTPKPEPPKPPVKPEPAPPKPEPKPEPEPK
PEPKKEEPKKPEPPKPEPKKEQPKPEPKKEEPKPKPEPKKEEPKPKPEPKKEEPKPKPERTLSD
LLNSLEKEQPAPPAKEKPQERPVESGSPPAAAGERLSISEEDALRRQIGQCWNVPSGARGVES
MQAEIRVLFDANMRVTGVQFIRGNGNLSDPHFRAFVESALRAPQLPACATLNLPRDKYGNRGSI
VMNFSPRDMF

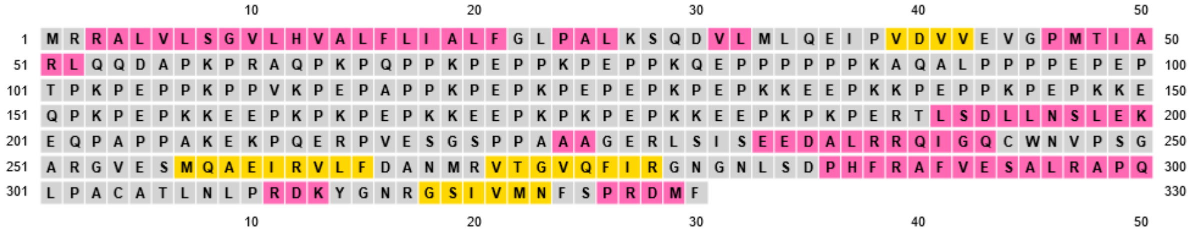

MRRALVLSGVLHVALFLIALF
**GLPALKSQDVLMLQEIPVDVVEVGPMTIARLQQDAPKPRAQPKPQPPKPEPPKPEPPKQEPP
PPPPKAQALPPPPEPEPTPKPEPPKPPVKPEPAPPKPEPKPEPEPKPEPKKEEPKKPEPPKPE
PKKEQPKPEPKKEEPKPKPEPKKEEPKPKPEPKKEEPKPKPERTLSDLLNSLEKEQPAPPAK
EKPQERPVESGSPPAAAGERLS**
ISEEDALRRQIGQCWNVPSGARGVESMQAEIRVLFDANMRVTGVQFIRGNGNLSDPHFRAFVE
SALRAPQLPACATLNLPRDKYGNRGSIVMNFSPRDMF

PPIIPRED:

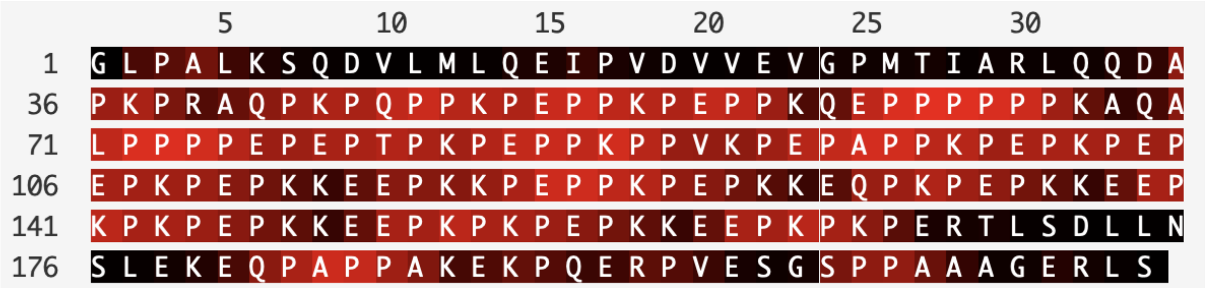

209 Domain II residues

71 Proline residues

24 α-Helix residues

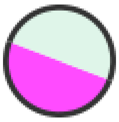

Sphingomonadales

Erythrobacter litoralis

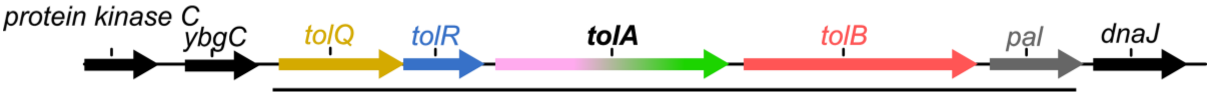

Locus: 23076- 28023

Flanked by *dnaJ* and protein kinase C gene

Used BLAST to search for CpoB, no matches.

>KEO89893.1 energy transducer TonB [Erythrobacter litoralis]

MGETATFRSEDRTGLLAALVLHALLVAVLALQWSMSPPPPVSQRMTVSLASEVGLEATAPDPVP
ESRAAIAPTLAESPAPEAEAVPAETRAQIPAPQTPTRTAARESAPSRERSRPDRQPARGTPSRP
AREEPAGGSRIGENFLEGAGSSTTTDETRIPASQIGASAKASIIQAIVRQIRPHWTAPSGADAELL
VTELAFDLNEDGSLKGRPRVLRQGGVNDANRAQQALHAERAIRAVQLAAPFDLPDEYYEAWKSI
RGARFDRNLSR

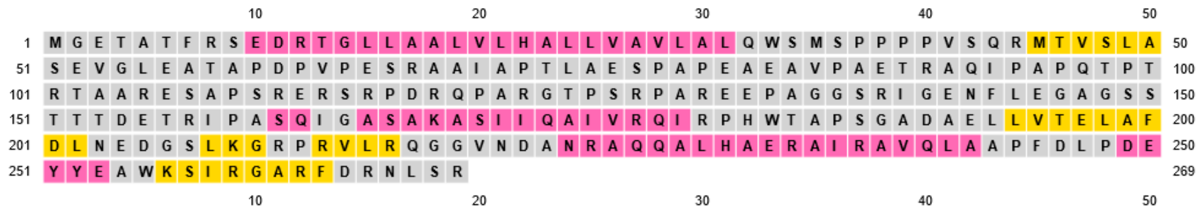

MGETATFRSEDRTGLLAALVLHALLVAVLAL
**QWSMSPPPPVSQRMTVSLASEVGLEATAPDPVPESRAAIAPTLAESPAPEAEAVPAETRAQIP
APQTPTRTAARESAPSRERSRPDRQPARGTPSRPAREEPAGGSRIGENFLEGAGSSTTTDET
RIPASQIGASAKA**
SIIQAIVRQIRPHWTAPSGADAELLVTELAFDLNEDGSLKGRPRVLRQGGVNDANRAQQALHAE
RAIRAVQLAAPFDLPDEYYEAWKSIRGARFDRNLSR

PPIIPRED:

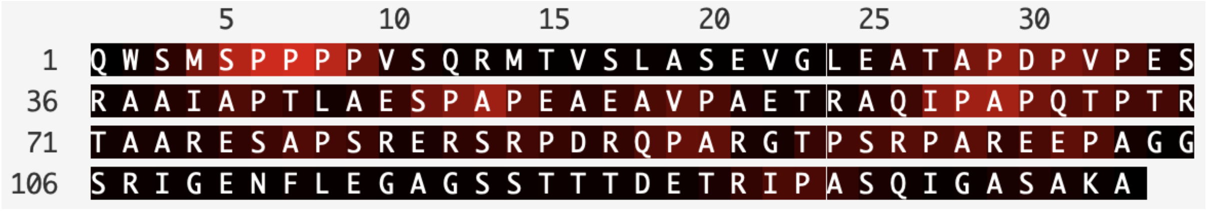

138 Domain II residues

21 Proline residues

7 α-Helix residues

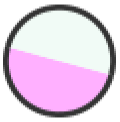

Sphingomonas wittichii

Locus: 2391681- 2400371

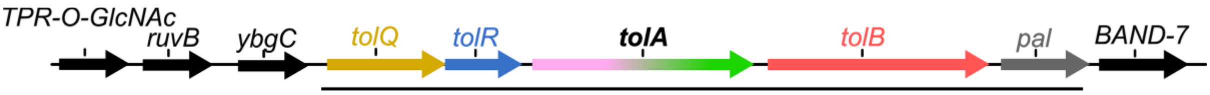

Flanked by a gene for a large TPR-OglcNAc multidomain protein (not CpoB but may fulfil similar role), *ruvB* and a gene encoding an uncharacterised Band 7 protein. No CpoB homologues detected by BLAST.

>ABQ68493.1 hypothetical protein Swit_2134 [Rhizorhabdus wittichii RW1]

MDRAEGAGLGISIAGHVALLVILSLNLMSVRKLPKLSEPMDVMLVDKAGLTSAAPEVSKEPPQAA
QAPEVGPTEETPPEPTPAPPKPQPAPPTPPRPAPTPKPAPKPVPQKPAPTPAPEKAVEKPTPAP
AKPKPKATSLGTDFLKGIPVEKSTGKAAAPRAAAISPIAMNGLVALIASQVKPCYTIPAGGTDTTSII
SRIRLRLKKDGSIAAAPEVIGNLGVTPANQPYVRQMNEAATRALQRCAPYKLPPDLFEAWQDLIF
TFRPAQMS

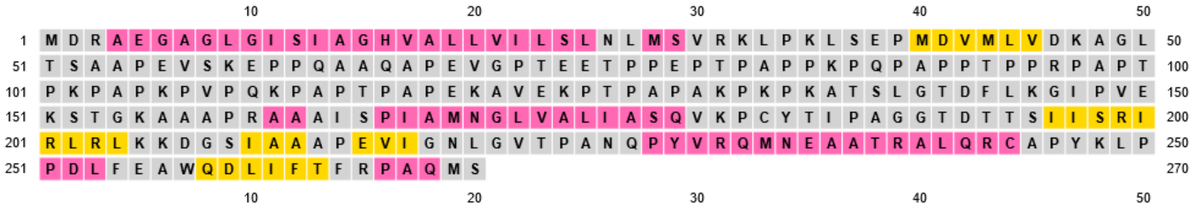

MDRAEGAGLGISIAGHVALLVILSL
**NLMSVRKLPKLSEPMDVMLVDKAGLTSAAPEVSKEPPQAAQAPEVGPTEETPPEPTPAPPKP
QPAPPTPPRPAPTPKPAPKPVPQKPAPTPAPEKAVEKPTPAPAKPKPKATSLGTDFLKGIPVE
KSTGKAAAPRAAAISPIAMN**
GLVALIASQVKPCYTIPAGGTDTTSIISRIRLRLKKDGSIAAAPEVIGNLGVTPANQPYVRQMNEAA
TRALQRCAPYKLPPDLFEAWQDLIFTFRPAQMS

PPIIPRED:

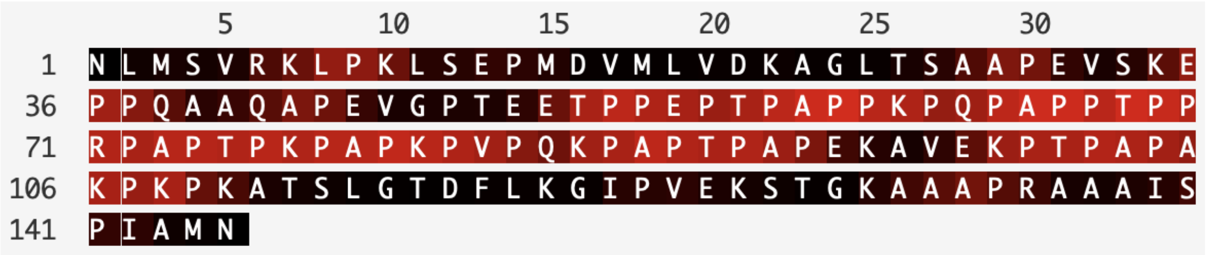

145 Domain II residues

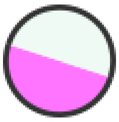

38 Proline residues

9 α-Helix residues

Rhodobacterales

Hyphomonas neptunium

Locus: 131539- 141876

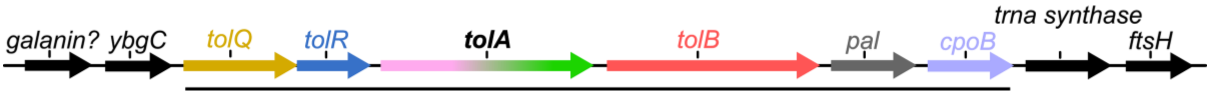

Flanked by DUF3137, encoding a putative secreted galanin homologue.

>ABI77259.1 conserved hypothetical protein [Hyphomonas neptunium ATCC 15444]

MLRGLTVSALVHASVLAMAVVSWPQDTNDCDDQIERLRREQPGISSVEILMQLPQCASSADLPI
DFLEIGRISDVAELRKSDTPEPEPEPEAAPEPVEETPPPPEPEAAEPEPEVEVPDPREPEPEPVE
EPEPKPEPKKEEPKPKPKPVEKPPAKPKDDLDFLDDFESTLQSKRASEQRPETQQTDRPVLANS
DRDQQGAGERTGATASLQAALRRQIMECWTGVDDLPPEHQIDVVVSVKLARDGSLAEAAKLISP
RSRPVGRSGIAVDRALLAVRKCGEIGNYRLPAEDYDLWKDINVTLGPKQG

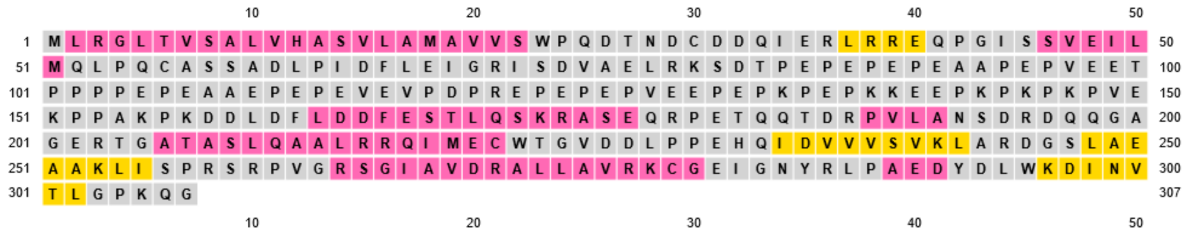

MLRGLTVSALVHASVLAMAVVS
**WPQDTNDCDDQIERLRREQPGISSVEILMQLPQCASSADLPIDFLEIGRISDVAELRKSDTPEPE
PEPEAAPEPVEETPPPPEPEAAEPEPEVEVPDPREPEPEPVEEPEPKPEPKKEEPKPKPKPVE
KPPAKPKDDLDFLDDFESTLQSKRASEQRPETQQTDRPVLANSDRDQQGAGERTGAT**
ASLQAALRRQIMECWTGVDDLPPEHQIDVVVSVKLARDGSLAEAAKLISPRSRPVGRSGIAVDR
ALLAVRKCGEIGNYRLPAEDYDLWKDINVTLGPKQG

PPIIPRED:

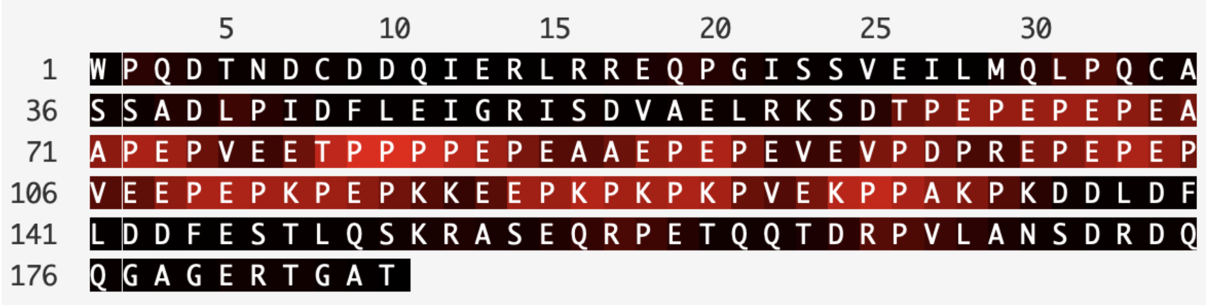

185 Domain II residues

35 Proline residues

27 Helix residues

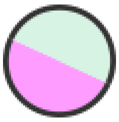

Rhodobacter capsulatus

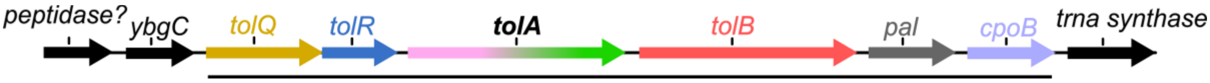

Locus: 64009- 71828

Flanked by an unknown peptidase gene (product) and a tRNA synthase gene

>SDE30424.1 hypothetical protein SAMN04244550_00068 [Rhodobacter capsulatus]

MRMDRAERIGTGVSAALHIAVILWAIIGTAWFKPEPNEPVEIKPAGMISEAEFAAMQAAAPKAGG
AAAAQPAPTQPEEPAAEPAPEEPPAEEAPTPEAVPDQPVPPEPKPEPPPDLSEIVPPTPPAEVT
EVPPSMMPPVEEPQDTEAPDFSPRPQPKPAQRVAPKPAEAPAPEVKVSETAEPETRPEEQPEP
EKPAEKPKEQAAPPEAATEIVPEEAKPVEQETTSAPKSSPRPPKKPAKPAVKPPEKTETAAAEDT
PAEKPADKPAEKAADKPAKPKSDKPAKPAKPASSKPAGADAVADALAEAMGAESDSGADTGTG
GLGIAKNGPPVTAGEKEALIVDVKACWNVGALSSEALRTTVTVRVDMQENGKPVVSSIKMVDAT
GGSDAAARQAFEAARRAIVRCGSDGFPLPSEKFGWWQQIEIVFNPTKMRMK

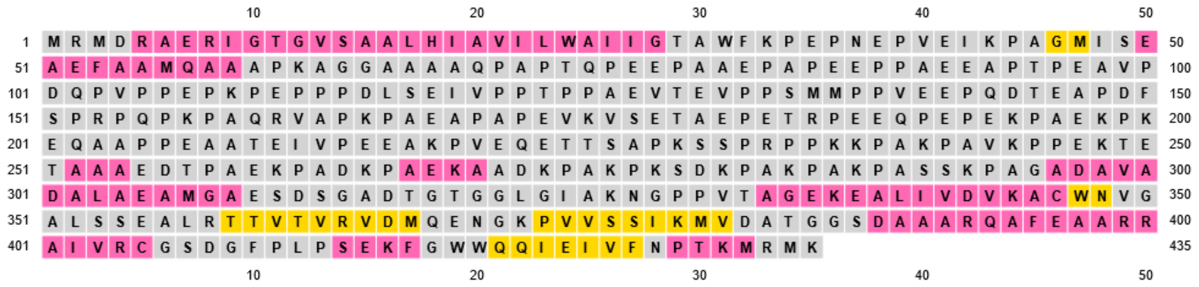

MRMDRAERIGTGVSAALHIAVILWAIIG
**TAWFKPEPNEPVEIKPAGMISEAEFAAMQAAAPKAGGAAAAQPAPTQPEEPAAEPAPEEPPA
EEAPTPEAVPDQPVPPEPKPEPPPDLSEIVPPTPPAEVTEVPPSMMPPVEEPQDTEAPDFSPR
PQPKPAQRVAPKPAEAPAPEVKVSETAEPETRPEEQPEPEKPAEKPKEQAAPPEAATEIVPEE
AKPVEQETTSAPKSSPRPPKKPAKPAVKPPEKTETAAAEDTPAEKPADKPAEKAADKPAKPK
SDKPAKPAKPASSKPAGADAVADALAEAMGAESDSGADTGTGGLGIAKNGPPVTAGE**
KEALIVDVKACWNVGALSSEALRTTVTVRVDMQENGKPVVSSIKMVDATGGSDAAARQAFEAA
RRAIVRCGSDGFPLPSEKFGWWQQIEIVFNPTKMRMK

PPIIPRED:

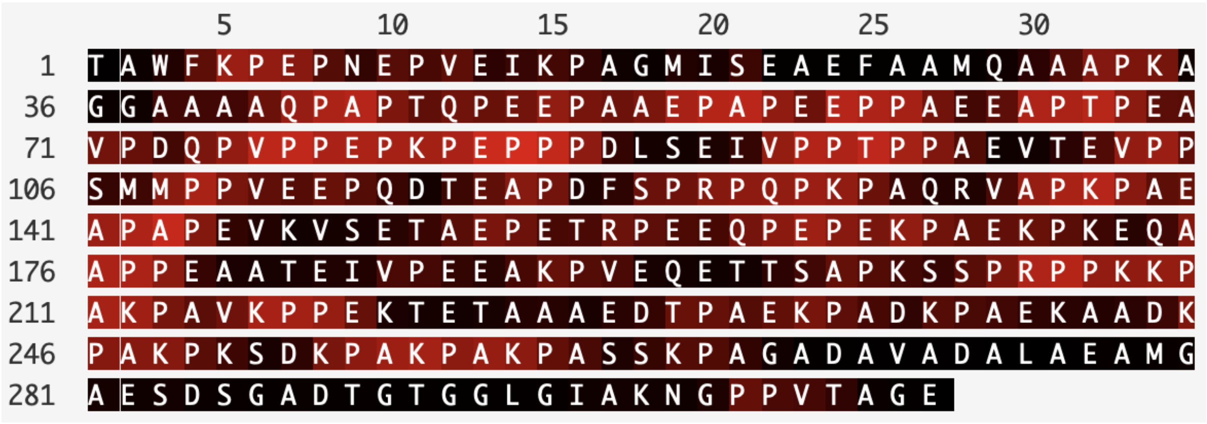

307 Domain II residues

71 Proline residues

34 α-Helix residues

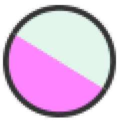

Caulobacterales

Caulobacter crescentus

Locus: 3490502- 3499234

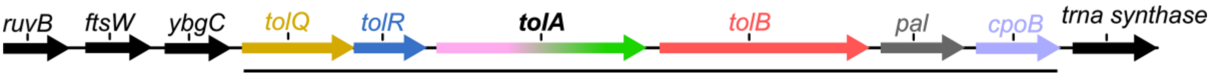

Flanked by *ruvB*, *ftsW* and tRNA synthase.

>AAK25193.1 hypothetical protein CC_3231 [Caulobacter vibrioides CB15]

MSARREQTLSPALLGSIALHGAVAAVIAFGLPWKQSKPITIGESVPVTIVTNGPTNVRPAEEALEE
QTAQTPEPTPEATPQPPAPTPAPTPTPAPAAPAKPTPAPKPTPKPTPAKPTPQKKADNDFFASL
EASIAKTQKATGKPTANAPKGPARAETSVSARPAMGAATGLSAAALGRLQGEVQDRWNPNCEV
EGGSNVNVRVVFVIGPGGRVVGQPESPGTSSPDPVVKAASDRAIRALFAASPFAYLPSDLYGQK
IALNFNAKQACSR

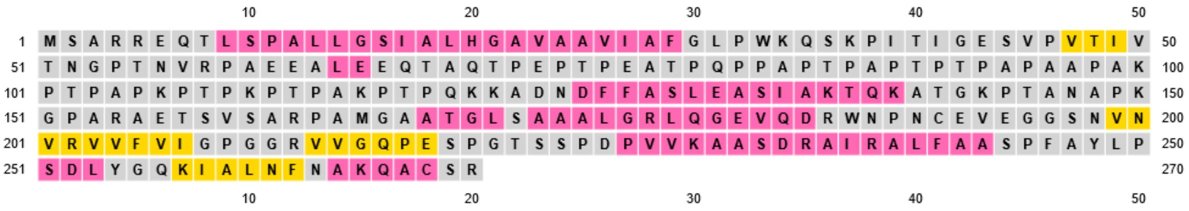

MSARREQTLSPALLGSIALHGAVAAVIAF
GLPWKQSKPITIGESVPVTIVTNGPTNVRPAEEALEEQTAQTPEPTPEATPQPPAPTPAPTPTP APAAPAKPTPAPKPTPKPTPAKPTPQKKADNDFFASLEASIAKTQKATGKPTANAPKGPARA ETSVSARPAMGAATG
LSAAALGRLQGEVQDRWNPNCEVEGGSNVNVRVVFVIGPGGRVVGQPESPGTSSPDPVVKAA SDRAIRALFAASPFAYLPSDLYGQKIALNFNAKQACSR

PPIIPRED:

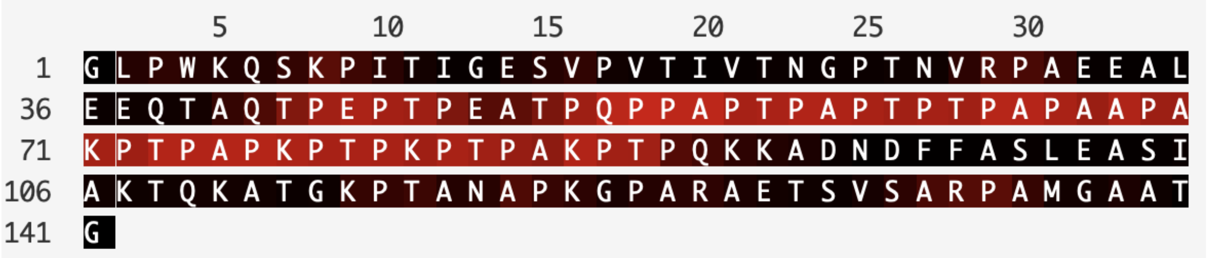

141 Domain II residues

31 Proline residues

20 α-Helix residues

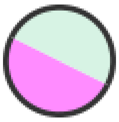

Parvularculales

Parvularcula bermudensis

Locus: 602377-609151

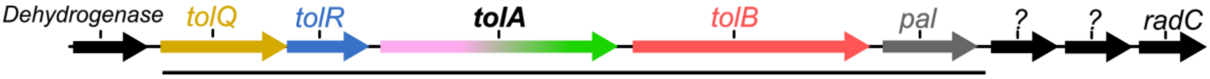

Flanked by an aspartate-semialdehyde deacetylase gene, two short unknown genes, and *radC* involved in DNA repair.

Distal *cpoB* detected. Fold confirmed.

>ADM08744.1 hypothetical protein PB2503_03342 [Parvularcula bermudensis HTCC2503]

MRFPALSLSLLLHGAVIGGLVVTVPRLADDPIYIPPVPVDVLSRAEFDDLVSIPAESATEEPAEREE
PAPLPEPAPEPAPQPEPDPTPEPEPEPEPAPEPEPEPQPAPEPAPEPEPQPEAEPEPQLPEPDP
EPAPPTSAPEPDPVEDLDFDSLSDGLVDLTPDRPRTAPRVVGDTGEGGERDQPQIGPGGQLSIT
EEAALQACVRANRVIDLSARDADQFVVELRIVLNIDQTLAAPIEITNQAAINRSGNPAYQAAARNA
VAAVRACLPLDALDPARYQQWRVINFRFRPGTS

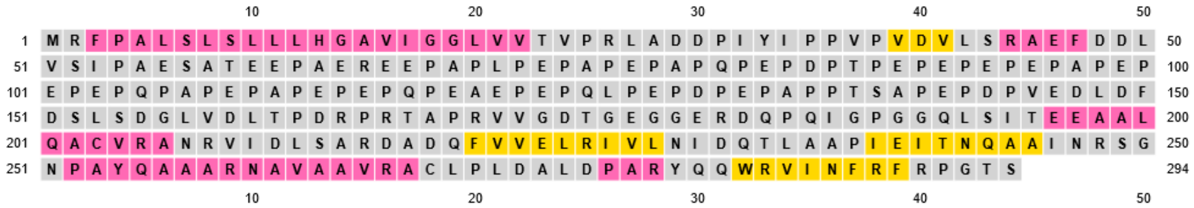

MRFPALSLSLLLHGAVIGGLVV
**TVPRLADDPIYIPPVPVDVLSRAEFDDLVSIPAESATEEPAEREEPAPLPEPAPEPAPQPEPDPT
PEPEPEPEPAPEPEPEPQPAPEPAPEPEPQPEAEPEPQLPEPDPEPAPPTSAPEPDPVEDLDF
DSLSDGLVDLTPDRPRTAPRVVGDTGEGGERDQPQIGPGGQLSI**
TEEAALQACVRANRVIDLSARDADQFVVELRIVLNIDQTLAAPIEITNQAAINRSGNPAYQAAARN
AVAAVRACLPLDALDPARYQQWRVINFRFRPGTS

PPIIPRED:

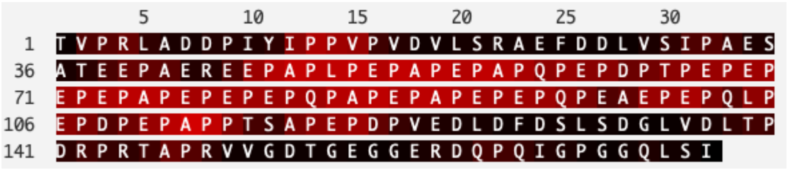

172 Domain II residues

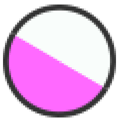

49 Proline residues

4 α-Helix residues

Parvibaculum lavamentivorans

Locus: 2298569- 307012

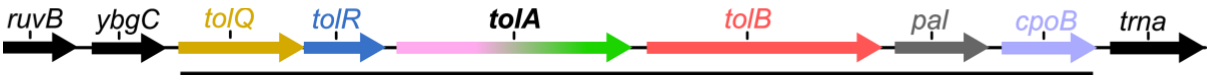

>ABS63735.1 hypothetical protein Plav_2121 [Parvibaculum lavamentivorans DS-1]

MRNSVFLSVAAHFLVVALALVAFPSPEELPLVQTRALPVELVTIDEVTNLKRLQKTEKPKPVEEPE
PPKPVEPEKPKAEPKPQPAPAPPPPPEEKVEPIPDKEAEKKEPPKPVEKKVEKPKPPQKQPEPK
KAFDPNQIAALLDKTPDTSSKPQPQEQAEEEFDSPVTDDPSAALTMSEIDAFKVQMRRCWSVPA
GAPDAEQLIVKIKVYLNQDGSLAAPPELRDKTKLVTGGSYYRAAADSAMRAIRLCQPFKMPAEK
YTSWREIDLNFDPREMLGG

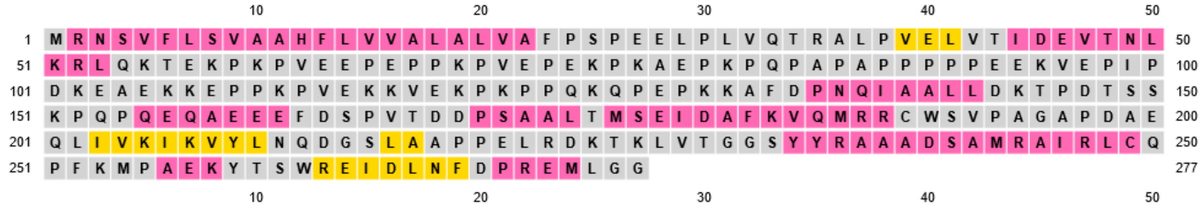

MRNSVFLSVAAHFLVVALALVA
**FPSPEELPLVQTRALPVELVTIDEVTNLKRLQKTEKPKPVEEPEPPKPVEPEKPKAEPKPQPAP
APPPPPEEKVEPIPDKEAEKKEPPKPVEKKVEKPKPPQKQPEPKKAFDPNQIAALLDKTPDTS
SKPQPQEQAEEEFDSPVTDDPSAALTMS**
EIDAFKVQMRRCWSVPAGAPDAEQLIVKIKVYLNQDGSLAAPPELRDKTKLVTGGSYYRAAADS
AMRAIRLCQPFKMPAEKYTSWREIDLNFDPREMLGG

PPIIPRED:

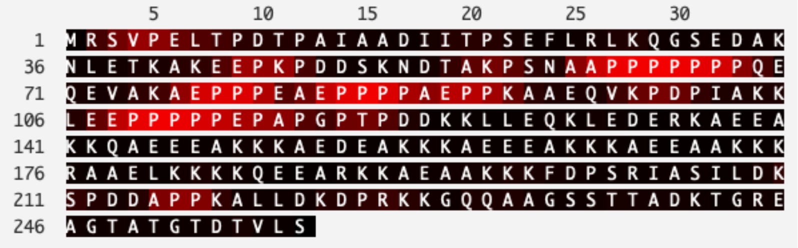

155 Domain II residues

31 Proline residues

32 α-Helix residues

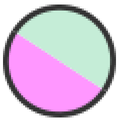

Rhizobiales

Hyphomicrobium denitrificans

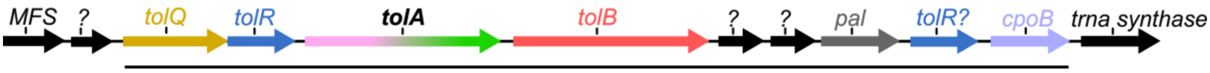

Locus: 2598681- 2608375

Strangely has two PG-binding proteins: *pal* (product) then an ompA-like PGB gene- product looks like TolR.

Flanked by *MFS*, unknown gene and tRNA synthase gene.

>AGK58814.1 protein TolA [Hyphomicrobium denitrificans 1NES1]

MPFGLVLSLTFHAALLGWALFSMRSVPELTPDTPAIAADIITPSEFLRLKQGSEDAKNLETKAKEE
PKPDDSKNDTAKPSNAAPPPPPPPQEQEVAKAEPPPEAEPPPPAEPPKAAEQVKPDPIAKKLEE
PPPPPEPAPGPTPDDKKLLEQKLEDERKAEEAKKQAEEEAKKKAEDEAKKKAEEEAKKKAEEAA
KKKRAAELKKKKQEEARKKAEAAKKKFDPSRIASILDKSPDDAPPKALLDKDPRKKGQQAAGSS
TTADKTGREAGTATGTDTVLSAREQDLLKGMLKSQLNGCWRPPGTGGGEEVPVVELHWEMNP
DGSLAGEPRVTSAPSTTAGQVYTEAALRAVRMCAPFRLPPDKYNAWKIVDWTFDPRQML

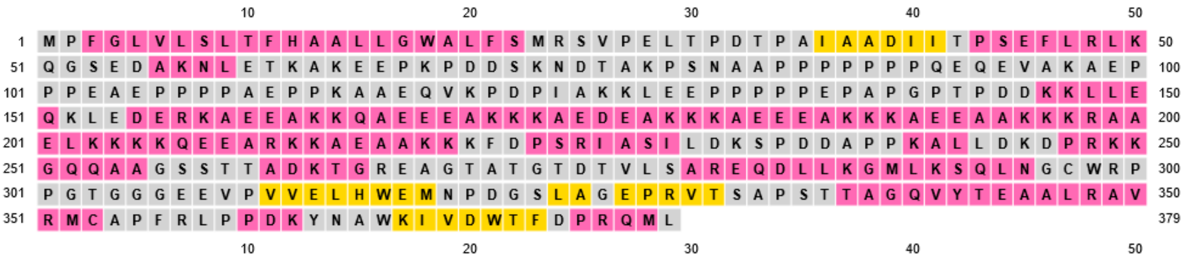

MPFGLVLSLTFHAALLGWALFS
**MRSVPELTPDTPAIAADIITPSEFLRLKQGSEDAKNLETKAKEEPKPDDSKNDTAKPSNAAPPP
PPPPQEQEVAKAEPPPEAEPPPPAEPPKAAEQVKPDPIAKKLEEPPPPPEPAPGPTPDDKKLL
EQKLEDERKAEEAKKQAEEEAKKKAEDEAKKKAEEEAKKKAEEAAKKKRAAELKKKKQEE
ARKKAEAAKKKFDPSRIASILDKSPDDAPPKALLDKDPRKKGQQAAGSSTTADKTGREAGTA
TGTDTVLS**
AREQDLLKGMLKSQLNGCWRPPGTGGGEEVPVVELHWEMNPDGSLAGEPRVTSAPSTTAGQ
VYTEAALRAVRMCAPFRLPPDKYNAWKIVDWTFDPRQML

PPIIPRED:

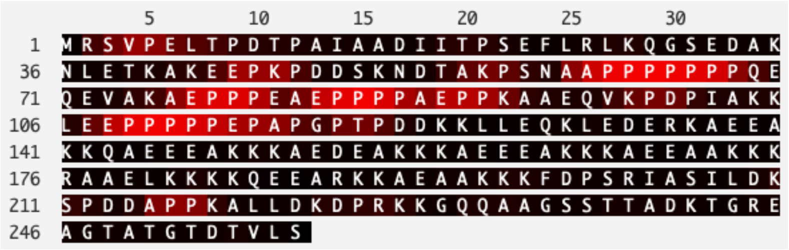

257 Domain II residues

39 Proline residues

107 α-Helix residues

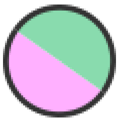

Bartonella clarridgeiae

Locus: 1370924-1376841

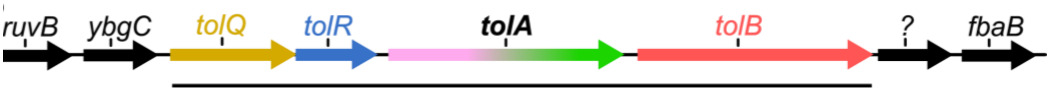

Flanked by *ruvB* and *fbaB*. Strange gene for disordered protein after *tolB.*

Distal *pal* (1358841) but no *cpoB* detected by BLAST.

>CBI76908.1 TolA protein [Bartonella clarridgeiae 73]

MHLRTLCNHEKKQQGIQMEKGSHHSMQKSLVLSFALHFILFSSGMMHFTPSSSFMQQPLEMAP
ITLSSFDQDLAFQEGSSNAPPLENSAPKPTTKPQEKEDARYFGEGTIDNQSPFQPKEQLQHMDT
IPSFFGQQDASEIFESSLKTQELIKTETEALLHTVPEFMKNETKTPPQESELKEQTVPPTALEQIKS
EDPIKKMLEELAQENPNESISTITPLDNIPLPSFKPKTKTVDPSHFQKEKKSPSKREQTIEDILAME
ETHLINRARTQGGGAKRSSEREALGTQKNIGDSAKKAQTLVNLVGSCIQKKLKLVAIGGNLNNRP
VVRLQFYLNNHGYVIGEPKIDPLRGEESQLTIMTQQVYAAVFSCQPYENLPRDQYNLWGQGFDF
NVDPFQETAP

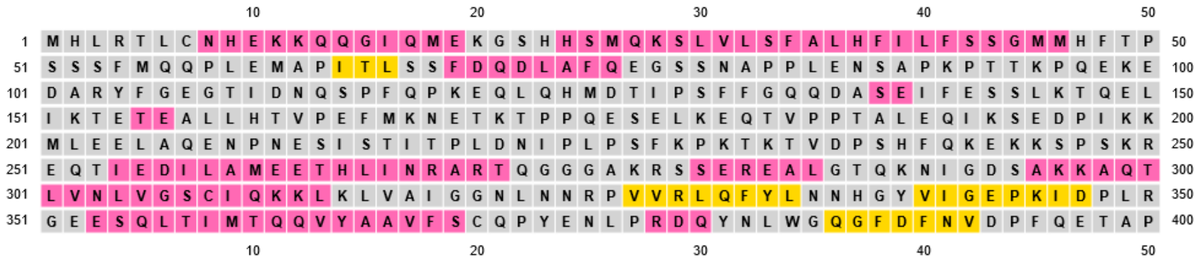

MHLRTLCNHEKKQQGIQMEKGSHHSMQKSLVLSFALHFILFSSGMM
**HFTPSSSFMQQPLEMAPITLSSFDQDLAFQEGSSNAPPLENSAPKPTTKPQEKEDARYFGEGT
IDNQSPFQPKEQLQHMDTIPSFFGQQDASEIFESSLKTQELIKTETEALLHTVPEFMKNETKTPP
QESELKEQTVPPTALEQIKSEDPIKKMLEELAQENPNESISTITPLDNIPLPSFKPKTKTVDPSHF
QKEKKSPSKREQTIEDILAMEETHLINRARTQGGGAKRSSEREALGTQKNIGDSAKKAQT**
LVNLVGSCIQKKLKLVAIGGNLNNRPVVRLQFYLNNHGYVIGEPKIDPLRGEESQLTIMTQQVYAA
VFSCQPYENLPRDQYNLWGQGFDFNVDPFQETAP

PPIIPRED:

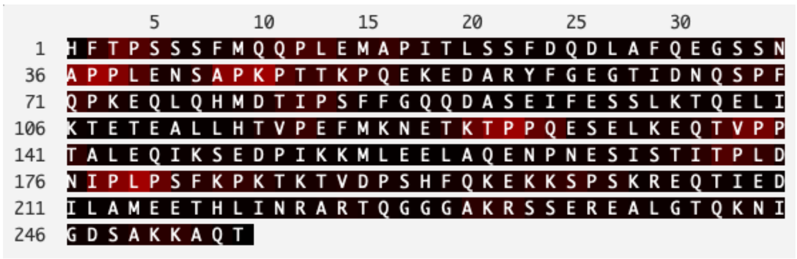

254 Domain II residues

24 Proline residues

42 α-Helix residues

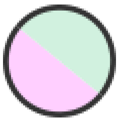

Rhizobium etli

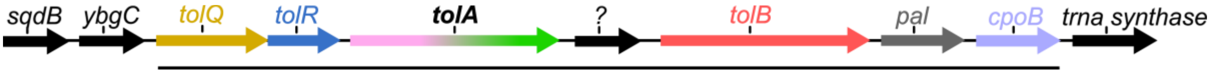

Locus: 3735971-3745393

Flanked by *sqdB* *ybgC* and a tRNA synthase gene. After TolA, before TolB, there is an unknown gene (product).

>ACE92641.1 outer membrane protein [Rhizobium etli CIAT 652]

MKTSVVTSAVLHCLVLTWAMVSLSAPESFKVEDFEAMPVDLVPVESITQMQQGDKKAPKKETS
APVPTTRPPVAQPAENAGDNNVDLKTPPVPNAKPSNSEAAAANSSEKPLPQVDPKPNDVKEVN
KEETEVDQPKEVASIPPPKPVEVTPPKPEEKPPEEQAKPEEPPKPDAEALPDKVPTPVVKPQVK
PPEPQKPAEKPPEKTPDEPKAAEKPTDKKKADKKQEVAKSASSMKSDFNADEISALLNKTDPSA
GGAKRSTQEASLGAKKSNGGSKLSQSEEDAMRSLIEGNWLITPGMEGLSGMVIKVHMKLDRDG
NIIGQPEVESSGGSSSDSTRRALESGAYRAVMKSAPFTTLPKDKYDAWNEFDLNFDPSSMGI

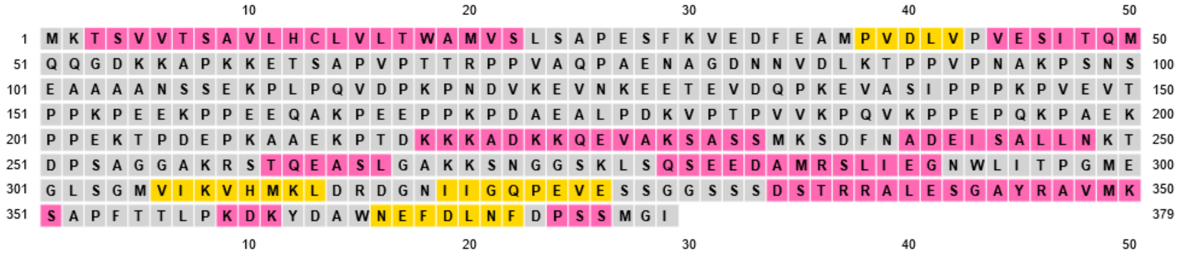

MKTSVVTSAVLHCLVLTWAMVS
**LSAPESFKVEDFEAMPVDLVPVESITQMQQGDKKAPKKETSAPVPTTRPPVAQPAENAGDNN
VDLKTPPVPNAKPSNSEAAAANSSEKPLPQVDPKPNDVKEVNKEETEVDQPKEVASIPPPKPV
EVTPPKPEEKPPEEQAKPEEPPKPDAEALPDKVPTPVVKPQVKPPEPQKPAEKPPEKTPDEP
KAAEKPTDKKKADKKQEVAKSASSMKSDFNADEISALLNKTDPSAGGAKRSTQEASLGAKK
SNGGSKLSQ**
SEEDAMRSLIEGNWLITPGMEGLSGMVIKVHMKLDRDGNIIGQPEVESSGGSSSDSTRRALESG
AYRAVMKSAPFTTLPKDKYDAWNEFDLNFDPSSMGI

PPIIPRED:

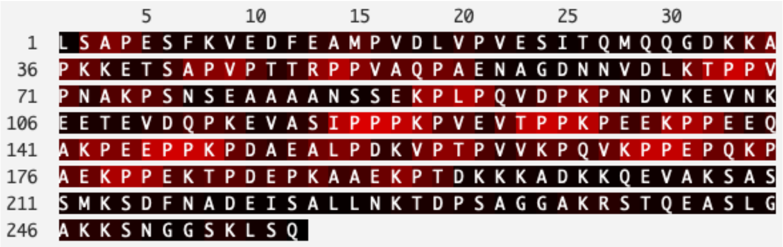

257 Domain II residues

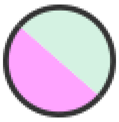

45 Proline residues

39 α-Helix residues

Nitrobacter winogradskyi

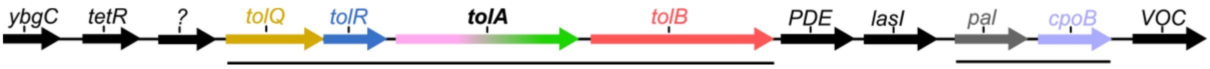

Locus: 2947158-2959234

Flanked by *ybgC*, *tetR* and an unknown gene. Between *tolB* and *pal* there is a diguanylate cyclase gene and *lasI*. After *cpoB* is an oxygen chelation gene.

>ABA05971.1 Cell division and transport-associated protein TolA [Nitrobacter winogradskyi Nb-255]

MRVKVDKTLAASVALHVLVIGWGLVSFSSKAFESVPEESLPVDIISADQLTHVTAGMKTGKKENPKPLVEKVAEPTPPPEDTVGKIDEKKPPVVTDVAPKEQPKPDDKAAAEAAAAAKAEAAEAKAKADAKAQAEAAAKAEAAKAQAEAVAEAKAQAEARAKAEAQAKAKAEARARAKAEAKAKADAKAKAEAKAKAEADAKAQAEAKRAERVFDRSKIAALLDKRDPSRHSVAGDTLNANAALGLAHGKAADNSATWGSMFKSQVERCWKKPYGGLEAQMTEAIFSIKLKRDGTLEATPTAISNPTTPYFRVYQESALRAIIECQPYKLPAAYFDEWKFFEPVFTERKL

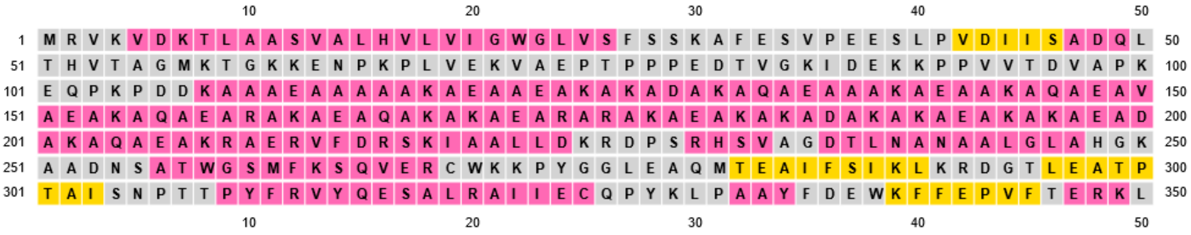

MRVKVDKTLAASVALHVLVIGWGLVS
FSSKAFESVPEESLPVDIISADQLTHVTAGMKTGKKENPKPLVEKVAEPTPPPEDTVGKIDEKKPPVVTDVAPKEQPKPDDKAAAEAAAAAKAEAAEAKAKADAKAQAEAAAKAEAAKAQAEAVAEAKAQAEARAKAEAQAKAKAEARARAKAEAKAKADAKAKAEAKAKAEADAKAQAEAKRAERVFDRSKIAALLDKRDPSRHSVAGDTLNANAALGLAHGK
AADNSATWGSMFKSQVERCWKKPYGGLEAQMTEAIFSIKLKRDGTLEATPTAISNPTTPYFRVYQESALRAIIECQPYKLPAAYFDEWKFFEPVFTERKL

PPIIPRED:

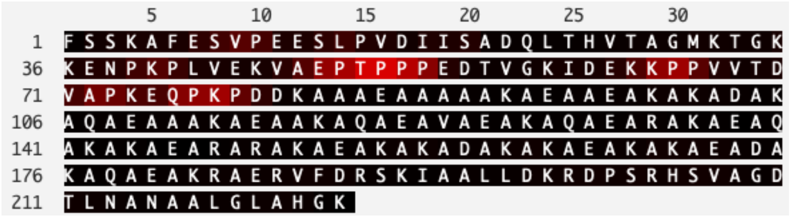

224 Domain II residues

14 Proline residues

136 α-Helix residues

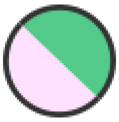

I found *pal* using a BLASTp search using *pal* from *Methylobacterium extorquens*, as *pal* was misannotated here

Methylobacterium extorquens

Split system

Locus: 380462- 388126

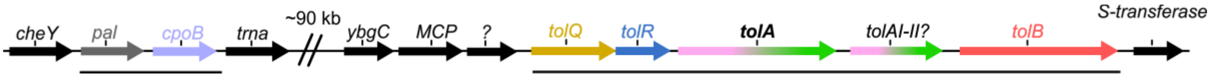

Flanked by *ybgC*, *tar* (a methyl-accepting protein involved in chemotaxis) and an unknown gene and short gene. In the middle of the locus there is a gene after TolA but before TolB that looks like TolAI-II.

pal is found distally (289612-290124)- AF prediction matches Pal. Found next to *cpoB*. These are flanked by *cheY* and a *tRNA*.

>SOR27041.1 membrane anchored protein in TolA-TolQ-TolR complex [Methylorubrum extorquens]

MALRWPTSLTESFRNREPGVWISAAVHAVVLGAALFVVAAPVLPEGEEGVPVEVLTEQQFSELTRGELNAEKPENKPNRADRV
AEKQEQREPENAKTDAPAAPTRTADTRLAAVDAMPLRADTADPVKEEAEAAAAKAEAAKAEAEKAAAAKAAEAKAKAEVKAKA
DAAAKAEAAKAEAAKAAEAKAAAAKAEAEKREELQKLVEHEQAEAEAAAKAAEAKAKARAEAKAKADAKAKAEAKAEAEAEAKA
KAEAAAKAEAEAEHRKQVAEAKADADAKAKAEAAAKAKAKAMADARAKADAEAKARQQAELANRTSPGDSRETQTRAASQST
GSTGREVQRTASLGTASGTAQRLSPSLRGALVGMLQQQIERCYSAPPGATQGVVLPMLDIRLNPDGSLTTEPRVMRAGNNSVD
QSIAQAALRAVRRCAPFKIPAQYAPYYNDWKAINAEFEFSPV

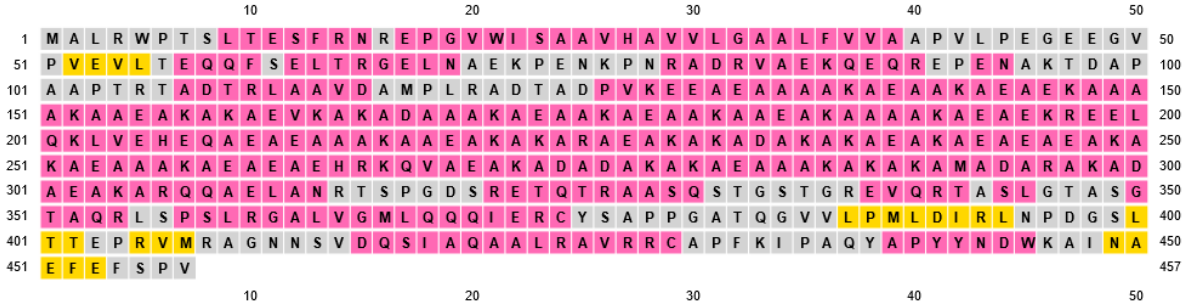

MALRWPTSLTESFRNREPGVWISAAVHAVVLGAALFVVA
**APVLPEGEEGVPVEVLTEQQFSELTRGELNAEKPENKPNRADRVAEKQEQREPENAKTDAPAAPTRTADTRLAAVDAMPLR
ADTADPVKEEAEAAAAKAEAAKAEAEKAAAAKAAEAKAKAEVKAKADAAAKAEAAKAEAAKAAEAKAAAAKAEAEKREE
LQKLVEHEQAEAEAAAKAAEAKAKARAEAKAKADAKAKAEAKAEAEAEAKAKAEAAAKAEAEAEHRKQVAEAKADADAK
AKAEAAAKAKAKAMADARAKADAEAKARQQAELANRTSPGDSRETQTRAASQSTGSTGREVQRTASLGTASGTAQRLSP**
SLRGALVGMLQQQIERCYSAPPGATQGVVLPMLDIRLNPDGSLTTEPRVMRAGNNSVDQSIAQAALRAVRRCAPFKIPAQYAPY
YNDWKAINAEFEFSPV

PPIIPRED:

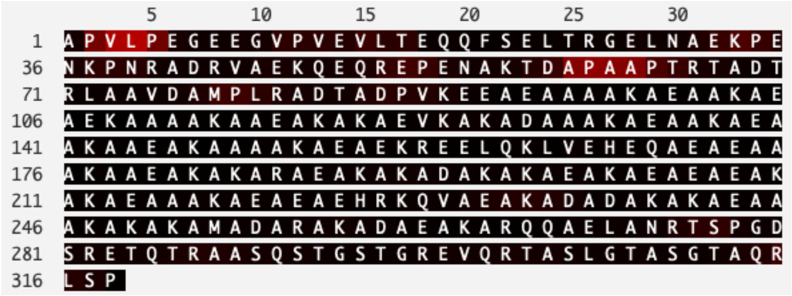

318 Domain II residues

12 Proline residues

246 α-Helix residues

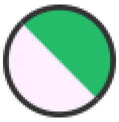

Beijerinckia indica

Split system

Locus:

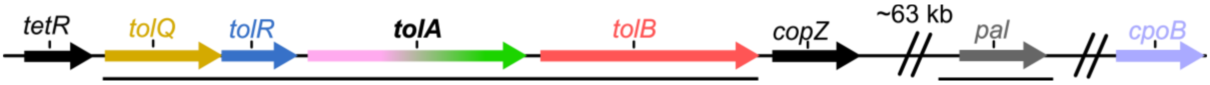

tolQRAB (309554-317574) flanked by *tetR* and *copZ* P-ATPase.

pal (3861310-3861828) is distal, AF confirmed structure. Flanked by *arsC* arsenate reductase and an unknown gene. *cpoB* also distal (4121963)

>ACB93926.1 TolA protein [Beijerinckia indica subsp. indica ATCC 9039]

MRLHALHHPGENFDDADDWDRQETGASAASLVSMIRQSISKKLNDNPGFGVSLTAHAALLLAFVISFS
STPQFPEAVESIPVDMVTTQDLNQIMKGEKTAKTVQSSHRAERVSDLAELHPQPPLAEAKKDVMPPP
RPGKPQPDPGESEKPQEAQRETPTPPKPPERPAQTPPPRPAQEAKLEPPTPPAKPQPPQKAEPKPQ
PVSPEAAEPLPAPRPKAETKPKPEPKSESKPEDKPKPHEAKPNETKPTPKFAPDAVAKLLEQTPAKTE
TKKAEAKPAQRPKSGSEAATEPSKFNAADISKLLSAEAPQHKASTGKTVQQTASLGLPNANAAKMSP
SMWDRLDSILQDQYKQCWSFIGLNKQKYVPEIHVQYAEDGSLIGQPSLLNPPSDPQLRGLADSAMRA
VRRCNPLKIPAQYQPYYDQWKGRIVRFDPEEML

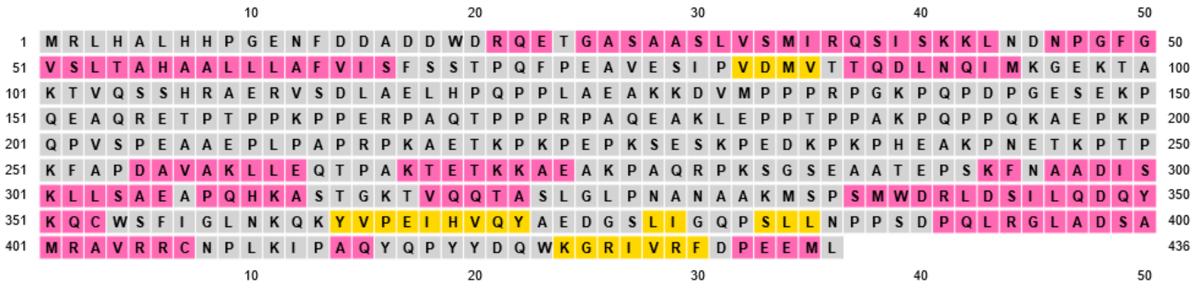

MRLHALHHPGENFDDADDWDRQETGASAASLVSMIRQSISKKLNDNPGFGVSLTAHAALLLAFVIS
**FSSTPQFPEAVESIPVDMVTTQDLNQIMKGEKTAKTVQSSHRAERVSDLAELHPQPPLAEAKKDVM
PPPRPGKPQPDPGESEKPQEAQRETPTPPKPPERPAQTPPPRPAQEAKLEPPTPPAKPQPPQKAE
PKPQPVSPEAAEPLPAPRPKAETKPKPEPKSESKPEDKPKPHEAKPNETKPTPKFAPDAVAKLLE
QTPAKTETKKAEAKPAQRPKSGSEAATEPSKFNAADISKLLSAEAPQHKASTGKTVQQTASLGLP
NANAAKMSP**
SMWDRLDSILQDQYKQCWSFIGLNKQKYVPEIHVQYAEDGSLIGQPSLLNPPSDPQLRGLADSAMRA
VRRCNPLKIPAQYQPYYDQWKGRIVRFDPEEML

PPIIPRED:

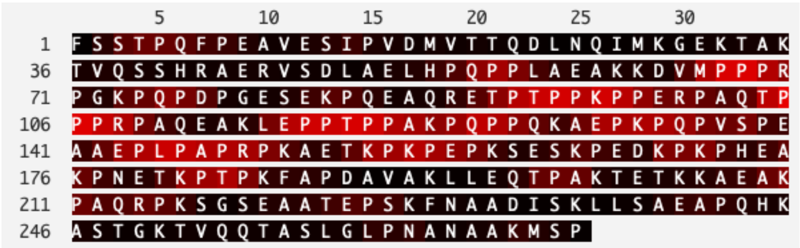

270 Domain II residues

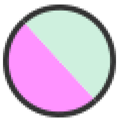

56 Proline residues

47 helix residues

Xanthobacter autotrophicus

Split system

Locus: *tolQRAB* (21805-26507) and *pal*-*cpoB* (46738- 48443)

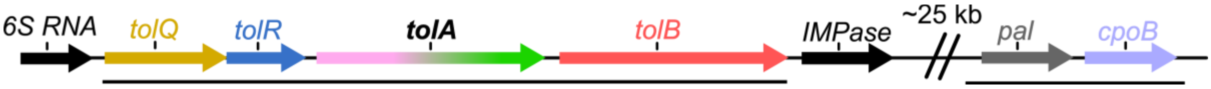

tolQRAB flanked by 6S RNA gene and inositol monophosphatase.

Pal-cpoB flanked by *pspC* and *tilS* tRNA synthetase gene

>TLX42366.1 cell envelope biogenesis protein TolA [Xanthobacter autotrophicus]

MRAGIAASTLLHAGVLVLTLVSFTGAKPFEPMPESLPVDVVSISEYTKLTKGARNAPKLDKPKQIA
EKVGDPTPVEDAKLKASEKAPVEATAPPPPPPPPPPKVEPLPKDAAPAKVEPTPPKEQAELAPK
PEQKKQEQPKDEAKTEAAAAPLPPRKPAPPKEQPKPVEANATPSEFNTDQIKQLLDKRAPSRQV
ASAEQVSTTSSLGSPRGEGQQLTASEIDAFRRRVMECWSTDGGNLDRNIRVDVNIYLNKDGTV
AGAPQIEPGQPGSGQPAFQAYAMNGIRAIMACQPYKMFRPETYANWKTLPITLNDKLFSR

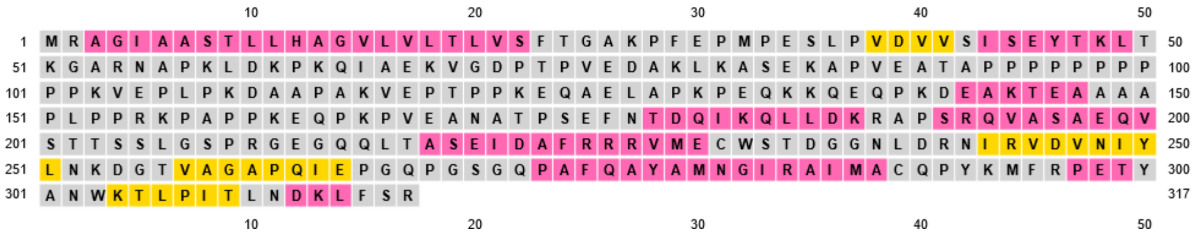

MRAGIAASTLLHAGVLVLTLVS
**FTGAKPFEPMPESLPVDVVSISEYTKLTKGARNAPKLDKPKQIAEKVGDPTPVEDAKLKASEK
APVEATAPPPPPPPPPPKVEPLPKDAAPAKVEPTPPKEQAELAPKPEQKKQEQPKDEAKTEA
AAAPLPPRKPAPPKEQPKPVEANATPSEFNTDQIKQLLDKRAPSRQVASAEQVSTTSSLGSP
RGEGQQLT**
ASEIDAFRRRVMECWSTDGGNLDRNIRVDVNIYLNKDGTVAGAPQIEPGQPGSGQPAFQAYAM
NGIRAIMACQPYKMFRPETYANWKTLPITLNDKLFSR

PPIIPRED:

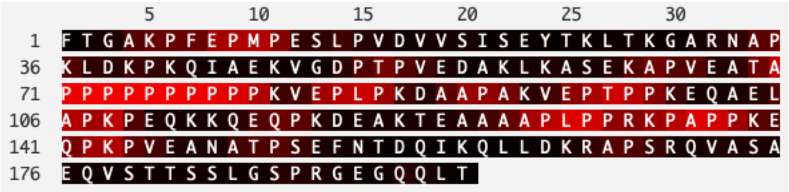

195 Domain II residues

39 Proline residues

33 α-Helix residues

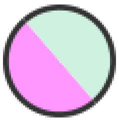

δ-Proteobacteria

Syntrophobacterales

Syntrophobacter fumaroxidans

Locus: 921088- 929333

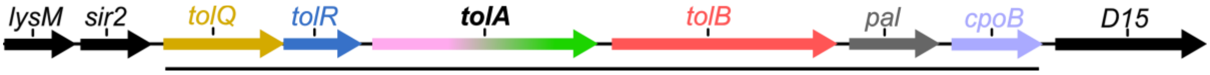

Flanked by LysM, *sir2* protein deacetylase and *D15* Surface antigen

>ABK16452.1 TonB family protein [Syntrophobacter fumaroxidans MPOB]

MESLTCTPRRLSKGEFLTGVGGSILVHIGVIVLAVAVSWHIPKKGVAPAFTTVNLVSMQDVAGAA
PKKGLQTKGAEGPRTQEAAKPVPTAKPGPMVPIRRLRMEDPVKKTEPEIKKIESRDIPKVAETAP
ASMSVEKSLEKLIPKPKAPAKPAQAAQASSQEPSAAPQEQRQARVKPSSTQENPAQGNPRGAV
DGHAGGTADTTARGNPEASGKADGGRVASALLALYANQVKNAINSQWSILDSLKSTRLEARLLI
AVSRDGRVLDLQVEKPSGNGLFDEACVRAVRKAAPLPPIPETYTSPRIEFVINFRPEGLS

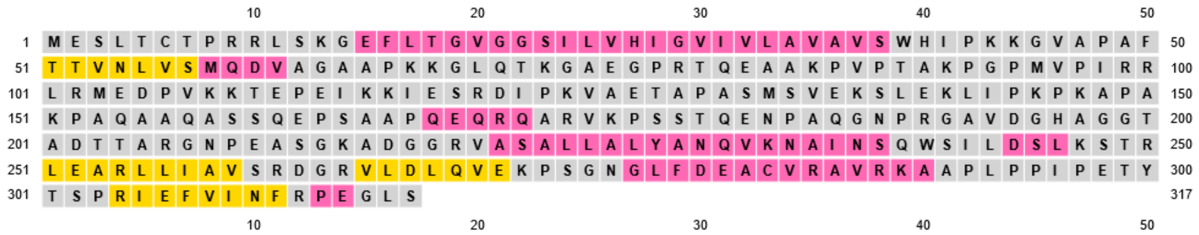

MESLTCTPRRLSKGEFLTGVGGSILVHIGVIVLAVAVS
**WHIPKKGVAPAFTTVNLVSMQDVAGAAPKKGLQTKGAEGPRTQEAAKPVPTAKPGPMVPIRR
LRMEDPVKKTEPEIKKIESRDIPKVAETAPASMSVEKSLEKLIPKPKAPAKPAQAAQASSQEPS
AAPQEQRQARVKPSSTQENPAQGNPRGAVDGHAGGTADTTARGNPEASGKADG**
GRVASALLALYANQVKNAINSQWSILDSLKSTRLEARLLIAVSRDGRVLDLQVEKPSGNGLFDEA
CVRAVRKAAPLPPIPETYTSPRIEFVINFRPEGLS

PPIIPRED:

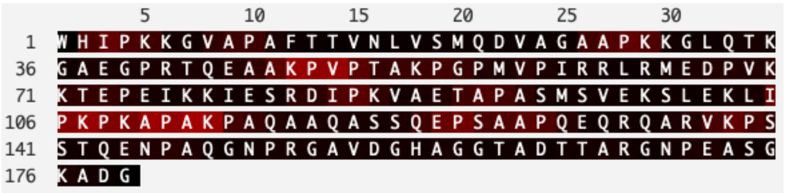

179 Domain II residues

23 Proline residues

9 α-Helix residues

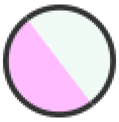

Desulfobacca acetoxidans

Split system

Locus: 2374137-2380678

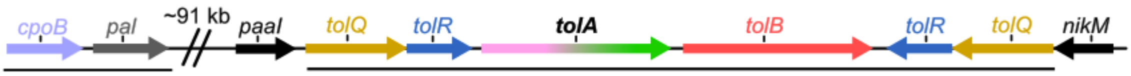

Flanked by *paal*, a thioesterase, and *nikM*, a nickel transporter

cpoB-***Pal*** **is located distally** (2464491..2466105)- (AF Prediction link)

>AEB09961.1 hypothetical protein Desac_2132 [Desulfobacca acetoxidans DSM 11109]

MNNGGLPDPSPSDRLGRMLLLSMAFHLVLFGVLWYFNAKSPKVKPLRPTYTVDLVNMPLPAPA
PTIVKPESSPAGETVRPNREVREKPGPAIGLAPEPKKTQPDRMPPKVEKKPLTPLKPARPVEAE
PAARAAAKPAQPAVKPIAPAKPVAKVETPPKTVAKPLAPPKPPIKTAEPVKEVPKPTLASKPKEIV
PPTPPVARSKPTPKGRGEAKATTESVKPVAEPAKKSGSPNSASGAADAKARDREIAQEISRVQS
KANRSSRDQTIQDSIRGLEKGLAAKSREQEYQEAVRQAAAKVSAGKVSAKVSGAGSGKGGSAG
GGALGSAQAAAYGSKVSQMVKRHWQPNCLQRSTMHHLKAVIVIRIQSDGTITASWFEKESGDR
LFDQSAMSAVTRSNPLPALPPGREQLEIGITFTPEWKASS

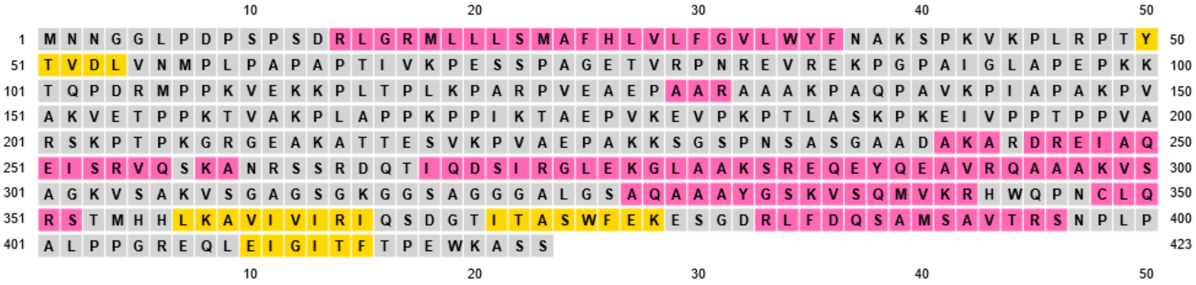

MNNGGLPDPSPSDRLGRMLLLSMAFHLVLFGVLWYF
**NAKSPKVKPLRPTYTVDLVNMPLPAPAPTIVKPESSPAGETVRPNREVREKPGPAIGLAPEPK
KTQPDRMPPKVEKKPLTPLKPARPVEAEPAARAAAKPAQPAVKPIAPAKPVAKVETPPKTVA
KPLAPPKPPIKTAEPVKEVPKPTLASKPKEIVPPTPPVARSKPTPKGRGEAKATTESVKPVAEP
AKKSGSPNSASGAADAKARDREIAQEISRVQSKANRSSRDQTIQDSIRGLEKGLAAKSREQEY
QEAVRQAAAKVSAGKVSAKVSGAGSGKGGSAGGGA**
LGSAQAAAYGSKVSQMVKRHWQPNCLQRSTMHHLKAVIVIRIQSDGTITASWFEKESGDRLFD
QSAMSAVTRSNPLPALPPGREQLEIGITFTPEWKASS

PPIIPRED:

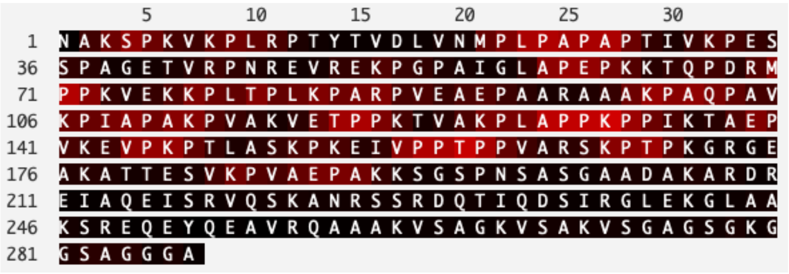

287 Domain II residues

47 Proline residues

58 α-Helix residues

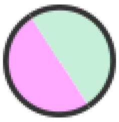

Desulfarculales

Desulfarculus baarsii

Likely a split system.

Locus: 2675968- 2683648

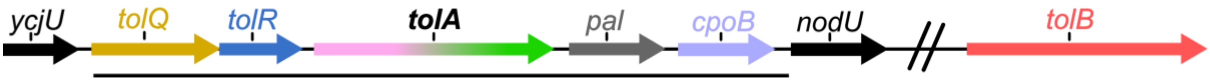

cpoB- and pal found without *tolB*.

Operon flanked by *ycjU*, a HAD-family hydrolase, and *nodU*, a carbamoyl transferase.

No *tolB* found in this accession, but *tolB* found via tBLASTn, suggesting distal presence. Fold checked by AF3, strange NTD.

Not annotated in this genome.

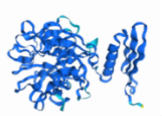

Description	Scientific Name	Max Score	Total Score	Query Cover	E value	Per. Ident	Acc. Len	Accession
Desulfarculus baarsii DSM 2075, complete genome	Desulfarculus b...	688	688	100%	0.0	99.70%	3655731	CP002085.1

>ADK85792.1 TonB family protein [Desulfarculus baarsii DSM 2075]

MASAAVEKNLEGHDQLGWALAVSLAVHVVVAAAVILWPASQPDRQFFSPAYQVALVSGAALPS
SGPPATAKKTAPPKKEMEKPKPAPKPEVKPKPDVKPKPAPPKPEPKPEPKEAIGSKKTVEPKRV
KREVKKAEPEPSYEDVLDQRLKKIKSKAESRRQSEQLDDALSAIESKVQAGGGATGPSGAVAM
PGGGELSTRFMLYYTEVWERIRRAWVLPEALAGDTSGAMAVVALRINRDGSLNKFWLEESSGN
VRLDQSALRAVERAAPFPPLPADLLGPYHEVGLRFRPEDAGQ

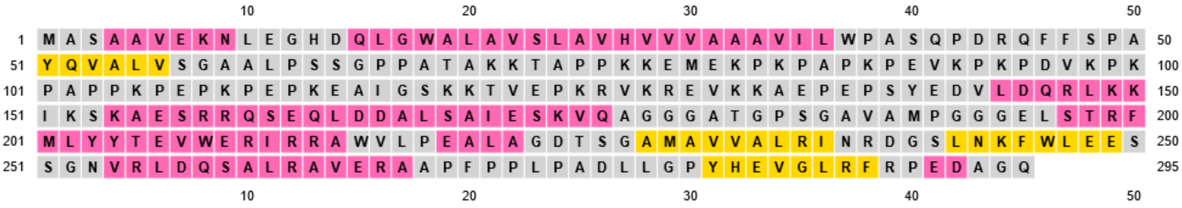

MASAAVEKNLEGHDQLGWALAVSLAVHVVVAAAVIL
**WPASQPDRQFFSPAYQVALVSGAALPSSGPPATAKKTAPPKKEMEKPKPAPKPEVKPKPDV
KPKPAPPKPEPKPEPKEAIGSKKTVEPKRVKREVKKAEPEPSYEDVLDQRLKKIKSKAESRRQ
SEQLDDALSAIESKVQAGGGATGPSGAVAMPGGGE**
LSTRFMLYYTEVWERIRRAWVLPEALAGDTSGAMAVVALRINRDGSLNKFWLEESSGNVRLDQ
SALRAVERAAPFPPLPADLLGPYHEVGLRFRPEDAGQ

PPIIPRED:

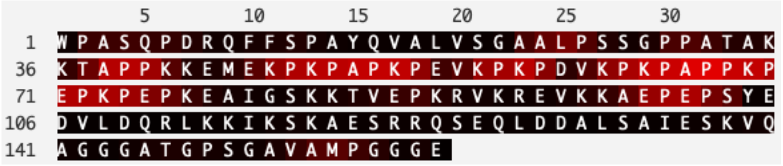

159 Domain II residues

27 Proline residues

30 α-Helix residues

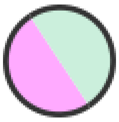

Desulfobacterales

Desulfocapsa sulfexigens

Locus: 3303676-3309846

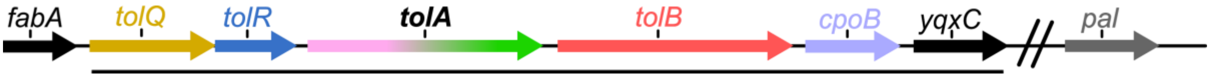

Flanked by *fabA*, an acyl-carrier dehydratase, and *yqxC*, encoding a ftsJ-like/haemolysin A methyltransferase

pal distal (multiple candidates- one, **two**, three).

>AGF79510.1 TonB family protein [Desulfocapsa sulfexigens DSM 10523]

MTSSANSWKLPFNLAVLSHVLILASAIILPKYFHKKPLIQEFLSVDLVNIAAPLPSTPQPTPAPPQIK
QEVKPSSSPPPAERKKTAPIVPIRPTVTKEVIPSPVEAISIQPLKRKVKVKIPKNTASETNRQRDRE
RERRQLLEEARRQKALADAEAAAANDAVKALKQLLQADSVTSATQTATQPTTTRSGGNSNTAIE
NQYIATIGSSLNQHWALPEIKPWNPDLSATVIIHIAKDGKIINHRFEKRSGDSVYDQFVSRTIQDAN
PLPPIPGAMKVSDFTIGLRFTPGQIR

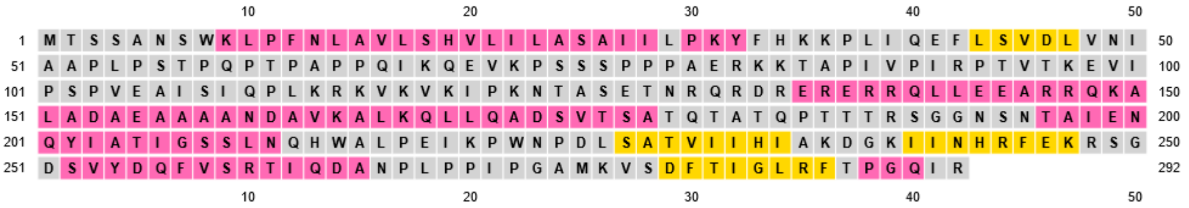

MTSSANSWKLPFNLAVLSHVLILASAII
**LPKYFHKKPLIQEFLSVDLVNIAAPLPSTPQPTPAPPQIKQEVKPSSSPPPAERKKTAPIVPIRPT
VTKEVIPSPVEAISIQPLKRKVKVKIPKNTASETNRQRDRERERRQLLEEARRQKALADAEAAA
ANDAVKALKQLLQADSVTSATQTATQPTTTRSGG**
NSNTAIENQYIATIGSSLNQHWALPEIKPWNPDLSATVIIHIAKDGKIINHRFEKRSGDSVYDQFVS
RTIQDANPLPPIPGAMKVSDFTIGLRFTPGQIR

PPIIPRED:

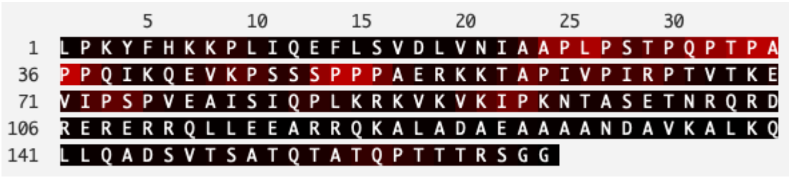

164 Domain II residues

21 Proline residues

47 helix

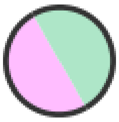

Desulfobacterium autotrophicum

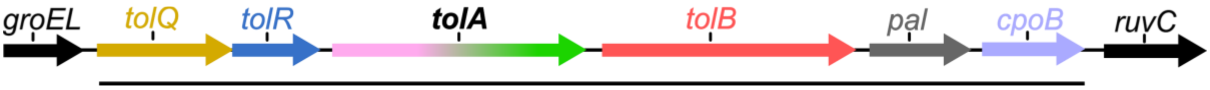

Locus: 3469031-3476526

Flanked by *groEL* and *ruvC*. *tolB* mislabelled "*algW*". CpoB mislabelled aspartate hydrolase.

>ACN16140.1 hypothetical protein HRM2_30570 [Desulforapulum autotrophicum HRM2]

MDAFYPSSSPQGADRLSSGSDESGFRGVWGLTLCLCSVLVHIALAWGLFFVQSIPPKRVLPLVI
QVDLVSLSLPGEVSATLGDAVEADVKEEPVIQPPPKPAAVKTPVKQTVTVPERVVHPPEPESVA
EKITEPVKAPEPVQAAEPDRKLALKKKTFKADKVMESARKSIAKKVETSTPSADNTLERAFSRLE
KAVEAQAGSRPVTGVTGVTGVTGVTGAGAKGDKTELTAIDLYNVELMYRIRQNWAFNERLANA
EQGIRAVVLIKILQNGQIRDVWFETRSGNRYLDESALKAVKKSNPLPPLPKGYVTYDLGLQFTPS
GLK

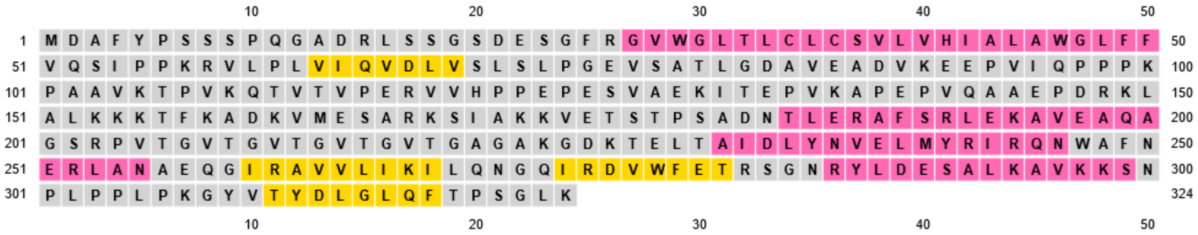

MDAFYPSSSPQGADRLSSGSDESGFRGVWGLTLCLCSVLVHIALAWGLFF
**VQSIPPKRVLPLVIQVDLVSLSLPGEVSATLGDAVEADVKEEPVIQPPPKPAAVKTPVKQTVTV
PERVVHPPEPESVAEKITEPVKAPEPVQAAEPDRKLALKKKTFKADKVMESARKSIAKKVETS
TPSADNTLERAFSRLEKAVEAQAGSRPVTGVTGVTGVTGVTGAGAKG**
DKTELTAIDLYNVELMYRIRQNWAFNERLANAEQGIRAVVLIKILQNGQIRDVWFETRSGNRYLD
ESALKAVKKSNPLPPLPKGYVTYDLGLQFTPSGLK

PPIIPRED:

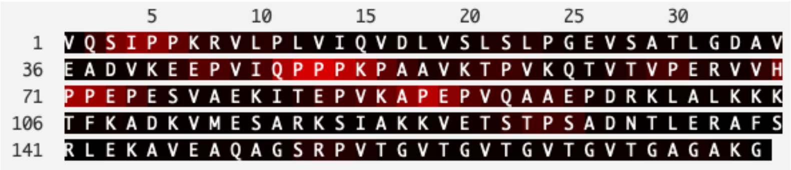

174 Domain II residues

20 Proline residues

17 α-helix residues

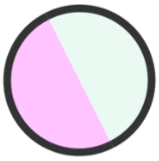

Desulfovibrionales

Desulfohalobium retbaense

Locus: 1833562- 1839944

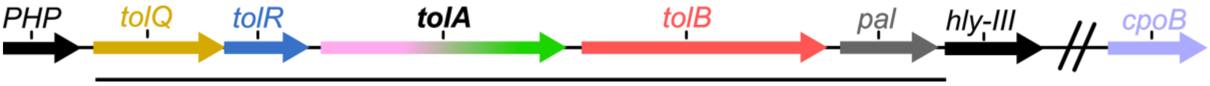

Flanked by PHP phosphoesterase and a haemolysin III channel protein

cpoB (1306085) detected distally via tBLASTn.

>ACV68870.1 TonB family protein [Desulfohalobium retbaense DSM 5692]

MRLISILASLVLHATIVLLGVTQVFFPSSKRIDLDKQIYEVNLVQAPAPSKPAKKMPEPKAESPSAP
EPKKTPAPQPKKTKSAPAPAKKEPAAAKKISAKKAPQPKKSAPKKAEKAQPKPKPKAAQSQPTP
DSVLKDALSDVAQRAEDESGEETMQALRSEVESLRASLQQQAGDAAAREGARAGAVEVYAAL
VEQRVKAQWRYAALGSSRQLRARVVVTISAQGTITGVQLQDASGNEGFDRSVLRAVEDTESLP
PPPGKKLRQISITFNLQAKG

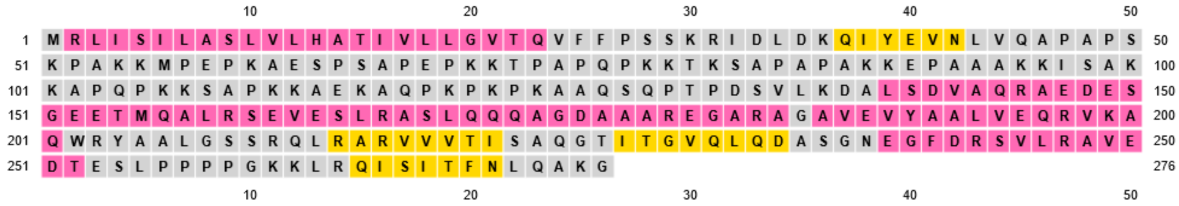

MRLISILASLVLHATIVLLGVTQ
**VFFPSSKRIDLDKQIYEVNLVQAPAPSKPAKKMPEPKAESPSAPEPKKTPAPQPKKTKSAPAP
AKKEPAAAKKISAKKAPQPKKSAPKKAEKAQPKPKPKAAQSQPTPDSVLKDALSDVAQRAE
DESGEETMQALRSEVESLRASLQQQAGDA**
AAREGARAGAVEVYAALVEQRVKAQWRYAALGSSRQLRARVVVTISAQGTITGVQLQDASGNE
GFDRSVLRAVEDTESLPPPPGKKLRQISITFNLQAKG

PPIIPRED:

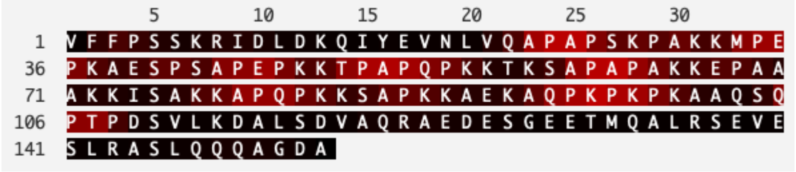

153 Domain II residues

23 Proline residues

38 α-helix residues

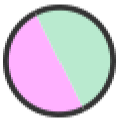

Desulfovibrio vulgaris

Locus: 3238096- 3247179

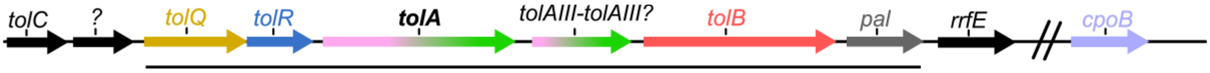

Features an additional TolA between before TolB, predicted to be a double-repeat of TolAIII

Flanked by TolC, a disordered-protein gene, and *rrfE*

Distal *cpoB* (2003452) detected via BLAST.

>AAS97572.1 tonB protein, putative [Nitratidesulfovibrio vulgaris str. Hildenborough]

MRATSFLLSLGLHCSVLLLVIFWPDSAPIRLDQPVYQVSLVSLGDPGGNRVPNALPGPAGPPAQAKAEPKPTPGPEKPDAPAIAAPKAEK
KPEPKPEPKPEPKPQPKPEPKPEPKVEPKPEPKAQPKPEPDAKAISEKKKDEPKKPEPETSKDAKDTKKAVAGKDDAKDKAKTEGKPAP
SKDDILKQALADAQGKAGSTGTAQKSSGTGGKTAGGSVNNALAEMQKMAAAGGGGGGGGGEGHGSGGGGVGDVYIGQVMAIVRQNW
EFPQLASRQDLQVRVRVTVDASGRIADAKLEGSSGRPDFDASALKALHKTQMLPPPPKEEFRDLLLVFNLNDMMGKR

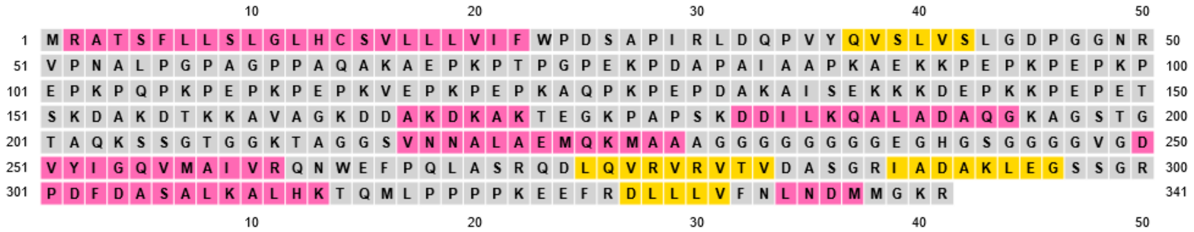

MRATSFLLSLGLHCSVLLLVIF
**WPDSAPIRLDQPVYQVSLVSLGDPGGNRVPNALPGPAGPPAQAKAEPKPTPGPEKPDAPAIAAPKAEKKPEPKPEPKPEPKPQPKPE
PKPEPKVEPKPEPKAQPKPEPDAKAISEKKKDEPKKPEPETSKDAKDTKKAVAGKDDAKDKAKTEGKPAPSKDDILKQALADAQGKA
GSTGTAQKSSGTGGKTAGGSVNNALAEMQKMAAAGGGGGGGGGEGH**
GSGGGGVGDVYIGQVMAIVRQNWEFPQLASRQDLQVRVRVTVDASGRIADAKLEGSSGRPDFDASALKALHKTQMLPPPPKEEFRDLL
LVFNLNDMMGKR

PPIIPRED:

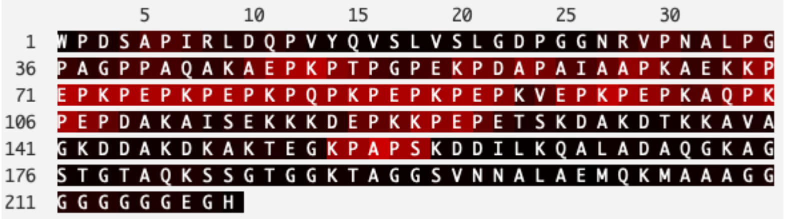

219 Domain II residues

39 Proline residues

32 α-helix residues

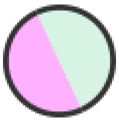

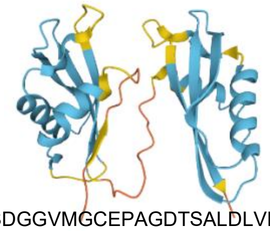

Second "TolA":

>AAS97573.1

MAEDVLSAADTMGGYGGRVLQRIAPHWRAPAGPAGRMVQVRVRIASDGGVMGCEPAGDTSALDLVDAACAAVARAGRMPVPDYGMS
GAVYLSFMTGVDAGQPAPQQGGDPYAAQVMAAVRKHWSPPPVQGEHLVRVRVRVGDDGKVLDYAIDTASGVAEVDAAALRAVSQAG
AMPVPPAGAARDIVLSFTVRGGQ

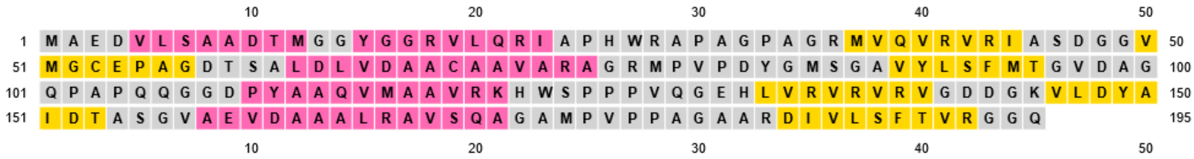

Desulfomicrobium baculatum

Locus: 657796-665006

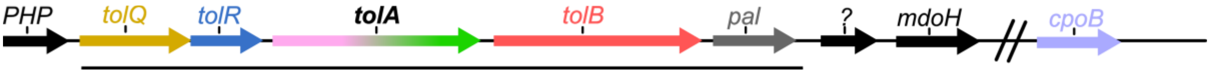

Flanked by a histidiniol phosphate phosphodiesterase (PHP) and a broken gene followed by *mdoH* glucosyltransferase

Distal *cpoB* (548033)

>ACU88711.1 TonB family protein [Desulfomicrobium baculatum DSM 4028]

MFNSLRHLSWVFSIVLHLVVLLGGAYVSTDTHIKLNLNKRMYEVDLVGPPNKGKPGAKSARKKA
PEVAEAPQKPEAKDAKTVKAPDEPKKPEKKAAPSETAKAIPSDTVNATKVAEAKPEEPKKPEPK
KEEPKKEEPKKEEPKKTAKEEPKSEPKAEKKPTKEEILAQALGEATKVAKSSTKSGADGAAKGT
ATSGSKDALADALADLGREVSGRGTRGDGTAEDGDGEGVSSGSLDQYYATQVIRAIRQNWRF
PRLSNVVLATTVELKVNKAGDILSSRMLNGSGRSDFDASVMRAIEDTKKLPPLPETLDATLAITFY
NTEN

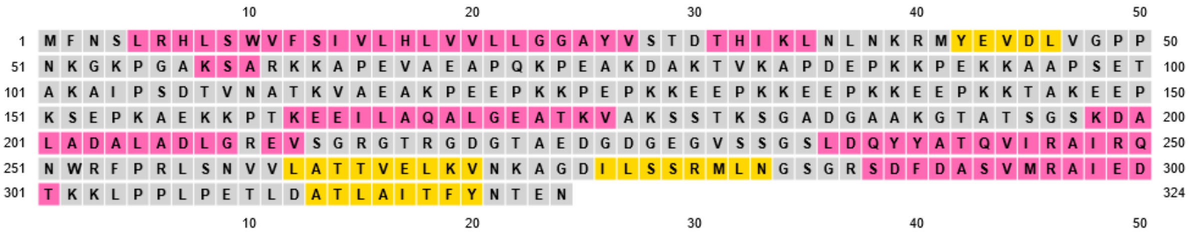

MFNSLRHLSWVFSIVLHLVVLLGGAYV
**STDTHIKLNLNKRMYEVDLVGPPNKGKPGAKSARKKAPEVAEAPQKPEAKDAKTVKAPDEPK
KPEKKAAPSETAKAIPSDTVNATKVAEAKPEEPKKPEPKKEEPKKEEPKKEEPKKTAKEEPKS
EPKAEKKPTKEEILAQALGEATKVAKSSTKSGADGAAKGTATSGSKDALADALADLGREVSG
RGTRGDGTAE**
DGDGEGVSSGSLDQYYATQVIRAIRQNWRFPRLSNVVLATTVELKVNKAGDILSSRMLNGSGRS
DFDASVMRAIEDTKKLPPLPETLDATLAITFYNTEN

PPIIPRED:

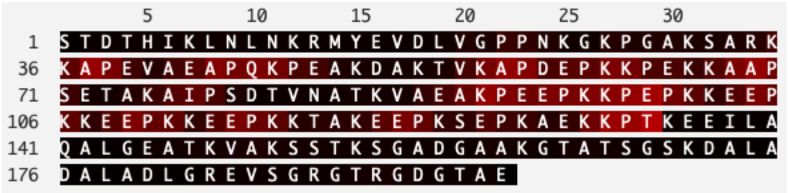

197 Domain II residues

21 Proline residues

37 α-Helix residues

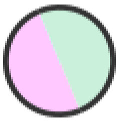

Bacteriovoracles

Geobacter lovleyi

Locus: 3729213- 3735379

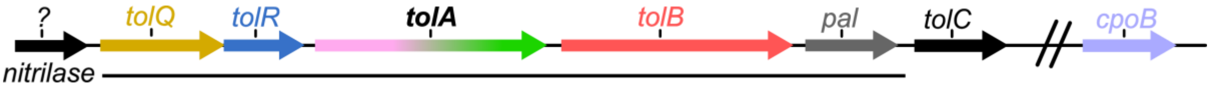

Flanked by a nitrilase/hydratase and *tolC*, an OM efflux pump

Distal *cpoB* (848527)

>ACD97174.1 TonB family protein [Trichlorobacter lovleyi SZ]

MYQLAARKDPGLGVTTALSVAFHAAAFAFLVWWQQMIPGQGPVQTTYYVDIVNLPVADPRAGS
PTQTGNEEKSAPPPPAPPVMTSPAAPTKPVPGKRPSPAPVAESAAFQERMAKLEGKVDAQRQ
AAAFETLRKKVAARGRVGMPRGTGTEAGSDYTAYLHSRLKDAFRETISFQSKNPFVMVRLTIDG
DGRIIRTRFEKSSGDKVFELSVTRAITLAEQTIVPPPGRTVFEGAFVFKPQGVSQQ

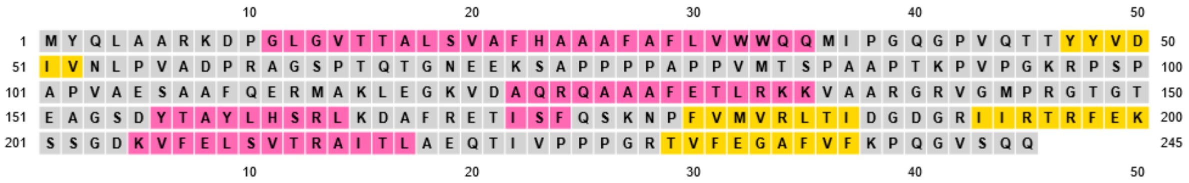

MYQLAARKDPGLGVTTALSVAFHAAAFAFLVWWQQ
**MIPGQGPVQTTYYVDIVNLPVADPRAGSPTQTGNEEKSAPPPPAPPVMTSPAAPTKPVPGKRP
SPAPVAESAAFQERMAKLEGKVDAQRQAAAFETLRKKVAARGRVGMP**
RGTGTEAGSDYTAYLHSRLKDAFRETISFQSKNPFVMVRLTIDGDGRIIRTRFEKSSGDKVFELS
VTRAITLAEQTIVPPPGRTVFEGAFVFKPQGVSQQ

PPIIPRED:

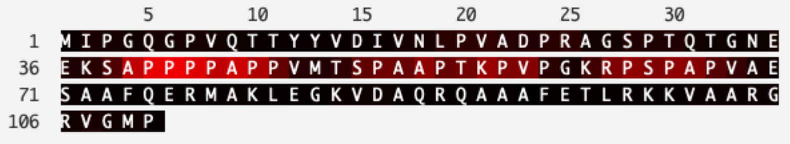

110 Domain II residues

19 Proline residues

14 α-Helix residues

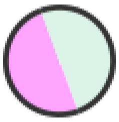

Pelobacter carbinolicus

Locus: 3480375-3489971

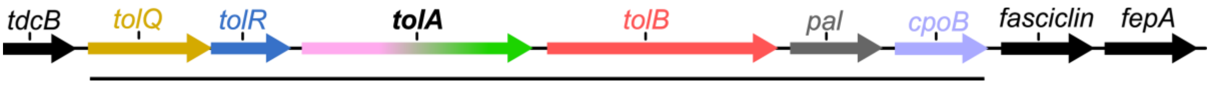

Flanked by *tdcB* (thr/ser dehydratase/deaminase), fasciclin extracellular adhesion homologue (*fas1*?) then FepA, a TBDT

>ABA90211.1 periplasmic energy transduction protein, TonB-related protein [Syntrophotalea carbinolica DSM 2380]

MTTPPVPPRNEPSLFWLLALSLALHAVVFVLFSGVFFASRRIEPRPVYYVDLSKMPVLNPQAGR
PDGGPAPKVKKTSRPKATKPKPAAAAPKAKAKPAPPKKTAVASKPAPTKPVASKTSTPSAKART
SPAPKATAPSHNYQSVRQKLAAMREKQQREQELAALKNKIAALSGDNAGTSGGTGSGAPLGM
PDGSGDEIGVDLQTWMQAYLKQNWTLSKYQVTRRDLTATVHLAFNAEGSLTSYRIVKPSGDAT
FDDSVKKAVLKARALPRKPGRTLQLDVVFNLKDLMD

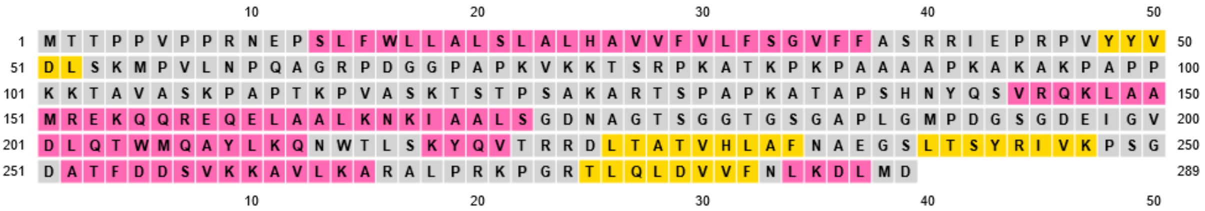

MTTPPVPPRNEPSLFWLLALSLALHAVVFVLFSGVFF
**ASRRIEPRPVYYVDLSKMPVLNPQAGRPDGGPAPKVKKTSRPKATKPKPAAAAPKAKAKPAP
PKKTAVASKPAPTKPVASKTSTPSAKARTSPAPKATAPSHNYQSVRQKLAAMREKQQREQE
LAALKNKIAALSGDNAGTSGGTGSGAPLG**
MPDGSGDEIGVDLQTWMQAYLKQNWTLSKYQVTRRDLTATVHLAFNAEGSLTSYRIVKPSGDA
TFDDSVKKAVLKARALPRKPGRTLQLDVVFNLKDLMD

PPIIPRED:

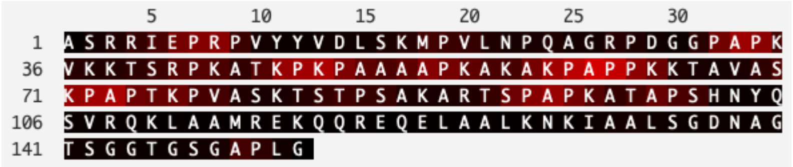

152 Domain II residues

22 Proline residues

29 α-helix residues

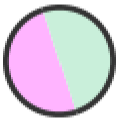

Desulfurellales

Desulfurella multipotens

Locus: 1- 4035

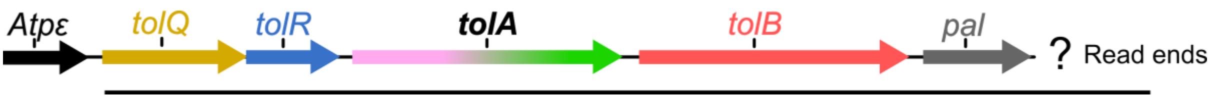

Unclear what comes after Pal but operon confirmed. CpoB could be next but unknown.

cpoB found via BLAST

>SDC92270.1 colicin import membrane protein [Desulfurella multipotens]

MKLASFLLSLFIHIIIIGLLFLLFKSVPTHTMPTYTVSLLTPGEGRAGHQHTIQPLTTSPKPIPHPATT
LPPKINKEIQKTQEQIPPKPQNIEEHKKVIEKPQTELKKVEKEVPKPKVNQKEIQENIQNKIQQLKA
KALQEKITQLKEAMLENKIREIAQQLRENSQTQSGVGNIKAQGSGVGNQDKLFSDYLSVVQGIIH
SNWFVDQNLLPNNKLVTRVKITIAPNGKIISVSIVKSSGNPYYDRTVITAINNSTLPPVPKKYLNNR
NTLDLILNFFIKD

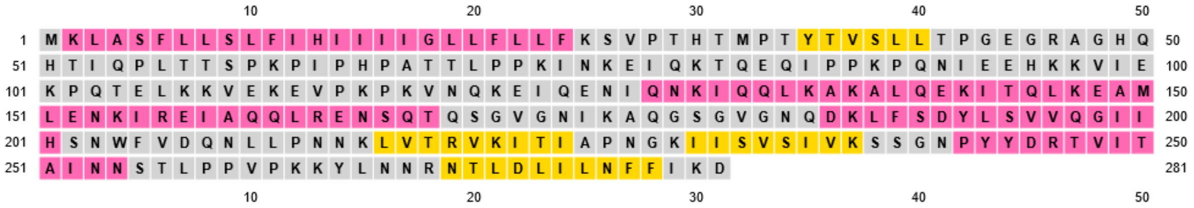

MKLASFLLSLFIHIIIIGLLFLLF
**KSVPTHTMPTYTVSLLTPGEGRAGHQHTIQPLTTSPKPIPHPATTLPPKINKEIQKTQEQIPPKP
QNIEEHKKVIEKPQTELKKVEKEVPKPKVNQKEIQENIQNKIQQLKAKALQEKITQLKEAMLEN
KIREIAQQLRENSQTQSGVGNIKAQGSG**
VGNQDKLFSDYLSVVQGIIHSNWFVDQNLLPNNKLVTRVKITIAPNGKIISVSIVKSSGNPYYDRTV
ITAINNSTLPPVPKKYLNNRNTLDLILNFFIKD

PPIIPRED:

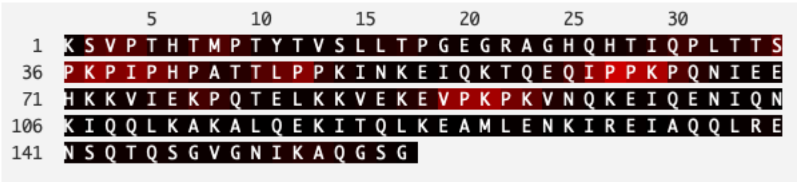

157 Domain II residues

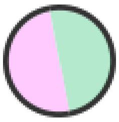

16 Proline residues

41 α-Helix residues

Oligoflexia

Bacteriovoracles (cont.)

Halobacteriovorax marinus

Split system

Locus: 2695527- 2701375

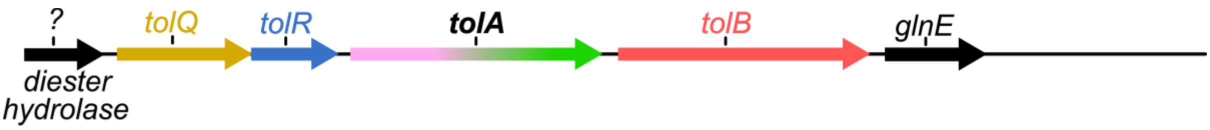

Distal *pal* detected (747172)

>WP_096910104.1 TonB family protein [Halobacteriovorax marinus]

MNTNVLANKSFKYYFSWSFFFHITIAVLFFLFGKLVHDKIQSTRDYNMKLVQASVKVDMVAMPKF
TLKELKAMQPPAKGEVVREKPSAKPAEVINKDDTVFEKKVSKPNFLDMMKDLSNKKVEVKKAKP
TPKKKGADDGIGIDSNTLKKLVAEGNKISKGVALSGTGRSDQELTEFQLYASTLSAKVKQFWKLP
GYLIDKNLKCRIRIYLKSDGSLLRSEIFESSGEEEFDKRAMAAIKQASPFSAVPPASRGNALKGEIV
LGFPL

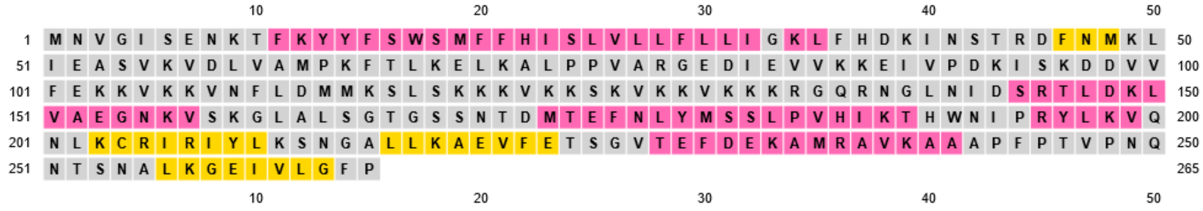

MNTNVLANKSFKYYFSWSFFFHITIAVLFFLF
**GKLVHDKIQSTRDYNMKLVQASVKVDMVAMPKFTLKELKAMQPPAKGEVVREKPSAKPAEVI
NKDDTVFEKKVSKPNFLDMMKDLSNKKVEVKKAKPTPKKKGADDGIGIDSNTLKKLVAEGNKI
SKGVALS**
GTGRSDQELTEFQLYASTLSAKVKQFWKLPGYLIDKNLKCRIRIYLKSDGSLLRSEIFESSGEEEF
DKRAMAAIKQASPFSAVPPASRGNALKGEIVLGFPL

PPIIPRED:

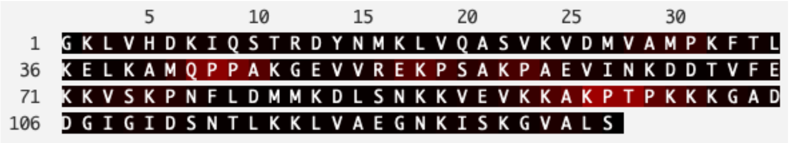

133 Domain II residues

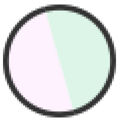

4 Proline residues

16 α-helix residues

Bdellovibrionales

Bdellovibrio bacteriovorus

Split system.

Locus: 164672-171334

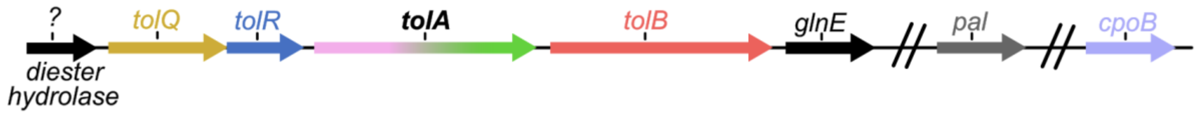

-Flanked by phosphoric diester hydrolase and glutamate-ammonia-ligase adenyl transferase

Distal *pal* present on a different locus. -CpoB is present but distal, AF3 confirmed structure.

>CAE77847.1 TonB-like protein [Bdellovibrio bacteriovorus HD100]

MNYLEEKDQQNDEQVTRGIGISFALHALIISIFTLKTVFFDPEPIDFSQAVRVDMVGLPDKVEPKD
LAPPAKENPKPALPEKEVAKEPVKEKPPEKAPEKVVEKKTPPKPEPVKLPPAKAKEEGINLEKVK
SQQQNALDKLKAMAALEKIKEDVAEDKKKAAAAAGTGKAATGSTPVRGNVLSPGTSLTGIAKLQ
NDNYISDLDRHIKQNWTIPEWLAKRDYKAQVRVFVDSRGNILGRKIVKSSGNPSYDEEVLATIDR
SAPFPAPPEKLIAVFSVDGILIGFPE

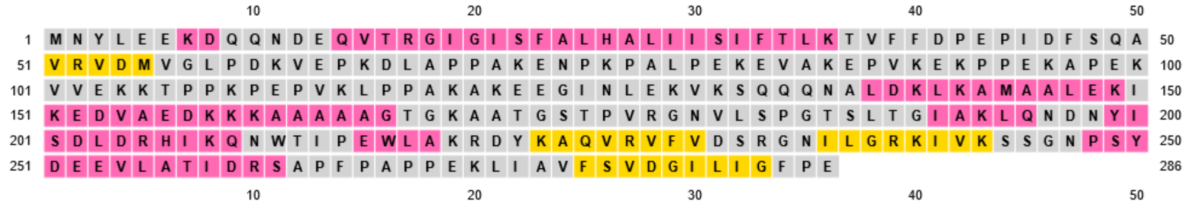

MNYLEEKDQQNDEQVTRGIGISFALHALIISIFTLK
**TVFFDPEPIDFSQAVRVDMVGLPDKVEPKDLAPPAKENPKPALPEKEVAKEPVKEKPPEKAPE
KVVEKKTPPKPEPVKLPPAKAKEEGINLEKVKSQQQNALDKLKAMAALEKIKEDVAEDKKKA
AAAAGTGKAATGSTPVRGNVLSPGT**
SLTGIAKLQNDNYISDLDRHIKQNWTIPEWLAKRDYKAQVRVFVDSRGNILGRKIVKSSGNPSYD
EEVLATIDRSAPFPAPPEKLIAVFSVDGILIGFPE

PPIIPRED:

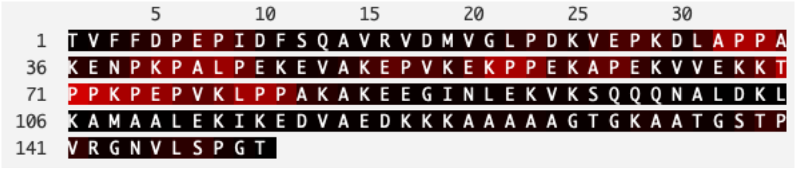

150 Domain II residues

21 Proline residues

28 α-helix residues

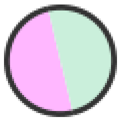

ε-Proteobacteria

Campylobacterales

Helicobacter pylori

Locus: 1119019-1124670

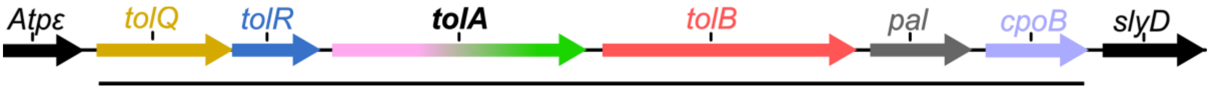

>ABF85133.1 hypothetical protein HPAG1_1066 [Helicobacter pylori HPAG1]

Flanked by ATP subunit epsilon gene and a peptidyl-prolyl-isomerase gene.

MSKSAIFVVSGFLAFLLYALLLYGLLLERHNKEAEKILLDLGKKNEQVIDLNLEDLPSDEKKDEKIA
EKAEEKKDEKAVEKNATDKEGDFIDPKEQEESLEDIFSSLNDFQEKTDTNAQKEEQKNEQEEEQ
RRLREQQRLRKNQKNQEMLKGLQQNLDQFAQKLESVKNKTLDLQIPKQDGVDEKAYQEWYAQI
YQILYKGWKGVFYHKASVSALIMITKDGEFDYTILSYSDFKDYNKSVMTLLNDLKKVDFPPYPGG
SMISIQVNFTTKEEQ

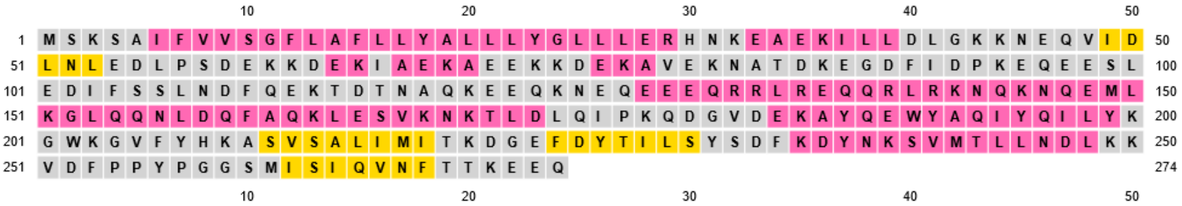

MSKSAIFVVSGFLAFLLYALLLYGLLLER
**HNKEAEKILLDLGKKNEQVIDLNLEDLPSDEKKDEKIAEKAEEKKDEKAVEKNATDKEGDFIDP
KEQEESLEDIFSSLNDFQEKTDTNAQKEEQKNEQEEEQRRLREQQRLRKNQKNQEMLKGLQ
QNLDQFAQKLESVKNKTLDL**
QIPKQDGVDEKAYQEWYAQIYQILYKGWKGVFYHKASVSALIMITKDGEFDYTILSYSDFKDYNK
SVMTLLNDLKKVDFPPYPGGSMISIQVNFTTKEEQ

PPIIPRED:

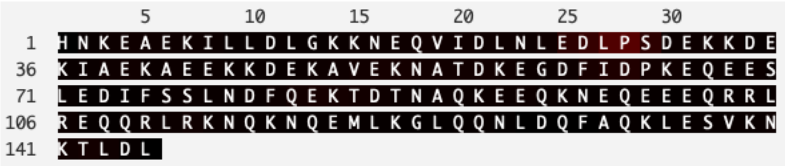

145 Domain II residues

2 Proline residues

62 α-Helix residues

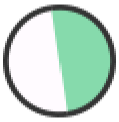

Wolinella succinogenes

Locus: 166155- 171832

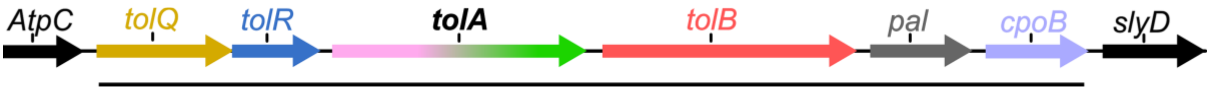

>CAE09657.1 hypothetical protein WS0520 [Wolinella succinogenes]

MDKFALHLSSGVIAVSAYLLLLIFLVFKFSSENSNPTRYSFAKETTFEVSLMEVKPEIKPEVQKLPP
KEEKKVEEIPVTEESASRTPHVGLGVNKLFAQVDAKVPVKEIKPADVHDPIAKKKKSDKPTQKDTI
DESLQKILSDVNVKNTMSFNVPAGQYDEFYAKVNEILYTHWNPLGFSYKEESKVIITITAAGKFNY
KISRKSGNSAFDSALEEFLEKMKTVDFPPFPRGDKTNIEVTFKTEGQV

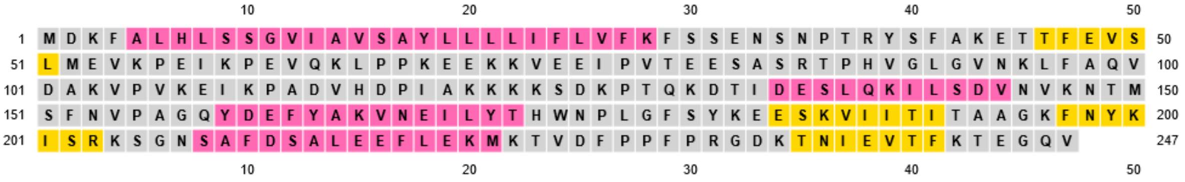

MDKFALHLSSGVIAVSAYLLLLIFLVFK
FSSENSNPTRYSFAKETTFEVSLMEVKPEIKPEVQKLPPKEEKKVEEIPVTEESASRTPHVGLG
VNKLFAQVDAKVPVKEIKPADVHDPIAKKKKSDKPTQKDTIDESLQKILSDVNVK
NTMSFNVPAGQYDEFYAKVNEILYTHWNPLGFSYKEESKVIITITAAGKFNYKISRKSGNSAFDSA
LEEFLEKMKTVDFPPFPRGDKTNIEVTFKTEGQV

PPIIPRED:

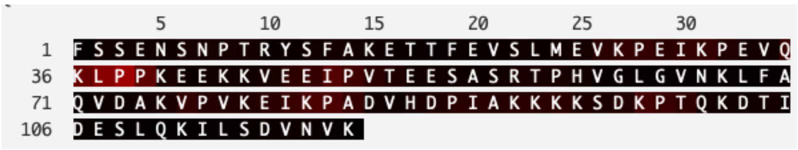

119 Domain II residues

11 Proline residues

13 α-Helix residues

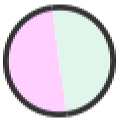

Arcobacter butzleri

Locus: 1601036-1605662

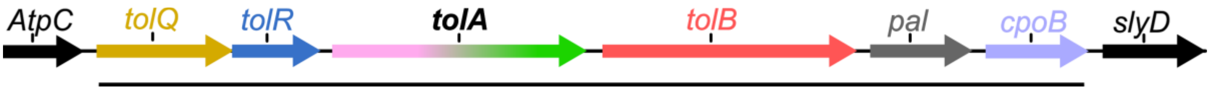

>ABV67839.1 conserved hypothetical protein [Aliarcobacter butzleri RM4018]

MQNKTSFIISGIIAITVYLSFCLLVMIYIFSPTKEQINITPSSTTIELEMIEEIAEKKMVQKKVEKIIEKEE
VVEKATSASNEKRPDLKSLFANVKETAQKVVKEEVNNVEKSIDPKRFKSKFEKEKKSTNIKIDRLL
EDEKTATDSKVKSSSKGEKNDDYASKIYEILQSGAPISQDTNLVAKVVITIDENGKFDYKFQKVSG
DAAYDASLKAYLDGQRNVAYPVPPSGKVVKYSVDFKFEG

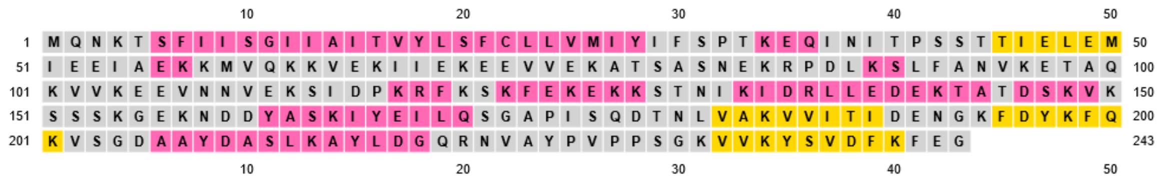

MQNKTSFIISGIIAITVYLSFCLLVMIY
**IFSPTKEQINITPSSTTIELEMIEEIAEKKMVQKKVEKIIEKEEVVEKATSASNEKRPDLKSLFANV
KETAQKVVKEEVNNVEKSIDPKRFKSKFEKEKKSTNIKIDRLLEDEKT**
ATDSKVKSSSKGEKNDDYASKIYEILQSGAPISQDTNLVAKVVITIDENGKFDYKFQKVSGDAAYD
ASLKAYLDGQRNVAYPVPPSGKVVKYSVDFKFEG

PPIIPRED:

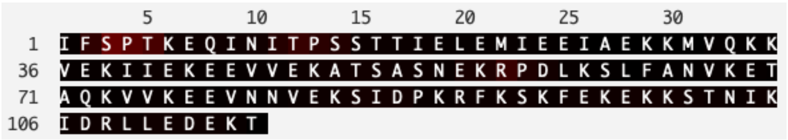

115 Domain II residues

4 Proline residues

28 A-Helix-helices

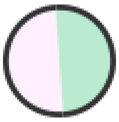

Campylobacter curvus

Locus: 1551290-1555500

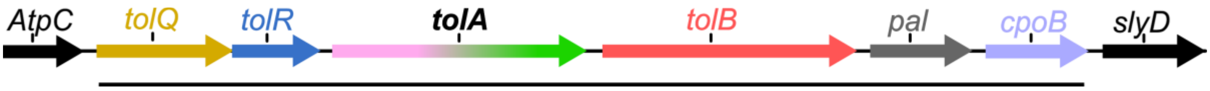

>EAU01084.3 Tol-Pal system subunit TolA [Campylobacter curvus 525.92]

MPSKVKFPTVSSFFVALSLYLTIVLMLFIKLTFFSEPAKKYTDDKDAFMDVVMVDREVAETIKAPK
QANEAVKQTQPEPKKESQETKVETTNKPVVPEEPLPTPSIPTPPKEQPKPEPKPEQKPEIPEPSE
KPDTKPEPPKPVETPNIKDLFSSIDTTKLKKDNGIAKPEQKVQSRKKSEVANSQAAKQASDIIKSL
QIDTVSKAPKSQATGVYDPLRGAIAKQIQRRWQSYKADSNDVATVKFMIDGSGNFSYEILELSYN
EEFNNKVRECLEKLTTEKFPFSPDGKSVTFNLKLEDKLE

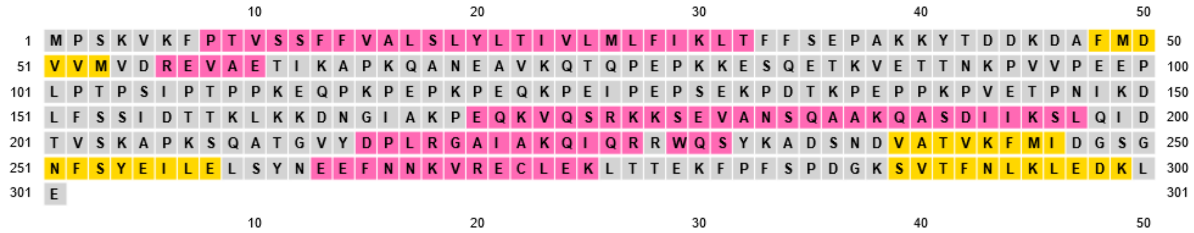

MPSKVKFPTVSSFFVALSLYLTIVLMLFIKLT
FFSEPAKKYTDDKDAFMDVVMVDREVAETIKAPKQANEAVKQTQPEPKKESQETKVETTNKP
VVPEEPLPTPSIPTPPKEQPKPEPKPEQKPEIPEPSEKPDTKPEPPKPVETPNIKDLFSSIDTTKL
KKDNGIAKPEQKVQSRKKSEVANSQAAKQASDIIKSLQIDT
VSKAPKSQATGVYDPLRGAIAKQIQRRWQSYKADSNDVATVKFMIDGSGNFSYEILELSYNEEF
NNKVRECLEKLTTEKFPFSPDGKSVTFNLKLEDKLE

PPIIPRED:

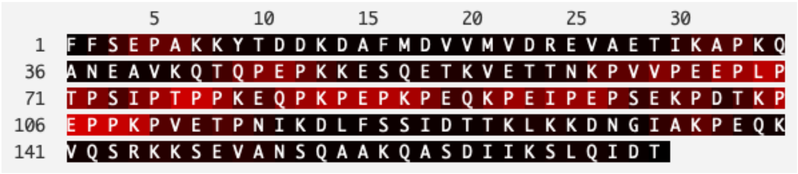

169 Domain II residues

26 Proline residues

33 α-Helix residues

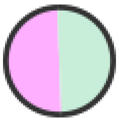

Wider phylae (Witwinowski tree)

These taxa were selected from Witwinowski et al., 2022. Where genome annotations were lacking, *tol-pal* loci were detected by tBLASTn searches against TolB and Pal protein sequences from *E. coli* and/or homologues from most-related neighbours. The *tol-pal* loci detected in less sequence-represented species specified to have *pal* by Witwinowski *et al.*, 2022 were predominantly selected from whole genome assemblies wherever possible.

Candidatus Elusimicrobia

Elusimicrobium minutum Pei191

Split system.

Locus: *tolB-pal-cpoB*: 1377007 – 1381754

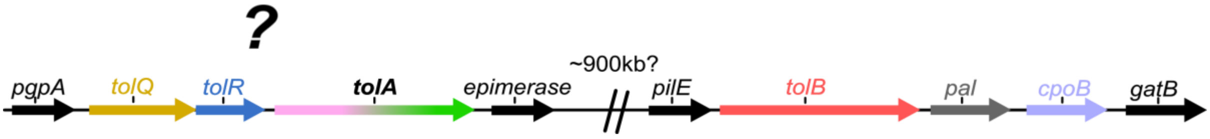

tolB prediction confirms correct structure

Flanked by *pilE* and *gatB*

Possible *TolQRA* locus: 477529-480385

CpoB putative lipoprotein.

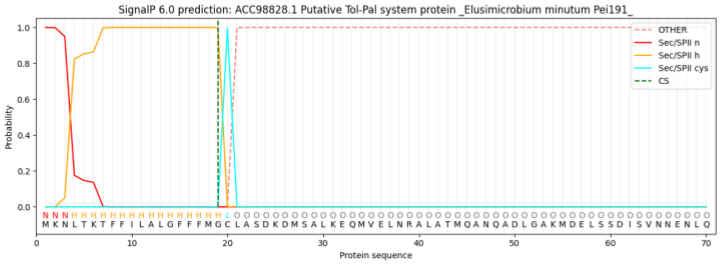

Flanked by *pgpA* and NADP-epimerase gene.

>ACC98002.1 Putative periplasmic protein TonB [Elusimicrobium minutum Pei191]

MFNRYFFISGVLHALMAGFLFFVIMPASVKKPLATYTIDFLGASFQAPADGSDSKKSDEPTPTPAAPKEEAKKEEIK
KPETPVADKKAYAAKSQITTKPQPAPKKKIVLGTPSILADNEKEKKAEAAKTSAKAGSSQGEGDGVGGIKTDFPNF
PFPWYITQVRNALWTEWEKRKPKQANVAALVTFAIQRDGSIKNLKVSKASGNELYDYAAKTSVDAAAPFPPLPAE
FEKSELTVTVEFKDEN

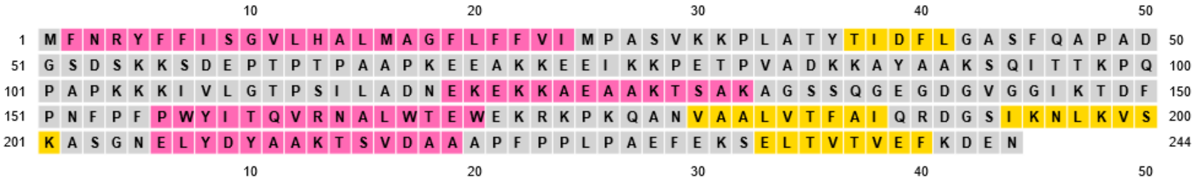

MFNRYFFISGVLHALMAGFLFFVI
**MPASVKKPLATYTIDFLGASFQAPADGSDSKKSDEPTPTPAAPKEEAKKEEIKKPETPVADKKAYAAKSQITTK
PQPAPKKKIVLGTPSILADNEKEKKAEAAKTSAKAGSSQGEGDGVG**
GIKTDFPNFPFPWYITQVRNALWTEWEKRKPKQANVAALVTFAIQRDGSIKNLKVSKASGNELYDYAAKTSVDAA
APFPPLPAEFEKSELTVTVEFKDEN

PPIIPRED:

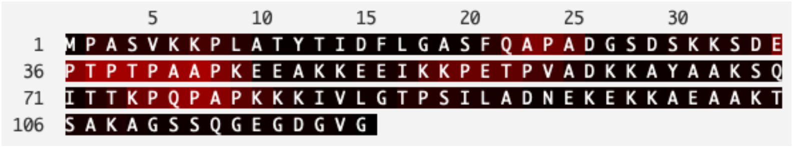

120 Domain II residues

13 Proline residues

14 α-Helix residues

Endomicrobium proavitum

This organism has an unusual gene order but they are all present on same locus.

Locus: 905792-914409

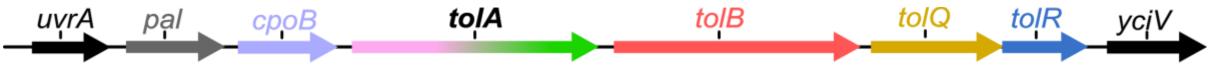

Flanked by *uvrA* nuclease gene and *yciV*, encoding a PHP domain phosphatase.

tolB gene Structurally predicted.

cpoB is a putative lipoprotein.

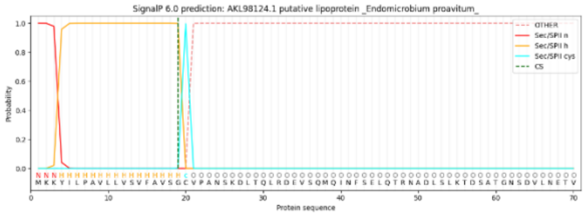

>AKL98123.1 TonB family protein [Endomicrobium proavitum]

MMLKRDNTDSLIKYLAIAIALHLTAVLIYEIHRIRHNRVFMSVPIEVSFFTPSVQRADPAPVAPKEEP
KKQQPKEEPPKKGDVAVKDKSKKPEKSKPVDNTPKTQQSETKPTNEVAGSQYGGLSLDTQDFK
YAYYTNTVVRKIGRFWQWTESYGKLRAVVFFRILKNGAVVDIKVTDSSGDSGYDRNAARAIELA
SPFAPLPEDYEKDSLGVYFEFKYRN

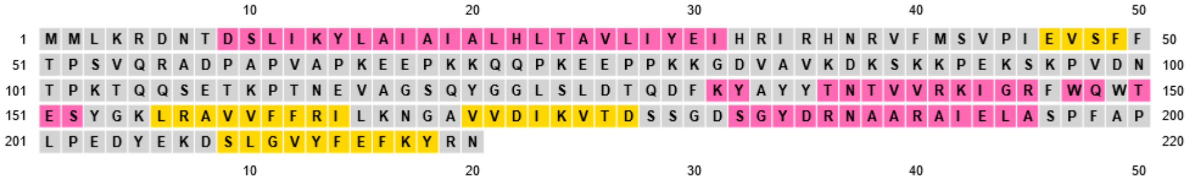

MMLKRDNTDSLIKYLAIAIALHLTAVLIYEI
**HRIRHNRVFMSVPIEVSFFTPSVQRADPAPVAPKEEPKKQQPKEEPPKKGDVAVKDKSKKPE
KSKPVDNTPKTQQSETKPTNEVAGSQY**
GGLSLDTQDFKYAYYTNTVVRKIGRFWQWTESYGKLRAVVFFRILKNGAVVDIKVTDSSGDSGY
DRNAARAIELASPFAPLPEDYEKDSLGVYFEFKYRN

PPIIPRED:

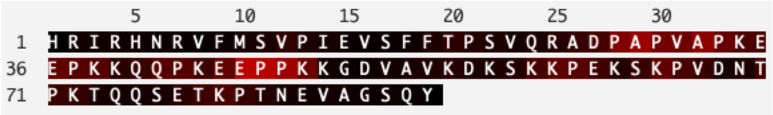

89 Domain II residues

13 Proline residues

0 α-Helix residues

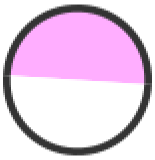

I used BLAST to compare these two elusiomicrobial TolA sequences, showing they are homologues. Assuming that the sequence for *E. proavitum* is correct due to its synteny with other components.

```
Score            Expect  Method                          Identities     Positives      Gaps
70.5 bits(171)   2e-19   Compositional matrix adjust.    60/183(33%)    93/183(50%)    21/183(11%)

Query  62   PTPAAPKEEAKKEEIKKPETPVADKKAYAAKSQITTKPQPAPKKKIVLGTPSILADNEKE  121
            P P APKEE KK++ K+ E P   KK   A   +  K +   K K V  TP       K 
Sbjct  59   PAPVAPKEEPKKQQPKE-EPP---KKGDVA---VKDKSKKPEKSKPVDNTP-------KT  104

Query  122  KKAEAAKTSAKAGSSQGEGDGVGGIKTDFPNFPFPWYITQVRNALWTEWEKRKPKQANVA  181
            +++E   T+  AGS        GG+  D  +F + +Y   V   +   W+  +     + 
Sbjct  105  QQSETKPTNEVAGSQ------YGGLSLDTQDFKYAYYTNTVVRKIGRFWQWTE-SYGKLR  157

Query  182  ALVTFAIQRDGSIKNLKVSKASGNELYDYAAKTSVDAAAPFPPLPAEFEKSELTVTVEFK  241
            A+V F I ++G++ ++KV+ +SG+  YD  A  +++ A+PF PLP ++EK  L V  EFK
Sbjct  158  AVVFFRILKNGAVVDIKVTDSSGDSGYDRNAARAIELASPFAPLPEDYEKDSLGVYFEFK  217

Query  242  DEN  244
              N
Sbjct  218  YRN  220
```

This homology suggests we correctly identified *tolA* in *E. minutum.*

Candidatus mciNerneyibacteriota

Candidatus Mcinerneyibacteriaceae

Candidatus Mcinerneyibacterium aminivorans

Witwinowski suggested no *pal.*

Some β-propeller structure predictions but all lack *tolB* NTD.

Aquificae

Thermosulfidibacter takaii ABI70S6

Locus: 1705923- 1711862

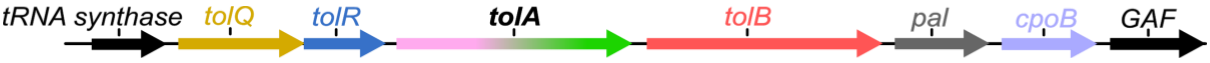

tolB has correct structural prediction.

Flanked by a tRNA synthase gene and a GAF containing gene for a diguanylate cyclase.

CpoB putative lipoprotein.

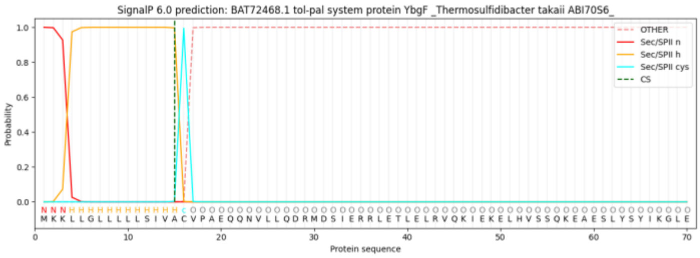

>BAT72471.1 periplasmic protein TonB [Thermosulfidibacter takaii ABI70S6]

MLNAKFFQGIVLSVLLHAFLVWFFYTVQGTAKV
TIPLGVVYNVEIVPAQKLSATGSRRVRTSKVSRRTKIKKPVRVRKKSESVKPKMPKIKKVIKLKK
NKNTVLISKKKAKVVKKRKANKVKPSKAVSEKQLLEQRLKELRAEKELEKKLEALRREKERE
QSTEEEGSLVSAKGAGSE
IAVSSASVKLDPVIYLYLSRLVSRIQLNWSLPEGITNKEAIVSVKIDRSGRLVHLALEKSSGDALFD
ESCLRAVRKSFPFDPLPSAYKGDYFEIGVRFKR

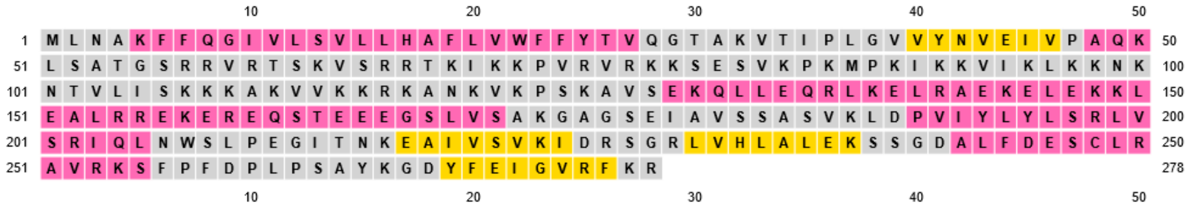

PPIIPRED:

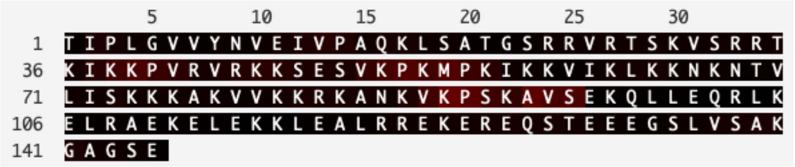

145 Domain II residues

6 Proline residues

43 α-Helix residues

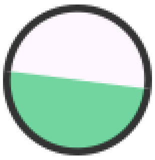

Thermodesulfobacteria

Gemmatimonas aurantiaca

Thermodesulfobacteria often have multiple TolBs (*Geobacter metallireducens* has 5?)

Gemmatimonas aurantiaca has multiple ExbDs, suggesting heterodimers.

Locus: 2268048-2274531

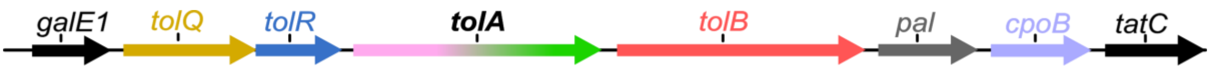

Flanked by NADP-binding glucose epimerase gene and *tatC*.

tolB mislabeled “pectate lyase”, but AF3 has correct predicted structure.

CpoB putative lipoprotein.

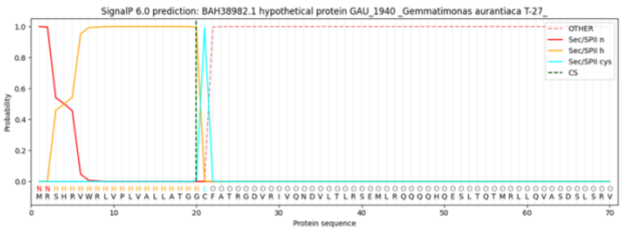

>BAH38985.1 hypothetical protein GAU_1943 [Gemmatimonas aurantiaca T-27]

MTSARATGNAPTSLTRGMLASVVVHGAMAALVAWWSTRPAPQRPPVYRVELVGQAGPRQAG
VETPTAAPAAKAPDVAGAERVKEEKVVPTPSKAKKVLPSPKATPSPTRSKQAGSKTAAPTATKS
TSAPRAGAGATGTKGADVANVRTDGIAFPFPGYLSNIVRQITLNWSPRRVSAALITEVKFMIRRD
GSVAGIEVVRASGDRLYDLDGMGAVEAVGSTRSFGPLPSGWSDDVLVVYFTFDYALRPNMTSA
RATGNAPTSLTRGMLASVVVHGAMAALVAW

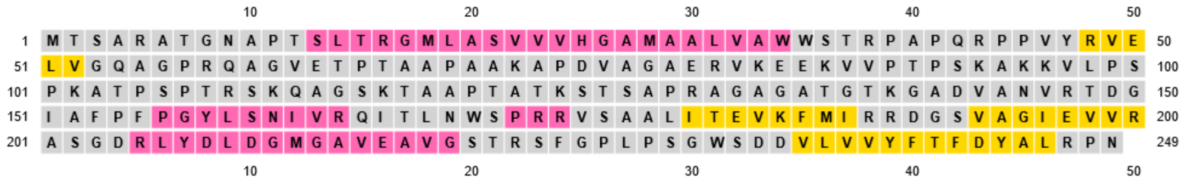

MTSARATGNAPTSLTRGMLASVVVHGAMAALVAW
WSTRPAPQRPPVYRVELVGQAGPRQAGVETPTAAPAAKAPDVAGAERVKEEKVVPTPSKAK
KVLPSPKATPSPTRSKQAGSKTAAPTATKSTSAPRAGAGATGTKGADVANVRTD
GIAFPFPGYLSNIVRQITLNWSPRRVSAALITEVKFMIRRDGSVAGIEVVRASGDRLYDLDGMGA
VEAVGSTRSFGPLPSGWSDDVLVVYFTFDYALRPN

PPIIPRED:

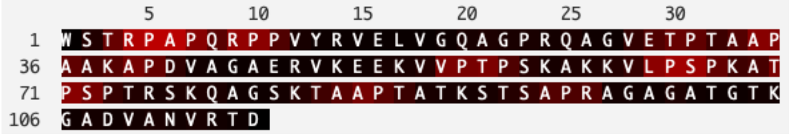

115 Domain II residues

16 Proline residues

0 α-Helix residues

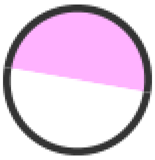

Candidadatus Calescamentes

Candidatus Calescibacterium sp. isolate PSS.bin.4 NODE_13_length_104994_cov_86.033305,

Split operon.

Since *tolQRA* is separated from *tolB-pal*, impossible to say which gene is *tolA*

Locus:

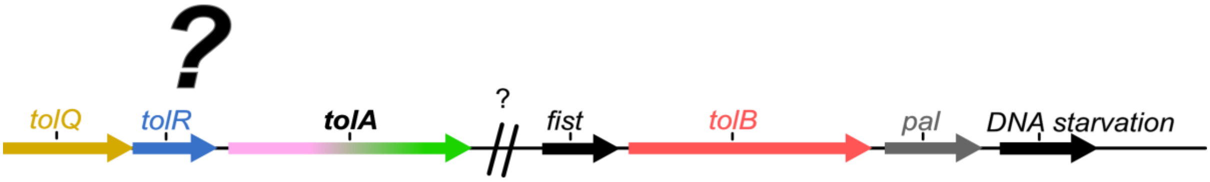

fist *tolB* *pal* *DNA-starvation*

TolB and Pal confirmed by Alphafold- Pal has an extra α-helical bundle in its traditionally disordered tether region:

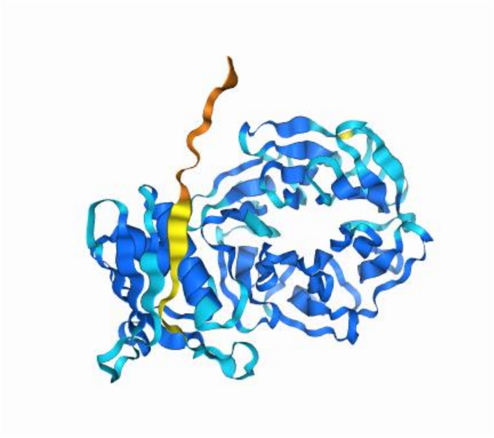

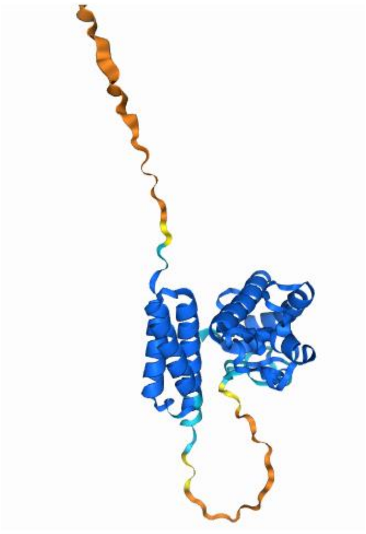

Candidatus Dependentia

Candidatus Dependentiae bacterium

Locus: 159816-166947

Several proteins in a row, predicted with AF3: *tolQ* *tolR* *tolA* *tolB* *????* *???*

Flanked by polynucleotide adenylyltransferase gene and *ftsH*.

Witwinowski found no *pal*, but on another sequence fragment there is *pal* (Prediction). Numerous search results with appropriate structural predictions suggest Pal is present within this taxon. Potential *cpoB* homologues found via BLAST lack trimerisation region.

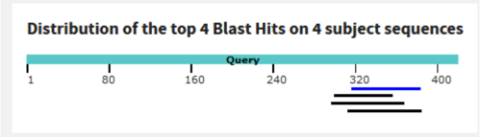

>HBZ73040.1 MAG TPA: hypothetical protein DEO28_00810 [Candidatus Dependentiae bacterium]- **confirmed via alphafold, looks unusual.**

MKNELNLRIILYSFLLHIFILVFASFILKERSPVRIFIKGDATSTRFWASRKHLKSIALVDFSKKRKRRFALNKKISSKV
TKKFVPKTIKVAQKTKIRVAKKANFVDEFSGKIAKNKKIVKQQKQVLLEEQKMLDAEKKELHKKEKQSQAKSEEVK
KIETAEKEEIKKIEQKTEDVKPLQEDIKPQKEEIKQEVADITSQEDPTAVEAEVSSEGIGSSSKPILGREEDLGV
ENIDFATAERFSKEFGGKLRAVRLFREFNMPVKFLVCSDGKVNDINFPKNVGTKVDQVAIIRALRQIDFAKDY
GREMFLVFNRNKKGKISIGGDLA

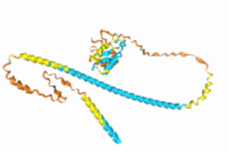

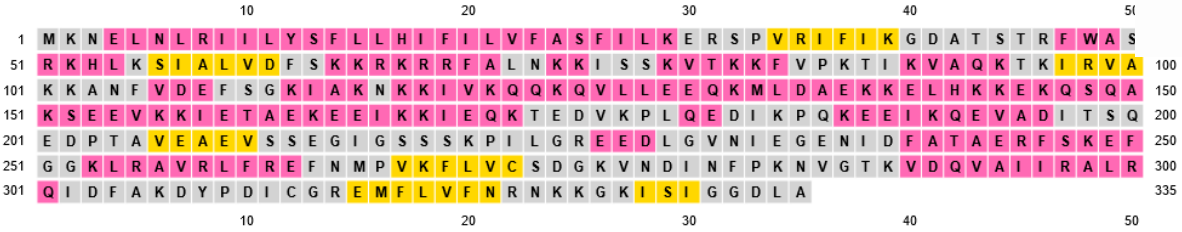

MKNELNLRIILYSFLLHIFILVFASFILK
ERSPVRIFIKGDATSTRFWASRKHLKSIALVDFSKKRKRRFALNKKISSKVTKKFVPKTIKVAQKTKIRVAKKANF
VDEFSGKIAKNKKIVKQQKQVLLEEQKMLDAEKKELHKKEKQSQAKSEEVKKIETAEKEEIKKIEQKTEDVKPL
QEDIKPQKEEIKQEVADITSQEDPTAVEAEVSSEGIGSSSKPILGREEDLGVNIEG
ENIDFATAERFSKEFGGKLRAVRLFREFNMPVKFLVCSDGKVNDINFPKNVGTKVDQVAIIRALRQIDFAKDYPDIC
GREMFLVFNRNKKGKISIGGDLA

PPIIPRED:

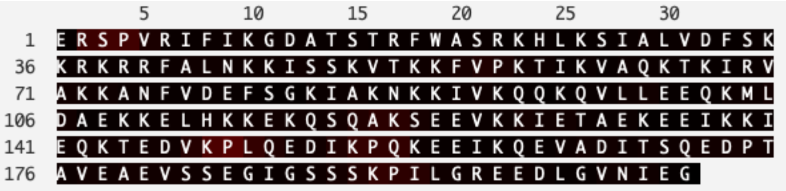

206 Domain II residues

6 Proline residues

110 α-helix residues

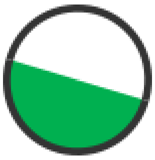

Epsilonproteobacteria

Used *Wolinella* as representative operon (page 86).

Candidatus Methylomirabilis

Methylomirabilis oxygeniifera

Locus: 432467-438878

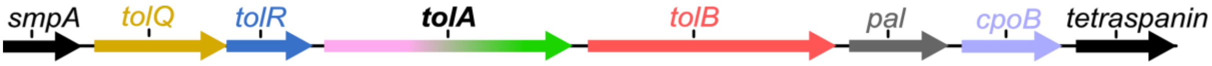

Has an *envC-cpoB* also described as “BamD-like”, AF3 looks like CpoB.

CpoB putative lipoprotein.

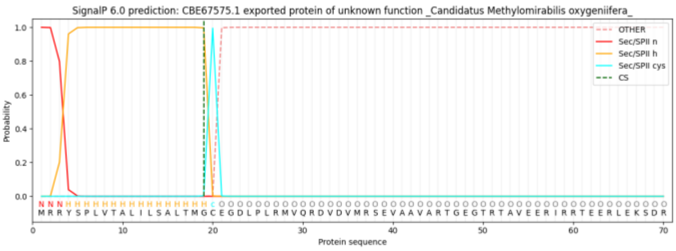

Flanked by *smpA* and a tetraspanin gene.

>CBE67572.1 protein of unknown function [Candidatus Methylomirabilis oxygeniifera]

MVVGAIGRRLPVSAVSSSCGLSFLLHGLLAVAVVYGPQWLHGKPFIAPLNYEVTLISPAEENREL
RRGAALPAQPKVATAAISVPAGSRELLTLPSLSRTTSPKAQTDELTLSAKRRAPTRLPVAPPLTT
PGPPKIVAPLVASVPVGAQPGIMSPVVDPAKAGAGRDPGTVAETGVTVGNTDPALAYYFVLIQD
KITSNWMPPKMSPGAMAGVSVSLRILRSGQIRNLAVGSSSGDRLLDDSAVRAISLSSPLPPLPPL
YKAEALSLELRFTFVGEKS

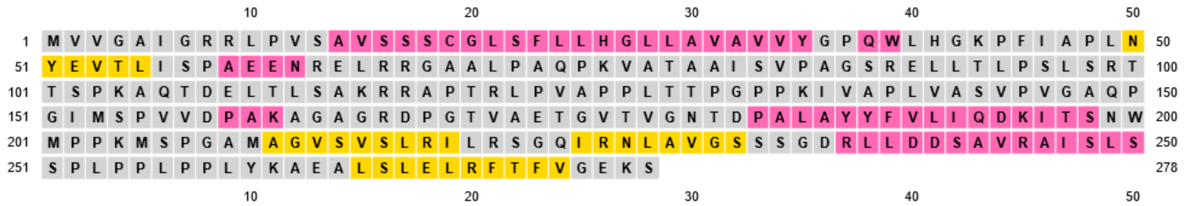

MVVGAIGRRLPVSAVSSSCGLSFLLHGLLAVAVVY
**GPQWLHGKPFIAPLNYEVTLISPAEENRELRRGAALPAQPKVATAAISVPAGSRELLTLPSLSR
TTSPKAQTDELTLSAKRRAPTRLPVAPPLTTPGPPKIVAPLVASVPVGAQPGIMSPVVDPAKA
GAGRDPGTVAETGVTV**
GNTDPALAYYFVLIQDKITSNWMPPKMSPGAMAGVSVSLRILRSGQIRNLAVGSSSGDRLLDDS
AVRAISLSSPLPPLPPLYKAEALSLELRFTFVGEKS

PPIIPRED:

143 Domain II residues

21 Proline residues

11 α-Helix residues

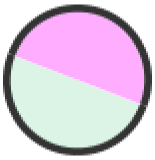

Candidatus Rokubacteria

Candidatus Rokubacteria bacterium CSP1-6

Locus: 3684 -9885

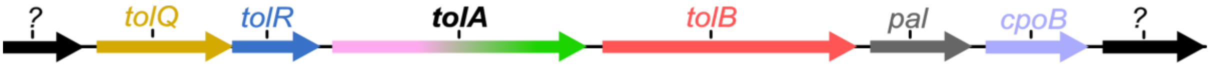

Flanked by unknown gene and unknown gene.

>KRT71774.1 MAG: TonB family protein, colicin import membrane protein [Candidatus Rokubacteria bacterium CSP1-6]

MYPRRRRLGPPLRGGSFPLFGVTVSAAGHVLTAAALLLLAGVWNQWNASKVYVVNLVPAVSIT
GSPTSRSTVPSLPPRPTISEPQRPAPEPVAREVPTAPKPAEPSLPQPRAASASRPVALPRPGEK
ELPPLMAAAERRSAPAAAPTPSEKPGTSSEARTAVPPVPLGRPDGSPAGVASLALEVSNFPFT
WYLQQVQRKVTEKWVQPTRTTGPGLRVVVLFEIARDGQVTGSKVEQSSGNSWYDQSALRAVV
EANPFPPLPEGFPGQSLRVHFGFDFKSS

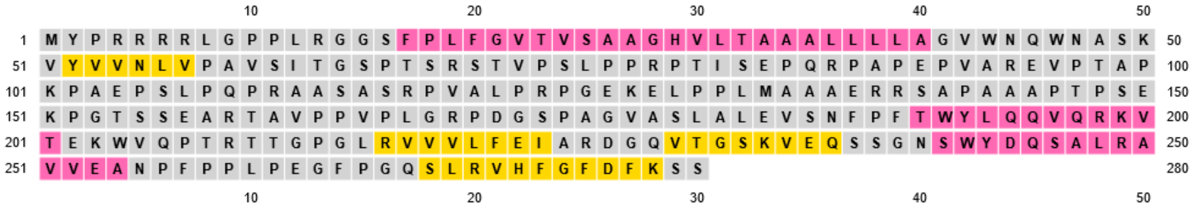

MYPRRRRLGPPLRGGSFPLFGVTVSAAGHVLTAAALLLLA
**GVWNQWNASKVYVVNLVPAVSITGSPTSRSTVPSLPPRPTISEPQRPAPEPVAREVPTAPKPA
EPSLPQPRAASASRPVALPRPGEKELPPLMAAAERRSAPAAAPTPSEKPGTSSEARTAVPPV
PLGRPDGSPAGVASL**
ALEVSNFPFTWYLQQVQRKVTEKWVQPTRTTGPGLRVVVLFEIARDGQVTGSKVEQSSGNSW
YDQSALRAVVEANPFPPLPEGFPGQSLRVHFGFDFKSS

PPIIPRED:

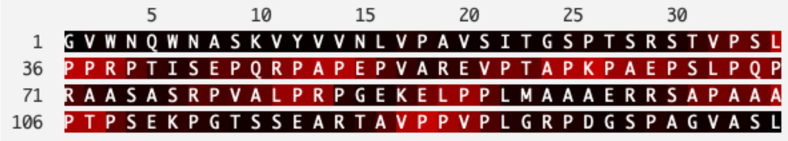

140 Domain II residues

30 Proline residues

0 α-helix residues

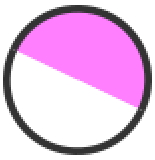

Candidatus Fischerbacteria

Locus: 10806- 16786

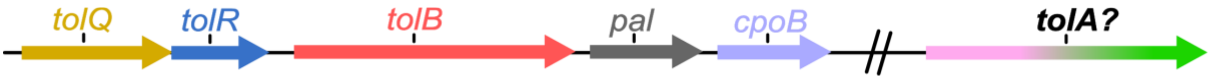

tolQ-*tolR*-*tolB*-*pal*-*cpoB*-

Unclear where *tolA* is, existence assumed.

Multiple TonB/TolA candidates, not possible to verify which is correct.

Candidatus Aminicenantes

Candidatus Aminicenantes bacterium

Split operon:

Locus: *tolQRAB 3097072- 3104664* *pal-cpoB 725362- 727651*

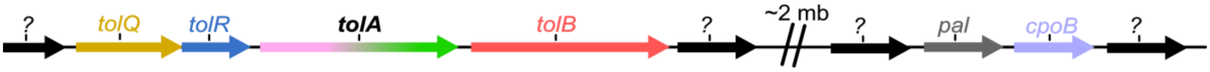

CpoB putative lipoprotein.

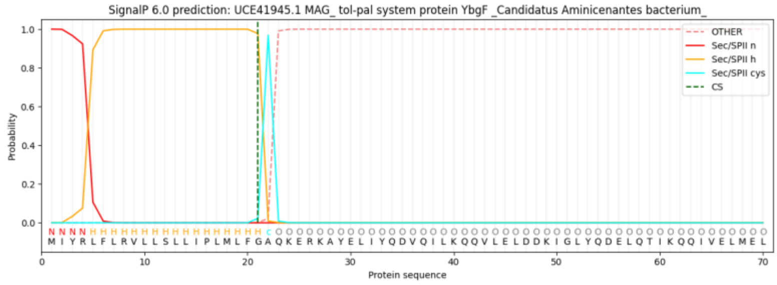

>UCE40448.1 MAG: TonB C-terminal domain-containing protein [Candidatus Aminicenantes bacterium]

MVTYIKQYPLFKKFVYISISFHIILFFLMIFSPKFPSLGRKKMIHYVSLHSFSGGGGGGRLAGGGPS
AKMQPKASESKEELVETPAESGQTLRDLTTPQKLDQQSLSSLRYPVEKPKREKNPPAQKKAVIQ
KQDPSAKKPTGDAESDAEEGTGSGSAISLGVGTGSGGGAGFGSEFSSQIGLSNFPFDYYLENMI
GRISSNWLKTQISSGLSDELFTVVRFKIYRDGKISVVDIEHGCGIRTLDLAAVRAIQSSAPFAPLPD
GYEEDSLIIHLRFEHIK

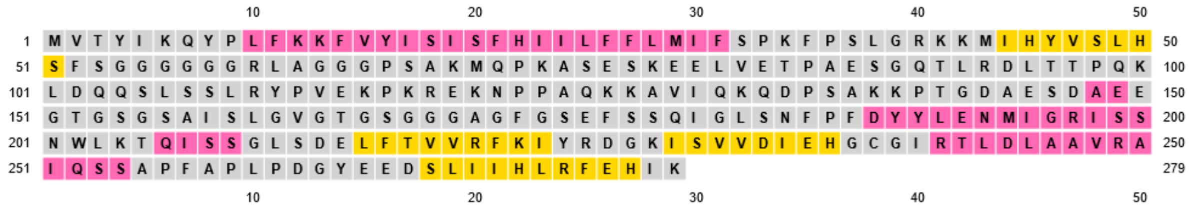

MVTYIKQYPLFKKFVYISISFHIILFFLMIF
**SPKFPSLGRKKMIHYVSLHSFSGGGGGGRLAGGGPSAKMQPKASESKEELVETPAESGQTL
RDLTTPQKLDQQSLSSLRYPVEKPKREKNPPAQKKAVIQKQDPSAKKPTGDAESDAEEGTGS
GSAISLGVGTGSGGGAGFGSEFSS**
QIGLSNFPFDYYLENMIGRISSNWLKTQISSGLSDELFTVVRFKIYRDGKISVVDIEHGCGIRTLDL
AAVRAIQSSAPFAPLPDGYEEDSLIIHLRFEHIK

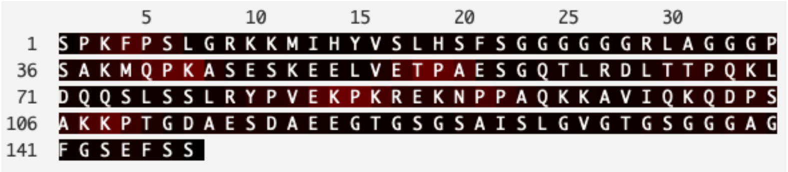

147 Domain II residues

12 Proline residues

0 α-helix residues

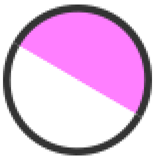

Acidobacteria

Locus: 718225- 725986.

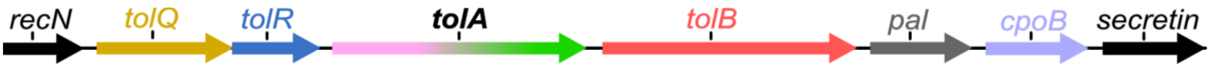

Flanked by *recN* and T2/3SS secretin gene.

Pal prediction has an extra beta-sheet domain in the disordered region. Looks like a carboxypeptidase E (CPE) domain.

>ABF39598.1 Cell division and transport-associated protein TolA [Candidatus Koribacter versatilis Ellin345]

MAVRAEIFEEHERWAPSLVASTVMHVGLTAVIVFTGWANFHHPGENWGGDTSGGGGAMSATL
VSAIPLPRPVEETQNVLANDSKGLTQTTPAVKETPPPEAIAIPDKQAKTKPQKAPPAPRDVKKPK
PVEEAKNNEIPYGEGGPASAMYSNAGTNFAMGTTKGGISVGQGGDFGNHYAYYVDAVRRKITE
NWLRYEIDPHTPPGKRTYITFDINRDGSPSNIRVEQSSGIPSLDISATRALQRIDTFGPLPGGYSG
NKVSVEFYFER

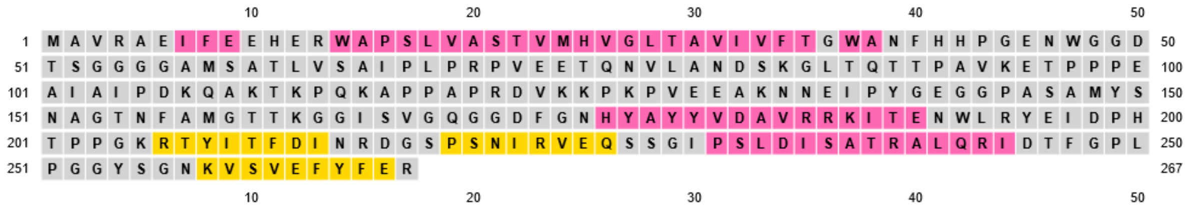

MAVRAEIFEEHERWAPSLVASTVMHVGLTAVIVFT
GWANFHHPGENWGGDTSGGGGAMSATLVSAIPLPRPVEETQNVLANDSKGLTQTTPAVKET PPPEAIAIPDKQAKTKPQKAPPAPRDVKKPKPVEEAKNNEIPYGEGGPASAMYSNAGTNFAM GTTKGGIS
VGQGGDFGNHYAYYVDAVRRKITENWLRYEIDPHTPPGKRTYITFDINRDGSPSNIRVEQSSGIP SLDISATRALQRIDTFGPLPGGYSGNKVSVEFYFER

PPIIPRED:

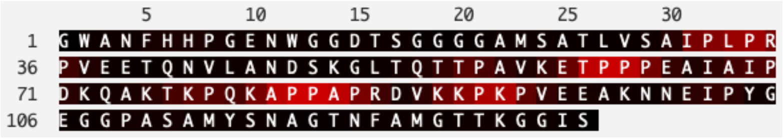

131 Domain II residues

17 Proline residues

2 α-Helix residues

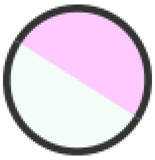

Deferribacteres

Deferrimicrobium borealis

[Locus](): 361012- 367726

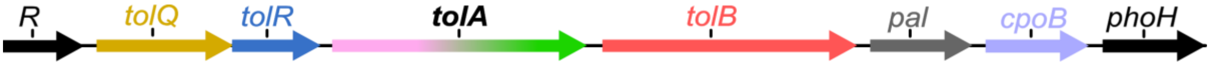

Flanked by a [transcriptional repressor gene]() and [*phoH*]().

CpoB is a putative lipoprotein.

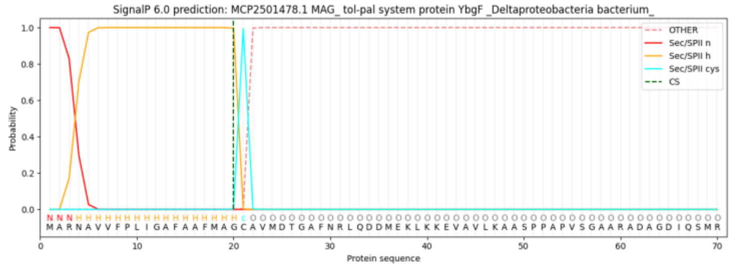

>[MCP2501481.1]() MAG: TonB C-terminal domain-containing protein [Deltaproteobacteria bacterium]

MLALSVAAHMALAMGGLVVLPWFGTPLRLEPVAVVDLIGGGEFRQEAGKPPEPTQAARETVEP
APRERTASAHPPVKREKAPKAAPDDYVPAKQRAADAAALSERLRKMREARAGSEAIRGAVEER
RSEAAARAAVRSVGERVAHRIEAPPPVRPGAREGAGGGGGAQGSVRLSPELSDYFRRLEESV
RNSWVLPGALVRDAGKLVVELRIVIEKDGRVSAQRIERGSGNTYFDDSVLRAIRKASPLPVPPEQ
LRGGEDHYEVGFRFHGGAR

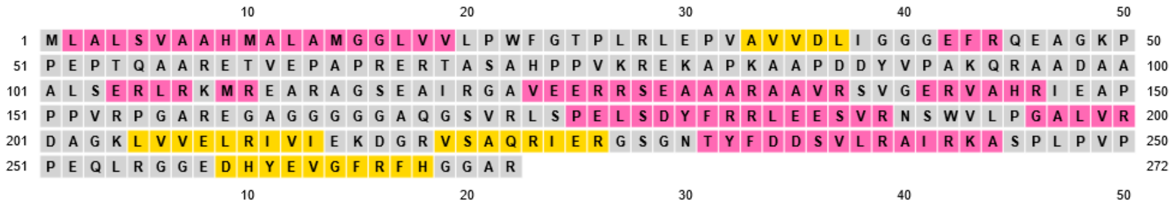

MLALSVAAHMALAMGGLVV
LPWFGTPLRLEPVAVVDLIGGGEFRQEAGKPPEPTQAARETVEPAPRERTASAHPPVKREKA PKAAPDDYVPAKQRAADAAALSERLRKMREARAGSEAIRGAVEERRSEAAARAAVRSVGER VAHRIEAPPPVRPGAREGAGGGGGAQGSVR
LSPELSDYFRRLEESVRNSWVLPGALVRDAGKLVVELRIVIEKDGRVSAQRIERGSGNTYFDDS VLRAIRKASPLPVPPEQLRGGEDHYEVGFRFHGGAR

PPIIPRED:

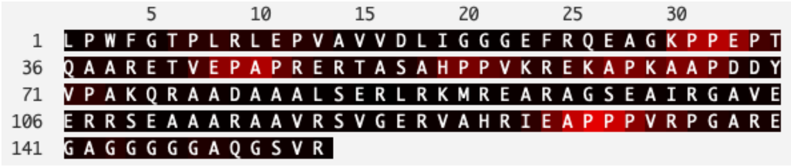

153 Domain II residues

17 Proline residues

29 α-Helix residues

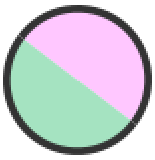

Chrysiogenetes

Desulfurispirillum indicum

Locus: 715999- 721765

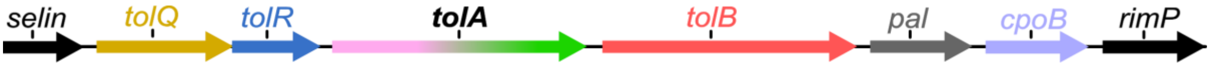

Flanked by a selin gene and *rimP* which encodes a ribosome maturation factor.

CpoB putative lipoprotein.

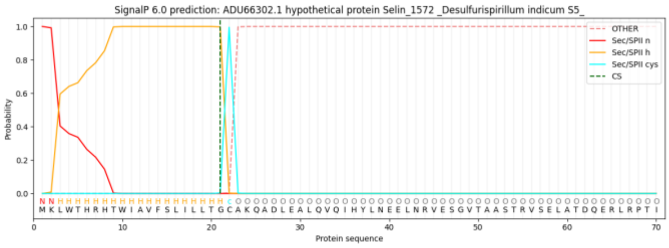

>ADU66305.1 hypothetical protein Selin_1575 [Desulfurispirillum indicum S5]

MLQWITPLLCLRNSTAASVALHGVFILIYALLSLSSSVSPPVTPNIRVHLIPEVADRTTTTQATPQE
APVIATSSKQASTAPPRVQPPVPATDVRQQTQAAVDRLRREQENQRLQQQTQQAIDRLARQQA
QTAPAPQTPPATRQEVPVAPPQATITGNSGAETEAQTVWDMSGDREAISYLEAITAIITEHFKNRI
DLSVEATRDPVVRFSLSRDGHIIPESVSIRTSSGSQIIDRALILAVQRGNPYMPFPASIPRQTITIDV
RGNIHD

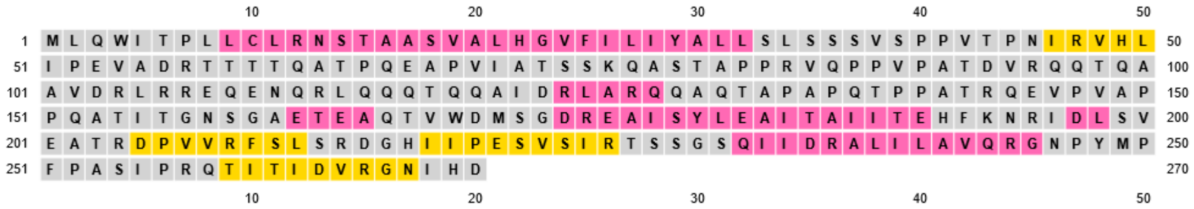

MLQWITPLLCLRNSTAASVALHGVFILIYALL
**SLSSSVSPPVTPNIRVHLIPEVADRTTTTQATPQEAPVIATSSKQASTAPPRVQPPVPATDVRQ
QTQAAVDRLRREQENQRLQQQTQQAIDRLARQQAQTAPAPQTPPATRQEVPVAPPQATITGN
SGAETEAQTVWD**
MSGDREAISYLEAITAIITEHFKNRIDLSVEATRDPVVRFSLSRDGHIIPESVSIRTSSGSQIIDRALIL
AVQRGNPYMPFPASIPRQTITIDVRGNIHD

PPIIPRED:

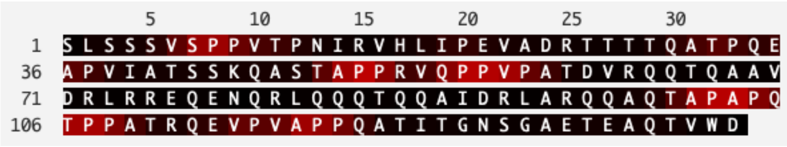

138 Domain II residues

18 Proline residues

9 α-Helix residues

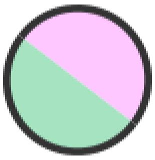

Nitrospinae

Nitrospina gracilis

Locus: 9389- 17352

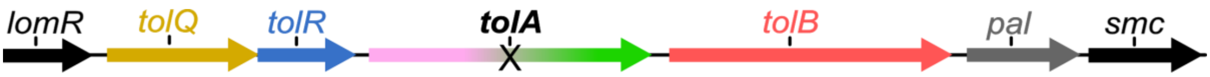

Features a broken *tolA,* split into a separate gene in domain II. Second tolA gene.

Flanked by lomR and smc, encoding a chromosome segregating ATPase

>CCQ90618.1 hypothetical protein NITGR_330016 [Nitrospina gracilis 3/211]

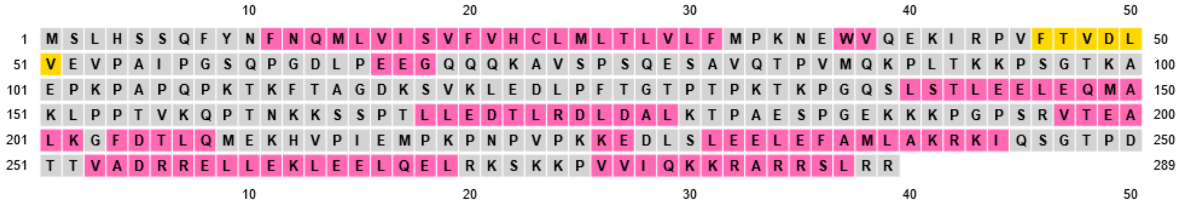

This gene is broken, as evidenced by lack of CTD beta sheets. However, the second gene is the CTD:

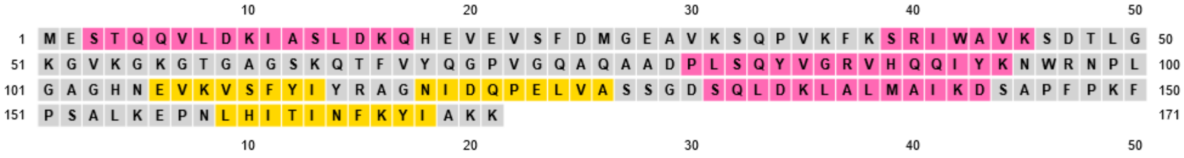

It becomes apparent that a mutation has severed the gene. This could be a sequencing error. To obtain a helix: Proline value for the dataset, the two genes were merged and analysed, but it is noted that it may be non-functional.

MSLHSSQFYNFNQMLVISVFVHCLMLTLVLF
**MPKNEWVQEKIRPVFTVDLVEVPAIPGSQPGDLPEEGQQQKAVSPSQESAVQTPVMQKPLTKKPSGTKAEPKPAPQPKTKFT
AGDKSVKLEDLPFTGTPTPKTKPGQSLSTLEELEQMAKLPPTVKQPTNKKSSPTLLEDTLRDLDALKTPAESPGEKKKPGPS
RVTEALKGFDTLQMEKHVPIEMPKPNPVPKKEDLSLEELEFAMLAKRKIQSGTPDTTVADRRELLEKLEELQELRKSKKPVVI
QKKRARRSLRRMESTQQVLDKIASLDKQHEVEVSFDMGEAVKSQPVKFKSRIWAVKSDTLGKGVKGKGTGAGSKQTFVYQ
GP**
VGQAQAADPLSQYVGRVHQQIYKNWRNPLGAGHNEVKVSFYIYRAGNIDQPELVASSGDSQLDKLALMAIKDSAPFPKFPSALK
EPNLHITINFKYIAKK

PPIIPRED:

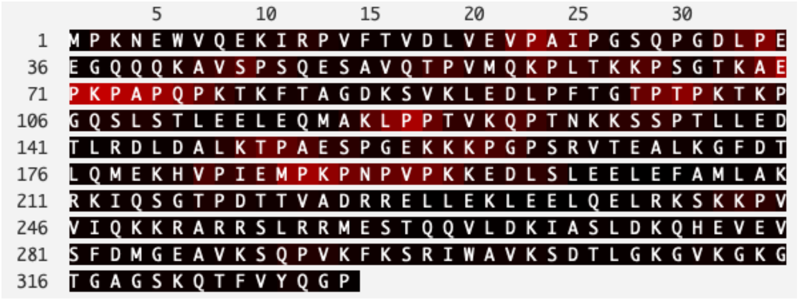

329 Domain II residues

35 Proline residues

28 α-Helix residues

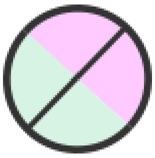

Nitrospina watsonii

Locus: 2410238-2419587

Flanked by elongation factor *efp* and *envC/amiA*.

cpoB homologue detected on a different sequence, labeleld *bamD*, but AF3 predicts CpoB conformation.

>CAI2719169.1 protein of unknown function [Nitrospina watsonii]

MRMNASQFYNFNQMLVISVFVHCLMLTAVLFMPKAEWVQQKIRPMFTVDLVEAPALPGSQRGD
LAEEGKKQEATAPPQQQVAVQKPATEKPMAKKPAAEKGVSKPVPEAKSQAATGQKQFKLEDLP
FTGTPTPKTKPGQSVSTLQELEQVAKLPPTVKQPTQKKTTPTLLEDTLRDLDALKTPSPSPGEKK
KPGPSRVTEALEGFDTLQMKKSVPIEMSKPVPVQQKEDLSMEELEFAMLAERKVKSGTPDTTVA
DQRELLQKLEELQKLKASKKPVVIAKKTDSREFETLIVQQLESTQEILDKIASLSKKHEVEVSFDM
DEASRKQPVQFKSRVWALNTLGGGVQGTGPGTASKQTFVYQGPVGRAQAADPLSQYIGRVHQ
QIYKNWRNPLGAGHNEVKVSFYIFRAGNIDQPELIASSGDGQLDKLALMAIKDSAPFPDFPSALK
EPNLHITINFKYVPKK

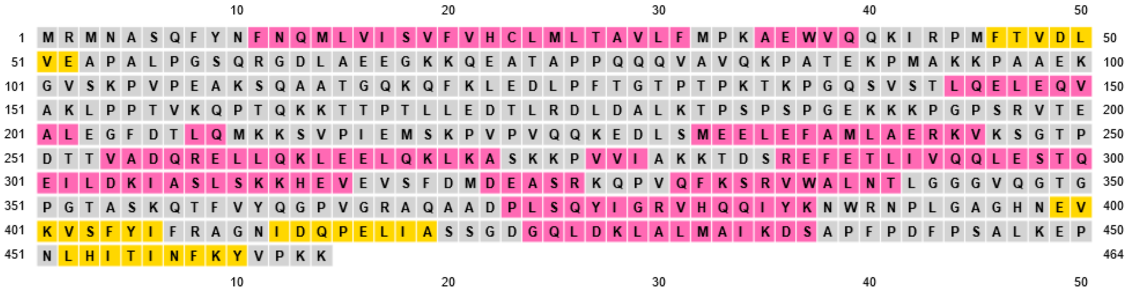

MRMNASQFYNFNQMLVISVFVHCLMLTAVLF
**MPKAEWVQQKIRPMFTVDLVEAPALPGSQRGDLAEEGKKQEATAPPQQQVAVQKPATEKPM
AKKPAAEKGVSKPVPEAKSQAATGQKQFKLEDLPFTGTPTPKTKPGQSVSTLQELEQVAKLP
PTVKQPTQKKTTPTLLEDTLRDLDALKTPSPSPGEKKKPGPSRVTEALEGFDTLQMKKSVPIE
MSKPVPVQQKEDLSMEELEFAMLAERKVKSGTPDTTVADQRELLQKLEELQKLKASKKPVVI
AKKTDSREFETLIVQQLESTQEILDKIASLSKKHEVEVSFDMDEASRKQPVQFKSRVWALNTL
GGGVQGTGPGTASKQTFVYQGP**
VGRAQAADPLSQYIGRVHQQIYKNWRNPLGAGHNEVKVSFYIFRAGNIDQPELIASSGDGQLDK
LALMAIKDSAPFPDFPSALKEPNLHITINFKYVPKK

PPIIPRED:

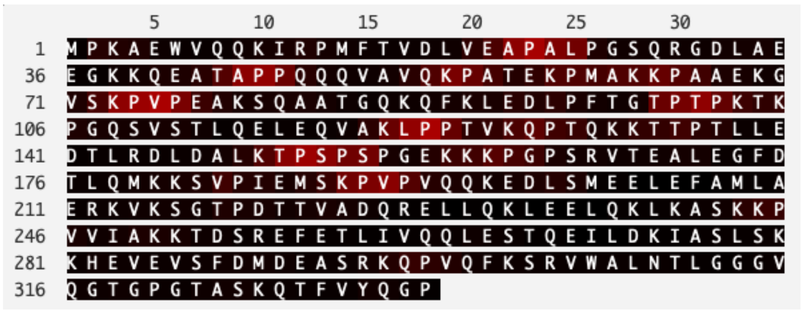

333 Domain II residues

32 Proline residues

98 α-Helix residues

Candidatus Schekmanbacteria

Candidatus Schekmanbacteria bacterium GWA2_38_9

Locus: 34083- 42001

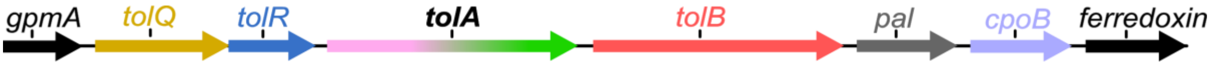

Flanked by *gpmA* encoding a phosphoglyceromutase and a truncated(?) desulfoferredoxin gene.

CpoB putative lipoprotein.

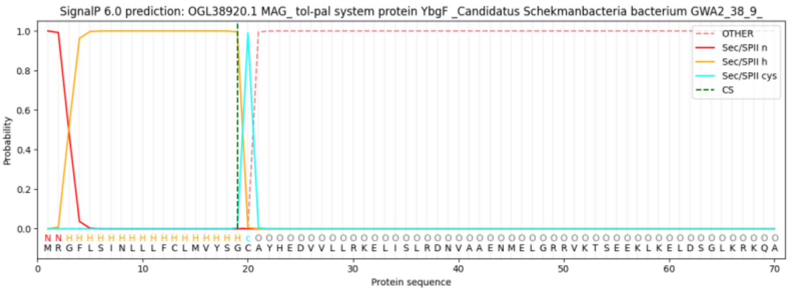

>OGL38921.1 MAG: hypothetical protein A2043_06970 [Candidatus Schekmanbacteria bacterium GWA2_38_9]

MTHLAENSFGGYAIQYEFKGLLISLLIHLVFISVLMYIPDFRKIEISSSQVISVDIVSLPRTEPASGLP
GSLVQEIKSPNPVLKKEVKTEESRSARKMAYKNENKKDKKKEKKKAEEKHEIKKEITAVSGYDEK
ADKAMEKAVEKIKQGLLAKKGDVKTGSVPGAEGAGVGYSLPGQGVSSGKTTDLRFQIYYSIIWS
KIKDSWVVPENTASADKKPEAIIAIRIKRDGEIVKVWTEKSSGNTYFDQSALRAIAKSNPLPPVPE
GYGEEYFELGIRFLPSEQ

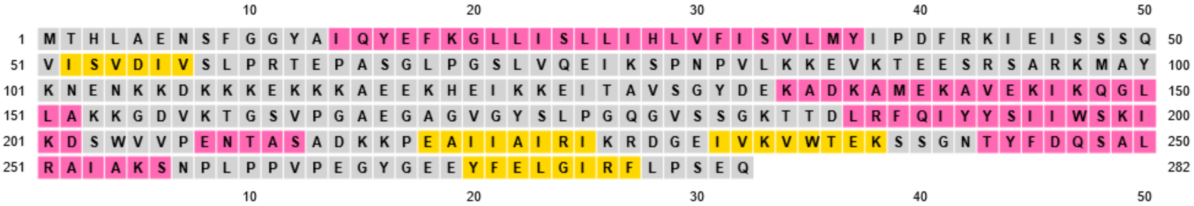

MTHLAENSFGGYAIQYEFKGLLISLLIHLVFISVLMY
**IPDFRKIEISSSQVISVDIVSLPRTEPASGLPGSLVQEIKSPNPVLKKEVKTEESRSARKMAYKNE
NKKDKKKEKKKAEEKHEIKKEITAVSGYDEKADKAMEKAVEKIKQGLLAKKGDVKTGSVPGA
EGAGVGYSLPGQGVSSG**
KTTDLRFQIYYSIIWSKIKDSWVVPENTASADKKPEAIIAIRIKRDGEIVKVWTEKSSGNTYFDQSA
LRAIAKSNPLPPVPEGYGEEYFELGIRFLPSEQ

PPIIPRED:

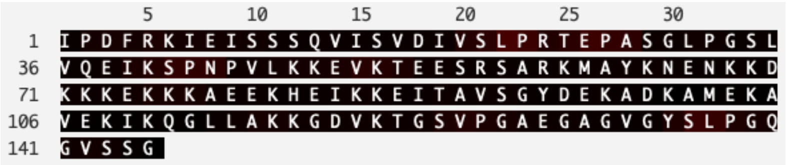

145 Domain II residues

8 Proline residues

19 α-Helix residues

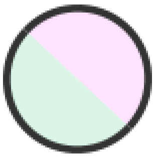

Nitrospirae

Magnetobacterium bavaricum

Locus: 11032- 17794

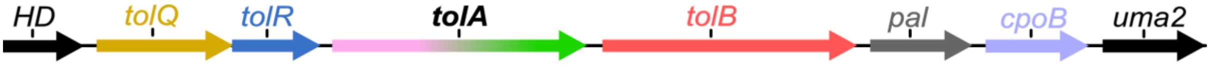

Flanked by a HD-domain phosphohydrolase gene and *uma2* restriction endonuclease gene.

CpoB putative lipoprotein.

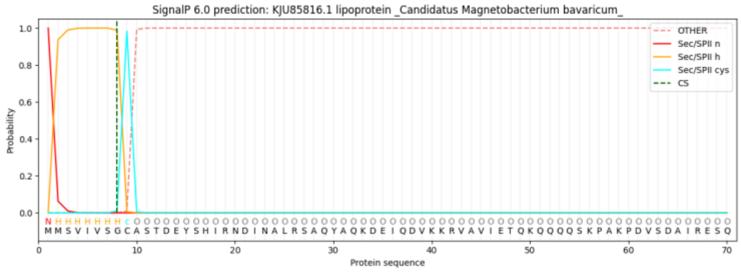

>KJU85813.1 protein containing TonB [Candidatus Magnetobacterium bavaricum]

MSRQPGISRQPGISRQPVMNMRPHIDRHPNILANTAVSFILHAVMLTLLAYTMKKQSPMLIPPAY
KVSLVSVGDGAKASQPPVREAAEPVAKPRVEKPEVQKPVAKKDPPVKKEVQAVPVPKEVPIPKK
EVPKPVAEVKKEPPKENKATPEPKVQEPKVSVEDSIAALKAKKKLQQQSKLRSTISLKADTGSSP
PQAATQRQGSTPSMYESPNGVESNDLMAQYIGLIGDLIRKRWIFPDSMKKDLEAIVFFNITKDGK
VEHVRLDKSSGNTFYDRSCLSAVNKAVPLPTPPVDNMEVAIRFYP

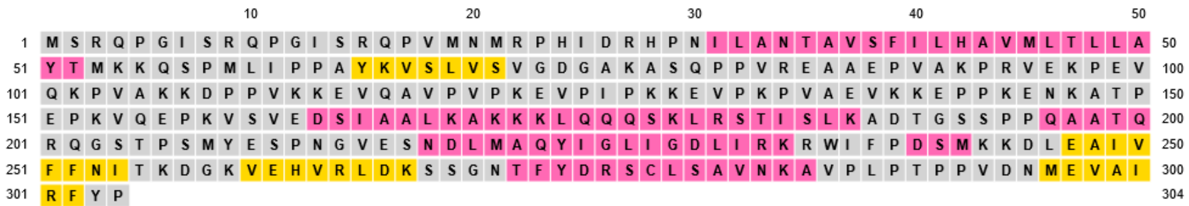

MSRQPGISRQPGISRQPVMNMRPHIDRHPNILANTAVSFILHAVMLTLLAYT
**MKKQSPMLIPPAYKVSLVSVGDGAKASQPPVREAAEPVAKPRVEKPEVQKPVAKKDPPVKKE
VQAVPVPKEVPIPKKEVPKPVAEVKKEPPKENKATPEPKVQEPKVSVEDSIAALKAKKKLQQQ
SKLRSTISLKADTGSSPPQAATQRQGS**
TPSMYESPNGVESNDLMAQYIGLIGDLIRKRWIFPDSMKKDLEAIVFFNITKDGKVEHVRLDKSS
GNTFYDRSCLSAVNKAVPLPTPPVDNMEVAIRFYP

PPIIPRED:

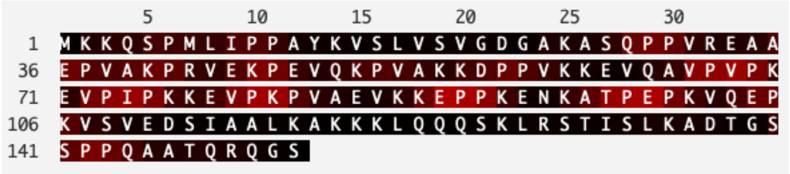

152 Domain II residues

24 Proline residues

20 α-Helix residues

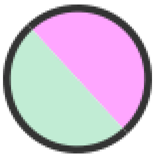

Candidatus lambdaproteobacteria

Candidatus Lambdaproteobacteria bacterium RIFOXYD2_FULL_50_16

Split operon. Sequences are not fully assembled-links to separate regions.

Loci: *tolQRAB-cpoB:* 17651

murE tolQ tolR tolA tolB cpoB nit

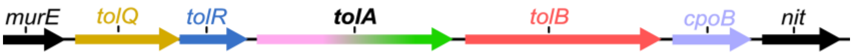

Flanked by an alanine racemase gene and *nit*

pal unidentified

>OGG96180.1 MAG: hypothetical protein A2527_04575 [Candidatus Lambdaproteobacteria bacterium RIFOXYD2_FULL_50_16]

MISWQGYGQSLFFHLGVLGLIWVLYQQEPPAPLFEIASEVFFEEAALAPNPDPRPAPVVAPTPAV
PAPVLPPTPKKEADAIRSAKLKSTNPKPVPPKKAEASPKAEGSPLPGSNATSIAAPSPPVETTST
SEPTGAGGAPAGTGLLKNQLNKRSYQSLLKQIVQAHWETPPVEADYRILVRCTITPDGRIDDLEI
KEPSGFELLDQAAKKAILTSQPLPPPPREFLVNGSYEAWFRFSPEEG

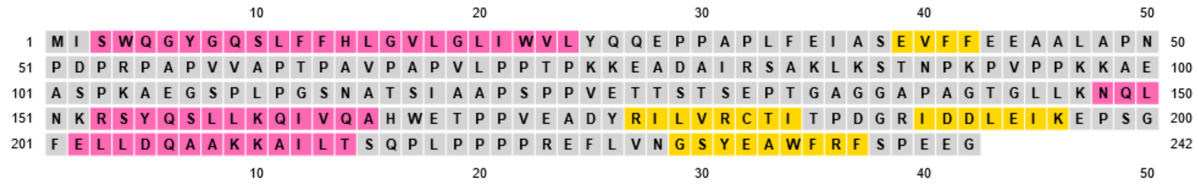

MISWQGYGQSLFFHLGVLGLIWVL
**YQQEPPAPLFEIASEVFFEEAALAPNPDPRPAPVVAPTPAVPAPVLPPTPKKEADAIRSAKLKS
TNPKPVPPKKAEASPKAEGSPLPGSNATSIAAPSPPVETTSTSEPTGAGGAPAG**
TGLLKNQLNKRSYQSLLKQIVQAHWETPPVEADYRILVRCTITPDGRIDDLEIKEPSGFELLDQAA
KKAILTSQPLPPPPREFLVNGSYEAWFRFSPEEG

PPIIPRED:

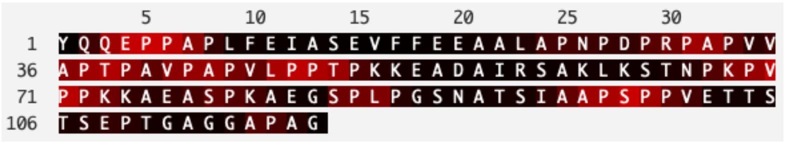

118 Domain II residues

27 Proline residues

0 α-helix residues

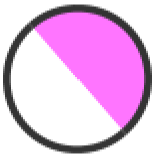

Candidatus Dadabacteria

Candidatus Dadabacteria bacterium RIFCSPHIGHO2_12_FULL_53_21 WGS

tolB   *aminomutase-pal-cpoB-pal-????-rutF*

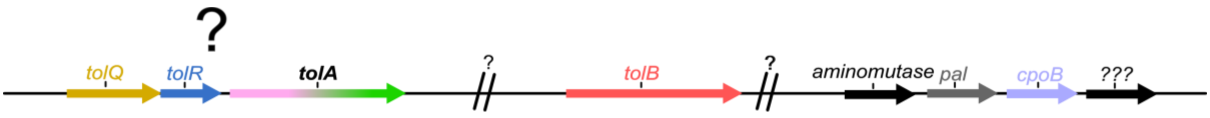

Second Pal has a helical bundle as observed in *Ca. calescamentes*

Candidatus Dadabacteria bacterium isolate J035 k99_373966

Accession

Via HMM search of Witwinowski database found another cluster featuring *tolB-pal-cpoB*

tolQRA not found nearby, suggests a split operon

Oligoflexia

See *Bdellovibrio bacteriovorus* (page 84).

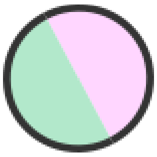

Candidatus Poribacteria

Candidatus Poribacteria bacterium

Split operon- TolQR assumed elsewhere.

Locus:

>MBI5116564.1 MAG: TonB C-terminal domain-containing protein [Candidatus Poribacteria bacterium]

MSRMLMYSVVFHLCLITSFMMARALNLSFRKLPIIYSVDLVDMSRPGAAMAKQVSSVIAKKLEQP
KPKPVKETKKAIQKPPDKKQVALASQKKVETPKKEKTPKPKEKPDEAAMSAAPPKKEEPVESEE
EPVRPEPKQVASKADQDFVTSGDPGDVVGPGNVSKDLINPELRWYIEIIRRKVWQNWIEPRHVL
TDGVHARVVIRFEIGRDGGFVAPPEVFETSNVPLVDQSGYRAVLRAAPFPPLPESYRGNIMGIRF
GFEYGENA

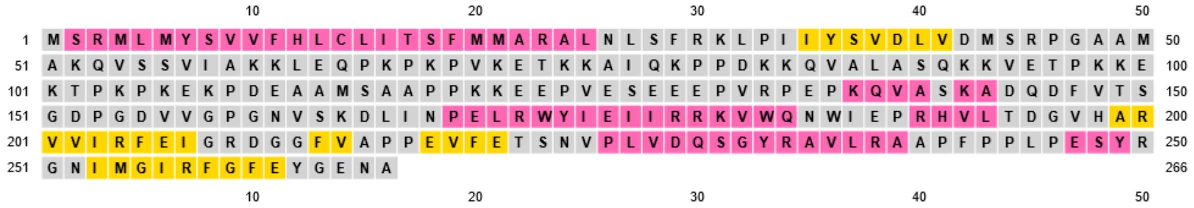

MSRMLMYSVVFHLCLITSFMMARAL
**NLSFRKLPIIYSVDLVDMSRPGAAMAKQVSSVIAKKLEQPKPKPVKETKKAIQKPPDKKQVAL
ASQKKVETPKKEKTPKPKEKPDEAAMSAAPPKKEEPVESEEEPVRPEPKQVASKADQDFVTS
GDPGDVVGPGNVSKDL**
INPELRWYIEIIRRKVWQNWIEPRHVLTDGVHARVVIRFEIGRDGGFVAPPEVFETSNVPLVDQS
GYRAVLRAAPFPPLPESYRGNIMGIRFGFEYGENA

PPIIPRED:

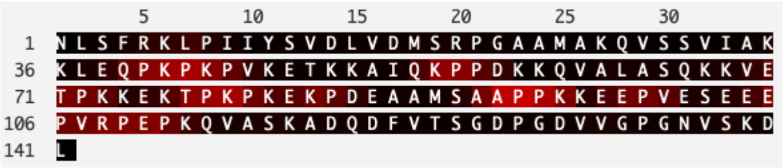

141 Domain II residues

19 Proline residues

6 α-helix residues

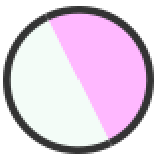

Candidatus Sumerlaeota

Candidatus Sumerlaea chitinivorans

acnA- *tolB*- *o-GlcNAc-transferase*

pal

A TPR gene was found nearby but does not appear to be CpoB based on AF3 prediction.

Planctomycetes

Witwinowski et *al.* showed no *pal* in this taxon.

Aquisphaera giovannonii strain OJF2

Detected possible *tolB* on chromosome with mostly appropriate predicted structure.

Flanked by DUF1501 and an S-layer gene.

Candidatus Ratteibacteria

Witwinowski showed no *lol* or *pal* in this taxon.

Detected *tolB* on with appropriate predicted structure.

Next to *tolR* and *tolA* and *pal*, but poorly assembled, short read. Unknown if *tolQ* or *cpoB* are adjacent.

***tolQRAB-pal* assumed, but not proven.** Nearest homologue is *Candidatus Omnitrophica* (98% conservation of ExbD), which has *tolQ* next to *tolR*.

>PIX77505.1 MAG: hypothetical protein COZ37_02335 [bacterium (Candidatus Ratteibacteria) CG_4_10_14_3_um_filter_41_18]

MSCLWGEREIMGKSFFISTLIHSLLIGVLFFNPAMRRNFYPGDRIQPVYLVKEPNISLGEEKEKAT
FIRRRVVLSSYSPLELKSLKEKILEKYSDTEEKKKSPEEKIEKKAREEEPAPPMSKKKASLLFLSPF
PYPWYVTLLKNKIYTNWSPPSKFSILQEGAFSAFSFRIFKDGSIEKIRLKESSNVEILDQSARKSIE
MIRDLPSLPDDWKEEYLDVTVRFSIEE

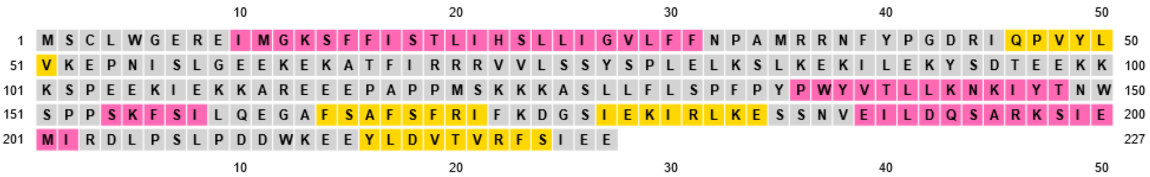

MSCLWGEREIMGKSFFISTLIHSLLIGVLFF
**NPAMRRNFYPGDRIQPVYLVKEPNISLGEEKEKATFIRRRVVLSSYSPLELKSLKEKILEKYSDT
EEKKKSPEEKIEKKAREEEPAPPMSKKKASL**
LFLSPFPYPWYVTLLKNKIYTNWSPPSKFSILQEGAFSAFSFRIFKDGSIEKIRLKESSNVEILDQS
ARKSIEMIRDLPSLPDDWKEEYLDVTVRFSIEE

PPIIPRED:

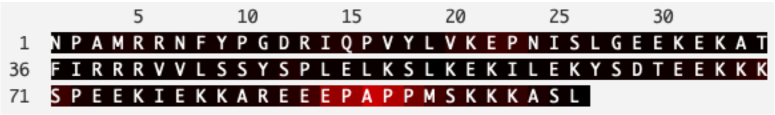

96 Domain II residues

9 Proline residues

0 α-helix residues

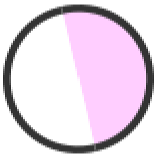

Candidatus Hydrogendentes

Candidatus Hydrogenedentes bacterium

-*tolB* hits accession shows nucleotides only

Candidatus Hydrogenedentes bacterium CG1_02_42_14

Locus:

???-tolB-*TPR*   *pal nearby but untranslated*

Candidatus Hydrogenedentes bacterium UBA2224

-*tolB* hits accession shows nucleotides only

Candidatus Hydrogenedentes bacterium UBA6118

-*tolB* hits accession shows nucleotides only

Hydrogenedentes bacterium JGI 0000039-J10

Candidatus Hydrogenedentes bacterium ADurb.Bin170 BWY07_contig000012

Locus: 19659-20993

Found a *tolB*, structure verified by Alphafold

Flanked by pilin and protease genes.

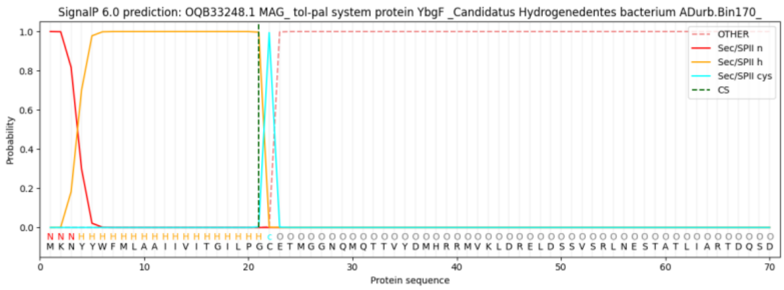

cpoB-*pal* on different locus (42156-43795).

CpoB putative lipoprotein.

No adjacent *tolQRA*

Candidatus Abyssubacteria

Locus:1- 4941

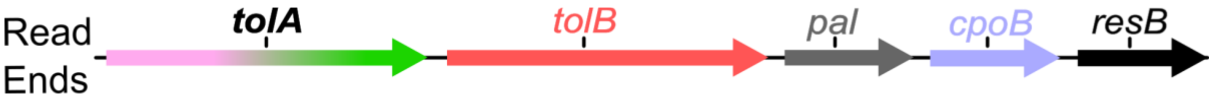

tolB- predicted structure correct

Read ends before Motor, assumed present.

Flanked by *resB*

CpoB plausible lipoprotein.

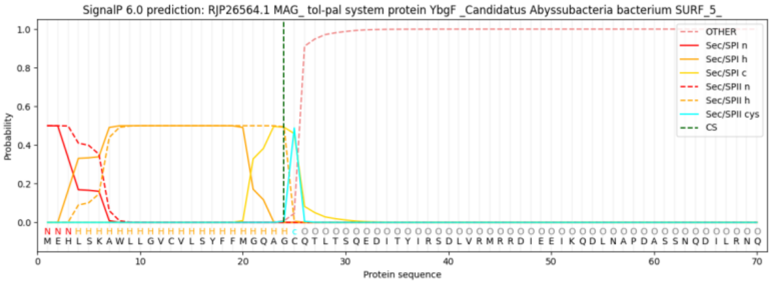

>RJP26562.1 MAG: TonB family protein [Candidatus Abyssubacteria bacterium SURF_5]

MLMYSVLIHVCLALTFVFVKVLNLPFRKPFQPVVYTVDLVEFEKKKPQKKAKAAQVAAKKEEPKP
KPKPEPKPEPKPEPKPEPKPVVEKKAAPPPPQKKIEAPKVVKAPEPKQEPPEVKAPIEPAKEEAP
PPPPQAEPTEVAKGEPEPVAESTVDLDAEYITPELKWYIEIIRRKVWQNWIEPLHALRLGTSARV
VIRFEIGRDGSLVSEPVVFESSNIFELDQSGYRAVLRSAPFPPLPEGYTGTSLGVRFGFAYGESA

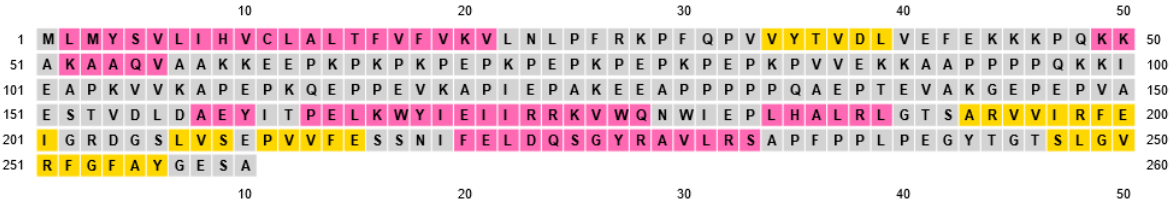

MLMYSVLIHVCLALTFVFVKV
**LNLPFRKPFQPVVYTVDLVEFEKKKPQKKAKAAQVAAKKEEPKPKPKPEPKPEPKPEPKPEP
KPVVEKKAAPPPPQKKIEAPKVVKAPEPKQEPPEVKAPIEPAKEEAPPPPPQAEPTEVAKGEP
EPVAESTVDLDAEY**
ITPELKWYIEIIRRKVWQNWIEPLHALRLGTSARVVIRFEIGRDGSLVSEPVVFESSNIFELDQSGY
RAVLRSAPFPPLPEGYTGTSLGVRFGFAYGESA

PPIIPRED:

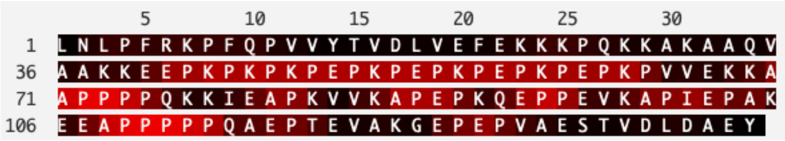

135 Domain II residues

39 Proline residues

10 α-Helix residues

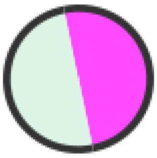

Candidatus Omnitrophica

Candidatus Hinthialibacteria bacterium OLB16

Another species, confirmed by Witwinowski et al to have pal.

Locus: 49956 – 58423

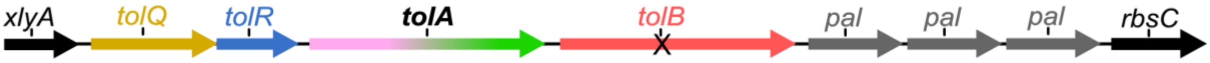

Flanked by *xlyA* and *rbsC*. Features three *pal* genes.

TolB looks to be broken into two parts, the NTD rossman fold and CTD β-propeller
One Two

>KXK40111.1 MAG: Gram-negative bacterial tonB protein [Candidatus Hinthialibacteria bacterium OLB16]

MRRYIALSAFLHLLVFVGFYLAGMLITSGDPPPAPMIGIYFPPSGEEEIQEEPTPRETESPEKTKK
ATPSPMPTDARAIVGDSEPTVSPTATPSPTRTRKPSATPSATATPLDPTSTPTVTTTRTETAPTD
TPTPKPTKEKPTETPKATLTPKPSKTPKPSATPKPSATPKATEAPTEQPTPSPNPTKEAIAKAAKE
LAKAIQNAGEKPQVPQPVKREGLPTGAPGGAPGGVPWGVPGGTGSGDGGLRTSVPFGDMMF
LGRLRDVIQENFHAPRVTRDQGKRVAVVYFKILKNGDIIDISVVESSGHKGIDRAAVKAVEAVRPF
DPLPPGTSDLGVTCDFVAE

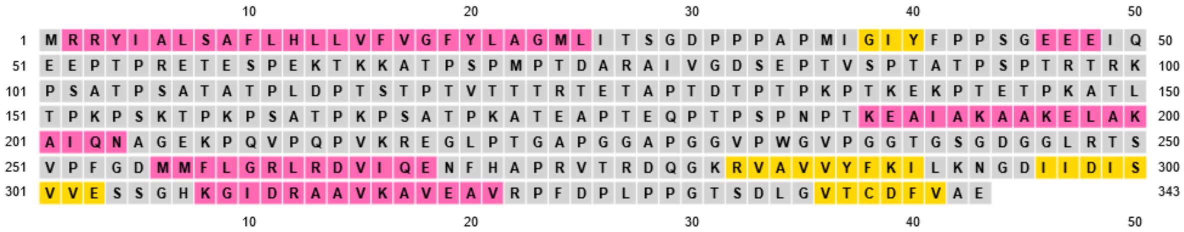

MRRYIALSAFLHLLVFVGFYLAGML
**ITSGDPPPAPMIGIYFPPSGEEEIQEEPTPRETESPEKTKKATPSPMPTDARAIVGDSEPTVSPT
ATPSPTRTRKPSATPSATATPLDPTSTPTVTTTRTETAPTDTPTPKPTKEKPTETPKATLTPKPS
KTPKPSATPKPSATPKATEAPTEQPTPSPNPTKEAIAKAAKELAKAIQNAGEKPQVPQPVKRE
GLPTGAPGGAPGGVPWGVPGGTGSG**
DGGLRTSVPFGDMMFLGRLRDVIQENFHAPRVTRDQGKRVAVVYFKILKNGDIIDISVVESSGHK
GIDRAAVKAVEAVRPFDPLPPGTSDLGVTCDFVAE

PPIIPRED:

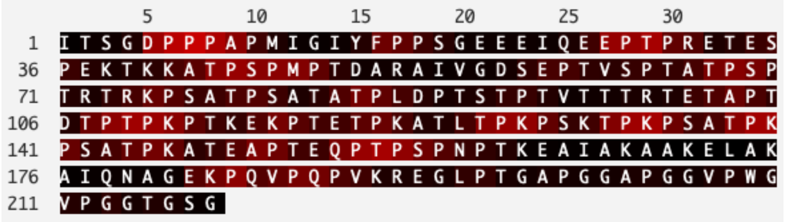

218 Domain II residues

47 Proline residues

20 α-Helix residues

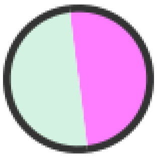

Chlamydiae

Chlamydia trachomatis

Locus: 993233-999726

tolQ-tolR-tolA-tolB-pal

Flanked by *dsdD* and *lysM*.

No CpoB detected by BLAST.

>CAP07252.1 histone H1-I [Chlamydia trachomatis L2b/UCH-1/proctitis]

MPKFQYAPFLCASIIIHIALGGMLFFSAPQKKKPRLSPFKERIVALPPEPKITTTLQTPSPQPIRKPV
KNAPAPEKKAAKPPAISNPQKSPQKPNKASPTPRNETLEKKQATLKKLAQLANQLAEEAETQES
HIAQFSWPAQAQVLTENTSYQQDAFCALFQQYVSLPFPGEVRLKLEFSSEGALLHCSILSTISHA
DKQHILNQIQKIPFQSFFSAYKTSKNIVFHIRLQGNSA

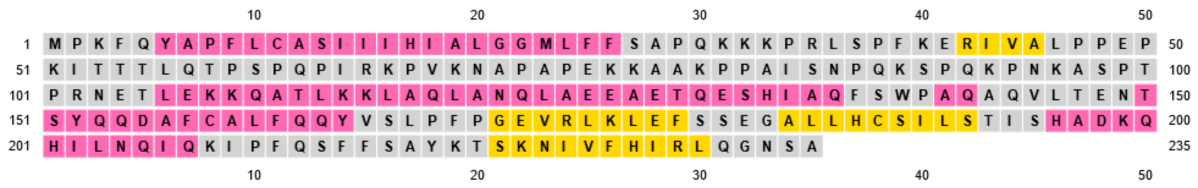

MPKFQYAPFLCASIIIHIALGGMLF
FSAPQKKKPRLSPFKERIVALPPEPKITTTLQTPSPQPIRKPVKNAPAPEKKAAKPPAISNPQK
SPQKPNKASPTPRNETLEKKQATLKKLAQLANQLAEEAETQESHIA
QFSWPAQAQVLTENTSYQQDAFCALFQQYVSLPFPGEVRLKLEFSSEGALLHCSILSTISHADK
QHILNQIQKIPFQSFFSAYKTSKNIVFHIRLQGNSA

PPIIPRED:

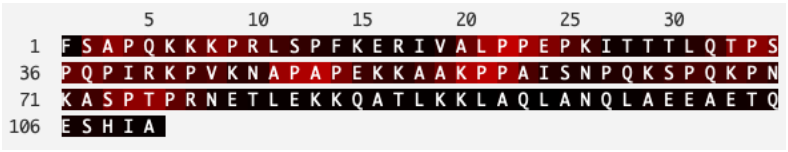

110 Domain II residues

19 Proline residues

30 α-Helix residues

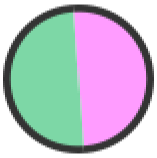

Candidatus Aureabacteria

Auribacter fodinae

Found a [tol-pal operon]

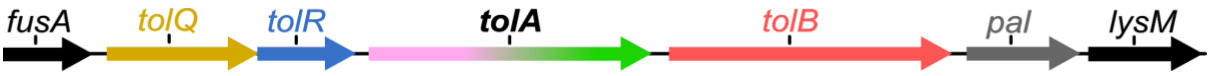

Unclear if TolA- No pal or TolB Nearby but there is a TPR

>RJP56484.1 MAG: TonB C-terminal domain-containing protein [Candidatus Auribacter fodinae]- **looks like split/broken TolA**

MKKTSTQAPQTVLLYYAGLVKKRVYAQWAIPGTLRGQEQIDIVTIKIRILNTGTVSEMEFVSDTDN VLLKKSIIDAIDNSKPFPPFPSDLHEESLDIIINFDTQQ

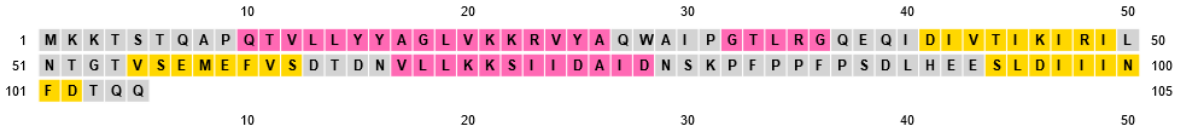

Also hypothetical protein:

MSKLDSLLLLFKQRLLVLFKQRKFLVISISCHIIIITAVVIMLPLWKQKKSEPIKIQEVELFTPQPQEQ EKPADKKEPQTQEKAAEKEPEPEKPLVEQKVIKRPPPEPLNSNLENNILKQWDDLEKKK

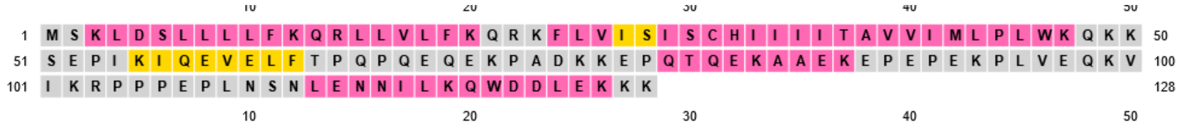

[Locus]:

Flanked by *mob* and PQQ gene.

Verrucomicrobiota

Chthoniobacter flavus

Locus: 333 - 6332

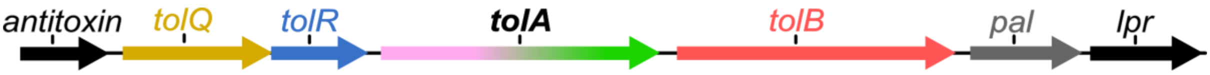

Flanked by an unknown antitoxin gene and an outer membrane *lpr* carrier.

>TCO86909.1 TonB family protein [Chthoniobacter flavus]

MPPDRSFRRNFFVIASLHVVAVGVLYFLGTFHRKPPAEQVMWLEGGSVGGGEPGAGEAAPDP
QPSPTQAEYKSESPPPPPEPKPELIPPPPVVKQAPSELVTPQTTPEPATPKPHTPKPETPKPHTP
KPETPKPETPKPHTPKPHTPHPTPKHTPKPATPKATPKEKDEGDDEATPKPKATSSEKEKEKEK
ETAREKPKGSPGPSKSEGTSTAMNKSTGSGGNGEGKGNGKGPGKAGNGSGASEFGWYFSMI
HDRFHSRWDQPTSIAHGGQDIVTTLKIRIAKDGTIQSREILHSSGDNTMDQSVMTAAERVLQIDP
LPAGLGNGESFEINIAFKLDQGQ

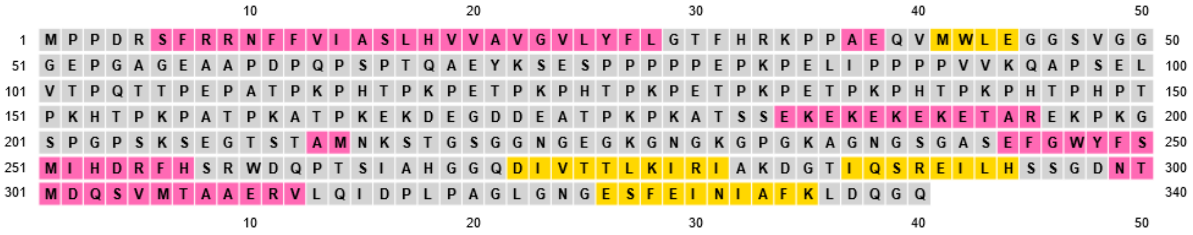

MPPDRSFRRNFFVIASLHVVAVGVLYFL
**GTFHRKPPAEQVMWLEGGSVGGGEPGAGEAAPDPQPSPTQAEYKSESPPPPPEPKPELIPPP
PVVKQAPSELVTPQTTPEPATPKPHTPKPETPKPHTPKPETPKPETPKPHTPKPHTPHPTPKH
TPKPATPKATPKEKDEGDDEATPKPKATSSEKEKEKEKETAREKPKGSPGPSKSEGTSTAMN
KSTGSGGNGEGKGNGKGPGKAGNGS**
GASEFGWYFSMIHDRFHSRWDQPTSIAHGGQDIVTTLKIRIAKDGTIQSREILHSSGDNTMDQSV
MTAAERVLQIDPLPAGLGNGESFEINIAFKLDQGQ

PPIIPRED:

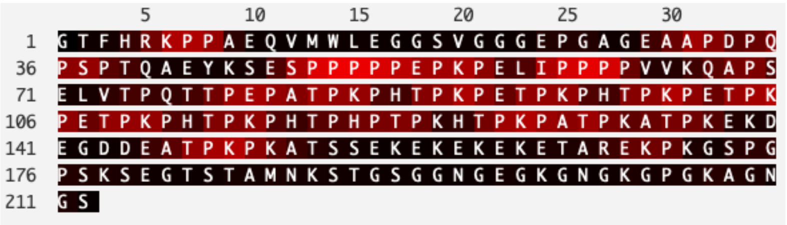

212 Domain II residues

49 Proline residues

16 α-Helix residues

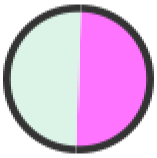

Lentisphaerae

Victivallis vadensis

Split operon.

Loci:

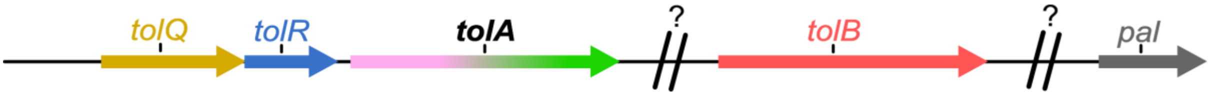

tolQ-*tolR*-*tolA*   *tolB*   *pal*

TolQ structure prediction has long cytoplasmic tail, likely forms polyproline helices.

TolB structure predicted correctly.

>NMD86568.1 cell envelope integrity protein TolA [Victivallis vadensis]

MSRLSPPRPGSSGKHLPPVPTGRQWKIFVPVSLLHVLLIFGPLAYLGWRGAHQEENAFRVKIGG
AELSHDWNVGKPERLRPGANPGRLPDPPPTEPDTPEPPPQEPARPDEPGIDVVRLQQQREAR
KRAAAEAAKKKAAAEAAKKKAAAEAARKKAAEAAKKKAAEAARKRAAEAARKKAAAEAAKRKA
AAEAARKKAEAEARRRRELEGVYNPNRTGPIGDGSGRNTNQNVPIGSQDRGQAYGKPDYRTP
GGGASSEIPDSYFNGLKRYFDARWQQPPRSLLGNNDPKVVVELTVSDTGKVLASRIISRSGINA
MDESVKRLLDNLDLVPRPPSQVTFQLVLKVE

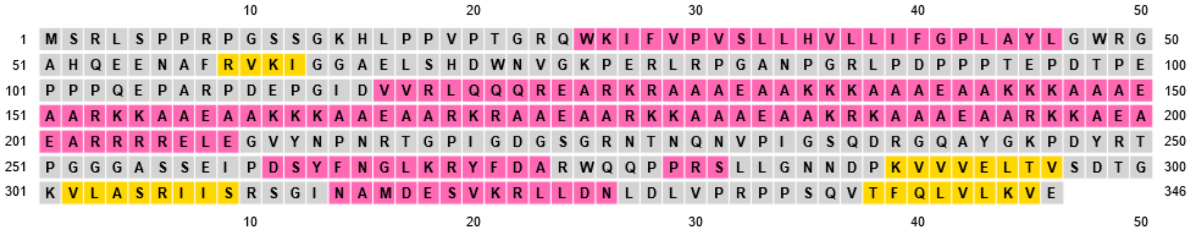

MSRLSPPRPGSSGKHLPPVPTGRQWKIFVPVSLLHVLLIFGPLAYL
**GWRGAHQEENAFRVKIGGAELSHDWNVGKPERLRPGANPGRLPDPPPTEPDTPEPPPQEPA
RPDEPGIDVVRLQQQREARKRAAAEAAKKKAAAEAAKKKAAAEAARKKAAEAAKKKAAEA
ARKRAAEAARKKAAAEAAKRKAAAEAARKKAEAEARRRRELEGVYNPNRTGPIGDGSGRNT
NQNVPIGSQDRGQAYGKP**
DYRTPGGGASSEIPDSYFNGLKRYFDARWQQPPRSLLGNNDPKVVVELTVSDTGKVLASRIISR
SGINAMDESVKRLLDNLDLVPRPPSQVTFQLVLKVE

PPIIPRED:

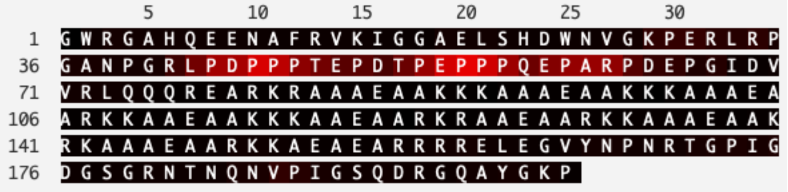

TolA phylogeny suggests acquired from Proteobacteria via HGT.

200 Domain II residues

19 Proline residues

94 α-helix residues

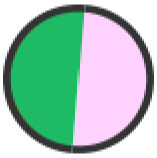

Lentisphaerae bacterium RIFOXYA12_FULL_48_11

Split operon.

Loci: *tolQ*-*tolR*-*???*- No TolA found

vacB-*tolB*-*pal*-*tatA* 25489- 30826

Structures confirmed: TolB   Pal

Could not proceed without TolA so used *V. vadensis* TolA for analysis

Kiritimatiellota

Tichowtungia aerotolerans

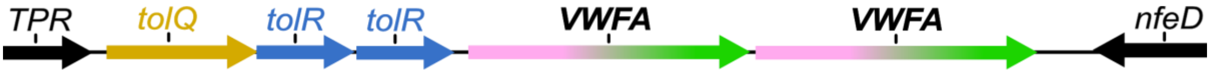

Found what may be an unusual motor-transducer locus featuring two force transducers which possess an entirely different C-terminal domain. Transducer 1 Transducer 2. May be evidence of monomeric force transduction for a third system that is not OM-stabilisation or substrate import, such as gliding motility *transducer 1* *transducer 2*

The transducers feature a Von Willebrand factor A (VWFA) CTD, which are often involved in Protein-protein interactions.

This locus also features two heterologous TolR-like subunits.

Transducer structures:

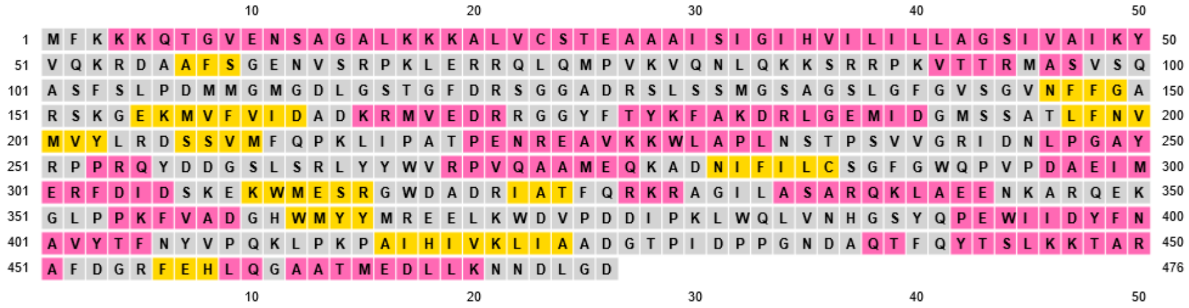

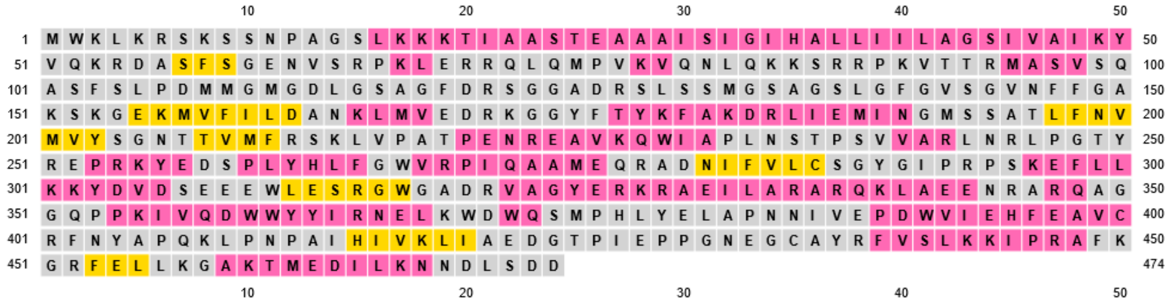

Both are predicted to have a stator box (~ residues 55-60) suggesting they use the motor in the same way as TolA/TonB.

Use of FoldSeek and BLAST revealed that this is a family of force-transducer previously unidentified.

Kiritimatiella glycovorans

Genome.

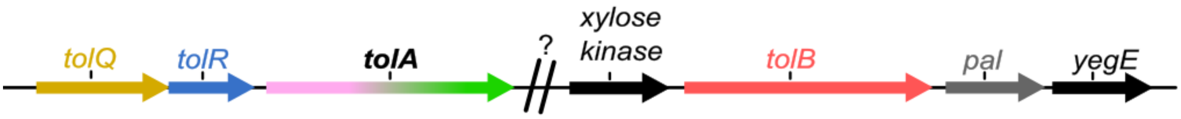

Xylose kinase – *tolB* – *pal* - *yegE*

TolB structure correct.

Split operon, unable to annotate TolA specifically.

Kiritimatiellaceae bacterium

Pontiella desulfatans

Candidadatus Goldbacteria

No *pal* according to Witwinowski et al.

Candidatus Goldbacteria bacterium HGW-Goldbacteria-1

Features Omp85-TolB fusion protein.

No recognisable “correct” TolB proteins.

Candidadatus Firestonebacteria

No *pal* according to Witwinowski et al.

Candidatus Firestonebacteria bacterium

No structural predictions available in UniProt.

Has a Omp85-TolB fusion protein.

Candidadatus Hydrothermota

No *pal* according to Witwinowski et al.

Candidatus Hydrothermota bacterium

Omp85-TolB fusion predicted.

S41-peptidase- looks like TolB but with wrong NTD.

Has an OmpA with what looks like carboxyphosphatase domains in it (prediction).

Candidatus Coatesbacteria

Candidatus Coatesbacteria bacterium

Seems to have split loci, unable to determine which transducer is *tolA.*

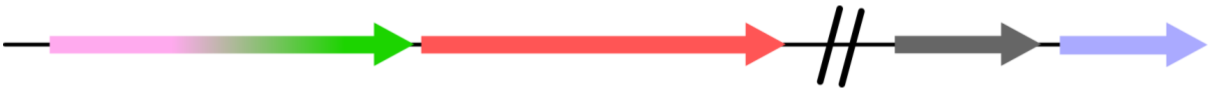

Loci: *tolQ*-*tolA*- *cheY*-*tolA*-*tolB*-*cmk* *3-isopropylmalate dehydratase*-*pal*-*cpoB*

TolB correct structure.

cpoB was also detected next to *pal*, with expected structural prediction.

CpoB putative lipoprotein.

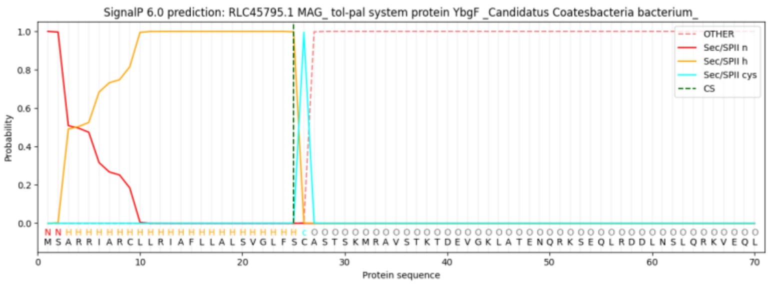

>RLC47582.1 MAG: hypothetical protein DRH70_03045 [Candidatus Coatesbacteria bacterium]

MSSSKPVTKMRPNKPGASFVVLSLVVHLTAASLLLSATLSRHQRAPVPSELRVRLAAAVVHQPN
EASPAEMLPRASEPDRLQAPKPAESKHAAKVKSSAKSELQKARPKPHPIRHKAARVSTPMSVD
GQRFGYPYYLNVVKSKLAEAWLYPSDAIAGKRSLRARVAFYIHRNGGIGRVRIERCSQSPVFDR
SVLRAVEAAAPFPPLPDGYKEETLGALFVTFEYHK

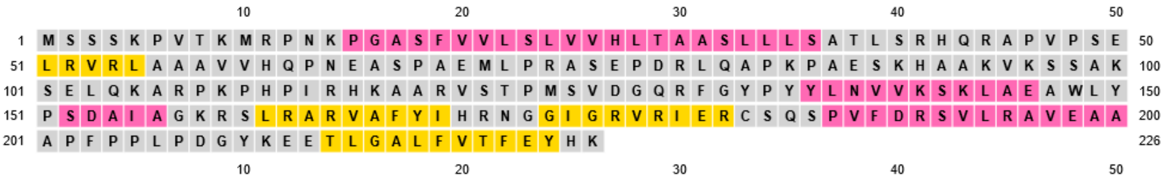

MSSSKPVTKMRPNKPGASFVVLSLVVHLTAASLLLS
ATLSRHQRAPVPSELRVRLAAAVVHQPNEASPAEMLPRASEPDRLQAPKPAESKHAAKVKS SAKSELQKARPKPHPIRHKAARVSTPMSV
DGQRFGYPYYLNVVKSKLAEAWLYPSDAIAGKRSLRARVAFYIHRNGGIGRVRIERCSQSPVFD
RSVLRAVEAAAPFPPLPDGYKEETLGALFVTFEYHK

PPIIPRED:

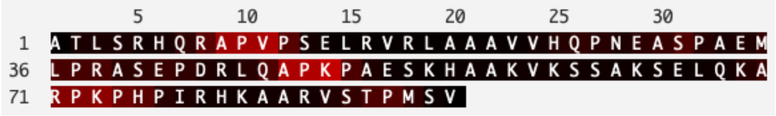

90 Domain II residues

12 Proline residues

0 α-helix residues

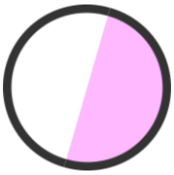

Suggests there is a tol-pal system.

Candidatus Stahlbacteria

Candidatus Stahlbacteria bacterium

Locus: *rpt1*-*pal*-*cpoB*-*tolQ*-*tolR*-*tolA*-*tolB*-*???*

TolB prediction looks correct.

CpoB putative lipoprotein.

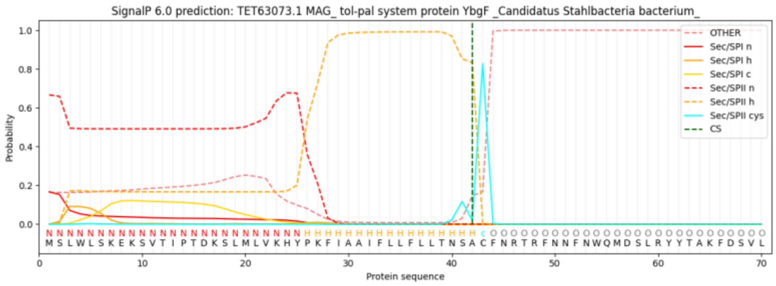

>TET63076.1 MAG: hypothetical protein E3J47_02400 [Candidatus Stahlbacteria bacterium]

MNRFYILSTSLHLIIFAVFIFANRAGFKPIPEFEVYKVSIAPLPQPKIVSMEESVEEDVKRETKTEEK
APPEQKAKSEKTKKPKAKTEKIIKKGLPDIKPKIYTGSGRGFTYSYYLNILLNKINKNWHNPFKDR
DIVLKSIVYFEVDKNGKLYNVRLEENSGNELYNETTIRAVILAKKLPPLPQEFSDDYLKVHLEFLTA
Q

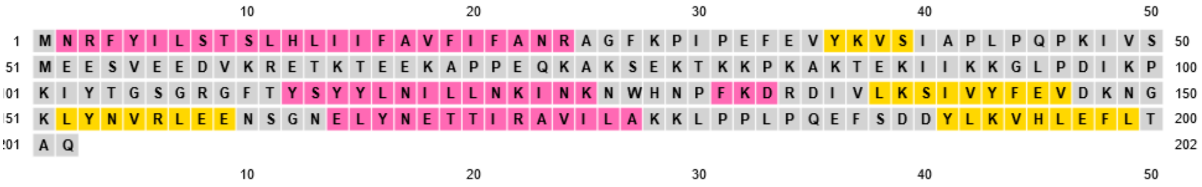

MNRFYILSTSLHLIIFAVFIFANR
AGFKPIPEFEVYKVSIAPLPQPKIVSMEESVEEDVKRETKTEEKAPPEQKAKSEKTKKPKAKTE KIIKKGLPDIKPKI
YTGSGRGFTYSYYLNILLNKINKNWHNPFKDRDIVLKSIVYFEVDKNGKLYNVRLEENSGNELYN ETTIRAVILAKKLPPLPQEFSDDYLKVHLEFLTAQ

PPIIPRED:

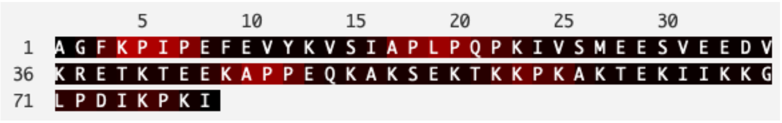

78 Domain II residues

10 Proline residues

0 α-helix residues

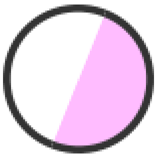

Candidatus Cloacimonetes

Candidatus Cloacimonas acidaminovorans str. Evry

Genome:

Locus: -2050023

tolQ-*tolR*-*tolA*-*agmX*

Used link to Pal from Witwinowski paper.

Looks like a Pal-TolB hybrid structure. Could be porE. Possible lipoprotein.

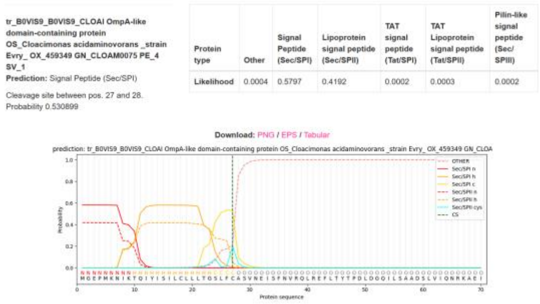

Peptidase- *pal-tolB* hybrid gene *??*

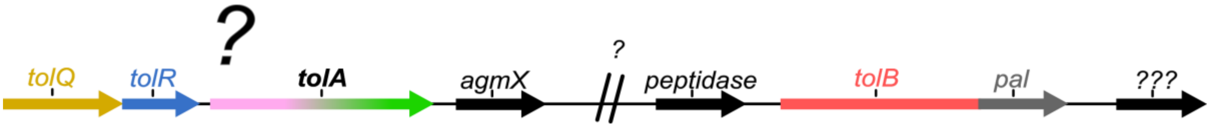

>WP_015425431.1 hypothetical protein [Candidatus Cloacimonas acidaminovorans]

MQILDKLKSSNKSERRVRKKFEEAKGLSITSLLDELTIILVFLVKNISTEVVKISAEPNITYPHTITND
KLLEKAGTTPIKIFPDRVVVGTEALEYGSPADLLTDANKRQDLLTYLKMEAADIIQHQKAETCLLV
QADYSIPCEYITEIVRAATAAGYSYIYFATLEDADWLKKYSVSLAE

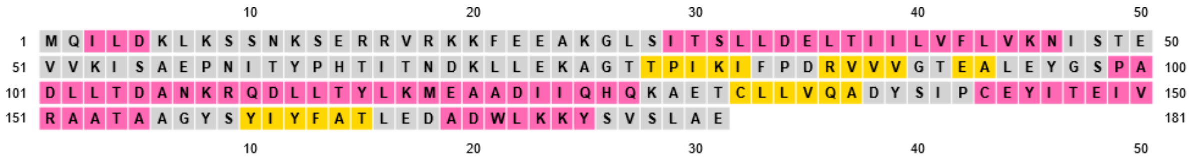

Too short to fit analysis parameters, but may be functional.

Candidatus Fermentibacter

Fermentibacter daniensis

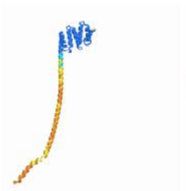

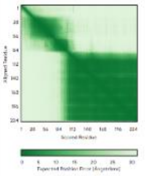

Locus: 177464- 182266

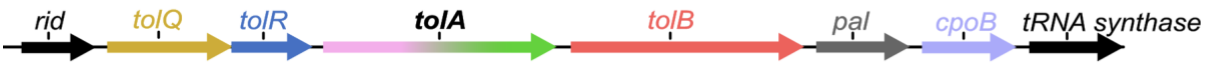

Flanked by a Rid-family hydrolase gene and tRNA synthase gene.

CpoB confirmed by AF3 prediction.

CpoB putative lipoprotein.

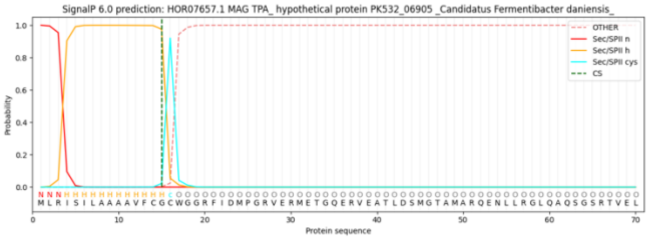

>HOR07660.1 MAG TPA: TonB C-terminal domain-containing protein [Candidatus Fermentibacter daniensis]

MNRSSLLISSAGHAAVILLALLMQGFSGSTPPLGGGDAVMVEMVTLGTPGPPPVRPDVTDRVEP
HAQVEPQDPVDEAEDVVEEPVQIEDIESPVIEEPVRIDPPVEDERDRQEEPVQPDRTQAERVDT
PGSQGGGYASVGGSGEAGGGAPGPATYEGRVFSAIRRNFRTSAVPQQSYRIEVTVRPDGSML
VDTLRKSGVDLFDRAVEHALAMAQMPPFPPGRTSPAVLRIEFLGLSEEQ

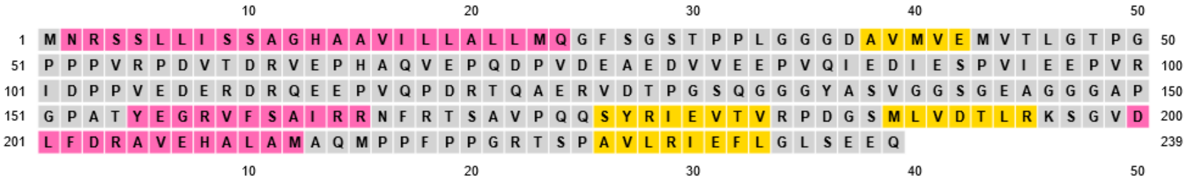

MNRSSLLISSAGHAAVILLALLMQ
**GFSGSTPPLGGGDAVMVEMVTLGTPGPPPVRPDVTDRVEPHAQVEPQDPVDEAEDVVEEPV
QIEDIESPVIEEPVRIDPPVEDERDRQEEPVQPDRTQAERVDTPGSQGGGYASV**
GGSGEAGGGAPGPATYEGRVFSAIRRNFRTSAVPQQSYRIEVTVRPDGSMLVDTLRKSGVDLF
DRAVEHALAMAQMPPFPPGRTSPAVLRIEFLGLSEEQ

PPIPRED:

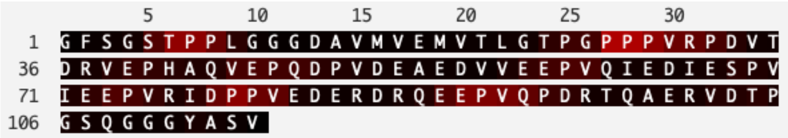

115 Domain II residues

18 Proline residues

0 α-Helix residues

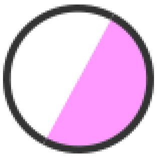

Candidatus Aegiribacteria

Candidatus Aegiribacteria sp. MLS_C scaffold_0

Locus: 96903-103271

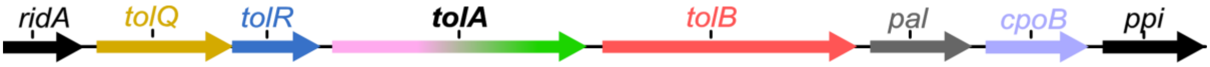

Flanked by *ridA* and a *ppi* gene

CpoB putative lipoprotein.

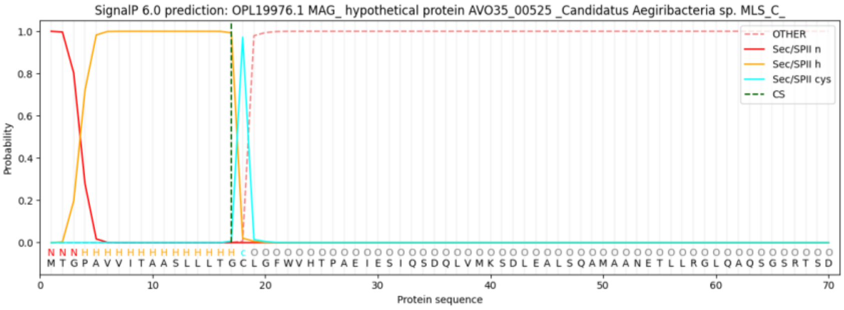

>OPL19973.1 MAG: hypothetical protein AVO35_00510 [Candidatus Aegiribacteria sp. MLS_C]

MTGFTDHEELKGRDYSPRRRDYIAAGAGHILLLLIGAFTFSGGASPLTGGEDAIMVRMVTTGTTE
VYREDATVQEEMQQNPQETQEESVIPEEVPEEIQEVPGEVQEEVPEDVQEVPEDVQEEITEDV
QEVPEDVQEVPEDLQEENDGFAAVSSMGDAGAGAPGPGTYESRVFNAVRRGYRTSVTPLQSY
RIILTVLPDGSTQVEVVRKSGTSAFDRAVENAIAMAQIPPMPPGRAAPAVINIEFLGPE

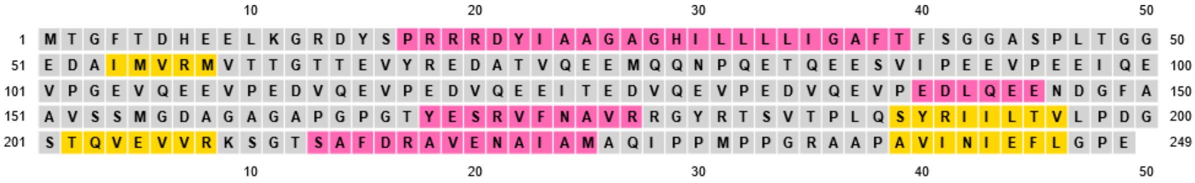

MTGFTDHEELKGRDYSPRRRDYIAAGAGHILLLLIGAFT
**FSGGASPLTGGEDAIMVRMVTTGTTEVYREDATVQEEMQQNPQETQEESVIPEEVPEEIQEVP
GEVQEEVPEDVQEVPEDVQEEITEDVQEVPEDVQEVPEDLQEENDGFA**
AVSSMGDAGAGAPGPGTYESRVFNAVRRGYRTSVTPLQSYRIILTVLPDGSTQVEVVRKSGTSA
FDRAVENAIAMAQIPPMPPGRAAPAVINIEFLGPE

PPIIPRED:

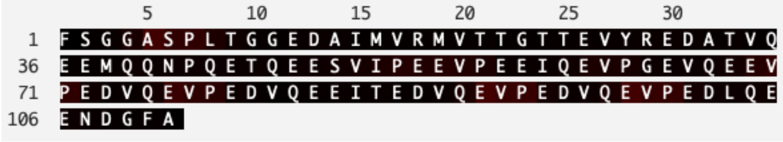

111 Domain II residues

9 Proline residues

6 α-Helix residues

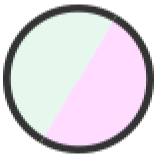

Candidatus Eisenbacteria

Candidatus Eisenbacteria bacterium

Locus:1- 6915

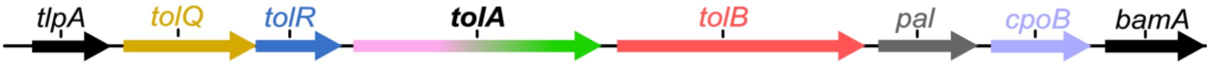

Flanked by *tlpA* and *bamA*.

CpoB probable lipoprotein.

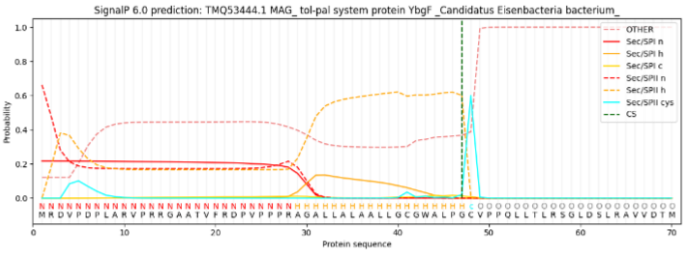

>TMQ53442.1 MAG: TonB family protein [Candidatus Eisenbacteria bacterium]

MRGSVMGSGLAHGAVLAALFALQASSPLVVPGPEVVQVALVDPTSTTVAVQPPPPKPEPEQSP
APEVQASEDVGVKLTPPKPPKRTKPKQEERAPETPAPALPYASVGNAGLKGQISVDAGDFEFTY
YLVLVRNRVAQNWMPPAGLVSAGQPVHAVVYFTIGRGGEVSAVRVESSSGVEFFDGSALRAVT
ISDPLPPLPLGFSGSSLGVHFGFEYAGP

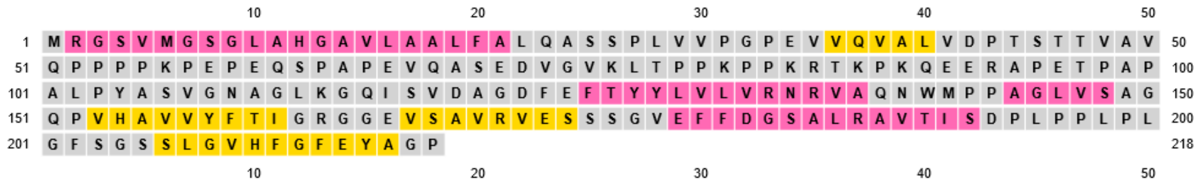

MRGSVMGSGLAHGAVLAALFA
**LQASSPLVVPGPEVVQVALVDPTSTTVAVQPPPPKPEPEQSPAPEVQASEDVGVKLTPPKPPK
RTKPKQEERAPETPAPALPYASVGNAGLKGQIS**
VDAGDFEFTYYLVLVRNRVAQNWMPPAGLVSAGQPVHAVVYFTIGRGGEVSAVRVESSSGVEF
FDGSALRAVTISDPLPPLPLGFSGSSLGVHFGFEYAGP

PPIIPRED:

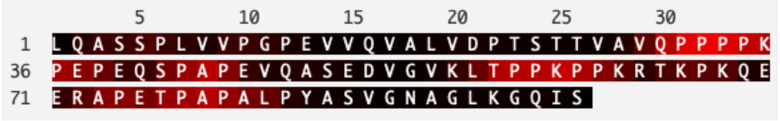

96 Domain II residues

21 Proline residues

0 α-helix residues

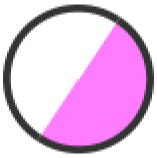

Candidatus Edwardsbacteria

Locus: 29262-35441

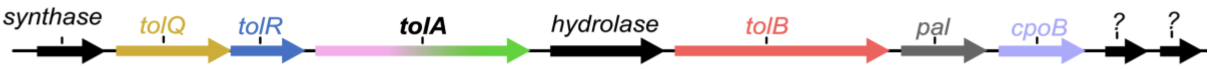

Flanked by phosphoribosylaminoimidazolesuccinocarboxamide synthase gene and short genes

Interloping hydrolase gene.

CpoB putative lipoprotein.

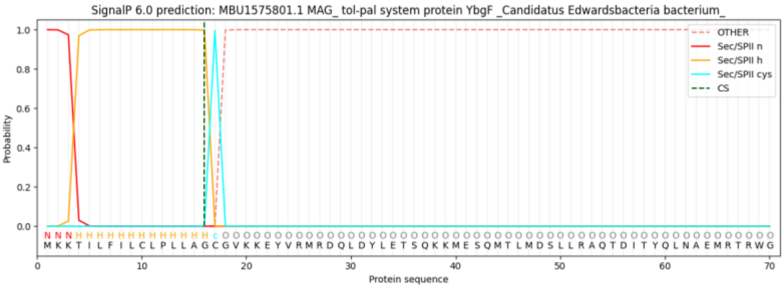

>MBU1575805.1 MAG: TonB family protein [Candidatus Edwardsbacteria bacterium]

MKREFSISLSFHLLLFLTVIFFARVLNLASQPSYPAVYQVNLVSLPQVSGSEGQDMESAPVPEAK
PSAQKEPAAPKVKPQPKAKTPDKKPKSAGGPVVPNLPQGMKVLAAEGVSPEGSYYLGMILTKIS
QHWRNPYQGEKQVIRSTIYFKIDRQGNLLEANLEKSSGDALFDQAAIRAVYQAKTFPPFPQEMK
VSVLGVHFEFEYVK

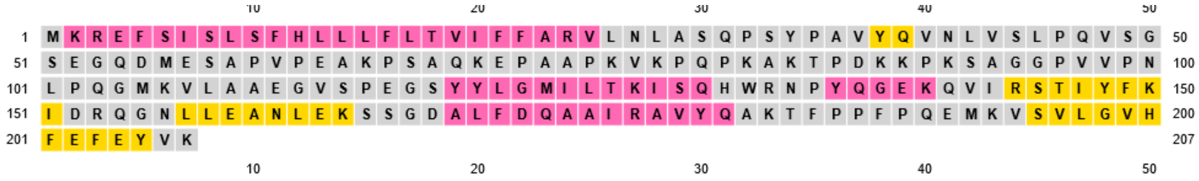

MKREFSISLSFHLLLFLTV
**IFFARVLNLASQPSYPAVYQVNLVSLPQVSGSEGQDMESAPVPEAKPSAQKEPAAPKVKPQP
KAKTPDKKPKSAGGPVVPNLPQGMKV**
LAAEGVSPEGSYYLGMILTKISQHWRNPYQGEKQVIRSTIYFKIDRQGNLLEANLEKSSGDALFD
QAAIRAVYQAKTFPPFPQEMKVSVLGVHFEFEYVK

PPIIPRED:

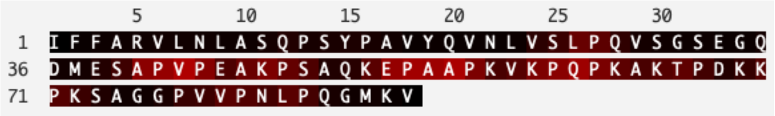

88 Domain II residues

15 Proline residues

0 α-Helix residues

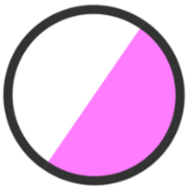

Candidatus Glassbacteria

Candidatus Glassbacteria bacterium

Locus: 31281-38198

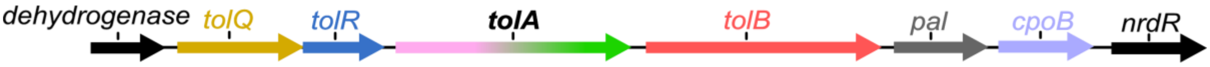

Flanked by a nucleotide sugar dehydrogenase gene and nrdR.

CpoB probable lipoprotein.

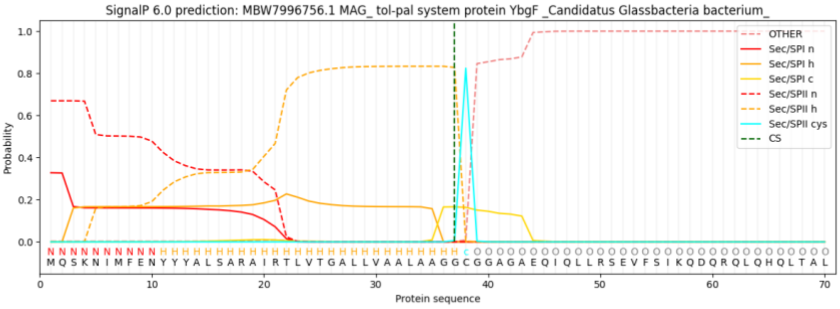

>MBW7996759.1 MAG: TonB C-terminal domain-containing protein [Candidatus Glassbacteria bacterium]

MRKAFTISIVTHVGLLLFLYYYGWERAITFAVPQVYRVQLVSMPQVQAAPVEETVPEVDVETIPP
PPEQKKKLKPKQKEAQPKAQQTERRVIQQKRPDQNLSGMRSDEQFEFLWYLRLVKDKIERNW
RNPYSGEALGTIYFCIQRNGQVTDARVEKSSGEPAFDRAALRAVINSSSFPQLPPDYKESRLTLH
IEFES

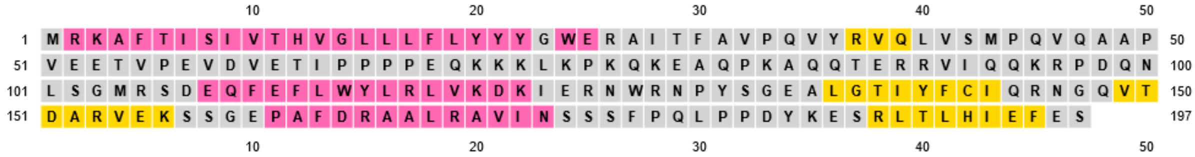

MRKAFTISIVTHVGLLLFLYY
**YGWERAITFAVPQVYRVQLVSMPQVQAAPVEETVPEVDVETIPPPPEQKKKLKPKQKEAQPK
AQQTERRVIQQKRP**
DQNLSGMRSDEQFEFLWYLRLVKDKIERNWRNPYSGEALGTIYFCIQRNGQVTDARVEKSSGE
PAFDRAALRAVINSSSFPQLPPDYKESRLTLHIEFES

PPIIPRED:

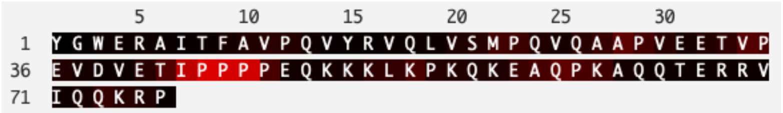

76 Domain II residues

11 Proline residues

2 α-helix residues

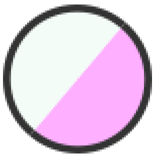

Gemmatimonadetes

Gemmatirosa kalamazoonensis

Locus: 4099599-4104931

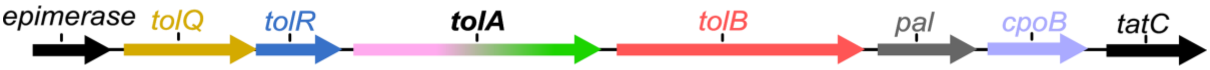

Flanked by NAD-dependent epimerase gene and *tatC*.

This species has 12 TonB paralogues

CpoB putative lipoprotein.

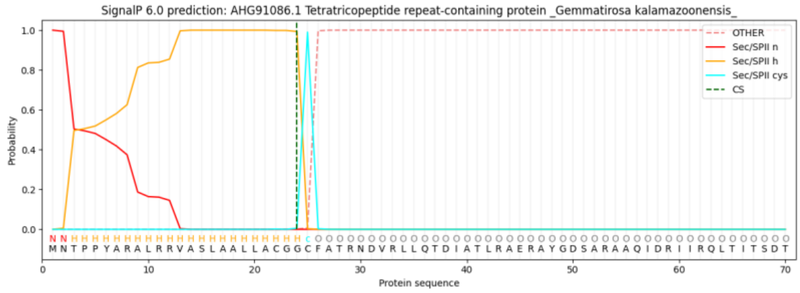

>AHG91089.1 TonB domain-containing protein [Gemmatirosa kalamazoonensis]

MSAAPAELGPSPARQRARRAASWRTPGPDAAVSKRLAGGIGVSALLHVAALAALLLHRAPPRP
AEPPVYRVDLVAAPPGPRSIGTVTPEPPAAAPAPAPTPPKATPAPTPPPRAEAPPPKAKPLPTPV
KAKPAPAKATPVPPTKANTAKAAPKTTTPPKATTPEKSTAKGTPGPRAGGGPEGGKGADVANV
HVKGLDFPFPGYLANIVRQVALVFSPPRGGAYTADVSFLIHRDGSVTDVRFVRRSGSYSFDLEA
QGAIEAVSSKRAFGPLPDEFRGDVLPVTFSFDPRVIR

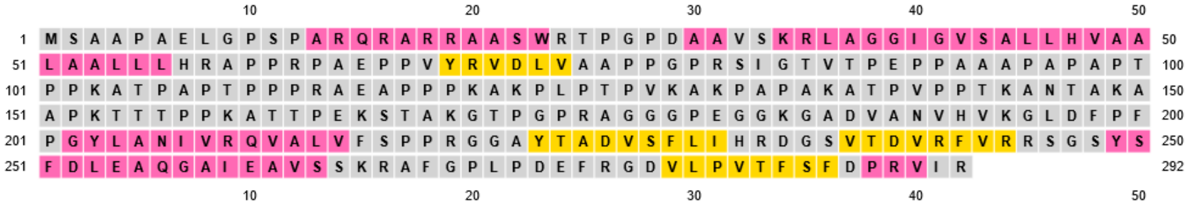

MSAAPAELGPSPARQRARRAASWRTPGPDAAVSKRLAGGIGVSALLHVAALAALLL
**HRAPPRPAEPPVYRVDLVAAPPGPRSIGTVTPEPPAAAPAPAPTPPKATPAPTPPPRAEAPPP
KAKPLPTPVKAKPAPAKATPVPPTKANTAKAAPKTTTPPKATTPEKSTAKGTPGPRAGGGPE
GGKGADVANVH**
VKGLDFPFPGYLANIVRQVALVFSPPRGGAYTADVSFLIHRDGSVTDVRFVRRSGSYSFDLEAQ
GAIEAVSSKRAFGPLPDEFRGDVLPVTFSFDPRVIR

PPIIPRED:

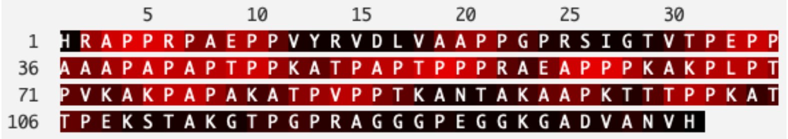

136 Domain II residues

39 Proline residues

0 α-helix residues

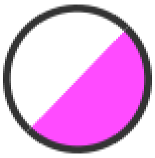

Candidate Division Zixibacteria

Candidate division Zixibacteria bacterium HGW-Zixibacteria-1

Locus: 89950-95535

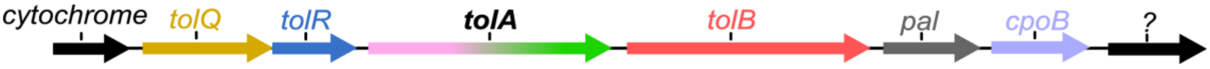

Flanked by a flavocytochrome c gene and an unknown gene.

CpoB putative lipoprotein.

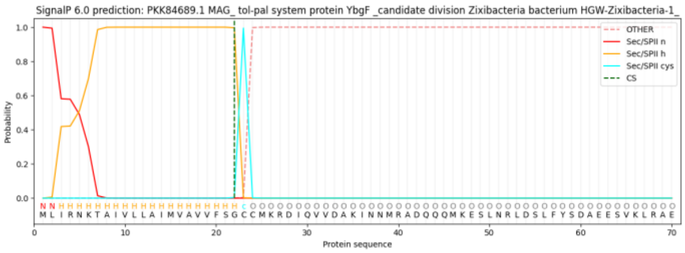

>PKK84686.1 MAG: hypothetical protein CVT49_02360 [candidate division Zixibacteria bacterium HGW-Zixibacteria-1]

MKQDLALSFLLHFLAILMLVVLTPFTPKYKIDLNDVINVRLAAMPAAQQQTEPEKLEPINIPKPIVAD
EPVAVVTETKSVTKAKPVEKPKPKPKEKPKDNAYKPKAETGTENKAGAENGQKDVSGNLGVGS
KFGGAAIDNASFDYPYWFVQAFSKIERNWTNPVYANKPISCIIYFQVIRSGRIIKTEVEKSSGVDAF
DSACERAVKLSQPLPPLPNEFTDEIIGIHLEFPYSPG

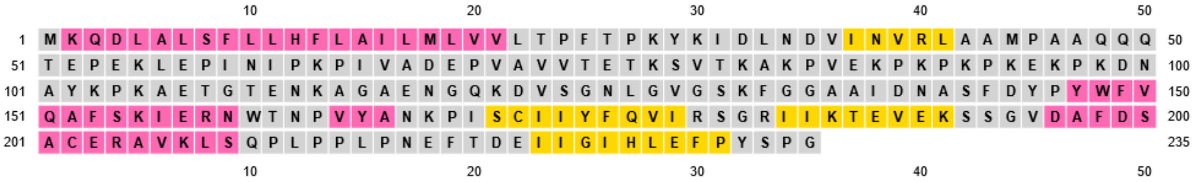

MKQDLALSFLLHFLAILMLVV
LTPFTPKYKIDLNDVINVRLAAMPAAQQQTEPEKLEPINIPKPIVADEPVAVVTETKSVTKAKPV
EKPKPKPKEKPKDNAYKPKAETGTENKAGAENGQKDVSGNLGVGSKFGG
AAIDNASFDYPYWFVQAFSKIERNWTNPVYANKPISCIIYFQVIRSGRIIKTEVEKSSGVDAFDSAC
ERAVKLSQPLPPLPNEFTDEIIGIHLEFPYSPG

PPIIPRED:

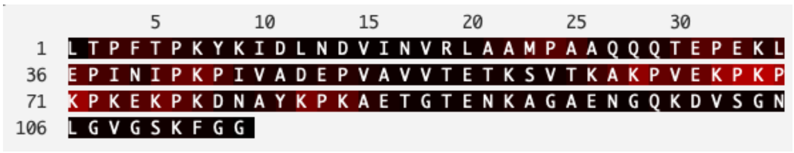

114 Domain II residues

14 Proline residues

0 α-helix residues

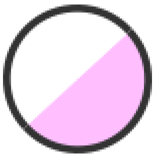

Candidatus Handelsmanbacteria

Candidatus Handelsmanbacteria bacterium RIFCSPLOWO2_12_FULL_64_10

Locus: 3177 - 9671

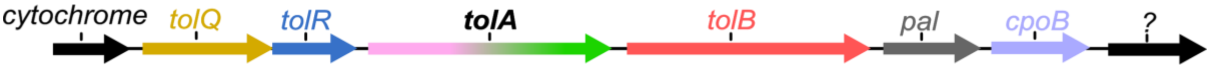

Read ends- -tolQ-tolR-tolA-tolB-pal-cpoB-mltD

CpoB putative lipoprotein.

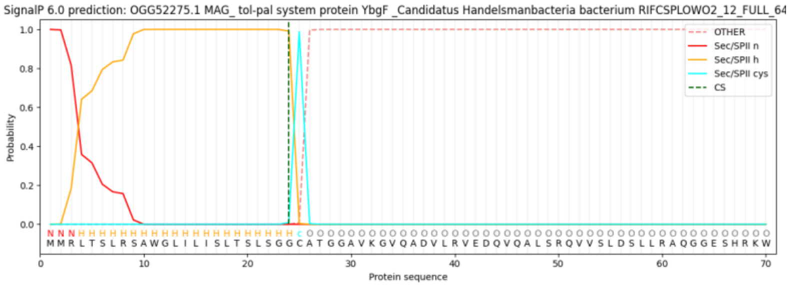

>OGG52277.1 MAG: hypothetical protein A3F84_16210 [Candidatus Handelsmanbacteria bacterium RIFCSPLOWO2_12_FULL_64_10]

MRRSILISLILHLGGMAVLIWGTERGGRVLGPPVRAYGVEIVGPPGRKGGFGWSEPKGDPSKPS
RPTPKTDVVRAKPRTGLKVEAAPSNDPFAPKGTKKPARTPKQKPQEEGSQGTAGDKGTPGGG
GGGLRIEGDLVFPYPGYLDSLVIRIQDHWEEPLAQGTLKATVFFRILRDGTITDFRVDASSGSLPF
DRSALDAVVRANPLSPLPEGFRGDNLGVYFDFEY

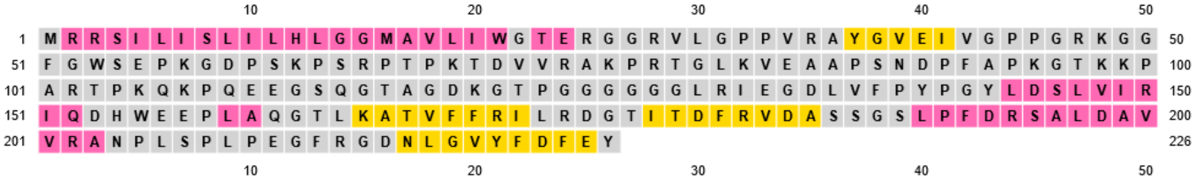

MRRSILISLILHLGGMAVLIW
**GTERGGRVLGPPVRAYGVEIVGPPGRKGGFGWSEPKGDPSKPSRPTPKTDVVRAKPRTGLK
VEAAPSNDPFAPKGTKKPARTPKQKPQEEGSQGTAGDKGTPGGG**
GGGLRIEGDLVFPYPGYLDSLVIRIQDHWEEPLAQGTLKATVFFRILRDGTITDFRVDASSGSLPF
DRSALDAVVRANPLSPLPEGFRGDNLGVYFDFEY

PPIIPRED:

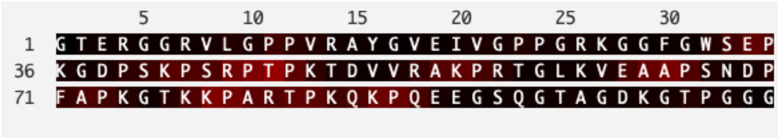

105 Domain II residues

17 Proline residues

2 α-helix residues

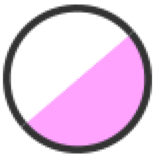

Candidatus Latescibacteria

Candidatus Latescibacteria bacterium 4484_7

Locus: 89950-95535

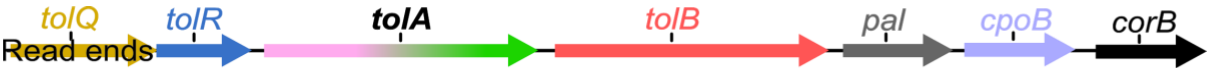

Read ends- -*tolR*-*tolA*-*tolB*-*pal*-*cpoB*-*corB*

Full *tolQ* assumed to be present.

TolB structure confirmed.

CpoB probable lipoprotein.

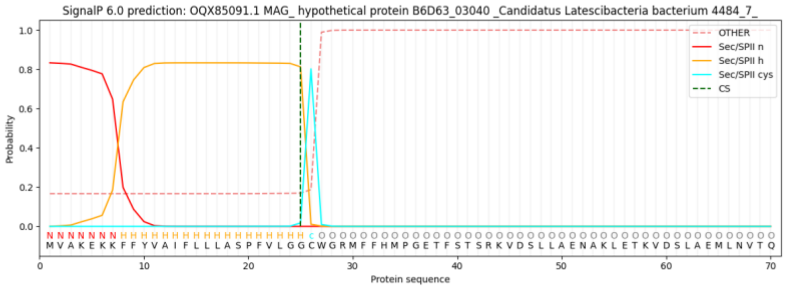

>OQX85093.1 MAG: hypothetical protein B6D63_03055 [Candidatus Latescibacteria bacterium 4484_7]

MKLSLVVSILIHAIVLGTLLFVFKIVPEVRLPQKIYSVRIIRAVTGAKKESPAAEKKAQHKKPRVIRKK
VPARKKKKAPPKKKPEKKPEKAAAEKEKPMDVTVKKEGNTSVAVDAERFPFSYYIEAVQGKVSR
NWFGAVAKGGEGLQCVVYFRLQRDGRVEDVTIEKSSGSTYFDQSALRAVRSSSPFPPLPRAFV
GSYLGIHFTFIQKG

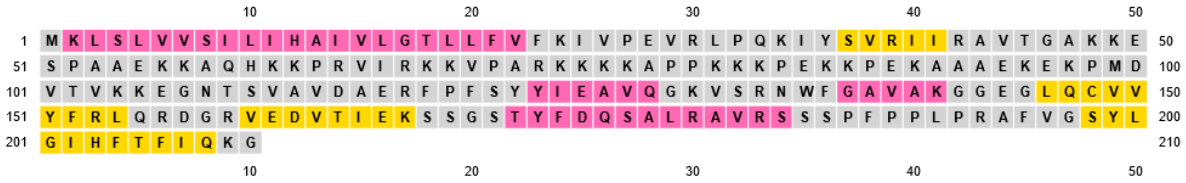

MKLSLVVSILIHAIVLGTLLFV
**FKIVPEVRLPQKIYSVRIIRAVTGAKKESPAAEKKAQHKKPRVIRKKVPARKKKKAPPKKKPEK
KPEKAAAEKEKPMDVTVKKEGNTS**
VAVDAERFPFSYYIEAVQGKVSRNWFGAVAKGGEGLQCVVYFRLQRDGRVEDVTIEKSSGSTY
FDQSALRAVRSSSPFPPLPRAFVGSYLGIHFTFIQKG

PPIIPRED:

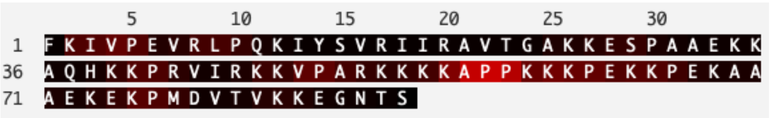

88 Domain II residues

10 Proline residues

0 α-helix residues

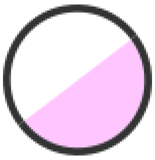

Fibrobacteria

Hallerella succinigenes

Locus: 2481433 -2487539

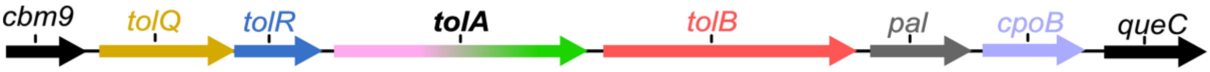

Flanked by a carbohydrate-binding-module 9 gene and *queC*.

CpoB putative Lipoprotein.

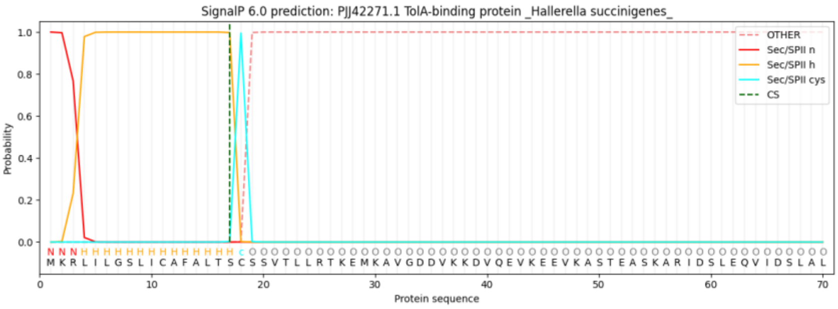

>PJJ42268.1 protein TonB [Hallerella succinigenes]

MAAKKQKENTPFFQSGWNGKLPKIVAISLCFHLMIVIGIVVLNKLNFEREPEPVKIFELVQLQQPQKVAPPKARPKKQPPKPEPPKEVKKEQPKPKPEVKKELPPEPKIAKEEPKPEPEPQPEPEEPQEEPVEETMDLPEDMDLPDEIAEESGLNPVGYVDMDPLMQVYLERLKQIIMQHFNPPSGLNVTKSTKTSVQFTVDRFGNITDISLRRSSGNKTWDNLSVRAIKISKLPELPPNYRAPALSLNFNFTPN

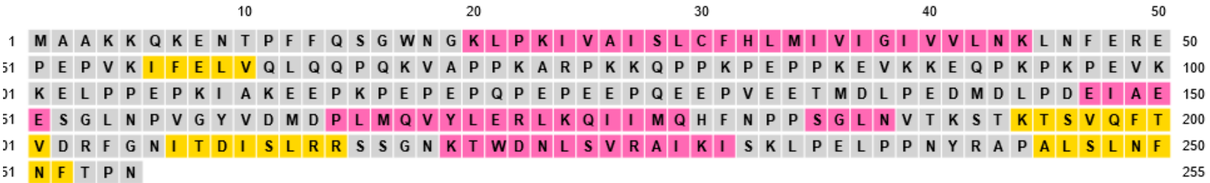

MAAKKQKENTPFFQSGWNGKLPKIVAISLCFHLMIVIGIVVLNK **LNFEREPEPVKIFELVQLQQPQKVAPPKARPKKQPPKPEPPKEVKKEQPKPKPEVKKELPPEPKIAKEEPKPEPEPQPEPEEPQEEPVEETMDLPEDMDLPDEIAEESGLN** PVGYVDMDPLMQVYLERLKQIIMQHFNPPSGLNVTKSTKTSVQFTVDRFGNITDISLRRSSGNKTWDNLSVRAIKISKLPELPPNYRAPALSLNFNFTPN

PPIIPRED:

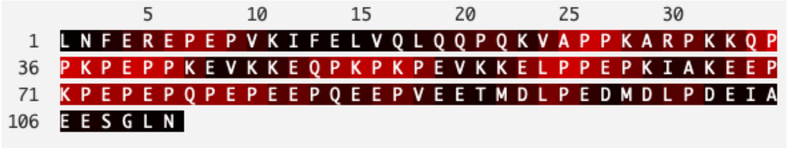

111 Domain II residues

27 Proline residues

5 α-helix residues

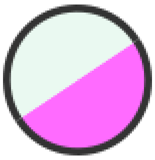

Candidatus Marinimicrobia

Marinimicrobia bacterium

Locus: 71258 – 79074

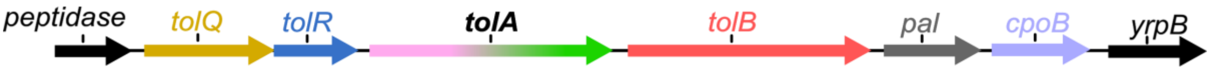

shows *S9-peptidase-tolQ-tolR-tolA-tolB-pal-cpoB-yrpB*

CpoB putative lipoprotein.

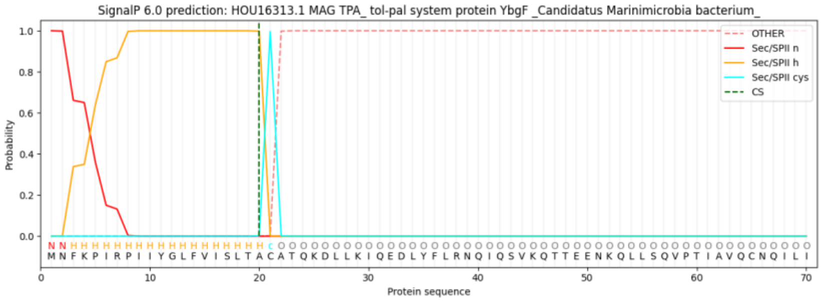

>HOU16316.1 MAG TPA: TonB family protein [Candidatus Marinimicrobia bacterium]

MNYTFAISLILHLTFFAVVSVLAGRHQPRVMAIPDQNIRWVNIAPPVVQTAEAPIQVKPATSVPVP
KPKVEKAATVLAEKPQKPQETKPLPEETIASSTTSPELPVEPEATSQPASGGIPGATGLRVDDPD
FNFIYYLNIIRNRIQEHWRPPYSSADSPYSQQVMIAFKITRSGKISDVRIEQSSGNFLFDQAALRAL
YETATLPPLPSEYGGKELNVHIEFETLK

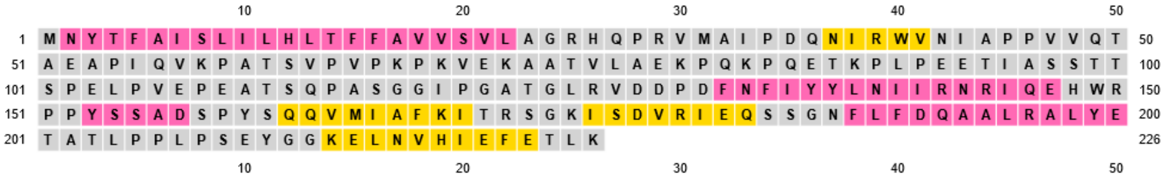

MNYTFAISLILHLTFFAVVSVL
AGRHQPRVMAIPDQNIRWVNIAPPVVQTAEAPIQVKPATSVPVPKPKVEKAATVLAEKPQKPQ
ETKPLPEETIASSTTSPELPVEPEATSQPASGGIPGATGLR
VDDPDFNFIYYLNIIRNRIQEHWRPPYSSADSPYSQQVMIAFKITRSGKISDVRIEQSSGNFLFDQ
AALRALYETATLPPLPSEYGGKELNVHIEFETLK

PPIIPRED:

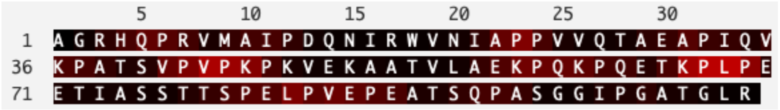

104 Domain II residues

18 Proline residues

0 α-helix residues

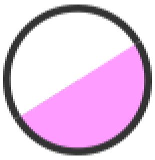

Candidatus Delongbacteria

Candidatus Delongbacteria bacterium

Has Omp85-TolB protein (prediction)

Strange OmpA-PGB-TSP fusion also predicted.

Gene tested by SignalP 6: secreted, unlikely lipidated. Could be lipidated but in species outside of canonical lipidation signal algorithm detection.

Potentially a Pal? (Prediction)- no secretion predicted.

Assumed no *tol-pal* loci, no canonical *tolB* or *pal* observed.

Candidatus Delongbacteria bacterium GWF2_40_14

Candidate division KSB1

candidate division KSB1 bacterium

Locus: 8051 -17142

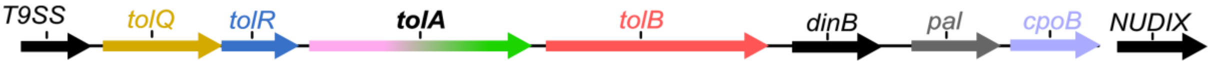

Flanked by T9SS genes and a hydrolase gene. Interloping *dinB* gene.

CpoB putative lipoprotein.

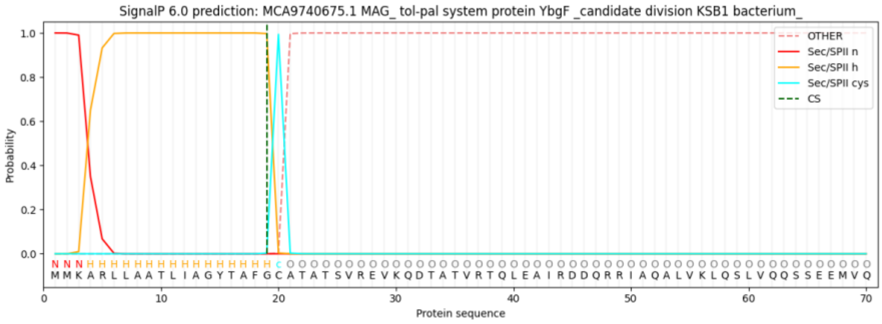

>MCA9740679.1 MAG: TonB family protein [candidate division KSB1 bacterium]

MQRSLLFSIVGHILVFGLLLILSWAFASRETRSYPKTITATFVAKSPSNSTARSAPRKVEPPKPEPT
IKPAVKAKVNRQAVKKEPERRKPQPAAANKSADKNTPGLSSPGMKIDAPEFPFPEYLALIQYRIE
SQWQPPTNAGGGLLTTVYFRIGQQGDLSEIKVTKASGSFVVDQSALRAVYAANPLPPLPAGSGL
SSLGVHFDFVVY

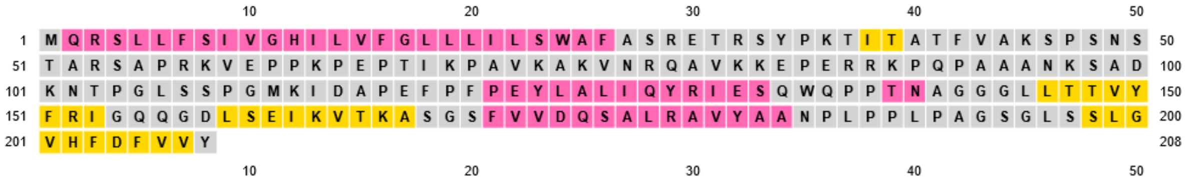

MQRSLLFSIVGHILVFGLLLILSWAF
**ASRETRSYPKTITATFVAKSPSNSTARSAPRKVEPPKPEPTIKPAVKAKVNRQAVKKEPERRK
PQPAAANKSADKNTPGLSS**
PGMKIDAPEFPFPEYLALIQYRIESQWQPPTNAGGGLLTTVYFRIGQQGDLSEIKVTKASGSFVV
DQSALRAVYAANPLPPLPAGSGLSSLGVHFDFVVY

PPIIPRED:

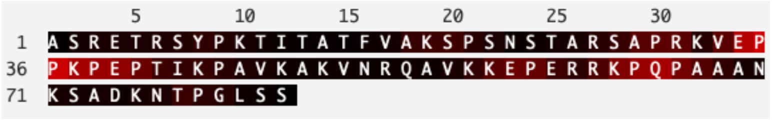

82 Domain II residues

12 Proline residues

0 α-helix residues

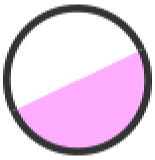

candidate division KSB3 bacterium

Locus: 12441- 17538

tolQ-*tolR*-*tolA*-*tolB*-*pal*-*cpoB*

Notably, features *cpoB* annotated to feature N-terminal *envC* domain.

TolA also proline rich

>PID60197.1 MAG: hypothetical protein CSB45_00635 [candidate division KSB3 bacterium]

MSEGNYEEQRGFRWILLGSVIFHFVLLAGLLGIFYQPSEVAVFPTETEDAQFVDLLDSSELPEELSLPTLPSERDTSPR
DEQLAFRSISSLAVVPSPTATATISPSPLPTATPAALPTLTPTFKPLPSPTPIRPRIPVPKFNWTPKPTPTRRETVKPAIRL
DPYQVPRRHGVLDPTPVMSRESQKDWGGGPNVRQQHFLGSRSSLLLDQENDFPFPGYLQHLEKKIAGLWFPQG
AGTVTIFLEVAQNGKILKSEVDKGTEVGVEKLHESVVRALSLIKHFEALPWEYRGRTLRVRIIVRR

Calditrichaeota

Caldithrix sp

[Locus](): 12095 - 17778

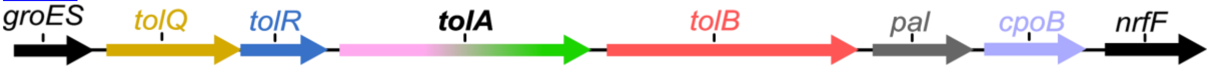

Flanked by *groES* and *nrfF*.

CpoB putative lipoprotein.

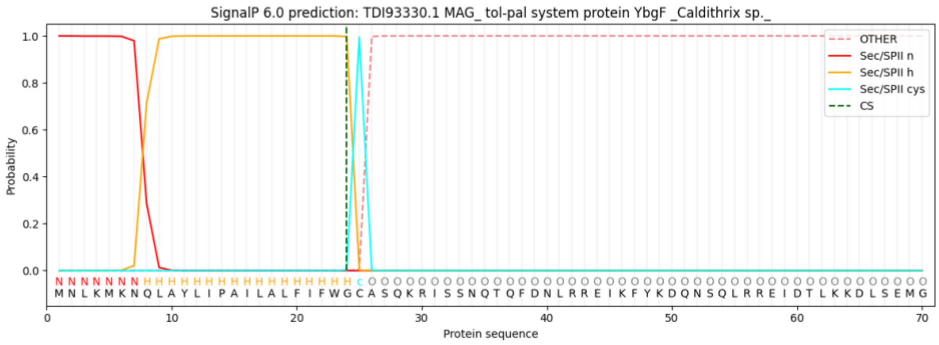

>TDI93327.1 MAG: TonB C-terminal domain-containing protein [Caldithrix sp.]

MKSNILFSFTFHIIIFAALFLYRSEPRKFEGYPIVIPVELVQIEPVSFKAPEVEKLKPKRRKVKPKPK
KLEGVTVEKKKVEQEPEEQPPKQKETPEKSNEGKSTIGGEKVRLDVKDFPFSYYLSLLQSRIQA
NWEPPFSSSRRSLFKKVIIYFKIRRNGKLTNLALESKSGDPRFDQAALRAVTLASPLPPLPFDFPE
PSLGVHFEFAQGN

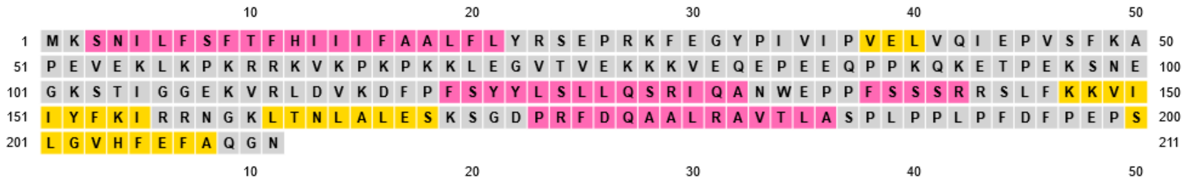

MKSNILFSFTFHIIIFAALFL
YRSEPRKFEGYPIVIPVELVQIEPVSFKAPEVEKLKPKRRKVKPKPKKLEGVTVEKKKVEQEPE
EQPPKQKETPEKSNEGKSTIGGEKVR
LDVKDFPFSYYLSLLQSRIQANWEPPFSSSRRSLFKKVIIYFKIRRNGKLTNLALESKSGDPRFDQ
AALRAVTLASPLPPLPFDFPEPSLGVHFEFAQGN

PPIIPRED:

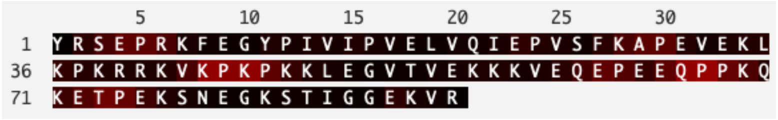

90 Domain II residues

12 Proline residues

0 α-helix residues

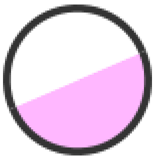

Candidatus Kryptonia

No *pal* according to Witwinowski et al.

Candidatus Chrysopegis kryptomonas

Omp-85-TolB fusion protein. TolB-like protein (prediction), but not a Rossman-fold in NTD.

Candidatus Kryptobacter tengchongensis

TolB-like protein (prediction) lacking Rossman-fold NTD.

Candidatus Kryptonium thompsoni

TolB-like protein (prediction) lacking Rossman-fold NTD.

Candidatus Thermokryptus mobilis

TolB-like protein (prediction) lacking Rossman-fold NTD.

All species featured a TolB homologue, where the NTD had a double β-sheet fold resembling a β-glucanate laminarinase domain rather than the traditional Rossman-fold.

Ignavibacteria

No *pal* according to Witwinowski et al.

Locus: 30589

Was found to have a PorE-like protein (TolB-CPE-OmpA) - no lipoprotein signal predicted

Ignavibacterium album JCM 16511

Omp85-TolB fusion protein.

Candidatus Kapaibacteria

No *pal* according to Witwinowski et al.

Candidatus Kapaibacterium sp.,

Was found to have an PorE-like protein (TolB-CPE-OmpA)- no lipoprotein signal predicted.

Bacteroidetes

No *pal* according to Witwinowski *et al.* However, PorE detected.

Porphyromonas gingivalis

Genome: 1124174-1126192

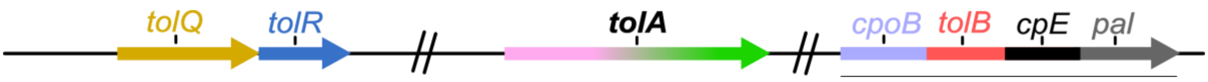

porE *"tolA"*-based on AF3 co-prediction below

Flanked by *traJ* and *queE*.

cpoB-tolB-carboxypeptidase-pal fusion protein structure

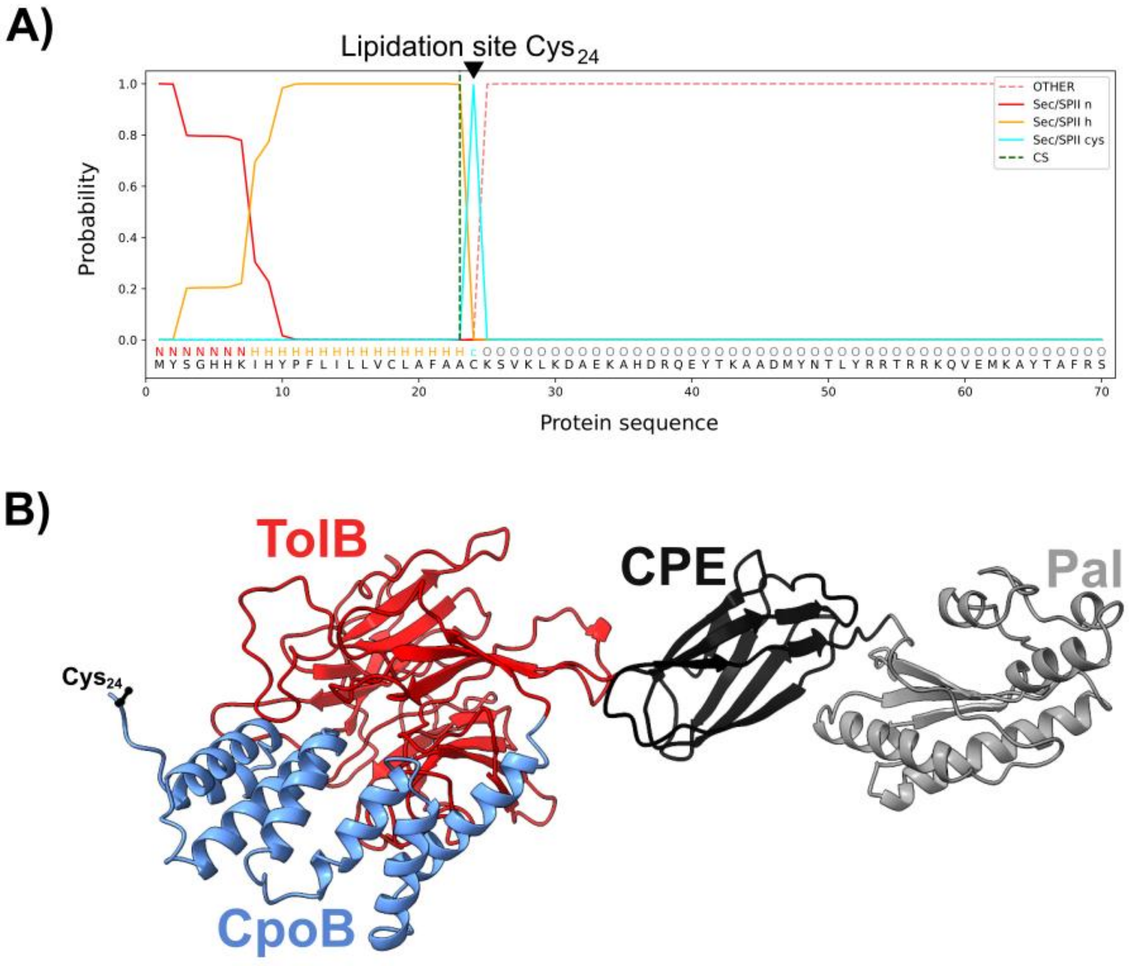

PorE is a putative multidomain OM-lipoprotein comprising CpoB, TolB, Carboxypeptidase and Pal -like domains. A) SignalP 6.0 suggests N-terminal lipidation at Cys_{24}. **B)** AF3 model of PorE. PorE prediction models are available on UniProt for *P. gingivalis* and its homologues. PorE homologues were found in Cytophagia, Flavobacteria.

Rhodothermaeota

Rhodothermaeota bacterium MED-G19

No *pal* according to Witwinowski et al.

Locus: 48978

Flanked by phosphoribosylformylglycinamidine cyclo-ligase gene and an ABC-transporter ATPase

Found a PorE protein. Also found an Omp85-TolB fusion.

Found to be lipidated at Cys_{18}:

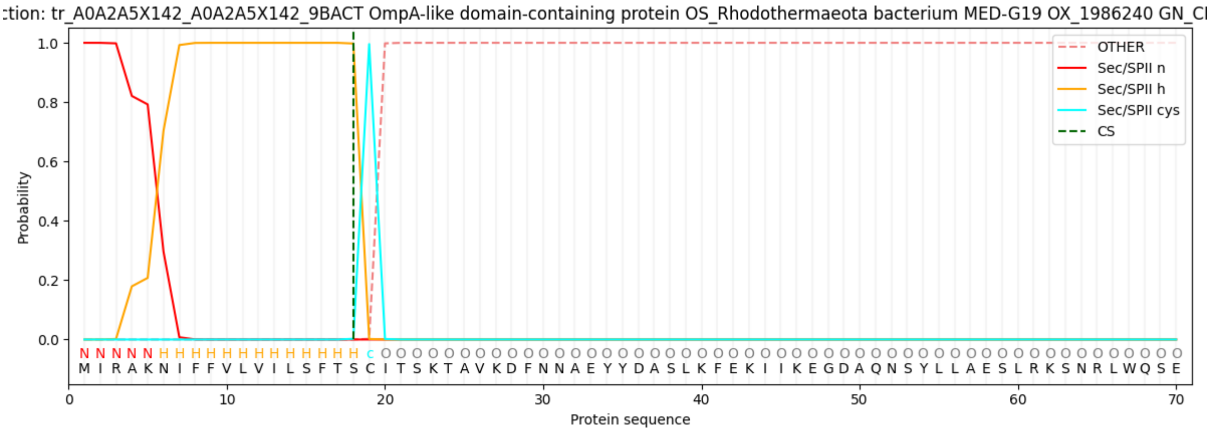

Balneolaeota

No *pal* according to Witwinowski et al.

Balneolaceae bacterium

Omp85-TolB-fusion predicted.

Also features a TolB-like protein with a β-glucanate laminarinase domain

Also appears to have a Pal-like protein with a resident protein. Secreted but not predicted to be lipidated.

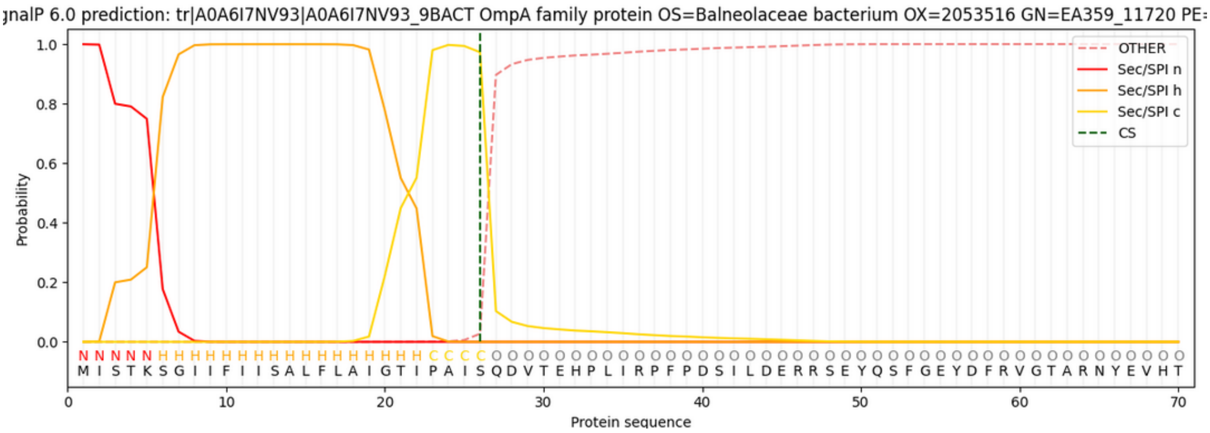

Candidatus Cyclonatronum proteinivorum

Omp85-TolB fusion

A Pal-like protein observed.

Secreted but not lipidated:

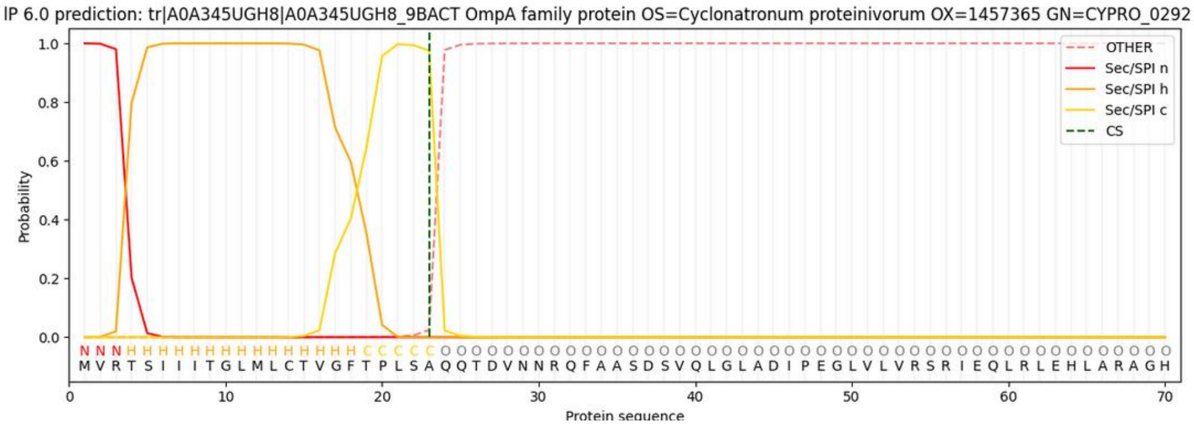

Chlorobi

Chlorobium tepidum TLS

Locus: 632305 – 639750

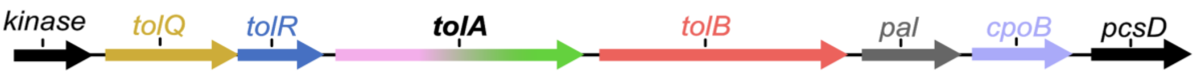

Flanked by Dolichol kinase gene and *pcsD* encoding a P840 reaction centre protein.

CpoB is a putative lipoprotein.

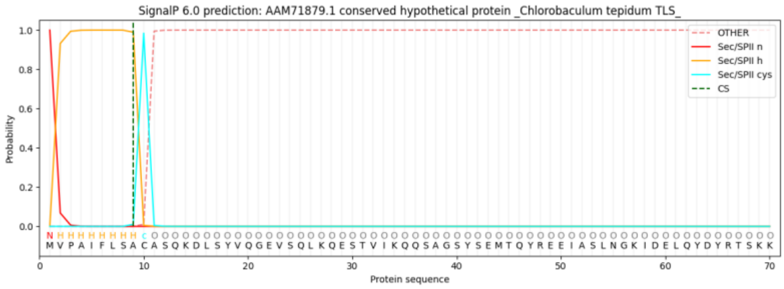

>AAM71874.1 hypothetical protein CT0635 [Chlorobaculum tepidum TLS]

MQRRVELKLEQRRFYVWLGIAAAAHVAVVAAVVVLQLLYVRMHPPMKIVNVSLVQMPGLPGPA
GGPKSPETPPAAIEKQAETPELASAKKVAEPPPQPVKKIAKPVAAVKKIPEKPPVKAPVKAPEPA
SKTQSAADERKKIAEPVAAVKKIPEKPPLKAPVKAPEPAPKTQSAADERKNLQEALERLKSKSAS
QKAETGKSAAPSNLSSTLANLQKKVASGGGGPARSGSGSGAGGGRYGTG
GGGAFDSYKARIADIIQNNWSFSSQMVRSTSGMEVYVSLLILPDGSVNEIRYDRKSSSEYLNNSV
KNALAKSMPFPSLPREYGAKGIWVGFVFTPEGVGR

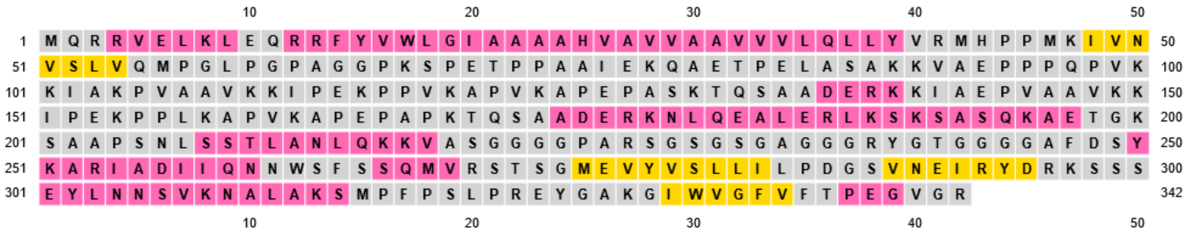

MQRRVELKLEQRRFYVWLGIAAAAHVAVVAAVVVLQLLYV
**RMHPPMKIVNVSLVQMPGLPGPAGGPKSPETPPAAIEKQAETPELASAKKVAEPPPQPVKKIA
KPVAAVKKIPEKPPVKAPVKAPEPASKTQSAADERKKIAEPVAAVKKIPEKPPLKAPVKAPEP
APKTQSAADERKNLQEALERLKSKSASQKAETGKSAAPSNLSSTLANLQKKVASGGGGPAR
SGSGSGAGGGRYGTG**
GGGAFDSYKARIADIIQNNWSFSSQMVRSTSGMEVYVSLLILPDGSVNEIRYDRKSSSEYLNNSV
KNALAKSMPFPSLPREYGAKGIWVGFVFTPEGVGR

PPIIPRED:

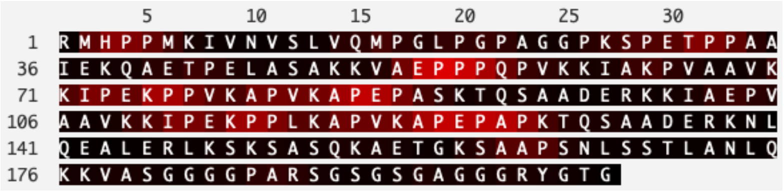

203 Domain II residues

31 Proline residues

39 α-helix residues

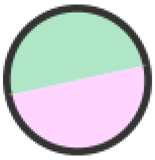

Terrabacteria

Thermotogae

Thermotoga maritima

Features TolB, despite being Terrabacterial. Likely acquired via horizontal gene transfer.

Analysis of nearby genes detected no other Tol-Pal genes, nor was *pal* detected in this phylum by Witwinowski et al.

WebFlags output:

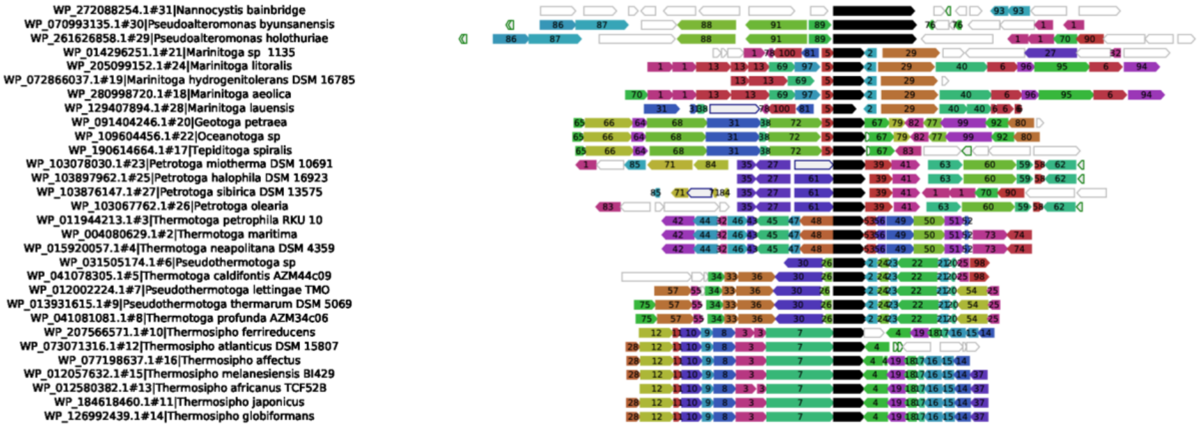

1: ABC transporter ATPase

2: CheW

3: Metallophosphoesterase

4: GatB/YqeY
