# Supplementary material for: Origin and Evolution of Bacterial Periplasmic Force Transducers
Source: Mol Biol Evol. 2025 Jun 4;42(6):msaf138. doi: 10.1093/molbev/msaf138 (PMC12204202; doi:10.1093/molbev/msaf138)
Supplement: msaf138_Supplementary_Data [file msaf138_supplementary_data.zip › REVISED- SI 2 Ton system catalogue.pdf]

Introduction:

The following index is laid out to display examples of a *tonB* gene and a TBDT gene from each proteobacterial species to demonstrate presence of functional Ton systems. Each gene was taken from the same genome accession as the *tol-pal* loci in SI. 1, to ensure that they are not *tolA* genes, and in no case are the neighbouring *tol* genes (*tolB, pal cpoB*) present. In cases where there were multiple TonB paralogues, the first *tonB* with the expected 3-domain secondary structure was analysed. Each identified TonB sequence is displayed in FASTA format, followed by its PsiPred structural prediction, followed by the domain II analysis, where domain II is outlined in bold and separated by spaces.

Every TonB in this list was also visually inspected in the embedded UniProt Alphafold prediction model to check for the presence of an N-terminal helix, domain II, and globular domain III. In cases of poor annotation, pBLAST searches were performed using *tonB* from the closest branches on the phylogenetic tree.

Each PsiPred prediction uses the following legend:

Strand Helix Coil

Each PPIIPRED predictions uses the following legend:

0 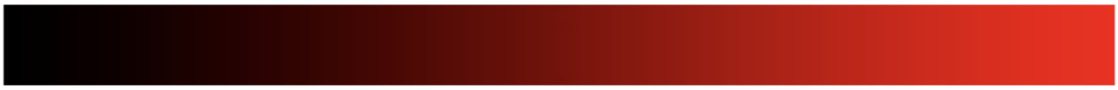 1

Where 0 indicates no propensity for PPII helix formation, and 1 indicates maximum propensity for PPII helix formation.

Helix: Proline analysis was performed by first defining domain II (indicated in bold for each protein), specifically after the predicted TMH, up to 100 residues before the C-terminus.

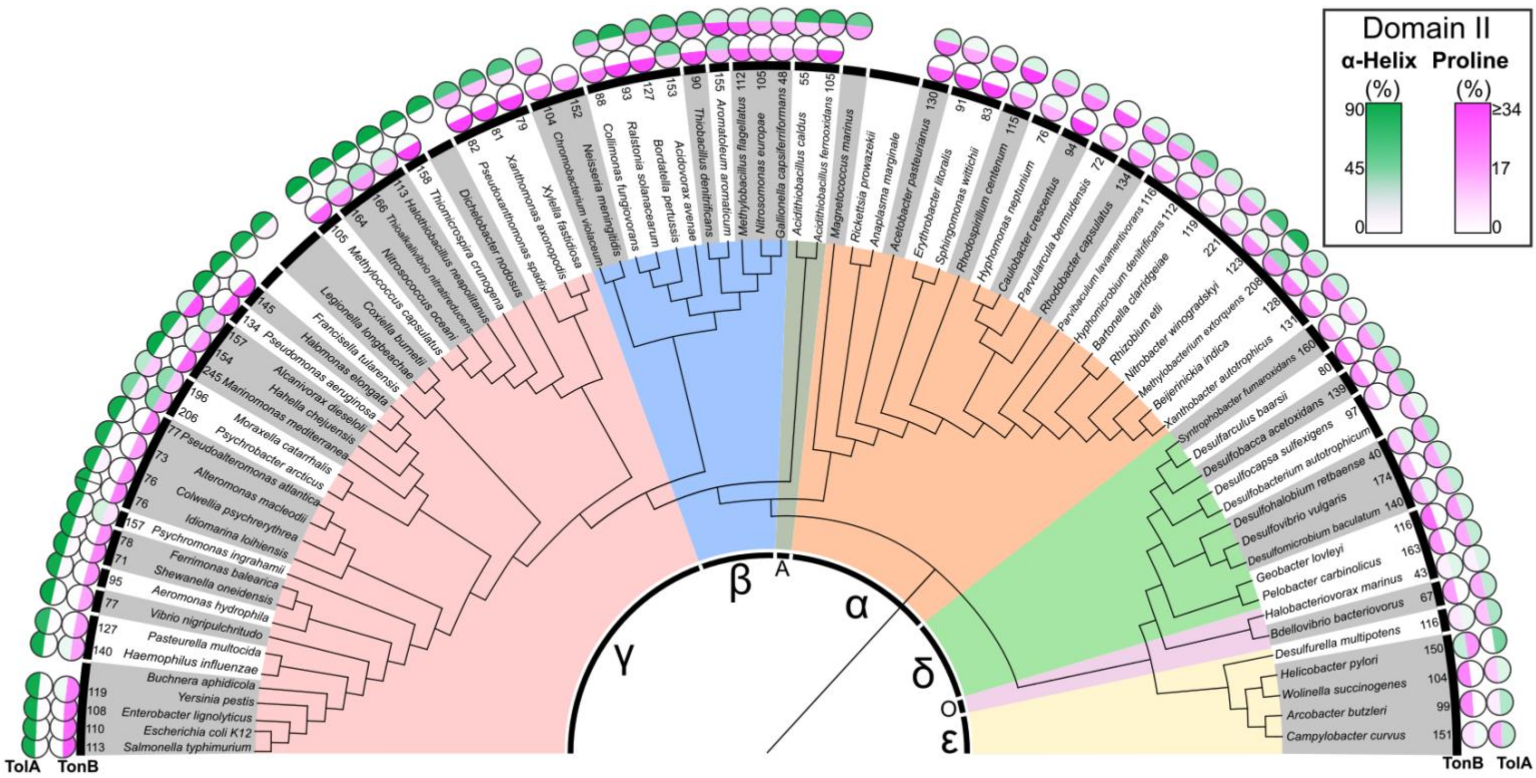

Figure S2.1 | TolA and TonB domain II length and secondary structure compositions vary amongst Proteobacteria. Genome accessions were screened for both *tonB* and TBDT genes (SS 1). Each representative TonB was structurally analysed by the same method as for TolA (SI 1). Proline content of some TonBII sequences exceeded 34%, but they are coloured the same for consistency. Phylogenetic tree adapted from Sharma *et al*., 2023. Circumferential numbers indicate respective length of TonBII per sequence analysed.

Contents

Introduction: **1**

Proteobacteria (Sharma tree) **5**

γ-Proteobacteria **5**

Enterobacterales 5

Pasteuralleles 10

Vibrionales 12

Aeromondales 13

Alteromondales 14

Iteromondales 20

Pseudomonodales 21

Oceanspirallales 24

Thiotrichales 29

Legionellales 32

Methylococcales 33

Chromatiales 35

Cardiobacteriales 38

Xanthomonadales 39

β-Proteobacteria **42**

Neisseriales 42

Burkholderiales 44

Rhodocyclales 48

Nitrosomonadales 49

Acidithiobacillales **53**

α-Proteobacteria **55**

Magnetococcales 55

Rickettsiales 56

Rhodospirillales 57

Sphingomonadales 59

Rhodobacterales 61

Caulobacterales 63

Parvularculales 64

Rhizobiales 66

δ-Proteobacteria **73**

Syntrophobacterales 73

Desulfarculales 75
Desulfobacterales..76
Desulfovibrionales 78
Bacteriovoracles 81
Desulfurellales 83
Oligoflexia 84
Bacteriovoracles (cont.)...84
Bdellovibrionales 85
ε-Proteobacteria 86
Campylobacterales...86

Proteobacteria (Sharma tree)

γ-Proteobacteria

Enterobacterales

Salmonella typhimurium

>AAA27031.1 vitamin B12 transport protein [Salmonella enterica subsp. enterica serovar Typhimurium]

MIKKATLLTAFSVTAFSAWAQDTSPDTLVVTANRFQQPRSAVLAPVTIVTRQDIERWQSTSV
NDVLRRLPGVDIAQSGGARQNSSIFIRGTNSSHVLVLIDGVRLNLAGVSGSADLSQFPVSLV
QRIEYIRGPPSAIYGSDAIGGVVNIITTRDNPGTELTAGWGSNSYQNYDISTQQQLGEITRAT
LIGDYEYTKGFDVVAKGGTGMQAQPDRDGFLSKTLYGALEHTFSDRWSGFVRGYGYDNR
TDYDAYYSPGSPLIDTRKLYSQSWDAGLHFNGESIQSQLVSSYSHSKDYNYDPHYGRYDT
SATLDEMKQYNVQWTNSVVVGHGNVGAGVDWQKQTTTPGTGYVPEGYDQRNTGVYLTG
LQQLGDFTLEAAARSDDNSQFGRHGTWQTSAGWEFIEGYRFIASYGTSYKAPNLGQLYGY
YGNPNLNPEKSKQWEGAFEGLTAGVSWRISGYRNDINDMIDYDDHLQKYYNEGKARIKGIE
ATANFDTGPLTHTVSYDYVDARNAITDTPLPRRSKQMAKYQLDWDVYDFDWGMTYQYLGS
RYDSDYSAYPYRTVKMGGVSLWDLTVAYPVTSHLTVRGKIANLFDKDYETVYGYQTAGRE
YTLSGSYTF

>CAA39818.1 tonB protein [Salmonella enterica subsp. enterica serovar Typhimurium]

MTLDLPRRFPWPTLLSVGIHGAVVAGLLYTSVHQVIELPAPAQPITVTMVSPADLEPPQAVQ
PPPEPVVEPEPEPEPEPIPEPPKEAPVVIEKPKPKPKPKPKPVKKVEEQPKREVKPAAPR
PASPFENSAPVRPTSSTASATSKPAVSVPTGPRALSRNQPQYPARAQALRIEGRVKVKFDV
TSAGRVENVQILSAQPANMFEREVKNAMRKWRYEAGKPGSGLVVNIIFRLNGTAQIE

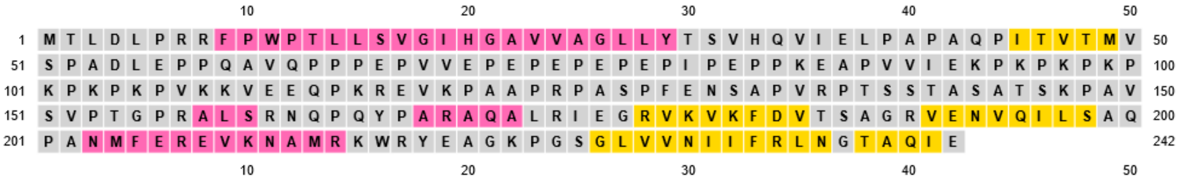

MTLDLPRRFPWPTLLSVGIHGAVVAGLLY
TSVHQVIELPAPAQPITVTMVSPADLEPPQAVQPPPEPVVEPEPEPEPEPIPEPPKEAPVVI
EKPKPKPKPKPKPVKKVEEQPKREVKPAAPRPASPFENSAPVRPTSSTA
SATSKPAVSVPTGPRALSRNQPQYPARAQALRIEGRVKVKFDVTSAGRVENVQILSAQPAN
MFEREVKNAMRKWRYEAGKPGSGLVVNIIFRLNGTAQIE

PPIIPRED:

113 Domain II residues

33 Proline residues

0 α-Helix residues

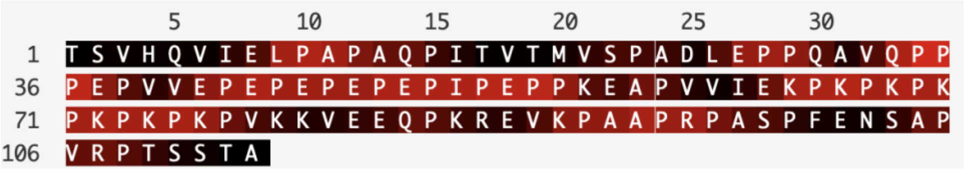

Escherichia coli K12

>AAA23524.1 B12 receptor protein BtuB [Escherichia coli]

MIKKASLLTACSVTAFSAWAQDTSPDTLVVTANRFEQPRSTVLAPTTVVTRQDIDRWQSTS
VNDVLRRLPGVDITQNGGSGQLSSIFIRGTNASHVLVLIDGVRLNLAGVSGSADLSQFPIALV
QRVEYIRGPRSAVYGSDAIGGVVNIITTRDEPGTEISGGWGSNSYQNYDVSTQQQLGDKTR
VTLLGDYAHTHGYDVVAYGNTGTQAQTDNDGFLSKTLYGALEHNFTDAWSGFVRGYGYD
NRTNYDAYYSPGSPLLDTRKLYSQSWDAGLRYNGELIKSQLITSYSHSKDYNYDPHYGRYD
SSATLDEMKQYTVQWANNVIVGHGSIGAGVDWQKQTTTPGTGYVEDGYDQRNTGIYLTGL
QQVGDFTFEGARRSDDNSQFGRHGTWQTSAGWEFIEGYRFIASYGTSYKAPNLGQLYGF
YGNPNLDPEKSKQWEGAFEGLTAGVNWRISGYRNDVSDLIDYDDHTLKYYNEGKARIKGV
EATANFDTGPLTHTVSYDYVDARNAITDTPLLRRAKQQVKYQLDWQLYDFDWGITYQYLGT
RYDKDYSSYPYQTVKMGGVSLWDLAVAYPVTSHLTVRGKIANLFDKDYETVYGYQTAGRE
YTLSGSYTF

>NP_415768.1 Ton complex subunit TonB [Escherichia coli str. K-12 substr. MG1655]

MTLDLPRRFPWPTLLSVCIHGAVVAGLLYTSVHQVIELPAPAQPISVTMVTPADLEPPQAVQ
PPPEPVVEPEPEPEPIPEPPKEAPVVIEKPKPKPKPKPVKKVQEQPKRDVKPVESRPASP
FENTAPARLTSSTATAATSKPVTSVASGPRALSRNQPQYPARAQALRIEGQVKVKFDVTPD
GRVDNVQILSAKPANMFEREVKNAMRRWRYEPGKPGSGIVVNILFKINGTTEIQ

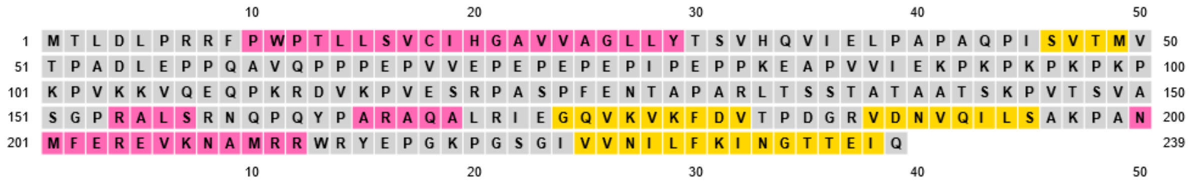

MTLDLPRRFPWPTLLSVCIHGAVVAGLLY
**TSVHQVIELPAPAQPISVTMVTPADLEPPQAVQPPPEPVVEPEPEPEPIPEPPKEAPVVIEK
PKPKPKPKPKPVKKVQEQPKRDVKPVESRPASPFENTAPARLTSSTAT**
AATSKPVTSVASGPRALSRNQPQYPARAQALRIEGQVKVKFDVTPDGRVDNVQILSAKPAN
MFEREVKNAMRRWRYEPGKPGSGIVVNILFKINGTTEIQ

PPIIPRED:

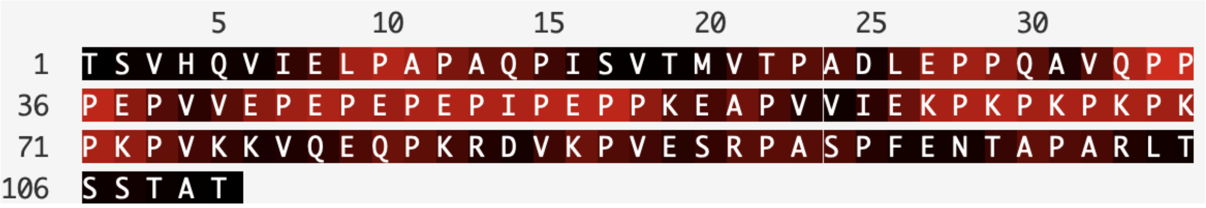

110 Domain II residues

29 Proline residues

0 α-Helical residues

Enterobacter lignolyticus

>ALR74858.1 vitamin B12/cobalamin outer membrane transporter [[Enterobacter] lignolyticus]

MIKKASLLTALSVTAFSGWAQDSGSDTMVVTANRVEQPINTVLAPVSVVTRQDIDRWQSTSVVDVMRRLPGVDV
AQYGGLGQQSSLFIRGTNSSHVLILVNGIRLNQAGVSGSSDLSQFPISLVQRIEYIRGPRSAVYGSDAIGGVVNIITT
RDRDGTTLNAGIGTYGYQSYGGSTQQTLGGNTRVTVAGDYTYTKGFDVVAEGNTGGVAQPDRDGFMSKTLYG
SIEHDFSDQWGAFVRGYGYDNRTAYDGSVFTPGVLVDTRQLYSQTWDAGLRYNNDIFHSQLTSSYSHSKDYNY
DPNLGRYDSTATLDDVKQYNLQWANSVEVGHGNIGAGVDWQKQSTEPGTSYVTDAYDIRNTGLYMTALQKLGD
FTLEGAARSDDNSQFGQHGTWQSSAAWEFIDGYRFVASYGTAFKAPNLGQLYGFYGNENLKPEESKQWEGAF
EGLTAGVTWRVSGYRNDIDNLIDYNTDLQKYYNVGQARIKGIEATASFDTGPLTHNIGYDYVDARDAKTNELLDRR
AKQQVKYQLDWQVYDFDWSMTYHYLGTRYDTDYSTYPSQKVKMGGVSLWDVAVSYPITSHLTVRGKIANLFDK
DYETVYGYHTAGREYNLSGSYTF

>ALR76948.1 energy transducer TonB [[Enterobacter] lignolyticus]

MTLDLPRRFPWPTLLSVGIHGAVVAGLLYTSVHQVIELPAPAQPIMVSMVAPADLEPPQAVQ
PTPQPVAEPEPEPEVVPEPPKEAPVVIHKPKPKPKPKPKPEKKVEQPEREVKPVEPRATSS
FENATTPSRPAMNSAPATSAPVAAAPSGPRALSRNQPQYPQRAQALRIEGNVRVKFDVTP
EGRVENVEILSAQPSNLFERDVKAAMRKWRYEPGKPGTGLVMNIEFRLKGVQMN

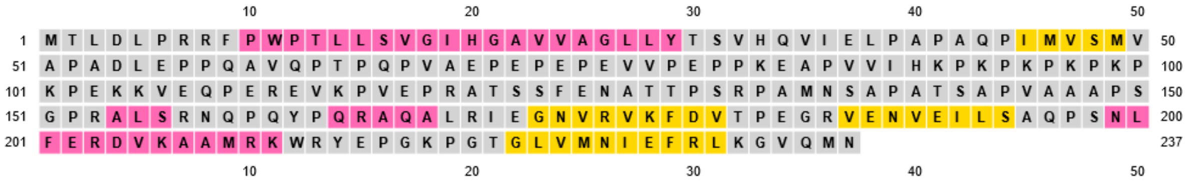

MTLDLPRRFPWPTLLSVGIHGAVVAGLLY
**TSVHQVIELPAPAQPIMVSMVAPADLEPPQAVQPTPQPVAEPEPEPEVVPEPPKEAPVVIH
KPKPKPKPKPKPEKKVEQPEREVKPVEPRATSSFENATTPSRPAMNS**
APATSAPVAAAPSGPRALSRNQPQYPQRAQALRIEGNVRVKFDVTPEGRVENVEILSAQPS
NLFERDVKAAMRKWRYEPGKPGTGLVMNIEFRLKGVQMN

PPIIPRED:

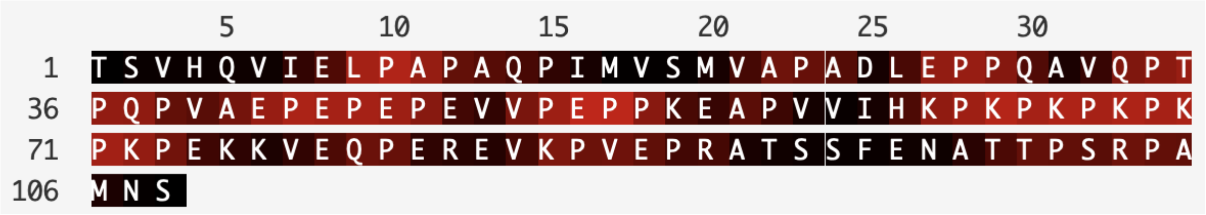

108 Domain II residues

27 Proline residues

0 α-Helix residues

Yersinia pestis

>ABG12081.1 putative vitamin B12 receptor protein [Yersinia pestis Antiqua]

MIIKNTMTIKKYTLLTALSVTAFSGWAQGNNTTDNNDEMVVTANRFPQPKSSVLAPVDVVTRADIDRWQSTNIND
VLRRLPGVDIAQDGGMGQRSSLFIRGTNSSHVLVLIDGVRLNQAGITGASDLSQIPISLVQRIEYIRGPRSAVYGSD
AIGGVINILTGRDKPGTTLSAGLGSNGYQTYDGSTQQKLGEDTTVTLAGNYTYSKGYDVVAGMPGAGGPRQPDR
DGFMGKMLWAGLEHQFNEQFNGFARVYGFDNRSDYDGYTNYSNPLALIDTRKLSSRTYDTGLRYKNGIYASQFI
ASYNRTKDYNYSPLFGQHDITASLDEAEQYNLQWGNTFQLTNGMISAGADWQEQRTERKSSNQNTTADFTQHN
TGIYLTGQQQISDVTLEGAVRSDDNSQFGWHSTWQTSAGWEFIDGYRLIGSYGTAYKAPNLMQLYSAYGGNANL
KPEESKQWEGGVEGLTGPLTWRLSAYRNDIDQLIDYSNLTNGYFNINKATIKGVEWTGSFDTGPLSHQVTLEYLD
PRNADTHEILVRRAKQQVKYQLDWQVADLDWSVTYQYLGQRYDKDYSTYPEETVELGGVSLWDLAVSYPVTSH
LTVRGRIANLFDKDYEMVYGYQTPGREYYFTGSYNF

>ABG13518.1 TonB protein [Yersinia pestis Antiqua]

MQLIKFFSDRRLTWSLIFSIGLHGSVVAALLYVSVEQMKIQPEIEDTPLAVTMVNIAEFAAPQP
AAAAPEPVQETPAVPEETPPVLEETPPEPEELPEPVPVPVPEPVKPKPKPVKKEVKKPEVK
KTQAPPDDKPFKSDEAALVANNAPVKSAPVASTPGLSTSAGPKALSKAKPSYPARALALGIE
GQVKVQYDIDESGRVTNVRVLEATPRNTFEREVKQVMRKWRFEAVAAKNYVTTIVFKLDGK
MEMN

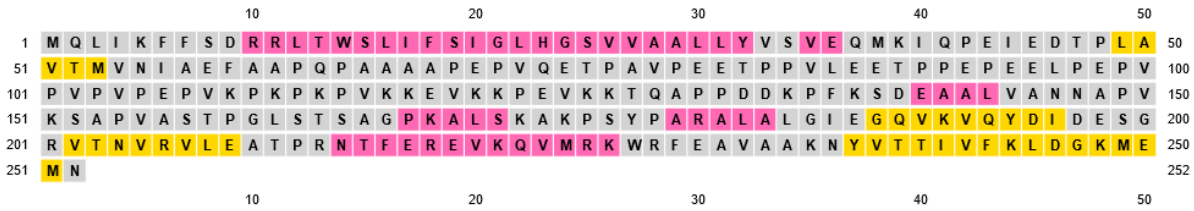

MQLIKFFSDRRLTWSLIFSIGLHGSVVAALLY
**VSVEQMKIQPEIEDTPLAVTMVNIAEFAAPQPAAAAPEPVQETPAVPEETPPVLEETPPEP
EELPEPVPVPVPEPVKPKPKPVKKEVKKPEVKKTQAPPDDKPFKSDEAALVANNAPVK**
SAPVASTPGLSTSAGPKALSKAKPSYPARALALGIEGQVKVQYDIDESGRVTNVRVLEATPR
NTFEREVKQVMRKWRFEAVAAKNYVTTIVFKLDGKMEMN

PPIIPRED:

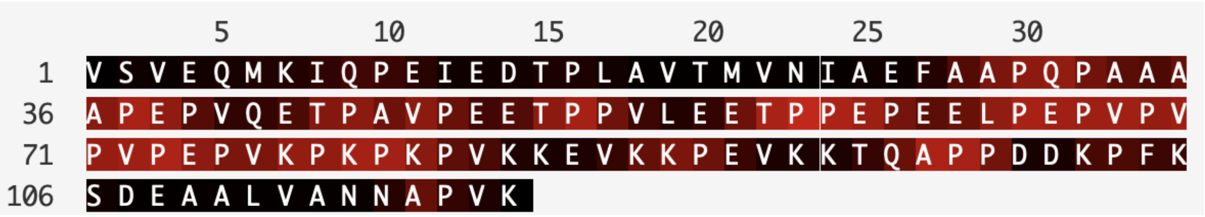

119 Domain II residues

27 Proline residues

6 α-Helix residues

Buchnera aphidicola

No *tonB* or TBDT genes detected.

Pasteuralleles

Haemophilus influenzae

>AAC21789.1 hemin receptor (hemR) [Haemophilus influenzae Rd KW20]

MNILINKRIFLLVTLVGIQLNVTAKQNSSNSNREELLPIIVNTNDDSNKLPGRSVLKQKNIEQXQADNAANLINILPGV
NMAGGFRPGGQTLNINGMGDAEDVRVQLDGATKSFEKYQQGSIFIEPELLRRVTVDKGNYSPQYGNGGFAGTV
KFETKDARDFLQENQKIGGFLKYGNNSNNNQKTYSTALVLQNEQKNIDLLLFGSVRNAGDYKRPDNSKILFSKNN
QKTGLIKLNWQISPEHLLTLSSVYGIHKGWEPFAAKRDILPKPSLSDIMRYGTDIAWKRKLVYRDQKDENYTLKYN
YLPENNPWINLSTQFSYSKTTQNDMRPKEASSGLVGSLGNQSWITYSDLTFDINNTSTFNIKTTVHELLFGLQWLK
NTRNTLMYDKSKVRKADYNYGYFQPYYMPSGRQYTQAFYLQDQIKWKNIIFSTGVRYDHINNIGQKNLALKYNDI
SAGHDYSQKNYNGWSYYLGLNYDVNHYLSLFTNFSKTWRAPVIDEQYETQFKQSSVPATSLNLEKEMINQTRVG
GIITLNHLFQENDAFQFRTTYFYNRGKNEIFKTRGVNCVGNAADTNNKVCPKIIENYRNLPGYVIQGAELEAYYQST
YLFGEITYSYVKGKRDTSPRNPWGKTSTWIAEIPPRKATTALGFNVPKYYLTVGWRAEFVRRQDRSPLSGDPKAS
SWSLPASRGYSLHNLFLSWSPAKIKGMNVKITVDNLFNRAYNPYLGELASGTGRNIKFSLSQKF

tonB flanked by motor genes

>AAC21917.1 tonB protein [Haemophilus influenzae Rd KW20]

MQQTKRSLLGLLISLIAHGIVIGFILWNWNEPSDSANSAQGDISTSISMELLQGMVLEEPAPE
PEDVQKEPEPEPEPGNVQKEPEPEKQEIVEDPTIKPEPKKIKEPEKEKPKPKGKPKGKPKNK
PKKEVKPQKKPINKELPKGDENIDSSANVNDKASTTSAANSNAQVAGSGTDTSEIAAYRSAI
RREIESHKRYPTRAKIMRKQGKVSVSFNVGADGSLSGAKVTKSSGDESLDKAALDAINVSR
SVGTRPAGFPSSLSVQISFTLQ

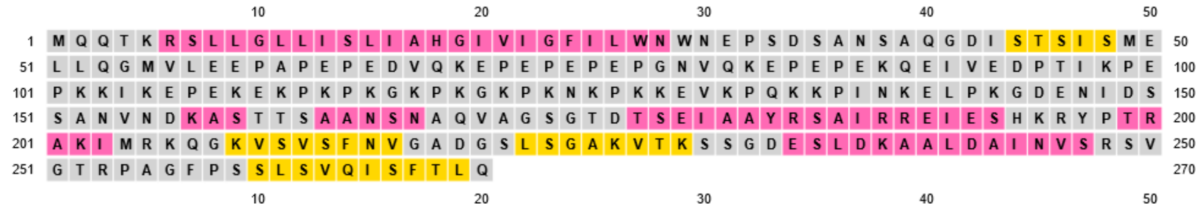

MQQTKRSLLGLLISLIAHGIVIGFILWNWN
**EPSDSANSAQGDISTSISMELLQGMVLEEPAPEPEDVQKEPEPEPEPGNVQKEPEPEKQE
IVEDPTIKPEPKKIKEPEKEKPKPKGKPKGKPKNKPKKEVKPQKKPINKELPKGDENIDSS
ANVNDKASTTSAANSNAQV**
AGSGTDTSEIAAYRSAIRREIESHKRYPTRAKIMRKQGKVSVSFNVGADGSLSGAKVTKSSG
DESLDKAALDAINVSRSVGTRPAGFPSSLSVQISFTLQ

PPIIPRED:

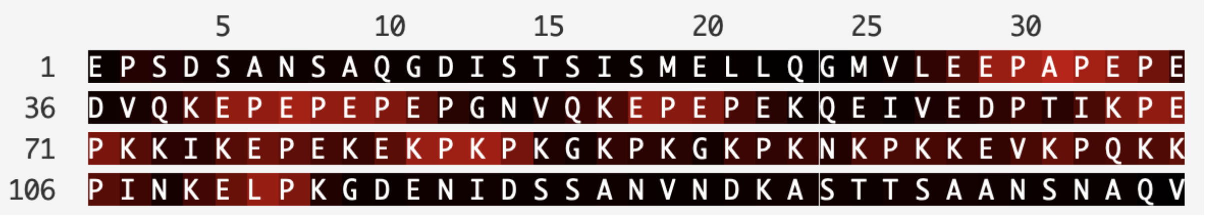

140 Domain II residues

22 Proline residues

8 α-Helix residues

Pasteurella multocida

>AAK03706.1 HasR [Pasteurella multocida subsp. multocida str. Pm70]

MQKQQPYPIHLGIFLMLGLPTWAFSQANLEKSTINKLETILVNESEEKNKFDENLIKTYLSSGSYSYLSQSDISTFR
GSSVGDFLSGVPGVIVGNKRNSGALSVNIRGIANENRVPVWIDKGLQSVPSYQGYAGSSTRTYLDPDLISQVEIEK
GPSLQMDATGATGGVVRVETLRWQDIIPQGKNWGVRLKLGTMTNTVSPPPYYTRGGYQTKYISKCLSNHTGLC
QTQTYAPNARYSSHGFDLNAYNYSLAFANKWQNADLVLAYAKRKQGNYFVGRHGQTPVIESIEFEEDSVEVKEP
RVHEEVEIGSLTFKENRSTLYRPGEEALNTSQDNTSYLAKINVYNDVHRLGLAYRHYHSRFGEIMSSILNFRAYGA
LQGEGTEVKVDSYHANYSYNPTTPYVNLNVNAYFTDSDSSNFTPFIEEYGYSLSSRHAHFLVSKQKGLSIENTSIF
QLNDKPFSLKYGLAHSYERIYQPRNAQARVRAKGYPEDAIGPLYIRDGKRKEWSAFVAANYPITSWLKADIGLRYL
QSTIYDYIVRTERVNIGGAFVPNPNGPGNIWVEKYKDVVHKQAPVKNKGMSPIVMLTFEPINGVQIYTKYAEALRS
PSLFQATKGWSMSATADNLEQLRPERAKNWEAGINLFYENLGGKDNILGFKLAYFNNRIKDYLTRSYSPKDKVTQ
TINIQSAQFKGIELSAYYDMGKFYAKLAGTYYTKTKFCLTAEQAGKGEQCNSGYIYRSNLNNAVPPRLNLHATLGT
RLFEQKLDIGARYSYYSKRLVPVLSAERFVNTSSIEWAPYSLVDLYANYNVSNNLKLTMTMDNVFNRYYLDINNM
GLNTAPGRTLHLGLEYRF

tonB next to motor genes.

>AAK03272.1 TonB [Pasteurella multocida subsp. multocida str. Pm70]

MIDKSRSCIGFAISLLFHASFVSFLYWIVQKDDDSANGFAADIISTHISMEMLAATVLEEPEPE
PEPAPPVVEPELPKEEVADPTVKPEPPKEPEKPKEPEKPKEKPKEKPKEKPKKPKKEQRDL
PKSDRQIDSNSSINQQATTTGNITTNNPNLVGKGNSTDEVNAYRSALRREIEKHKRYPNRAR
MMRKQGVVTITFHLNNAGVISNARISKSSGSEELDNAALVAVNNARPIGPLPAGMPNEVSVP
VSFRITN

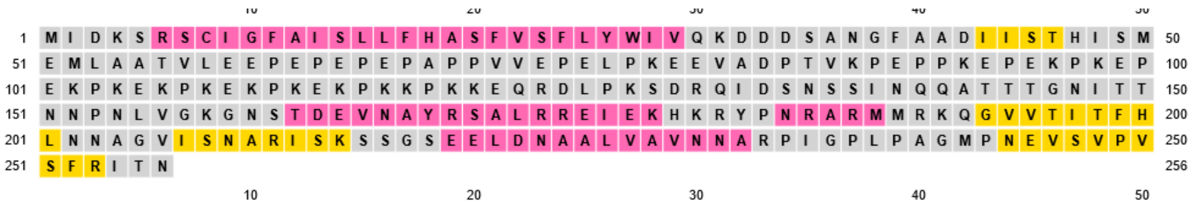

MIDKSRSCIGFAISLLFHASFVSFLYWIV
**QKDDDSANGFAADIISTHISMEMLAATVLEEPEPEPEPAPPVVEPELPKEEVADPTVKPEP
PKEPEKPKEPEKPKEKPKEKPKEKPKKPKKEQRDLPKSDRQIDSNSSINQQATTTGNITT
NNPNLV**
GKGNSTDEVNAYRSALRREIEKHKRYPNRARMMRKQGVVTITFHLNNAGVISNARISKSSG
SEELDNAALVAVNNARPIGPLPAGMPNEVSVPVSFRITN

PPIIPRED:

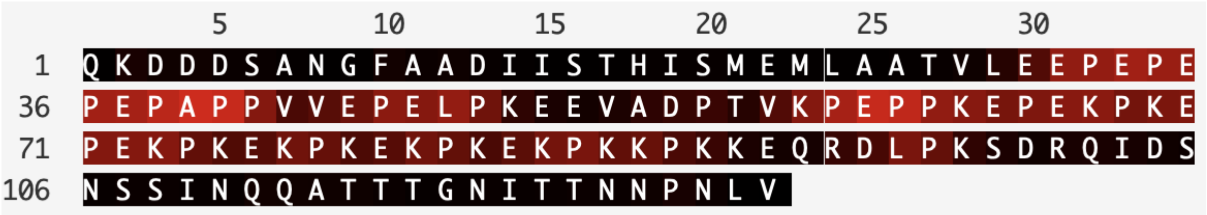

127 Domain II residues

22 Proline residues

0 α-Helix residues

Vibrionales

Vibrio nigripulchritudo

[Locus]

>CCO59343.1 putative Outer membrane vitamin B12 receptor BtuB [Vibrio nigripulchritudo]

MKKTLLATTVASLLTHASISVAQEAAETETLVVTANRFEQSTQSTTLPVVVVTKAEIEAIQANDIYEVLRRLPGIQVG
SNGGYGGQSQSLFVRGTNSDHVLVLIDGVRTSSATTGQANIAAIPLVGVQRVEFVRGTRAALYGSDAVGGVINIITD
TSVSEAKANLGFGSNNYQKASLAVSGEIGESTSGSFAAKFAETEGFSAQNSSGNEDADGHKVAEFAASLTHQLN
NEISFKFNSTYSDGEVEYDPADSKKDQQLYSIVGSANYSGEKLSSKLAVSKAQDSSYYPSFNGTFQTDSQSVAFT
NLYSVTSEISVGAGVDWYKDDISETSTKYDETSRTNKSAYVSGFFDNQTFQAELAVRGDDNQRYGKNSTWQVG
LGYKVTDNHRVTANAGTAFKAPTFNDLYYPGSGNPDLKPEKSTNYELALEGRQSWIDWRVAAYQNKIKDMLIGW
PAKNVGEAEIKGVELSGLFETGAFSHEVSLDFMDPKDKINDVQLSRRAKQSAKWNATYVADEWQADISYLYQGK
RVDNDGKTELGAYSLVDVAASYFVTEQLTVRGRIANLFDQNYVLAKGYNTQERSYFVNLDYKF

tonB next to motor genes.

>CCO56590.1 TonB2 [Vibrio nigripulchritudo]

MKRLFLALPVAFIVSASLFYFMAWMVSSQNGKLEESKPSVMFDLVMQEPEANVQRRQREL
PEPPKLPDQPKPLPTSTPNVAKAQVPQTLTAPKLALANLGVAVSMPSLDGIGQDQQALPLY
RVEPRYPPRAQKRRMEGYVVLSFTIDEQGRPEDINLVEVHPNKIFVREAMNALKRWKYQPK
LEGGKAVKQLDQRVKIEFKMSK

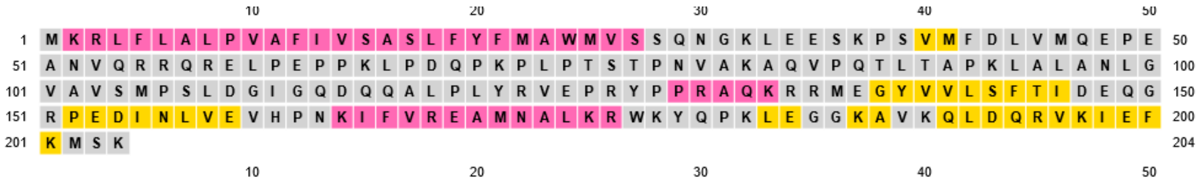

MKRLFLALPVAFIVSASLFYFMAWMVS
**SQNGKLEESKPSVMFDLVMQEPEANVQRRQRELPEPPKLPDQPKPLPTSTPNVAKAQVP
QTLTAPKLALANLGVAVS**
MPSLDGIGQDQQALPLYRVEPRYPPRAQKRRMEGYVVLSFTIDEQGRPEDINLVEVHPNKI
FVREAMNALKRWKYQPKLEGGKAVKQLDQRVKIEFKMSK

PPIIPRED:

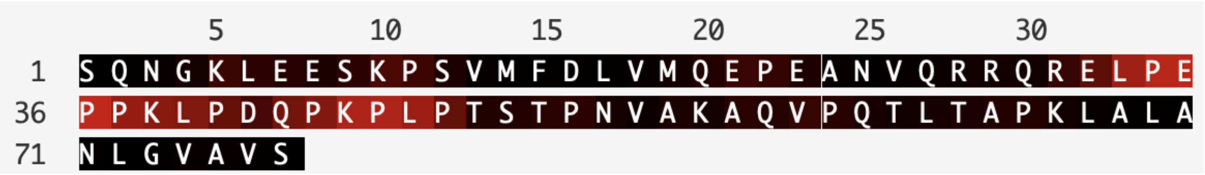

77 Domain II residues

12 Proline residues

0 α-Helix residues

Aeromondales

Aeromonas hydrophila

Locus

>ABK39619.1 vitamin B12 transporter BtuB [Aeromonas hydrophila subsp. hydrophila ATCC 7966]

MSKKLLAAALLPTAAFAQTTIPVNPTLVITANRVEQPISSVLAPVVVIDRAEIESRQVQSLPALLKTLPGVQITTLGGR
GHMSSLFIRGTNSNHSLVLMNGRPIAAMVAGTPDLSQIPLGNIERIEYIRGPRAAVYGSDAIGGVINLITKTSAKNGS
ETHLKGGVGSNGYGQGQLRTVQALGQQTDMNMMIGYERTDGFDVVANAQQPDRDGFDSLNGQFGLSHAFND
AWSADFNAQGYDNQTEMDDAYQSADQSRVQAFQYDGGLKYQSGALSSRLEASYGENKLKSWLESKGESSAEP
IHTGLTRFSWINSWSGVEGLNLTGGADWQQEQLKSDTRSYGQAFNAPDRDNTGLFVVGSYRWQALLWELSGR
TDDNKQYGRHNTWSAASGLDLDDNHNVRLSYGTAFKAPTFLDLYYPGYENPALKPEESKNLELGFSGRYTGWD
WSLNLYRNQIQNLIACQSASSTCRPDNTDAEIRGVEVALGLETGPLRHDLSFDYTRAEDKNDGDQQLLRRAKQK
ASWLTQVQLGPVDLSTELLYVGKRDDKNFSSFPAERVELGSYTLINLGASYGVTPQFTLGGRIDNLFDRDYAPAY
GYASAGTEFKLTADYRL

tonB next to motor genes. Two copies of *exbD*, very large *exbB* gene, predicted to form long periplasmic extension.

>ABK37434.1 outer membrane receptor-mediated transport energizer protein TonB [Aeromonas hydrophila subsp. hydrophila ATCC 7966]

MKYKLMSLGLGIALSLGILLFMATLVEPPRGEKASAEQKSIVINMQNEVTEVQVRERPIPQEP
EPLPEPPAALASTPLPMPAAAPLPSVQPSLDLVSSLSAVQVYAPGVATSPTPVASGNFHGQ
QQGAGIGAGDMLMPLQRIEPVYPYRAQQAGIEGSVTLRFSVDADGRVQDVEVVEAKPKRQ
FERAAIQAINKWRYQPRPGATDKLVQIITLKFKLES

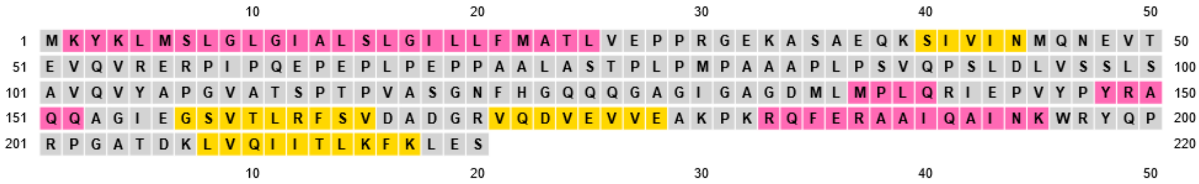

MKYKLMSLGLGIALSLGILLFMATL
**VEPPRGEKASAEQKSIVINMQNEVTEVQVRERPIPQEPEPLPEPPAALASTPLPMPAAAPL
PSVQPSLDLVSSLSAVQVYAPGVATSPTPVASGN**
FHGQQQGAGIGAGDMLMPLQRIEPVYPYRAQQAGIEGSVTLRFSVDADGRVQDVEVVEAK
PKRQFERAAIQAINKWRYQPRPGATDKLVQIITLKFKLES

PPIIPRED:

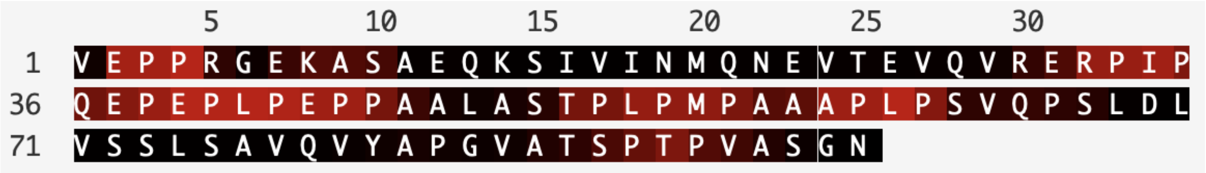

95 Domain II residues

18 Proline residues

0 α-Helix residues

Alteromondales

Shewanella oneidensis

Locus:

>AAN53891.1 TonB-dependent vitamin B12 receptor BtuB [Shewanella oneidensis MR-1]

MGTHPTKIALLIGSLCSFSIIAPVMAADDTSTAKVDEHLVVIGRSDKTPLNIAANVNVIDAAAIEMSGATNLTDVLRG
QSGIQISDNNIGTSFAMRGFSASTAVNNTLILLDGRRLNNIDIAAPSLNAIPLNLVERVEILSGSAGVLYGDQAVGGV
INIVTKSPENTGGSVQLSGGSFNTYEGRGDVSGAINKDWRYFLSGSYNQGDNYRQHNANETGSILGRIQYKTAT
DSFFVETSYYDDDRENPGSLTVDQFKFDPRQSSNKSEYVHEMTTAARSGYQHQLNQNWALAADLDYSDTLVSS
VNWGAGSHNTRSLLMFSPKALANYSTRQGELNLVTGLDISRGKADYDSMARSNVQDMQSAYLQATVPLSHTLS
YVVGGRYARVTDELVDGNVYPNGIDLDEDATAFEFGLNYRPSAEHRFYLRGDENFRFAKVDEQAYTPKDVFGLK
PQTGRSYEAGWDWTVASQSLRVSLYRLDLEDEIVFDPSAEKPVGGSFQGANVNADASRRYGASADWDWQVTQ
ALQLGLEYNYIDAEFTDGLNDGKKLSWVAEHSGRGYVSMDVAEHIQLFAEAIYTGERFVEGDNANQGEKLASYVL
GNLALNYSRDAWLASLRIDNLFDKDYVSSGYYGGNWGDGYYSGRGRDIRLTVGYRF

tonB not near motor genes.

>AAN57525.1 TonB mediated energy transduction system energy transducer component TonB [Shewanella oneidensis MR-1]

MGQNVIVNRTGIIVVSALIPLGLFVFMAQLIHNPQPLSGQATDAPQINILMSERTPIPPKESRK
PEPPKPIPTRERITTPGESTEVVDFNPQTFTPEMPIQTTLFTQSSMSAEALPLVQVSPRYPIE
AAQNGKEGYVVVGFDITADGTVSNVRVLDANPKRIFDKEALSAVQNWKYKPKFDAGKAVP
QLNQQVQLDFKLDQKI

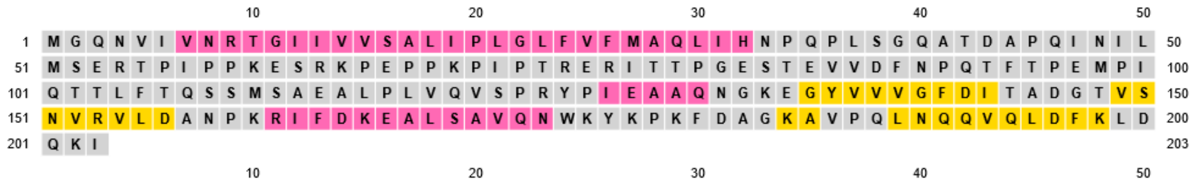

MGQNVIVNRTGIIVVSALIPLGLFVFMAQLIH
NPQPLSGQATDAPQINILMSERTPIPPKESRKPEPPKPIPTRERITTPGESTEVVDFNPQTFT PEMPIQTT
LFTQSSMSAEALPLVQVSPRYPIEAAQNGKEGYVVVGFDITADGTVSNVRVLDANPKRIFDK
EALSAVQNWKYKPKFDAGKAVPQLNQQVQLDFKLDQKI

PPIIPRED:

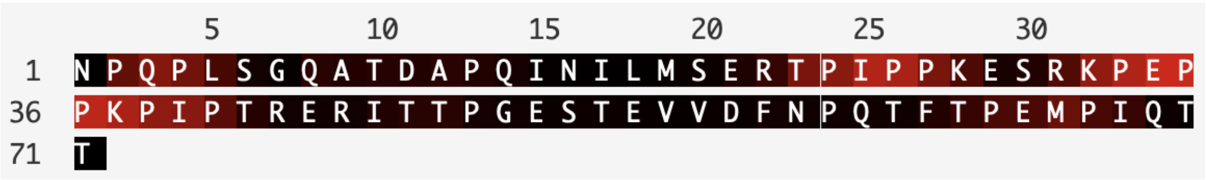

71 Domain II residues

15 Proline residues

0 α-Helix residues

Ferrimonas balearica

Locus:

>ADN77205.1 TonB-dependent receptor [Ferrimonas balearica DSM 9799]

MKYSALSVALFAAAPLAWSAPAVDVDETLIVTGDRLAQSVDSVLAPVTVIDRASIVAMQAKSLSDILRTLPNVDVN
QYGGRGQNATVTVRGATSAQSLVLIDGMRSTTSALGPLNINSFPVAEIERIEFIRGARASVYGSEAVAGVINIITRD
GRDGTTLTAGAGSFEQVEASVRHQSQLAGGTLKAVLAYEDEEGYNVHPVPGVNDGDKHGFTGKSALLAYDRAV
TERLSLYAAARWFQNESQYDNSSLGNPAWGAPDVRERKENRFENVDYQLEARYQGERWHSSVQAQYSDSES
QDFVDTLSYKDSPDFSHLRQYNLAWVNRVQLTDAISVGAGLDWRDEQLRGDSTYLDWATGVATPFADGTLSRD
NTGAYALLRVEEGGHQFEASVRSDDNEQYGQHTTWQVGGRIAATESVALVASVGTAFRAPTFYDLYYPGSGNL
DLRPETAEHYELALEGAVASIDWRLGWYRQDAEDLIQFDSLTYRPVNIGEAVIDGIEAEAAFDTGPVHHQLSYGW
RDSEDRTTGNELAASARHNAKWNLSYDWDALNLTGNLVYRGSRWGDAANTVKLDAYFLVNLAASYQLTESLAL
RLRLENLLDEEYVTIADTFSGGSYPGQERSVYGQVEYRL

tonB appears to be near a heterologous motor, where one of the two copies of ExbB has an extended periplasmic domain.

>ADN75628.1 TonB family protein [Ferrimonas balearica DSM 9799]

MRDYLLALCLGGAITAGLFSLMAMMVTGGPGKAPSEPGPAPVSVLMADRPEQLNRRSRAL
PEPPPPLPELPQAQVVENSSATNVQMNLDLPTLSIDGPGDIQIGMPAKADLATADRQAMPL
YRVEPAYPQRALRMRAEGYVLMEFTIDEQGRPRNIRVIESEPVRLFDQAAMKALARWKYQP
KQLDGKAVSQPGQTAKLEFKLNR

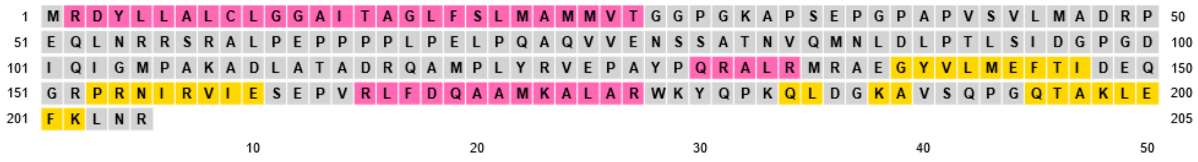

MRDYLLALCLGGAITAGLFSLMAMMVT
**GGPGKAPSEPGPAPVSVLMADRPEQLNRRSRALPEPPPPLPELPQAQVVENSSATNVQ
MNLDLPTLSIDGPGDIQIGM**
PAKADLATADRQAMPLYRVEPAYPQRALRMRAEGYVLMEFTIDEQGRPRNIRVIESEPVRL
FDQAAMKALARWKYQPKQLDGKAVSQPGQTAKLEFKLNR

PPIIPRED:

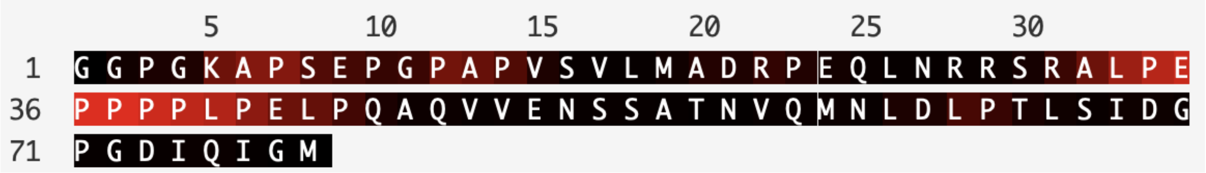

78 Domain II residues

15 Proline residues

0 α-Helix residues

Idiomarina loihiensis

Locus:

>AAV82015.1 Outer membrane protein [Idiomarina loihiensis L2TR]

MNTNTFRKSVTALAVTATLGFPAFVAAQDSGANDVERIQVTGSRIKRTDMETASPVSVFDAEAIEASGFTTMENFIHNLPSMNGA
MNGSSTNNGSGGYATANLRGLGSARTLILINGRRYASGDLNAVPMAMVQRVEVLRDGASTIYGSDAIAGVINFITKTDFEGAEFS
AQYDLTGEGDGETTKLSGVIGTSSGKGSVVLGLEYQNRNAIGQADRDFSRIPLAEKNGKAVFDGSTINEFGSFVPTGEKKTYVLD
PQTGEQRLFNAQKDGYNYATDSYLKSPQELFTINAVANYDITRDLRVFLEGGYANRQSNQLLAPTGTFWGAPVDADFEYNKTG
KDLSVYRRLTETGGREYTQDFSDYRMVLGFEGYLDNGWSWDISYNYARYVDTTVEYNQANRTRFNTLLDEELCNSDEECPGIW
NPFEADSLTDEMMDYALIANSPVVRGTTKQFMANLTGDTGSWSLPAGSIAWAAGVEKRWESYENQPDGAAAIGQIYSTPGIPSE
GSYDVEEAYIEVDAPLLRDLPGVQSLDLSAAVRRSDYNFLDAQTTTKFGLEYVPKDGLLVRATIAEGFRAPTIDNLNSPQVISFSR
YVDPCTEYGDSSNDILDANCKADGLDSDFTPKTSQSATILSGNADLKPETSDSFTLGVVYQPEFMDNLNVAIDYYDIEIENGIGAP
DISLIAEQCYTSENFSSASCDLILGPEAVDTKSWPSSDYRDAQGTLAGTDATTQNISTFETSGIDFDVNWNDEVAGGLLNLRLDG
TYLKDYNYQVQEGAAPLELAGNFGADSNFAGRIAAFSKWRTNFTASYKVEDVAVTWISRFQSGVDDVNYDSSDLSSSVGSYIYH
DMQASYFFNSETTFTLGIRNLFDKQPPYVSNNNDMNTLNTSYDTAGQYWYARVGFRF

tonB next to motor genes, heterologous motor observed with large *exbB* genes and two *exbbD* genes. ExbB-associated gene ExbB

>AAV82687.1 TonB-like periplasmic protein [Idiomarina loihiensis L2TR]

MVRFLVSLLLGAAVTFALFAFMAYLIGGGAQRNEAPAPTPVIDIVTSPPESDVQERRRTPPP
PPPPPEQPPETPQSEPDTSDSGMNMNMGFDVDVGGADTGLSGPGGGLSSDGDATPIVRI
EPRYPPSAARNGTEGWVQLRFTIDQQGGVTDVEVIDSEPRRVFDREARRALLRWKYKPKV
VDGKPIRQEGMTVQLDFTMDGN

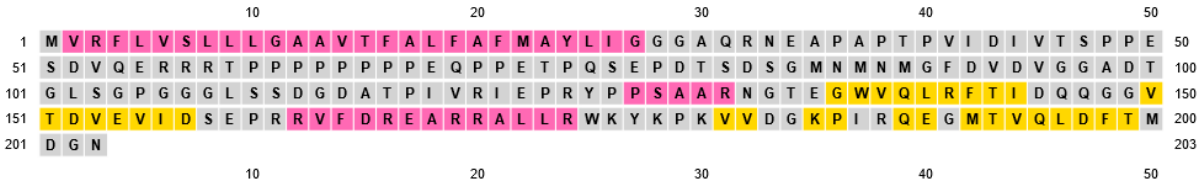

MVRFLVSLLLGAAVTFALFAFMAYLIG
**GGAQRNEAPAPTPVIDIVTSPPESDVQERRRTPPPPPPPPEQPPETPQSEPDTSDSGMNM
NMGFDVDVGGADTGLS**
GPGGGLSSDGDATPIVRIEPRYPPSAARNGTEGWVQLRFTIDQQGGVTDVEVIDSEPRRVF
DREARRALLRWKYKPKVVDGKPIRQEGMTVQLDFTMDGN

PPIIPRED:

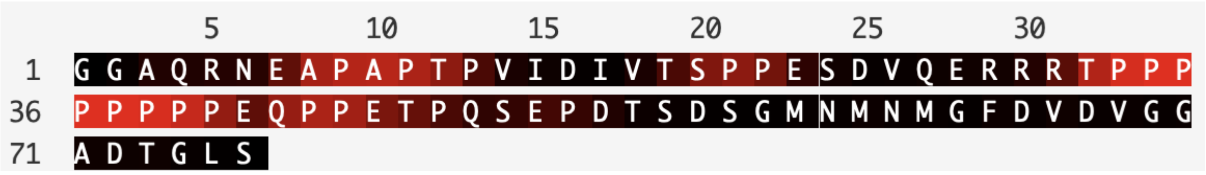

76 Domain II residues

17 Proline residues

0 α-Helix residues

Colwellia psychrerythraea

Locus:

>AAZ28743.1 tonB-dependent vitamin B12 receptor [Colwellia psychrerythraea 34H]

MNKTIIALSISSLLYSSLSFAEQTSPTTIDKADETIVITANRSIQDKFDTLASVNIFTRENIEQIQPLSISDLLGRVAGISI
NTQGTSAHKTSAFVRGTNSDHVLVLVNGVRIGSATLGSKSLASIPVQLVERVEVIKGPRAALWGSDAIGGVIQIFT
RDLAAGEGQVGIKVGSDSLYQGYGALGFGNKEHGYTLSATAEKSDGFDVVVPDGNSQYTVDQNDDDGYDRQSI
ALSGTSQFTQAYALELNTQYDQGTTEFDANTKYGGDETTYENYHFLLRNHLQLEQVYLQLGLSTSEDSTEDNYS
KYNSQNSASNANSLFTTNREQVTALAQLPLADSSEITAGFEWYTESIESNKEYDETDRDANAVFVTGRHDIEQVK
LEASVRYDKVGDIDSETTYQVAAGYQVNDQLLFSLSHGTAFKAPSFNDLYYPWQGNPDLISETADTSEFLARFQN
DTLSAEVSIYQSDIDNLIEWAPTASGAYKPSNVAKAKILGAEATFTAAIYNTNNTLTLSHVDAEDKTTDHQLARRPN
FSANYNFVYSLKQFDFTFDVDYQGSRYDGANDPVKTLKAFTLLDVGLNYRLNNQVSLLAKVTNLTDKDYQTSSEY
PGSERGYTLTVDYKF

tonB next to heterologous motor with periplasmic domains. ExbB prediction.

>AAZ26539.1 putative TonB protein [Colwellia psychrerythraea 34H]

MVRFLVSILLGVVVTFGLFAFMAFLVSSGDRNKEEQQENIIVEVNTTPPKSAAEQRRRVPPP
PPPPPKAPPKQQTPEPEANNNTGGLNFNMPGVQMAGASTGLSAPGAGMGRDGDATPIVR
IEPKYPIQAARDGKEGYVILSFTINKIGGVEDVKVLEAQPKRVFDKEAKRALRKWKYKPKVVE
GKPLRQPGLTVQLDFKMGG

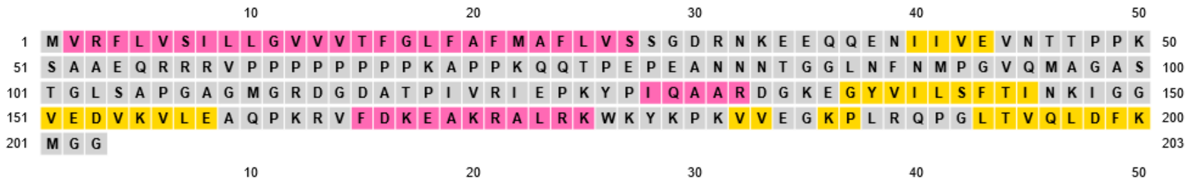

MVRFLVSILLGVVVTFGLFAFMAFLVS
**SGDRNKEEQQENIIVEVNTTPPKSAAEQRRRVPPPPPPPPKAPPKQQTPEPEANNNTGGL
NFNMPGVQMAGASTGL**
SAPGAGMGRDGDATPIVRIEPKYPIQAARDGKEGYVILSFTINKIGGVEDVKVLEAQPKRVF
DKEAKRALRKWKYKPKVVEGKPLRQPGLTVQLDFKMGG

PPIIPRED:

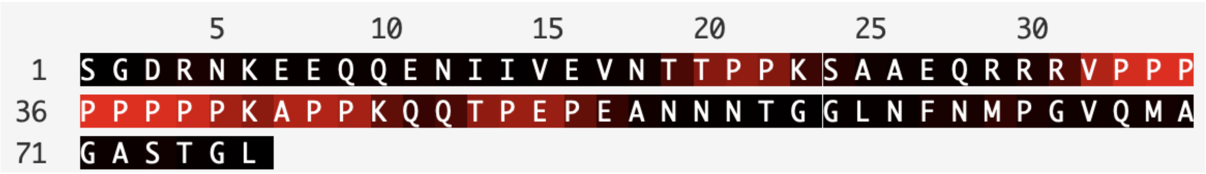

76 Domain II residues

15 Proline residues

0 α-Helix residues

Alteromonas macleodii

Locus:

>CAI3964402.1 iron complex outermembrane recepter protein [Alteromonas macleodii]

MFSFLLDSSFSVSSKSSSGLSVSIAALPLLLTSSFSVAQGAPLNEVERYTVTAARPYYDETLSRIFPQYEFDKSGLV
APLHTNDVLLQSPSVSLNGQGGQIQSISIRGYSRWRIQTLLDGVPIVSDRRAGSSIGFIPPDFISTVSVLPGAASTYL
GSGAIGGAVNLHFGEIQKPHLRVGYSSNQQMKALSYAGSTRRADNSNSLTEVSETDWNISYRSADNGEDANGG
SLFDQFEQSGLFVRHRPVDSVIKEAWTLYSDNNDIGKSSSDFPESRITTYPDNTHWLGKIAFESHLFTGNVWWHK
SSLETSVLRPESRINDSENKAFDYGFNISTDTQLKDWDLNWQLQVTGRDGVVADEREFTLTPAERALPDTTSPSF
ITNAFEAELAYEVRTLDASEITTAAVADASRQWQSLSLALGARVDWQRQSDDSDANTFQSSTAQTNTNLSGYLG
ANYQLSQHWAAGMYVSSAFRNPSLTERFFAGETPRGTVLGSLKLETEQALNMQASVAYSSEQLQGSIEVFNQKI
DNYIERIMVAEDVLQYSNLDSATIEGVSYQLSWQSHNDALDARLSGMWISGEDDLGNSIADIPANSQRLDLGVQW
QAVRFFTIFAYRASKTDIADGERALDEVFTLDMGADWQLNERVQLQASWRNLTNQQYYTSADDKAAFAQGESV
QLAITYLL

No motors near *tonB.*

>CAI3955250.1 protein TonB [Alteromonas macleodii]

MQTTILPNANPKHLTHQSVLPFEKAPFAKRALNAATIAIAGCTVTAGLFVLMAELIKQDKVNIS
EAPPVFFEPIIYQPEDERTITKKPMVPIEQVIKTPPQKVIEIDPEPNEGPGGFEIKTHIPNTGLD
LSINLNSAQGDMQATPQFRVDPTYPPEASRDGIEGWVKLGFTISASGSVSDITVLDSEPKRV
FDRAARRALKKWKYKPKLNDGKPVSQSGMVVVLDFKLEQ

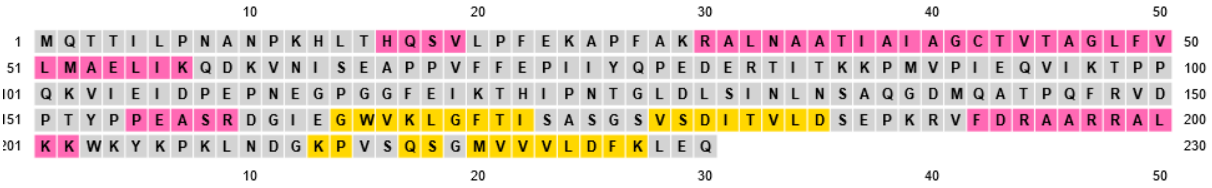

MQTTILPNANPKHLTHQSVLPFEKAPFAKRALNAATIAIAGCTVTAGLFVLMAELIK
**QDKVNISEAPPVFFEPIIYQPEDERTITKKPMVPIEQVIKTPPQKVIEIDPEPNEGPGGFEIKT
HIPNTGLDL**
SINLNSAQGDMQATPQFRVDPTYPPEASRDGIEGWVKLGFTISASGSVSDITVLDSEPKRVF
DRAARRALKKWKYKPKLNDGKPVSQSGMVVVLDFKLEQ

PPIIPRED:

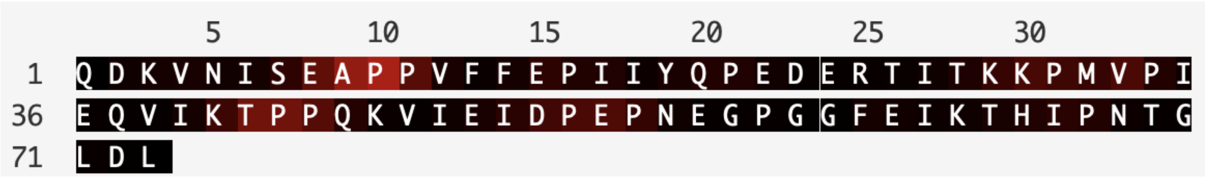

73 Domain II residues

12 Proline residues

0 α-Helix residues

Pseudoalteromonas atlantica

Locus:

>ABG40383.1 TonB-dependent receptor [Paraglaciecola sp. T6c]

MTKHADIHRKCRLSALIDESSHTKGGKNKNQVSNSIKHTLSNTLRTVMFRESVKKRIPVFQLSMLAVTSLVAASIAE
AKAAEANTAKANMFKEDRSESQVTTTDISDVIVVTGTRQAENLLTLTGNLARINRQDIQTVSSVYPSDLLNRASGV
YVQANNGMESLPSLRSPVLTGPGAGGAFLILEDGIATRAAGFANNNGLSELNLAQAQEVDIVRGPASAIYGSNAV
HGVVNVINQAVRDGGDVSLLLGPNEHYQVQGTLGNDFGAHGLSINTQLIDNGGYQDDSDFTSAKVGLRHEYRDD
LNAITTSVAGFVLDQNTAGFIASGDNGEGCFESTYADDRLFKDTQAMEKNCEDDAYRKWSSMRVASKWQRTLS
DDRYFVLTPYVRTNDMEFRQHYLPSEAIEENSHSSFGLTSSYHWQFDPRLALVMGADAEWTSGELTETQQKAST
FSFGKARQQGVHYDYEVDVLTLAPYVQADWQYSHALRMSASVRFDSTHYDYKNRLADGTTQADGSPCVNGNN
EPVACLYQRPADSKDDFDNVSSKIGFNYLMNESVALFADWSQGFRAPQTTDLYRIQNQQVAGQIESEEIRSQEV
GVRGLALDMNFELVAFYMTKDHFFFRDDDGLNVTDAETSHKGVEVGLDYPILSNVSIAVNYTYAIHQYESEHANS
GVLKGNDIDTAPRQLGNVRLAWQPTEDSSLELEWAHVGSYYLDPSNEHEYQGHNLLQLRGQFSLTDKVRLLARL
ENVLDERYASRADYAFGSYRYFGGQPRAIHLGIEARF

tonB near heterologous motor where ExbB has periplasmic domain.

>ABG41494.1 outer membrane transport energization protein TonB [Paraglaciecola sp. T6c]

MGRLIVSVLLGGVIAFVLFVVMAKLIANSSRPADKVPPAPVIDIVMSTPDDSTQTRTRVPPPP
PPPPQQPPKLEPVEPDTADANTDGLSFNMPAVDIGGASVDIGGVGAMQRDGDATPIVRIEP
KYPAQAARDGKEGWVKLSFTINEVGGVEDVDVIEADPKRVFDREAKRALRKWKYKPKVED
GKPMKQFGMKVQLDFKLDQS

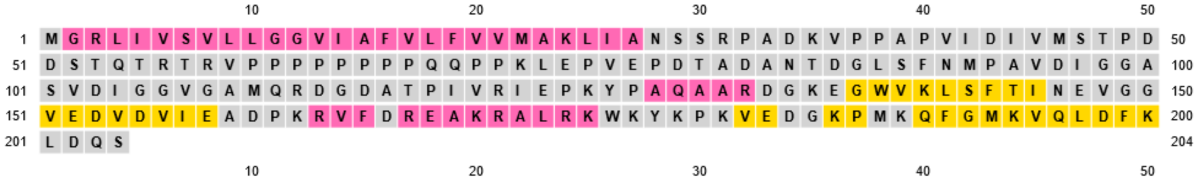

MGRLIVSVLLGGVIAFVLFVVMAKLIA
**NSSRPADKVPPAPVIDIVMSTPDDSTQTRTRVPPPPPPPPQQPPKLEPVEPDTADANTDG
LSFNMPAVDIGGASVDI**
GGVGAMQRDGDATPIVRIEPKYPAQAARDGKEGWVKLSFTINEVGGVEDVDVIEADPKRVF
DREAKRALRKWKYKPKVEDGKPMKQFGMKVQLDFKLDQS

PPIIPRED:

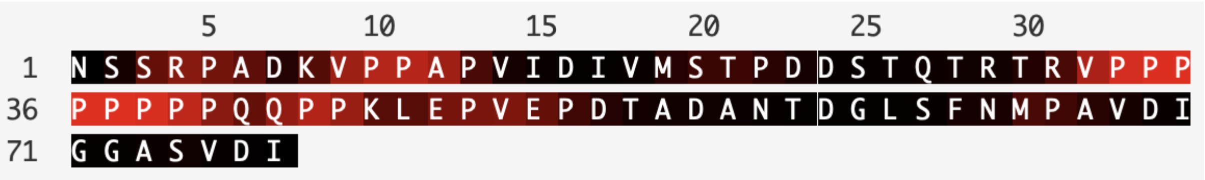

77 Domain II residues

18 Proline residues

0 α-Helix residues

Iteromondales

Psychromonas ingrahamii

Locus:

>ABM05027.1 TonB-dependent receptor, plug [Psychromonas ingrahamii 37]

MPRLYYTPIAFALTTLFSATSIAAAPPISEQDTRDDIVISASRVETKRIESGSSVTVLDEQYIKENQARTVAELLQDVP
GVSVASNGGLGQATSVFIRGANSNQTLVIIDGIEVNNLGNFEGGYDFAYLMADNIERIEVLKGSQSALWGSDAMG
GVINIITKKGKAGFHPTASIEVGGNNYHKENVTLSAAQGNSHYSLSASNMKTDGISATDTDPDDDGYKNQSVSLK
AGHQFTDIFSMDTVLRYNDAETEYDSGYTTNSQRQAKLSSHLNLLNNQWKNRLSVAFSDSNTEDFSSWGDSKY
EGKKIKTDLQSDYYISAINGYTQRISFLAEHESDKYQSLSMTQDERIEASGVVLGYGVDWAKTIFVNVAVRSDFNN
KFDDTTTYHIDTSVWVNDGTRLHASHGTGLKNPNLGQLYGENASWGYVGNADLKPEKSRSWDAGVEYNFVGT
DAYIDLTYFDSLYTDMHTWSGSFPNSTYINLNNKATARGIEFTGKVKVSNKLRVNTGYTYMETNDGNGNELARRP
KHAASINANYKYTPELSANIGARYVGKRLDSDDSTLSSYTVVNISTAYQIQEHITLSARIENALDKDYQEVSGFNTD
PLTAYIGFSFK

tonB near a motor where ExbB has large cytoplasmic domain.

>ABM05033.1 outer membrane transport energization protein TonB [Psychromonas ingrahamii 37]

MKIISSYFPFLIISLVFYGFIFLYLDQPKRITTLPLNSGEQSVQVQFISQPNKGQNPAEDKLAAPTEQ
KIVEAKSYVTEEEVVERAKVKSKKVIKYSAKSLILSESEIKLRKQALLESFATAGTDNVAKLKAEIFL
EQLATEKILTNETEQLSKTKDQASSPQTSNGPAKKAVIKKSPSENSSSLASSTKNQGVLQEAIVV
SGRKPVYPQRAILRNQQGRVVIKLTITKKGLPKNPKILTSSGFPLLDDAVLAFVDQELFMPALQGE
DQVMSEQLFAFRFELN

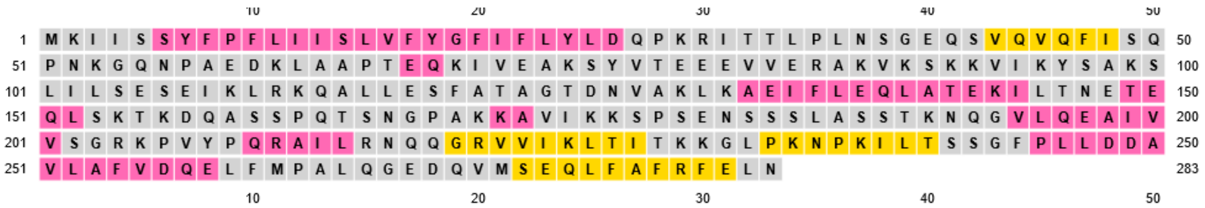

MKIISSYFPFLIISLVFYGFIFLYLD
**QPKRITTLPLNSGEQSVQVQFISQPNKGQNPAEDKLAAPTEQKIVEAKSYVTEEEVVERAKVK
SKKVIKYSAKSLILSESEIKLRKQALLESFATAGTDNVAKLKAEIFLEQLATEKILTNETEQLSKT
KDQASSPQTSNGPAKKAVIKKSPSENSS**
SLASSTKNQGVLQEAIVVSGRKPVYPQRAILRNQQGRVVIKLTITKKGLPKNPKILTSSGFPLLDD
AVLAFVDQELFMPALQGEDQVMSEQLFAFRFELN

PPIIPRED:

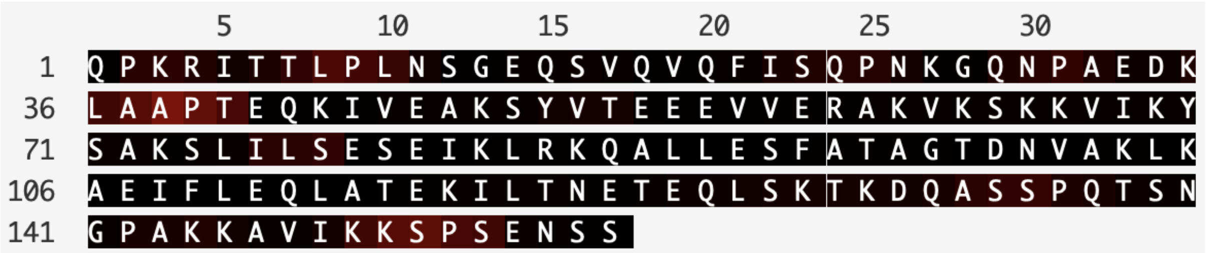

157 Domain II residues

8 Proline residues

21 α-Helix residues

Pseudomonodales

Psychrobacter arcticus

Loci:

>AAZ18822.1 outer membrane receptor for transport of vitamin B [Psychrobacter arcticus 273-4]

MVLSRSSSTTYLRLCILSAFGAFAMSANAATDVNIVSDSDLPQVELDKIVVTATRTPTKTSNVIAQTRVIDSEELQRYQGQTVTDVLKNQPGINIAQSGGMGTISNFYMRGYDSKQVLVLIDGIRYSSISAGTSSLNLLPADQIDRIEILYGASGSSIYGSDAMGGVIQIFTKGNIVEQSNISTSVGYGSNNHYQVGVTGQLKNDTSSLSLGVSRNKTDGFNAIADSSSSDYNADDDGFESTNASLGLQHKLSDSLSAGLSALYSDSTTDIDSAGTLFPNAYSDQKNGSANAFIQHQTPLTVTKFSYGQSIDRLTSHDNNSINYQQGSQFDTTQEQARLESQINAQPGTVTIGAEWLSQKLDASDVLDFSGYPNPAVQTAYNPDDRTVKSAFVGYQLSESYYDLQANYRVDDNSQYGNESTYNIGAAVRPLDGVRIGASYATGFRAPTFNDLYYPGYSNPDLKPETSKNTELFVEYNKGNQISRLTGYHTDVEDLIGGNTNTGEAKIKGLSLISDWRVNSFLFGLGYDYLDAKDKTANSANYNNDLVYRPQNSGLIYIGYQQPTFDVRLEAKHTDSRFSDVSNQTKLDDYTLVNLSGSAYITPNLRANLRVDNLTDENYTLSNQFGTEYATDGTNFFTSLTYNWF

tonB next to motor.

>AAZ19512.1 outer membrane transport energization protein TonB [Psychrobacter arcticus 273-4]

MSSMDLERPPLKSILIAILVVVGLHVLTAVALVAMKPSAPKIAPPKQTPPIEIELVSLPAALPKEIVKVAKPVKKEAAKVAIEKTAPVPKQQAIAKPKPFAEMSKQQVVQEKKPVIAPVVKKEKTTSITQEKPPQKQIQQEEPHPAIDKAISEQRANKPQANDQQAEQRRIVAAQAEKAAQEAQMQQAQAKEAQRVANAKAARDAQAKASAEAANDAAQKAVRAAKQVEGAIKAATGSNDPVNFTASSANWVSAPNFSFPERVARRANSGDTLNVVLVLRVNKQGGIDSVRIAQSSGIAALDKEAQRQVRSGKFKPFMKNGAPVVGNVTLPVTYIVP

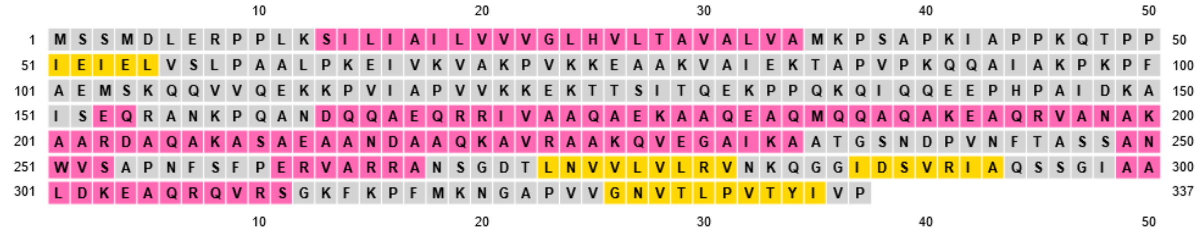

MSSMDLERPPLKSILIAILVVVGLHVLTAVA **LVAMKPSAPKIAPPKQTPPIEIELVSLPAALPKEIVKVAKPVKKEAAKVAIEKTAPVPKQQAIAKPKPFAEMSKQQVVQEKKPVIAPVVKKEKTTSITQEKPPQKQIQQEEPHPAIDKAISEQRANKPQANDQQAEQRRIVAAQAEKAAQEAQMQQAQAKEAQRVANAKAARDAQAKASAEAANDAAQKAVRAAKQVEGAIKAATG** SNDPVNFTASSANWVSAPNFSFPERVARRANSGDTLNVVLVLRVNKQGGIDSVRIAQSSGIAALDKEAQRQVRSGKFKPFMKNGAPVVGNVTLPVTYIVP

PPIIPRED:

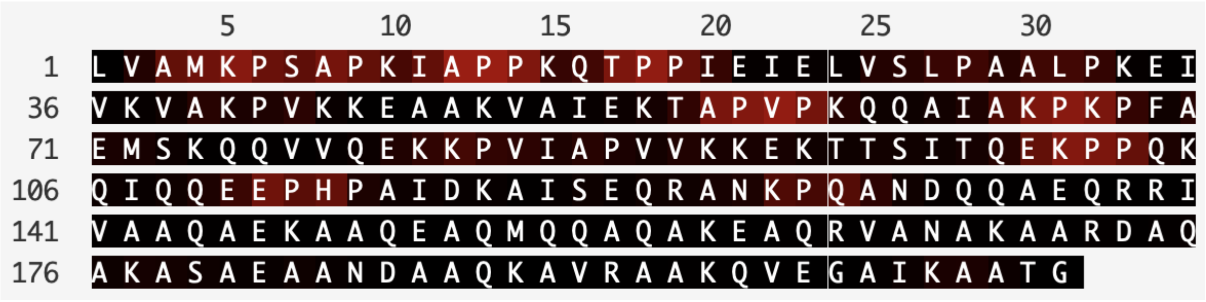

206 Domain II residues

20 Proline residues

74 α-Helix residues

Moraxella catarrhalis

Loci:

>AZQ93065.1 tonB-dependent Receptor Plug domain protein [Moraxella catarrhalis]

MIKKPLVCAISATFAMPAVADNTKLGEEPTTTLKGVLVSSQTNQNTGFVSNDSKQSSDLTLSKDKLKYRSATLGNALSGELGIHS
NPFGGGSSAPVVRGQEGVRLKILQNGTDVIDVSSISPDHVVATDTLLASKVELVRGADTLLYGLASPAGVINVVDDRIPNRMPSG
AIHDKIEGETMLRYNTNNHEKLATAGVSFGVGDRIAVRVEGLKREADDYQVPHFQADRMLDYVPGSANNSTVGMIGVSYIHDNG
HIGASYSHRKDRYGIPGHIHCDSQREHFIKWHNITKSNYYLPIYPHLMEDSDIDDNPHTHCRHNHEDHIGEHNPTGVPINHEHHS
PWIDMKTNRYDIRGEVYRPIQGLDKIKLSLTYADYYHDEKDAGNEQDPNNHKPSERDTTVDKGHASSIFTKKGVNGRLELYHTPT
KRLSGVLGIGYQTQKSAAGEAYLPSYFQSEAEWQKAQSQNINQYRPYLLVPNTNKSLGIFGLEQLKLNQMTFKVAMRHERQKTP
IEYDQHLLDHALQYFLSKAQLKAPDHPDLTTYKQHATSYAGSALWDITPNHRLSLTYSHNERIPSPMELYYQGGHLATSSFEHGN
KNLVKEKSDNYELGFMHTADKVSYKASTYYSNFDNYIFNETIAKEGNLYIRRYNQTTAKFYGVEGSLTYQPNANHSVMFFGDMV
QGKIGALSDIKGKLVYAGRKWVYFDDDIKDMTVDDNGDYDADGGLTCALKTPEQWGQINDNNDCSTTINVYKNGTTTSGEEDY
DRLARNPTYAPRVPPSRLGIRWQGYFGDHWSANAEFNHVFAQNKVATSTVAIKPQFKQPEGCQRHESHCRISDYGSDNNPLM
MQPRYITENKTAGYNLLNVGLDYNNAYRNVDYTLSIRANNLLNEQIYIHNSFLPFVPQMGRNLTLGLTAKF

tonB next to motor

>AZQ92463.1 gram-negative bacterial tonB family protein [Moraxella catarrhalis]

MDNSKKIKVLAISIAAVLHGLVGFGLANMTIKPITPPPVTPPLEIEFIKEPVPEPIALNDLESPEPPGEPVV
TPEPVVEPEVATPEVIEPPEPIPEPEPLPEPIPEPEPEPEIDVEAILEQQRLQEAWVAQQQQLAKIQEQ
ERLEQERLENERREQERLEQERLENARREQERLDNERRAREQAQKEAQVAAARRAAAQAAANAAK
KAGNHGGGQPGQNQTVEGGINISNASWKTKPRVNNFCSARSDIDTTLQVSFRVDAEGKISNVNLNG
STGDAKLDRQIIRQIGRGRLHPFREGNITRVGTAIYPITLKLQKDESCTN

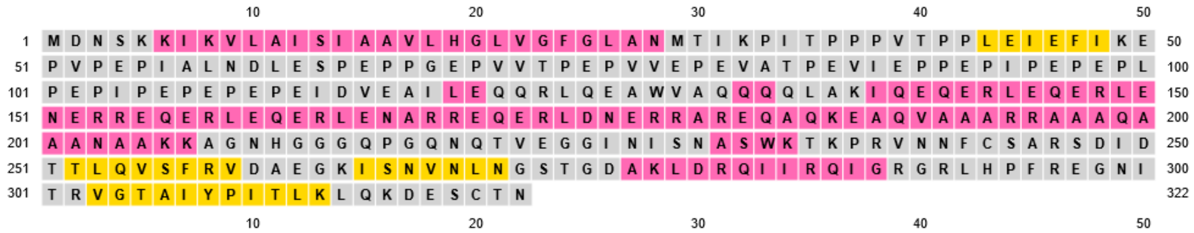

MDNSKKIKVLAISIAAVLHGLVGFGLAN
**MTIKPITPPPVTPPLEIEFIKEPVPEPIALNDLESPEPPGEPVVTPEPVVEPEVATPEVIEPPEPIPEPEP
LPEPIPEPEPEPEIDVEAILEQQRLQEAWVAQQQQLAKIQEQERLEQERLENERREQERLEQERLEN
ARREQERLDNERRAREQAQKEAQVAAARRAAAQAAANAAKKAGNHGGGQPGQNQTV**
EGGINISNASWKTKPRVNNFCSARSDIDTTLQVSFRVDAEGKISNVNLNGSTGDAKLDRQIIRQIGRGR
LHPFREGNITRVGTAIYPITLKLQKDESCTN

PPIIPRED:

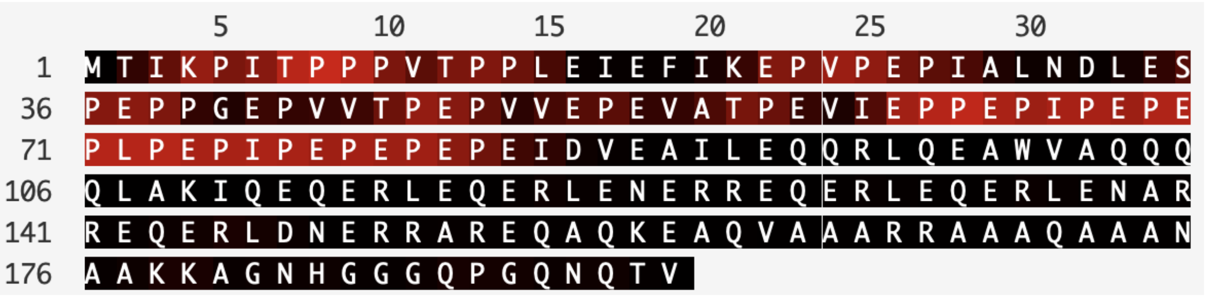

194 Domain II residues

30 Proline residues

74 α-Helix residues

Pseudomonas aeruginosa

Locus:

>AAG04660.1 probable tonB-dependent receptor [Pseudomonas aeruginosa PAO1]

MNRVFLTPAAVALCGASSLSLAEPVSLADQVVTATRTAQTASQSLAAVSVIDREDIERSQARSVPELLRQVPGVSL
ANNGGFGKNTTLFLRGTESDHVLVLIDGIKVGSASAGLTAFQDLPVELIERIEVVRGPRSSLYGSEAIGGVIQIFTRR
GDGQGAKPFFSAGYGTHQTLEGSAGVSGGAGNGWYSLGVSSFDTAGINTKRAGTAGYEPDRDGYRNLSGNLR
GGYRFDNGLELDGTLLRAKSHNDYDQVFGNSGFNANADGEQNLVGGRARFTPFDPWLVTLQAGRSEDKADAY
QDGRFYSRFDTRRDSLSWQNDLTLAEGHVLTLGYDWQKDEISSSEAFSVDSRLNKGWFAQYLGQYGRQDWQL
SLRRDDNQQFGVHDTGSAAWGYALSDALRFTVSYGTAFKAPTFNELYYPDYGNPDLDAETSRSLEVGLSGTHG
WGHWAVNAFRTNVDDLIGNDPRPAPGRPWGQPNNIDEARIRGVELVLGSQWLGWDWNANATFLDPQNRSGG
VNDGNELPRRARRMFNLELDRRFERLSLGASVHAEGRRYDDPANKVRLGGYATLDLRSEYRLNDEWRLQGRIA
NLFGADHETAYGYNQPGQAVYLSVRYQAL

tonB not near motor.

>AAG08916.1 TonB1 [Pseudomonas aeruginosa PAO1]

MSPQPSRSPDRFSLAALAEDHPTAPAQGDESESLPCVNAQRGEPNLRVVDCSGARRDEE
VAVEEVLIPYAHGSDPEDVPGEPPKSRWWLSSGAAVAMHVAIIGALVWVMPTPAELNLGH
GELPKTMQVNFVQLEKKAEPTPQPPAAAPEPTPPKIEEPKPEPPKPKPVEKPKPKPKPKPK
PVENAIPKAKPKPEPKPKPEPEPSTEASSQPSPSSAAPPPAPTVGQSTPGAQTAPSGSQGP
AGLPSGSLNDSDIKPLRMDPPVYPRMAQARGIEGRVKVLFTITSDGRIDDIQVLESVPSRMF
DREVRQAMAKWRFEPRVSGGKIVARQATKMFFFKIEKRR

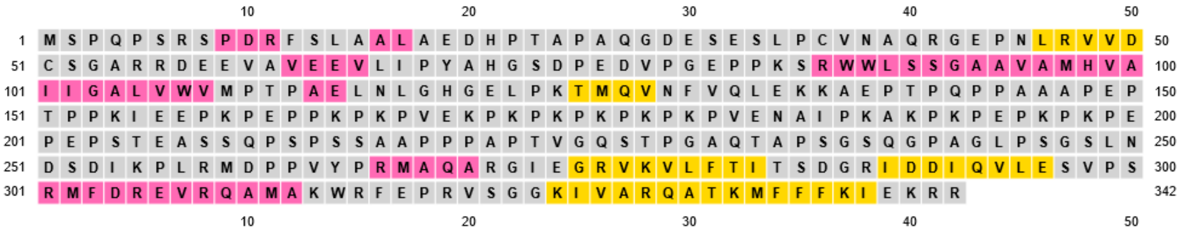

MSPQPSRSPDRFSLAALAEDHPTAPAQGDESESLPCVNAQRGEPNLRVVDCSGARRDEE
VAVEEVLIPYAHGSDPEDVPGEPPKSRWWLSSGAAVAMHVAIIGALVWV
**MPTPAELNLGHGELPKTMQVNFVQLEKKAEPTPQPPAAAPEPTPPKIEEPKPEPPKPKPV
EKPKPKPKPKPKPVENAIPKAKPKPEPKPKPEPEPSTEASSQPSPSSAAPPPAPTVGQST
PGAQTAPSGSQGPA**
GLPSGSLNDSDIKPLRMDPPVYPRMAQARGIEGRVKVLFTITSDGRIDDIQVLESVPSRMFD
REVRQAMAKWRFEPRVSGGKIVARQATKMFFFKIEKRR

PPIIPRED:

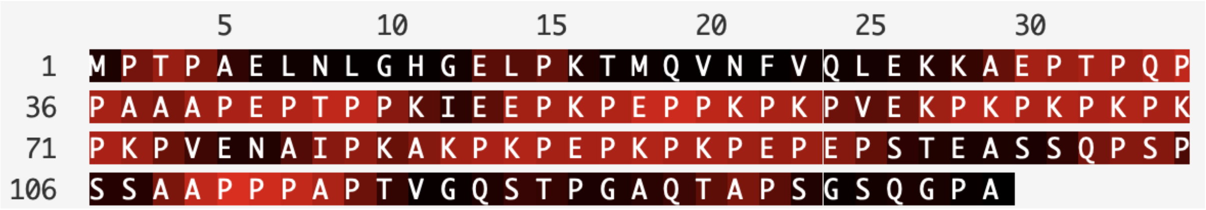

134 Domain II residues

40 Proline residues

0 α-Helix residues

Oceanspirallales

Marinomonas mediterranea

Locus:

>ADZ90088.1 TonB-dependent receptor [Marinomonas mediterranea MMB-1]

MKKILRIALLLYSDILLADDKSVLLDTLVVTGTKTEKTVADSPVAVNVVTKKDILASNATSVAEAIEALPGLRLKEIHGQGGDELVMQGMTGN
HILVLKDGAPIHNFGISGSNLSRININDVQRIEIVPGNASVLYGSSAMGGVVNIITNTPKEEWLSLTTTIKQNQDDKYTPSESVFQVDNGLKL
DNWTLASQWQFRNRQPYDTPPTDNSEDIAQLIAADVNEKLTYRHTKKTYSDLWFSLVREDTDREYDEQTPGSVLRETQQRNYRSQELG
YRYKGHSWDLSLKSNAAKLTSDTDNLKTSNYLDERRQTNSDNVLLQANKTFSTENHDITSGVSFSQIDIEQTLTETQLSATTAKTEVNDTIK
GTEFFIQDDWYVTDELELVSGIRTQWDSGFGQQTVPNIAVRWDVSPDLFIRSSAGLGYRVPNIKERYYRFDHSSSGYEVIGNPDLEPEVA
KSLNVELSYKDLTVQTFYKDITNLIDTSLDESIENGLDTYLYSNINSALIYGASIGISHKINKVQLSANYQWTKTKDQDTGLQLDHRPTHIASL
KLKRPVSLPHIKSSNVSLSARYTGEQFYDAKKNEKSQGYLLLNANLSMHFNDQWSVSFSGKNLSDVKNESGIDYDLRPMIGRQWM
LTLSYTPY

tonB next to motor with heterologous ExbD.

>ADZ89752.1 TonB family protein [Marinomonas mediterranea MMB-1]

MIKKGHWVLASTAALSLHAGAFYLAFYNPQQEGSVAPGTQGIEFGLGMVGDLGDSAETTVAKENISE
EPVVEETDVEEVEQPPEPQLEPEPEPEPEPEPEPEPEPEPEPEPEPIVASIEPEIIPEEVVVAKQESDILIPEP
EPEPEPEPEPEPEPEPEPEPEPEPEPEPEPEPEPEPEPEPEPEPEPEPEPEPEPEPEPEPEPEPEPEPEPEPE
PEPEPEPEPEPIQKPVVEAVITSNEPVDVPATPVPATSAPSTEGSSSVATNSNSESLKASSGSGVSSR
SGGSPGAIATYKSTLMAILAKNKKYPRASQRRGETGVVKVAFSLDANGELVSASVRESSGYRRLDKA
ALKAVKRSRFPAPPSDLSKNELSFTIPILFEQK

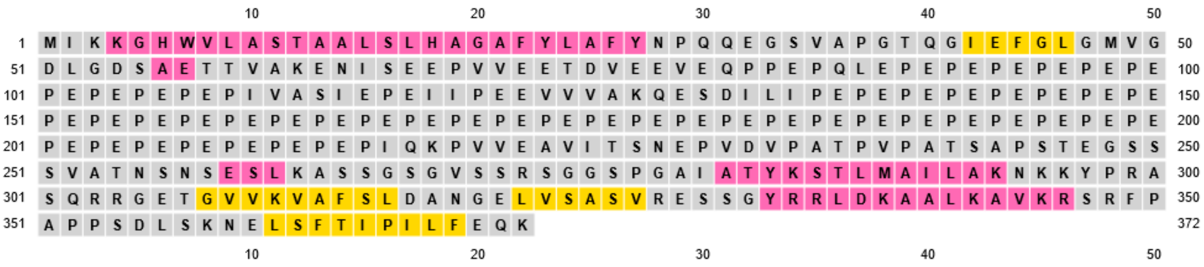

MIKKGHWVLASTAALSLHAGAFYLAFY
**NPQQEGSVAPGTQGIEFGLGMVGDLGDSAETTVAKENISEEPVVEETDVEEVEQPPEPQLEPEPEP
EPEPEPEPEPEPEPEPIVASIEPEIIPEEVVVAKQESDILIPEPEPEPEPEPEPEPEPEPEPEPEPEPEP
EPEPEPEPEPEPEPEPEPEPEPEPEPEPEPEPEPEPEPEPEPEPEPEPEPEPEPIQKPVVEAVITSNEPV
DVPATPVPATSAPSTEGSSSVATNSNSESLKASSGSGVSSR**
SGGSPGAIATYKSTLMAILAKNKKYPRASQRRGETGVVKVAFSLDANGELVSASVRESSGYRRLDKA
ALKAVKRSRFPAPPSDLSKNELSFTIPILFEQK

PPIIPRED:

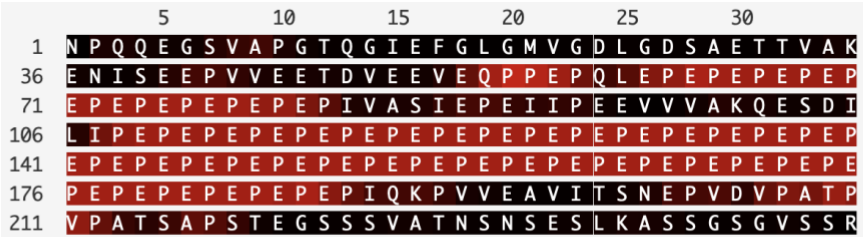

245 Domain II residues

66 Proline residues

5 α-Helix residues

Hahella chejuensis

Locus:

>ABC27848.1 Outer membrane cobalamin receptor protein [Hahella chejuensis KCTC 2396]

MRLVTLLLLVGSQLTGARAEELGPVVVEAKAPSADEIDAEYQTGFVKVIKREQFDSKVATVADVLKNE
TGVQVRQSGGLGSYSAVYLRGSTSKQVNVYLDGVLLNDATGGAVDLSQILLSGIEQIEIYKGATPIQLG
YSGVGGAINIKSLRFSKPIKQLALGYGSFNSRKGALTFADSVGETNYLASLDYLGSDNDFEMLNNNQT
EFNPYDDRVERRRNADFSQFNGMLTAERSLSDRTDLQLMAQHFNKDQHLPDIANLESTRTSLDTDFT
RLQSKLSFARNARENYTGRIFWSTQEERYDDSQSRVGLGVQKTRANTDVVGAELYGSYSLGLHLFS
ATLEARKETYQEEDLTRRSQKQDFERMTYLLGVQDEWADAEDIWLVRLSARQYFLEDETPAASMSG
RVETASDSAQYHSLQAGVRYRLTDWLSLKTNASRDVRFPQLAEKFGDRGFFIGNSELQPETALNGDI
GFEVGVDSFSVSAAYFYRDLKDAIVPSYDSRGVGRFENVGKARVSGVEVDAMYRPVPAWTFIARST
AQDTENLSDARDLHGKQLAGAYTYSHFLSAAWSIGRITTSVEYRHESGAYYDSAQETEVEDQNIVDV
VGRWSDGETTVEVSANNVTDEVVEAFNGFPSPGRHFFVSLMHLF

tonB next to motor.

>ABC33592.1 Periplasmic protein TonB, links inner and outer membranes [Hahella chejuensis KCTC 2396]

MKRHIIGITVALCLHISLAAWLTSGVSTPAPAPIAAAPVSLVLSQMRIVEQQVEAKQAEAEPAPEVEEVV
EAEQPVIEEPEPEVVQETPKPVETPPEPKPEVAKVEKPKEPKKPVKKQPEPKKIVEKVRKPQPTKKEE
VAKVDPEPNKKLAENPIPSPDPHTDISTAPAIAEQPQLSSAQIVSIEQAYINALQLALEKNKRYPLSAKR
RRQEGTSTVEFTIMKSGDIAEVRLLDSSGISRLDEAAVKALVTLAQFHPIPDELDRNHWKMSVPIRFQL
N

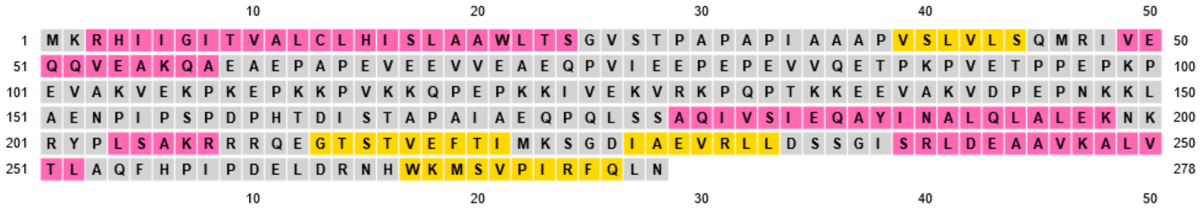

MKRHIIGITVALCLHISLAAWLTS
**GVSTPAPAPIAAAPVSLVLSQMRIVEQQVEAKQAEAEPAPEVEEVVEAEQPVIEEPEPEVVQETPKP
VETPPEPKPEVAKVEKPKEPKKPVKKQPEPKKIVEKVRKPQPTKKEEVAKVDPEPNKKLAENPIPS
PDPHTDISTAPAIAEQPQLSS**
AQIVSIEQAYINALQLALEKNKRYPLSAKRRRQEGTSTVEFTIMKSGDIAEVRLLDSSGISRLDEAAVKA
LVTLAQFHPIPDELDRNHWKMSVPIRFQLN

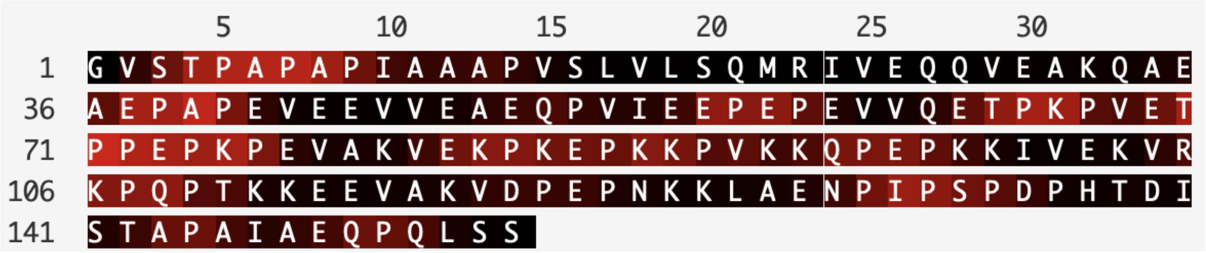

154 Domain II residues

30 Proline residues

10 α-Helix residues

Alcanivorax dieselolei

Locus:

>AFT72403.1 Putative vitamin B12 transporter BtuB, tonB-dependent receptor family [Alloalcanivorax dieselolei B5]

MSRFKLTPVAAAVIGLCATASHAGENPTDPILLAQLSLPEVEPHENRLDPVVVTPSMTTQTARASLTP
VAVIDEQTLREQQPLEMADILRAQPGVDIVGNGAFGKSTSVYMRGTGGSSTLLLLDGVRIRPATGGTP
SWQFLPPSMIDRVEVVRGPRGSLYGSDAVGGVVQVFTPDGHGDPGAWLELGGGSFNTRSIGGGAA
GEKDGTRYSMAVNRFDTDGTRLRRHGDDRGYDNTSGIVKLSHRFGERAEIGVMGFRAEGNTEFEP
GTTANREDTDYLIQVGALRGELLVSDDWLMQLQVSEARDENENFTDGDLTSTFNTKTRAVNLKNHFA
VNDYVELIVGGEYLRDDIESTTQYSLDNRENKAAFAQLMTTFGDLDAQASVRYDDNEAFGHKSTGGV
ALGYALNPHHRLRVSYGTAFRAPTFNELYYPGFGNPDLSPEKSENKEIGIRGQYGRGFWDLSVFQND
VEDLIASELRDGKYLPYNVNEARIRGAELQTGLDLDQWRLAMAITVLDPKDEETDELLQRRNRKSVRF
DVDRDFGSVSLGGSAVFQDHRYNDAAATQRLPGYGLLDLRASWRFAPDWTTRVSVKNVFDKEHAT
ARNYSGWNYLNAGRTVFVSVRYDIR

tonB not near motor.

>AFT68359.1 TonB family protein [Alloalcanivorax dieselolei B5]

MASAQVTAADRLGFTLFMALAVHGLVLFGVGFAPEQPRATPRTLDVTLATHSSDQAPDEADFIAQAN
QEGAGDEEEKQELTTTEQAPFTDAQPRPVQLEPDSAPPPRPVSRQKVIVTHSFSPRAEPRQQEQKQ
EPSEPQRHDDSLDQLSREIASLQARLDQQKQAYAKRPRVRRLTSVSTKAHYEALYIDAFRRKVEITGT
RHFPARALTSNTFGGVRLMVALGKSGEIRDIRVLQSSGHAFLDEAAVQSVRLSAPFEPFSTEMREHM
DVLEIIRTWQFDANRQVSSR

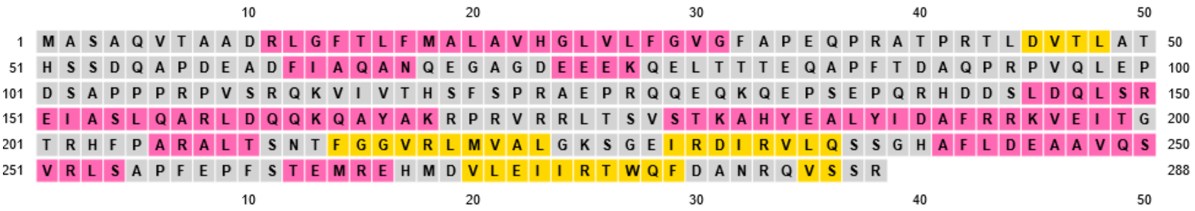

MASAQVTAADRLGFTLFMALAVHGLVLFGVG
FAPEQPRATPRTLDVTLATHSSDQAPDEADFIAQANQEGAGDEEEKQELTTTEQAPFTDAQPRPV QLEPDSAPPPRPVSRQKVIVTHSFSPRAEPRQQEQKQEPSEPQRHDDSLDQLSREIASLQARLDQ QKQAYAKRPRVRRLTSVSTKAHYEALY
IDAFRRKVEITGTRHFPARALTSNTFGGVRLMVALGKSGEIRDIRVLQSSGHAFLDEAAVQSVRLSAPF
EPFSTEMREHMDVLEIIRTWQFDANRQVSSR

PPIIPRED:

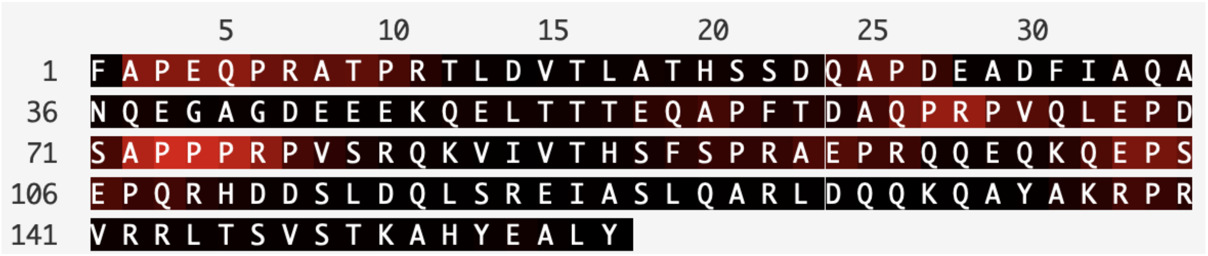

157 Domain II residues

17 Proline residues

44 α-Helix residues

Halomonas elongata

Locus:

>OBX37423.1 vitamin B12 transporter BtuB precursor [Halomonas elongata]

MTTSRYFRWGLASLCLGATPLANAAPNDTDADTEQRLDPVVVSATLAPRTASQSLSSVSVIDEATLR
RQDPTDITDVLRAQPGVDVSSNGSFGKNSSVYLRGAGSQSTPLMIDGIRLRSATAGGPSWEFLDPR
MFERVEVVRGPRGSLYGADAVGGVVQLFTPEGEGEPTPRITLGGGSFDTRRASASLSGSEDGTRYY
VAASRLESDGYEIVDGEGDKGYDNTTGLMSLSHTFESGAELGVLGLRARGNTEFDSFGTPSDTDYV
QQVAGVYGELPITDTWRSRLTLSEARDERETHGGTGESVFDTRTRTARWENTLALGRHELVAGAEY
ARDDVDSTVDYDEDSRDNTAVFTQALLDFSPLTVQAALRHDDNEAYGEETTGSLALGYELDDIHTLR
ASYGTAFRAPTFNDLYYPGFANPDLDPEESESVELGLRGQLGRGFWDLAFYQTDIDDLISNIDTDGD
GFVDTPENVERARIRGVELSTGAEVLDWSLRAALTYTDPEDRETGNQLARRAKKSLRFDADRELGD
WTLGGSVIAQGERYDDAENQQRLGGFATLDLRASWDFAPGWSTRLTLENLLDKQYQTVGGYDSAG
RAAFLSVSFGG

tonB next to heterologous motor with multiple ExbB, one with large periplasmic domain.

>OBX35796.1 transport protein TonB [Halomonas elongata]

MLRGPFSLVAGAALALVLFMLLALLVVPPEAEPEQRLETMTMNMAEAPTEEAEPNPAPAAN
APAPPPPAAATPPQPAPAPSISSDIALPEPELPPMEAPEVPLDESLPELQETPPEPQPEPEP
KPEPKPEPKPEPTPQPEPAPEPSPEPTSEPAAASTTAAPSESASAASAPAASQEPVDVGTS
AQATRRGKPTYPPRAQRRGIEGHVVVQFIIRPDGSVDPGSVEVLEANPPRVFERAARQSIV
EWQFQQADQRRRARQRFEFNLR

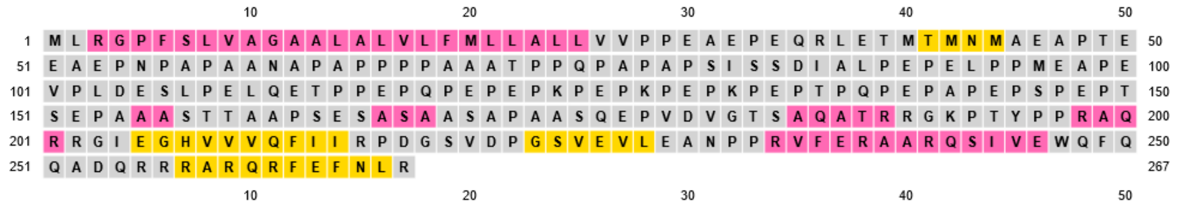

MLRGPFSLVAGAALALVLFMLLALL
**VVPPEAEPEQRLETMTMNMAEAPTEEAEPNPAPAANAPAPPPPAAATPPQPAPAPSISS
DIALPEPELPPMEAPEVPLDESLPELQETPPEPQPEPEPKPEPKPEPKPEPTPQPEPAPEP
SPEPTSEPAAASTTAAPSESASAAS**
APAASQEPVDVGTSAQATRRGKPTYPPRAQRRGIEGHVVVQFIIRPDGSVDPGSVEVLEAN
PPRVFERAARQSIVEWQFQQADQRRRARQRFEFNLR

PPIIPRED:

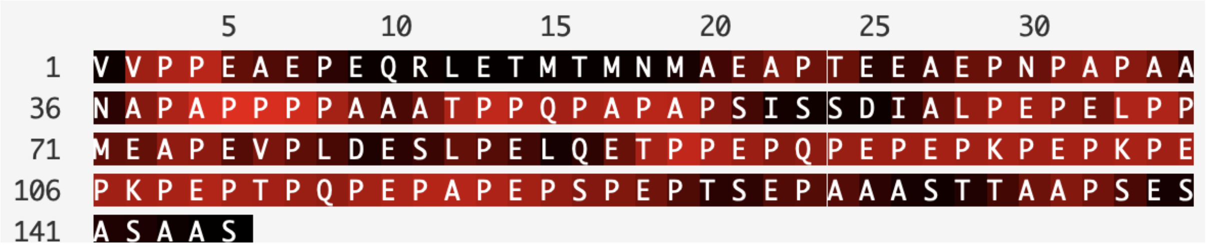

145 Domain II residues

45 Proline residues

4 α-Helix residues

Thiotrichales

Francisella tularensis

No *tonB* or TBDT genes detected.

Algorithm detected *tonB* and TBDT genes in other *Francisella* species.

Francisella sp. LA112445

Accession:

>QIW10982.1 TonB-dependent receptor [Francisella sp. LA112445]

MKKNILVKIMLSLSMFSCAYAQQNQQVNNGDTVDLGEIEVAAVKKADVGSVKKDKKKKESVEQSANAAPPLSTSKSKNSLILSTA
DQLNKLQGVQIFSGSREISQNISMGGLAFNRVFVGIDGINNNFASFGHNSTRQLPSESLYKQVTADQSGSNIVYGNGALGGAVN
FETIDPSDLLKGRSFATKLGVGGQTGDLGVNGNTAVAFKAGDFSYLFDIVGGRNNDIRLGNHTNLPYSASNNLQFLLKGVWNISK
IQTFKAYWLSMQNNGLYPATINDVVGEYNPPSKFNFLQQQVMLEYYLNPNNPYVNLEAKVFYIRNHYNSNPINDGKGFAVPQDI
NINTTGFRVTNKTFVLKQNLVYGTEFKFFNGFNVGKNSLSFPTANQQTYSGYLQDSWDITSKLNITLGGRVNGYHSQSGNMQN
GGAFFTKQGSISYKVIKPLKVYFAYTEGYRIPSLQDLFLGGSHPGMSFLQFVPNPNLQPEIGHNKSVGITINKQLTPTQSFYFNSNI
FLNDVSNFIVDSKVNAASYPLKIQNINISHAQLFGYSLAFKYHNTWFDFGTNFTYTRGESLSAYQGSGGSLVPSGSPLPIPRAKGI
ATLDIPIKPIDSSFQTSINYALTQNRVPPGGEHPISSVPGYVLLNLGYKWQPKRFKGLNVSAGVDNVFNRYYQNYDGQNLVPAMG
RNFYLQASYNF

tonB next to TBDT but not motor.

>QIW10981.1 TonB family protein [Francisella sp. LA112445]

MLRTLKFTVAVVGSIFVDYSIYATLNQSKSTEWYIGETQLKSQSIEEIVKKINIVSQPKTSSKE
VAAKQLKHATKAAKKTKNTKAKLTKNKPHKQEESKVHKKSANIDVKENKAIAKITQQPKLKY
TPPKLGYPSSAIKNNEQGHVRIKANINSKGQVIGVHILQSSGFDDLDQAAIKWFEKLVFTPAK
SGFTNVATTVEQTISFNLKEQQNA

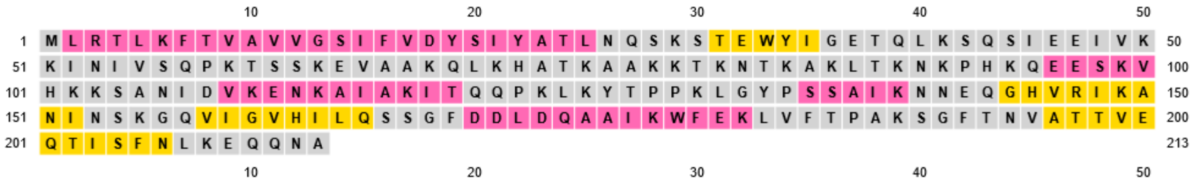

MLRTLKFTVAVVGSIFVDYSIYATL
**NQSKSTEWYIGETQLKSQSIEEIVKKINIVSQPKTSSKEVAAKQLKHATKAAKKTKNTKAK
LTKNKPHKQEESKVHKKSANIDVKENK**
AIAKITQQPKLKYTPPKLGYPSSAIKNNEQGHVRIKANINSKGQVIGVHILQSSGFDDLDQAAI
KWFEKLVFTPAKSGFTNVATTVEQTISFNLKEQQNA

PPIIPRED:

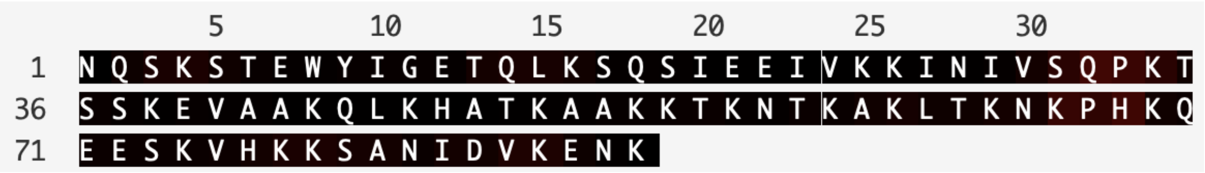

88 Domain II residues

0 Proline residues

10 α-Helix residues

Thiomicrospira crunogena

Locus:

>ABB41558.1 TonB-dependent receptor [Hydrogenovibrio crunogenus XCL-2]

MTFKIRPLSLTLGFCLGTSTVYATPQSLDKIVVSANKAEQKTPSVTANITIITADEIEQKHYQTLDDALKT
VPGLAIKKSGGAGQQTSIFVRGASSSDRQVLILQDGIELTDPSGFGASIANIQLSSVERIEIIKGPQSGT
WGANASAGVINIITKKGGKQASVNLKAGSFNTKQLSSTLGAGNDKVDFLVHFNQHTTDGFSVIRKYDE
SQAQFENDAFKQTDVGFKLGLTPVKNHRFETTVNKTSATADYDSGSNPDDATSNSAYDSTTRQLQY
VYSGQAVNTKLFVQDHHIERTLNDTSYGTSQYEASIEEYGGQATFDYRSTDQLTLSANSKNYRDIDKK
RTFYNTGVGINNTNTLTENLVLTEALRYDDFNRFKNATTGKIGIKNNFSTNVFISANYGTGYNVPSLYQ
LSTNNPEQLKPESIDGYDVTLGAYNATLTYFKSTTQDLITALGSWPSNYYANASGKTLNEGVEFAYQR
SIDSLEADVSFNATWLNARNKDNQVLAYRPEQTANLTLDYYGLPQTQIGLVTRYTGTTYSADNQKGA
QIGEYFVTDLTANYQVNRHVSLYGQVLNLLNQQYVEGVVDYPATNPVPNYVYSTGGIQAFVGVKGEL

tonB next to motor.

>ABB42216.1 TonB protein [Hydrogenovibrio crunogenus XCL-2]

MGKPGTLAFGITASIYSIIGFFLWHTFQSQQHSSQLAPIAVTTAMFVTQPEPKPEPKPEPKPE
PKPEPKPEPKPEPKPEPKPEPKPEPKPEPKPEPKPEPKPEPKPEPKPEPKPEPKPEPKPEPKPEP
KPEPKPEPKPEPKPEPKPEPKPEPKPEPKLDSQPVETPATKQPIVKKERRRPNPYSIAEQIN
AEALYINKLARHLATLAQNHYPKRAKRRHQQGEVTLQFTLHPDGHISNLSIVKPAHFESLNE
ATLNIIRHTMAYQYTPFPKEMRQRPKTIQVPIEYILR

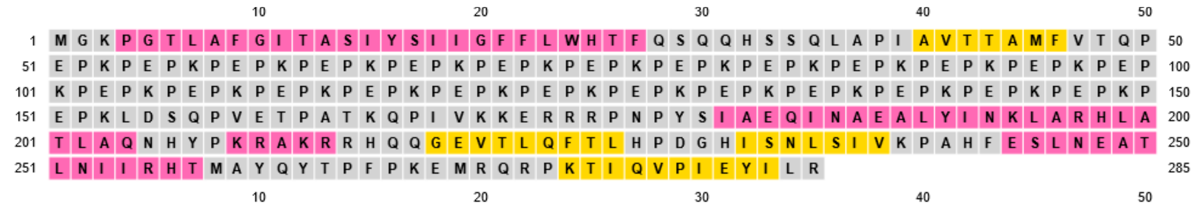

MGKPGTLAFGITASIYSIIGFFLWHTF
QSQQHSSQLAPIAVTTAMFVTQPEPKPEPKPEPKPEPKPEPKPEPKPEPKPEPKPEPKPE
PKPEPKPEPKPEPKPEPKPEPKPEPKPEPKPEPKPEPKPEPKPEPKPEPKPEPKPEPKPE
PKPEPKLDSQPVETPATKQPIVKKERRRPNPYSIAEQI
NAEALYINKLARHLATLAQNHYPKRAKRRHQQGEVTLQFTLHPDGHISNLSIVKPAHFESLN
EATLNIIRHTMAYQYTPFPKEMRQRPKTIQVPIEYILR

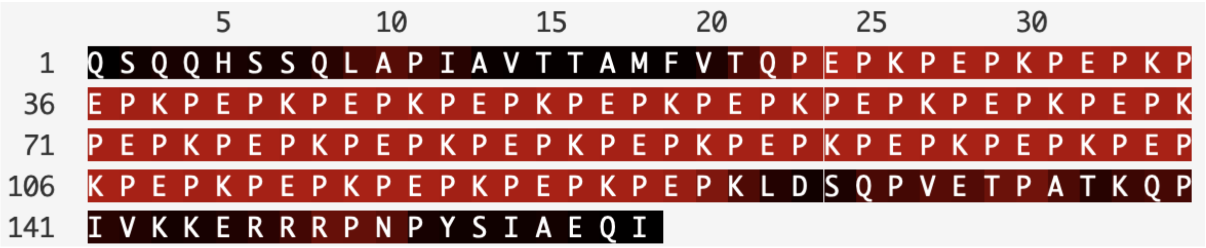

158 Domain II residues

58 Proline residues

5 α-Helix residues

Legionellales

Legionella longbeachae

Coxiella burnetii

No Legionellales found to have *tonB* or TBDT genes.

Methylococcales

Methylococcus capsulatus

Locus:

>AAU90376.1 putative TonB-dependent receptor [Methylococcus capsulatus str. Bath]

MNGAELVKHWSRFAIRCRKFMNTHSGSGLPATGVRRSRLRREAWVLAPFLFIFGSAGLRV
GSAEAAEDVVELDKITVYGEEDEVSPETEDLPEEQLEEKKAQTMDTASLFKDVPGVSLYTG
GGVSSLPAIHGMADDRVKILVNGMNITSACSNHMNPALSYVAPASVGKASIMAGVTPVSQG
GDSIGGTISVDPAAPSFAKAGQMAYTGKLGGMFRSNSDTFGNNFSGSIASEDFAFDYGGS
WAKGNNYMAGNGGPTVLPTRYLTTNMNLRASTKLDKGFFSADVTGQHIPYQGFPNQRMD
VAENDGLLGGFSYENDFDWGKLDARVYYHSTWHLMDMLSERKAGRMPMKADGSDLGYR
VRAHLPVDDVHTVRVGSEYFQQGLQDWWPGQGMHMPQDFLSINDGQRNRLGTFAEWEA
AWNKEWLTQLGVRNDMVWMNTGRVQGYDNGAFYSFWATPFNAANRSRVDHNFDATATT
AFTPDANSRYELGLSRKQRSPNLYERYAWAGPNSMITWFGDGNSYLGNLDLKPEAAYTAT
FTANWHDERQQVWSVSLTPYYTRVNDYIYGQKVAQYSNGFHGMQFVNLSHADMYGGDF
SARYAFLPESPAGSFAAKVVAAYVRGVGNDGQRSMGCPYVGTSLAGLCNNNQQFWPVN
GFLAPTSVNLYHMMPLNATVSLEHSIPAGSAVVDSAISVNLVDSKTAVAQSYAEPTTPGYVL
LNLRTSYQREHLRVDVGADNLLNKLYYHPLGGVDIVESIKAQDVVRLPAIGRSVYVSLNLEF

tonB next to motor and TBDT. Motor is heterologous, featuring ExbB with large cytoplasmic domain.

>AAU90377.1 TonB domain protein [Methylococcus capsulatus str. Bath]

MDFVRLPPRPAESGPPPSQTRKTVTESPAESSPSDARPDSAKPSTPSAPRLSRAPVVSRS
KPEPRMPGVAAPRLDIPARGTGVEFPAVAGTDSRLTAPPAQWNLRKMPAAPGDLASGAEG
AGGGGNPLIVLFRVVPEYPEVARRRGIEGWVRLEVRVTAAGLLGDARVVAASPRDTFDEAA
LEAIRHWRFKPAFREGRAVEQRAMLTMEFRLKRR

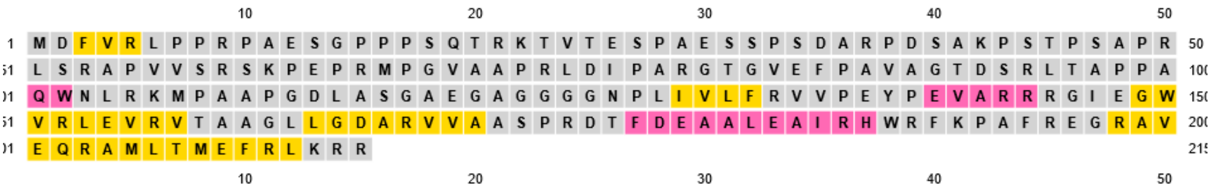

Broken. Selected the next *tonB*:

>AAU90375.1 TonB domain protein [Methylococcus capsulatus str. Bath]

MAAVPLVQTDSKSSGAESLYRPGSAILVVVSLAHLAVWWSYRQLKLLEPEERPSQPIEVSL
EMIAPAPKPAEPAAQPAPAPTAPPKPVTPPKPKPVAKPKPLTKTLPRPLPVQPERTLGDAAP
RSEPAAPPPEASSAAGGEGVPGGRSGSSVPASTPASFNANYLHNPLPEYPAFARKQRWQ
GRVTMKVHVLPSGIPAEVELQASSGHDILDESALETVRRWRFVPATKGGKAVASWVVVPLE
FSLTH

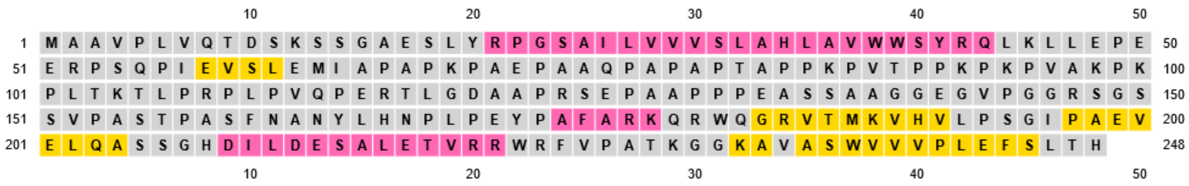

MAAVPLVQTDSKSSGAESLYRPGSAILVVVSLAHLAVWWSYRQ
**LKLLEPEERPSQPIEVSLEMIAPAPKPAEPAAQPAPAPTAPPKPVTPPKPKPVAKPKPLTK
TLPRPLPVQPERTLGDAAPRSEPAAPPPEASSAAGGEGVPGGRS**
GSSVPASTPASFNANYLHNPLPEYPAFARKQRWQGRVTMKVHVLPSGIPAEVELQASSGH
DILDESALETVRRWRFVPATKGGKAVASWVVVPLEFSLTH

PPIIPRED:

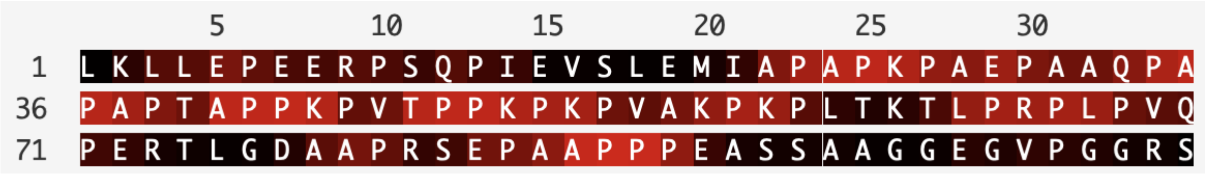

105 Domain II residues

29 Proline residues

0 α-helix residues

Chromatiales

Nitrosococcus oceani

Locus:

>ABA57061.1 TonB-dependent receptor [Nitrosococcus oceani ATCC 19707]

MLKRTPIFVLVATACAASPLLAQDEEVTLPELEVVGQPVERAGVEAENKPFATPDTADLLRRVPGANVNANGPIA
GMVQYRGMFGPRMNVRINGTPIESGGPNWMDPPLSYAPRSLVESIEVTRGVGSVSTGSGIGGYVEAKTKSSHFT
GSEQFEFHSDVDIAGHSVDGGYNLGGILSLSNDRHRLHFLGSRDDGGDTEFGDGTIKATEYERNTYGAGYGFKT
GDHEFSADYQRLDTENSGTPSLPMDISFIRTNRAGGKYTGFWNDIQLETRFGYSDVDHQMNNFRLRQAANFCAL
PAPFCQGDDKRLVNAGSEDFNYGLKATFALWGGDLAIGADGHAAQHNATVLDPDFAPFFVTNFNNAQINKYGFF
SEWTKEIYSNWELQLGARYDRVEMDADGVNAFPAQLADRGMGGMPAQMIQMLRNRFNGADRGQNNNNVDWV
AELSYQMNSALRWTFGAARKMRSPTYIQRYLWIPLEVNAGLGDGNAYVGNVALDPETSHELGFGFDWQTQAAY
FSPQAYYRRVDDYIQGTPAADPVVIAVSTNAAGDATPLQFSNVDAEIYGLDADFGYRFHPRWRLDGVASYVRGK
RRDINDNLYRIAPPNLRLSLTHERIHWFTTVESVLVYRQENISDTNTLDPGNANNTNEATPGYVLVNLYGQYRLLN
QGITFTAGINNLLDKTYTNHLTGFNRVLNSDVSQGRRLPGPGRNFYAALRYEF

tonB not near motor genes.

>ABA56895.1 TonB-like protein [Nitrosococcus oceani ATCC 19707]

MLSPSAMIPTMNYPLILTLLLSVLFHGILLLGMDQLKFFPPPTPIPLPLEIILVPFKQEKIPKKPN
VLAQAGKISKSEKEERHPLAPSAPSPLAAPPRVPTSPAIQPAPAVSKQKLPPPKQTALSPLLT
QKRATPSPSKPQQIPASTSPEIPETTSPRPSARELIAALEGQLAREIQRYANQPRKRYLDSN
TTEYAATAYLDAWRKKIERVGKMNYPEEAKRRGLSGSLILVVDLNPDGTVANIVVRHSSGH
QALDKAAIRIVRLAAPFANIPANVLQGHDILSITRTWQFHSGKDFSSR

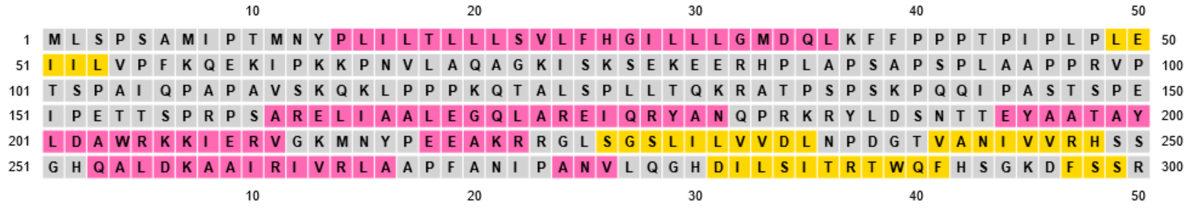

MLSPSAMIPTMNYPLILTLLLSVLFHGILLLGMDQL
**KFFPPPTPIPLPLEIILVPFKQEKIPKKPNVLAQAGKISKSEKEERHPLAPSAPSPLAAPPRV
PTSPAIQPAPAVSKQKLPPPKQTALSPLLTQKRATPSPSKPQQIPASTSPEIPETTSPRPSA
RELIAALEGQLAREIQRYANQPRKRYLDSNTTEYAATAY**
LDAWRKKIERVGKMNYPEEAKRRGLSGSLILVVDLNPDGTVANIVVRHSSGHQALDKAAIRI
VRLAAPFANIPANVLQGHDILSITRTWQFHSGKDFSSR

PPIIPRED:

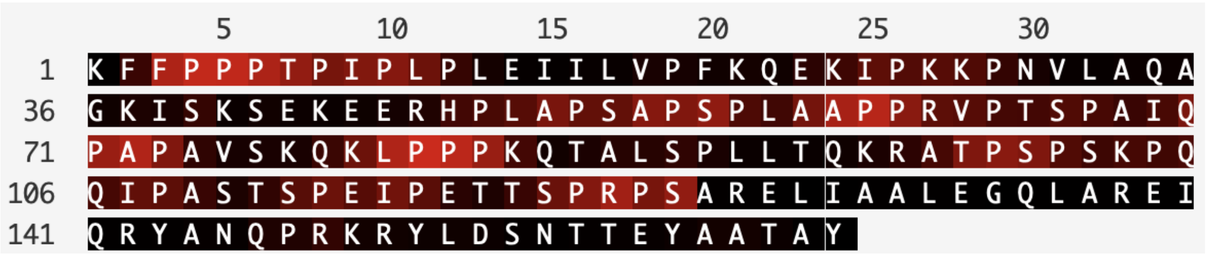

164 Domain II residues

32 Proline residues

28 α-Helix residues

Thioalkalivibrio nitratireducens

Locus:

>AGA32297.1 TonB-dependent receptor [Thioalkalivibrio nitratireducens DSM 14787]

MFAVLAAAMLGQSGPSAASDATLLPEITVTARGYEADTIATPQSVEILAPANAPSAAAPVGGLFRGEP
GLAVQSDGAWGQNPVLRGLKKESIVILVDGVRVNSAQPQGAIASFLDLGLLDRVEVVKGPTSVLYGS
GALGGAVNLITPEPVFSDAPAQSTRFSVGGSSADQALSGAALYRYSSPHHGLVIGGAARDADDYRSP
DGRVPRTGYSSDSLLARYAFAWSTETTLKLNLQRHEDRDVWYPGSARTGGQPGGAGIPPMLGTVTL
RSPRQTRELAEIGLDHEVLGGTLSGTIYRQEVYRQIRAWSDVLNRDYVRNDVTFATDGARLGYLRPV
GEIHLLNVGAEFWRMTGDPERYIDQPPAFEQDNTVRRSPFRNGEIETAGFFVQDEILLGATTLVAGLR
FDRTRGDADQKGFGPAPQTTGLGKTDNTLSWSLGAIHAFSDTLNPYVNLGSAYRAADMRERFEDSA
RGDGFFHVGNPQLDPERSTSLEIGLKGTSGVFDYRLAAFYTRIDDYIAGRITGENHPGSGLPIKRTENL
DRVVIRGLEGALAMPVGAFVADATFTWLRADNRQDDEPLAETPPPELRFGFGQPAERGFYWRAQTR
LVARQDRVATKFTNGGEDETSGFATADLNLGWNFGSAGALKQASLDLNLTNLFDRRYHEHLTQGVS
GQEILAPGRSIAAQFTGRF

tonB not near motor genes.

>AGA32018.1 TonB protein [Thioalkalivibrio nitratireducens DSM 14787]

MPDESSRLTLPLFLALVLHALVILGVGFEMLPEETPAQVTLDVTWVDAPAPAPEHAEHLAPQAQ
DASGAADQERMARTPEPVGERPADPAPAGDSGLIPEPIETQLEPFDPAPERPPDPVVTRAPEVP
PAPAPEEPSPTDTSQAPEQPSAALLMARGLEAARAMPAETEQTLLSRATRTRYLDTLAARGAPE
AAYLEAWIRKVERIGNLNYPDEARRRGLSGTLVLSVRLDAGGQVLDVAVAQSSGESILDQAAIRI
VELAQPFAPFTESMRESYDQLVITRTWAFRRDRVERVR

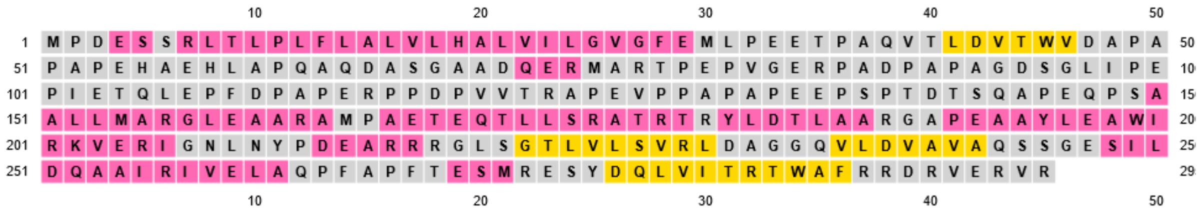

MPDESSRLTLPLFLALVLHALVILGVGFE
**MLPEETPAQVTLDVTWVDAPAPAPEHAEHLAPQAQDASGAADQERMARTPEPVGERPADPA
PAGDSGLIPEPIETQLEPFDPAPERPPDPVVTRAPEVPPAPAPEEPSPTDTSQAPEQPSAALLM
ARGLEAARAMPAETEQTLLSRATRTRYLDTLAARGAPEAAY**
LEAWIRKVERIGNLNYPDEARRRGLSGTLVLSVRLDAGGQVLDVAVAQSSGESILDQAAIRIVEL
AQPFAPFTESMRESYDQLVITRTWAFRRDRVERVR

PPIIPRED:

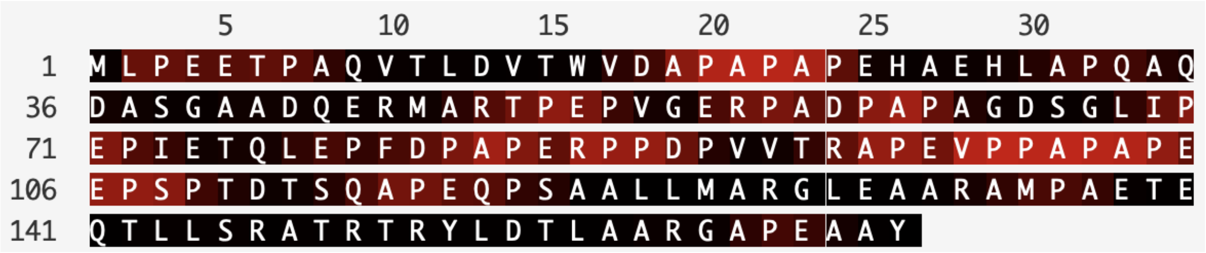

166 Domain II residues

30 Proline residues

43 α-Helix residues

Halothiobacillus neapolitanus

Locus:

>ACX95375.1 TonB-dependent receptor [Halothiobacillus neapolitanus c2]

MYKFPMSGRHRTGRTTLACAYLTACSFPLSALAGSPSTLPDLNITASHGAPVAFAGVPETVITRAEIERLQATSVLSLLENRAGIN
MVNQGGPGKITTASIWGMSGSQTLVLIDGVRIGSLTAGAAYLENLPVALIDHIDIIKGPRSGQYGADAMGGVIQIFTRKGKKGIQPS
FSIAGGSNKTFETSANVSGGNGPFDYSIGASHQGTGGFNAYTDTTGSPFAVNQPDNDGYRNNSASGRLNYTGDNGARLGVHW
LGTNAFTDYDGSSFAGNQQRSRQQNFGLDGALMKIGIWQLRASAGRNIERQTQYYDGKYVSEYNSRQDSFSLANDFSIGQGLL
TLGADRLNDHVSSSVNYDVTSRHNNGVFAQYATQIADLSIQTALRHDSNSQFGSANTGSVDLSWQLNPSLALTGGYGTGFHVP
SFNFLYYPGGYANPDLRPEHSRTARLGFNWQNNNGWTSSLTAFRTRTRDLIASSASTNFVPYNINQAQSIGAEWQLGWHNAD
WALNSSATWISAVDRETGVSIPRQPKWSGRVDVDRKLGAWRMGVTVRGQTATHESTFSQINNGFATADLRLSYAYSQNWHIE
AKLSNVLDKQYQTAYDYHQAGRTVLFTLRYGL

tonB not near motor genes.

>ACX95365.1 TonB family protein [Halothiobacillus neapolitanus c2]

MDHWALDQNFIDSDKRFGAALLVALVVHALFFAIYFTGDKPSPPPPRPIELTLDAPLAAKTND
ASPTPSDKINDKVTTQALASAAGEQTNTLQKPKSGKGITTTGNNGITDTPTTNAAPDAFNRL
NEAVSRSLKTGFLTSKTVNGPAGDYLSRWKRQVEAYGNDHYPEDLISQKISGQLILAVTIDK
EGHVMDIAIQHSSGNPKIDTAARRLVQLASPYPPFPPALAARFDQIVITRTWQFSSGHGLTT
R

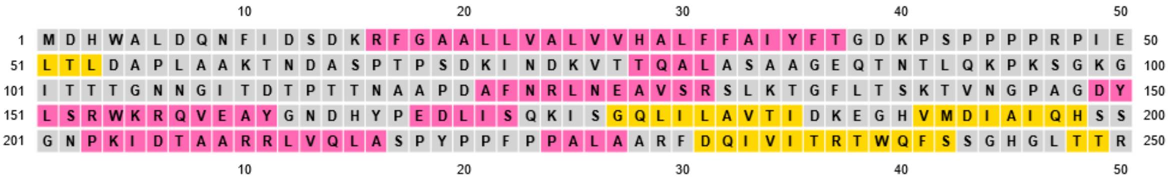

MDHWALDQNFIDSDKRFGAALLVALVVHALFFAIYFT
GDKPSPPPPRPIELTLDAPLAAKTNDASPTPSDKINDKVTTQALASAAGEQTNTLQKPKS
GKGITTTGNNGITDTPTTNAAPDAFNRLNEAVSRSLKTGFLTSKTVNGPAGDY
LSRWKRQVEAYGNDHYPEDLISQKISGQLILAVTIDKEGHVMDIAIQHSSGNPKIDTAARRLV
QLASPYPPFPPALAARFDQIVITRTWQFSSGHGLTTR

PPIIPRED:

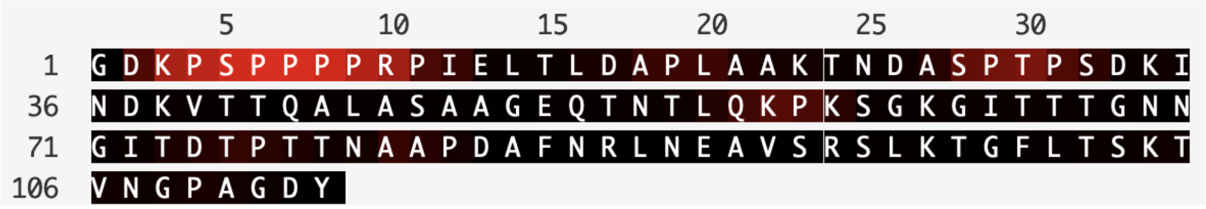

113 Domain II residues

13 Proline

17 α-Helix residues

Cardiobacteriales

Dichelobacter nodosus

PFAM search confirmed no *tonB* or TBDT genes.

Xanthomonadales

Pseudoxanthomonas spadix

Locus:

>AER54876.1 TonB-dependent receptor [Pseudoxanthomonas spadix BD-a59]

MLLAAGSLALPEAMAQQSTAGQQMQEEPASTPTELDTVIVTGQRQAQREAIQTKRETFAVSDVVASDDIGKLPD
HNTAAALQRVPGVAVWEDQGEPREPIIRGLTSTYNRTTIDGALVSSVSEEGRNVPLDIVPSVMADRIEVIKTVTPD
QDANAIGGIINIVTRSAFSTGQPFLDATGSLGFAEQHGDVRNDKQPYRVNFAAGRLFGADDQFGIVVSASDEQLD
YDIPQVESASPSVREYTAAGAPVDSGSALGNGIQVPTQRRLFWYNNTKNRSGLNAKLEWRPNPDFRWDTSAAY
NTMEDDEERIENRLEPIGNVANQTATSGDFASGRQFIQLNLPDTERSIWLGRSAVEWTAAERLKVSGDVIYSRAE
LDQTTTAIEFRTPTGTRARDFGFSYDTSDFYPIFTPYDQAAARDPANYRFQQYRDTQLDSIEESQQARLDLEYDN
GGLENFFKLKFGGVARFTDRELDQDRITYSTRSGFNWTLDSVSRAGPDKLIKGRYLLSPRIDYRAANDFLNANIGN
FNAALDNIGSDFTVSEDVYAGYGQATIKRGPFTVIAGMRYERTEVDTDSTRASDDVYLPVSDSGSYHNWLPGLHL
RYDATDNLVLRAAWTNTIGRPDFADITANSSINFDGSVAVLTRGNPSLKPRESEGFDLSLEYYLDEGLVALGLFRK
RIDNEIFTLTSNVEMDLGIGRGVEMVQVQQPMNAQSAKITGAEVAWQQTLSFLPAPFDGLGFNLNATFLDTEFAFI
TSAGLRETGLYMQPDVITNAALFYQYGPVEFRVSHNYLGGFLETINDTIPNADQYWKGRHTYDASVDWQLSDRL
TVFVQGQNLSDTGRREVTGPGKQYLQESADYGRTYWVGLSGHF

tonB next to motor genes, heterologous exbD, unusual gene order.

>AER54858.1 macromolecule import protein [Pseudoxanthomonas spadix BD-a59]

MAEQLVINRYREQEGPQGLNWSRIIGYAFVIALHVAVFLLLLIPAVAPPAQKEEEQKMLVQIV
DPPPPPPPHPPPPPKQETPPPPVKELSPPKPSPVPPPPEAPPVDVPDPRPMDTYAPPAPP
APPAPSPDIGASVDISSKNMNPPRYPPAAARAGVEGTVILIIDVDANGNVTNVSVEKSSRNR
DLDRAATEAARKWRFNPGKSGGQSVAGRVRVPVDFSMGG

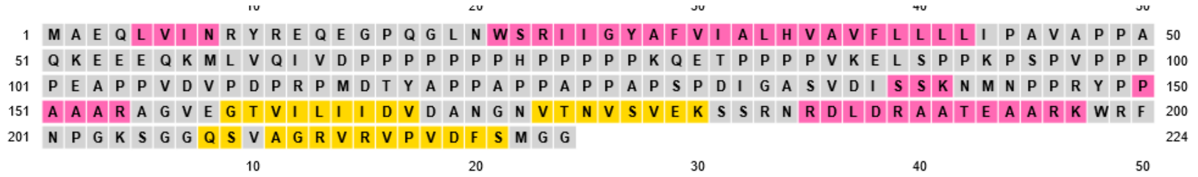

MAEQLVINRYREQEGPQGLNWSRIIGYAFVIALHVAVFLLLL
**IPAVAPPAQKEEEQKMLVQIVDPPPPPPPHPPPPPKQETPPPPVKELSPPKPSPVPPPPEA
PPVDVPDPRPMDTYAPPAPPA**
PPAPSPDIGASVDISSKNMNPPRYPPAAARAGVEGTVILIIDVDANGNVTNVSVEKSSRNRD
LDRAATEAARKWRFNPGKSGGQSVAGRVRVPVDFSMGG

PPIIPRED:

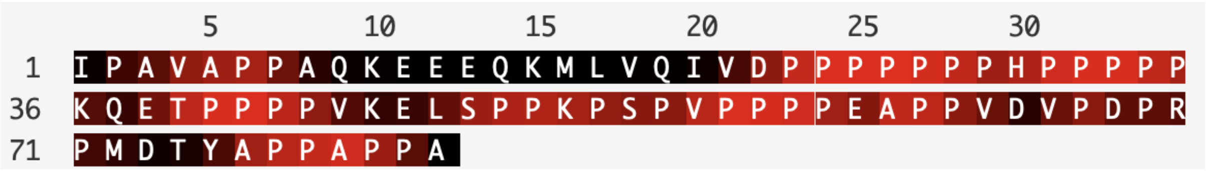

82 Domain II residues

36 Proline residues

0 α-Helix residues

Xanthomonas axonopodis

Locus:

>AAM34966.1 TonB-dependent receptor [Xanthomonas citri pv. citri str. 306]

MSRPPHPHALSLALGLALIAWLPWQSTAADMSGGADALTMDAVQVRNPFQSQNTRAIATKQAAPTIVDSVAADSI
GQLPDFNVGDALRRVTGVSTVEYQGEPRYVTVRGLNGNYNSMLIDGFAFASNDIGSRQALMDVLPANFVDRIDV
VKSLLPENDGGAIGGVTDLVTATGFARPDGLLTASAKGGANLMGSRYGGRTPVGEGELKWGKRFGRDGQFAFL
GAASIWRREISVPQQENGGALNWYNADGTRAPSAYGGTGAAVPSERRWYNYDNTRERRGVTARVDWQPDGP
LSGHVSGYTFNQHEASDRNAQVAQVQNSARLTRSGAQSGTLDNLNQLVELGQLRWKRGLSGINGELLAELPGQ
WRGALRASTSRATVDNPQTWDRFQQNRLAYGFDWNGTLPAFTALDPALADDPTRYANLNHQQERTTYAERVS
DLQLELRRNMDEDSRGLGLAFGLRQVRTHMQTAFQRTTWSGLPYSLADVLGDPTCALGCNAPMWTIDPDLADA
RWDAASGQARGVADITAQNSGTYTVDEQVRAGFAQAQWRGERWRLAGGVRLEQTRFGSSGQQLSGTVWAPV
SAQRTYRNWLPSLAGQVQTSANGTLRFGASRSIGRPRLDQMALNGGVLALGSNPPTLNQGNPDLQPRRSQNLD
LGHDWTFDDGGSLLSIALFHKTIDNEIFRYGQLQNIDGEQVLVTQPRNTDRPVRMRGAELGAIKELGPWLPALAG
LSVGANVTFLDVDYPVVLGDGSRTTLSVLPQQPKQLWNLTLNYARGPLRGTLAWSRTGELWDDRYPNYSNQQE
FYRNRYQQPLDRLDLKLAWDVSPAVAVSLDVLNLTGQGYEYRIGRHQEYVQSAWKMAPTVMLGVNVKL

tonB next to motor genes, heterologous *exbD*, unusual gene order.

>AAM34900.1 TonB protein [Xanthomonas citri pv. citri str. 306]

MTEQLVIHRHDYDAGNQGLSWARIIGIAFVIALHLTALMMLLIPAVAPKAPAEKERTTMVTLV
DAPPPPPPPPPPPPPEDKPPPPVKNLSPPKPSPVPPPPEAPVVDVPEPRPSDIVTPPSPPS
PPAPPSDIGASVDISSKNMNPPKYPPAAFRAGVQGEVILIVDVDASGNVTNVSVEKSSRNRD
LDRAAMDAARKWKFNASTVNGQKAAGRVRVPVNFALN

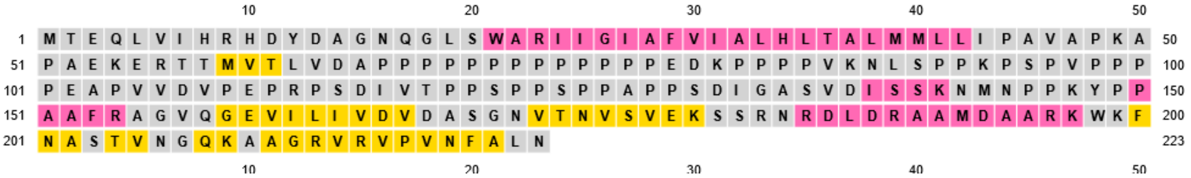

MTEQLVIHRHDYDAGNQGLSWARIIGIAFVIALHLTALMMLL
**IPAVAPKAPAEKERTTMVTLVDAPPPPPPPPPPPPPEDKPPPPVKNLSPPKPSPVPPPPEA
PVVDVPEPRPSDIVTPPSPP**
SPPAPPSDIGASVDISSKNMNPPKYPPAAFRAGVQGEVILIVDVDASGNVTNVSVEKSSRNR
DLDRAAMDAARKWKFNASTVNGQKAAGRVRVPVNFALN

PPIIPRED:

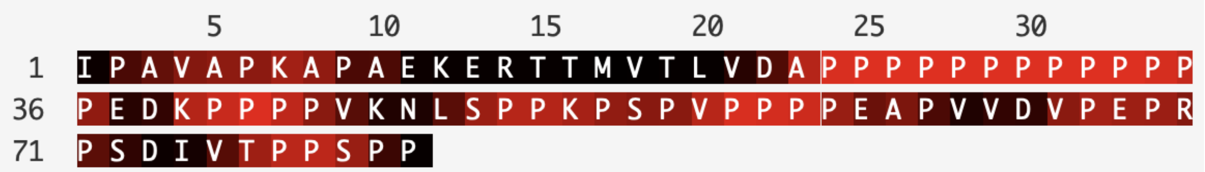

81 Domain II residues

36 Proline residues

0 α-Helix residues

Xylella fastidiosa

Locus:

>ACA12650.1 TonB-dependent receptor [Xylella fastidiosa M12]

MKTFSRYTPALNHLALALASTLLLIATYTANATAAESEHSTNEDSKAAPHDSKTTELDRVVITGSRIPRAGFDTLEP
ATVVNRQYIDSFGFTNVADALFSQPGFNSSASVRGNQAGFGAGVSFLGRFGLGSNRQLTLVNGRRLVTSNPPTV
FGPGDGGTQVDLNVIPTSLIERTETIGVGGAPTYGSDAISGVTNIILKKNYEGAEVNIGYGLTERGDNARYSYSALL
GSNFANSRGNVTIALDADDSAGVLGEARRYYRNGYSMQTNPNAAAIAQFQPGRQYATDGRVNPTIPFNTSNTDG
IPGRVLIRNRRINNMTWGGLLYPSTGRYIRDASQQPRGFGPNQDQFLQFDKHGNLVSYNPGVNFGNSSSSGGD
GLDLNQTTQVTSQLDRKSVFMLGNYDVTDNISSFFEGSYYTSKALELTDQSIYNAASFGTGNVNGSGDQSGALN
FNIDNPFLSEQNRQALRNLGITDFRLSRASRDLVIGNSSTDTRLWRAVAGLNGHFDAGGHPFTWETSLNHGEGN
FDYYRTGLIQQHFINAINVTRNSAGEIVCDSSAAGTTVDPNCVPLNLFGEGVASAAARNYVTTPTHAKAQMKQTV
FNANLSGSFIDLPGGKLGFNVGYERRREEGRFTPDEYQRLGLGRTVPTTGSHGTFTTNEYFGEILAPLVNPDAHL
PGLHRLDLTAKLRRVNNTVNGWFTAYTYGIQYEPFQGLQLRGNKTHSFRAPAIVELYTSQQPRYYSIPEPCTPANI
TAGNRPELRQRNCEAFFAYYNGVNPGTFQEAPTTQLGSVSGNPTLENELAKSWTAGIVFQPEWAHGLRVAADW
YNIKISNVITQLDSGDITSGCFDNETFNRNDVPNANRFCSMITRDPSTGVASSVRGEYTNGPYINFRGWTAEINYR
FDLKELRWGGSGILDLGFYGYFPKNLKYTAAQDIPAEESVGTIDNSKRQFQWNARYLTGPWNFGVSANYKSAAL
YSLTNTVETRDYLGIPSYTSYDANIGYTFDKHSSLNLAILNATDKMVAFPYVYDGLGRRYMLTFNYKFH

tonB next to motor with two copies of *exbD*, unusual order.

>ACA11062.1 TonB protein [Xylella fastidiosa M12]

MSEELIVHRYEQEDVSDALNWPRILGIAFVIALHLAALMLLLIPAASLKAPPEKQRLAAVTIVD
SPPPPPPPPPPQEEEKKPAPPIKTLAPPRPAPVPPPPQPPVVDVPQPRPSDVVVPPAPPAPP
TPPSDISASVDISSKKMNPPKYPPTALRAGIQGEVVLIIDVDADGNVTNVSVEKSSRNRDLDR
AAIDAASKWKFNPSVINGKKSAGRVRVPVNFSL

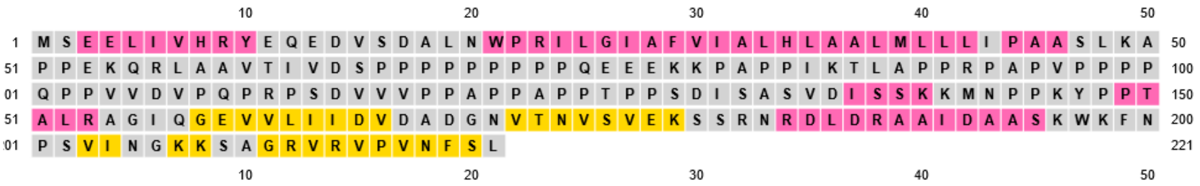

MSEELIVHRYEQEDVSDALNWPRILGIAFVIALHLAALMLLL
IPAASLKAPPEKQRLAAVTIVDSPPPPPPPPPPQEEEKKPAPPIKTLAPPRPAPVPPPPQPP VVDVPQPRPSDVVVPPAP
PAPPTPPSDISASVDISSKKMNPPKYPPTALRAGIQGEVVLIIDVDADGNVTNVSVEKSSRNR
DLDRAAIDAASKWKFNPSVINGKKSAGRVRVPVNFSL

PPIIPRED:

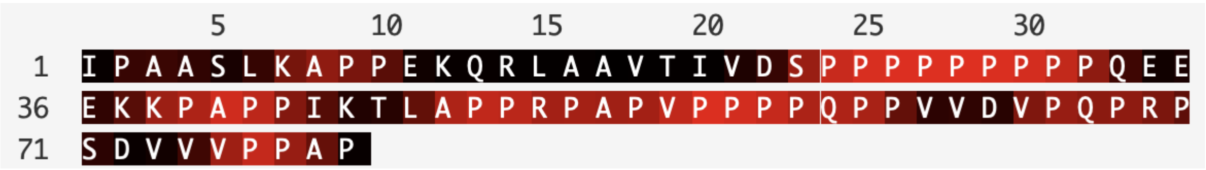

79 Domain II Residues

31 Proline residues

0 α-Helix residues

β-Proteobacteria

Neisseriales

Chromobacterium violaceum TolA

Locus:

>AAQ57756.1 probable TonB-dependent receptor [Chromobacterium violaceum ATCC 12472]

MRVKTLAQAIAAIGLLGSGFAHAADDNQLERVTITGSNIKRSIKQEKALPVTILKTEDLAKQGLTTVEQVVNSIAANQ
STQGSSSAVGASSGGGSFASLRGLGNQYTLVLLDGRRMANQAIDGTSADLNAIPLSVIDRVEVLLDGASAIYGTD
AIGGVMNFITKKSMKGFSIGGSFANPQHGGGDEKRVNASYGIGDLAKDGFNVYGAVDYLKQDMIMASQRGFAST
ITPQTDTVPSVNAWPGSYYDSVKKKYVQPSAPNCRPPYSQFVNGRCREYFPLYPSIVPEVEQISGVFKATKRIGD
DHELSLQYTRTETITTAQNAPLPTAGDVTRANLTDPSQRDQLYVRAVPLGNRETEANSVTQRLMLNLEGLVAGW
DYRAGIGRSENLIKENLTSGYISKSKMQSAIDSGALDPFDLSSNNLAAWRSVSMTGQTKEAKSTIDMADAKVSKEL
FDLPAGKLGVALGAEVRHESLSTIYNKSVTRDALSTGQDKTEDSMGSRSAYAFYGEADIPVLKTLDVQLAARYDH
YSDTESSLNPKVAFKFQPDPKVMFRGSASTGFRAPSLYNIYRPNQLQLTGKPFKDTGACVNGAPLPGTNGDACT
EMQRNKRIAGNKNLQPEKSSSLSFGMVIEPTKDFTASADLWWTMIKDQISVLDESLIINNPALYADRYVRDANGNL
SYLVDDYSNLGNLSASGVDLRLSWILPKTSFGVFTAGLDGTYLAKYEYQREKNGDYFSNLGTFKDGGPTFRWQH
NLTLNWMYGPWSAILSQNYKSGYTDENPKKENHLVKPYSTWNLSASYAWNKQLTFTAGVKNLFDQEPPFSNQQ
TRTTQMNYDPRLVDPTGRSFFLKGAYKM

tonB next to motor.

>AAQ58078.1 conserved hypothetical protein [Chromobacterium violaceum ATCC 12472]

MYGGRRCAGLCPQYADDRGDCKRAAPRRPATDWKMRIVCIIISDMKSSPVLQYSALGGVLAAH
ALALALLNGMAARPQTVTPPQALRMEMISLAPAEKPAVAPKAAPAPEPVKPQPQAKPAPSKPTV
QSKPALQPAKTQADAPAPAKAISAAAAPAAPTPAAEAPKSAGGKADSRDLPVTEPLAHGGYLNN
PAPAYPTVSREEGEEGTVRLRVHVSAQGLPQEVSVQSSSGFPRLDRAALAAVKRWRFIPAKRG
GEAIAYPFIVPIEFSLKSANT

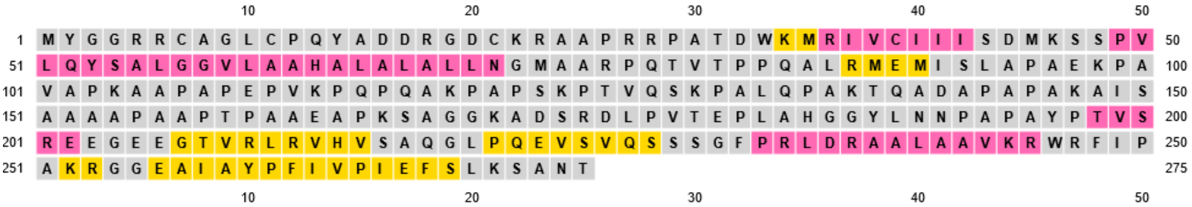

MYGGRRCAGLCPQYADDRGDCKRAAPRRPATDWKMRIVCIIISDMKSSPVLQYSALGGVLAAH
ALALALLN
**GMAARPQTVTPPQALRMEMISLAPAEKPAVAPKAAPAPEPVKPQPQAKPAPSKPTVQSKPAL
QPAKTQADAPAPAKAISAAAAPAAPTPAAEAPKSAGGKADSR**
DLPVTEPLAHGGYLNNPAPAYPTVSREEGEEGTVRLRVHVSAQGLPQEVSVQSSSGFPRLDRA
ALAAVKRWRFIPAKRGGEAIAYPFIVPIEFSLKSANT

PPIIPRED:

104 Domain II residues

22 Proline residues

0 α-Helix residues

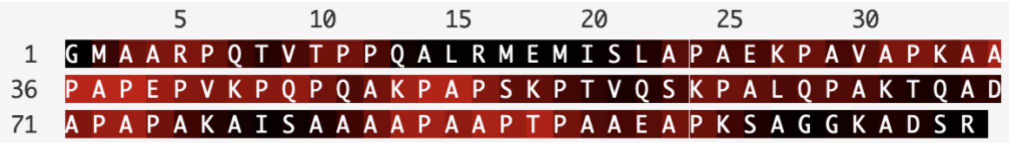

Neisseria meningitidis

Accession:

>AAF42164.1 TonB-dependent receptor [Neisseria meningitidis MC58]

MGQFMSVFRINMTAATVLAALSSSVFAAQTEGLETVHIKGQRSYNAIATEKNGDYSSFAATVGTKIPASLREIPQS
VSIITNQQVKDRNVDTFDQLARKTPGLRVLSNDDGRSSVYARGYEYSEYNIDGLPAQMQSINGTLPNLFAFDRVE
VMRGPSGLFDSSGEMGGIVNLVRKRPTKAFQGHAAAGFGTHKQYKAEADVSGSLNSDGSVRGRVMAQTVGAS
PRPAEKNNRRETFYAAADWDINPDTVLGAGYLYQQRRLAPYNGLPADANNKLPSLPQHVFVGADWNKFKMHSH
DVFADLKHYFGNGGYGKVGMRYSDRKADSNYTFAGSKLNNTGQADVAGLGTDIKQKAFAVDASYSRPFALGNT
ANEFVIGADYNRLRSTNEQGRSTLSKSVALDGFRALPYNGILQNARAGNKGFNHSVTEENLDETGLYAKTVFRPL
EGLSLIAGGRVGHHKIESGDGKTLHKASKTKFTSYAGAVYDIDGSNSLYASASQLYTPQTSIGTDGKLLKPREGNQ
FEIGYKGSYMDDRLNTRVSFYRMKDKNAAAPLDSNNKKTRYAALGKRVMEGVETEISGAMTPKWQIHAGYSYLH
SQIKTASNSRDEGIFLLMPKHSANLWTTYQVTSGLTIGGGVNAMSGITSSAGIHAGGYATFDAMAAYRFTPKLKLQ
INADNIFNRHYYARVGSESTFNIPGSERSLTANLRYSF

tonB next to motor genes.

>AAF42075.1 TonB protein [Neisseria meningitidis MC58]

MDKERILTPAVVFSVALLHLAMVALLWQAHKLPVIESGNVIEFVDLGDFGGGDGAPEGAGAPAA
PEPQPVPEPPKPVEPPKPVLKPVVTKKADADIQQPKEEPKPEEKPKPEEKPKPEPKPEAKPVPK
PAEKPVEKPSEKPAEHPGNASAKADSEQGNGEDKGTGTKGDGTGRGEGSGKGSGGVKGEHG
EGAGSSKGNPLRANGSIPRPAYPTLSMENDEQGTVVLSVLVSPGGHVESVKIVKSSGFSRLDNA
ARKAAQNGHFQANAWTEFKVPVKFELNT

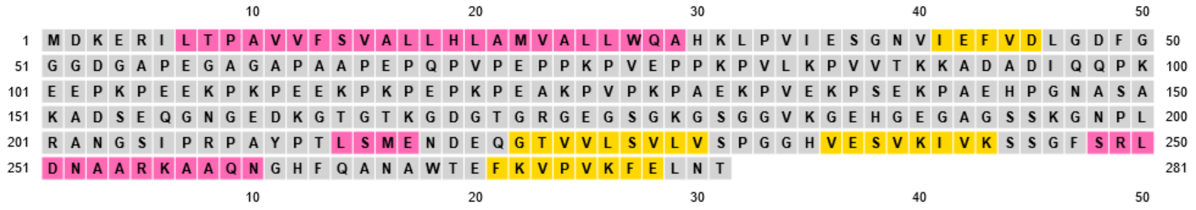

MDKERILTPAVVFSVALLHLAMVALLWQA
**HKLPVIESGNVIEFVDLGDFGGGDGAPEGAGAPAAPEPQPVPEPPKPVEPPKPVLKPVVTKKA
DADIQQPKEEPKPEEKPKPEEKPKPEPKPEAKPVPKPAEKPVEKPSEKPAEHPGNASAKADS
EQGNGEDKGTGTKGDGTGRGEGSGKGS**
GGVKGEHGEGAGSSKGNPLRANGSIPRPAYPTLSMENDEQGTVVLSVLVSPGGHVESVKIVKS
SGFSRLDNAARKAAQNGHFQANAWTEFKVPVKFELNT

PPIIPRED:

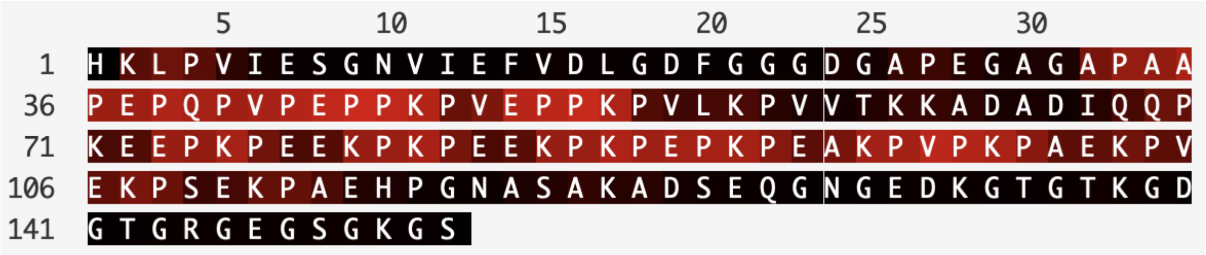

152 Domain II residues

30 Proline residues

0 α-Helix residues

Burkholderiales

Collimonas fungivorans

Locus:

>AMO93008.1 tonB dependent receptor family protein [Collimonas fungivorans]

MGKRARARARMYCAQKMVLTLGVALPFGAALAQTAGSTAPAATTEAPRSGSSLQLETMTVTAQRRVEKIKDVPI
SVTLLKGETLDVLNSGGQDIRVLAGKVPSLNIESSNGRTFPRFYIRGYGNTDFSTFASQPVSLIFDDVVQENAALK
GFPMFDLAGVEVLRGPQGTLFGRNTPAGVVKFDSAKPSLDKVSGYYSVSDGTHNTANVEAAVNVPLSDQWAMR
FSTLVQHRDNWVTNNGGGTPFLEGYDEQAGRLQFLYKPDGTFNALFNIHARATTGSARLFRANILEKGSDGFIAG
FDPSQITTNAINTQALHTAGGSARLSWNLDQVKLYSITGYETISSYFSRGDIDGGTPTGPGFIPFQVQTGGGVADH
KQFSQEFRVESKNEGPLNWQTGLYYFYEDVAGNSSNFDSTTQLQTSYLQNRQKNTAYAVFGSLTYDVNDRLKL
RGGLRYTEDKKDFSNEVANNVTFTGPGAISENKSNVSWDLSANYALTKDISTYARVATGFRAPSIAAPSSSVPITV
ANAETITSYEAGIKADLFNRRARANFSVYSFDVKNQQLTAVGGNSNSIMLVNAAKTVGRGAELDLEANLSERLRV
TLGGSYNYTQIRDPNLLVAGCASCTMLNTQVRPGLFSIDGNPLPQAPKWIANVTARYGIPMGNNDELFFYTDWAY
RSKINFFLYNSTEFTGKPLLEGGLRIGYKWDGDKYEVAAFARNITNKIVAVGGIDFDNRTAFINDPRMFGVQFRSN
F

tonB next to motor genes.

>AMO93146.1 tonB family C-terminal domain protein [Collimonas fungivorans]

MSGASPLPEPETSLRWPLALLIAGAVELTLIFLVLGTHAKPVAALLPAPVKIARLVTIADVPGE
PDPAPTAPPQKLESPKPAIKPLPQPSPKPQADAAPVESKPELQVDSTQQEAPPAALPVVQR
TQASIPAKADKPGTVRRGIVPLVRVEPDYPARALAANTEGVVVAHVTIEKDGSVSSVNIVRA
QPPKIFDPEAIRALMRWKFSQNDGGTVGEVELHFTLN

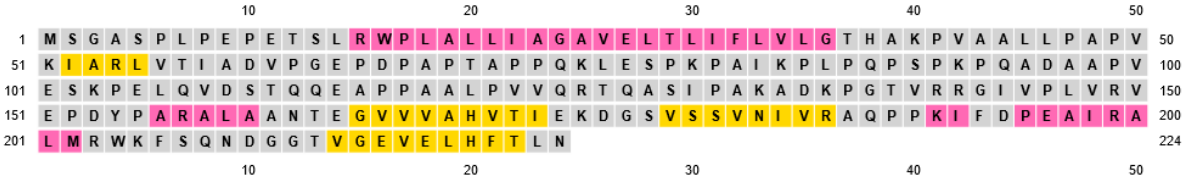

MSGASPLPEPETSLRWPLALLIAGAVELTLIFLVLG
**THAKPVAALLPAPVKIARLVTIADVPGEPDPAPTAPPQKLESPKPAIKPLPQPSPKPQADA
APVESKPELQVDSTQQEAPPAALPVVQ**
RTQASIPAKADKPGTVRRGIVPLVRVEPDYPARALAANTEGVVVAHVTIEKDGSVSSVNIVR
AQPPKIFDPEAIRALMRWKFSQNDGGTVGEVELHFTLN

PPIIPRED:

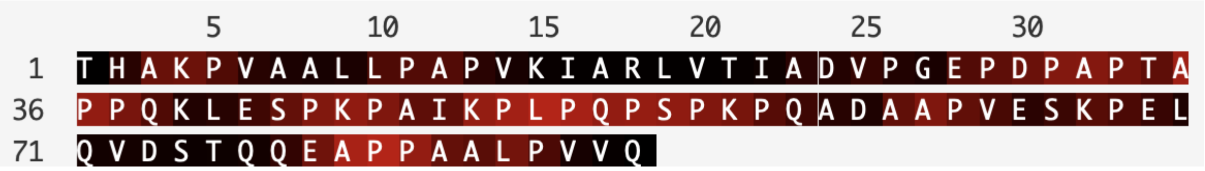

88 Domain II residues

21 Proline residues

0 α-Helix residues

Ralstonia solanacearum

Locus:

>CAD16107.1 probable tonb-dependent receptor protein [Ralstonia pseudosolanacearum GMI1000]

MGALAGALAGVAAGPVWAQGDVQSAGMPVGELNPTVVTASRSEQKLADALPHTTVISRADIERSQAPDAVSLLR
REAGIEIAQAGGPGAAASLFMRGAGSNQTLILIDGVRVSSGTTGTTQIEQLMADQIDHIEIVRGNVSALYGSDAIGG
VVQIFTRNGRGHAPLANAEIEYGARNTKRAQAGISGQLDARGDTSFALSVSALKTTGFSSINPKQAPNANPNDNA
YANKSVSAQLQHRFSSDWQAGLTWFQTWSTVSYDNAFGAPTDDNESHNQVRAMSAYVDGKLTPDWKTRLTLS
QGDDKNLNFTNGQVQEPGRFNTRNRQASWQNDWAFLPDQMLKVGFEHLDQTIDTDAYSPPTRRVDSGYIGYE
GKFGRHQLQLNLRRDRYSDFGGASSYYAGYGFAFNPQWKAVASISNAFRAPSFNELYYPFFGNPNLQPEKARS
VEGGVEYQSTVGLVRMTAFETDYSNLITSVFDPVSGNFLAANVNRARVNGLETSWRGTLHGVDVRASLTIQNPQ
DLSANRLLARHARHFGSVSAYKSFGPFSAGLEWNAAGERQDSARTLGGYGLLNLTGRYQITHDWALSARVENLL
NKNYQLIAPYNTASRGVFFTLSWQQHAPQR

tonB next to motor genes.

>CAD15665.1 probable tonb transmembrane protein [Ralstonia pseudosolanacearum GMI1000]

MIHPRTLKILVVVLLMHVGVLTLIQLGLIASPLRQEPPPELQVNIIPDTPPQPVRQPEPPKPEP
AKPAPPKQVNIVKPVPQPKPAPTPAPALPPSDTAPVVPAAPPAPPAPPVEAAPAPAPAPVQ
SGPINVGINDIQCSNPPLVYPTMSQKMGEEGRTLVKITIGPEGDVVNALVATTSGSARLDRA
ALESARSLHCSPYKQNGRAMTVVANKPFVFKLDN

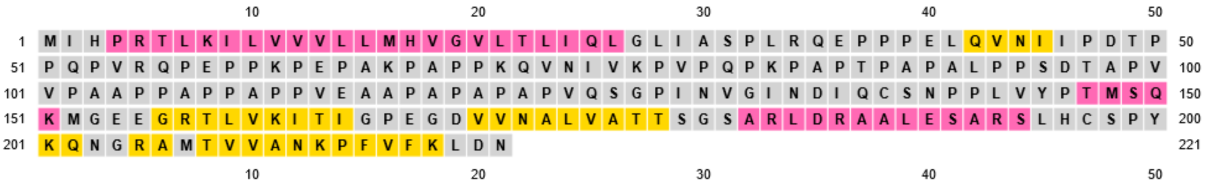

MIHPRTLKILVVVLLMHVGVLTLIQLGL
**IASPLRQEPPPELQVNIIPDTPPQPVRQPEPPKPEPAKPAPPKQVNIVKPVPQPKPAPTPA
PALPPSDTAPVVPAAPPAPPAPPVEAAPAPAP**
APVQSGPINVGINDIQCSNPPLVYPTMSQKMGEEGRTLVKITIGPEGDVVNALVATTSGSAR
LDRAALESARSLHCSPYKQNGRAMTVVANKPFVFKLDN

PPIIPRED:

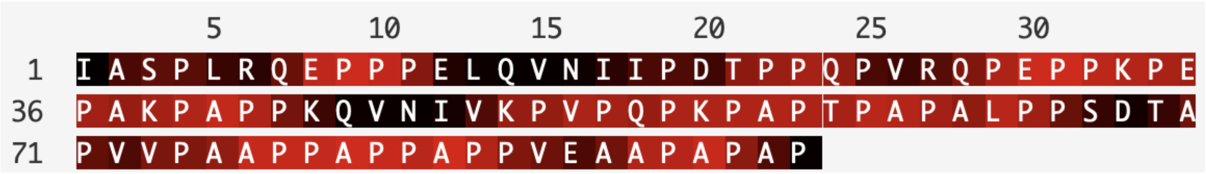

93 Domain II residues

36 Proline residues

0 α-Helix residues

Bordetella pertussis

Locus:

>CAE43854.1 putative outer membrane protein [Bordetella pertussis Tohama I]

MKSRSLRRCAGVLACVAPLAGHAQAGAAAGQPIPELDPVVVTAARSPQLLKNVLADASVIERDTLARAGQSSLAE
VLAQQHGIEFADSGGPQSVTSLFMRGANSNQTLVLLNGQRINNANGGGIALNALPPEAIERIEIMRGAASSLYGAD
AIGGVINIITREPGDKALSAYANAGYGTYGTSRYDAGVSGAADGFSYSLSTGYGQSHGFNATNRRSFSYNPDKDS
YYQNYATGTLGYEWRPEQKVVAQVYRSRINGGYDASASYDYNDRYIQDLQAYSLASENRLTRYWKSTLRAGYV
EDKNDSRAEGMFEDNNTRFRTRQMQYLWQNDFTLAAGQTLTLAYEHLDQRADGQMSTATGIGNYTETRRHVN
SYTGVYLGDFGRHHVQASLRNDNNSQFGSHTTGGLAYGFDLTPNLRATVAANTGFRAPSFNDLYTPTSAFGYR
GNPDLKPEESRNAEIGLKYQDEDSELGVVYYQTRIKNLIQVTEDFSTVENVGRARLQGFTISGAHRFGNTRLRASL
DLSNPRNEDTGKQLLRRARTVLRAGIDHRFDRLLVGAEWYASDERYDYGFPEEKRLGGYGLVNLTAAYDLSRNM
QVQVRWNNVLGQRYTLADGYNTAGSNAFVNPSWRM

tonB next to motor genes. ExbB predicted to have an extra helix either in periplasm or membrane.

>CAE43790.1 siderophore-mediated iron transport protein [Bordetella pertussis Tohama I]

MPSPQSGRSFSSHTSLALRAGAGFTVLALHAAVIGAVFLSKTERPELLEPEAVMVSIIEAPLPQLA
QAEPTPEPPTPVEETPPEPEPVIEPEPEPEPEPVVEPEPEPEPVVEKPPEPAPKPKPKPKPKPKP
KPQPKPEVPPEPKKIETPPAGAPQGAQQVTQAPRQAPPPDEPIMVSSVEYQGARPMPVYPMAS
KRLREEGRVVVLVEISTQGLVDRASIDTSSGFSRLDESALAAARKARFKPLTRNGVAYPAKAKLP
FDFVMRN

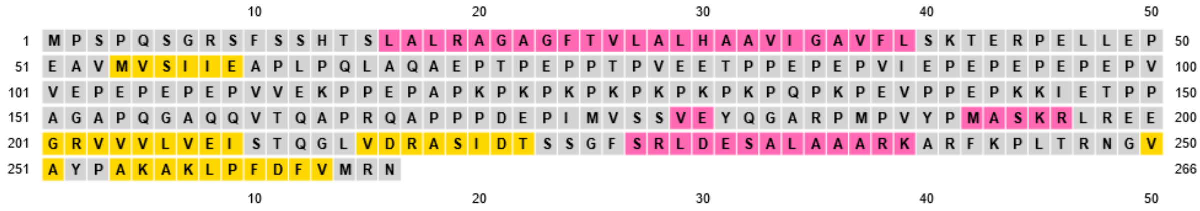

MPSPQSGRSFSSHTSLALRAGAGFTVLALHAAVIGAVFL
SKTERPELLEPEAVMVSIIEAPLPQLAQAEPTPEPPTPVEETPPEPEPVIEPEPEPEPEPVVEPE
PEPEPVVEKPPEPAPKPKPKPKPKPKPKPQPKPEVPPEPKKIETPPAGAPQGAQQVTQAPRQ
APPPDEPIMVSSVEYQGARPMPVYPMASKRLREEGRVVVLVEISTQGLVDRASIDTSSGFSRLD
ESALAAARKARFKPLTRNGVAYPAKAKLPFDFVMRN

PPIIPRED:

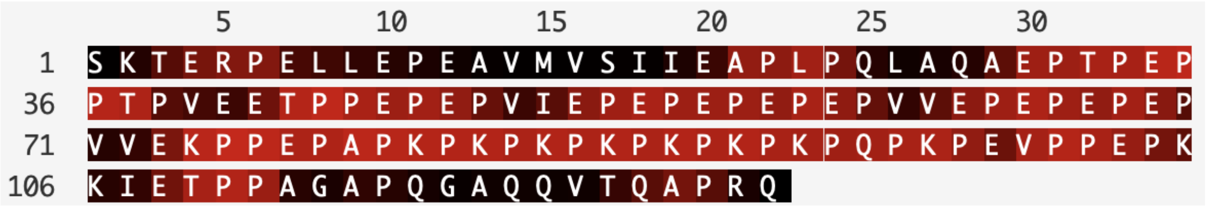

127 Domain II residues

42 Proline residues

0 α-Helix residues

Acidovorax avenae

Locus:

>ADX44735.1 TonB-dependent receptor [Paracidovorax avenae ATCC 19860]

MRDSMRSSPRGGLPPLALSLTLALAALSPAVHAQSDASAVVQSYDIPPGALGPALNRFAQEAGVSLAVDASRVAGATTAGLQG
RYGVEEGFAQLLQGSGYAIRKVGAGYGLAPALPAAAPAAGGRDRTGAQAAPAQTVTAGAAAPASAAAEAAPAVRTAEGRDTLE
TVTVSASRSNMEAEKAPQTVQVITRAQIEQQLSLSTNSSDVLSNLIPSYTPSRGKMNGSGETLRGRTPLILVDGVPQSNPIRPTG
REAHTIDFAMIDRVEVVQGANAINGLGATGGTINLITKRPENGALNQHVDMQATMPAGHGGSDSLSTKMAYSANGRQDRLDYLF
SIAAEDQGLWRDAQGRGIGADNTQGDLMDARSYDVLGKLGYWLDDDQRLQLSINRYRIKSQAHYIGVAGDRTRGIPTTSIEGTP
AGAPPFNEVQTSAITYNHYNLAGMELSALAFSQEFEALFGGDRSATFQDPLIAPRGTLVDQSRAKSSKLGTKVTLTKADLLDSRL
KLTGGFDTLVDKGKQDLFGTGRTYVPESEYRNLSLFLQGEYRLLDQVTLHGGVRRESADLKIDSYRTLAAYNRVAVQGGTLDFD
ETLYNAGIVFEPAKDWNVYASYSEGFGMPDVGRVLRSINTPGQSVDDMKSLSPIVTQSVELGTRVKRGAWDAEASWFRSSSDY
GTRVLRVNEAFMLAREKNRIDGLEASLGWQVNRAHKARLAYSRTKGRYDSNGDGHLDARLDGLNIAPDRLVASWTAQWNEQL
SSFVQVQRAFSRTFDNPAMNFSGYTLVDASVQYRLPKGRLRLAVANLFNRDYITYYSQSALVEPLRYFAGRGRTLTLGYSLDF

tonB not near motor genes.

>ADX44697.1 TonB family protein [Paracidovorax avenae ATCC 19860]

MRLPALLQRFSTLQIALAFSVAVHAVLLSARFIDPEGFNRVFQDTPLEVILVNAKSNERPEKA
QAIAQSSLAGGGDADKGRATSPLPYSAITQVGEDFEEAQRKLDAMQEQQAQMLAQLRKQL
ATMPPPDPRDQSPNTDRVQQEERRRQLLKLLAEIEKRINEENSRPKKRYISPATREEVYAVY
YDGLRRKIEDKGTESFPEQAGRKLYGELTMIVTVNHDGQVLDTEIVQGSGNRLLDQRAQAI
VRASGPFGTFSPAMRAKADQIAVVSRFKFTREQTLETSVR

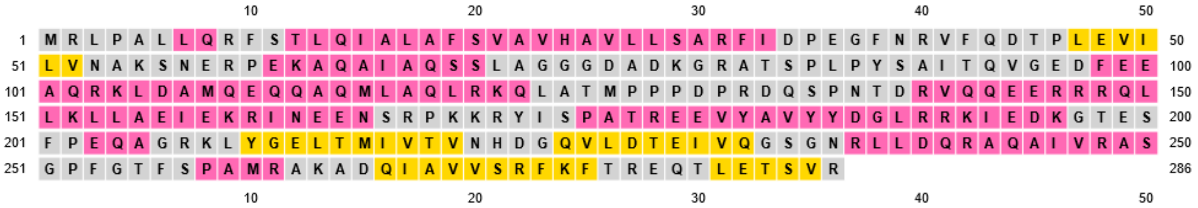

MRLPALLQRFSTLQIALAFSVAVHAVLLSARFI
**DPEGFNRVFQDTPLEVILVNAKSNERPEKAQAIAQSSLAGGGDADKGRATSPLPYSAITQ
VGEDFEEAQRKLDAMQEQQAQMLAQLRKQLATMPPPDPRDQSPNTDRVQQEERRRQL
LKLLAEIEKRINEENSRPKKRYISPATREEVYAVYY**
DGLRRKIEDKGTESFPEQAGRKLYGELTMIVTVNHDGQVLDTEIVQGSGNRLLDQRAQAIV
RASGPFGTFSPAMRAKADQIAVVSRFKFTREQTLETSVR

PPIIPRED:

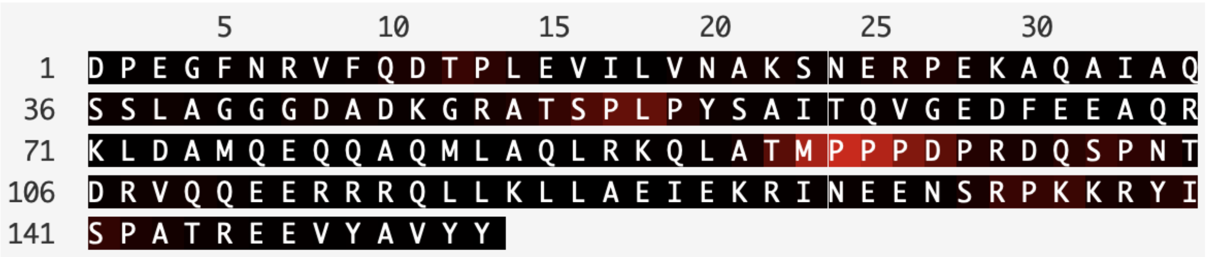

153 Domain II residues

12 Proline residues

73 α-Helix residues

Rhodocyclales

Aromatoleum aromaticum TolA

Locus:

>CAI07294.1 Hemin receptor precursor, TonB-dependent outer membrane uptake protein [Aromatoleum aromaticum EbN1]

MPTLRPSVVYAALACAFSSLSHADEAAHLSEVTVTAKGYASANVETPASVTAVEREEWLDRGADNVGEALRGQPGLAVASDGA
QGQNPVIRGLKKESVVLLVDGMRLNSAQPAGAIASFMSLGLAERIEVVRGPASVLYGTGALGGAINVLLPQARFEAGVQTRAGA
EFETANDGVRGTAVMNASGGDHALMLGASLARIGDYRASDGEVDHTDYDSDSFIAQYRFRLDDSQQLRLSLQQHEDRDVSYP
GSIKPHPHPQIRSSIIHSPSQTRELVEVGYSRRGSGDTPVNVDVRVYRQEMEREIAARAKGPFGRDFSDTEVTFATDGLDARAD
WLAHPDHLLSFGVNAWQMEASPERFLSSPPTFARGRNDPFDDGRIEAIGVFMQDDMRFGRLNVLAGLRRDWVEGDADSVNN
GAITRGLERSDSATSGSLGAIFEVSKLLRPYANVSRAFRAGEMRERFESSPRSDGYYYVGNPRIEPEIATQFEIGLKGADDDLSY
ALSAYRTRITDYITGRNVSGAPGTNRCPAANASACKETFNLGRATLVGVEAEARWQAVRGQWLTAAYSRVRGENEDLDEPLFQ
MPADEMSLGWEGRVAAAWTADATLRLVRKQDRVATVFAAGTENETSGFATADFGATWHGQRQRVRVALKNAFDKPYHEHLT
EGVSGMEIDSPGRSLMVSWQGQF

tonB not near motor genes.

>CAI07061.1 putative TonB protein [Aromatoleum aromaticum EbN1]

MSSRPPLRRSTPAASALRLLAKLTGGAGRDARRLQLALAVSVLLHALVLSISFTLPVRPPNRDPGLEV
VLVNARHARAPDKAEALAQANVDGGGNSDRQARPKSPLPPQEVHRDGNAPVEARKRVAPPAPLQR
SVLTQPKVASTISRPPPRTSDAPPALAPTPSGLDLLNSAATVARLEAEIGRQLDDYAKRPRKKFIGART
REYRFAQYVEDWRHKIERVGTLNYPEAARGRLYGSLLLVVAIRADGSIERVEIRRSSGEPVLDEAAKRI
VRLAAPFAPFPGDIRSDTDILEIARTWTFTNADKVTAQ

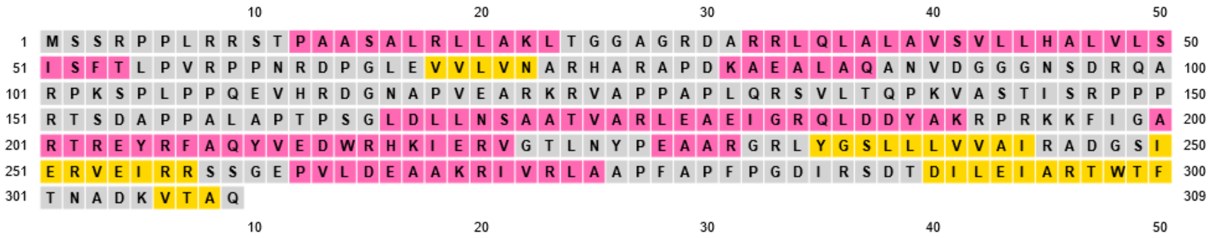

MSSRPPLRRSTPAASALRLLAKLTGGAGRDARRLQLALAVSVLLHALVLSISFT
**LPVRPPNRDPGLEVVLVNARHARAPDKAEALAQANVDGGGNSDRQARPKSPLPPQEVHRDGNAP
VEARKRVAPPAPLQRSVLTQPKVASTISRPPPRTSDAPPALAPTPSGLDLLNSAATVARLEAEIGRQ
LDDYAKRPRKKFIGARTREYRFAQ**
YVEDWRHKIERVGTLNYPEAARGRLYGSLLLVVAIRADGSIERVEIRRSSGEPVLDEAAKRIVRLAAPF
APFPGDIRSDTDILEIARTWTFTNADKVTAQ

PPIIPRED:

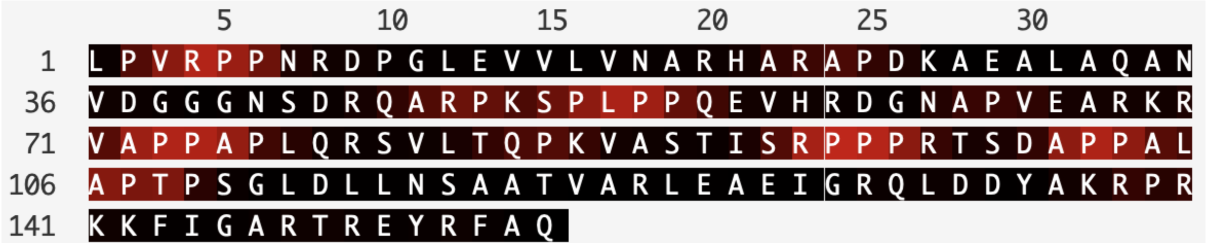

155 Domain II residues

22 Proline residues

43 α-Helix residues

Nitrosomonadales

Thiobacillus denitrificans

Locus:

>AAZ96668.1 TonB-dependent siderophore receptor [Thiobacillus denitrificans ATCC 25259]

MKFRLSALAGALSLAFSASPVNAQTPQENPPGELDTVTVWSEYAGSFKSDTVQVGTFREASPLDVP
QTSNVVTREVLDAQAASTLFGALRNTAGVTRSQLNGSTYDNIAIRGILVENRGNYRLNGSLPIINLIDVP
LENKERVEVLKGASSLYYGFVPPSGIVNLVTKRAGKDPVSSLALSANHYGGANVHADVGRRFGEEQ
QFGARVNLLAGREDIGVDNYSGDRSLGAVAFDWQASDALSFKLDVEHYRKDVSEQAAIKVLAPNAD
GTITLPAIPDARTNLAGEWQTYDADATNVLFRTDYVLSDDWGLLLEAGHAQTNRDRRFSQFENYNLA
TGDGTLKIFFSPNQEYENSNYRAESFGHIPGTWVSHDLSFGYTANQRDQNSRSGGSVSVAQNLYDP
TEVPEQTAPSAPSHNPSTIEDRGWYLFDRMSFGEKWQLMLGARASRYVSETTSTRYEADDVNPSVA
IMYKPVAGVSVYGSYLEGLEESGQAPAHRANAGELLPPALSKQKEIGVKAEVAQGVLLQAAYFDIERP
STTVDASNRFVLNGLAQYRGIELAASGEVTRYLALIGSALFLDAEQQNAANPATFGKVPDNTPERTAS
LFAEYRLPAVPGLALSGGLYYVGKRPVDNENRAFVDGYTTLSLGARHSTKLNGKRTTFQAVVDNVTN
ESYWSTAGNGLLGVGAPRTLKLTAKVEF

tonB next to motor, where ExbB predicted to have two large periplasmic beta-sheet domains. Unlike previous ExbBs seen.

>AAZ96312.1 TonB-dependent receptor, putative [Thiobacillus denitrificans ATCC 25259]

MEQGKRKPAWMRWGGVAVGLVAAVVIAVWLKDMLTPKGPVQKMEVRQITLVKPPPPPPPPPPPPP
PEEKPPEPEIKEEVKLEEPQPTPDEPQQADAPPPDGIAEGPAGGMSTDIARGVPTLPGSGNGNPWA
WYDALVNDAVNSAFQAALVREKALKNKTYKVIVKVWIDGSGQVTRVALAGSTGDAHADEVLKQALTG
MRALRDYPPVDMPQPMKIRVTSRA

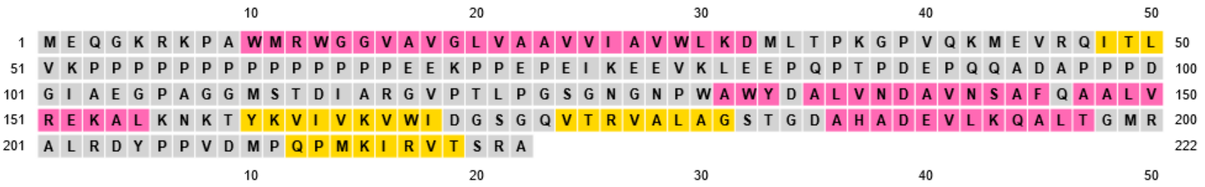

MEQGKRKPAWMRWGGVAVGLVAAVVIAVWLKD
MLTPKGPVQKMEVRQITLVKPPPPPPPPPPPPPPPEEKPPEPEIKEEVKLEEPQPTPDEPQQADAPP PDGIAEGPAGGMSTDIARGVPTLP
GSGNGNPWAWYDALVNDAVNSAFQAALVREKALKNKTYKVIVKVWIDGSGQVTRVALAGSTGDAHA
DEVLKQALTGMRALRDYPPVDMPQPMKIRVTSRA

PPIIPRED:

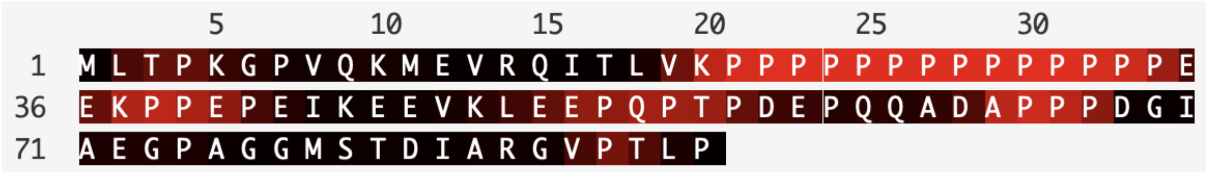

90 Domain II residues

29 Proline residues

0 α-Helix residues

Methylobacillus flagellatus

Locus:

>ABE49140.1 TonB-dependent receptor [Methylobacillus flagellatus KT]

MKYSTMGVSMLALIATSGPSHAHDELPLHSTERIQKLETTSVTARRRLEKEQDVPATVSVVKGNQLEA
GRIYQLQDLPQLMPSLTAQFLHPRQSSIAIRGIGNNPANEGLEGSAGIYLDNVFLGRPGLAVFDLLDIE
QAELLRGPQGTLFGKNTTAGVLNITTRKPVFKPEYSAEASYGSRDFEQYKVMLNQPINEMAALRIAAY
KTHDDGWVKNTYDGKNLNEINRTGARGQLLLQPSESFDLRLIAEHNEEDSSTGTLVPYRYGPWTPNA
FMFLGIHPVTGQPVFGPDYSSNLPAGTPGSNATDAITHANGLGASNRAYDPKDYKVTLDGRQRTRTS
QDALSAEANWHFNGYTLTSITAWRQWQFKPDNDLDRTDLFAVNGGVDARHKQYSQEIRLASPLSDV
FDYVAGVYYYYQDVSNREQYMTGPDAFALTTIYPDNSAFSGSGYAKTHSYALFGQGTWHLSEKLDL
TAGLRATMEKKEARVVQDQITPLPPILSPLLDHYDTGVQHQKDESLSSLLTASYWLNDRVLGYATYSS
SEKSGGFNTNGVASPGAVLGTEALNIDPEKANNLEFGIKSSWLDQRLVANASVFLTRVKDYQAITGTS
INGTYAGLLTNVGDLSSKGMELDLRARVSQHLSLSLSGAYTDASFDRGTAPTPAEEFNGPGGTSDSG
YGKGTRSIAGNQVNGASRWAFSSGAHLRWNPADNLEHYANAQYSWRSESYGDVNNSEYSKIAGYG
ILNVSTGLRVTSGKHRWDFSLWAKNVLDKRYFLGLTNAGSNLYVGSAGQPRTIGASLRYDF

tonB next to motor genes.

>ABE49144.1 outer membrane transport energization protein TonB [Methylobacillus flagellatus KT]

MSTESALARLSKEQYIEGQLSQTSTRNLVPKRTPRTPDAQHFSVKALIVTILVHAAVFTALIIASTREPH
REESAPPIMVSLVQSPEPVPEVAITEPEPQPTPKPPQPKPKPKPKPKVVDKPVPIEAPQPVEQVEEVR
EEVSEPQPQEAQQAPSQAPVQDAPVVAERIEPVPPPEPKEQPIPEVAISGVSYSHQEIPVYPAMSRRL
GEQGVVMLRILISETGVPESIEIESSSGSERLDKAALEATKKSRFNPYKRNNKPMKVSVIAPVRFSIAD

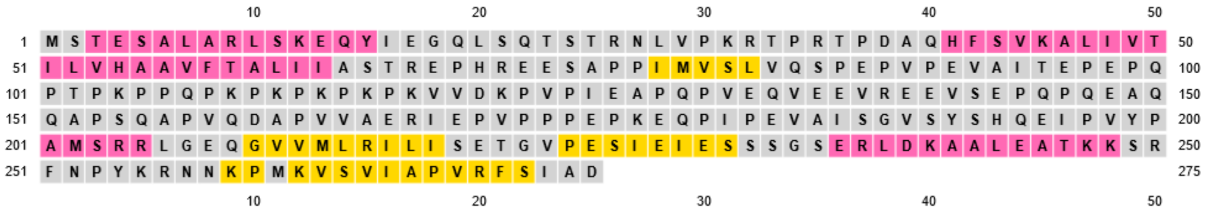

MSTESALARLSKEQYIEGQLSQTSTRNLVPKRTPRTPDAQHFSVKALIVTILVHAAVFTALII
**ASTREPHREESAPPIMVSLVQSPEPVPEVAITEPEPQPTPKPPQPKPKPKPKPKVVDKPVPIEAPQP
VEQVEEVREEVSEPQPQEAQQAPSQAPVQDAPVVAERIEPVPPPE**
PKEQPIPEVAISGVSYSHQEIPVYPAMSRRLGEQGVVMLRILISETGVPESIEIESSSGSERLDKAALEA
TKKSRFNPYKRNNKPMKVSVIAPVRFSIAD

PPIIPRED:

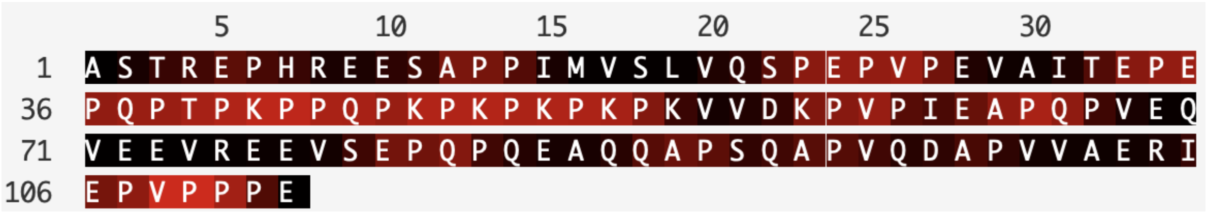

112 Domain II residues

30 Proline residues

0 α-Helix resides

Nitrosomonas europaea

Locus:

>CAD84232.1 TonB-dependent receptor protein [Nitrosomonas europaea ATCC 19718]

MYKTYAIWIGVVLFVCKDVIAQPDATGQEYSGDIPKVTMKEITVSSEALAIPNERLLLDTPTTTGSRLGL
TPRETPASINIIDRATFELRGAQTTQQILERSPGVTVSDQPGAAGTVCMRGFCGAQITQLFNGITVQYD
AVAARPIDNWITERVEVLGGPSSFLYGQGAVGGSVNYISRTANRDQQGHESLVLLGSWLNRRAAYG
YNGRIGDTNNWLQIHAGYKGSNGYIDKTRHNSGVFSFSLLSDLTSRISNTVAVEYQIEAREGYWGTPI
LNPVTAGKYDPETRFRNYNAENSVFDQQVIWVRDIVDFRLSEATQVRNTFYWYDAYRKYRNVEVYR
WNGDNTLINRSASFAVDHKQNLIGNRLELSHQQNLFGLPARWLAGTDIAFNDQTRFPSTESGLAVDTI
DPYNFTVGDYFDNPRASGPIKDRRNKLFTVAGYAENRLTLFPGFNLVSGIRIDSIQLDSRYFSPATATE
PAAFSRNWTPVTWRAGFVYDVTDSFNFYTQYSTAASPPAGVLTTTNLNSIRDFGLSTGRQIEGGMKF
DFWGKRGTATIAGYYLQRKHLSTRDPDNPINAIPIGAQSSRGVEVNLGVRLSPQWSFQGNMAFVDAR
YDDFNELVSGVSVSRKGNRPENVAKWVANTWLTWDFHPDFQWMLATRYVGDRYANAANTVPVKS
HVRLDTQLAWQAHRNARIIGRIMNLTDTDYIEWATSAPMYLIGAPRSYEVAVKLDF

tonB not near motor genes.

>CAD84265.1 possible TonB protein [Nitrosomonas europaea ATCC 19718]

MTKIRHKPALTGLSLVSFLVVVATHAAVLYGLWHHRFSTTTPDTITLYAQFIAPPEKQEEAAKAPKVEL
KAEHAPPQVKPTPPVKKVQPQPKHQLVAKTPAVTPQEYVAPPPVEEHEPEPEPEKKTVSESVIAAKP
AQMPTGPVTLSSELSVSCPDLASPAYPALSRRLGEEGKLVLQVELDETGRIGKAKIVQSSGYSRLDNA
ALSAVKTWRCRPATRNGHPVPAVALQPFNFVIEGS

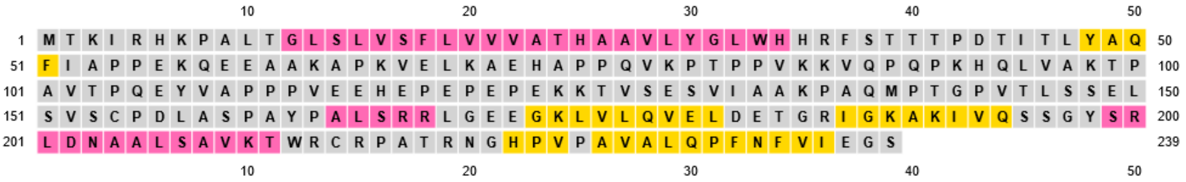

MTKIRHKPALTGLSLVSFLVVVATHAAVLYGLWH
**HRFSTTTPDTITLYAQFIAPPEKQEEAAKAPKVELKAEHAPPQVKPTPPVKKVQPQPKHQLVAKTP
AVTPQEYVAPPPVEEHEPEPEPEKKTVSESVIAAKPAQM**
PTGPVTLSSELSVSCPDLASPAYPALSRRLGEEGKLVLQVELDETGRIGKAKIVQSSGYSRLDNAALS
AVKTWRCRPATRNGHPVPAVALQPFNFVIEGS

PPIIPRED:

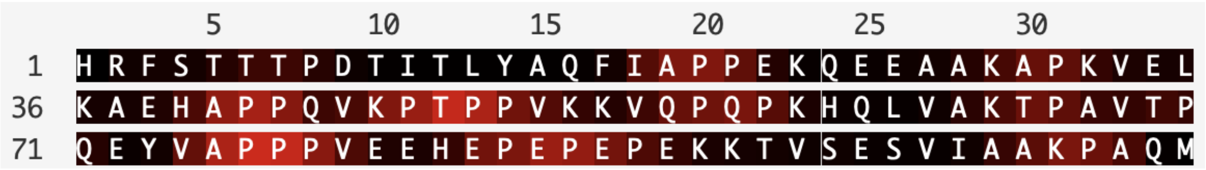

105 Domain II residues

20 Proline residues

0 α-Helix residues

Gallionella capsiferriformans

Locus:

>ADL54357.1 TonB-dependent receptor [Gallionella capsiferriformans ES-2]

MHQKLIIIALFGAITLPTVSFAETASLDEVVVTATRFATTSENTAVNVTVITAADIEKSAAKTLPSLLAQHV
GIQVRSTDGTPDLAVDMRGFGMTGNQNTLVLLDGQPLNDIELTSIHWSAIPLDSIERIEIVNGSGAVLY
GGGASGGTIHIVTKHPGKGVHGIASVGAGSYGAKEWQLALSGSGERLGMRITASGLDTANYRANNN
VTQNNLEADIRGDVGQGDAFVKFGADNQTIRYPGNRSVNPNTGVNQLLTDPRGTATPLDYGTRNGV
HVSLGTAQQFEFGEFAGELSYRDTKRTAYFAAFGGAYLDTALNLLSFTPRIKVPYQLGGAANELTVG
MDLSNWNYDSSRSSAPANMGTPTTKILAGQSDRALYLQNVNQLGADTKLTLGARSQRVSYQARDAV
NPAAYASANQVRTANAYELGLRHNLNQTLAVFGRIGRSFRIATVDEIFAQYGGPFFDSKVTMLEPQTS
QDSELGLDYKSAKDKIRATLYQMNLNNEIHYNALTFTNMNLSPTRRYGLELEGTHAYSDSVEVGAAY
SYTVAKFSNGTYGGINVSGNNIPLVPRQRLALSTALKLTEKTSLSGSAMYVGQQHFDNDQANTFGQK
MPAYTTVDMKLTHREGAWQLAAAVNNLFNRQYYTYAVSSTFTPGVYNAYPMQGRNFSLNASYQF

tonB not near motor genes.

>ADL54217.1 hypothetical protein Galf_0172 [Gallionella capsiferriformans ES-2]

MKSRFLHNESARIAIALGLSVLAHIAILTLQPHFNAAPFAHQTTGKTLSVTLNKNSAKTPPRPSTINLTST
LKPAITPSSPAAAATSDTHYFNTNELDQPPHTDQDQLAFNLPDPPKQIDIVMRLWIDKSGKVVKVEPV
TAELSQALISEIRSHLMNSRFSPGHRLGYPANSVVEITLHYE

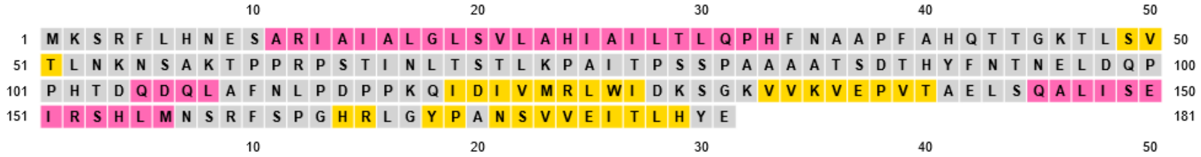

MKSRFLHNESARIAIALGLSVLAHIAILTLQPH
FNAAPFAHQTTGKTLSVTLNKNSAKTPPRPSTINLTSTLKPAITPSSP
AAAATSDTHYFNTNELDQPPHTDQDQLAFNLPDPPKQIDIVMRLWIDKSGKVVKVEPVTAELSQALIS
EIRSHLMNSRFSPGHRLGYPANSVVEITLHYE

PPIIPRED:

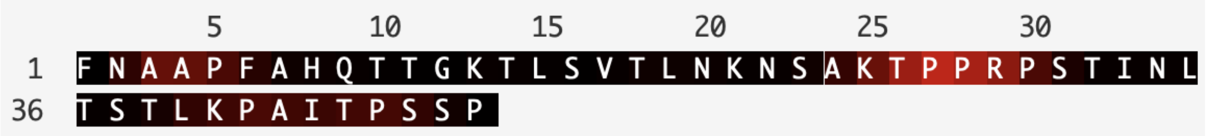

48 Domain II residues

7 Proline residues

0 α-Helix residues

Acidithiobacillales

Acidithiobacillus caldus

Locus:

TBDT gene on different contig

>OFC32715.1 TonB-dependent receptor [Acidithiobacillus caldus]

MKHPTALRCLSSAVLASLWLVPASILAATAASDGGIDIGEVSSSTNAAGTAAARAKALRESPENQVQITHQQIRDLVQPGGSIVTA
LSAAPGVQVKGYGSGNGASRYQIRINGIQVGFALAPGNPEKNGLSVLFDGVPMNNPLAQYDGWESSETPISSIFSNIHVTQGPG
NPDHRWYDSMGGTINLIPVEPTAKAGASLDLGGGSFATYSASTMLQTGSINGWRSELGGGYTRSAGFRTGPYHAPNQAEAFFA
KTIKDFHGGHFSAGFFFSHTQEFRSLYIPASPIPGVTTGGYGQPGELLSQGTTGYYYTVPTSQYFKNDTAHMYLVYARLMVHLA
QRLSVDNTLYFRSGYRHHYRVNDYFPHTVNTEDFTGTTRTIGDRLAFHWNLPMNSVRFGGYYQHSRYESLYYGYNPAVLASSA
SKPLFAADYYDYWDGATAFLQDDFHPISALHITPGLQLINYHVQFVNNSEAAIPPGSTPVTYKAGNNDNSYTKLAPSLGINYRILP
GLHAFAIWAKNFQTAPAAAYGNYQQATVEVPKSPTDINSYIGGLKWHMGAWQAQLSGFHQHLGNAVIATFLPSDLISKLDRVSAI
YNGVNLLLQYGKGLGFFAGTRDTIQHAYYPSYLPAGGSPVIDARMPGTPTLILGFDAGYRWYAANTRFAVRLSDQYASSVTTFN
NNTNLPDPTRLPGSAYNIVNLGLSADTLAFDSMIPGLKRVGVSLMIDNLLNRKYNSQGYITSGGEYGPNSQGAILVIPGAPRAVYA
SFSASF

tonB not near motor genes.

>OFC35725.1 hypothetical protein BAE27_07210 [Acidithiobacillus caldus]

MKRQARGRRGFLRDLRARAPWSAYFGLSARDLDRLFPWLLLSSLGLGGIFLFVRDVGLLHVPRVPPI
SSYVQIDLVSAPAEATGTPALPMPTRGPGLPVVRAPATRAPSTAQSDPKAQLAAYLLAWERRVAEVA
GASLAGHSIPQGRLIVAITIDPTGQLRRVEILRGQQHRDLVLAVESILQQAAPFPPLPPSWQHPPQELRI
VRTWNFE

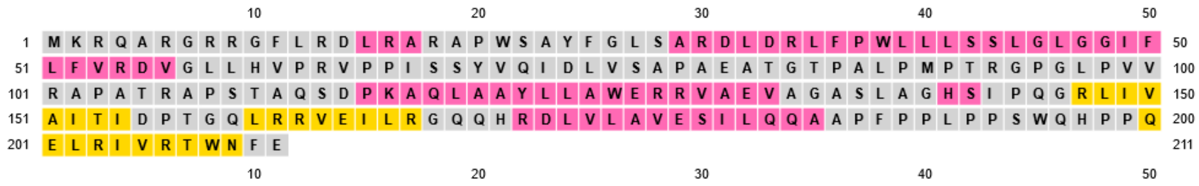

MKRQARGRRGFLRDLRARAPWSAYFGLSARDLDRLFPWLLLSSLGLGGIFLFVRDV
GLLHVPRVPPISSYVQIDLVSAPAEATGTPALPMPTRGPGLPVVRAPATRAPSTA
QSDPKAQLAAYLLAWERRVAEVAGASLAGHSIPQGRLIVAITIDPTGQLRRVEILRGQQHRDLVLAVES
ILQQAAPFPPLPPSWQHPPQELRIVRTWNFE

PPIIPRED:

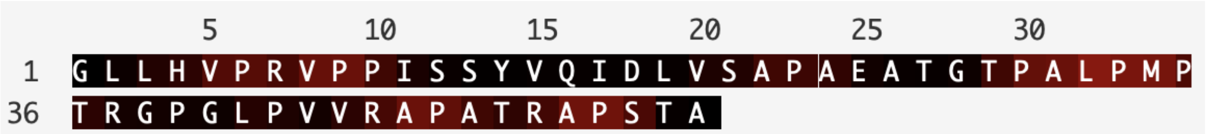

55 Domain II residues

11 Proline residues

0 α-Helix residues

Acidithiobacillus ferrooxidans

Locus:

Different contigs to *tol-pal* genes.

tonB next to motor genes.

>OAP87712.1 TonB-dependent receptor [Acidithiobacillus ferrooxidans]

MYKKLPLKPLLVAIAATGALNITVMAAAQAGVTAPTTSAPTHRIINVGRVSAGLALNTLNKVPTKKEVFE
STQSVKVIGKKQMETAGPAGGAGQALAVAPGVNTQTDSPAGASRTTISINGMKTGWSNIAGNANDG
TVMVTFDGVPMVDPAYGVWGSPEVPQMSMIKGIAVTYGPGYPVNRWFNNIGGSINFIPLQPTAKAGA
SIGGFYGSFNTRGVHFNVRTGNIDGWSAILAGGMTSTGNYLTGYGFNNPSNNYAYYAKVIKRFHGGH
FSFGAYAARGVSYRPLGIPVSPNSNVTVNGTDANGNPIPGPIYSQQTTGFYTTLPYSEYWKEAFNTSY
ILYSKFFDHLSRDLTLHNLLWYRHGSREHLHYNYYTGGNSNVAMNQYYLAKSDTYGDKLYFTDRLPW
NTVSFGGYFLNNKYNSLLEFYNPTQASPINGAQVYGSLAVPNGDSHSSYMYMTDLAAFIQDDIQPVS
NVRITPGLRVVTFNTNFVNNEAAQFPLNGYNNGVNSYGTSGDRQPNSSTNFTDVEPSIGANWQITPN
VAVYANYSTAYKAPAGATGTYAHMLASSLKPQSSSQYQFGVKAYVPHDGILNDASFGANYYHLNDT
NEIIPIPVVSHLYSEFASGSSTFSGVNLYFEDDPLYNIHIFSNLSFEKAVYSNYVNPHGVSYDGMPVSD
VPAQTANIGAYYQWYHAGVVYEPRIWWQYTGAQNIYNNNTGAPTNQKLPAFGIWNAALRVNVGHQY
LFAGLHKLSVNVDVLNLLNKQYNSYQYISGGGYYGVAGQMLADPGMPRAVYVSLEGHFA

>OAP91342.1 energy transducer TonB [Acidithiobacillus ferrooxidans]

MNTIVSDPSWAPQRPKGHFGRALLIGAVLEVVLVGGLIWGGSNTPAPKLMVKKIIAIHMVQPAPPKPK
PVPPPPKPVVHPKPVPKPLPHPIPQPRPKPVVHHAPAPKPLMAKTPLPTAPVVPPSPPVTTPPPPPPP
PAPSMAAREAAIDQYAGMVRTRVQADAHVPEAVRLMHLSGTAVITFRLTPSGRLLWARIAQSSGVGP
IDKAALKSVEEGTYPPFTKNMPKHASNFNVEVHLSSHSS

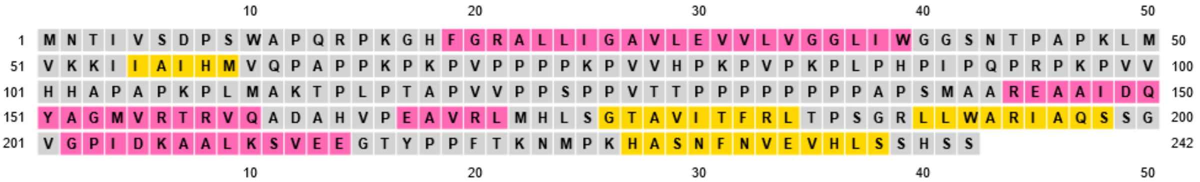

MNTIVSDPSWAPQRPKGHFGRALLIGAVLEVVLVGGLIW
**GGSNTPAPKLMVKKIIAIHMVQPAPPKPKPVPPPPKPVVHPKPVPKPLPHPIPQPRPKPVVHHAPAP
KPLMAKTPLPTAPVVPPSPPVTTPPPPPPPPPAPSMA**
AREAAIDQYAGMVRTRVQADAHVPEAVRLMHLSGTAVITFRLTPSGRLLWARIAQSSGVGPIDKAALK
SVEEGTYPPFTKNMPKHASNFNVEVHLSSHSS

PPIIPRED:

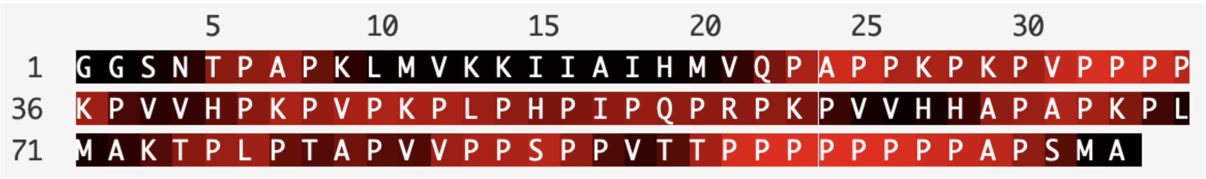

103 Domain II residues

41 Proline residues

0 α-Helix residues

α-Proteobacteria

Magnetococcales

Magnetococcus marinus

Locus:

>ABK46045.1 TonB-dependent copper receptor [Magnetococcus marinus MC-1]

MKARSHLAMLSALLALPITSVQAQQSTTLAPLTVEDTLLEEDGAVILYMDNRNKGPVADGGELLKTIPG
VSAARKGGHGLDPSIRGQNQTRLNIVLDGAFVHGGCPNRMDPPSAYGASESYNSVTVLKGAQSVEY
GAGGSGGVVLFNRQTETFDADERYRGKAGVGYRGNINALESYLDMASGTPSAFIRFIGNYSDGNDY
KDGDNNVVRSSYTESTGNLLLGWTPDAENRVELSHEASYSSDMLYAGAGMDAPYTRNNVTRFKFA
RGLHDGVMQKLEGQISYSQVAHLMDNYSLRTAPAAAMYMQAPSTSDTLTGKLSLQFNHAGMDWKV
GVDHQGNQRDAVRYRGTVGAVTNLQSYLWPDVTTLTTGLFAQLDRPLGETNRLKAGLRYDYVRADA
GKTHATPNDAMGALAKLSGHDLYTTYYGGSTDASTEHNMGGFLRYEQGYWGNQGNFYVALSRTVR
TADATERYMAANHNTSADSRWVGNPFIKPEKHHQLDVGTRIQWQALKSDVTLFYNRVDDYILQDHQ
HSAGADGNATVYHNVEATLLGGEAAFTYNFDPHWVGGMSLAYVYGHNETSNQVLAQIAPFEAGFTL
DYRADRWDFGGKLRVVTKQTRADTDSTTATGLDTDQTPGFSTLDLYSAYRPMPNLTLKMGVNNLFD
KRYAEHLNASNSFDATQLQVNEPGRTLWFTLGTEF

tonB next to motor and TBDT genes.

>ABK46046.1 outer membrane transport energization protein TonB [Magnetococcus marinus MC-1]

MPTPAVQPRPAPESFQLVLLDLPTQPAPAQPATVAPQVGPEPPAEPLPPVAETVESQVVPE
PLPEPPPPVAETTEPQVVPEPLPEPPPPVAETTEPQVVPEPLPEPPPPVTETIEPQVVPEPL
APSAPPQRVPESVLPATPRAHAQAPPLPRVVPPPLPKPRARPVVLPVTAAQPMLVAPLPSS
GAMAKPVTERPSKAAPPTAPRPAAVMFPYIPPQYGDNPLPAYPPRARHGRLQGSVLLEIQV
SKTGLVIGVTILQSSGHLILDRTARQAVKRWSFKPALRHGLAVAATVQVPIHFRLNRGAKPH
A

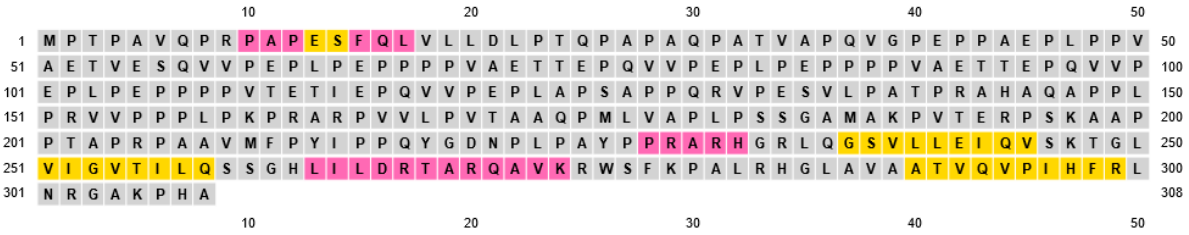

Appears to have lost its TMH- no further analysis.

Rickettsiales

Rickettsia prowazekii

Anaplasma marginale

Rhodospirillales

Acetobacter pasteurianus

tonB and TBDT genes on different contig to *tol-pal*.

tonB not near motor genes.

>OAZ72116.1 Catecholate siderophore receptor Fiu [Acetobacter pasteurianus]

MSRSELSHPSSQGKGLRVAVALGVSVGLGVTESKAQAEDAAQPLQPSSSDTSIKLSAVRVGGHAESKNESYEAGNTNSKILNIS
RMPASVRDTPQTINVVPQELMKQQRAFTLDQALSNVPGITLSTGEGAGGLNGDQFRIRGLQARQDIYTDGLRDFGTYTRDVFNT
ESVEVIKGPSGEYFGAGNVGGVINQSQKHAHAGNSYSYDQSFGSGPLFRGVGDINYQINDDMAIRVNGMFNKQDVVDRNNVTS
NRYGAAVDFGVGLRSKTSWHLTWQWLNSDSKPDYGVSMIQVNGIYRPITEYGLSRNTSYTRNFDFDRSNIHSLTSSLKSEISRW
FTLTNDTRLSMYNRDYTATTPAACSGACATAFLAGGNYALPYGAGGGAAYRQEGWGVQNILMGRSQFKTGFVHHDIKAGVDV
NYASDSRWPGSFTNRVNNQTIRNPQYSYSGTSVSFPSSGHGNANARDVGLFFSDRIQLTKQLSLFGTGRWDSFSSTYYARSKA
AQGRAEQKSDRWSPSASIMYSPLKNASFYFTFARSYKPVGTDVSSLVTIKPQAGDVAQKGVNLSPQRSDLFEFGSKADFLHKRL
GTTVAFFQISENNSRYYDVNGDMETGFADSGSGRRIRGVELSANGKLTRDWQIFASYSYMDGRVTHSSTGDNGKTAPQVPHN
TMSVWSSYDLSRQLLNPEWGTLKIGGGVQYSSGYWAGPDTANTARIPNTFLLNGMLSWDYKHYRVSFNANNITNKLNYASSFS
GSRAVPALGRYFIGNIGVTF

>OAZ72119.1 Vegetative cell wall protein gp1 [Acetobacter pasteurianus]

MSATTYKNIAGKVHSTTFSDWKIQQIRKARQEDTFRWGISFLIVVATTGAATLSFLHMPHTAPAIPEPP
PAAIAIDMAPEPVATPSPPTDAPPGPQQTISESDPSPVDPAKLEAPPAPVPHPPIPVPQPEKLHKIVKK
NKPTVNLKKPVPDKTPPAEKTTAPPTSKAPPAQTQAAPIEGSSSSHASQSPNTWQGALLARLEKYKR
YPAEAMSAHQEGTPNLHFTMDRKGRVLSAHIEKSSGHSLLDSEALALVRRAEPLPSPPDSVAGDSITL
TVPIEFYMEHAQD

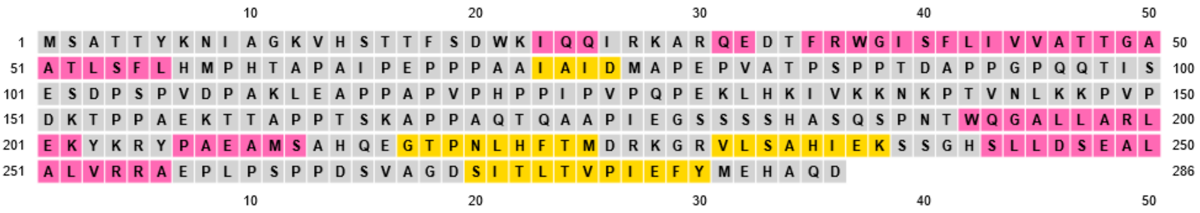

MSATTYKNIAGKVHSTTFSDWKIQQIRKARQEDTFRWGISFLIVVATTGAATLSFL
**HMPHTAPAIPEPPPAAIAIDMAPEPVATPSPPTDAPPGPQQTISESDPSPVDPAKLEAPPAPVPHPPI
PVPQPEKLHKIVKKNKPTVNLKKPVPDKTPPAEKTTAPPTSKAPPAQTQAAPIEGSSSSHAS**
QSPNTWQGALLARLEKYKRYPAEAMSAHQEGTPNLHFTMDRKGRVLSAHIEKSSGHSLLDSEALALV
RRAEPLPSPPDSVAGDSITLTVPIEFYMEHAQD

PPIIPRED:

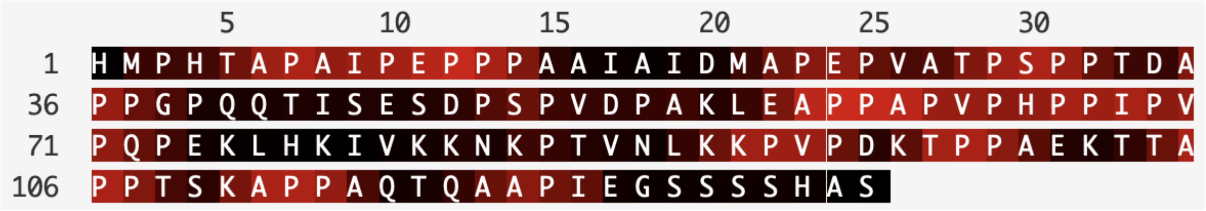

130 Domain II residues

36 Proline residues

0 α-Helix residues

Rhodospirillum centenum

Locus:

>ACI98016.1 TonB-dependent receptor, putative [Rhodospirillum centenum SW]

MKMTDTQRRVRRAALLAGAAVALAMPAMVPVHAVAQEAGALEEIVVTGSRIRAPNLVSTSPVTAVTGEDIKAQGVTRVEDLVNS
LPQAFAAQGGQVSNGSTGTATVNLRGLGSSRTLVLIDGRRLMPGDPSVPSPDLNFIPSSLVQRVDVNTGGASAVYGSDAIAGVV
NFIMERDFEGVRVDVQHGFYMHHNENDDVQSIIRAKSASASVPTDFAVPDSNVTDGEQSEATLVIGANSADGKGNVTAYAGYR
EIKAVLQGDRDFSACSLNSGDVFTCGGSGTAYPARFGNWVVDPAGPGNTFRARNASTDVYNFGPTNYYQRPDERYVLGAFAH
YEIQPWADVYADVMYMDDRSIYQIAPGGIFAGTFSVNCDNPFMTPTQQQQLCGANAGTQNNFTGTIARRNIEGGGRQGDMRHS
AYRFVTGVRGDITDEWNYDAYLQYGATSFNRAQTGFFQTQKIRNALIARRDANGNIVCQAAIDGSDTSCVPYNIFKIGGVTQEAL
DYLETPSYATGNTEEMILSASLAGDLTNYGVVSPFATSGVGVAFGIEYRSEYLDSASDYTAAAGLLNGSGGASLPVEGDFDTKEI
YGEIRIPLVEDKPFAQSLELDAGFRYSEYSSVGSTDAYKAGLIWSPVSDLRFRGSFQRAVRAPNILELYDPQVVQLDGSTDPCAG
LSAGDALVARCAQAFGLTAAQVLAIEENPASQYNGQNGGNPNLDPETSDTYSIGVVLTPEFAPGLTVSFDYFDIKVEDYISGIGAD
LIISRCLETLDPYFCGLVNRDANGSLWLSDQGYITNTTLNTGSLKTTGIDVNIDYSLDLEDVGLNNAGSLAFTMAGTWLDTLKTQP
LPNDKAYDCTGYYGTVCGTPNPEWRHKARLTWTTPITGLTLSTQWRYYSKAELDATSDQPGLNNPAQVAGTDLKLGTQNYFDA
TITYQFLESYTLRIGANNIFDRDPPLTGSANCPTGPCNGNTWPQVYQDAMGRYVFVGLTAKY

tonB not next to motor genes.

>ACI98096.1 protein TonB, putative [Rhodospirillum centenum SW]

MGTAGAGPGILIPGRVPAPREGPPLPLLAAATPAPLSPDPAARRARITRRALTLSAAAHLLGLGLLAGL
AADPQRRAPLEETAVEVLVRPAGGEPSRAALRRAAAAALATVTGMPPATALDGTETAAGPETPQGLP
HLAWPELPVRIAPPRLTAQATQALRAPDAPPPLPAPEPVDLESFIPRVPAGREASPSPARARPDNPTP
AYPEAARRQGLQGKVLLRVTVGPSGEPVQVSLVESSGWALLDDAALETVKGWRFDPAHRDGLPVA
AVVEVPVAFSLDQP

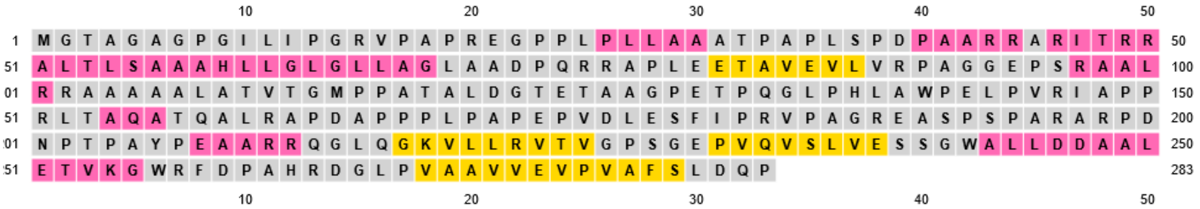

MGTAGAGPGILIPGRVPAPREGPPLPLLAAATPAPLSPDPAARRARITRRALTLSAAAHLLGLGLLAG
**LAADPQRRAPLEETAVEVLVRPAGGEPSRAALRRAAAAALATVTGMPPATALDGTETAAGPETPQ
GLPHLAWPELPVRIAPPRLTAQATQALRAPDAPPPLPAPEPVDLESFIPR**
VPAGREASPSPARARPDNPTPAYPEAARRQGLQGKVLLRVTVGPSGEPVQVSLVESSGWALLDDAA
LETVKGWRFDPAHRDGLPVAAVVEVPVAFSLDQP

PPIIPRED:

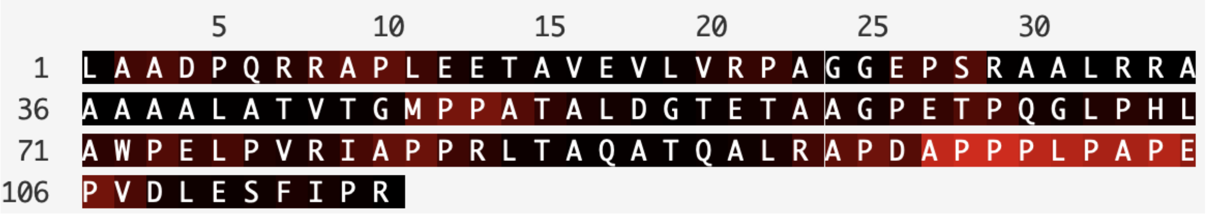

115 Domain II residues

21 Proline residues

8 α-Helix residues

Sphingomonadales

Erythrobacter litoralis

Locus:

>KEO90001.1 TonB-dependent receptor [Erythrobacter litoralis]

MMRFSLLSGAAGIAFAAIAAAPAIAGEVRGTVVDASETIALRAAQIEIEEIGRQVNSERGGTFDFGEVPAGTYTITARYVGADPVSQ
RVTVPETGAVTVNFALGQSGSQILVVGGQGASQASALSRKRAADGVSDVLTRDAIGQFPDQNVAESLRRLPGINVLNDQGEGRF
VAVRGLDPNLNATSVNGVRIPSPEGDIRGVALDVISSEIIESIEVKKSLTPDMDADTIGASIEINTTSAFDRKEDLYVVKLGGSYNEL
RDTLTPDLGADFAAKLTDNFGVSGGISYYNREFQTDNVEADDWTEDDGLIYAEEVQYRDYLVERERISATLGFDARVGSSTELYL
RGVFSQFDDQEFRRRLTFDLGDANVSGSGLNPVFDDIRDPADPDDEAAIAVERDVKDRFERQRIRTVTFGGESQWGGLKAEYA
LAWAKSSERENGSVDPTIFVGEFEDSGLGVGFDYADERRPRFNVTGNTADFFDPSFYELDEIEFTALSDAEDEEFSGRLDLGYE
WFTDAGTLTVQGGAKARLREKRFNGEIEFYERDDYTLADVLGEGPSNTLENLAPLPGLTQATDFFRDNFASFELNAVDTALDSA
NGDYSVEEDILAGYLLGRFENDQWLVIGGVRYERTDNRLSGFETALFEEGQDLPGGGVADDDTVIVTPVEFERDYDHWLPSLNI
RYTPMPEFVMRFAGYRSIVRPNLEQLAPRFELDENREAVIGNPTLEPFEAWNLDASFEYYMTGNGAITAAFFYKDIDNYVTTLVL
DQPGSIFGVDYEQGETFVNGPAAEIFGIELGFYQRLDFLPGFLDGLLVQANYTYTDASGLVADGEVGDITAAPTFREIPLPATSEN
TFNVALGYDKGPVSLRLAGTYRDGFLDEVNADGPEFDRFTDSLFQLDFTARYRVTDKVQVYFDWININDAEFYAFNRLGGRQNI
LQFERYNWTMKGGVRLNF

tonB on different contig, next to heterologous motor where ExbB subunit has disordered cytoplasmic domain.

>KEO91069.1 energy transducer TonB [Erythrobacter litoralis]

MAYADQQMSGNKVVSIVIVALIHVAVGYLLISGLAISAAKKIIERVDTFNVEEPPPPPEEPDEPPPEQPQ
ETAPPPPVAPPPPINIAPQPPQIQTQREIPPPSPPALVLPPPAPAAPPAPPPPPSQARGVQPRNQSRW
VQRIIEDYPSRALRQEEEGTVGVRVTVGADGRVAACSVTSSSGSSILDDAACRSMQRYARFEPALND
AGNPISGNWSTRITYQIR

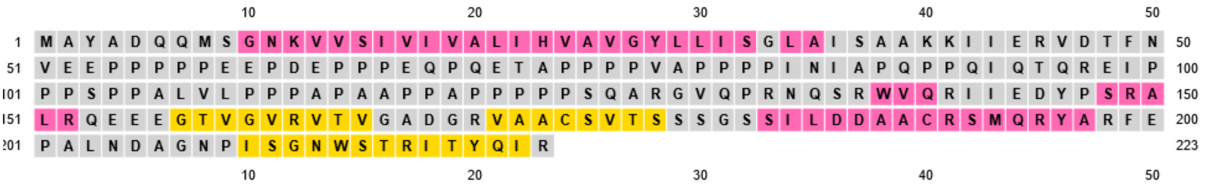

MAYADQQMSGNKVVSIVIVALIHVAVGYLLIS
**GLAISAAKKIIERVDTFNVEEPPPPPEEPDEPPPEQPQETAPPPPVAPPPPINIAPQPPQIQTQREIPP
PSPPALVLPPPAPAAPPAPPPP**
PSQARGVQPRNQSRWVQRIIEDYPSRALRQEEEGTVGVRVTVGADGRVAACSVTSSSGSSILDDAA
CRSMQRYARFEPALNDAGNPISGNWSTRITYQIR

PPIIPRED:

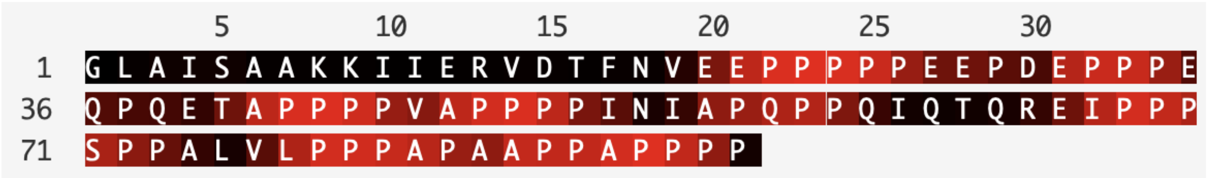

91 Domain II residues

36 Proline residues

2 α-Helix residues

Sphingomonas wittichii

Locus:

>ABQ66648.1 TonB-dependent receptor [Rhizorhabdus wittichii RW1]

MKQSLHIASIATIAFLTMIPVEAQAQEAARAAPADEAPAAGGIDDIVVTARRRAERLQDVPLAVSAVST
AALERANIQNVAQLSRLVPSLTSVPGQGGSRSLPNFSIRGLSQQELTILADQSVSTYIGDIVAARTQGI
NSALFDIASVEVLRGPQGTLFGRNTTGGAIIIRPARPTDEFEGRVGVTVGNLDTFNVEGMVNVPLSPNI
AIRVAGQRQRDDGFVYDEILQRNVNDTKQEGARASILMHNDDLESLTVYNYFHENDGGTASFLQNVR
ATGSLNGAAVRASRGYRPLEDLLAEQQARGIYRIANGSPIFTKVETHDIANTTSFDLSETIKLKNIVGYR
HVKDHIFDDMDGSSNALHPQERIDRAKQFSEEFQILGDTANLNWIAGLYYFRESGRNQGISAVGAVD
PGLVEPDNVAAFPGTAYSNTDTAARNTSYAAFAQGTYRFGGGLSLTAGIRYNRDERQATIRNRTATA
CRFTRDLDNNPATPETAVPLAQCELGVKDSFSEVTYNVSLEYKIAADKLVYIAHRHGYRTGGFGARAS
TEAGLRRTFRPETVDDVEVGVKADWRMGDAFLRTNLAGYYAKYKDIQRLLTDPVAVPPTTVTTNAGR
ARIYGIEADILFRPVKMIELTANYAYTNAKFTKFIYPDGTDHSNDPFARAPRNVYTLGARLLAPLDEGQ
GDASVGVSYYHTGDYSGNDTYVPGYTDVKGYSLLNFDASWNRVMGSGFDVALFVNNVTKKKYDFLL
INLDSLGYTSHTPGMPRTYGATLRYHF

tonB next to motor genes.

>ABQ66489.1 TonB family protein [Rhizorhabdus wittichii RW1]

MAYGDQTLSTRKVVSIALVIVLHAVIGYAFVTGLAYNVVKKVARDLKTFDVAEEAPPPPDQPPPPPPET
RIEPPPVVAPPPIVQVAPTMAPPIVSVPVAPPVVITPAAPPAPPPPAVSRRGEPRGTPGEWVTPEDYP
SADLRAENQGTTSFELAVGPDGKATDCKVTNSSGFPSLDTKACQMLLRRARFKAQLDGNGQPMPFT
YRNRVRWQIPKD

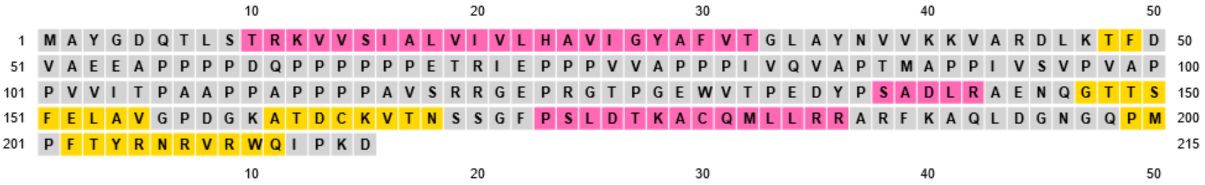

MAYGDQTLSTRKVVSIALVIVLHAVIGYAFVT
**GLAYNVVKKVARDLKTFDVAEEAPPPPDQPPPPPPETRIEPPPVVAPPPIVQVAPTMAPPIVSVPVA
PPVVITPAAPPAPPPP**
AVSRRGEPRGTPGEWVTPEDYPSADLRAENQGTTSFELAVGPDGKATDCKVTNSSGFPSLDTKAC
QMLLRRARFKAQLDGNGQPMPFTYRNRVRWQIPKD

PPIIPRED:

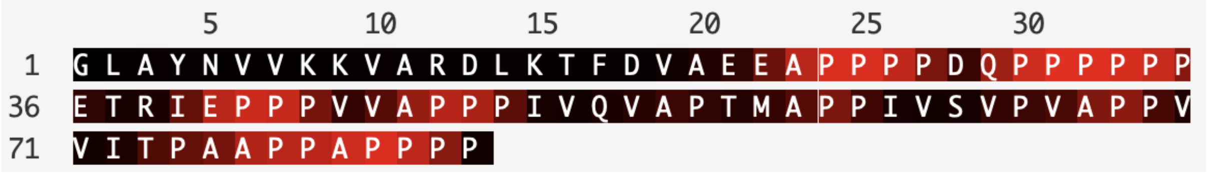

83 Domain II residues

29 Proline residues

0 α-Helix residues

Rhodobacterales

Hyphomonas neptunium

Locus:

>ABI77930.1 TonB-dependent receptor [Hyphomonas neptunium ATCC 15444]

MKRANMKTHLLCSAGLLAITAMGHSLPAIAQENDTSADEVSRTLETVTVTAQFRDQQLQDVPNSIVAF
TAQDIEDAGIRSTEDFIALTPNVSFDNSFTYLNSFVVVRGVTQINNADSPVAIIVDGVPQNNQKQFKMP
LFDVEQIEVLKGPQGSLYGRNAIGGAINIVTRQPTNEIEGFVSGGLSNGSGYRMEGGISGPVIKDQLM
FRLSGSYYETDGLIENAYLGRTVDTVDHDYSIRGKLAYTPNDVFSLDLRAAYTDARAGAAYDAVVNSQ
LNPDFRSGNDSNDIFAPASNLAGITEADILDLSGKASLDMGFATATYILGYTDLTETYRSDLDFSNPDQ
PTFGPFGQLGQGQNLSLDLLSQELRFVSPDERTFRWIAGGYYLETDRSLLTRGFLDLTGSVDQFDTG
IAIIDRSEDNDNTAWALFGQAEFDLNDRTVLQLGARYDEDERNQTQGAAGPVRTRSFSAFQPKITLSY
DILPDVLGYATYSTGFRSGGFNAPGVVLDSFGDETLTNYEAGLKTGFADGNGTLNIAAFSSESEGFQF
FFVDASTVSQIISNLDKVDLRGFDADFRYRVSESFTLSGGVGYTDSEIRSIGNAQLESYLTASGVDTDA
VIGSRSPKTTDWSVSLSGQYVRPVRSGLDAVLRADYEYQGNKYWQIDNLDVRDPVNLLSLRFSLESD
IWSASLWGKNILDEEYYADFNPAEYAGAPFDLGSQARPATYGVDFKYRF

tonB next to heterologous motor genes including ExbB with large periplasmic domain.

>ABI75541.1 TonB family protein [Hyphomonas neptunium ATCC 15444]

MRLLVGVVIAIPIVVILFLMMSALISVDEIELAEGENRTLAAITPQKQDSEVRTRSRSQPKRIDAAQKPPP
PPKQSATKSSINLPTPKIDGAVPQNLDLGRMNSLAIDPVAVSDRDAQPIRPPTPSFPQRAAERGLSGE
CDVRFDVDTRGKPYNISATCSDNIFKSEAERAVSRVEFAPKIVRGQAVERRNVVYPLEFKIQ

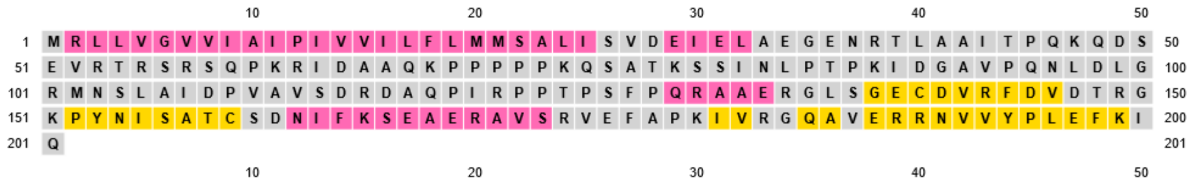

MRLLVGVVIAIPIVVILFLMMSALI
**SVDEIELAEGENRTLAAITPQKQDSEVRTRSRSQPKRIDAAQKPPPPPKQSATKSSINLPTPKIDGAV
PQNLDLGR**
MNSLAIDPVAVSDRDAQPIRPPTPSFPQRAAERGLSGECDVRFDVDTRGKPYNISATCSDNIFKSEAE
RAVSRVEFAPKIVRGQAVERRNVVYPLEFKIQ

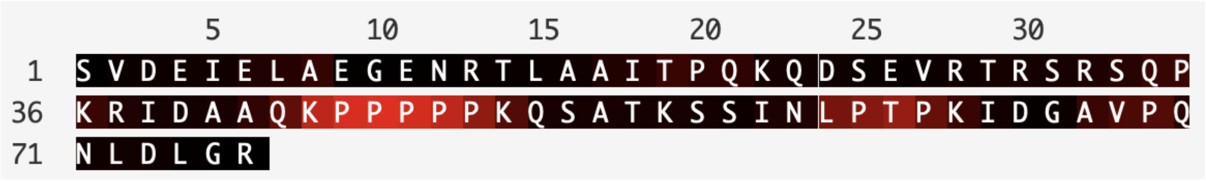

76 Domain II residues

10 Proline residues

4 Helix residues

Rhodobacter capsulatus

Locus:

tonB next to motor genes.

>SDE40641.1 protein TonB [Rhodobacter capsulatus]

MSLALTDPPEAPGRALLWGLALSVALHAVAVSVLSQTEAAPAALVAAGAGGEDLDSLDAIAATFVDLA
PQITLPEPEMTLPLTAPVIDIPEVRLPDPVLLPPEPPKITPPKVAEDKPTPPPPKPKPEPRAEKPKQAKP
APAQSESRAASRAAGSGAGAQAGTGGADAQATVSAGTAKSLLSKWGATIRTRIERRKSYPAAAGRA
AGSVGLALRVSRDGGLISAAVNRSSGHPALDAAALAAVQKAGRFPAAPAELAKPSYAFSISVKFSR

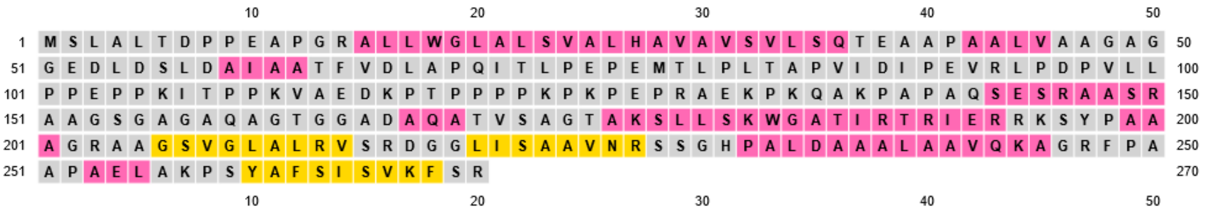

MSLALTDPPEAPGRALLWGLALSVALHAVAVSVLSQ
TEAAPAALVAAGAGGEDLDSLDAIAATFVDLAPQITLPEPEMTLPLTAPVIDIPEVRLPDPVLLPPEP
PKITPPKVAEDKPTPPPPKPKPEPRAEKPKQAKPAPAQSESRAASRAAGSGAGAQAGTGGADAQ
AT
VSAGTAKSLLSKWGATIRTRIERRKSYPAAAGRAAGSVGLALRVSRDGGLISAAVNRSSGHPALDAA
ALAAVQKAGRFPAAPAELAKPSYAFSISVKFSR

PPIIPRED:

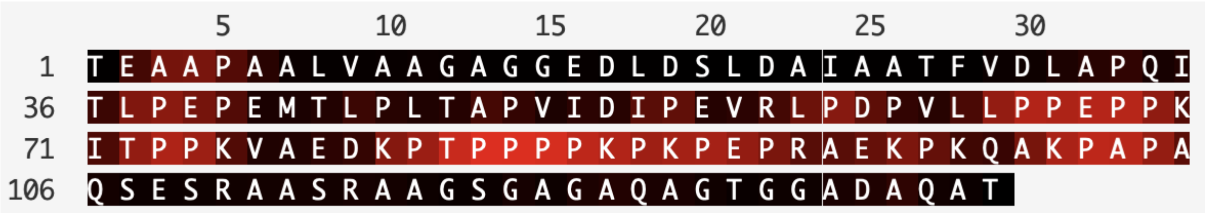

134 Domain II residues

26 Proline residues

11 α-Helix residues

No TBDTs found in this strain as poor coverage- used a different strain.

>TKD26225.1 TonB-dependent receptor [Rhodobacter capsulatus]
MRLASRQLAAFLFCSTALSLPLAAEQADDTLWLGQIVIGYAEDGSPILAGENATHLTADDLRGATSTS
DLDGLLRVQTSVFTQKDPGNPGVSVNIRGFEGSGRVAMSLDGVPQTYRLTGHAAQGYVFVDENLLS
GIDITRGPVTGLGGSGIAGSANFRTLEVGDVLKEGQTRGGLVRLDHASNGDTTSGMAAAAMKAGRF
DALAAISRHSGESYEDGDGVEVPNTDTDISSALVKLGYDLTETQRLTFSLMRYETDFFATSYFQHLTN
DVAKIGYHYDPGSSLIDLRVNAYAATTETDWLRGGSPFASSVGRNMSTKTRGLDVTNTSELRFGGW
QLTSVNGIEASQDSLGGSKGGVNPTTGKARRLSAFSENVFVQGKWEITAGLRANSYRLDGEASQGTI
DIKNSSLDPKLTLAYQATDWVQPYVTLSRAMRSPTLQETMLGGSHPGGGIGMIANPRLEAETEEGVE
LGFTLDRAGLFAEGDRLSGRVNYYNMEVENYIVTSMAFTNAFGQTGAAFVNVPGRTKTEGVEIELDY
DHRAFDLGLSYTHNSSHLPSQTPGLGAGQYLPDDTVSVRLSRDFMADRLTLGAQYTHVSGGLFTDA
YTSTPYQKDDSYELVDLFASYDVTEDAVIYAKVTNLFDEVYAPWLSASATENGQGRTLHVGTSIRF

Caulobacterales

Caulobacter crescentus

Locus:

>ACL95946.1 TonB-dependent receptor [Caulobacter vibrioides NA1000]

MGRKLWLHKVMGAASLLALATAGAAHGQVVEELVVTAQKRVENVQDVPIAISAFSANALQERAVGNV
AQLSNLAPNVTLDGGTPFSGSPSVLSAYVRGIGSDDFAFNIDPGVGIYVDGVYLARTVGANQDLLDVE
RIEILKGPQGTLFGRNTIGGAISIVTREPGDTFKFKGDVTTGSHKLMQARGSADLPLAEGLSSSITFGIK
SRQGYVERIPYPDARARNTTSYTAFPSSGYDTASHEGGENSWNLRGKLKWKGELTKVVLSADYTRE
DGGGLANTLLATTEMVPGNFAGTSNLPGTAFDPTGTTGFLFAGLYNFCIGATPAQIAARNAQALCGV
RGTQYSPGLRVASLAGVNVDADPTNDRLPFDSRFVSKDIDKSYATGNSFSNMVNWGVGGTVEHSLT
DDVTVKSITAYRKLLWKSGTDADGSPLNIGQLSFNMKQWQISQELQLLGSALDKKLNYVLGGYYFKE
KGSLHDYVTFDEGLLQVDGPNQLETKNYAAFGQVDWRPSELIGVTIGGRYTKEEKLFEGGQQDLNG
FNYKLFGCSDANGNIRPNGAFPLAPISCQVGLSYPDPSNPVRVYAPGVNKQTFSNFSPKVGVQLHPA
DDLMTYVSWSKGYKTGGWTTRLTNPQLTAQPFGEEVATTWEAGFKSKLLDRRLQLNGSVFQTEYD
GIQLNFQQGTSPTIRNAGDAKIKGVELEAVAVPLEGLTINASVGYLRARYTDVLAGVLAVSGPNVFQA
GTFVGAPLPKTPEWKINLSPRYAFKLANGGSVTAIADWTYTSSIWNNAQRTYVLKRPAVQVVNASVA
YADPDGAWSVTVGGTNLTDKRYLTTGNENIAAGAVFGTYSRPREAYVRLGVTF

tonB next to motor genes, where ExbB has a long disordered periplasmic domain.

>ACL95884.1 TonB1 protein [Caulobacter vibrioides NA1000]

MADQNTPEHRRYDPLTSEGPRRKKGFGAFGISLTVVAGLHALLFAYLAKQKFEAVFQEYSDDATVVE
LPPPPPPPPPPPPPPPPPTNEPPPPPAVVQPRPPVAPPPDVTPPPPLPIPPVEKRVEQTAPPVISPTPP
VPSNPPARASVITRPDWARKPSGEDMARYYPDRAQRMEVSGRATISCTVTAKGTLEGCSVVSESPA
DMGFGDAAIRLSRLFRMKPKTLDGAPVDGGQVTVPLVFQVPQ

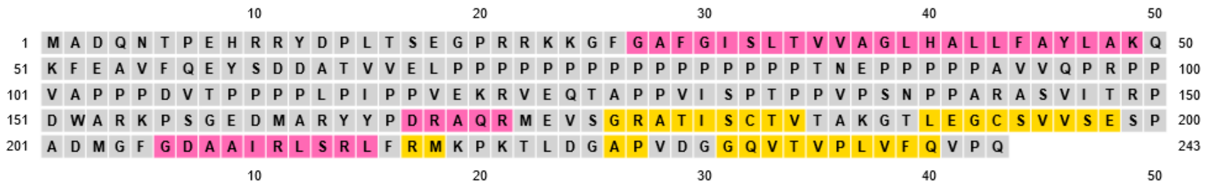

MADQNTPEHRRYDPLTSEGPRRKKGFGAFGISLTVVAGLHALLFAYLAK
QKFEAVFQEYSDDATVVELPPPPPPPPPPPPPPPPTNEPPPPPAVVQPRPPVAPPPDVTPPPPLPIPPVEKRVEQTAPPVISPTPPVPSNPPAR
ASVITRPDWARKPSGEDMARYYPDRAQRMEVSGRATISCTVTAKGTLEGCSVVSESPADMGFGDAAIRLSRLFRMKPKTLDGAPVDGGQVTVPLVFQVPQ

PPIIPRED:

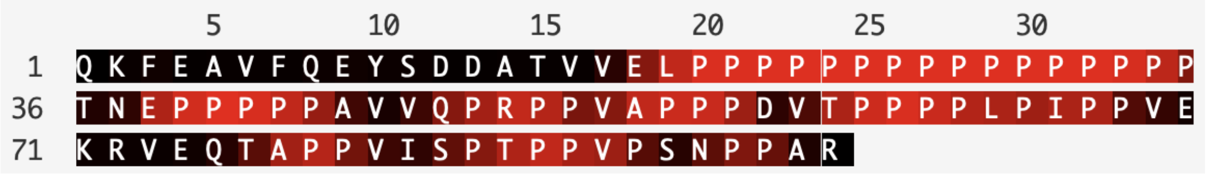

94 Domain II residues

42 Proline residues

0 α-Helix residues

Parvularculales

Parvularcula bermudensis

Locus:

>ADM08760.1 TonB-dependent receptor [Parvularcula bermudensis HTCC2503]

MRSKLHARLLSGAAVLALGAPAFAQDAEPALQDVIIVTGTRIDPATETGITPDAAPLQGGDITYLTARTP
GGARLGNGELSGQMQYRGLFGERLNLRVDGQRFASGGPNLMDPVFHYAPAPLVAAIVIDRGVSPVS
AGPGLAGGADAVFKRVDYAQGDEARFGYDLTVGARSVNDSLSTGGVVGLASDAWRVNLLGAWEKG
DDTEFGDGEIGGTAFERGVYGLSAGARTALGEFTLDLRRQNTGPSGTPPFPMDIQYFDTDFARLGYA
KAFGDLHFTASVHATDIVHLMDNFSLRPAPMPGRQRATFADASTQGGEGSISFPAFGGDLALGLDGE
QVEHDVTITNPTNTDFFVTPFPRVEMQRFGGFAEWSGALGAINAQLGLRADRNGYDAGEASLGSAL
PMGPRMLATAFNDADRSGEDTTVDAVARLWTTERGGLSWRVTLAHKQQVPGYIQRFGWLPINASG
GLADGNIYVGDLALEPETAWIAEAGYDYASARAYLRPTVFIRQIDDYIQGVPFDGTVGVADSPVEMIAT
MNGDPTPLRWANVEARLYGLDMDGGYDFDGPLRLDGVFSYVRGERRDIDDNLYRVAPLNLTLGLTW
EADVWSATVETRAVADQEEVSLTNSEEATDGYVVLSAYGEWDLYDGVTLSAGVENLLDEVYQDHLA
GYNRNGFGDVPVGDRLPGAGRGMFVRLNMSY

>ADM09361.1 hypothetical tonB2 protein [Parvularcula bermudensis HTCC2503]

MASLLRGFVGLPLAAIVTVFLFLFMWGLIRVNELPPAEDRQQANISFTRQIQDTEIRNQKVFERPSLDQ
PPPPPPAINNATFQPSVEGVRAQAPSFDADVDIGSGFNPDRDAQPIVRIPPTNWERCIDDRQAGTTQ
RVSLEFDVTPEGQVTNVNVLDSTDRCYERYATRAAERWKYNPKIVDGEAQPRFGVRTVIEFQIGSE

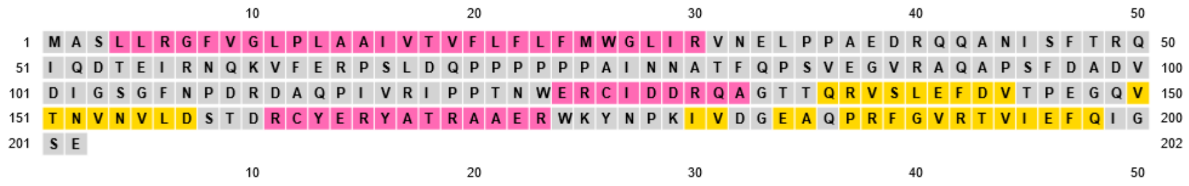

MASLLRGFVGLPLAAIVTVFLFLFMWGLIR
**VNELPPAEDRQQANISFTRQIQDTEIRNQKVFERPSLDQPPPPPPAINNATFQPSVEGVRAQAPSFD
ADVDI**
GSGFNPDRDAQPIVRIPPTNWERCIDDRQAGTTQRVSLEFDVTPEGQVTNVNVLDSTDRCYERYATR
AAERWKYNPKIVDGEAQPRFGVRTVIEFQIGSE

PPIIPRED:

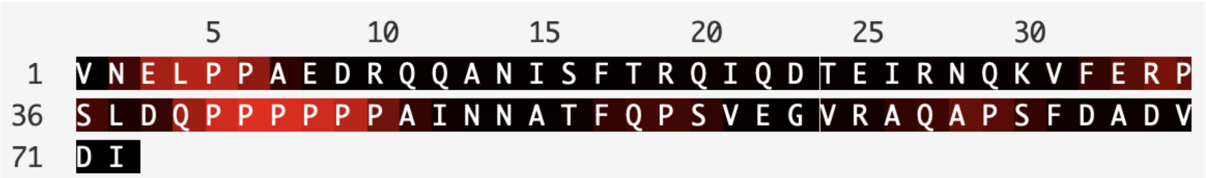

72 Domain II residues

11 Proline residues

0 α-Helix residues

Parvibaculum lavamentivorans

Locus:

>ABS61808.1 TonB-dependent hemoglobin/transferrin/lactoferrin family receptor [Parvibaculum lavamentivorans DS-1]

MATINTRRLPLLSLLFATTSLAAISVAQAQDTTGTDTGEDAPKATQLDAVTSTATRNPTAINDVAGTVS
VITAEELERRNANSMQDIVRYEPGVSVSNSPTRVGAGNFTIRGIGGNRVLVLVDGFTAPDYIDASAGG
LYTRDFFDLDSMKRVEIIRGPASALYGSDGLGGVVSYVTKDPSDYLDIFDKDWYASLKSGYDSSDESF
TESATGAVRAGNVEMLGLYTRRDGHEVEPNSSYVANPIDYESNSFLAKLVFNGPDSNKLTFTGQLDN
KQADIRLLSEETGTVNRSEATDETDRYFVSAAYSHRMPTPIADRMELKIGYTKLDRVEHTEQLRSGST
VLRLSDLHFNQEILSGDFQFGLTREWAGFTHDFTYGVTADYTETSRPRYRTETNLITNTTTNVVGFET
FPNKNFPDTETIKAGVYVQDDIGIGRLNIIPALRFDYYSLDTKIDRMFLNSAGTNVPVDMTETEISPKLG
LTFALTEEYKIFAQYAHGFRAPPYDSANYAWQHTTFGYEIIPNPNLKPETSDGFEAGLRGKFRDGSSF
SVAGFYNTYEDFIDQVLLGVGGVPPLMQFQYQNLAEVTIYGAEAKGEYRFLPEWALIGSLAYARGED
DQTGDPIDSVDPLKGVLGLAYEGDSWGSQLVATHAWRDTRNATPGNFEAPSYTLVDLMAYYDMSE
HLTVNAGIFNIFDEEYYFAQDVAGLASGSPLTGRYAQPGRNFGINATVRF

tonB not near motor genes, next to heme uptake TBDT. Likely specific to that TBDT.

>ABS61807.1 TonB family protein [Parvibaculum lavamentivorans DS-1]

MSMTATANGRMTDESDGSSVLVWNHRALRRPVAVSLLLHAAIFTGALAWWQGEGAPAPKGHPDGF
SVTLVNFSEPAPKAPQIQAKPVVAPAQPEVKPQPKPVVKPAVVERKVEPEPQITPDVTPEISAAAAEPL
QAAEAAAQPVQTASLAPSVSDGRGQGAAKGFEDTQGRESDKVFLTEPRFRSPPRPPVYPRRARDLE
QEGEALIRVRLDPSGNAAEVLVWKSSGFALLDKAALTAVRGWQFEPARRGGKPVVAWVQIPVRFAL
N

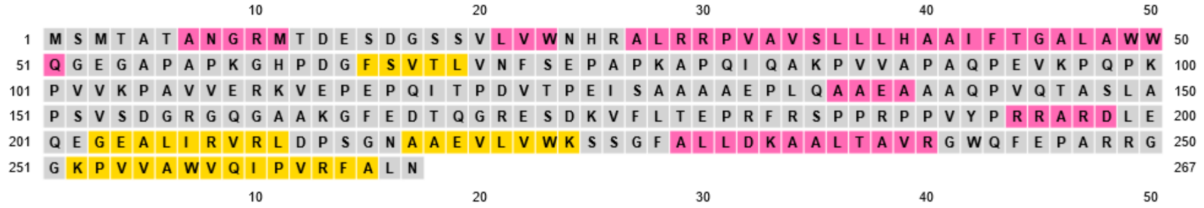

MSMTATANGRMTDESDGSSVLVWNHRALRRPVAVSLLLHAAIFTGALAWWQ
**GEGAPAPKGHPDGFSVTLVNFSEPAPKAPQIQAKPVVAPAQPEVKPQPKPVVKPAVVERKVEPEP
QITPDVTPEISAAAAEPLQAAEAAAQPVQTASLAPSVSDGRGQGAAKGFED**
TQGRESDKVFLTEPRFRSPPRPPVYPRRARDLEQEGEALIRVRLDPSGNAAEVLVWKSSGFALLDKA
ALTAVRGWQFEPARRGGKPVVAWVQIPVRFALN

PPIIPRED:

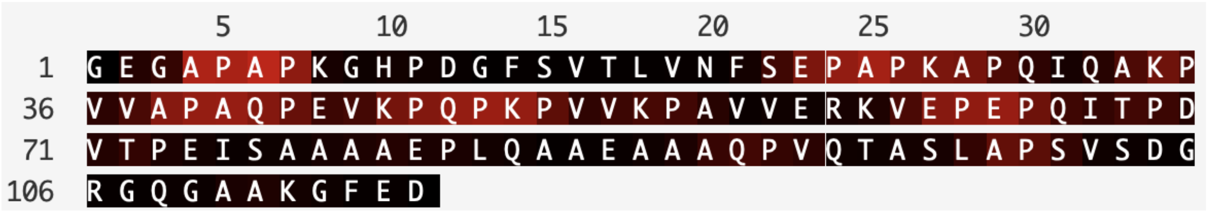

116 Domain II residues

20 Proline residues

4 α-Helix residues

Rhizobiales

Hyphomicrobium denitrificans

Locus:

>AGK56356.1 TonB-dependent receptor [Hyphomicrobium denitrificans 1NES1]

MKAARKQGVKLMAGRILAAAWGLTLAFAVVGPASALAQESGGATTAQPPAAPNAPSPNTPSEQTAPTQAPSTETPNQQSTTPG
TENPSTVLPEVQVIQQQQKAKPKPVKQAVKPKKAPTPVAAAPAPPPSPAAIEAAQPSEIKMSPVAGSEIPLAKVPIGVSSVTAADV
QRTGDVTVQNVLETRVPGIIVNDMQGNEFQTEVQYRGFSSSPVNGVPQGLAVYQNGVRINEAFGDIVNWDFLPSNAISNMTVM
SSNPVFGLNAIGGAISIDMRDGFNFHGAEFDVRGGSFGRIQGSGAIGAQSGNWAMFIAAEDINDDGWRQFSPAEIHRMYADLGF
KNRDAEFHVNFTGADNFVGAVTAAPVQLLDEGWNRVYTNPQTTQNDMEMVSINGSVKATNTLTFSGVTYYRHFQQAHLDGNIL
EVEDCGDGNLCLDGDPLTTSGGAEIPSSVGGGAPLGSLDRISQNTDSFGGSLQAVERAKLFDHNNQFLLGGSIDHGHVIYGANS
ALGAIGPNFTISPLGIPLTGPADISPRSLTTTNDYYGLYFTDTFDVNDRLSLTVGGRYNFARVTLEDKTGEDPFLNGSHTYQRFNP
MGGATYELAKGLTFYTSYAEANRAPTPAELACADPDNPCIIESFLTGDPPLKQVVSRTGEVGFRGESLNALTNEKINWSFGLFRT
LNQDDIISVASPTSGRGFFQNAGDTLRQGIEASISYQTQRLFLYANYAFIDPTFQSSLELASPNNPSPDVVPCAGDPTEKCINVRP
GDRLPGVPRHRFKAGFDYWLTPKWKFGSDLIASSDQVFFGDEANLNKPLAGYAQVNLHTSYDITDHIQIYGLINNLFDNRYGTFG
NYFNTAEGTEASLGTIDFIEGTEASLGTIDFTDARTIVPSMPFAAYGGMKFKF

tonB next to motor.

>AGK56470.1 TonB family protein [Hyphomicrobium denitrificans 1NES1]

MIAVVLSVLIHGSIGYAMLPALRDANSEALDMGKGTDIVLVEQGIAEEGISKLGDAMETIETAEIKPVQA
APPPPPEQVKPDELRDVIASEESTVEQEVVKTDDPPPIKEPPPPEAVQAQSQPEQIAVVTQQSSGEE
KSGGNAKMLGLYLGQVNDHVQRAKVNPRSAIAGTVIVRFTIDVDGKLLSKEVAKSSGSKVLDDAATAA
LDRAAPFPPIPPEVSIKPLAFTQPFKFVMR

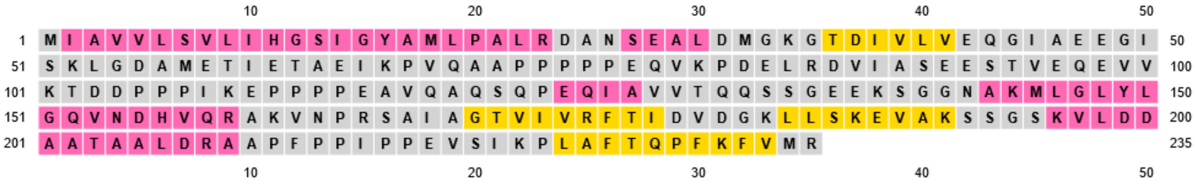

MIAVVLSVLIHGSIGYAMLPALR
**DANSEALDMGKGTDIVLVEQGIAEEGISKLGDAMETIETAEIKPVQAAPPPPPEQVKPDELRDVIASE
ESTVEQEVVKTDDPPPIKEPPPPEAVQAQSQPEQIAVVTQQSSG**
EEKSGGNAKMLGLYLGQVNDHVQRAKVNPRSAIAGTVIVRFTIDVDGKLLSKEVAKSSGSKVLDDAAT
AALDRAAPFPPIPPEVSIKPLAFTQPFKFVMR

PPIIPRED:

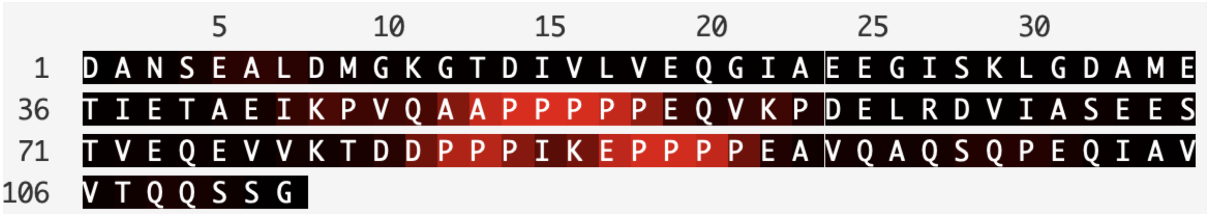

112 Domain II residues

15 Proline residues

8 α-Helix residues

Bartonella clarridgeiae

Locus:

tonB next to hemin-related genes. Not next to motor genes.

>CBI76097.1 TonB protein [Bartonella clarridgeiae 73]

MKGASIWRISALWIGAFGGALALHIGIGAQLYFKSIYMNDIVFPPMVMLLVEPEIMHPNVNADSEILELG
VLKQEEILKSELSKVHPEEHEIAEELESIVEKSNFTVLKPLEKSSPSKINRKVFIQKQLSTLNVTAKKINV
KKAHSFTASRDNNTALFDNALSGQWLAKVQAQLEKQKNYIVGQRISSVQGVVQLEFKVSEQGDIFAS
RIMLSSGNQELDWLAMMTLKRVDIFPPPPREMVDKTIRVSLIFS

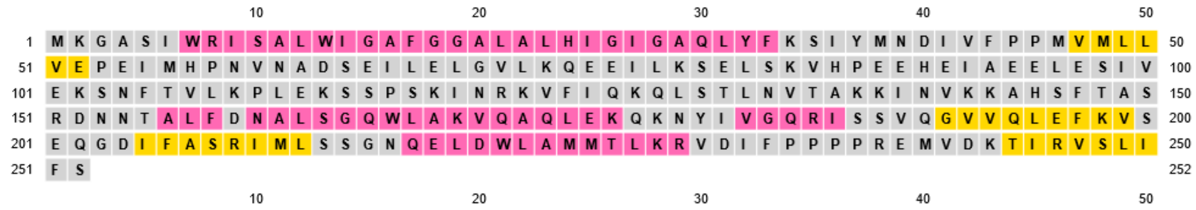

MKGASIWRISALWIGAFGGALALHIGIGAQLYF
**KSIYMNDIVFPPMVMLLVEPEIMHPNVNADSEILELGVLKQEEILKSELSKVHPEEHEIAEELESIVEK
SNFTVLKPLEKSSPSKINRKVFIQKQLSTLNVTAKKINVKKAHSFTASRD**
NNTALFDNALSGQWLAKVQAQLEKQKNYIVGQRISSVQGVVQLEFKVSEQGDIFASRIMLSSGNQEL
DWLAMMTLKRVDIFPPPPREMVDKTIRVSLIFS

PPIIPRED:

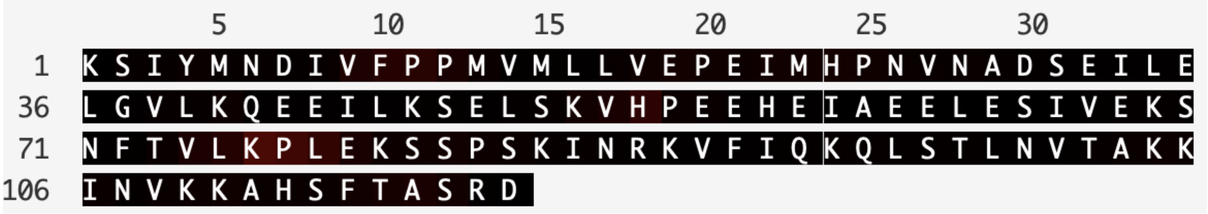

119 Domain II residues

7 Proline residues

0 α-Helix residues

TBDT detected by BLAST in many other strains.

>WP_026501097.1 TonB-dependent hemoglobin/transferrin/lactoferrin family receptor [Bartonella clarridgeiae]

MRIKRNVYKRYVTLSMLLSVYMPSFVFAQNNDKDFITKLKPIVIEKKIENPLNKATVLTDRETVQNIDKKQINDIYDVNHLNPSINYS
TDNNSFIIRGLNSNRVLTTMDGIPLPWLNDGVRGIKGGSSAFTFNSLSTIDIVRGSDSSIHGSGALGGIIALRTLNPEDLLTKEKNW
GALTKGSYNSVDKSWHVDQAFAVRANQILLLFQGSRTEGHQRKNMGTVESYGDKRTRENPADFDKNNLLFKVHQYLNDNHRL
GFTAERFAYNKETHSLNTSTEIFLPKSVYNEKHTHRKRFSVSYDYNGNGDATFDAFHGQFYWQKQSNNHTLNGYRIQAPKGDY
LRDNFMRNTGYGFNAHSFKKLDIGSVNHKLKFATNVFASKFHQYASGKDNCHLRGNERGCVFLGTNKSDAPDTNSHGFGLAFE
DEIGFSNNDIRFIPGIRYDWYKHIPQKTPAFEKALGSRIMPKENSSSHFSPKIRMEWDANNQVLFYTQWAQAFRAPSVSELYLTFI
KPSFYYMIGNPNLKPETSNGYDIGLQYKNMHFNGSFSVFTNEYKNFIDVIDKGPSQEFIYARKHYINRAHVRVSGVETKAHFTLM
NGFYSNVSLAYVEGKDLDKDEYLNSIPAFKTVFGLGYEKEIWGTDVVLTLAAKRDKVEGDSDYAKVPGYGVVDILGWWKPFGEK
GPVIRAGIYNLFDQKYWNATDLPSPSPIPKRQPLPPKDYFSQPGRNFKVSYVQKF

Rhizobium etli

Locus:

>ACE91783.1 putative exported protein, TonB-dependent receptor [Rhizobium etli CIAT 652]

MALAFAVPGFGLPAMADPVQRPTPVAGSVIARKTGEEVRFIDVSDWRVVDINQDLLTGDVLRTNANGQLAIVFSDHTQVRLGRNSSLQVKKMAASG
DTVLELQAGTIWARAERGGQGLTVETPAAAAAIRGTDWTMTVKGDQTSMIVLEGRVSLSNPQGSVDVSEGEGAVATIGQAPHKLISVNPDDREQM
LFYLDLRDGFGLMPTSPLRADRMATERRRLLALPLEHRSIEDWLELAEVQSAFDGRQAAAASLQNIRGRKLTVAQQARVDLLDATIAGSEKRYGDA
AKLFQKALPHLDPTRRNMAQYGGYFARSLADPAHSEPPPATTTGPYGAIMQAYTAGFLENPRAALEIIKKAERRYPDDPTLPAVRAQLAQLIDDRQQ
MKEAIERSLALDPDHPMALSARAEYKAAYESDIDGALADLNHAIELAPGDSGALNSLGLLQSSRDANGEAEKAFKRAIELDPQDPLLHANLAMLYLD
QSRMKEAKREIDTAIALDPSFDIALLVRGRYYLQSGERDKSLEDLLAASTANPAHSQSQLMLAAAHYEKGDRIPSQQALDNADRLDNNDPAISAFRT
AVDIDDYDADGAIHNAQEYLRRSRARGGDYSSLGANASAGSTLNNAFRLQGLDAWGRYYGDAVFDPFKGTGYIDQSIKGSIFPFVNATSFSDDSVS
QYSVNASTYSSFIQGLLLSPHMLSGRSRSASLFDVPFIEGSLGGGINSVDGHTRRIGEAEIQGYSNETIPISFRADMTWEELALDRDYRDFGGVQTD
NKLLSADGYLTATVTPDDRVVAFVNHGKNDGALSALSSNTGFMELLFGVPFPLPLYTTEETARESTYAGVGWSHTFAYENVVNGALLYSGSKSRTS
DALDVDLDPLFIRAGVPFIIPFTDVTQETESKTYIGALSHSIGAGPLTFRYGVEGGWMDVSSTVDKTLLGVTAPPDHSESTIDIGRAYVDVLHEITPDLK
GEYALFATHLEGGGTDITRLEPRFGLAWAPVQNHWLRAAFIRQSFDTGIPTLAPIGILGLQANQFSADPQGYTDTIALQWDAEWTDRFFTSVEYQHQ
ELHDFSVDFPLISLPSDTSLPISRGSIDRAAVTANVALGNGFGLSATYAYMDSENRDPLEPIYGGPLPFIPQNSGQIALTWVNEAKVKATIAANYIGER
DGDRFGTKLDDYWSLDAHLVWEPFDKRMELEAAAYNLLDEDFEITPGVPGWGRAFKGTLKVRF

-looks like CirA with TPR fusions.

tonB on different locus.

>ACE92456.1 putative membrane protein, transduces energy to outer membrane receptors [Rhizobium etli CIAT 652]

MAISAKTRSRQVLIGEADAGGSLNDNTPGMHPGHELSELRNAPRQPAGETVIHYTRFAQISSFPDHPETVPAAPVPAPPMDAAV
EKQEDEQKPVRRRVALSCAGSFFFHAALAATLFAVMPAPSDETLMEAGEAISVVMLGSSDADQSAAGETEVTIQEEVVPEAVEP
DTVKPVETAEVQPEVVQPETVQPANAEPVEIVQPLQDVTRQSAETVATAEPEVLVSETSAETSVAQPMSTVVPEQLVISSEPPTA
AAIQSESEEIKPVEAAPVSPEPEPPTEVVIPQKKPKLEKPVEKKVVDRKQPPKKTQGSEGDAKQESRRGAADGQADANSDNNSR
TSGGKEGAGGASQANYTGKVFSRLSRCIDRLKSQYRDTAVSLRVRVMVNSDGNVTLSRITSGSGMADVDNAVLGSLNSCNLPA
LPDGWGASHTFDFPVQVTAR

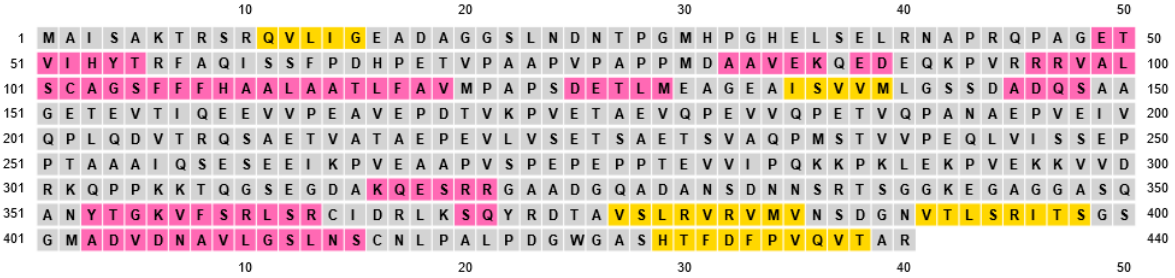

MAISAKTRSRQVLIGEADAGGSLNDNTPGMHPGHELSELRNAPRQPAGETVIHYTRFAQISSFPDHPETVPAAPVPAPPMDAAV
EKQEDEQKPVRRRVALSCAGSFFFHAALAATLFAV
**MPAPSDETLMEAGEAISVVMLGSSDADQSAAGETEVTIQEEVVPEAVEPDTVKPVETAEVQPEVVQPETVQPANAEPVEIVQP
LQDVTRQSAETVATAEPEVLVSETSAETSVAQPMSTVVPEQLVISSEPPTAAAIQSESEEIKPVEAAPVSPEPEPPTEVVIPQKK
PKLEKPVEKKVVDRKQPPKKTQGSEGDAKQESRRGAADGQADANSDNNSRTSG**
GKEGAGGASQANYTGKVFSRLSRCIDRLKSQYRDTAVSLRVRVMVNSDGNVTLSRITSGSGMADVDNAVLGSLNSCNLPALPD
GWGASHTFDFPVQVTAR

Confirmed TMH by TMHMM

PPIIPRED:

221 Domain II residues

26 Proline residues

14 α-Helix residues

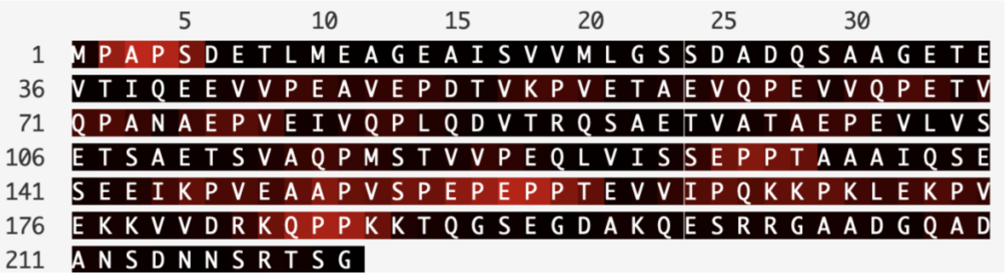

Nitrobacter winogradskyi

Locus:

>ABA03991.1 TonB-dependent receptor protein [Nitrobacter winogradskyi Nb-255]

MAGRSVSGVAAIRMARLLASISFAALLLPSQAAFAQTAPSSAPSAGALQQSITFAIPPQSLSSGIVAFS
RAAGIDLVFDGAVPRGARTSGVSGTFTVRDGVNRLLAGTGLSARFTNARTVQIVNPSAAGGVSGAAP
SGAISLDTIDVQGETAWGPANGFVASRSATATKTDTPIIEVPQSISVVTRDQIEIRRNQTLNETLQYTPG
VFAESSGMQHSGPTFQIRGFSAVPADGPIFLNGLRTINNGDIEPFGLERIEVMRGPASVLYGQNQPSG
VIALVTKRPTEGPVHEVQLRGGSFGQKSGAFDFGGPVTEDRTLLYRFTGLLREGGNGIDFSNDQRAF
VSGAVTWRPTDATELTAFSLFQKDKGRWNYGLPAQGTALPNPNGRIPVTRFIGEPDLDYNVTERAVV
GYNLQHRATESLTFRQNLQYARENWDYRNATPIGLQADLRTLDRSYETLRASWDTFAIDNQAELKTT
TGVLHHTVLFGVDYRWRRYNSLGLWGGVASPIDVFAPVYGQPVLISADNYGSRQTQNFPGIYLQDQI
KLDRWILTFGGRHDWAESKTVNAFSAAVTNQNDRAFTRRAGLGYEFDSGVVPYVSYSESFMPVSGT
TFDQVPFRPKPERSTRQASNTSRMI

tonB detected near motor genes with large periplasmic domain in ExbB. Seems to have a large disordered region.

>ABA03971.1 TonB protein [Nitrobacter winogradskyi Nb-255]

MMKIDWRDPDGRAGDDVNRAAALRWTAAGLAVVALHAGGIWMALHWPAAAEAPGDPPAAIMMELA
PMAVAPEAPQQDVAPGPEMVEAEEQVDPDKPIEEKPDPEPTPPEVKEAEVKLPETPKVEKAEVVLPA
KVEPPKPKPKKKKQKKAPRTTAPQSSQAQRADRAAAPAEGMASSMSPATWRAALMAHLNRHKRFP
PGAGMGVASVAFTIDRSGRVLSARLVRSAGDSSLDAEAVSLPRRASPVPAPPSNVGGGSITLAVPIRF
NR

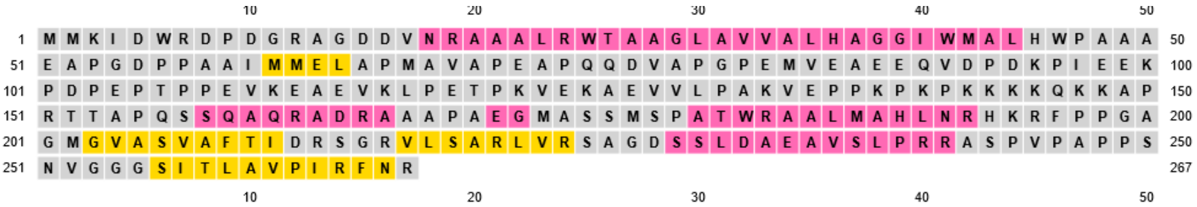

MMKIDWRDPDGRAGDDVNRAAALRWTAAGLAVVALHAGGIWMAL
**HWPAAAEAPGDPPAAIMMELAPMAVAPEAPQQDVAPGPEMVEAEEQVDPDKPIEEKPDPEPTPPE
VKEAEVKLPETPKVEKAEVVLPAKVEPPKPKPKKKKQKKAPRTTAPQSSQAQRADRAA**
APAEGMASSMSPATWRAALMAHLNRHKRFPPGAGMGVASVAFTIDRSGRVLSARLVRSAGDSSLD
AEAVSLPRRASPVPAPPSNVGGGSITLAVPIRFNR

PPIIPRED:

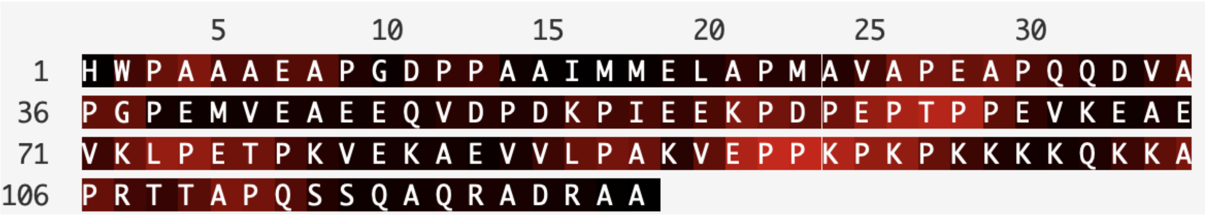

123 Domain II residues

25 Proline residues

9 α-Helix residues

Methylobacterium extorquens

Locus:

>SOR31005.1 putative TonB-dependent siderophore receptor (putative ferrichrome outer membrane transporter) [Methylorubrum extorquens]

MGSVRTLVGVRALRTMALTGASLMALTLAAAAQQATARLEELSVEGSGRGAGATGGPPAQRGADGRAAPEDPRGPVAGYVAT
RSVTATKTNTPLIETPQAITVIGREQIEAQGAQTLTQATQYTAGIYSGVYGADERVDFFTLRGFVASDYGIYKDGLQLLNYGFGTL
KTETFGLERIEVLRGPAAVLFGAGNPGGLVNQVTKRPTLAPFGYVEIAGGSFGQVYGAFDVGGPADDSGHLFYRLTGIGRQGGT
QVEGADSDRAYIAPAFTWRPDAGTTFTVLTSYQRDSTAVTANFLPYAGTVRPNASGLRIDRSLNVGDPRINSFQREQVFAGYEF
EHAVDDVWTVRQNFRYSFSDATQNSYIGQLGYADLPQTQLARYQFQYSDKVSLFQVDNQAEARFSDGLVAHDVLFGIDYKNYS
LYDNQASLFPAPNLSIINPLYGQIVGRPLPYQVDVNSFRQLGFYAQDQIKLTDRLSLIGGLRYDIANSDVLRKLAPDTSTSRTDTAL
TGRIALLYNFDPGIAPYVAYSTSFQPQIGVDAITGGSLSPDRGEQIEVGLKIEPVGERFSLNIAAFDLVRDNPPFPVIVGFGSTQLG
TVRSRGIEGQFVASLAEGLNVVAAVTHYDLEYTRVRDAALGDLVGKTPTNVPETFASVFADYTIPLGDWRGFGFGGGVRYVGR
SYADQLNNLKVPDYVLFDATVHYNWERWRMAITAANLGDRRFVTSCQSANSCFYGEARRVLASVSYRW

tonB next to motor pseudogenes.

>SOR30983.1 putative TonB family protein [Methylorubrum extorquens]

MPPMQAPTSAPMPAPVPAYAGQGLPSGPSEGGGQGRLAAAFALALALHAAGLIGITYLHLTPPAPPG
EQEITIDLAPQMAEAETQAPAQTAQSEAIPEEAKPEGEPETAEPVETPDEVKPPPPPEMTEVMPEEVQ
PPPPPPEAVTEVPPDTLPPPPEEQIIASEAQEAEPLAPPPPVVAKVPERPKPDPKIEERRKAALEKKRE
AEREARRQEILEKKREEAQKEARIKAAKAKAERDAARRAQAAQAGNAQRNSAATSRQSATGTAAAA
SDPNAMAAWKGSIAATIRGRMNREAAAGTSGGVATVRFTVSRSGAVSGAAVTGSSGVGAIDSAALA
AVRGGLPPAPAGVTQPSLAVTVPLRFSPGR

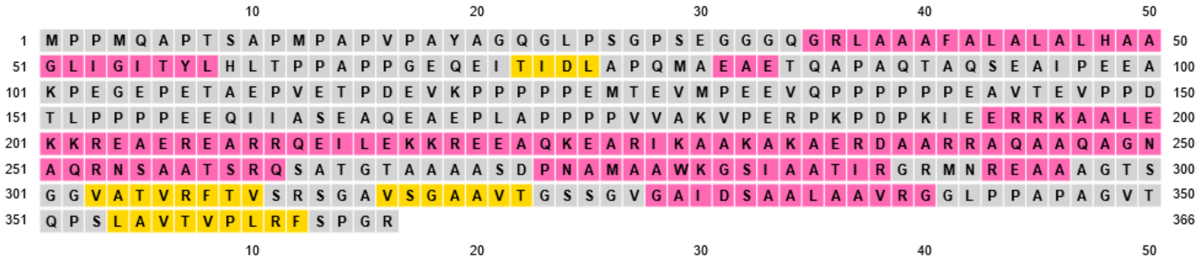

MPPMQAPTSAPMPAPVPAYAGQGLPSGPSEGGGQGRLAAAFALALALHAAGLIGITYL
**HLTPPAPPGEQEITIDLAPQMAEAETQAPAQTAQSEAIPEEAKPEGEPETAEPVETPDEVKPPPPPE
MTEVMPEEVQPPPPPPEAVTEVPPDTLPPPPEEQIIASEAQEAEPLAPPPPVVAKVPERPKPDPKIE
ERRKAALEKKREAEREARRQEILEKKREEAQKEARIKAAKAKAERDAARRAQAAQAGNAQRNSA
ATSRQSATGT**
AAAASDPNAMAAWKGSIAATIRGRMNREAAAGTSGGVATVRFTVSRSGAVSGAAVTGSSGVGAIDS
AALAAVRGGLPPAPAGVTQPSLAVTVPLRFSPGR

PPIIPRED:

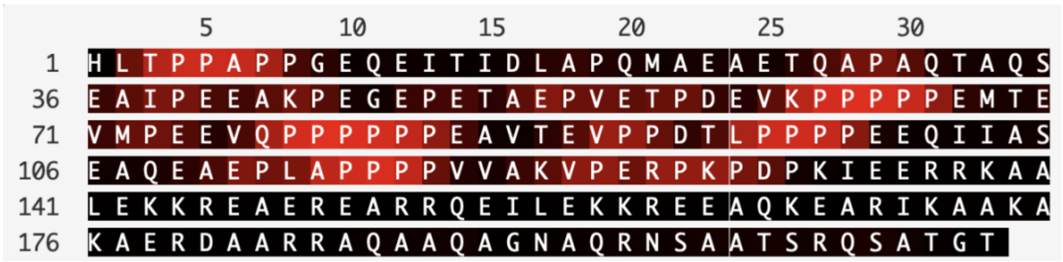

208 Domain II residues

38 Proline residues

72 α-Helix residues

Beijerinckia indica

Locus:

>ACB93894.1 TonB-dependent siderophore receptor [Beijerinckia indica subsp. indica ATCC 9039]

MKRDEFEIFEGSAANTPATELYIKAKARPPLKNAVVGLASLMLGGGAATAQESTNLALEQIEVEAGNA
SNNGSAYNPRSTGLDRLPEPILDTAQSINVIPQQLLRDQQTTTMVEALRNVPGISINSAEGGTQGDNIN
IRGFSARNDIYRDGIREPAWYSRDSFSVENIDVYKGPSSFIFGRGSTGGVINVVSKLPVFTNFTDFSLS
GNTAPGVRSTIDVNRTFGDIAARIIILGTDWDTAGRNFIGTKRVGAAPSIAFRFTEQTKLTLSYIYQHDD
NVPDYGIPIVPGSIFGTSYGKPAPVSRGTYYGQQTPGFNDTEKVDAHIVTAKLEHEFNKDWMLTNGT
RFSNVDRFLRARAPQWVASTSAIYAQPVGGTALTSLAGVPIGSLYSIDNNHFQNHTINSMISNLTNVV
GHEETWGLKHTIQMGLDLSKEDRYNARQATNNTTAATTNQRINLLYPDPYPFGNNFGPNTTLTRTEA
RDIGMYVSDQVKFNDYFELKGGFRYDFYKAWQFTANSSRADGSLSQNGTTPFNLSSTNNFASWNG
SALFHPAPNATLYYSFGTSVNPLAEYTTITNGGQNLGATTNESHEVGAKIDLFDSRLSLTGAAFMITQN
NAVETIDATTSPPTVALVGKTRVKGIEVGATGEVTPGWNVFAGYTYLDGRLLQSQQYANFVGNVVQN
VPRNQASFSTTYQVTPDWKIGTSVYFVDSRWTNVQHAGWVPSYWRWDLMTSYQVTENFSLQANLF
NVLNTTNYESIAGAGFAVPGVGRSLQLTARLHW

tonB next to motor genes, where ExbB has a long disordered periplasmic domain.

>ACB93889.1 TonB family protein [Beijerinckia indica subsp. indica ATCC 9039]

MSFISDRPFLPPEEEGLDTKKWVLAGTLVLLAHAGLVFWLTRPHEDDLAAGQLEPAVMIDLGPEAMA
PPVDTPAEVAPQQMTEADPEENKEKPDPDPVPDQPVPPPELPPLPETPVVPKAEVVMPAKPVVKKP
PPKKKDVKKEHAPKTTAPVQTNARASNAAASAATGAGGSSAAANDWKSRVFSQLARNKRYPDSER
QNGVQGVVGLAFTIDRSGNVLSAHVSRSSGSSALDNAAVEMVHRASPLPAPPDDIGGSRITLSAPVR
FSVH

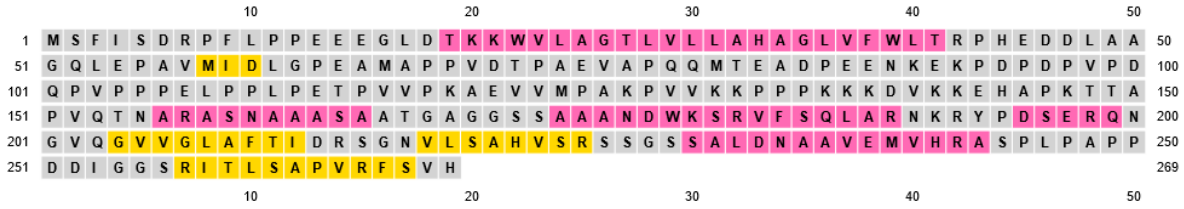

MSFISDRPFLPPEEEGLDTKKWVLAGTLVLLAHAGLVFWLT
**RPHEDDLAAGQLEPAVMIDLGPEAMAPPVDTPAEVAPQQMTEADPEENKEKPDPDPVPDQPVPPP
ELPPLPETPVVPKAEVVMPAKPVVKKPPPKKKDVKKEHAPKTTAPVQTNARASNAAASAATGA**
GGSSAAANDWKSRVFSQLARNKRYPDSERQNGVQGVVGLAFTIDRSGNVLSAHVSRSSGSSALDN
AAVEMVHRASPLPAPPDDIGGSRITLSAPVRFSVH

PPIIPRED:

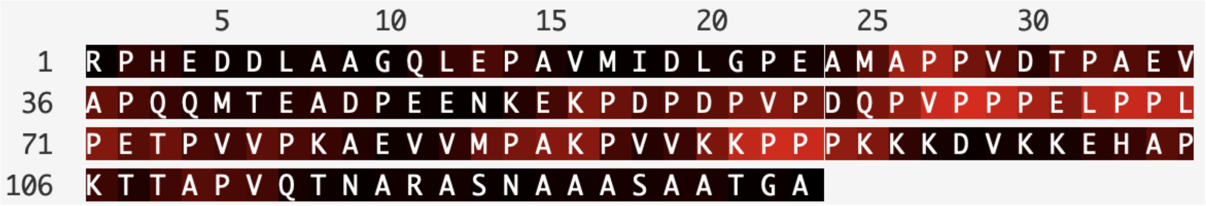

128 Domain II residues

28 Proline residues

10 helix residues

Xanthobacter autotrophicus

Locus:

>TLX43811.1 TonB-dependent hemoglobin/transferrin/lactoferrin family receptor [Xanthobacter autotrophicus]

MALRPQRAGVCIWTRRILCSGVAACALLSGMAGVARAQSATAATAQSESDGEISLDTITVAASLTPER
TIDALAAISVVRPEDLEQLMPARTQDVFLGMPGTTVIQNGNSTQASINIRGMQDFGRVAVFVDGARQN
FTQLGHGTGAGSFFLEPGLLADVDVVRGPVSNIYGSGAIGGVVTMRTKDADDIIKKGQTWGVEASSD
FGSNGPMGFGALFAAARVGQNVDLFLGGTYRAQNDYKDGNGDVVPGTGYDTWTGIAKATFRPADF
HEVKFTGLNYNADYTTYNTALVNGALPSTATQYGSTVLNQTATASWNYTNPNDNVFDWRSTLYWNK
VKQDQIKVAGTASSITGAIGNPRYFTINTVGFDANNTSRFVWNDIRNAITIGGDYFHDDVDNIDDYGFG
EGYNPSGERGVGGAFVQWKANYSSWLEAQGAVRYDTYSLNGDGVSTDGQRFSPKFTLGITPWNW
LTFYGTYAEGYRAPAVTETLVNGAHPPNIPLVFCPDGSYGVFCFVPNPYLQPEVGKNKELGLNIRFDD
IFVKGDKFRGKANIYRNDVDDYIELVGYQMTRYGTYANYQYQNIAQARLQGFELESNYDAGAWFAGF
NVTVSDGENTETGGPLSNTMPNNVAGTLGARFLDRKLTVSVRWQWVAAVTEADMPADAPYEPTPS
FNLVNVYLGYQPSENVLASVSVENLLNEQYTQYQQFLPSAGLTVRGGLKIRFGADTIAAATPSPVFK

tonb next to motor genes with ExbB with long disordered periplasmic domain.

>TLX43694.1 TonB family protein [Xanthobacter autotrophicus]

MSAVHHWHDFDRTHVRDTVLRLAAGALFIVTVHAVAVYAALFWRKLEAGESAPPAAIMIELAPVAVSE
QQQVEDAAPGPQMVQAPESVPDAPDIPAEAAPPPEPVVNEVLEKLPEVEPSKEQAEVVLPKPEPKKE
EVKQPPKQKVDKPKKDAKPTRRPSAPTTTAAPRSDAPAAQATAAPSPGAAAMRSAALADWRSRAFA
HISRYKRSQGSNVGQPRVTISLSSSGSVTSARLLNASGNEVLDREAVALVYRASPFPAPPDNRPLTFS
VPINFTTR

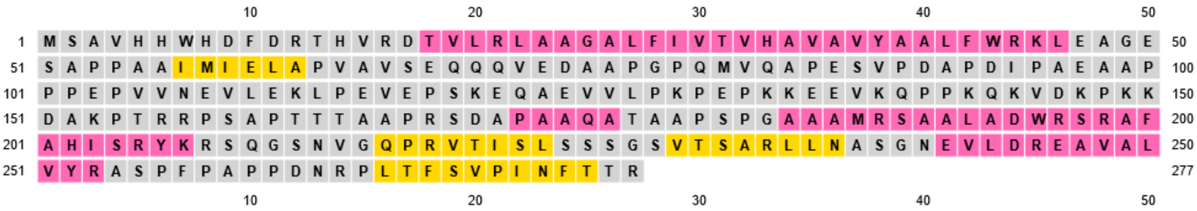

MSAVHHWHDFDRTHVRDTVLRLAAGALFIVTVHAVAVYAALFWRKL
EAGESAPPAAIMIELAPVAVSEQQQVEDAAPGPQMVQAPESVPDAPDIPAEAAPPPEPVVNEVLEK
LPEVEPSKEQAEVVLPKPEPKKEEVKQPPKQKVDKPKKDAKPTRRPSAPTTTAAPRSDAPAAQAT
AAPSPGAAAMRSAALADWRSRAFAHISRYKRSQGSNVGQPRVTISLSSSGSVTSARLLNASGNEVLD
REAVALVYRASPFPAPPDNRPLTFSVPINFTTR

PPIIPRED:

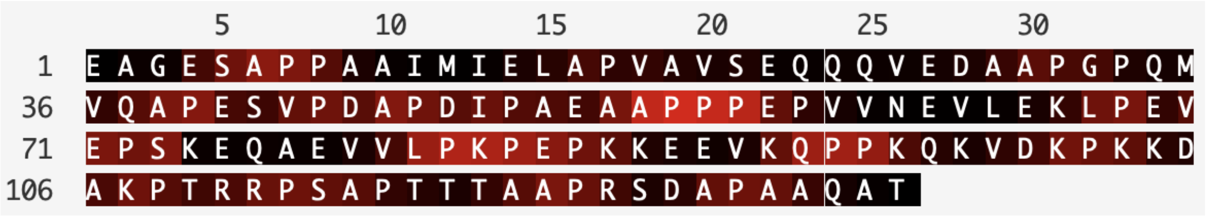

131 Domain II residues

26 Proline residues

5 α-Helix residues

δ-Proteobacteria

Syntrophobacterales

Syntrophobacter fumaroxidans

Locus:

>ABK16192.1 TonB-dependent receptor [Syntrophobacter fumaroxidans MPOB]

MFLLLLPGLLLPAVAVAADAAEKVAPAESRTGSSEIGYTAAEGAPAPGHADETAKKDHPLDPIVVTATRTEKSIVDVPAATSVVTR
EDIESRNIQKVDEAMNLVPGLFDKRAKGLDTTGRVTLRGLPDQKRTLVLLDGQPLNNGYTGHVDWNSINPEDVQQIEVARGPFS
SLYGGNAMGGVVNIITRMPEKREFTVKGGYGSDNYWSTYGSYGDKLFDRLRLLASFGYKSSDGYPSGLVVKKPSGSGGTPAR
GAVPTTDTYGNPAFIIGDTGDNSWWTQSGGLKLSYDVCEEAKVFFSYRNNQYGYGYDNPNSYLTNAMGDTIWSGPVNFRGNT
LSFTEGSFLSGGGGILQNLYNGGLETRLFGDSVLKLSGGLIDMPTNWYVTPSSSTATRSGGPGTISETPTRAYHADAQLSIPVLE
KHMLIVGTAFRHDEANNQEHALTDWTDTGSKADLTYEAKGADNIFSFYSQAEIALFRDVTLYAGLRGDYWETFDGMADQVGVA
GYPQYYDSKGEFSVNPKGSVVYKPLDGTTFRASVGTAFRPPNVYELYRTWVTSYGTVYQGNPKLDPESSFSWDIGVEQKIGQS
TVLKLTYFNNTIDDFIYFRTVTPTLLVQTNAGKAETDGFELEAETRPWECLKLFTSFTYTHSEMLDNPANPLTEGKQLPGVPRYMF
SAGGEVKYRRLSFTAIGRYVSKQYSSDQNLDTVSGVYGSYDAYFVGEFAARYKLTDWATIDFAVNNIFDNNYYSYYKAPGRQFF
GGVTARF

tonB not near motor.

>ABK16191.1 TonB family protein [Syntrophobacter fumaroxidans MPOB]

MRLWQNLTISILGHLGVACVLWATPLAIYRPPPWMEVRLISPADFHGRAEIPCDGNGAGEGGPAADD
NIHAAPKEVCAVKQLPPPEQRPEEPKLEPPVRNRSVKKEKPPAKPKGASRVVPLAAIRADAQRPADP
AESSAGEGAAAAGSHASIGPGTFASGTPSGGSVDAGAGAGSLSPGGPGEGVVDARFGAANGPRFH
RLVKPRYPSVARQLGKEGTVVLRVTIDEKGRAIHVEVLTRAGSGFEEEAILAVRESTFTPARLNGHAV
KSRAVLPIRFALTNSG

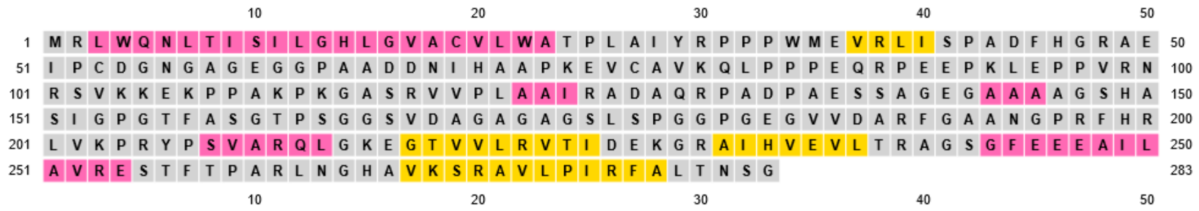

MRLWQNLTISILGHLGVACVLWA
**TPLAIYRPPPWMEVRLISPADFHGRAEIPCDGNGAGEGGPAADDNIHAAPKEVCAVKQLPPPEQRP
EEPKLEPPVRNRSVKKEKPPAKPKGASRVVPLAAIRADAQRPADPAESSAGEGAAAAGSHASIGP
GTFASGTPSGGSVDAGAGAGSLSPGGPGE**
GVVDARFGAANGPRFHRLVKPRYPSVARQLGKEGTVVLRVTIDEKGRAIHVEVLTRAGSGFEEEAILA
VRESTFTPARLNGHAVKSRAVLPIRFALTNSG

PPIIPRED:

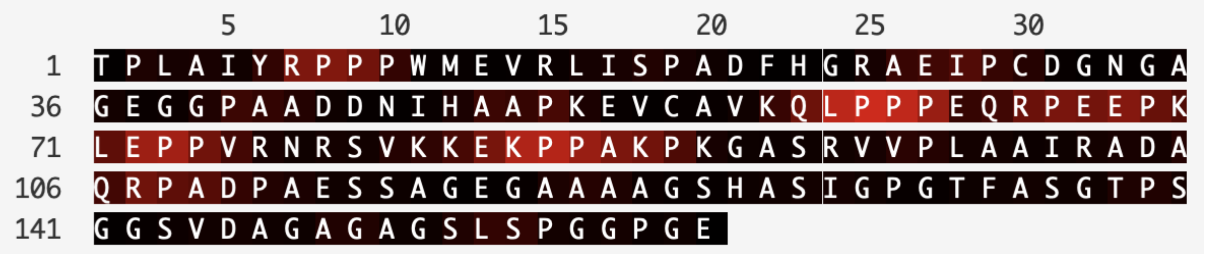

160 Domain II residues

25 Proline residues

6 α-Helix residues

Desulfobacca acetoxidans

Locus:

>AEB08475.1 TonB-dependent receptor [Desulfobacca acetoxidans DSM 11109]

MKRILFLLICLVLHPGIGLAQETASSEGVNPEETTNVAALGQSTSTSEDVQKVDEIIITATGTEEQLKKTS
VSSTVITAPEIEKRQVTRVEEMLRSVPGVVVNQTGSQGGNTSLFMRGGNSNMSQVLLNGIRLNNAG
GTYDWANLTVDNVERIEVVRGPMSSLYGADAMTGVTNLITKKGVGPPTFTYSGAWGAHSEKGQFIS
ENRFSLMGSAKDRFGYSIGFSRIDDQGILPINNRFGSNVLNGRFDLDPTEKLSFTFSTLFIDTYFGYPT
EYGDRVDPAPDPNQNQTRTDLLFGLTTKYSPYSWWENELTLSTYHRDWSYDDAFDPVDIFGGSVFH
PIEDRSSLDYRSNLRFNFSDRIGTTTTIGLAAFTERLKQSYESFGVWPYSSETRAHRRSVDFYFQEQIS
FWERLFLTAGMRLEDNTAFDGTEFSPRASAAFRITETDTTLRAAGGRAIKAPTFSEQYYQSTTAVGNP
NLKPEKNTSWEVGVDQYALNDRLKFGLTYFENHFTDLIAYITRAWPATSFYGNISDARVRGLETSLTA
KPLPYLTLSASYTYLLSQVLSTGGEDLGLNYEVGKPLVRRPKHTVSFTINYDHKPFNVNLNGLYVGKR
DDFYYITDPFFVTHTGRVFNPSYLLFNLAATYDIAENLRYAKKMQLQARIGNLFDQNYQETYNYSSPG
FNFVTGLRFVF

tonB next to motor genes.

>AEB08240.1 TonB family protein [Desulfobacca acetoxidans DSM 11109]

MECHYQEWISTWKTKDNWTWAILGSLLVHGLLVAGMLTSFMSHPAKKCVVVPVEAIALVPGPKGGG
GGRPAAATRPEAVPPKPSPVCPPARPQVKPKTVLKTRLAPPPPEPTMAPVIPTPASPPAITRGKSSPA
TVAACQAGSSGAAVGSSVSGQGGRGTGAGGGVGPGRGRGSGPGSGPGSALQGYLREIRRLLEKQ
KEYPLMARRRNIQGMVVVMFTIASGGQVDASQVSRSSGHDLLDEAARNTVRRVGRFPPIPVDLKRQ
KLTVAVPLAFCLNNE

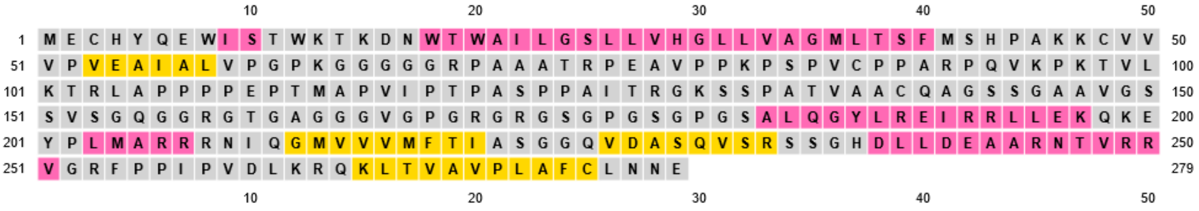

MECHYQEWISTWKTKDNWTWAILGSLLVHGLLVAGMLTSF
**MSHPAKKCVVVPVEAIALVPGPKGGGGGRPAAATRPEAVPPKPSPVCPPARPQVKPKTVLKTRLA
PPPPEPTMAPVIPTPASPPAITRGKSSPATVAACQAGSSGAAVGSSVSGQGGRGTGAGGGVGPGR
GRGSGPGSG**
PGSALQGYLREIRRLLEKQKEYPLMARRRNIQGMVVVMFTIASGGQVDASQVSRSSGHDLLDEAARN
TVRRVGRFPPIPVDLKRQKLTVAVPLAFCLNNE

PPIIPRED:

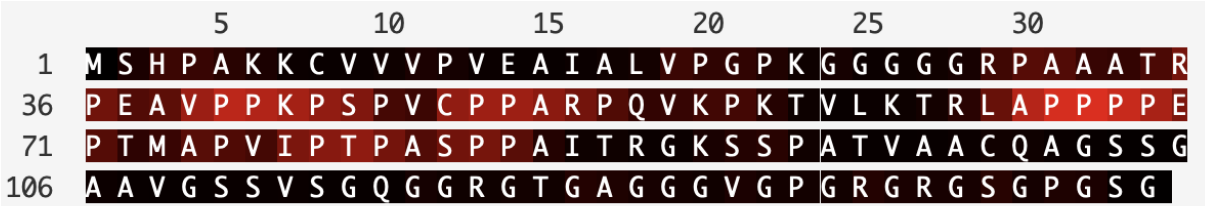

139 Domain II residues

27 Proline residues

0 α-Helix residues

Desulfarculales

Desulfarculus baarsii

Locus:

>ADK85828.1 TonB-dependent receptor [Desulfarculus baarsii DSM 2075]

MKRLLALLCLFCCPPCWPTAAMAAAPPPDGDIVVFSAADIQRMSVRTIQDLLNLAPGVKAGDSSVSIR
GDSAVAVYLDGMSLINNISAHKSVNWGLVSLEDVELIKVIKGGGAVSFGDNSSGGVIVITSKKAARAKA
GVEVEAGNHDYWKTSANVSQRAGAWGVSANGELESTDGFRPNDDEKHRRAGFKLSYAPEAWLAW
AGPDGQAPTLALDYGHTERGNPGLPGYATPHARSRDEALGLSLNLGAKGYKSTSSFTNFQNEFENT
DTAADTMLRGWTVQEDLRKSYDLPLLGRTAFGALISHTAASGNKIAGVDEQAYSLFANKKLGLKPLPL
SLAMGLRANAYSQFDGALNPELRLSWTRGPARLEAGFQMTNNTPSIRQRYYETTSTRPNPDLGSES
SANYSLGASYQPLKWLGGNATVFRNNIDDRITYMRVGGVGRYENVGRSHLSGVDASLSITPWAWLS
LRPSYTYLEAIDDTSGLWLPAKPRHKLMIDLQLRPLDGLMLGLTVAHCSEVFTNAANSAIAPAYEEIDL
RGEYQYKNARVYFRVENLADADYLYADGYPAPPRTWLVGLGWDF

tonB next to heterologous motor genes where one copy of ExbB has a large periplasmic domain.

>ADK85655.1 TonB family protein [Desulfarculus baarsii DSM 2075]

MSATMTKRRGGRWVGASLALGGAALVNVLVFCAAPLLQQPQHAAAEMPTYELTTFMPIKPPAEEEP
EPTPPPPPPKLETKVVRLDVSATDVTLVKPELTFEVNPKLAVGPAVAPPGPGRFDMGQVDQLPMAYG
RVPPEYPYLARRRGIEGAVKIRFLVDTQGRVMHLQVLAADPKGVFEESVLRTVQRWRFKPGVKDGA
PVETWVETAVRFTLDGK

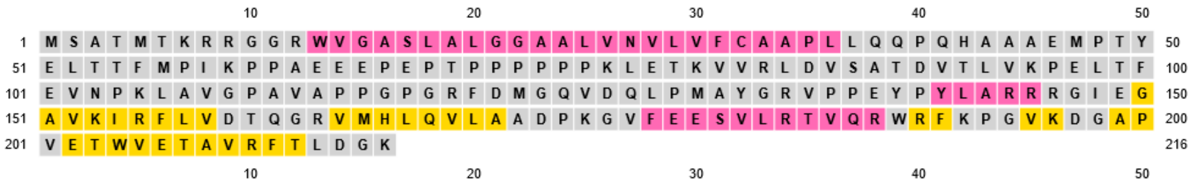

MSATMTKRRGGRWVGASLALGGAALVNVLVFCAAPL
**LQQPQHAAAEMPTYELTTFMPIKPPAEEEPEPTPPPPPPKLETKVVRLDVSATDVTLVKPELTFEVN
PKLAVGPAVAPPG**
PGRFDMGQVDQLPMAYGRVPPEYPYLARRRGIEGAVKIRFLVDTQGRVMHLQVLAADPKGVFEESV
LRTVQRWRFKPGVKDGAPVETWVETAVRFTLDGK

PPIIPRED:

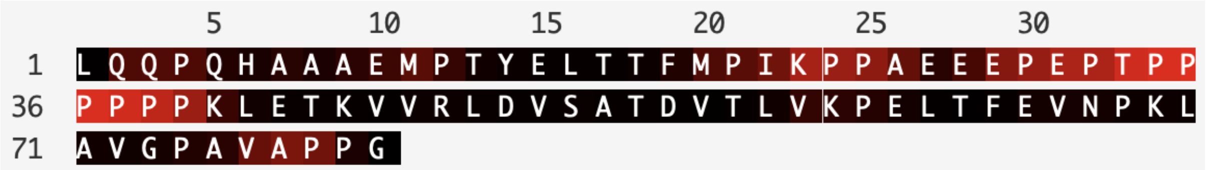

80 Domain II residues

18 Proline residues

0 α-Helix residues

Desulfobacterales

Desulfocapsa sulfexigens

Locus:

>AGF77330.1 outer membrane cobalamin receptor protein [Desulfocapsa sulfexigens DSM 10523]

MTKRYYPLLCAQLFTIAVVPGISAAETNSTTLMEEVVVTASRTEESRATVSSNISIISEDDIRQSASDNV
ADLLAEQSIATIKKYPGTLTSIALRGFSTDTHGNDLQGHVLVLLDGRRAGTGNLAKILTKNVARIEVIRG
PGAVQYGSAGMGGVVNVITKRATDNSIFTEAYGGSFDTAEGSIGGSFLKDGVDFTGSYTYGTTGDYD
TGNDEHYRNSGINYETGMSTNLGYSFSELNRIGLIFTRFEVDEAGTPGFLNRNDLDDYSNKENYSADL
NFEGQCPLTESRIFARYFFGQDENSWMDPTVSNPSGWNDGQLSKNKTDQQGAQFQLSRALGLATL
TAGFDWLDYEVENSWTPNTTEYSNSAIFLLSRLSFLEDRLSANIGLRYDWYDVAVTNPAGHNADDSH
FTPQFGVAWMVSDTLKLRAQYGEAFMMPSADQMSADYSSFGSRVVGNPGLSPEKSATWEGGVDY
GQNGFTGSLTYFHTDFKDKIVSDPLLDGSRSWKNLGDATISGIEAEFAYDIGLPLGLAWEVRPYLNMTI
LTRYDDEQSGEDLQNISATNYSAGVVVNDGDGIFCRLNTAYSGSQNIKDYESDFPYRNGTLDSHIVTD
LTASYRFYEDSRLGSFTLRGELQNIFDQDYAYVKGYPMPGRGFYAGLRWDF

tonB next to motor genes.

>AGF77335.1 TonB family protein [Desulfocapsa sulfexigens DSM 10523]

MRRFIPALILALCLHIFLLISRFPHLPTLPPRLTGQESVTISLSPGSSVEKQTETVTKEEQRNTIEKP
EKDEAAVETSEKINTHDKQPVVLKKKAVQKIVHSPSVPEQKKAGVQNSARPREESELPAVPILVK
ASPHYASNPKPEYPALARRRNQQGTVMVSVTVSEKGVPDRVSLHKSSGYPLLDKSALKAVTLW
HFLPGTTAGHPVATEVLIPVHFKLH

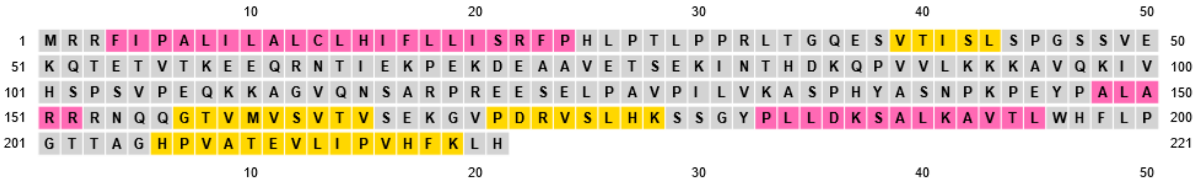

MRRFIPALILALCLHIFLLISRFP
**HLPTLPPRLTGQESVTISLSPGSSVEKQTETVTKEEQRNTIEKPEKDEAAVETSEKINTHDKQP
VVLKKKAVQKIVHSPSVPEQKKAGVQNSARPRE**
ESELPAVPILVKASPHYASNPKPEYPALARRRNQQGTVMVSVTVSEKGVPDRVSLHKSSGYPLL
DKSALKAVTLWHFLPGTTAGHPVATEVLIPVHFKLH

PPIIPRED:

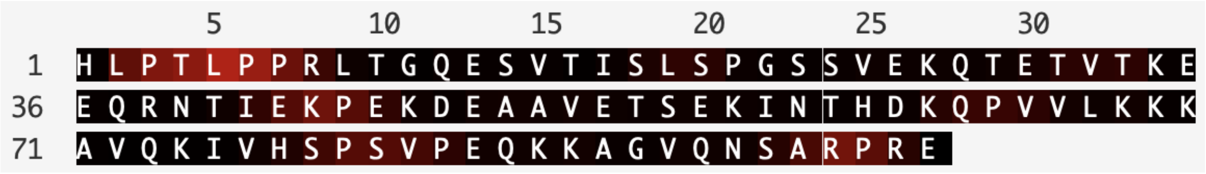

97 Domain II residues

9 Proline residues

0 helix

Desulfobacterium autotrophicum

Locus:

>ACN14007.1 TonB-dependent outer-membrane uptake protein [Desulforapulum autotrophicum HRM2]

MTRMPDLKRLAEARHPEASKQMHGLKVFLQPISIVLLLILAATGTAIPGEGYTQTEEQEQER
RITLDPVLVTARGRQSLASETPGGVGVVDQEEISESNSISLTNAAARIPGVDKSSDSAWGSA
MTIRGLGRNAVIFLVDGCRVNTSTDINARFGLVNPLDIEQIEVLKGPISALYGSGSTGGVVNII
TRKGKFTQTPTWTGEITNTIASNPQGNDSYGLATYNSKNVWVSASAGFRDHASYESGSGE
KIDNSQFEDSTLNASLGLRENPLNTTQFQVQHLRGEEIGIPGKGLALPTGPDVTYPDVNRTL
VNLTHTLTPDTPLISESSINLFYQKIERRVRLDHFPGGPLTELRPQADHDTWGAKWQNTLTP
RAHTIVAGVDVWNWKISNSNRYREFASGLTGVDASLADVEQFSGGVFMEDEWRIGKTVSL
NLGARLDYISAESQDLYNWITPPTPSTPVTLVQSGRSYSDTSWNAHGGVTWKFMPDWTMT
FITASAFRAPDLMERFKYINLGGGVELFGNPDLDPERSIFLEYGLKHTTDRFTISASAYVNFL
DDLITETVVDDTTHEMMNVDKARIHGGEIDVACYLTPSLTATATLAYIYGKNTRTDEPLAFIAP
LNGLAGLRYDGTSRFWGTIEMEWAADQNRTPDATPSGKGWERFNLHMGKKFTALGKNQE
LILGIDNLFDRDTTNYLSTSRSMELKEPGINFFCTWKIII

tonB next to motor genes.

>ACN13127.1 TonB [Desulforapulum autotrophicum HRM2]

MRLKEPDPEIVKKEPEKKRPDSESKGKKDTLKNIKQPPRSAPQRVAHDFPTLDFKVNPNLP
TTPQAPVFPMEQVAFSPQVMDRIYTGADIDNSLVPKVHIPPVYPFQAKRRGIEGWVKVRFL
VNPKGEVEDISILDSSPKNVFDQSVLNALPRWKFTPGTVEGVAVKTRVETTIRFELEN

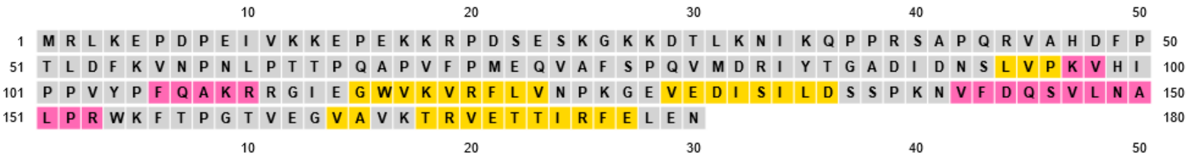

No TMH- analysis halted. No other *tonB* genes detected.

Desulfovibrionales

Desulfohalobium retbaense

Locus:

tonB next to motor genes.

>ACV67330.1 TonB-dependent receptor [Desulfohalobium retbaense DSM 5692]

MRSVLAAALCVAVVCVGTSVGAQNATQLSEIVVTATAAPATEQDVPVSTQIVDREAIERSGARDLEGL
LQREMAADFEIQPGGYSSFGIRGFESYESLGAGFDSGVLVLLDGTRIATGTLSLIPLDLVQRVEIVHGP
GSALYGGSAMGGVVNVITRTGGQETAGQVGVGYGSWEEKTAHVGVSGCSSEETFGYVLAGSKRTR
DDYAIGGGKTYPNTAIDMQSWGGKLTAGPGQQHQLHGVYLESRNNDLGSPGPLDDNVAENYIDDTL
RHLLVTYQGESTDGKQSWRLQPFHSEHEYAFYSNDMSKYITQMAGVRGQYNHQTARLGTLSLGAE
YTQIQDERSGSLVYEPNSEYDVTAVFAEHRFGSGPWDLWVGARYDHYRARIEKTSELKNVETGTET
YDALTWRVGGKYWLTANLGLHAAVGTAFRPPMAKELTGKFTSYGTTYIGNPNLKSEHSVTYETGME
WGGQHGRIEGTVFHTKYTDKIEGPSFGETGKYVNIDGWTVQGLELQGQTAFDWSEMLIVRPHATAR
YYTKREQEDSGGSSQTIFQLPEYEGQIGLAVDLGAKLSLDSWLEIRGPWEGKNVGRQTEFAVFGSRI
SYRPVVDLQVYLDVENLFDKRYSYKNGYPMPGRSVAAGIEYTF

>ACV68205.1 TonB family protein [Desulfohalobium retbaense DSM 5692]

MDERHCWVALLLLSCVLHGLVLDLDLFRIPSVAQKGDPVSLETVPPHQQTSDALALTAEMPATEADK
PHQSAADKRRSLLAQYVHKVRQRIERHKFPPTAGNCSDLIGKAKYAFTITGEGCFTNVHLIHSSGVAR
LDKTALLAIRRASGEVKRPPVTGSRPLFLQVTVKYQHGL

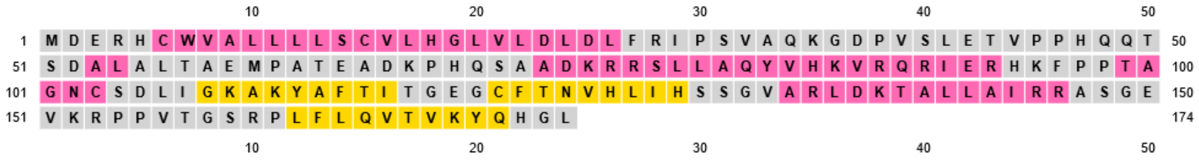

MDERHCWVALLLLSCVLHGLVLDLDL **FRIPSVAQKGDPVSLETVPPHQQTSDALALTAEMPATEAD**
KPHQSAADKRRSLLAQYVHKVRQRIERHKFPPTAGNCSDLIGKAKYAFTITGEGCFTNVHLIHSSGVA
RLDKTALLAIRRASGEVKRPPVTGSRPLFLQVTVKYQHGL

PPIIPRED:

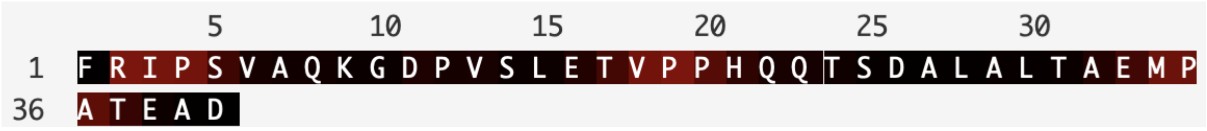

40 Domain II residues

5 Proline residues

4 α-helix residues

Desulfovibrio vulgaris

Locus:

>AAS94584.1 TonB-dependent receptor [Nitratidesulfovibrio vulgaris str. Hildenborough]

MGKRLMAALALAAMVCAGTAHAEDAPPASGGATLLEEEVVVSAPIIEGNEVTRYAGQYTEVTQEQITDLNAQDMSSALRRTPGV
NISRYNPVGSFGGAEGGGVFIRGMGSSRPGSEIMTMYDGVPRYNPVFSHPLLDMLPMDPAQSIRVYKGVQPTEFGNAFSAIDVI
PKRMTEDGYRTRLFTSYGSYNTFTQGVEHGGKQGGVDYYAGQSVRLSDGHREHSDGQIENYFGRLGYDLYENWNVSWFGNV
TRNVARDPGSSLSTGNDGRFYTDDVLNVITLANDYGDADGHIKVYWNNGYANWAGQKGNADTTISDWDLYGLRAREAFRAWE
GGEVTAGFDYDVMSGKALFENDNGTSSEFEREDFVLFSPYAAVSHEFGSRDDFYAIPSAGMRFYTHNEFGDQVAPHTGLVFGY
RDTELHAGYARGVNYPGLNVAVFSENVIPPIMGGPFRDDWKNLDPELVDHFEAGVSQKLWGKVKADLTAFWDEGTDRYAVVQ
GTPKRFRNIESFTIQGLEGTLTYTPFADLSVYAGATWLDTFFPSDLPYAPEWTFSAGLNWRFLQHFRLSLDAQYVTGEQVLSQGR
TENAVNASEVDPYHVVNGRIAYVFDYAPWEIKEAEVYLALENITDSDYEYRPGYRMPGSSATVGLSVTF

tonB near motor genes and ExbB with a large periplasmic domain.

>AAS96863.1 TonB domain protein [Nitratidesulfovibrio vulgaris str. Hildenborough]

MNGWWDRGTGIAVAQHLVLVGFLLFGLASGGDGKLEGDGVGGGSQLMELSLGGPGMPVSKPARA
AQATQPPPAARPPAAQTPTTRPRPDEATAVSPRKRRVEAKPRPKKETPRREVAQRAPVPRAEEKRT
VDTQPTQTTSTTTAPESSHEGDSAVANTAHAGVPGTGTTPGSGGPEKGMAYGTGGGTGGGSTAHA
GTGGEGLGGTGAGTGGYDRGPRAVYTPRPPYPREALHKGAEGVVMVRLLLDPSGRVVQSRIAGGD
MADIFAETTLETVERWRFKPCGRQGRNVSCEVEIPVEFRIDR

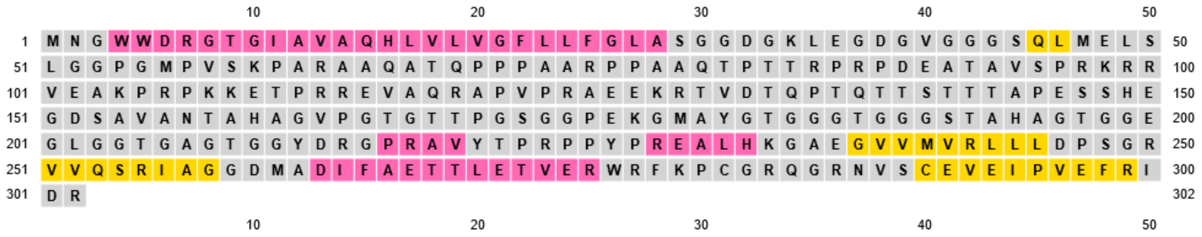

MNGWWDRGTGIAVAQHLVLVGFLLFGLA
**SGGDGKLEGDGVGGGSQLMELSLGGPGMPVSKPARAAQATQPPPAARPPAAQTPTTRPRPDEA
TAVSPRKRRVEAKPRPKKETPRREVAQRAPVPRAEEKRTVDTQPTQTTSTTTAPESSHEGDSAVA
NTAHAGVPGTGTTPGSGGPEKGMAYGTGGGTGGGSTAHAGTGGEGL**
GGTGAGTGGYDRGPRAVYTPRPPYPREALHKGAEGVVMVRLLLDPSGRVVQSRIAGGDMADIFAET
TLETVERWRFKPCGRQGRNVSCEVEIPVEFRIDR

PPIIPRED:

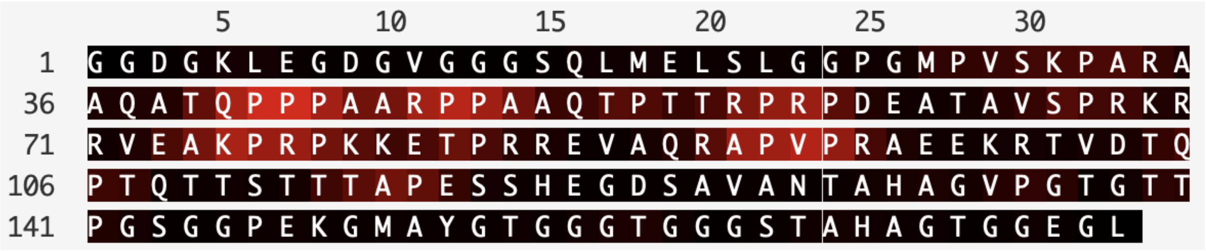

174 Domain II residues

22 Proline residues

0 α-helix residues

Desulfomicrobium baculatum

Locus:

>ACU90676.1 TonB-dependent receptor plug [Desulfomicrobium baculatum DSM 4028]

MVPHGSQSVAERDDHGSGNGTSPHNASGRSEGSADQWQALLTDQGDVVYQLEPVNVLGDKHQSG
KATIEGAELQSLPSHTGSITEAIKGFSNVQFSNEDTSSLTGGEIRPPRVSIAGAKPYENNFLIDGMSVTN
TLNPSGLDADGDGIGPNDLHVNGGDQTIFYDSSLVDTVTVYTSNVPAKYGGFVGGVVGAELIDPRIDR
WHAVFSGGHSRSEWFDLRGVNEDSTTSANQPRFRTYALRAGADGPLTDNVALLVAASQRRSVIPLK
METPEDSFYDKDQKRSSENFFTKLLFTPDDDLKLTLDATYAPYSEERWKPLYENSDWKTQNEAFRLA
GSATLGGTWGELGGRVAYSRNGYSRDSMSNLRETYAGTGVPEEDWYYRGGLGDAKVVNRGIDAG
LDVDLSTFKTGDLSWGLSSGLTLSNVTTDMWNEEARMEILTLPSSGKWTQVFTTYPESDQRRTLNTL
GWYVQAEMEWSRFTLTPGLRVDYDDFSHNTDISTRLKMELDTMGDGALRVVAGVNRYYGGQLRAY
AFDRYRPSSSLLIRYNTDPDNLPPVKVSDDQSYEAKGLDTPYSDELMGGLLGDVAGFEYGLEFVHRD
HRKQIISKAREEDVYELTNDGKSTYDGISLTLARSFETKRFGSHSLSLGVSQSRTKTFNGAFDSEIDEY
EESNGYEYDYDRVFYEGELVDRSSLPPDDYNAPAVVTLRWMGLFFDDRFRVNCVSRWRDSTSGLK
NDARTFDETPYGTAEKKKTTSSSKWLDEDGLYHDAYKTGIISGSLVTDVSLELDVVKEELFTMSLMLD
VFNVFAADGHVGVSQIGSGDVPAPRSEYGRGYYAGVRCEF

tonB not near motor genes.

>ACU90059.1 hypothetical protein Dbac_1971 [Desulfomicrobium baculatum DSM 4028]

MSPSETGFLTLSDFDQGIQAAQLKRMGQDVRIFGIRKFVLMGCIVLSLLLHALSGWMADFRPVPQLVK
DRLLLLDIVALSDETVSPAAQVPVAEKVTPRPVTSSDVTSSVSAASSSPVRKPVPEVKAVSKEKSPPA
QSKPRAQAEPAPERVRNPSPALQESVKSKTPIGQIVPRAGVTVLANESGQDALKHGAEARFMHGVA
TEEFVEENYVGEYSMGKSGKVWIEDDRAGSGHLILHAETMGLRRQLFRFNRFIYVYGESPDSPEPIL
GSVTFFSDGYHINNFLWQHNSTHAYYPRRE

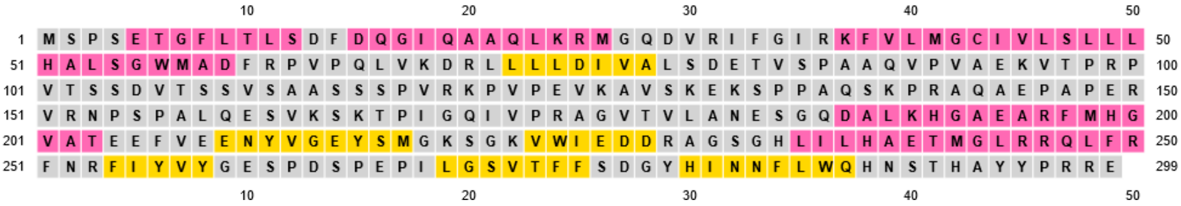

MSPSETGFLTLSDFDQGIQAAQLKRMGQDVRIFGIRKFVLMGCIVLSLLLHALSGWMAD
**FRPVPQLVKDRLLLLDIVALSDETVSPAAQVPVAEKVTPRPVTSSDVTSSVSAASSSPVRKPVPEVK
AVSKEKSPPAQSKPRAQAEPAPERVRNPSPALQESVKSKTPIGQIVPRAGVTVLANESGQDALKH
GAEARFMH**
GVATEEFVEENYVGEYSMGKSGKVWIEDDRAGSGHLILHAETMGLRRQLFRFNRFIYVYGESPDSPE
PILGSVTFFSDGYHINNFLWQHNSTHAYYPRRE

PPIIPRED:

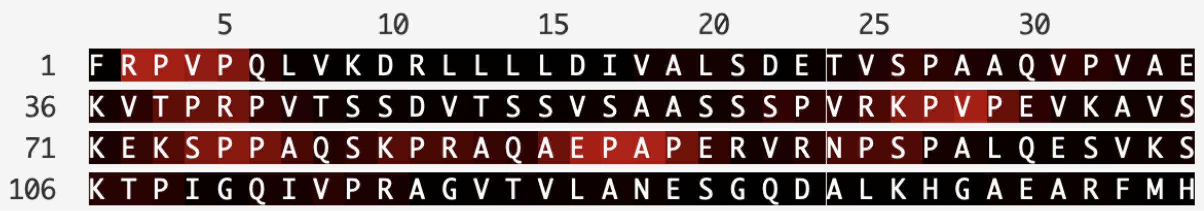

140 Domain II residues

18 Proline residues

13 α-Helix residues

Bacteriovoracles

Geobacter lovleyi

Locus:

>ACD95195.1 TonB-dependent receptor [Trichlorobacter lovleyi SZ]

MTVQQLSLLALLLTLTAQTARAEHLLLDEIIVRADKESPKEESLSVREVRESPARDMGEALKQVEGINIV
HKGAIANDVVLRGFQKDNINVLVDGVRLHGACPSRMDPPSFHYDFAEIEQVRIIKGPYDLSNPGGLGG
LIDAQTKRPGKGFGGELSLGYGDWHGTNAAATASYGTERYDALMGYAYKYSDVPQSGNGKLLTQIY
PATSPNRYKNTAIDSKAYEINTGWGKLGLNPTANSRTELSYTYQDADHVLYPYLKMDADYDRTDRMN
WSYRIQNLSALLQDLKLQVYWDRVEHLMDDRSRFSSSTSPRFYSMQTDASTQTYGAKLQAELKLGP
GSLKSGLDYYNRNWDATNRRAMYFNYRDLAMIPDVTIENFGLFGEYTLPLGGKLSLTGGVRGDLTRA
KADRANTMVTAGSSKEFSTISANLQLNYTPFKELVIFTGLGRGTRTPDQQELFLDLPGNPAWRGNQG
LKATVNHQADLGAKYATDRFYVNASIFYSDLQDYVNFYQASATLKSYQNIHASIWGAELGSQVSLPAD
LFLRGTLSYTEGRNISGNRPLSEMPPLKGTISIRYDNGSFFAELLETLSREQDRIDSSLNEQKTAGWVT
TDLKAGYQYKGFSVTGGINNILDTQYYSHLSYLRDPFVSGVGYRVPENGRNVYLTMAYKF

tonB not near motor genes.

>ACD96479.1 TonB family protein [Trichlorobacter lovleyi SZ]

MSAAHQSRKTRSIRLYLSVSLLLHGLFFVAAIVLLPPLVQAPPKEPALVMTHLVSLPTPNAGKETTSPA
PALPVINRPTPKSQPQPQPPPPKSVAPTTFSSNPPLKPSEPAPQAPPAPTFSAQQTKAGVASKPETG
SQPAVQTSTTTGGNRIAPQEMAFGSASGPAFRRQAVPVYPALAKRRNKEGVVLLRLSISETGQLTQL
EVLEDPGYGFGEAAQEAVRNSSFTPARHNGKPVAVRAVLPIRFSLR

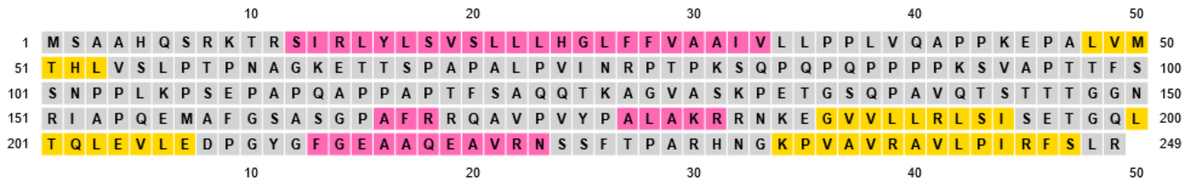

MSAAHQSRKTRSIRLYLSVSLLLHGLFFVAAIV
LLPPLVQAPPKEPALVMTHLVSLPTPNAGKETTSPAPALPVINRPTPKSQPQPQPPPPKSVAPTTFS
SNPPLKPSEPAPQAPPAPTFSAQQTKAGVASKPETGSQPAVQTSTTTGG
NRIAPQEMAFGSASGPAFRRQAVPVYPALAKRRNKEGVVLLRLSISETGQLTQLEVLEDPGYGFGEA
AQEAVRNSSFTPARHNGKPVAVRAVLPIRFSLR

PPIIPRED:

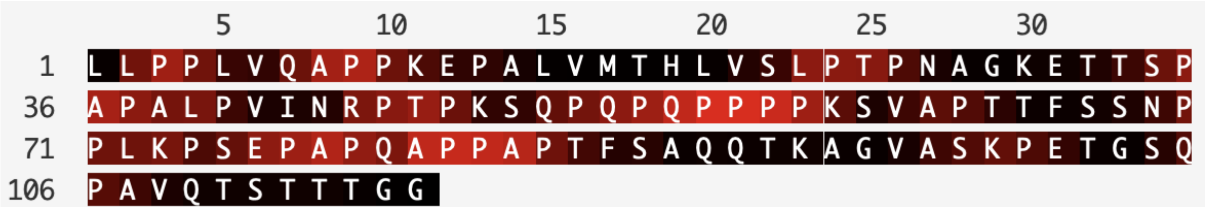

116 Domain II residues

29 Proline residues

0 α-Helix residues

Pelobacter carbinolicus

Locus:

>ABA87714.1 cobalamin uptake ligand-gated TonB-dependent outer membrane channel [Syntrophotalea carbinolica DSM 2380]

MVKIPSLALVGLLMLSATASASDVTTLEPVVVTATKIETPTREVASSITVVTAEEIEEKQQRTVLEALRD
VPAVDVVQSGGDGQQTSVFMRGANSEHTLVLIDGIEVNDPISPSKAFNFANLTTDNVERIEILRGPAS
ALYGSDAIGGVINIITKKGQGKPSITLSAEGGSFETHHETVSLSGGTQLVNYALSASYLDSNGITAARW
SDGNHERDGYENLTTSLRLGLTPTKNFDLDFILRYMETDTDIDNSAGPLGDDPNYTTEEEKLFFRTQA
GLYLFDDLWEQKLGFSITDYDRSTRDDTDAAHPLDWVRSSYQSTLYKVDWQHNLYLHKTNTLTLGIE
HEEEEGKNKFLSESAYGPWNTYYPRQKTRTTGYYVQDQFKLWNRFFTTLGLRLDDHEEFGRRVTYH
VASSYIFDSTGTKIRATWGTGFKAPSLVQLYDTNFGGNPDLNPEKSEGWDVGIDQNLWQDRLTISLT
YFENSFEDLIVNEYLGWGPTGAIYLYKNVDSANSKGIELLLTCRPLETLTITAGYTYTDTENEDSHDQLL
RRPRNKFTADINYRFLEKGNVNLSMIYVGKREDSFYNNATWASGRVELASYTLFNLGASYEVTKWLT
LSGRIENLLNEHYEETWGYDTAGIAGYMGAKLTF

tonB next to TBDT gene, not near motor genes.

>ABA87713.1 periplasmic energy transduction protein, TonB-related protein [Syntrophotalea carbinolica DSM 2380]

MLSQETFLPWIILSLGIHITALTLWPLPPSWEATRPGPIAVDLRYSTQPGGNNQPNKTPGPGTHGPKAI
MPPKVLHPHPKPAPPHKSSRPITPPAPIKKAAPHRTPRKSQSQKSAPAAISAPEQQPEKSLEPPSDQP
ESDLADTSMENLNGAASATDDTNASHASLSPASGTGTDNTGYGSGENSGTNGGGGGDGIIRATPLG
YGENPPMPYPRSARRRGWEGEVLLKVDVSAHGRVLAVHIEQSSGYGILDDTALGAVSEWRFRPARV
NGSTRPDTVIVPIHFRLDAP

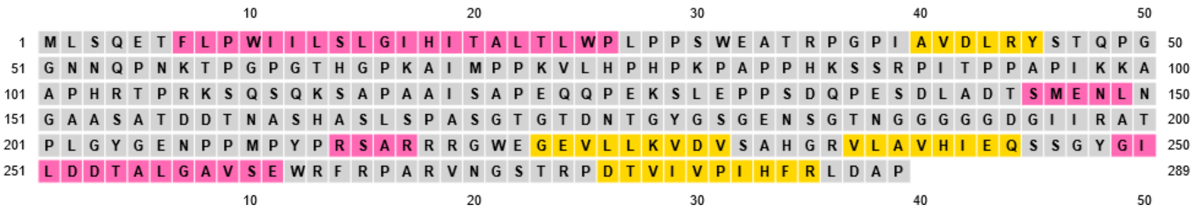

MLSQETFLPWIILSLGIHITALTLWP
**LPPSWEATRPGPIAVDLRYSTQPGGNNQPNKTPGPGTHGPKAIMPPKVLHPHPKPAPPHKSSRPIT
PPAPIKKAAPHRTPRKSQSQKSAPAAISAPEQQPEKSLEPPSDQPESDLADTSMENLNGAASATD
DTNASHASLSPASGTGTDNTGYGSGENSGTNG**
GGGGDGIIRATPLGYGENPPMPYPRSARRRGWEGEVLLKVDVSAHGRVLAVHIEQSSGYGILDDTAL
GAVSEWRFRPARVNGSTRPDTVIVPIHFRLDAP

PPIIPRED:

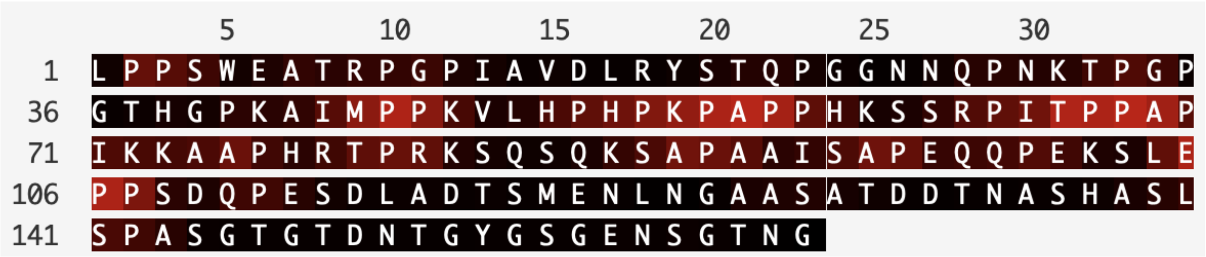

163 Domain II residues

29 Proline residues

5 α-helix residues

Desulfurellales

Desulfurella multipotens

Locus:

TBDT found on different scaffold.

>SDC20763.1 iron complex outermembrane recepter protein [Desulfurella multipotens]

MKRVWLVCLFLTILSNYAFADDSKNLEAVNVSATPIVEPTKQTEQTVFSGLEVTKQGMDLGGTQAQTSVYNAINILPDVSIENADS
SGLYAEGTNVRIRGVRGYLGAMTVEGIPNYGGNPMGPRDYIYNMQNFKAIKVYESAMPASLGVGVGNRGGAIELVPLWPLKTT
QFKMQQTYGSYELYENFFRFDSGNIDKTNTRFSVSYSYGIANKWRGPGEIGPRNNVNFMLDQPVGDVFDAKVFFNYNFVKQNL
YRALTYAQIDDTNLDYNSSKTSVASKDINYFGYNHGDFQNTDFFSILTIKPTNIFDVVLKPYYSKENTHIWQGISPQNIVQKRTRDID
RKGIIAQFDLNVNHFKVSSGMQYEVSNMDIYSQNYAILGDNLSYRGYGVFATTGDTYIKSPFLQVAFEDQKFKAQAGIKYFRFND
SASQGYVTSKTAPYSLVRAPDLDRKERLYDIWLPNVGLSYKFSDEFELYANYGRNFIRPYAYMPLINLYNTNRQAFQKAHITLNDL
FDGYNIERSDNIDLGARIRGSWFDLLPTFFYSKQKDLLTTVYDPRVKLNYQQNIGKATGYGFELAFNAYVNDYLTFFFNPSFTRLT
YDNDISYQGSLIGCKDKQIVDVPKWLIKSGFIVNYDKWQFIPSVIYVGSRYADAGHTQEVASYATLNAQINYTLKNIYKMHELKLSL
QLNNILNKKYIAVINSMDDALAGNTSFYPGEPFNASLTASLTF

tonB next to acyl-co-A thioesterase gene, not near a motor.

>SDC93247.1 outer membrane transport energization protein TonB [Desulfurella multipotens]

MEKFYTKKTFTSSLALSIFVNVIFFALGIKLLSGFYKPIKKPIEVVLLGISQPKTDDKILHVKNKTLAQKKQ
NILITQESIPKEKINNKVFPKQEIQKTGNLKTADKTDKIEKTNNAIVHTDTISNNESNTKSSKGSAVQTTQ
NIQTTQTTNSFQSVSKEDYAFLRKLIEEHLKYPYLARRNSYEGTVVISFIIDNGIIKDIEVKSSGYSILDKS
AIEAIKKIEPLVKLDKNVKIVIPINFKLNNS

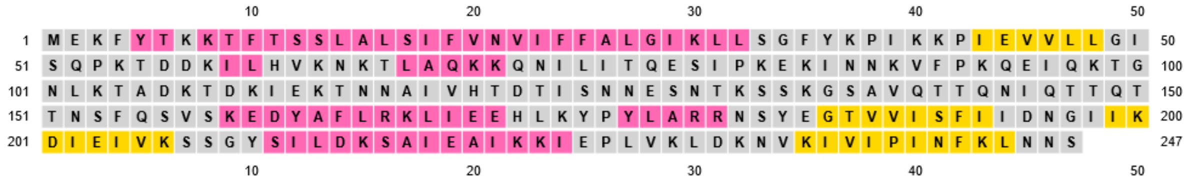

MEKFYTKKTFTSSLALSIFVNVIFFALGIKLL
**SGFYKPIKKPIEVVLLGISQPKTDDKILHVKNKTLAQKKQNILITQESIPKEKINNKVFPKQEIQKTGNL
KTADKTDKIEKTNNAIVHTDTISNNESNTKSSKGSAVQTTQNIQTT**
QTTNSFQSVSKEDYAFLRKLIEEHLKYPYLARRNSYEGTVVISFIIDNGIIKDIEVKSSGYSILDKSAIEAI
KKIEPLVKLDKNVKIVIPINFKLNNS

PPIIPRED:

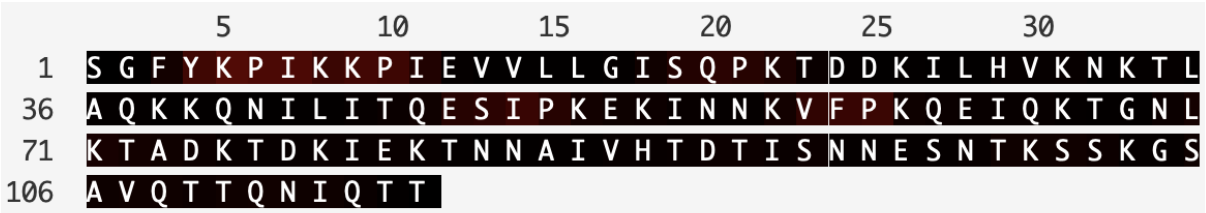

116 Domain II residues

5 Proline residues

7 α-Helix residues

Oligoflexia

Bacteriovoracles (cont.)

Halobacteriovorax marinus

Locus:

>WP_096908354.1 TonB-dependent siderophore receptor [Halobacteriovorax marinus]

MKLSFIVATLLCFNLQAQTNILVSAEKISLDKDQTVSDVDVIDAAEIEASGESSLVDLLADRASLYINSNG
GFAKATSLFLRGADSSYTLIEIDGVEYNDRSSVGGAAILEHIDLSNIEKVEILKGAQSVLYGSDAMAGVI
KITTKTPGKYVGANASIGYGSYDNKRASFSTSQKGKTMNYVLGMSFQDVEGFSSYNEKRAPFAERD
GMNNLTATFKGVKKLGSTDQLMFNVRGVKAQSDFDASTSDKLDYMGRDEQLIAGVSYKKRIGDYWI
PELSITYNKSDRLSNSFSLSRLVAETKKVELKNPLYINESITILNGLEYEDIEASIENINNKKNYNSYATYL
DSHLQRGAFKLQAGLRWSKEKSLSDQIVWKGGASYRLFENTFAKLNASTGYKSPSLYQLFSSFGNE
QLRATKSKSYDFTLEQRFSAFVLSGTIFQNEYENVIDFDSTLNKYTNTFKSETKGVELGLDISTEKFDL
SSSATILRAFNKSAGSDGIYLARRPREKYYLGLKYKFNEKLSLSNNYNYVGRRENSDFDTVVLSSYLL
VGLNLNYEISNDQAVLLKIDNALDKEYEQIHGFGTAGRSFFLRYNFKL

tonB not near motor genes.

>WP_096908355.1 energy transducer TonB [Halobacteriovorax marinus]

MNSSLIFGIFISLVFHSLLVIDLSIKSEAEVTMMMSSSKSSFKVNLNKEKKSPKRKGIGQNKKVSNEVKA
DESNLAKTSVHVANQFKPQYPYRSRLFSEEGIVFVNVKIDKSAKVIDASIAKSSGFKRLDNAALEAARK
SFFTVTQKGRESVVEYVLEFNFKLNEGE

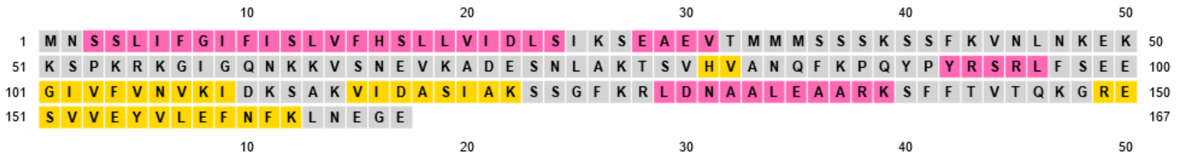

MNSSLIFGIFISLVFHSLLVIDLS **IKSEAEVTMMMSSSKSSFKVNLNKEKKSPKRKGIGQNKKVSNE**
VKADESNLAKTSVHVANQFKPQYPYRSRLFSEEGIVFVNVKIDKSAKVIDASIAKSSGFKRLDNAALEA
ARKSFFTVTQKGRESVVEYVLEFNFKLNEGE

PPIIPRED:

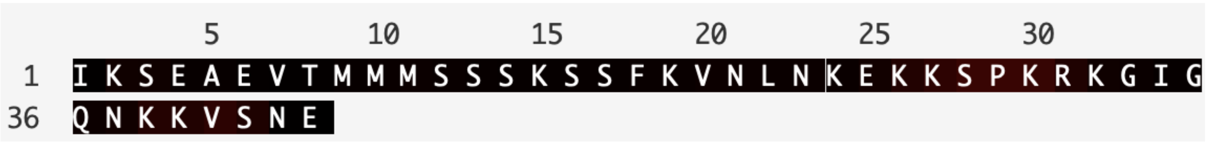

43 Domain II residues

1 Proline residues

4 α-helix residues

Bdellovibrionales

Bdellovibrio bacteriovorus

Locus: (different to tol-pal locus)

>ASD62360.1 outer membrane receptor protein [Bdellovibrio bacteriovorus]

MKYSVLLACLFLGLAAHAQEAVPSFETVVEDVVFNTSNKVVIDEKTIKDSRAPNITSLLSSQANITVVST
PFQPNSIFIRGGDSGHVMIIVDGVPFYDASTVQRTFNLNSLDIKSVRRIEIIKGGQTVLYGGQALSGVIKI
DTIPQEIKSQSSLQGQLGTQNFRDVTVGHTEALNDNNALVLRGHGAWRDAESPVLDSTETYSRNNW
NAEGAYVWKGAVDGNLKALFLQDFNTSPTTDRTTNQVMDTEDLEMFTRQIGGSTYMKFNELPFEPR
LALSMQNSLRTYEWPVVPVQNPTGTDQKYGANLRTARLDLTPYKSEKISVAAGLSYLYEDFTYRDKG
VESVNTFSEQRGVFAKADYEFHPGFSLAVGGRVENWADQDAVSTYQIGLTVFEHTKLEVASGYKIPS
LFQLYSSYGNPDLKAERAVQYSLMQEFEISESQSASITFFRSDFTDLIQISGSFPSIQYENVSKTETRG
VDISYTIRPWTGGTFIATYGYQEPRNVDTGTWLLRRPLVNGSLKYIQNHDKHTAALELVGAGERMDN
GAPISMTAYASTSIPGYVTANAAYSYQWNDNINVFTRLNNLTDHRYEETYSFYSEGFSGSLGAEYLF

tonB not next to motor genes.

>ASD62361.1 energy transducer TonB [Bdellovibrio bacteriovorus]

MRRSKGLKLKLWILLSVILHLLAVTALQWGASPVSPDLGTVVDLTVTSPAGVSGLPPAPPAAVSKPAP
TVPSESVKSEAPETTANADAGAAGGGGDSTTPVGFSEITRLPKVKREFKAQYPEEAKKAAVDGPVILE
ILIDREGKVRHVQVLSGPGHGLNESAVEALKKFEFQPAFKGEESVAVKIRYTYRFKLEVN

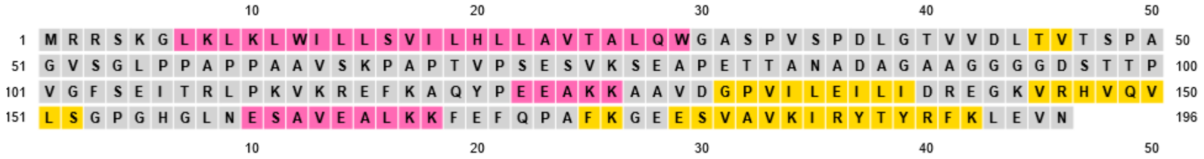

MRRSKGLKLKLWILLSVILHLLAVTALQW
GASPVSPDLGTVVDLTVTSPAGVSGLPPAPPAAVSKPAPTVPSESVKSEAPETTANADAGAAGGG GD
STTPVGFSEITRLPKVKREFKAQYPEEAKKAAVDGPVILEILIDREGKVRHVQVLSGPGHGLNESAVEA
LKKFEFQPAFKGEESVAVKIRYTYRFKLEVN

PPIIPRED:

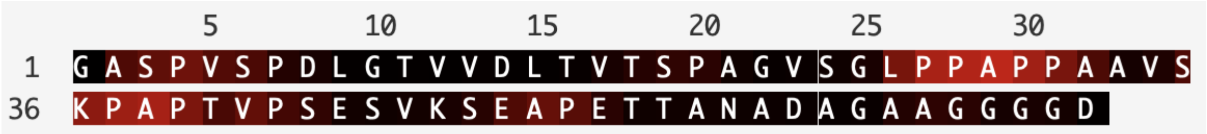

67 Domain II residues

11 Proline residues

0 α-helix residues

ε-Proteobacteria

Campylobacterales

Helicobacter pylori

Locus:

>ABF84926.1 iron-regulated outer membrane protein [Helicobacter pylori HPAG1]

MFLRSYPKLRYALCFPLLAETCYSSDHTLGKVTTQAKRIFTYNNEFKVTSKELDQRQSNEVKDLFRTN
PDVNVGGGSVMGQKIYVRGVEDRLLRVTVDGAAQNGNIYHHQGNTVIDPGMLKSVEVTKGAANASA
GPGAIAGVIKMETKGAADFIPRGKNYAASGAVSFYTNFGDRETFRSAYQNAHFDIIAYYTHQNIFYYRS
GATAMKNLFNPTQADKEPGTPSEQNNALIKMNGYLSDRDTLTFSWNMTRDNATRPLRSNAIGLAYPC
EAPFSPDGAQGCPNVLDSFTRYMYHSINSANNLSLQYKREAGNSFGDPRLDFTLYTSIRNAQFDPLF
DPNGVYAKFPTSQASAWERENYPCVEGAYCTPSFSDVDKPSSQPRDLFLNNTGLNLKVAHVIDEAT
DSLFEYGFNYQNLSVFDARIPKSELYRPNQVYTDDKGQKQIACSLVDNNPNDPTLCQRGKANGNIYG
GYVQANYSPHKIITFGAGVRWDAYTLYDKDWNHRYTQGFSPSAALVLSPIEPLSLKITYSQVTRGVMP
GDGVYMRQNDLRYAKNIKPEVGSNAEFNIDYSSQYFSGRAAAFYQALDNFISQYAQNLIVTNLSQAIRI
YGYEVGGTFRYKGVSLNVGVSRTWPTTRGYLMADSYELAASTGNVFIIKLDYTIPKTGINLAWLSRFV
TGLDYCGFDIYLPDYGTAEKPKTPTDLAKCGSKLGLVHMHKPGYGVSNFYINWSPKTKSRWKGLLLS
AVFNNVFNKFYVDQTSPYYVMSPDMPGTDAVKRAIAEPGFNARFEVAYKW

tonB next to motor genes.

>ABF85355.1 siderophore-mediated iron transport protein [Helicobacter pylori HPAG1]

MKISPSPQKLSKVSTSVSFLISFALYAIGFGYFLLREDAPEPLAQAGTTKVTMSLASINTNSNTKTNAES
AKPKEEPKEKPKEEPKKEEPKKETKPKPKPKPKPKPKPEPKPKPEPKPKPEPKPEPKVEEVKKEE
PKEEPKKEEAKEEAKEKSAPKQVTTKDIVKEKDKQEESNKTSEGATSEAQAYNPGVSNEFLMKIQTAI
SSKNRYPKMAQIRGIEGEVLVSFTINADGSVTDIKVVKSNTTDILNHAALEAIKSAAHLFPKPEETVHLKI
PIAYSLKED

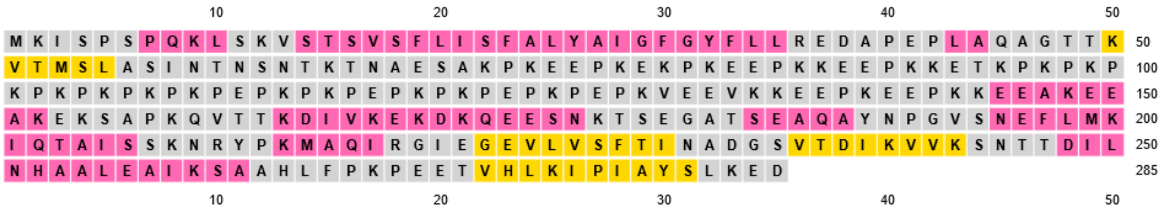

MKISPSPQKLSKVSTSVSFLISFALYAIGFGYFLL
**REDAPEPLAQAGTTKVTMSLASINTNSNTKTNAESAKPKEEPKEKPKEEPKKEEPKKETKPKPKPK
PKPKPKPKPEPKPKPEPKPKPEPKPEPKVEEVKKEEPKEEPKKEEAKEEAKEKSAPKQVTTKDIVK
EKDKQEESNKTSEGATSE**
AQAYNPGVSNEFLMKIQTAISSKNRYPKMAQIRGIEGEVLVSFTINADGSVTDIKVVKSNTTDILNHAAL
EAIKSAAHLFPKPEETVHLKIPIAYSLKED

PPIIPRED:

150 Domain II residues

27 Proline residues

26 α-Helix residues

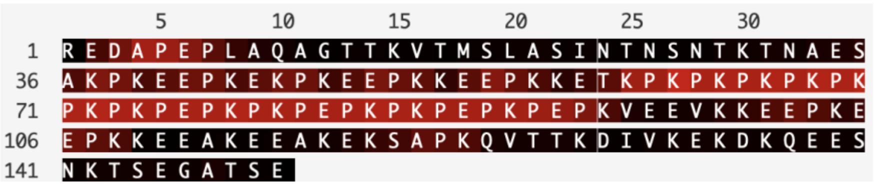

Wolinella succinogenes

Locus:

>CAE10216.1 TONB-DEPENDENT OUTER MEMBRANE RECEPTOR [Wolinella succinogenes]

MKNSIVLWGSLGLASVLMADSSVYELGRVEINEKSDISKNPSLEIVTQETIRETEAKSVVDALQSVPGIY
TDYAGARGETKVRLRGFSSTRVPLYIDGIPIYVPYDHNIDLGRFKTYDIGEIDVSKGYVSPMYGANTMG
GAINLVTRRPTKEFEGEVGAGIFSGKGQEQYLTLGTKQELFYGLISASNVQRDYYKLSGDYEEAGHQ
QGKKRSNSESKDQKVNLKVGYTPNDTDEYSFNYIVQRGEKEQPWFAMERRKNSDGSWKDGSGMY
NRNWDWADWDKTSYYLITKTAIKEHYLKTRWYYDTFYNKIFFRGTPGSPTQNIITETSEYDDNTFGGM
VEGDLQLAKGHLLKLFVSQKYDNHKDKNYVDSSNTRYADLKDEGKTTSLGAEYSWLMSDQWTWTL
GGSYDKNEVTKAEYRNSAGTQILGEFPKYETNAFNPQTILSYKAKEGLTFYGSISKKSNMPTLKDRYS
SKFGDYVLNPGLDSERSTTYELGSNWAVNENHYAKTALFLTKTKDYIASYSGVSNSDSTLGCVGANC
KQMQNFDQEEHKGVELTLDSYWSDQFKSTLSYVYIDASIEDSQTKDAKYTTDTPDHTLYASVTYSPF
YSINITPVVRYESERYLSVDGSDKSTPHTLVDLRVAYRPVKSFEVAAGVKNMFDKNYYISYGYPQEGR
NYYLNVRYSF

tonB not near motor genes, near TBDT gene.

>CAE10218.1 hypothetical protein WS1128 [Wolinella succinogenes]

MPPMSERRYSQVGWSFSIVLHGAIWLFWAFLGVTPPVIEERKISLPLGMILSQEPSPLLSAPTPAPPPV
MPLESSEPLEIPKPSLKNPAPHPKPLVKNRDKMAIEPKVTPLMSQERESISTDSLLSPAPLAVMEGVR
ETKEVEEEAYLFEQIRQIIQKNRSYPERARRRNIEGEVVVEFCLTPRGEVKDLVLVQSSGSAILDQHSL
RLIEESKEQFPRPKESIKIELPIGYRLL

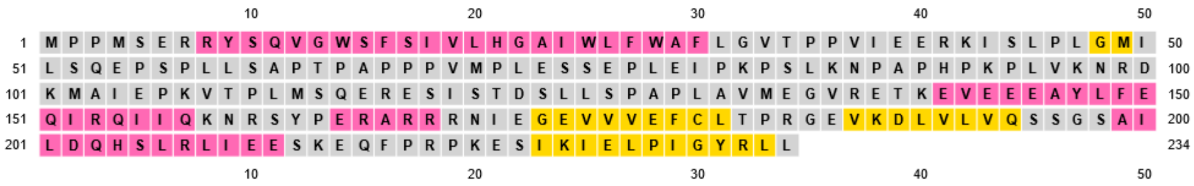

MPPMSERRYSQVGWSFSIVLHGAIWLFWAF
**LGVTPPVIEERKISLPLGMILSQEPSPLLSAPTPAPPPVMPLESSEPLEIPKPSLKNPAPHPKPLVKN
RDKMAIEPKVTPLMSQERESISTDSLLSPAPLAVME**
GVRETKEVEEEAYLFEQIRQIIQKNRSYPERARRRNIEGEVVVEFCLTPRGEVKDLVLVQSSGSAILDQ
HSLRLIEESKEQFPRPKESIKIELPIGYRLL

PPIIPRED:

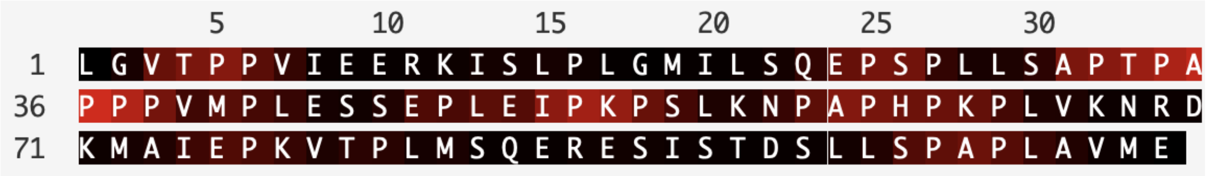

104 Domain II residues

22 Proline residues

0 α-Helix residues

Arcobacter butzleri

Locus:

>ABV66603.1 TonB-dependent receptor protein [Aliarcobacter butzleri RM4018]

MKPIKKTITYSICLSMSLLSQTFAQETTLLDEIKVSTENSNYSTQNDNYYKTKSQSATKTDTPIRETPQS
VQVVSNEIIKELNAVKIEDVLDYTSGVSRQNNFAGMWDNFSIRGFAGNENTGMSLLKNGFADNRGFN
APRDTANIESIEFLKGPSGSLYGNSEPGGTINIVTKQPKFTSEHSIKTDVGSYDFYRMALDSTAPINDNL
AYRLNVATEKKGSFRDHIESQRYVVAPSLLYSINDDTFVSYMGEFIEQKAPFDRGIALIDGKNVMNPKN
FLGNPDDGDVTLKNQTHQLKLEHYFSDSWSSRMGVAYKNNSLKGFGSEVTPATKITTDSINLRTRYR
DYSSDDIQFQVDLQNVTDIKDATNTLLFGVETYRFEQDSILYTNKDTVRVDNIRSNPTYTVLKTGLGSL
STDKYEEQKGVALFVQDEIAYKDFRFLTGLRYDEVRMDNVNHLDSSSVKQNDYAVSPRVGITYLIDD
MWSVYTTSGTSFRPNTGTDIDGKTFESERSVSIETGLKFESEDKKTGGTLSLYQIEKKNVLTKDPNDD
LFSIAAGKVKSKGIEFDFNGKITDNIKANFNYTYTDAKVVEDSTYEGKELLNIPKHTSSVLLMWEDTLSL
NSSYGIGTGVTYVGRKAGNVNNDFYLPDYTTAKIVSYYKVNKELNFSLNIDNLFDKEYIASSYDRSWLT
VGNPRTATLSMTYKF

tonB next to motor genes.

>ABV66968.1 TonB-dependent receptor protein [Aliarcobacter butzleri RM4018]

MNRYLNSFFITSGVYFIAAFFLFFVFADMLITPPAKEEIVTKISLNSVSIVEPQPVVEPTPEPIVEPQPVIE
KPKPVKNKPEKPKKEHKKPVEKIVQKEEVTEVVAAVPPPTKTPPKEEVSAPSINQNQIDTIETKYLAKV
RATIEKNKIYPKAAKRLNQTGKVNVNFDILKSGEIRNVKVLGKSSFAKLDEASIELLIKISNFDEIPEELKK
SVWNVTIPIDYSIN

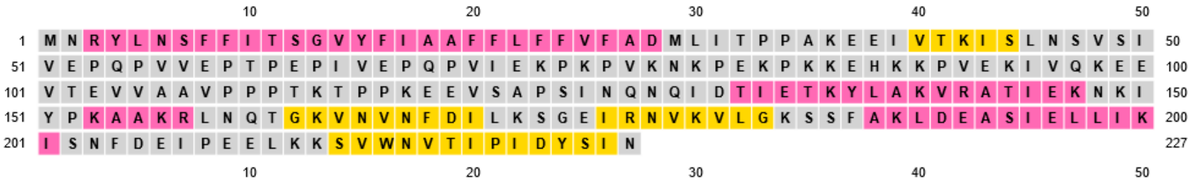

MNRYLNSFFITSGVYFIAAFFLFFVFAD
**MLITPPAKEEIVTKISLNSVSIVEPQPVVEPTPEPIVEPQPVIEKPKPVKNKPEKPKKEHKKPVEKIVQK
EEVTEVVAAVPPPTKTPPKEEVSAPSINQ**
NQIDTIETKYLAKVRATIEKNKIYPKAAKRLNQTGKVNVNFDILKSGEIRNVKVLGKSSFAKLDEASIELLI
KISNFDEIPEELKKSVWNVTIPIDYSIN

PPIIPRED:

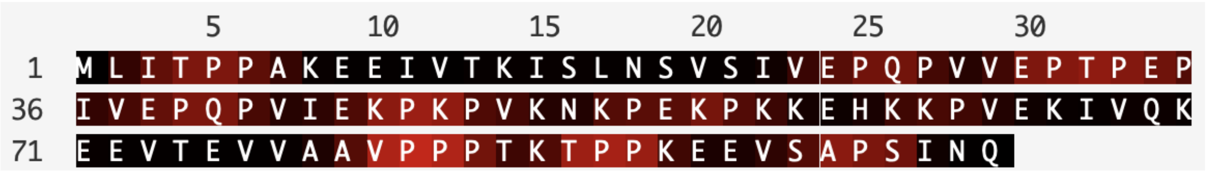

99 Domain II residues

20 Proline residues

0 α-Helices

Campylobacter curvus

Locus:

>EAT99645.1 TonB-dependent heme/hemoglobin receptor family protein [Campylobacter curvus 525.92]

MNKICKFSIICTALLSLSNAAQNESEDLTFDTLEVSAKSVSNAEKSFATPGAVSSRDDVKSKTQSIDSIV
RAMPGSYTQVDQSQGSVSVNIRGLTGLGRVNTMVDGVTQTYFGTSSDSGGFHNFTGNLGTSAFGA
LIDQNFLVGIDIERGTFSGPNTGLMGSANFRTIGVDDVITEGKPFGFLGRYSYGSNKVGPSYMGAIAA
RHRFENGTSLGFVFGYSGKKTSQNYTVGGGGKISDKQPDLDGDGVPDVPPSLDTDDLVQKPKSYLF
KTEYAHEDSSAILSYRNLNNYLAKRKINNDSYQLNYRYDPSSDLVDVKFLAAYNRSKQNYDKGASISW
GTLKNGMKFYNKSSQFDLSDTMKFNPASELFITTSAGINLLTNKYTKQTGDSDYKTLTTNYAVPTGEQ
KAVSYYLNNAFSYDIFEFDANFNLQKWQTKGRKGKCALSNPLCSQKEAGEFKTDDTYLNASLMASAK
LHDLFSPFVSYSRTSRPLNVQELFASGTVYEDINTALRPEKTQTWQIGFNSYKHGLFADDDVFGLKAV
YYRSKVKDFIYDRLLMFDTPDTTMFLARLNDKAKFRGFELELTYDIGYFYTNLSYTRQKTTYLPSDSSA
LDWTNGPASGQSQFSELPKDYATLDIGTRLFGEKLTFGSLIKYTGKAKRIYPKAEFTPSPNNQNPLFK
KLQTQELPKIPTVIDLYASYEPIKNLTLKFEVQNLTDKNYMDALYTYNSSYSSQVFNDDIVLFNNSARG
RTFLMSFEYRY

tonB next to motor, where ExbB has large periplasmic domain. Also near TBDT.

>EAT99643.1 energy transduction protein TonB [Campylobacter curvus 525.92]

MTRRRFLLPFAFLLSLSVHIFIVFLFMQSAPSQNNGGYIGESGEFQSVRIVSSLPIGELMDTAINSQKQT
QLQTPPEQETSEGSLSDTDVKSQITVKKSKVFSKKQTVKRLPEKEKKSNEKRETTEDQTQKDKDTGD
SQVDSVSANSVASAPVQGSGDKLSSPNDGNSQSTGASWQGAVMSHLNKHKKYPNEALAKKQEGK
VLLLVKIAEDGAVLECKIKKGSGVALLDEEALNLFKRASPLPKPPQSVLKGKREITFSIPIDYNIKKFLER
DLLRNGG

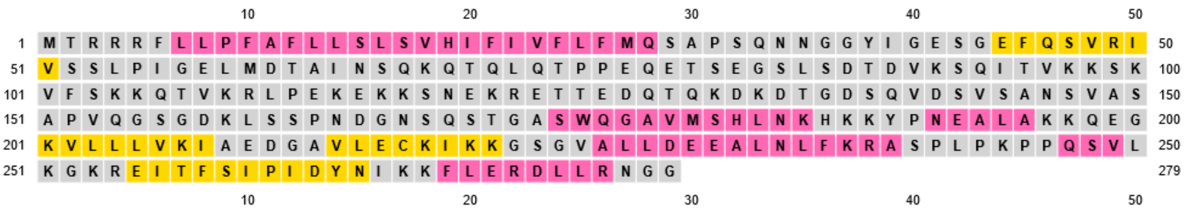

MTRRRFLLPFAFLLSLSVHIFIVFLFMQ
**SAPSQNNGGYIGESGEFQSVRIVSSLPIGELMDTAINSQKQTQLQTPPEQETSEGSLSDTDVKSQITV
KKSKVFSKKQTVKRLPEKEKKSNEKRETTEDQTQKDKDTGDSQVDSVSANSVASAPVQGSGDKL
SSPNDGNSQSTGASWQGAV**
MSHLNKHKKYPNEALAKKQEGKVLLLVKIAEDGAVLECKIKKGSGVALLDEEALNLFKRASPLPKPPQ
SVLKGKREITFSIPIDYNIKKFLERDLLRNGG

PPIIPRED:

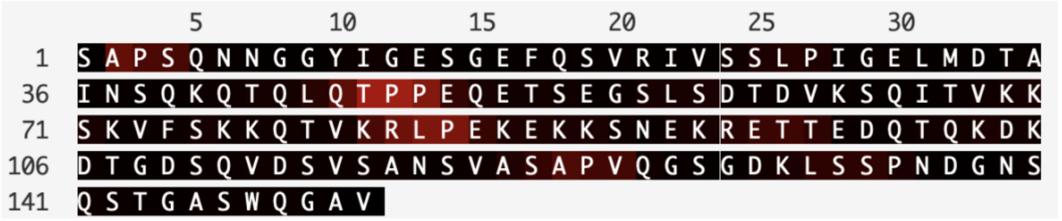

151 Domain II residues

7 Proline residues

6 α-Helix residues
